# An Immunomodulatory Peptide Dendrimer Inspired from Glatiramer Acetate

**DOI:** 10.1002/anie.202113562

**Published:** 2021-11-05

**Authors:** Dina Erzina, Alice Capecchi, Sacha Javor, Jean‐Louis Reymond

**Affiliations:** ^1^ Department of Chemistry, Biochemistry and Pharmaceutical Sciences University of Bern Freiestrasse 3 3012 Bern Switzerland

**Keywords:** dendrimers, Glatiramer Acetate, immunomodulation, multiple sclerosis, peptides

## Abstract

Glatiramer acetate (GA) is a random polypeptide drug used to treat multiple sclerosis (MS), a chronic autoimmune disease. With the aim of identifying a precisely defined alternative to GA, we synthesized a library of peptide dendrimers with an amino acid composition similar to GA. We then challenged the dendrimers to trigger the release of the anti‐inflammatory cytokine interleukin‐1 receptor antagonist (IL‐1Ra) from human monocytes, which is one of the effects of GA on immune cells. Several of the largest dendrimers tested were as active as GA. Detailed profiling of the best hit showed that this dendrimer induces the differentiation of monocytes towards an M2 (anti‐inflammatory) state as GA does, however with a distinct immune marker profile. Our peptide dendrimer might serve as starting point to develop a well‐defined immunomodulatory analog of GA.

## Graphical abstract

To identify an alternative to the polymeric peptide drug Glatiramer Acetate (GA) used to treat multiple sclerosis, we tested peptide dendrimers with a size and composition similar to that of GA for their ability to induce the release of the cytokine IL1‐Ra from primary human monocytes. The best dendrimer is as active as GA and is shown by fluorescence labelling to internalize into monocytes and to trigger a distinct anti‐inflammatory response.

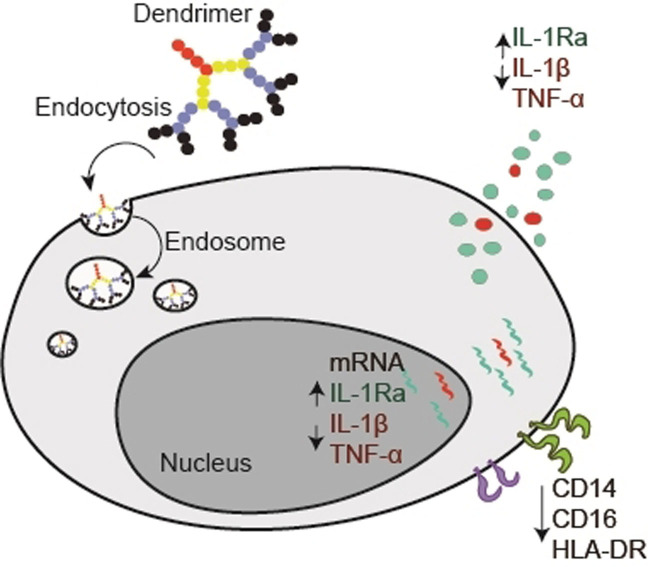

Glatiramer acetate (GA) is a random polypeptide of approximately 5–9 kDa composed of L‐alanine, L‐lysine, L‐glutamic acid and L‐tyrosine in a 4.2/3.4/1.4/1.0 ratio approximating the composition of myelin basic protein.[^1^, ^2^, ^3^, ^4^, ^5^] GA has been on the market since 1996 as one of the most successful first‐line treatments for multiple sclerosis (MS), a chronic autoimmune neurodegenerative disease.[^6^, ^7^, ^8^, ^9^] Notwithstanding the subsequent introduction of new modalities for targeting MS,[^10^, ^11^] GA remains a blockbuster drug.^[12]^ There are currently no second‐generation GA drugs and only a few generics of the original GA have been very recently introduced, probably due to the difficulty of replicating a polymeric preparation.[^4^, ^5^]

Although its mechanism of action is still debated, one of the main effects of GA is to induce the differentiation of immune cells towards an anti‐inflammatory rather than a pro‐inflammatory state, an effect which can be tracked by monitoring various cell surface markers and cytokines.[^13^, ^14^, ^15^] In view of the many successful applications of dendrimers[^16^, ^17^, ^18^, ^19^] including immunomodulation,[^20^, ^21^, ^22^, ^23^, ^24^, ^25^] here we asked the question whether a peptide dendrimer^[26]^ with a size and composition similar to GA might exhibit GA‐like effects and provide a new starting point for immunomodulation. Similar to immunomodulatory synthetic peptides[^27^, ^28^] and peptide dendrimers,^[29]^ we envisioned a peptide dendrimer with a precise amino acid sequence prepared by solid‐phase peptide synthesis.[^30^, ^31^, ^32^] As detailed below, these investigations led us to discover the immunomodulatory peptide dendrimer **1** (Figure 1).

**Figure 1:**
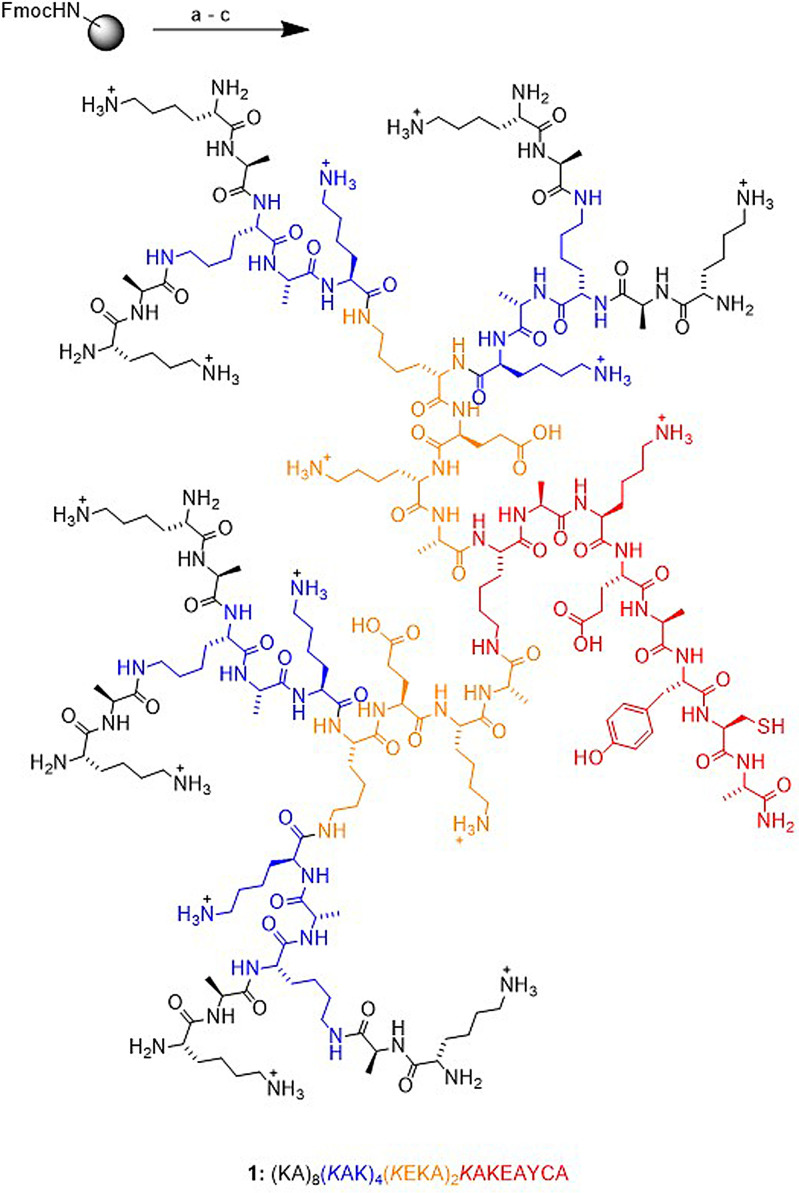
Synthesis and structural formula of peptide dendrimer **1**. SPPS conditions: (a) 20 % v/v piperidine in DMF, 5 min, 50 °C twice; (b) Fmoc‐amino acid (5 equiv./coupling site), Oxyma (7.5 equiv.), DIC (10 equiv.) in DMF, 15 min, 50 °C; (c) TFA/*i*‐Pr_3_SiH/DODT/H_2_O (94:2.5:2.5:1), 4 h at room temperature.

To initiate our search, we synthesized sixteen G2 peptide dendrimers sampled from virtual libraries with GA‐like composition (**6**–**22**, see supporting information for details). Considering that these G2 dendrimers were only reaching the lower MW end of GA, we designed an additional 10 GA‐like G2 and G3 dendrimers with longer sequences (**1**–**5**, Table 1, **23**–**30**, Table S1). We also prepared a 30‐mer and a 40‐mer linear random peptide with GA‐like composition (**31** and **32**, Table S1). In selected cases we masked *N*‐termini, which we assumed to be uncharged at neutral pH in analogy to similar polycationic dendrimers,^[31]^ by acetylation, or turned them into cysteine reactive groups by chloroacetylation or by acylation with monoethyl fumarate to mimic the MS drug dimethyl fumarate.[^33^, ^34^] This provided in total 62 test compounds.

**Table 1:** Synthesis, structural properties, and activity of selected peptide dendrimers.

ID	Sequence^[a]^	Yield, [%]^[b]^	MS calc./obs.^[c]^	Total ch.^[d]^	A.A. ratio, E/K/A/Y^[e]^	Act.^[f]^
**GA**	Random polypeptides	–	4.9–11 kDa	≈13	1.4/3.4/4.2/1	+
**1**	(KA)_8_(*K*AK)_4_(*K*EKA)_2_ *K*AKEAYCA‐NH_2_	61.9 (7.7)	4695.9470/4695.9625	12	3/15/17/1	+
Ac**1**	(AcKA)_8_(*K*AK)_4_(*K*EKA)_2_ *K*AKEAYCA‐NH_2_	53.9 (22.8)	5032.0315/5032.0448	12	3/15/17/1	−
**2**	(KAEKAYA)_4_(*K*EKYAKA)_2_ *K*EKYKA‐NH_2_	221 (25.6)	5447.0133/5447.0332	7	1/1/2.4/1	−
**3**	(AKA)_8_(*K*YEK)_4_(*K*EKA)_2_ *K*AKY‐OH	23.7 (9.0)	5775.3771/5775.3854	8	1.2/3/3.8/1	−
**4**	(KA)_8_(KKAKE)_4_(KYKAKA)_2_KAYKKA‐OH	127 (9.0)	6016.7929/6016.7972	17	1.3/7.3/6/1	+
Ac**4**	(AcKA)_8_(*K*KAKE)_4_(*K*YKAKA)_2_ *K*AYKKA‐OH	52.8 (19.9)	6352.8774/6352.8918	17	1.3/7.3/6/1	−
**5**	(AK)_8_(*K*AKAKY)_4_(*K*AKEYEY)_2_ *K*AKEYEY‐NH_2_	51.3 (5.8)	6916.1419/6916.1596	13	0.6/1.9/1.9/1	−
**33**	(KAK)_4_(*K*EKA)_2_ *K*AKEAYCA‐NH_2_	21.9 (14.1)	3102.8903/3102.8874	4	3/11/9/1	−
**34**	(AK)_8_(*K*AK)_4_(*K*EKA)_2_ *K*AKEAYCA‐NH_2_	23.2 (23.2)	4695.9470/4695.9469	12	3/15/17/1	+
**35**	(KK)_8_(*K*AK)_4_(*K*EKA)_2_ *K*AKEAYCA‐NH_2_	18.9 (18.9)	5152.4098/5152.4134	12	3/23/9/1	+
**36**	(KA)_8_(*K*KKAK)_4_(*K*EKA)_2_ *K*AKEAYCA‐NH_2_	28.0 (8.6)	5720.7067/5720.7070	20	3/23/17/1	+
Ac**36**	(AcKA)_8_(*K*KKAK)_4_(*K*EKA)_2_ *K*AKEAYCA‐NH_2_	28.6 (9.4)	6056.7912/6056.7988	20	3/23/17/1	+
Fum**36**	(FumKA)_8_(*K*KKAK)_4_(*K*EKA)_2_ *K*AKEAYCA‐NH_2_	27.5 (8.3)	6728.9602/6728.9614	20	3/23/17/1	+
**1‐1**	((KA)_8_(*K*AK)_4_(*K*EKA)_2_ *K*AKEAYCA‐NH_2_)_2_	7.9 (43.6)	9247.8040/9247.8422	24	3/15/17/1	+
D‐**1**	(ka)_8_(*k*ak)_4_(*k*eka)_2_ *k*akeayca‐NH_2_	56.8 (18.3)	4695.9470/4695.9606	12	3/15/17/1	−
*sr‐* **1**	(KA)_8_(*K*AK)_4_(*K*EKA)_2_ *K*AKEAYCA‐NH_2_	32.9 (7.6)	4624.9098/4624.9212	12	3/15/17/1	+
D‐**4**	(ka)_8_(*k*kake)_4_(*k*ykaka)_2_ *k*aykka‐OH	67.1 (16.7)	6015.8089/6015.8149	8	1.3/7.3/6/1	−
*sr‐* **4**	(KA)_8_(*K*KAKE)_4_(*K*YKAKA)_2_ *K*AYKKA‐OH	89.6 (15.6)	6016.7929/6016.8081	8	1.3/7.3/6/1	+
**1**Fl	(KA)_8_(*K*AK)_4_(*K*EKA)_2_ *K*AKEAYC(Fl)A‐NH_2_	6.5 (40.6)	5207.0373/5207.0391	12	3/15/17/1	+

[a] One‐letter code amino acids are used, *K* is the branched lysine residue, Ac (acetyl), ClAc (chloroacetyl), Fum (monoethylfumarate) cap the *N*‐terminus, OH is the carboxyl *C*‐terminus, NH_2_ is carboxamide *C*‐terminus, Fl is fluorescein diacetate 5‐succinimide. See Figure 1 for correspondence between linear notation and dendrimer structure [b] Isolated yields as trifluoroacetate salt after preparative RP‐HPLC purification. [c] ESI‐MS data. [d] Calculated formal net charge at neutral pH assuming cationic lysine side chains, anionic glutamate side chains and carboxyl *C*‐terminus, and neutral *N*‐termini. [e] Amino acid ratio without counting branching Lys. [f] Ability to induce IL‐1Ra on primary monocytes after 48 h of incubation in the presence of 25–50 μg mL^−1^ compound. See supporting information for a full list of all compounds synthesized and tested with their SMILES and activities.

As an activity screen we focused on interleukin‐1 receptor antagonist (IL‐1Ra), a cytokine released by circulating antigen presenting cells (APC) in response to GA.[^15^, ^35^] IL‐1Ra crosses the blood–brain barrier (BBB) and is therefore a possible mediator of GA action in the CNS since GA itself does not cross the BBB.^[36]^ To test our dendrimers, we quantified by immunoassay the release of IL‐1Ra from human primary monocytes of healthy donors, an easily accessible type of APC, stimulated or not by addition of lipopolysaccharide (LPS), which in our hands provided a reliable read‐out.^[15]^ We tested at defined weight/volume (25 and 50 μg mL^−1^ compound as typically reported for GA)[^15^, ^35^] to account for a possible activity increase with MW as often observed with dendrimers when using molarity. In the initial screen the levels of secreted IL‐1Ra remained below 1 ng mL^−1^ for all the smaller test compounds as well as with the linear peptide analogs of GA. However, two of the largest G3 dendrimers, **1** and **4**, induced IL‐1Ra release to a level comparable or higher than GA (Table 1, Table S1, Figure 2 and S2), with a concentration dependent effect in the range 12.5–100 μg mL^−1^ (Figure S3).

**Figure 2:**
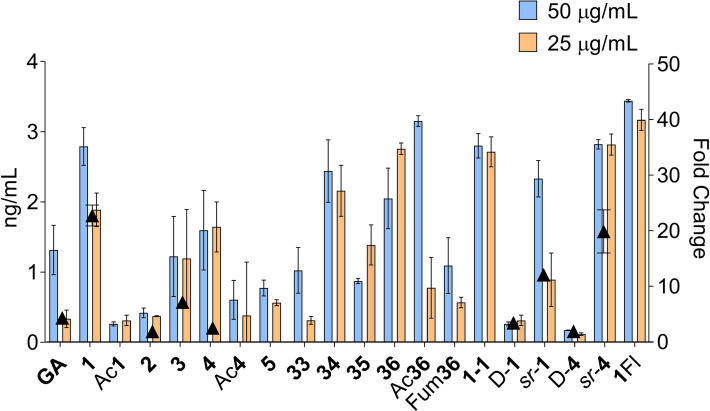
Cytokine level of IL‐1Ra in supernatants of primary human monocytes of healthy donors treated with peptide dendrimers, evaluated by ELISA (bars, left vertical axis) and mRNA levels (▴, right vertical axis). For ELISA 5×10^4^ cells 200 μL^−1^ in 96 well plates were incubated with 25–50 μg mL^−1^ dendrimer or GA for 48 h (mean ± SD, *n*=2, for p‐values computed relative to the inactive analog D‐**4**, see Table S3). For mRNA levels evaluation, 2×10^6^ cells 3 mL^−1^ in 6 well plates were incubated with 50 μg mL^−1^ dendrimer or GA for 18 h.

To assess the activity requirements for **1** and **4**, we performed a structure–activity relationship (SAR) study on the level of induced IL‐1Ra release measured by immunoassay, which we verified in selected cases at the mRNA level by RT‐qPCR (Figure 2). During this study we tested a selection of active and inactive dendrimers on PBMCs and found that they did not measurably affect viability, suggesting that any modulation of IL‐1Ra release indicated a specific effect (Figures S2).

Activity was abolished by *N*‐terminal modification of **1** and **4** (Ac**1**, Fum**1**, Ac**4**, Fum**4**, Table S1) as well as in dendrimer **39** (Table S2), a smaller G2 analog of the most active dendrimer **1**. On the other hand, activity was preserved in close analogs of **1** featuring an additional lysine residue in the G2 branch and the *N*‐termini free (**36**) or acylated (Ac**36**, Fum**36**), or with modified G3 branches either in reversed order (**34**) or containing only lysine (**35**). The disulfide bridged dimer **1‐1** was also active (Table 1, Table S2, Figure 2).

In terms of stereochemistry, activity was lost with D‐enantiomers D‐**1** and D‐**4**, similar to the inactivity reported for D‐enantiomeric GA.^[2]^ However, the stereorandomized analogs *sr‐* **1** and *sr‐* **4**, which are well‐defined mixtures of all possible diastereomers obtained by synthesis using racemic building blocks and used to test the effect of folding on activity,^[37]^ were as active as the pure L‐enantiomers, suggesting that secondary structures were not required for activity (Figure 2). Indeed, the circular dichroism (CD) spectra of **1** and **4** indicated an unordered conformation, while GA as well as the inactive GA‐like 40‐mer **32** were α‐helical (Figure S12).

The induction of IL‐1Ra by GA and dendrimers **1** and **4** was specific for monocytes and did not occur with lymphocytes (Figure S4). Furthermore, a survey of monocytes from five different healthy donors (HD) showed different levels IL‐1Ra secretion in response to GA and the dendrimers (Figure 3). For instance, three donors reacted stronger to dendrimers **1** and **4** than to GA (HD1, HD4, HD5), one stronger to GA (HD2), and one donor did not react significantly to the compounds (HD3). Similar donor dependent responses of cytokine release to GA have been reported for IL‐27.^[38]^

**Figure 3:**
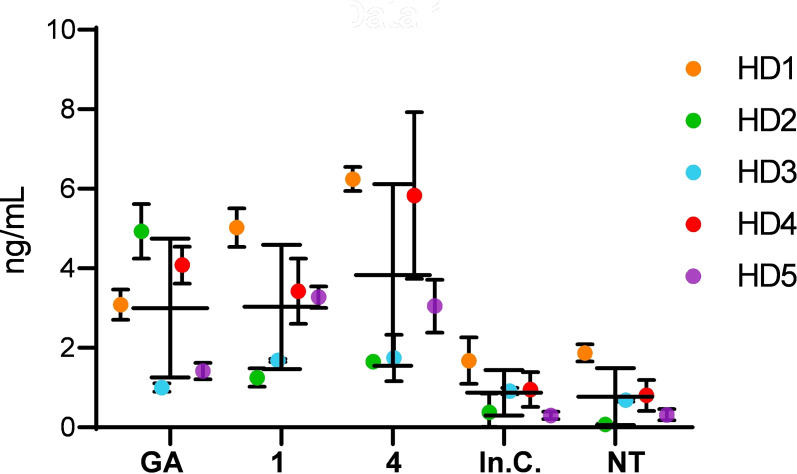
Healthy donor responses to GA, peptide dendrimers **1** and **4**, In.C.(inactive control, an inactive dendrimer from screening) and NT (no treatment) in IL‐1Ra secretion for 5 different donors. Each colour represents a different healthy donor (HD 1–5). Supernatants were analysed by ELISA after 48 h of incubation 5×10^4^ cells 200 μL^−1^ in 96 well plates. Concentration of GA and peptide dendrimers was 50 μg mL^−1^ Data is presented as a mean ± SD, *n*=2–3, p‐values computed relative to the untreated sample, See Table S4.

For closer characterization, we selected dendrimer **1** due to its smaller size and the presence of a cysteine residue that could be used for labelling. A time‐profile of cytokine release showed that dendrimer **1** significantly inhibited the production of the proinflammatory cytokine IL‐1β, which occurred in the first few hours in both inactivated and LPS activated monocytes (Figure S5a/b).^[39]^ Dendrimer **1** also inhibited the release of TNF‐α triggered upon the initial activation of monocytes by LPS (Figure S5c/d). Furthermore, dendrimer **1** induced the release of IL‐1Ra both with and without LPS activation, but in the latter case the effect was detected only after 18 h incubation (Figure S5e/f). These cytokine modulation effects suggested that dendrimer **1** shifted monocytes towards an anti‐inflammatory M2 phenotype.^[40]^

To further probe the immunomodulatory effects of dendrimer **1** in comparison to GA, we measured levels of CD14, CD16, CD68, CCR2 and HLA‐DR as typical markers of monocyte inflammatory activation, and of CD206 as marker of the M2 anti‐inflammatory phenotype (Figure 4 a–e, Figure S6–11).[^41^, ^42^, ^43^, ^44^, ^45^, ^46^, ^47^] Under non‐activated conditions, dendrimer **1** downregulated the innate immune response receptors CD14 (LPS receptor), CD16 (Fcγ receptor III), HLA‐DR (Human leukocyte antigen class II)^[48]^ and CD68 (peptide transport, antigen processing), indicative of a general immunosuppressive property (Figure 4 b). Dendrimer **1** also downregulated the chemokine receptor CCR2, which is often used as a marker for M1 (pro‐inflammatory state)[^49^, ^50^] and upregulated CD206, (Figure 4 a–c), a mannose receptor used as marker for M2 (anti‐inflammatory state).^[51]^ Similar but weaker effects occurred with analogs D‐**1** and Ac**1** although they did not induce IL‐1Ra release. By contrast, GA upregulated CD14, CD16 and CD68, but did not affect HLA‐DR, CCR2 or CD206. Under LPS‐activated conditions, the levels of most surface markers in non‐treated cells were slightly to strongly enhanced compared to non‐activated conditions, and generally downregulated by the dendrimers and GA alike (Figure 4 e).

**Figure 4:**
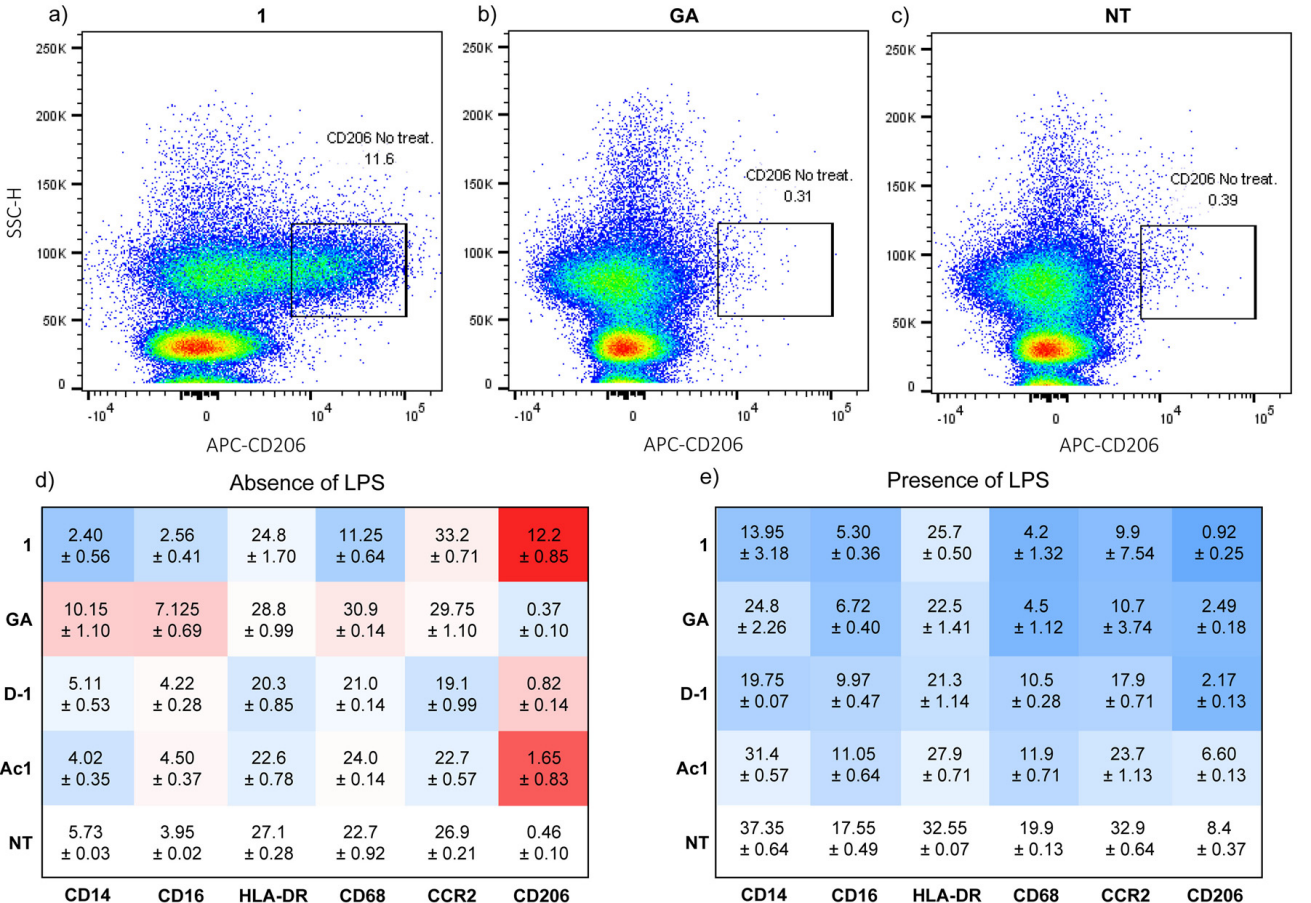
Flow cytometry dot plot showing the difference in CD206 expression on PBMC of a healthy donor in response to the treatment (50 μg mL^−1^) with **1** (a), GA (b), or without treatment (c) in the absence of LPS for 18 h. Heatmap of surface markers expression in response to treatment with active dendrimer **1**, GA, inactive dendrimers D‐**1** and Ac**1** in the absence (d) and presence of LPS (100 ng mL^−1^) (e). The color code indicates downregulation (blue) or upregulation (red) relative to no treatment (last line, NT, white). Data is presented as a mean ± SD, *n*=2 independent experiments. (See the SI for full FACS plots.).

In contrast to GA which is a polymeric mixture, our dendrimers are entirely well‐defined and therefore can be selectively labelled.^[52]^ Here we prepared **1**Fl, an analog of **1** bearing a fluorescein label at the dendrimer core, to directly visualize the extent of its interaction with monocytes. Dendrimer **1**Fl showed similar IL‐1Ra release activity as the unlabelled dendrimer **1** (Table 1 and Figure 2). Confocal imaging in the presence or absence of LPS showed that **1**Fl was mostly bound to the cell surface, with only partial localization in endosomes indicated by a punctuated pattern. This localization is consistent with an interaction at the cell surface to trigger a biological response (Figure 5).

**Figure 5:**
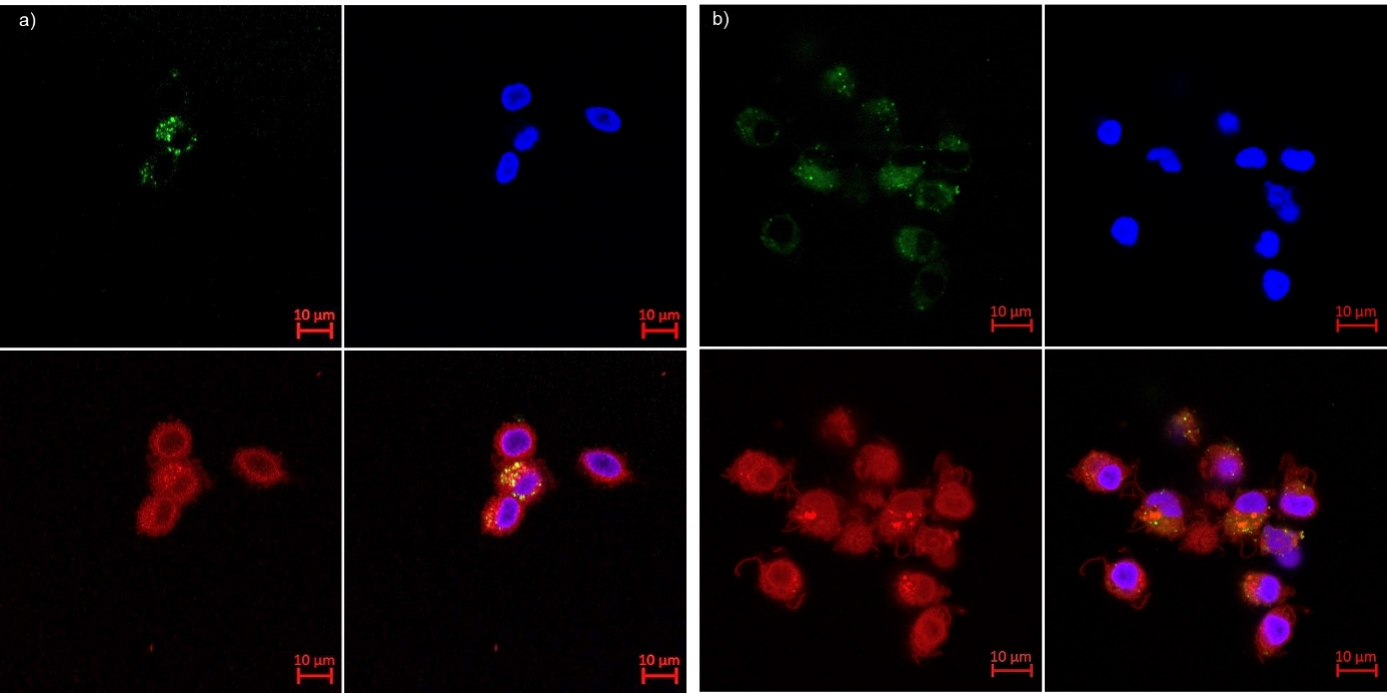
Confocal microscopy of primary human monocytes incubated for 48 h with 1Fl (50 μg mL^−1^) in the absence of LPS (a) (10^5^ cells 200 μL^−1^) or presence of LPS (100 ng mL^−1^) (b). Membrane is in red (CellMask Deep Red), nucleus is in blue (Hoechst33342), compounds are in green (Fluorescein).

Dendrimer **1** belonged to the largest dendrimers tested, in line with the size requirement for activity in GA. This can be appreciated in a tree‐map (TMAP)^[53]^ analysis based on the MAP4 molecular fingerprint,^[54]^ which showed that the designed dendrimer library spanned a broad range of sizes and sequence types (Figure 6). The TMAP analysis also illustrated that dendrimer **1** featured a particularly large number of positive charges from lysine side chains, which probably favoured cellular uptake by monocytes and might be necessary for activity. While **1** contains a higher fraction of lysines compared to GA, GA analogs lacking lysines have been reported to be inactive, underscoring the importance of cationic residues for GA as well.^[55]^

**Figure 6:**
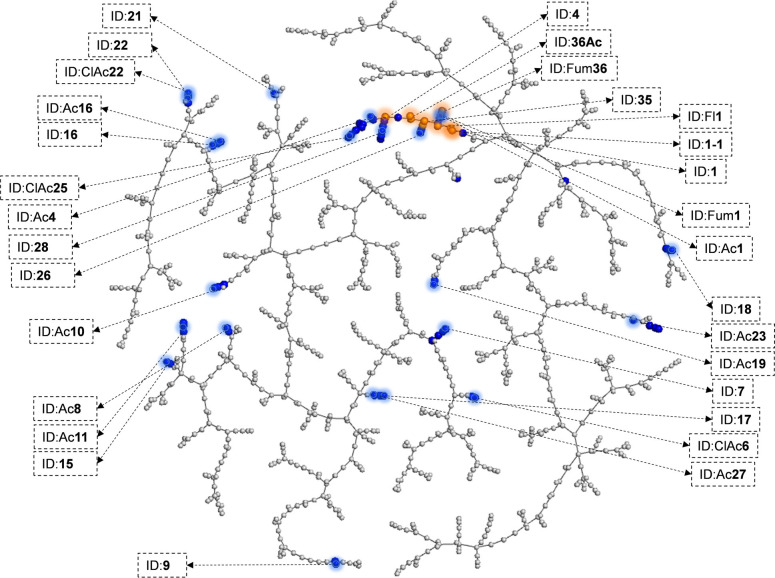
TMAP visualization of GA‐like peptide dendrimers. The TMAP was computed with MAP4 fingerprint similarity for the virtual library of G2 peptide dendrimers (1000, 16 of which were synthesized) and 52 manually designed analogues. Color‐code: grey=virtual library, orange=synthesized active, blue=synthesized inactive. An interactive TMAP is available at https://tm.gdb.tools/map4/glatiramer_analogs_1000_tmap/.

In summary, screening a focused library of peptide dendrimers with a GA‐like amino acid composition for induction of IL‐1Ra release from purified human monocytes led to the discovery of peptide dendrimer **1** as a new immunomodulatory compound. Dendrimer **1** steered monocytes towards an M2 anti‐inflammatory state, however with an immune marker profile substantially different from that of GA. Structure‐activity profiling showed that the activity of dendrimer **1** was strongly influenced by variations in amino acid sequence and stereochemistry. Considering that dendrimer **1** did not show measurable cellular toxicity and its exact sequence is perfectly defined, this compound provides a suitable starting point to develop new immunomodulatory compounds. To the best of our knowledge, this is the first example of an immunomodulatory molecule designed as an analog of GA.

## Conflict of interest

The authors declare no conflict of interest.

## Supporting information

As a service to our authors and readers, this journal provides supporting information supplied by the authors. Such materials are peer reviewed and may be re‐organized for online delivery, but are not copy‐edited or typeset. Technical support issues arising from supporting information (other than missing files) should be addressed to the authors.

Supporting Information

Supporting Information

Supporting Information
