# Supplementary material for: An Immunomodulatory Peptide Dendrimer Inspired from Glatiramer Acetate
Source: Angew Chem Int Ed Engl. 2021 Nov 5;60(50):26403–8. doi: 10.1002/anie.202113562 (PMC9298260; doi:10.1002/anie.202113562)

Angewandte Chemie
Eine Zeitschrift der Gesellschaft Deutscher Chemiker

Supporting Information

An Immunomodulatory Peptide Dendrimer Inspired from Glatiramer Acetate

*Dina Erzina, Alice Capecchi, Sacha Javor, and Jean-Louis Reymond**

anie_202113562_sm_miscellaneous_information.pdf
anie_202113562_sm_SI.csv
anie_202113562_sm_SI.zip

Supporting Information for:

Table of Contents:

Cheminformatics 3
TMAP visualization 4
Peptide Dendrimer Synthesis 6
Manual solid phase peptide synthesis 6
Automated solid phase peptide synthesis 6
Semi-automated solid phase peptide synthesis 7
Cleavage and Purification 8
Coupling of peptide dendrimers in solution 8
Synthesis and Activity Data (Tables S1-S2) 9
Cell culture and Isolation of human primary monocytes 12
Materials for bioassays 12
Extraction of peripheral blood mononuclear cells (PBMC) 12
Monocytes purification using magnetic beads labelling Isolation KIT II 13
Monocytes enrichment using Percoll gradient centrifugation 13
Cell Viability assay by AlamarBlue 13
Cytokine production 14
Enzyme-linked immunosorbent assay (ELISA) 14
mRNA Quantification 19
Confocal Microscopy 20
Membrane and nucleus staining 20
Flow Cytometry experiments 21
CD Spectroscopy 25
Synthesis, HPLC and MS Data for all Dendrimers 26

Cheminformatics

Random peptide and peptide dendrimer sequences were generated using the following templates (50,000 sequence per template, X = Lys, Ala, Tyr, Glu, *K* = branching lysine, Ac = N-terminal acetylation):

(i) G2 dendrimers: $(XXXXX)_4(BXXXXX)_2BXXXXX$;

(ii) acetylated G2 dendrimers: $(AcXXXXX)_4(BXXXXX)_2BXXXXX$;

In all cases, the four amino acids had an equal probability of being picked in their respective positions. To allow sequences of different lengths, X positions were also allowed to be empty with a probability of 50%. Then, to assess the difference in composition of a peptide from Glatiramer, we counted the number of Tyr (Y), Glu (E), Ala (A), and the non-branching Lys (K). Then we considered the number of Tyr as unit, and we calculated the ratio relative to Tyr of the other three amino acids. Next, for each amino acid, we calculated the absolute value of the difference between its relative ratio and the relative ratio of the same amino acid in Glatiramer. Finally, we summed the four values, and we obtained the difference in composition (DC, Equation 1) from Glatiramer.

$$P_{yr} = [\frac{A}{Y}, \frac{K}{Y}, \frac{E}{Y}, \frac{Y}{Y}]$$

$$G_{yr} = [4.2, 3.4, 1.4, 1.0]$$

$$DC = \sum_i |G_{yri} - P_{yri}| \quad \text{(Equation 1)}$$

The two resulting libraries were filtered based on their calculated composition, and only sequences with a DC from Glatiramer lower than ten were kept. For both libraries, the filtered sequences were clustered in 500 k-means clusters using Scikit-learn[1] and the macromolecule extended atom pair fingerprint (MXFP),[2] and the head of the cluster was picked. The procedure resulted 500 second-generation dendrimers of linear sequence length spanning from 13 to 17 and 500 second-generation acetylated dendrimers of linear sequence length spanning from 9 to 13. We then selected 8 G2 peptide dendrimers and 8 G2 acetylated peptide dendrimers by inspecting the cluster list sorted by GA-similarity for sequence diversity.

TMAP visualization

A TMAP was produced featuring of 1,052 peptide dendrimers comprising the virtual library of 500 selected G2 peptide dendrimers, 8 of which were synthesized, 500 acetylated G2 peptide dendrimers, 8 of which were synthesized, and an additional 52 analogues and manually designed sequences (Table S1). The SMILES (Simplified Molecular-Input Line-Entry System) for each dendrimer was produced from the sequence using the open-source code of the Peptide design genetic algorithm.[3] Note that among the synthesized compounds (Table S1) only the one with defined sequence were included in the visualization, excluding the four random sequence GA analogs **31**, **ClAc31**, **32** and **Ac32** for which a SMILES and a fingerprint cannot be computed. Then the MAP4 (MinHashed Atom Pair fingerprint of diameter 4) was calculated using the open-source MAP4 code.[4] The difference in composition was calculated as described above. Positive charges were calculated as the number of non-branching Lys, negative charges were calculated as and number Glu. A free *C*-termini was considered as an additional negative charge. Molecular weight (MW), was calculated using RDKit.[5] The MAP4 values were used to generate the TMAP (TreeMap)[6] layout using 32 LSH trees (*n*) and a 20 nearest neighbors graph (*k*), then the compounds were visualized using Faerun. Finally, the calculated properties, the sequence distance from Glatiramer, and the compound activity in the IL-1Ra based assay were used to color the resulting interactive map (available at: https://tm.gdb.tools/map4/glatiramer_analogs_1000_tmap/).

[1] F. Pedregosa, G. Varoquaux, A. Gramfort, V. Michel, B. Thirion, O. Grisel, M. Blondel, P. Prettenhofer, R. Weiss, V. Dubourg, J. Vanderplas, A. Passos, D. Cournapeau, M. Brucher, M. Perrot, É. Duchesnay, *J. Mach. Learn. Res.* **2011**, *12*, 2825−2830.

[2] A. Capecchi, M. Awale, D. Probst, J. L. Reymond, *Mol Inf* **2019**, *38*, 1900016.

[3] A. Capecchi, A. Zhang, J.-L. Reymond, *J. Chem. Inf. Model.* **2019**, DOI 10.1021/acs.jcim.9b01014.

[4] A. Capecchi, D. Probst, J.-L. Reymond, *J. Cheminformatics* **2020**, *12*, 43.

[5] "RDKit," can be found under https://www.rdkit.org/, **2020**.

[6] D. Probst, J.-L. Reymond, *ArXiv190810410 Cs* **2020**.

[7] U. Repnik, M. Knezevic, M. Jeras, *J. Immunol. Methods* **2003**, *278*, 283–292.

Materials and Reagents

All reagents, salts and buffers were used as purchased from Merck, Fluorochem Ltd, Iris Biotech Gmbh, GL Biochem. Amino acids were used as the following derivatives: Fmoc-Ala-OH, Fmoc-Glu(*t*-Bu)-OH, Fmoc-Cys(Trt)-OH, Fmoc-Lys(Boc)-OH, Fmoc-Lys(Fmoc)-OH, Fmoc-Tyr(*t*-Bu)-OH and purchased from Iris Biotech GmbH or GL Biochem. Tentagel S RAM resin was purchased from Rapp Polymere GmbH (loading: 0.24 mmol·g^{-1}). Rink Amide AM LL was purchased from Novabiochem (loading: 0.26 mmol·g^{-1}). Fmoc-Ala-Wang LL resin, Fmoc-Glu(*t*-Bu)-Wang LL resin, Fmoc-Lys(Boc)-Wang LL resin, Fmoc-Tyr(*t*-Bu)-Wang LL resin were purchased from Novabiochem and Merck (loading: 0.32 mmol·g^{-1}), resins were used for manual solid phase peptide synthesis, by CEM Liberty Blue Automated Microwave Peptide Synthesizer or by semi-automated peptide synthetizer. OxymaPure (ethyl cyanoglyoxylate-2-oxime) and DIC (*N*,*N*' -diisopropyl carbodiimide) was used for peptide coupling. Peptide dendrimers synthesis was performed manually in polypropylene syringes fitted with a polyethylene frit, a Teflon stopcock and stopper or automatically by CEM Liberty Blue Automated Microwave Peptide Synthesizer or glass syringes fitted with a polyethylene frit.

Analytical RP-HPLC was performed with an Ultimate 3000 Rapid Separation LC-MS System (DAD-3000RS diode array detector) using an Acclaim RSLC 120 C18 column (2.2 µm, 120 Å, 3×50 mm, flow 1.2 mL/min) from Dionex. Data recording and processing was done with Dionex Chromeleon Management System Version 6.80 (analytical RP-HPLC). All RP-HPLC were using HPLC-grade acetonitrile and Milli-Q deionized water. The elution solutions were: A Milli-Q deionized water containing 0.05% TFA; D Milli-Q deionized water/acetonitrile (10:90, v/v) containing 0.05% TFA. Preparative RP-HPLC was performed with a Waters automatic Prep LC Controller System containing the four following modules: Waters2489 UV/Vis detector, Waters2545 pump, Waters Fraction Collector III and Waters 2707 Autosampler. A Dr. Maisch GmbH Reprospher column (C18-DE, 100×30 mm, particle size 5 µm, pore size 100 Å, flow rate 40 mL/min) was used. Compounds were detected by UV absorption at 214 nm using a Waters 2487 Tunable Absorbance Detector. Data recording and processing was performed with Waters ChromScope version 1.40 from Waters Corporation. All RP-HPLC were using HPLC-grade acetonitrile and Milli-Q deionized water. The elution solutions were: A: Milli-Q deionized water containing 0.1% TFA; D: Milli-Q deionized water/acetonitrile (10:90, v/v) containing 0.1% TFA. MS spectra were recorded on a Thermo

Scientific LTQ OrbitrapXL. MS spectra were provided by the MS analytical service of the Department of Chemistry and Biochemistry at the University of Bern (group PD Dr. Stefan Schürch).

Peptide Dendrimer Synthesis

Manual solid phase peptide synthesis

Manual solid phase synthesis was performed in 10 mL polypropylene syringes with porous polyethylene filters and Teflon caps. Stirring of the reaction mixture at any given step was performed by attaching the closed syringe to a rotating axis. For synthesis 300 mg of TentaGel S RAM resin (loading: 0.24 mmol·g^{-1}) or Fmoc-Wang LL preloaded resin, (loading: 0.32 mmol·g^{-1}) was swollen in DCM for 20 min. Then, the following conditions were used:

Deprotection of Fmoc group

For deprotection at each step the Fmoc protecting group was removed with 8 mL of piperidine/DMF (1:4, v/v) for 30 min. After filtration the resin was washed with DMF (3 × 6 mL), MeOH (3 × 6 mL) and DCM (3 × 6 mL).

Coupling of Fmoc-protected aminoacids

For coupling 5 eq. of Fmoc-protected amino acid, 5 eq. OxymaPure and DIC 5 eq. in DMF were added to the resin and the reaction was stirred for 90 min. The couplings were repeated according to the generations and performed once for the 0th generation, twice for the 1st generation, three times for the 2nd generation, four times for 3rd generation. After filtration the resin was washed with DMF (3 × 6 mL), MeOH (3 × 6 mL) and DCM (3 × 6 mL).

Automated solid phase peptide synthesis

Automated microwave synthesis was performed with Liberty Blue CEM synthesizer under SPPS conditions at 0.25 mmol scale. Was used 180 mg Rink Amide AM LL (loading: 0.26 mmol·g^{-1}) resin or Fmoc-Ala-Wang LL resin, Fmoc-Glu(*t*-Bu)-Wang LL resin, Fmoc-Lys(Boc)-Wang LL resin, Fmoc-Tyr(*t*-Bu)-Wang LL resin (loading: 0.32 mmol·g^{-1}). The resin was swollen in DMF/DCM 50:50 for 15 min at R.T.

Deprotection of Fmoc group.

For deprotection at each step the Fmoc protecting group was removed with 5 mL of piperidine/DMF (1:4, v/v) for 2 min at 75 °C. After filtration, the resin was washed 5 times with 5 mL DMF.

Coupling of Fmoc-protected amino acids.

For coupling 5 eq. of Fmoc-protected amino acid, 5 eq. of Oxyma and 5 eq. of DIC all at a concentration of 0.2 M, were used as coupling reagents in 4 mL of DMF. The reaction was stirred for 5 minutes at 75 °C. The resin was then washed with 4 mL DMF 4 times. The couplings were repeated according to the generations and performed once for the 0th generation, twice for the 1st generation, four times for the 2nd generation. For 3rd generation synthesis was performed manually.

Semi-automated solid phase peptide synthesis

Semi-automated synthesis was performed with an in-house built synthetiser consisting of a heating element, keeping the temperature at 50 °C, glass reaction vessels and a vacuum operated filtration system. For each synthesis 300 mg of Rink Amide AM LL (loading: 0.26 $mmol \cdot g^{-1}$) resin or Fmoc-Ala-Wang LL resin, Fmoc-Glu(*t*-Bu)-Wang LL resin, Fmoc-Lys(Boc)-Wang LL resin, Fmoc-Tyr(*t*-Bu)-Wang LL resin (loading: 0.32 $mmol \cdot g^{-1}$) was used. The resin was swollen in DMF/DCM 50:50 for 15 min at R.T. During all the steps the resin was mixed by passing N_2 through the syringes.

Deprotection of Fmoc group.

For deprotection at each step the Fmoc protecting group was removed with 5 mL of piperidine/DMF (1:4, v/v) for 5 min at 50 °C twice. After filtration, the resin was washed 5 times with 5 mL DMF.

Coupling of Fmoc-protected amino acids.

For coupling 5 eq. of Fmoc-protected amino acid, 5 eq. of Oxyma and 5 eq. of DIC all at a concentration of 0.2 M, were used as coupling reagents in 4 mL of DMF. The reaction was stirred for 15 minutes at 50 °C. The resin was then washed with 4 mL DMF 4 times. The couplings were repeated according to the generations and performed twice for the 0-2nd generation, four times for the 3rd generation.

Cleavage and Ppurification

The cleavage was carried out by treating the resins with 7 mL of TFA/*i*-Pr_3SiH/DOTT/H2O (94:2.5:2.5:1, v/v/v/v) solution for 4 h. The peptide solutions were precipitated with 40 mL of TBME, centrifuged for 10 min at 3500 rpm, evaporated and dried under high vacuum for 60 min. The crude was then dissolved in a H_2O/CH_3CN (10/1, v/v) mixture, some drops of MeOH added when needed and purified by preparative RP-HPLC. The fractions of the crudes were then lyophilized. Yields are given as SPPS total yields. In all cases, yields are calculated for the corresponding TFA salts.

Coupling of peptide dendrimers in solution

Fluorophore labelled peptide dendrimers.

Peptide dendrimer (1 eq., 5-10 mg) was solubilized NH_4HCO_3 (50 mM) solution. Then, fluorescein diacetate 5-maleimide (Fl, 1.1 eq., 0.5-1 mg), 7-diethylamino-3-(4-maleimidophenyl)-4-methylcoumarin (Coum 1.1 eq, 0.5-1 mg) were solubilized in 500 µL acetonitrile and added dropwise to the stirring solution. Reaction stirred at RT for 2 h, lyophilized and purified by preparative HPLC. Yields are given for the coupling step. In all cases, yields are calculated for the corresponding TFA salts.

Dimerization of peptide dendrimers.

Peptide dendrimer (2 eq., 10-20 mg) was solubilized NH_4HCO_3 (50 mM) solution. Then, reaction stirred for 24 h, lyophilized and purified by preparative HPLC. Yield is given for the coupling step and calculated for the corresponding TFA salt.

Synthesis and Activity Data (Tables S1-S2)

Table S1. Synthesis, structural properties, and activity of initial library.

ID	Sequence[a]	Yield, mg (%)[b]	MS calc./obs.[c]	Tot. ch.[d]	A.A. ratio, E/K/A/Y[e]	Act.[f]
**1**	(KA)$_8$(*K*AK)$_4$(*K*EKA)$_2$*K*AKEAYCA-NH$_2$	61.9 (7.7)	4695.9470/4695.9625	12	3/15/17/1	+
Ac**1**	(AcKA)$_8$(*K*AK)$_4$(*K*EKA)$_2$*K*AKEAYCA-NH$_2$	53.9 (22.8)	5032.0315/5032.0448	12	3/15/17/1	-
Fum**1**	(FumKA)$_8$(*K*AK)$_4$(*K*EKA)$_2$*K*AKEAYCA-NH$_2$	47.6 (22.9)	5704.2005/5704.2057	12	3/15/17/1	-
**2**	(KAEKAYA)$_4$(*K*EKYAKA)$_2$*K*EKYKA-NH$_2$	221 (25.6)	5447.0133/5447.0332	7	1/1/2.4/1	-
**3**	(AKA)$_8$(*K*YEK)$_4$(*K*EKA)$_2$*K*AKY-OH	23.7 (9)	5775.3771/5775.3854	8	1.2/3/3.8/1	-
Ac**3**	(AcAKA)$_8$(*K*YEK)$_4$(*K*EKA)$_2$*K*AKY-OH	20.3 (9)	6111.4616/6111.4644	8	1.2/3/3.8/1	-
ClAc**3**	(ClAcAKA)$_8$(*K*YEK)$_4$(*K*EKA)$_2$*K*AKY-OH	20.9 (9)	6383.1499/6384.1461	8	1.2/3/3.8/1	-
**4**	(KA)$_8$(KKAKE)$_4$(KYKAKA)$_2$KAYKKA-OH	127 (9.0)	6016.7929/6016.7881	17	1.3/7.3/6/1	+
Ac**4**	(AcKA)$_8$(*K*KAKE)$_4$(*K*YKAKA)$_2$*K*AYKKA-OH	52.8 (19.9)	6352.8774/6352.8813	17	1.3/7.3/6/1	-
Fum**4**	(FumKA)$_8$(*K*KAKE)$_4$(*K*YKAKA)$_2$*K*AYKKA-OH	51.8 (18.1)	7025.0465/7025.0531	17	1.3/7.3/6/1	-
**5**	(AK)$_8$(*K*AKAKY)$_4$(*K*AKEYEY)$_2$*K*AKEYEY-NH$_2$	51.3 (5.8)	6916.1419/6916.1596	13	0.6/1.9/1.9/1	-
**6**	(KA)$_4$(*K*AYE)$_2$*K*K-OH	77.2 (35)	2053.2048/2053.2054	2	1/2.5/3/1	
Ac**6**	(AcKA)$_4$(*K*AYE)$_2$*K*K-OH	26.6 (18)	2221.2471/2221.2492	2	1/2.5/3/1	-
ClAc**6**	(ClAcKA)$_4$(*K*AYE)$_2$*K*K-OH	106.0 (40)	2357.0912/2358.0922	2	1/2.5/3/1	-
**7**	(KA)$_4$(*K*EYA)$_2$*K*KAK-OH	24.8 (20)	2252.3369/2252.3399	3	1/3/3.5/1	-
Ac**7**	(AcKA)$_4$(*K*EYA)$_2$*K*KAK-OH	10.8 (10)	2420.3791/2420.3812	3	1/3/3.5/1	-
ClAc**7**	(ClAcKA)$_4$(*K*EYA)$_2$*K*KAK-OH	9.3 (8)	2556.2232/2556.2283	3	1/3/3.5/1	-
**8**	(AK)$_4$(*K*KYE)$_2$*K*AAA-OH	12.2 (10)	2252.3369/2252.3332	3	1/3/3.5/1	-
Ac**8**	(AcAK)$_4$(*K*KYE)$_2$*K*AAA-OH	10.4 (9)	2420.3791/2420.3770	3	1/3/3.5/1	-
**9**	(AYK)$_4$(*K*E)$_2$*K*KAA-OH	34.8 (28.4)	2379.3315/2379.3345	2	0.5/1.25/1.5/1	-
Ac**9**	(AcAYK)$_4$(*K*E)$_2$*K*KAA-OH	50.2 (28.4)	2547.3315/2547.3775	2	0.5/1.25/1.5/1	-
**10**	(AKA)$_4$(*K*KEY)$_2$*K*E-OH	24.3 (17.6)	2452.4166/2452.4192	2	1.5/3/4/1	-
Ac**10**	(AcAKA)$_4$(*K*KEY)$_2$*K*E-OH	49.3 (31.1)	2620.4588/2620.4612	2	1.5/3/4/1	-
ClAc**10**	(ClAcAKA)$_4$(*K*KEY)$_2$*K*E-OH	43.4 (32.3)	2756.3029/2756.3066	2	1.5/3/4/1	-
**11**	(AKA)$_4$(*K*EK)$_2$*K*YEY-OH	34.2 (26)	2452.4166/2452.4208	2	1.5/3/4/1	-
Ac**11**	(AcAKA)$_4$(*K*EK)$_2$*K*YEY-OH	25.0 (21)	2620.4588/2620.4626	2	1.5/3/4/1	-
ClAc**11**	(ClAcAKA)$_4$(*K*EK)$_2$*K*YEY-OH	10.2 (8)	2756.3029/2756.3050	2	1.5/3/4/1	-
**12**	(KAA)$_4$(*K*EKA)$_2$*K*EKA-NH$_2$	169.5 (49.3)	2650.5646/2650.5679	4	3/7/11/0	-
ClAc**13**	(ClAcK)$_4$(*K*AYE)$_2$*K*AKYAEA-NH$_2$)	159.6 (46.1)	2575.2079/2579.1768	4	1/1.7/1.7/1	-
**14**	(KYA)$_4$(*K*EA)$_2$*K*KKA-OH	87.6 (39)	2578.4635/2578.4656	3	0.5/1.5/1.75/1	-
Ac**14**	(AcKYA)$_4$(*K*EA)$_2$*K*KKA-OH	88.0 (43)	2746.5058/2746.5094	3	0.5/1.5/1.75/1	-
ClAc**14**	(ClAcKYA)$_4$(*K*EA)$_2$*K*KKA-OH	65.3 (31)	2882.3499/2882.3549	3	0.5/1.5/1.75/1	-
Ac**15**	(AcAKA)$_4$(*K*EY)$_2$*K*YKK-NH$_2$	31.7 (49)	2653.4956/2653.4904	4	0.7/2/2.7/1	-
Fum**15**	(FumAKA)$_4$(*K*EY)$_2$*K*YKK-NH$_2$	15.0 (23.6)	2485.4533/2485.4561	4	0.7/2/2.7/1	-
**16**	(AKE)$_4$(*K*AYA)$_2$*K*KKK-NH$_2$	13.5 (11)	2708.5701/2708.5633	3	2/3.5/4/1	-
Ac**16**	(AcAKE)$_4$(*K*AYA)$_2$*K*KKK-NH$_2$	13.8 (13)	2876.6124/2876.6340	3	2/3.5/4/1	-
Fum**16**	(FumAKE)$_4$(*K*AYA)$_2$*K*KKK-NH$_2$	13.5 (11)	3212.6969/3212.7355	3	2/3.5/4/1	-
**17**	(K)$_4$(*K*AYAEY)$_2$*K*AKAEYA-NH$_2$	108.2 (43.5)	2741.4905/2741.4934	2	0.6/1/1.4/1	-
**18**	(KAE)$_4$(*K*AEK)$_2$*K*YYA-OH	49.3 (30.0)	2768.5072/2768.5122	-1	3/3/3.5/1	-
Ac**18**	(AcKAE)$_4$(*K*AEK)$_2$*K*YYA-OH	74.1 (34.1)	2936.5495/2936.5543	-1	3/3/3.5/1	-
ClAc**18**	(ClAcKAE)$_4$(*K*AEK)$_2$*K*YYA-OH	91.6 (33.8)	3072.3936/3072.3961	-1	3/3/3.5/1	-

Ac**19**	(AcKAA)$_4$(*K*YYE)$_2$*K*AY-NH$_2$	31.7 (30)	2794.4694/2794.4734	2	0.4/0.8/1.8/1	-
AcCl**19**	(ClAcKAA)$_4$(*K*YYE)$_2$*K*AY-NH$_2$	29.4 (26)	2930.3135/2930.3170	2	0.4/0.8/1.8/1	-
Ac**20**	(AcKAK)$_4$(*K*AEY)$_2$*K*AEA-OH	3.0 (2)	2876.6488/2876.6463	4	1.5/4/4/1	-
**21**	(AKK)$_4$(*K*YEK)$_2$*K*KEA-NH$_2$	168.3 (49.3)	2650.5646/2650.5662	8	1.5/5.5/2.5/1	-
**22**	(AKE)$_4$(*K*KAY)$_2$*K*YKY-NH$_2$	25.4 (18.6)	2892.6225/2892.6243	3	1/1.75/1.5/1	-
Ac**22**	(AcAKE)$_4$(*K*KAY)$_2$*K*YKY-NH$_2$	35,6 (27.9)	3060.6648/3060.6693	3	1/1.75/1.5/1	-
ClAc**22**	(ClAcAKE)$_4$(*K*KAY)$_2$*K*YKY-NH$_2$	8.9 (7)	3196.5059/3196.5148	3	1/1.75/1.5/1	-
**23**	(KYA)$_4$(*K*AE)$_2$*K*AEYKCA-NH$_2$	127.1(29.5)	2913.5687/2915.5439	2	0.6/1/1.6/1	-
**24**	(KAA)$_4$(*K*EY)$_2$*K*AKEYEYA-NH$_2$	180 (41.1)	2920.5811/2920.5847	1	1/1.25/2.5/1	-
ClAc**25**	(ClAcK)$_4$(*K*EAKAKEY)$_2$*K*AKEYEY-OH	159.9 (22.8)	3640.7309/3640.7417	2	1.5/2.3/1.3/1	-
**26**	(AK)$_4$(*K*AEAKAKE)$_2$*K*KEYEYCA-NH$_2$	138 (39.1)	3865.0924/3865.1068	3	3/4.5/5.5/1	-
**26**Coum	(AK)$_4$(*K*AEAKAKE)$_2$*K*KEYEYC(Coum)A-NH$_2$	6.1 (55.3)	4267.2504/4267.2627	8	3/4.5/5.5/1	-
Ac**27**	(AcKEAKY)$_4$(*K*KYEA)$_2$*K*EKAYKA-NH$_2$	76.6 (34.3)	4719.5316/4719.5442	5	1/1.7/1.1/1	-
ClAc**27**	(ClAcKEAKY)$_4$(*K*KYEA)$_2$*K*EKAYKA-NH$_2$	56.7 (24.8)	4855.3757/4857.3959	5	1/1.7/1.1/1	-
**28**	(YAKAKE)$_4$(*K*AYKAKA)$_2$*K*AYKKA-NH$_2$	169.6 (23.4)	4988.8484/4988.8659	10	0.6/2/2.3/1	-
**29**	(YKAKAKY)$_4$(*K*EAKAKY)$_2$*K*AKEYEY-OH	154(12.3)	5976.3226/5976.3381	12	0.3/1.4/1.1/1	-
**30**	(KAA)$_8$(*K*KAKAK)$_4$(*K*AAKKY)$_2$*K*EKAKCA-OH	17.0 (2.6)	6934.4362/6934.4379	24	0.5/13/15/1	-
**31**	(X)$_{30}$-NH$_2$	46,3		3		-
ClAc**31**	ClAc(X)$_{30}$-NH$_2$	67,2		3		-
**32**	(X)$_{40}$-NH$_2$	34		4		-
Ac**32**	Ac(X)$_{40}$-NH$_2$	62,4		4		-

[a] One-letter code amino acids are used, *K* is the branched lysine residue, Ac (acetyl), ClAc (chloroacetyl), Fum (monoethylfumarate) cap the *N*-terminus, OH is the carboxyl *C*-terminus, NH_2 is carboxamide *C*-terminus, Coum is a conjugate with 7-diethylamino-3-(4'-maleimidylphenyl)-4-methylcoumarin. [b] Isolated yields as trifluoroacetate salt after preparative RP-HPLC purification. [c] ESI-MS data. [d] Charge of a dendrimer at neutral pH. [e] Amino acid ratio without counting branching Lys. [f] Ability to induce IL-1Ra on primary monocytes after 48 h of incubation.

Table S2. Synthesis, structural properties, and activity of analogs of **1** and **4**.

ID	Sequence[a]	Yield, mg (%)[b]	MS calc./obs.[c]	Tot. ch.[d]	A.A. ratio, E/K/A/Y[e]	Act.[f]
**33**	$(KAK)_4(\mathit{K}EKA)_2\mathit{K}AKEAYCA\text{-}NH_2$	21.9 (14.1)	3102.8903/3102.8874	20	3/11/9/1	-
**34**	$(AK)_8(\mathit{K}AK)_4(\mathit{K}EKA)_2\mathit{K}AKEAYCA\text{-}NH_2$	23.2 (23.2)	4695.9470/4695.9469	12	3/15/17/1	+
**35**	$(KK)_8(\mathit{K}AK)_4(\mathit{K}EKA)_2\mathit{K}AKEAYCA\text{-}NH_2$	18.9 (18.9)	5152.4098/5152.4134	12	3/23/9/1	+
**36**	$(KA)_8(\mathit{K}KKAK)_4(\mathit{K}EKA)_2\mathit{K}AKEAYCA\text{-}NH_2$	28.0 (8.6)	5720.7067/5720.7093	20	3/23/17/1	+
Ac**36**	$(AcKA)_8(\mathit{K}KKAK)_4(\mathit{K}EKA)_2\mathit{K}AKEAYCA\text{-}NH_2$	28.6 (9.4)	6056.7912/6056.7988	20	3/23/17/1	+
Fum**36**	$(FumKA)_8(\mathit{K}KKAK)_4(\mathit{K}EKA)_2\mathit{K}AKEAYCA\text{-}NH_2$	27.5 (8.3)	6728.9602/6728.9614	20	3/23/17/1	+
**1-1**	$(KA)_8(\mathit{K}AK)_4(\mathit{K}EKA)_2\mathit{K}AKEAYCA\text{-}NH_2)_2$	7.9 (43.6)	9247.8040/9247.8422	24	3/15/17/1	+
D-**1**	$(ka)_8(\mathit{k}ak)_4(\mathit{k}eka)_2\mathit{k}akeayca\text{-}NH_2$	56.8 (18.3)	4695.9470/4695.9606	12	3/15/17/1	-
D-**4**	$(ka)_8(\mathit{k}kake)_4(\mathit{k}ykaka)_2\mathit{k}aykka\text{-}OH$	67.1 (16.7)	6015.8089/6015.8149	8	1.3/7.3/6/1	-
sr-**1**	$(KA)_8(\mathit{K}AK)_4(\mathit{K}EKA)_2\mathit{K}AKEAYCA\text{-}NH_2$	32.9 (7.6)	4624.9098/4624.9212	12	3/15/17/1	+
sr-**4**	$(KA)_8(\mathit{K}KAKE)_4(\mathit{K}YKAKA)_2\mathit{K}AYKKA\text{-}OH$	89.6 (15.6)	6016.7929/6016.8081	8	1.3/7.3/6/1	+
**1**Fl	$(KA)_8(\mathit{K}AK)_4(\mathit{K}EKA)_2\mathit{K}AKEAYC(Fl)A\text{-}NH_2$	6.5 (40.6)	5207.0373/5207.0391	12	3/15/17/1	+

[a] One-letter code amino acids are used, *K* is the branched lysine residue, Ac (acetyl), ClAc (chloroacetyl), Fum (monoethylfumarate) cap the *N*-terminus, OH is the carboxyl *C*-terminus, NH_2 is carboxamide *C*-terminus, Fl is fluorescein diacetate 5-succinimide. [b] Isolated yields as trifluoroacetate salt after preparative RP-HPLC purification. [c] ESI-MS data. [d] Charge of a dendrimer at neutral pH. [e] Amino acid ratio without counting branching Lys. [f] Ability to induce IL-1Ra on primary monocytes after 48 h of incubation.

Cell culture and Isolation of human primary monocytes

Materials for bioassays

Glatiramer acetate (GA) was purchased from Brunschwig AG. Ficoll-Paque PLUS solution was purchased from GE Healthcare. Hanks` Balanced Salt Solution (HBSS) was purchased from Gibco. RPMI-1640 Medium, heat-inactivated Fetal Bovine Serum (FBS), streptomycin, penicillin, PBS tablets, Lipopolysaccarides from Escherichia coli O111:B4, mRNA extraction Single Cell RNA Purification Kit, AlamarBlue, Percoll density gradient, BSA extra pure, DEPC, Poly-L-Lysine were purchased from Sigma Aldrich. dNTP Mix 10 mM each, Superscript IV Reverse Transcriptase, RNaseOU Recombinant Ribonuclease Inhibitor, Random Hexamers, PowerUp™ SYBR™ Green Master Mix, IL-1Ra ELISA kit, Hoechst 33258, Cell Mask Deep Red were purchased from Termo Fisher Scientific. Pre-Separation filters, 30 µm, Classical Monocyte Isolation Kit, CD14-FITC, Anti-Biotin-PE, autoMACS Rinsing Solution, MACS BSA Stock Solution, LC MidiMACS Separator, MACS MultiStand, LS Columns were purchased from MACS, Miltenyi Biotec. PE Mouse Anti-Human CD14, BV605 Mouse Anti-Human CD16 Clone 3G8, PE-Cy™7 Mouse Anti-Human CD68 Clone, Y1/82A, BV711 Mouse Anti-Human CCR2 (CD192) LS132.1D9, BV421 Rat Anti-Human CX3CR1 Clone, 2A9-1, APC Mouse Anti-Human CD206 Clone 19.2, BV421 Mouse Anti-Human HLA-DR, DP, DQ, Clone Tu39 were purchased from BD Bioscieces. Glycergel Mounting Medium was purchased from Agilent. 8 Chambered Coverglass System CellVis was purchased from IBL Baustoff+Labor GmbH.

Extraction of peripheral blood mononuclear cells (PBMC)

Anticoagulant-treated blood was obtained from Interregional Blood Transfusion SCR Ltd. Bern in blood bags 45 mL each. In accordance with the ethical committee of the Interregionale Blutspende SRK AG obtained informed consent from the healthy donors, who are thus informed that part of their blood will be used for research purposes. Blood was diluted twice with HBSS and carefully overlayed on 20 mL of the Ficoll-Paque PLUS medium (1.077 g/ml). After centrifugation (400×g, 30 min, 20 °C) buffy coat at the interface was collected, washed with 40 mL of RPMA-1640 supplemented with 10% heat-inactivated FBS, 50 g/mL streptomycin, 50 U/mL penicillin, 2 mM glutamine, (RPMI supplemented). After centrifugation (200×g, 7 min, 20 °C) sedimented cells were diluted with RPMI-1640 supplemented and overlayed for second time over 20 mL of the Ficoll-Paque PLUS medium

and centrifuged at the same conditions. PBMC collected from the interface were washed 3 times with supplemented RPMI.

Monocytes purification using magnetic beads labelling Isolation KIT II

Monocytes were purified from whole PBMC by negative selection, all procedures were carried out according to manufacturer protocol. Briefly, the non-monocytes were labeled with a cocktail of biotin-conjugated monoclonal antibodies as a primary labeling reagent. Then as a secondary labeling reagent were used anti-biotin microbeads. The mixture was run through the MACS column (MACS, Miltenyi Biotech) in the magnetic field of a MACS Separator (MACS, Miltenyi Biotech). In this case, non-monocytes are retained in the column and the unlabeled monocytes pass through the column. The purity of the monocytes was checked by fluorescent microscopy counterstaining with CD14-FITC. In the same way, the presence of the non-monocytes can be verified by staining with fluorochrome-conjugated anti-biotin antibody (Anti-Biotin-PE, AntiBiotin-APC).

Monocytes enrichment using Percoll gradient centrifugation

Monocytes enriched fraction was obtained from PBMC by hyper-osmotic Percoll solution[7]. Briefly, for 100 mL of solution, 48.5 mL of Percoll, 41.5 mL of water and 10.0 mL of 1.6 M NaCl were mixed. 150-200×10^6cells were overlayed over 10 min of density medium and centrifuged at 580 g for 15 min. Cells at the interface were collected and washed 2 times with RPMI supplemented.

Cell Viability assay by AlamarBlue

PBMC (5×10^4cells/200 µL; 96-well plates) were incubated in presence of peptide dendrimers (100 µg/mL, 50 µg/mL, 25 µg/mL and 12.5 µg/mL) for 24 h in in RPMI-1640 medium supplemented with 10% heat-inactivated FBS, 50 µg/mL streptomycin, 50 U/mL penicillin, 2 mM L-glutamine, and 5 µg/mL. After the incubation 25µL of AlamarBlue was added to each well and cells were incubated for additional 12h. Then, plates were measured on a Tecan Infinite M1000 Pro plate reader at λ_{ex}= 560 nm and λ_{em}= 590 nm and value normalized to the one of untreated cells.

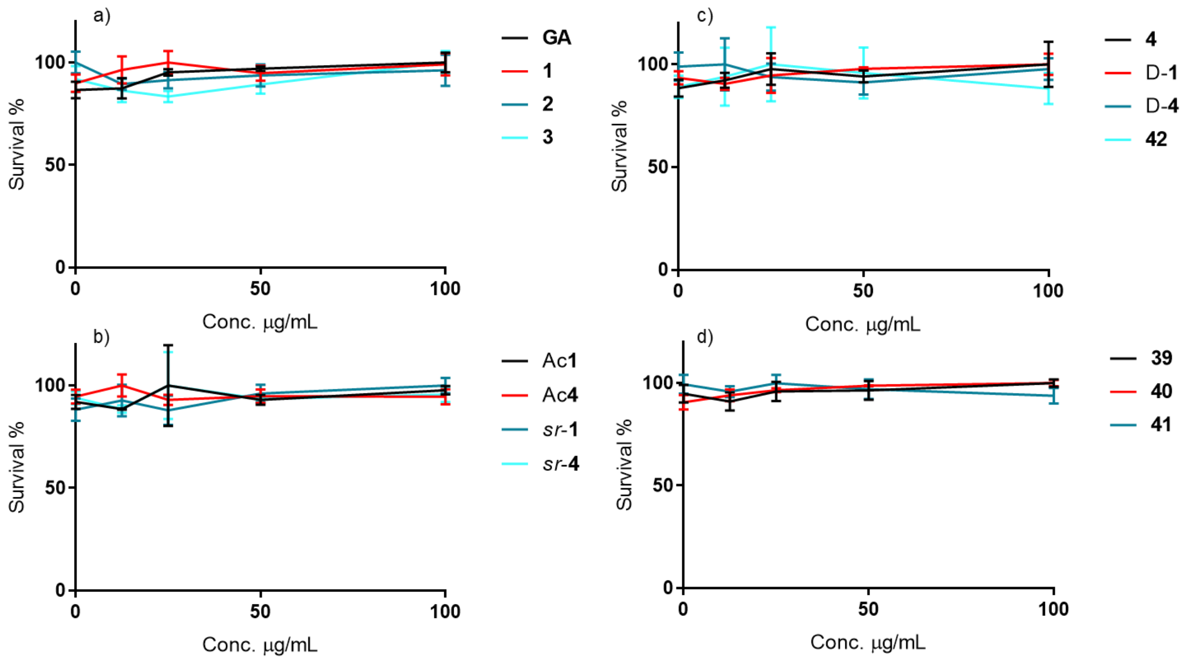

Figure S1. Viability of PBMC after 24h of incubation in presence of peptide dendrimers in a range of concentrations (100-12.5 µg/mL) $5x10^4$ cells/200µL in 96 well plates, were incubated with dendrimers or GA. (Normalized rows means ± SD, n = 3 different experiments). After incubation 50 µl of 50% AlamarBlue was added in supplemented medium for additional 12 h, then fluorescence was measured according to manufacture protocol.

Cytokine production

Enzyme-linked immunosorbent assay (ELISA)

Monocytes (5×10^4 cells/200 µL well; 96-well plates) were preincubated for 1 h with or without compounds in RPMI-1640 medium supplemented with 10% heat-inactivated FBS, 50 µg/mL streptomycin, 50 U/mL penicillin, 2 mM L-glutamine, and 5 µg/mL polymyxin B sulfate and then cultured for 48 h in the presence or absence of LPS (100 ng/mL) (2). The production of IL-1Ra was measured in culture supernatants by commercially available enzyme immunoassay according to manufacture protocol.

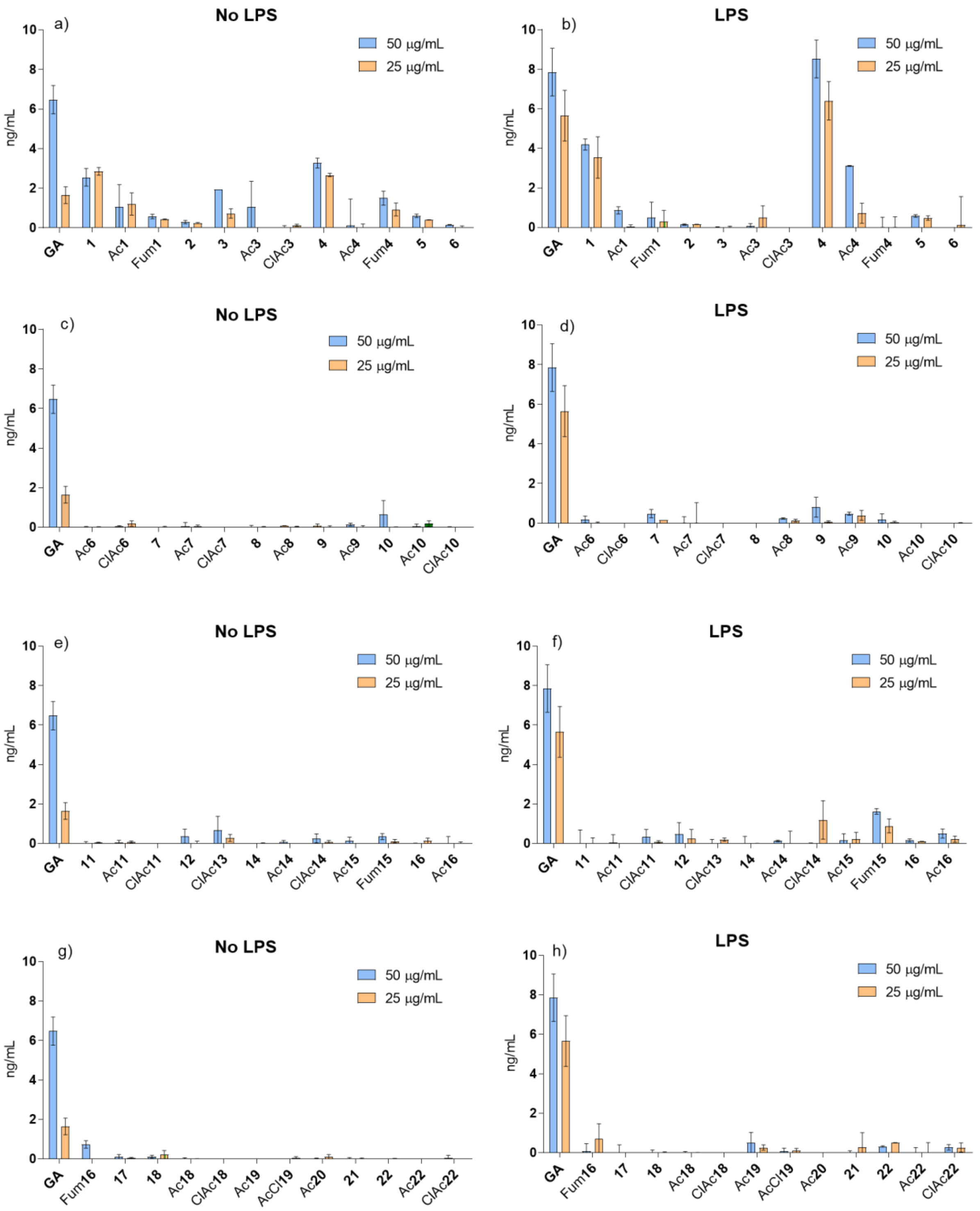
a)
No LPS
b)
LPS
c)
No LPS
d)
LPS
e)
No LPS
f)
LPS
g)
No LPS
h)
LPS
50 µg/mL
25 µg/mL
ng/mL
0
2
4
6
8
10
GA 1 Ac1 Fum1 2 3 Ac3 ClAc3 4 Ac4 Fum4 5 6
GA Ac6 ClAc6 7 Ac7 ClAc7 8 Ac8 9 Ac9 10 Ac10 ClAc10
GA 11 Ac11 ClAc11 12 ClAc13 14 Ac14 ClAc14 Ac15 Fum15 16 Ac16
GA Fum16 17 18 Ac18 ClAc18 Ac19 AcCl19 Ac20 21 22 Ac22 ClAc22

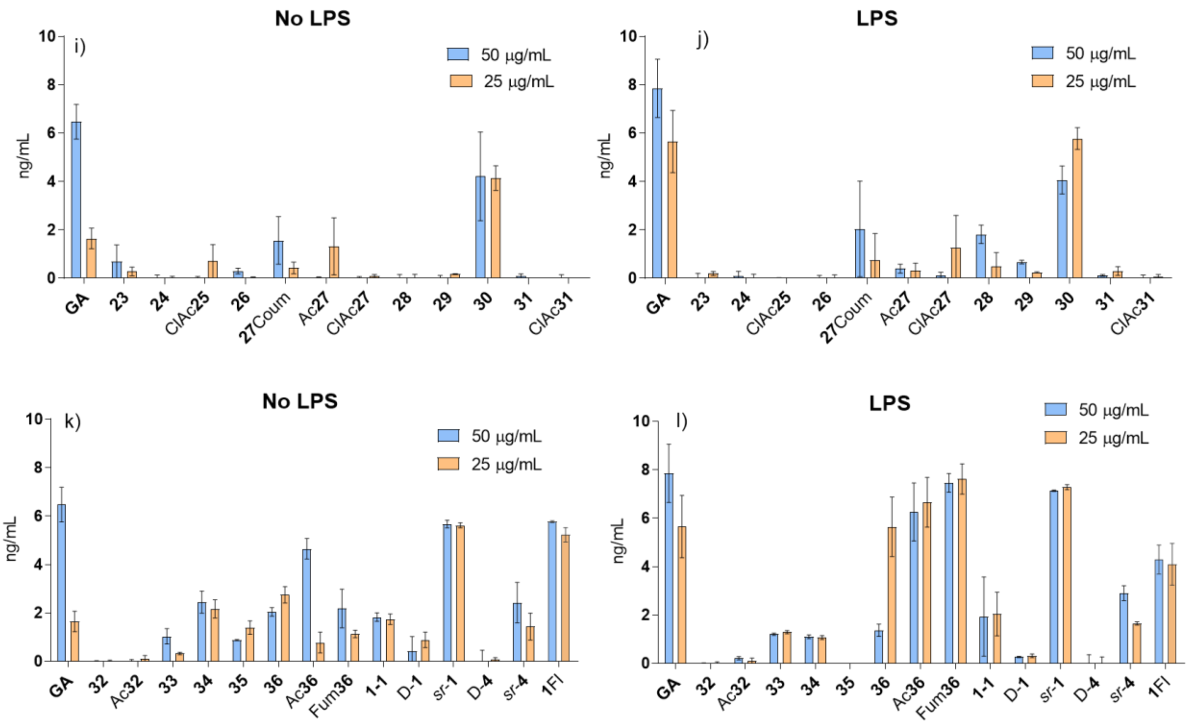

Figure S2. Cytokine level of IL-1Ra in supernatants of monocytes for active dendrimers and analogues evaluated by ELISA assay on primary human monocytes of healthy donors. 5×10^4 cells/200 µL in 96 well plates, were incubated with dendrimers or GA as a control for 48 h in presence or absence of LPS (100 ng/mL), in a range of concentrations (50-25 µg/mL) (mean ± SD, n = 2 different experiments). The results are present as the difference between each sample and the non-treated sample to take into account the difference between base line for each donor.

Table S3. List of p-values computed relative to the inactive analog D-**4** in the ELISA screening in Figure 2.

ID	p-value for 50 µg/mL	p-value for 25 µg/mL
**1**	<0,0001	0,0065
**4**	0,0255	0,0229
**34**	0,0003	0,0015
**36**	0,0022	<0,0001
Ac**36**	<0,0001	-
**1-1**	<0,0001	0,0001
sr-**1**	0,0005	-
sr-**4**	<0,0001	<0,0001
**1**Fl	<0,0001	<0,0001

Table S4. List of p-values computed relative to the not treated control in the ELISA screening in Figure 3.

ID	P-value for HD1	P-value for HD2	P-value for HD3	P-value for HD4	P-value for HD5
**GA**	0,0161	0,0002	0,4595	0,0116	0,0090
**1**	<0,0001	-	0,0036	0,0397	<0,0001
**4**	<0,0001	0,0287	0,0024	0,0006	<0,0001

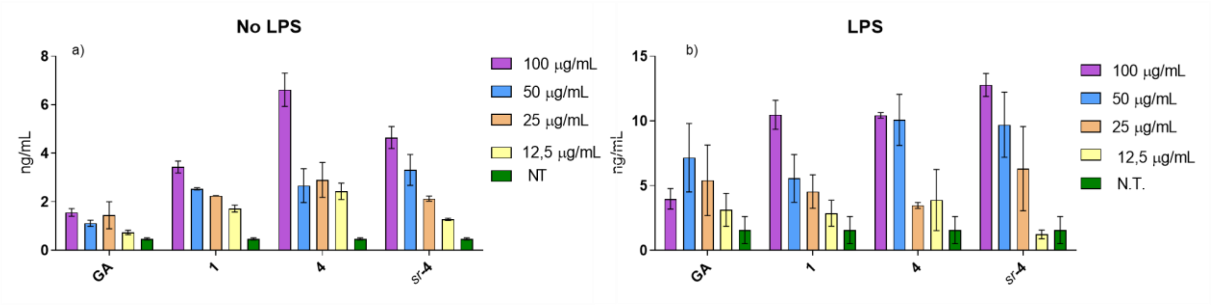

Figure S3. Cytokine level of IL-1Ra in supernatants of monocytes for active dendrimers and analogues evaluated by ELISA assay on primary human monocytes of healthy donors. $5{\times}10^4$ cells/200 µL in 96 well plates, were incubated with dendrimers or GA as a control for 48 h in presence or absence of LPS (100 ng/mL), in a range of concentrations (100-12.5 µg/mL) (mean ± SD, n = 3 different experiments).

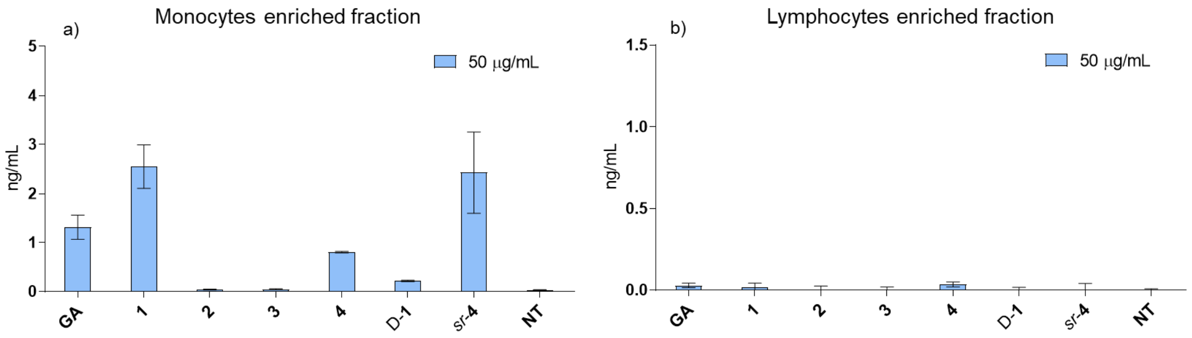

Figure S4. Cytokine level of IL-1Ra in supernatants of monocytes enriched fraction (a) and the rest of leukocytes (b) for active dendrimers and analogues evaluated by ELISA assay. Monocytes fraction was separated from whole PBMC by Percoll density gradient centrifugation. $5{\times}10^4$ cells/200 µL in 96 well plates were incubated with dendrimers or GA (50-25 µg/mL) as a control for 48 h (mean ± SD, n = 2 different experiments).

mRNA Quantification

Enriched monocytes after Percoll centrifugation (4×10^6 cells/3 mL well; 6-well plates) were preincubated for 1 h with or without compounds in RPMI-1640 medium supplemented with 10% heat-inactivated FBS, 50 µg/mL streptomycin, 50 U/mL penicillin, 2 mM L-glutamine, and then incubated for the indicated duration. After the incubation cells were collected, total RNA was extracted, reverse transcribed and analyzed by SYBR Green Master Mix. As a housekeeping gene was used Actin 5'-CAC TGG GGG CTA CTG GAC-3', 3'-AAC ATG GTG TTG GCA GAA ACT-5', IL-1Ra 5'-TTC CTG TTC CAT TCA GAG ACG-3', 3'-CTT CTG GTT AAC ATC CCA GAT TC-5'.

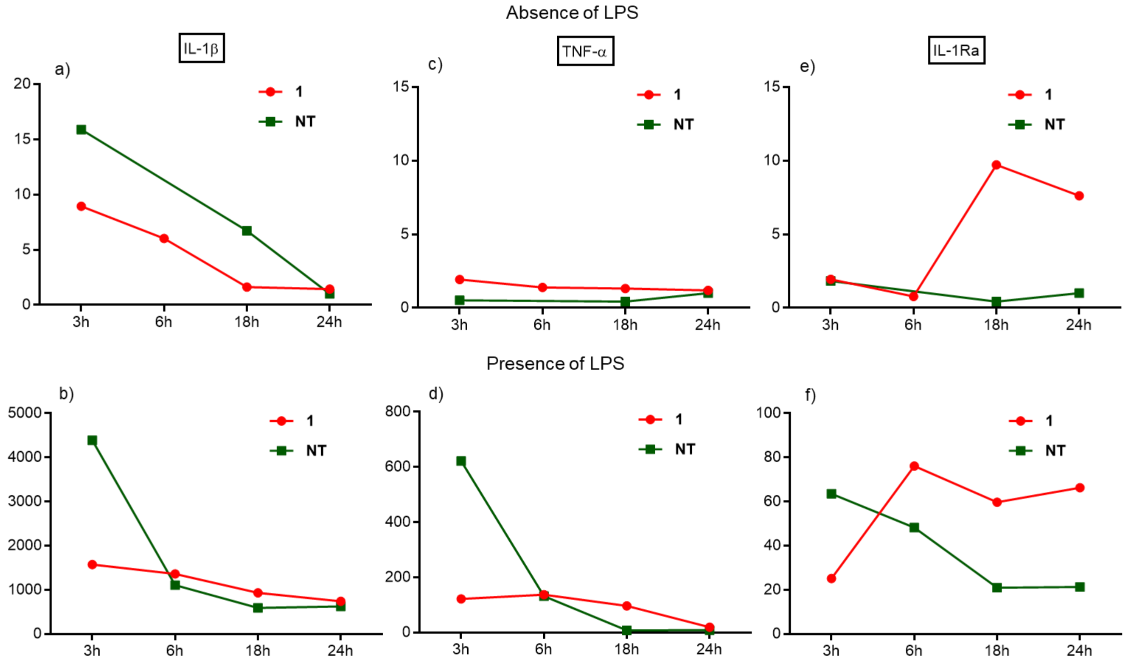

Figure S5. Dendrimer **1** affected mRNA levels in both LPS-activated and resting monocytes. Monocytes 4×10^6 cells/3 mL in 6 well plates were incubated for indicated time with 50 µg/mL of **1** or without any treatment (NT) (a, c, e); or preincubated for 1 h with 100 ng/mL of LPS, and then with 50 µg/mL of **1** (b, d, f) or without any treatment (NT). Total RNA was isolated, reverse transcribed and analyzed by RT-qPCR for presence IL-1β, IL-1Ra and TNF-α.

Confocal Microscopy

Membrane and nucleus staining

8-well chambered coverglass plates were treated with poly-L-Lysine for 30 min, dried at RT for 2 h. Monocytes were plated at 2×10^5 cells/300 µL per well and incubated for the indicated duration with dendrimers in presence or absence of LPS at 37 °C in a humidified atmosphere in 5% carbon dioxide following the removal of the full growth medium. Then, cells were washed with warm PBS twice and the cell membrane was labelled with CellMask Deep Red plasma membrane stain in PBS (0.25 µL in 0.25 mL/well) and nucleus was stained with Hoechst 33258 in PBS (0.25 µL in 0.25 mL/well) for 30 min at 37 °C. After the incubation cells were washed with PBS twice and prewarmed Glycergel Mounting Medium was added. Images were taken on a Zeiss LSM 880 confocal microscope with Oil compatible lens x63/1.3.

Flow Cytometry experiments

PBMC of healthy donors were obtained by Ficoll density gradient centrifugation. Cells were incubated for 1 h in presence or absence of LPS (100 ng/mL) in 6-well plates, 4×10^6 cells/well, then with GA, selected dendrimers (50 µg/mL) or without any treatment for 18 h. After incubation cells were detached, washed twice with staining buffer (0.5% BSA in 1× PBS) and stained according to manufacture protocols for 30 min on ice with following antibodies: CD14 - PE, CD16 - BV605, HLA-DR - BV421, CD68 – PECy7, CCR2 – BV711, CD206 - APC. After two washes with staining buffer, samples were a acquired on LSR II SORP H274 and analyzed using FlowJo software.

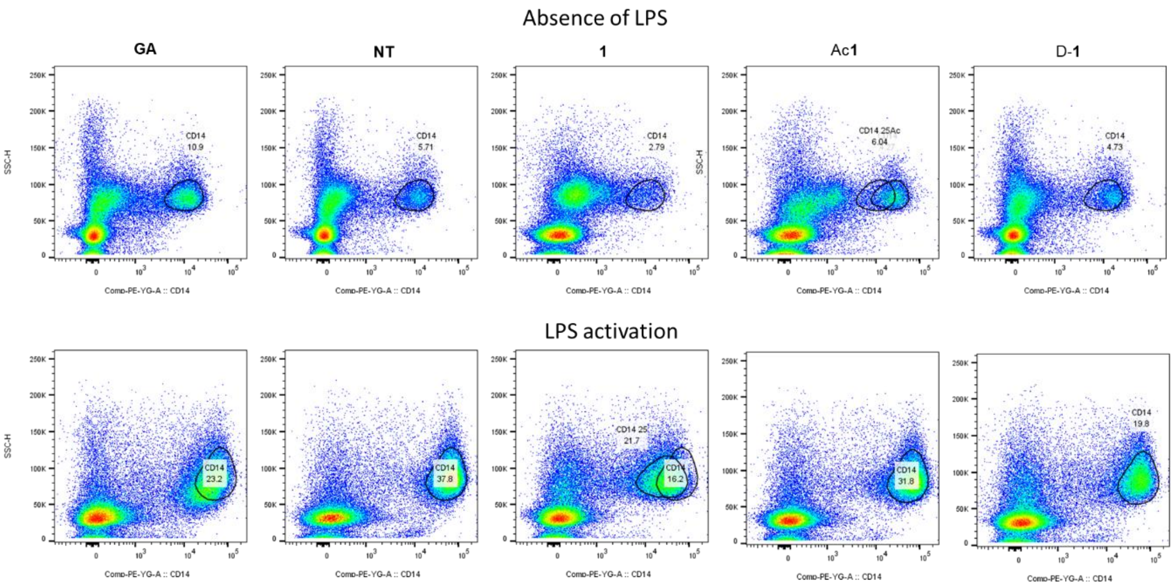

Figure S6. Flow cytometry dot plot showing the difference in CD14 expression on PBMC of a healthy donor in response on the treatment (50 µg/mL) with the dendrimers and GA or without treatment (NT) in absence of LPS for 18 h.

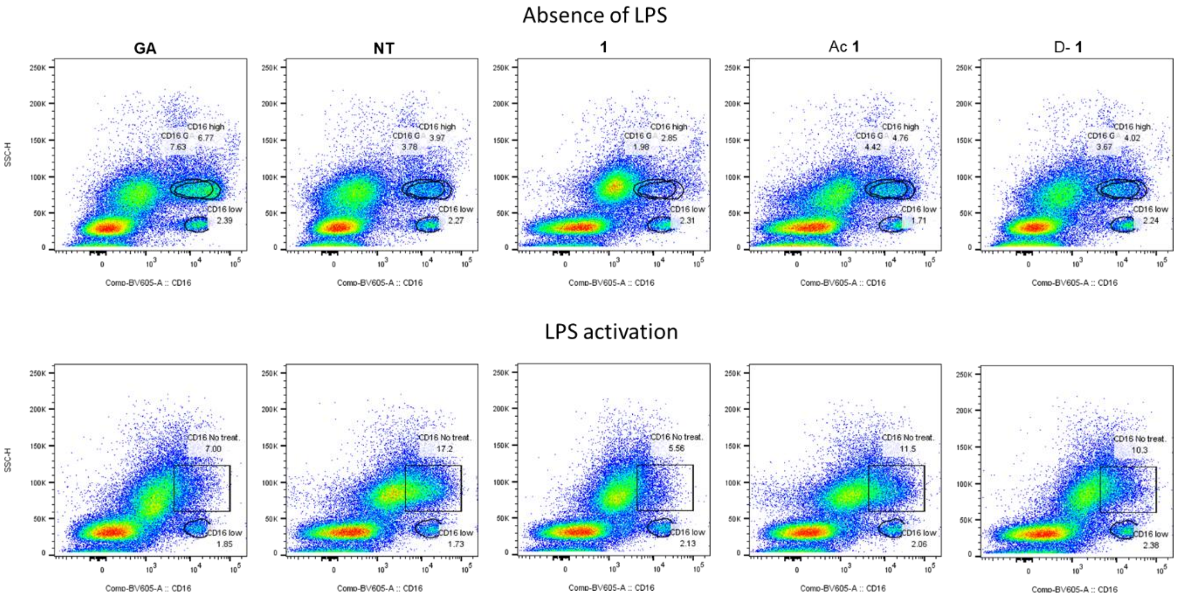

Figure S7. Flow cytometry dot plot showing the difference in CD16 expression on PBMC of a healthy donor in response to the treatment (50 µg/mL) with the dendrimers and GA or without treatment (NT) in absence of LPS for 18 h.

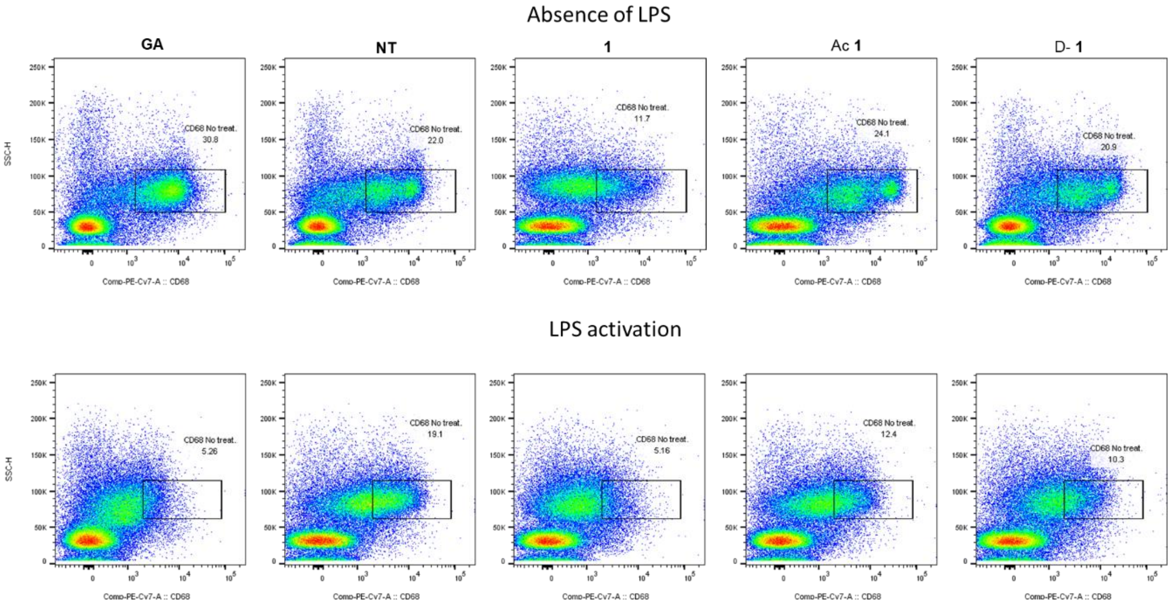

Figure S8. Flow cytometry dot plot showing the difference in CD68 expression on PBMC of a healthy donor in response to the treatment (50 µg/mL) with the dendrimers and GA or without treatment (NT) in absence of LPS for 18 h.

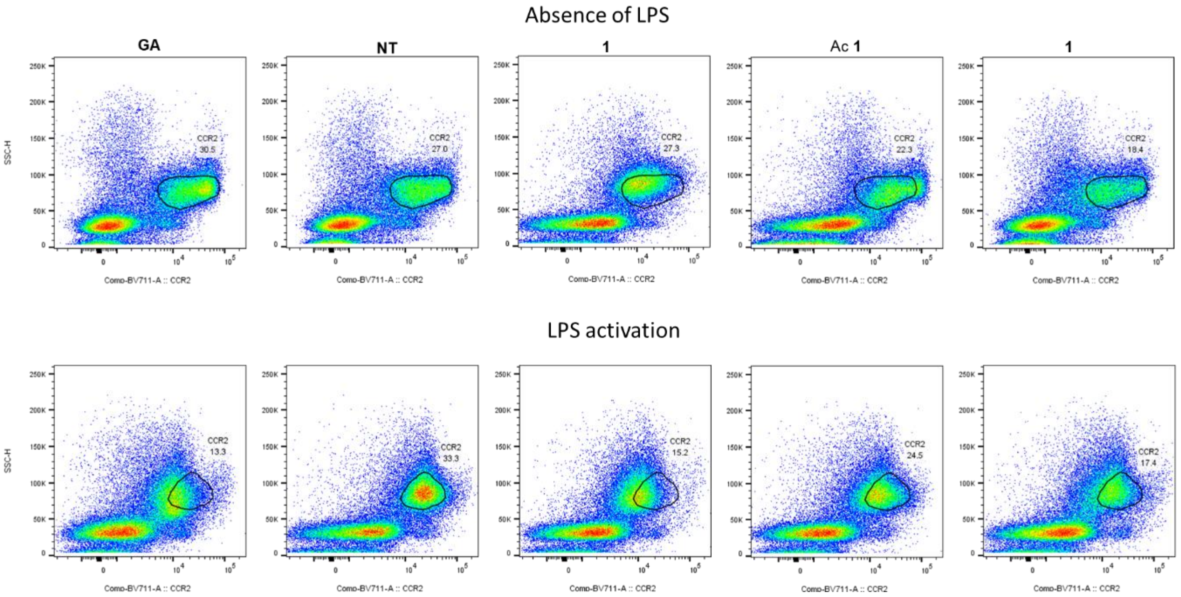

Figure S9. Flow cytometry dot plot showing the difference in CCR2 expression on PBMC of a healthy donor in response to the treatment (50 µg/mL) with the dendrimers and GA or without treatment (NT) in absence of LPS for 18 h.

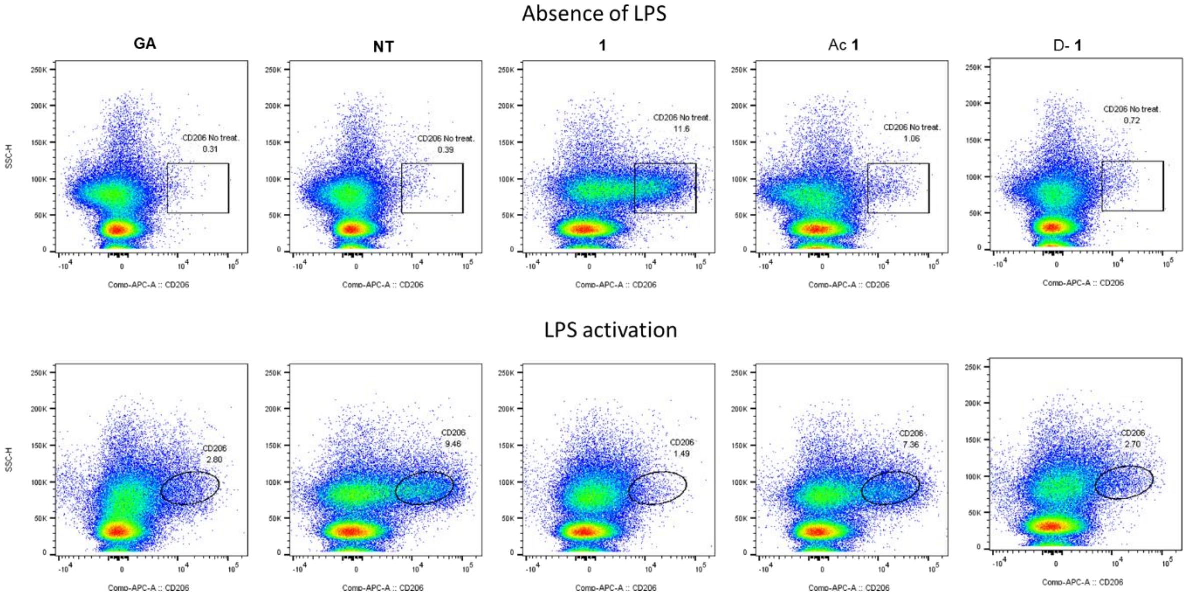

Figure S10. Flow cytometry dot plot showing the difference in HLA-DR expression on PBMC of a healthy donor in response to the treatment (50 µg/mL) with the dendrimers and GA or without treatment (NT) in absence of LPS for 18 h.

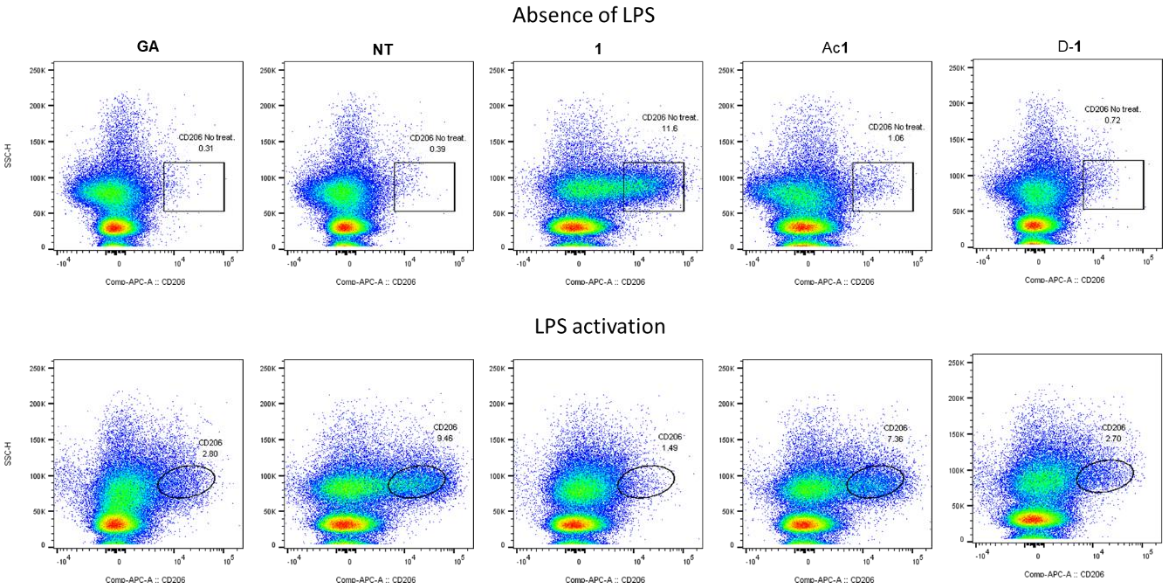

Figure S11. Flow cytometry dot plot showing the difference in CD206 expression on PBMC of a healthy donor in response to the treatment (50 µg/mL) with the dendrimers and GA or without treatment (NT) in absence of LPS for 18 h.

CD Spectroscopy

Circular dichroism (CD) experiments were measured on a Jasco J-715 Spectropolarimeter. All the experiments were performed using Hellma Suprasil 110-QS 0.1 cm cuvettes. For each peptide, the measurements were performed in phosphate buffer (PB, pH=7.2, 8 mM) and in the presence of 5 mM dodecylphosphocholine. The concentration of the peptides was 100.0 µg/mL and each sample was measured using one accumulation. The scan rate was 10 nm/min, pitch 0.5 nm, response 16 sec and bandwidth 1.0 nm. The nitrogen flow was kept >8.5 L/min. After each measurement, the cuvettes were washed successively with 1 M HCl, milli-Q H_2O and PB buffer. The baseline was recorded under the same conditions and subtracted manually.

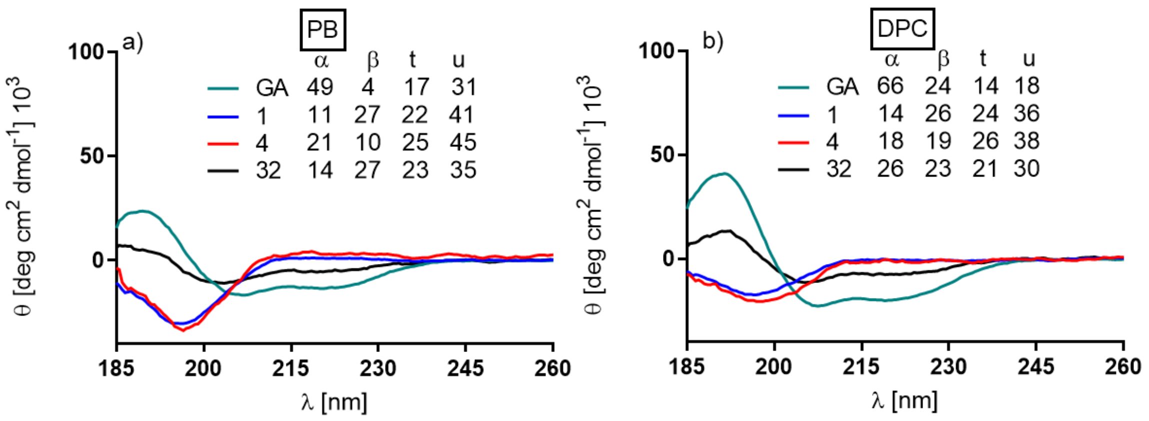

Figure S12. Circular dichroism spectra of GA, **1** and **4** (100 µg/mL) in 8 mM phosphate buffer at pH 7.4 (a) or in presence of 5 mM dodecylphosphocholine. Percentage of α-helix and β-sheet was processed by Dichroweb using the CONTIN analysis program and reference set 3.

Synthesis, HPLC and MS Data for all Dendrimers

1 ((KA)$_8$(*K*AK)$_4$(*K*EKA)$_2$*K*AKEAYCA-NH$_2$) was obtained from the CEM Liberty Blue synthetiser as foamy colourless solid after preparative RP-HPLC (61.9 mg, 1.1 µmol, 7.7%). Analytical RP-HPLC: t_R=1.19 min (100% A to 100% D in 5 min, λ= 214 nm). HRMS (ESI+): $C_{210}H_{387}N_{67}O_{51}S$ calc./obs. 4695.9470/4695.9625 Da [M].

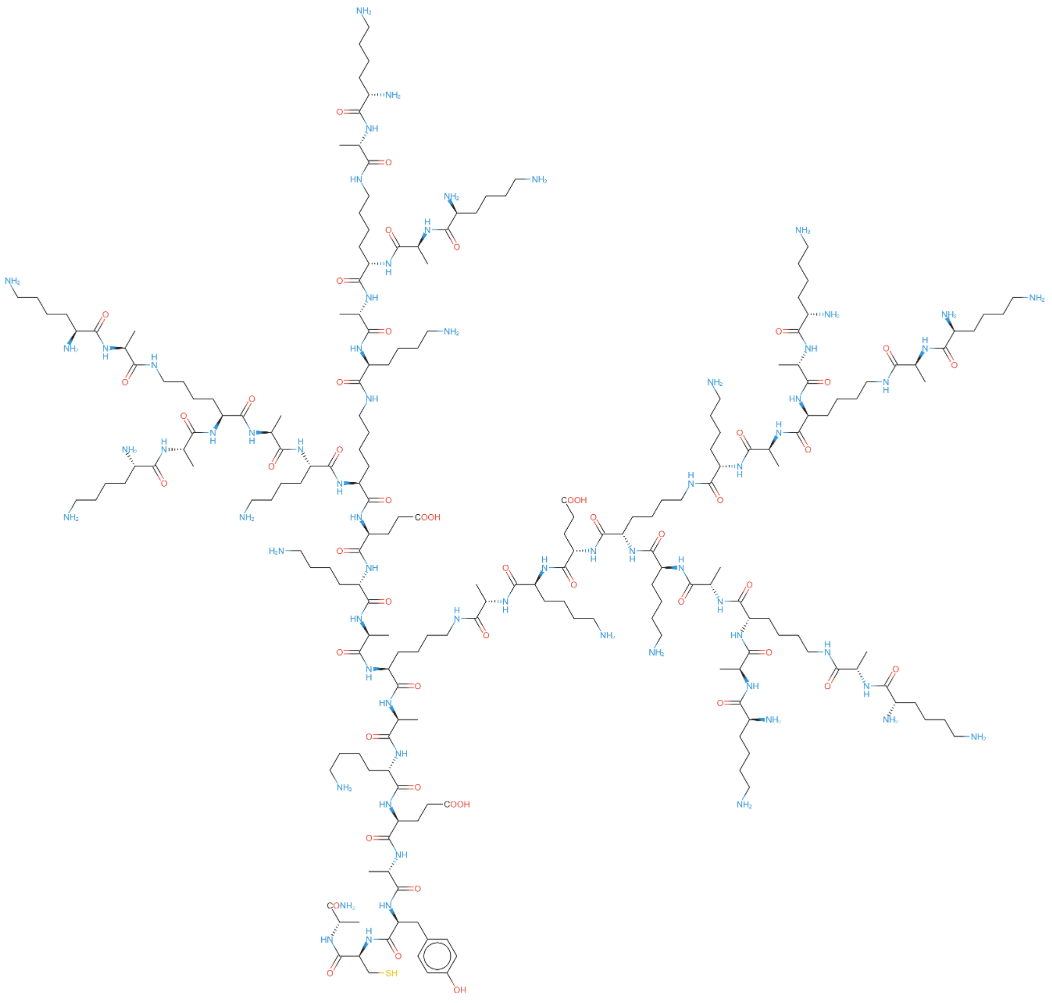

Analytical RP-HPLC:

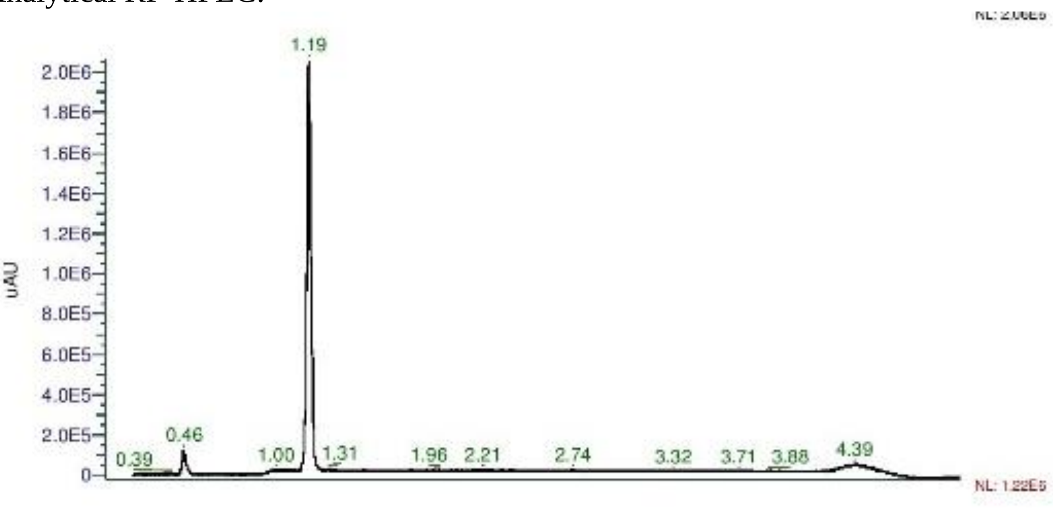

HRMS (NSI+):

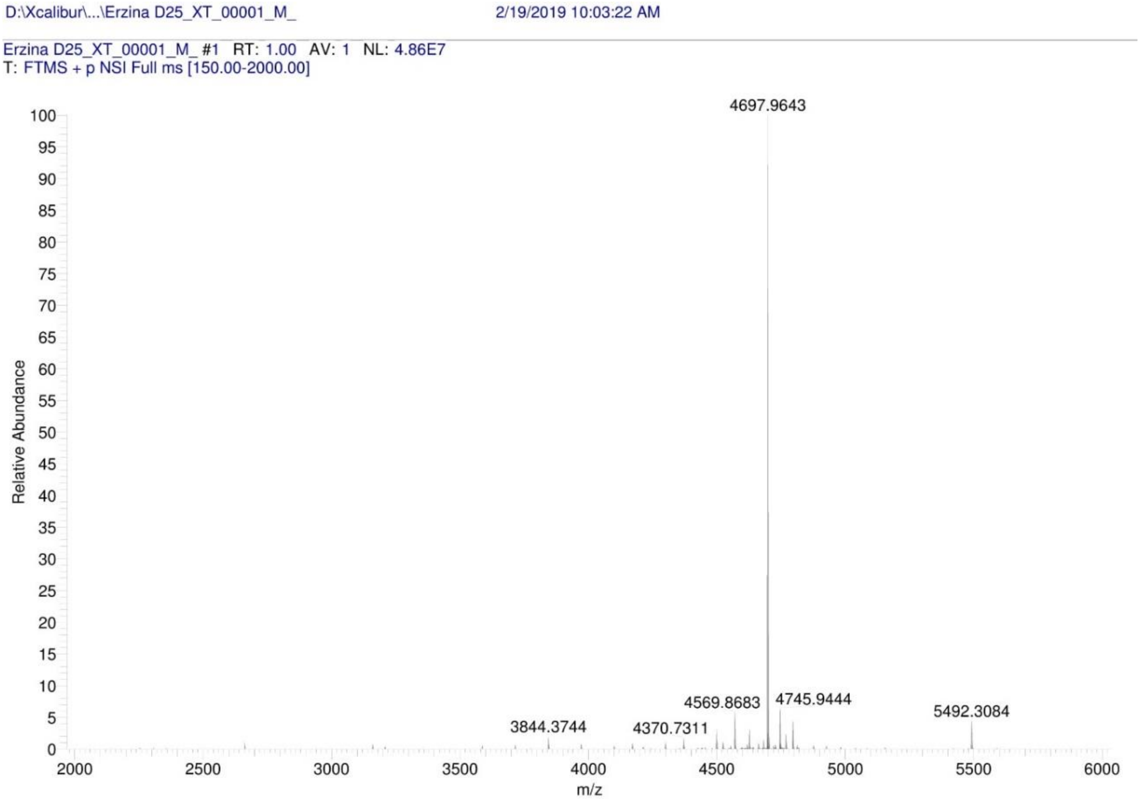

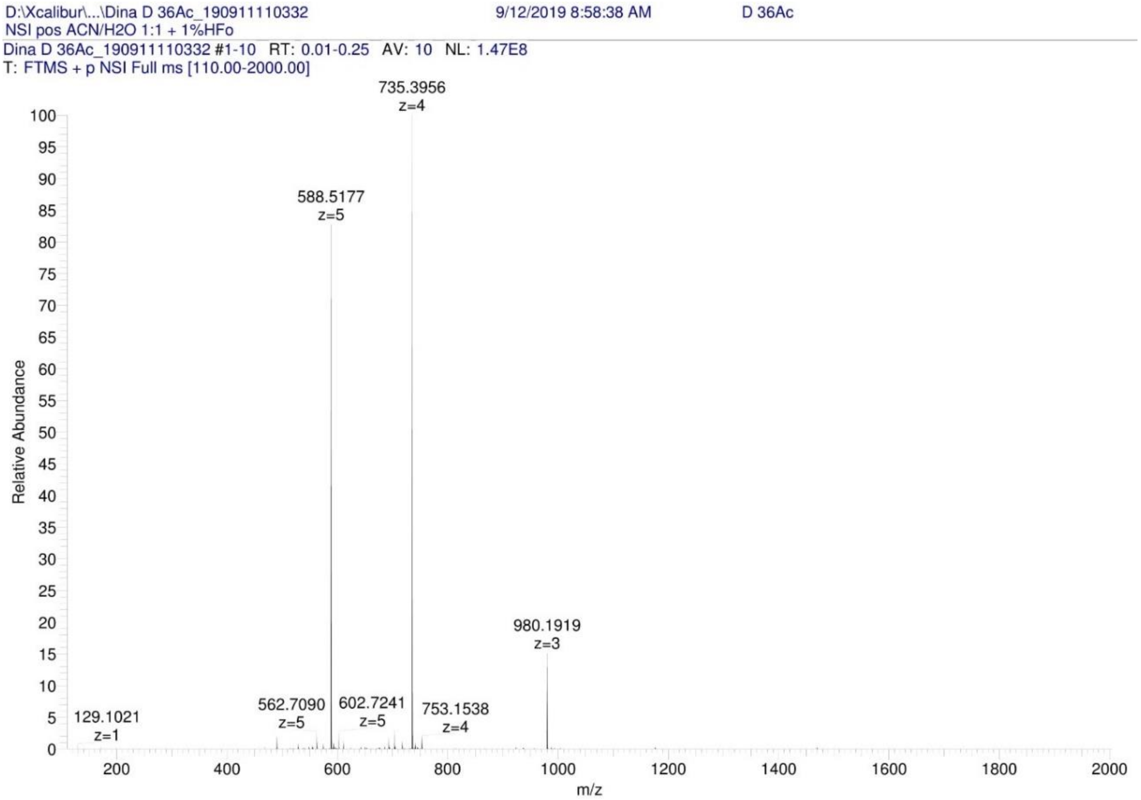
D:\Xcalibur\...\Dina D 36Ac_190911110332
9/12/2019 8:58:38 AM
D 36Ac
NSI pos ACN/H2O 1:1 + 1%HFo
Dina D 36Ac_190911110332 #1-10 RT: 0.01-0.25 AV: 10 NL: 1.47E8
T: FTMS + p NSI Full ms [110.00-2000.00]
735.3956
z=4
588.5177
z=5
980.1919
z=3
129.1021
z=1
562.7090
z=5
602.7241
z=5
753.1538
z=4
Relative Abundance
m/z

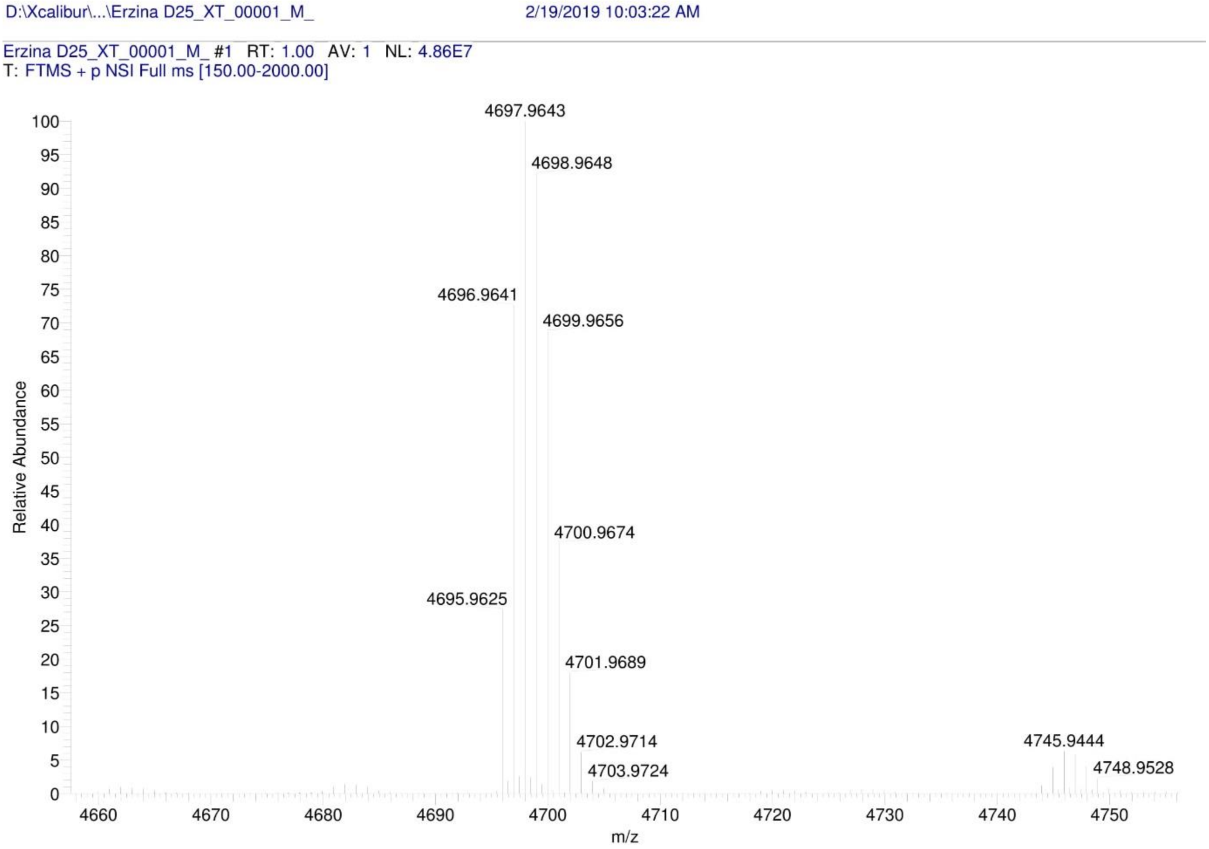
D:\Xcalibur\...\Erzina D25_XT_00001_M_
2/19/2019 10:03:22 AM
Erzina D25_XT_00001_M_ #1 RT: 1.00 AV: 1 NL: 4.86E7
T: FTMS + p NSI Full ms [150.00-2000.00]
4697.9643
4698.9648
4696.9641
4699.9656
4700.9674
4695.9625
4701.9689
4702.9714
4703.9724
4745.9444
4748.9528
Relative Abundance
m/z

Ac**1 ((AcKA)$_8$(*K*AK)$_4$(*K*EKA)$_2$*K*AKEAYCA-NH$_2$)** was obtained from the CEM Liberty Blue synthetiser as foamy colourless solid after preparative RP-HPLC (53.9 mg, 3.4 µmol, 22.8%). Analytical RP-HPLC: t_R=1.25 min (100% A to 100% D in 5 min, λ= 214 nm). HRMS (ESI+): $C_{226}H_{403}N_{67}O_{59}S$ calc./obs. 5032.0315/5032.0448 Da [M].

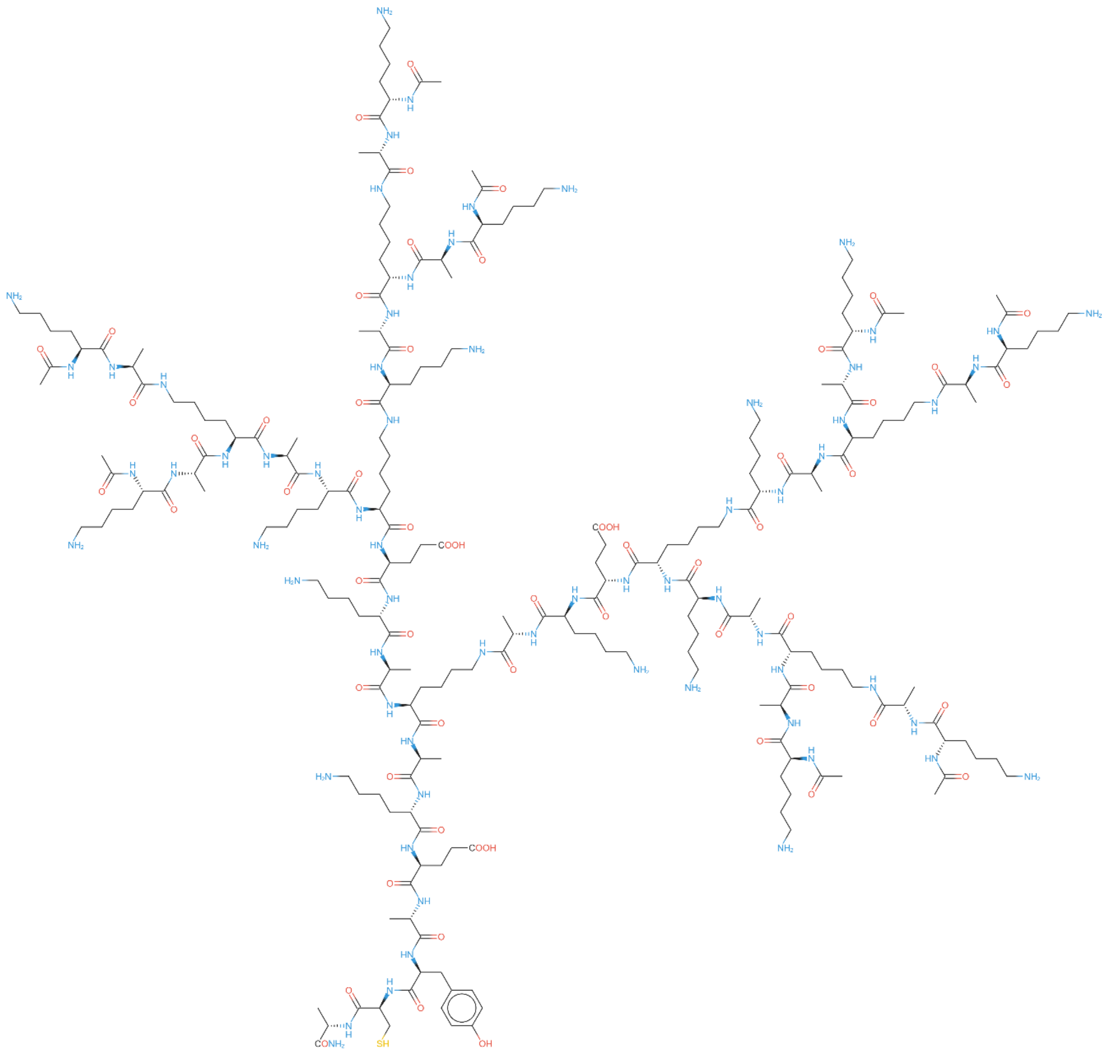

Analytical RP-HPLC:

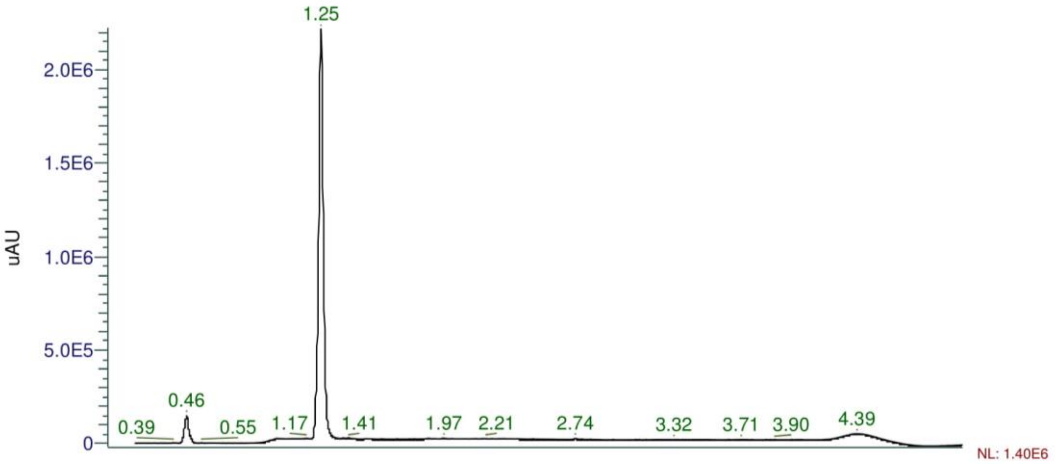

HRMS (NSI+):

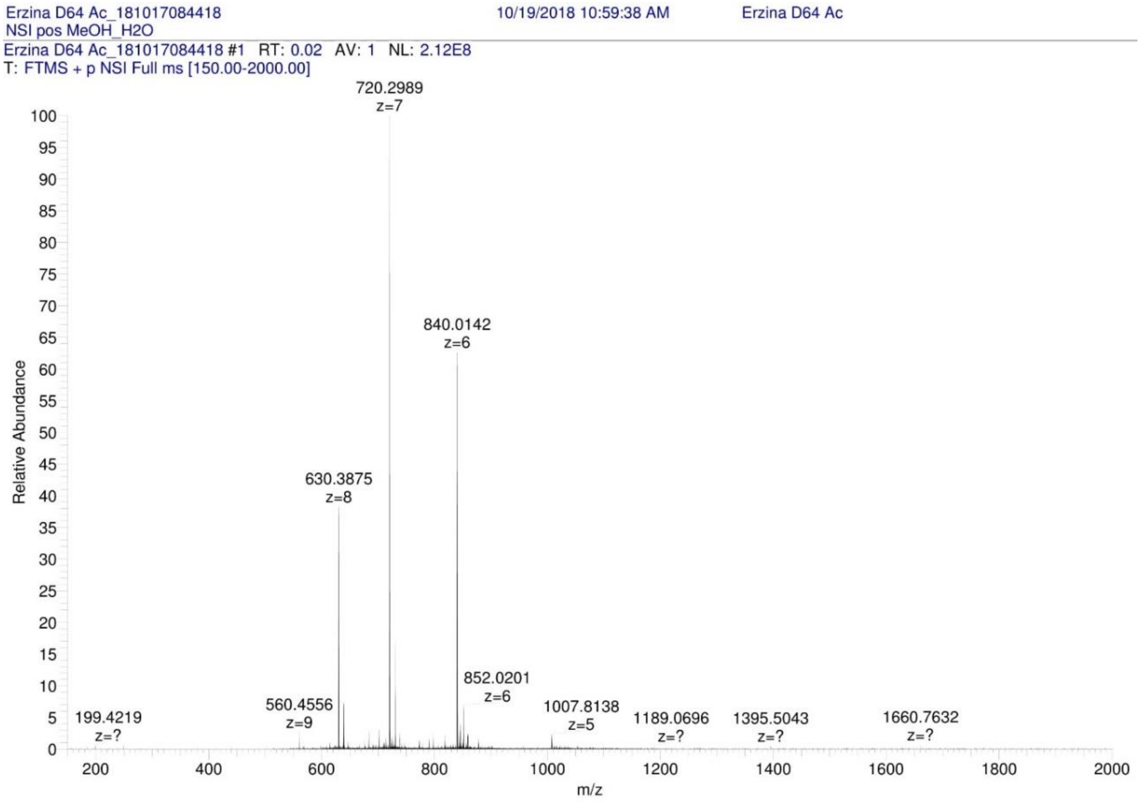
Erzina D64 Ac_181017084418
10/19/2018 10:59:38 AM
Erzina D64 Ac
NSI pos MeOH_H2O
Erzina D64 Ac_181017084418 #1 RT: 0.02 AV: 1 NL: 2.12E8
T: FTMS + p NSI Full ms [150.00-2000.00]
720.2989 z=7
840.0142 z=6
630.3875 z=8
852.0201 z=6
560.4556 z=9
1007.8138 z=5
199.4219 z=?
1189.0696 z=?
1395.5043 z=?
1660.7632 z=?
Relative Abundance
m/z

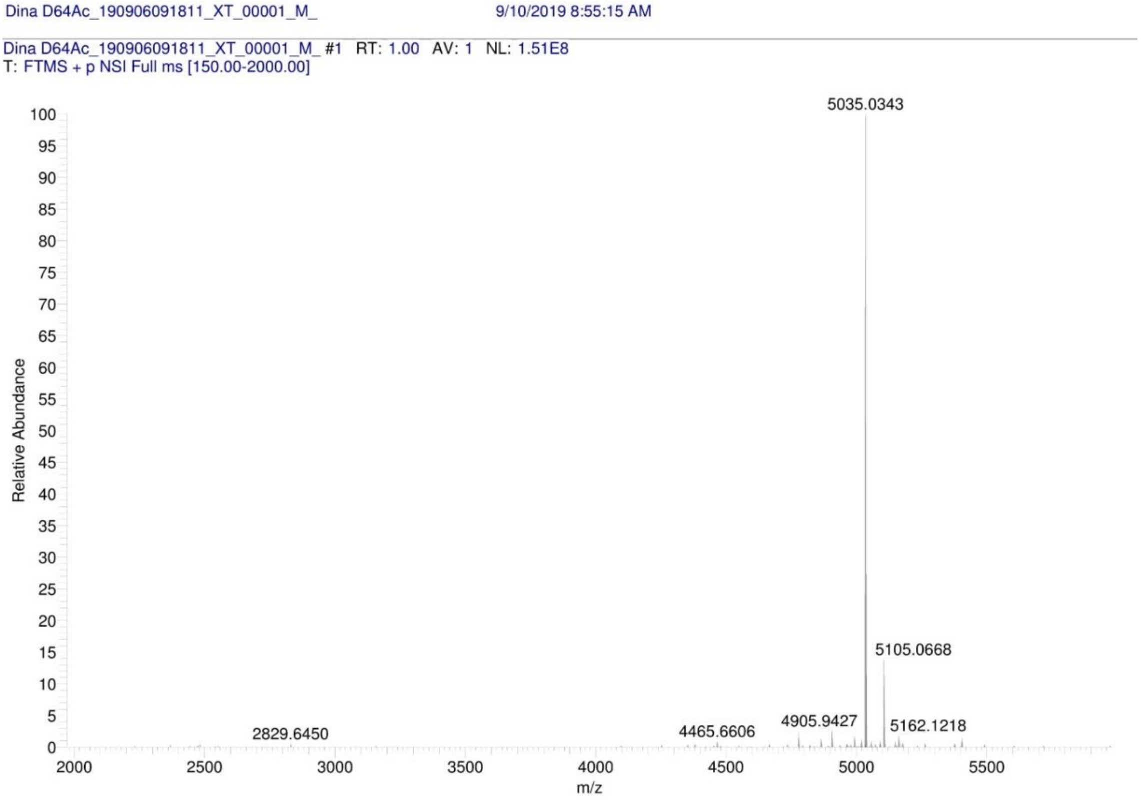
Dina D64Ac_190906091811_XT_00001_M_
9/10/2019 8:55:15 AM
Dina D64Ac_190906091811_XT_00001_M_ #1 RT: 1.00 AV: 1 NL: 1.51E8
T: FTMS + p NSI Full ms [150.00-2000.00]
5035.0343
5105.0668
4905.9427
5162.1218
4465.6606
2829.6450
Relative Abundance
m/z

Erzina D64 Ac_181017084418_XT_00001_M_ 10/19/2018 11:00:06 AM

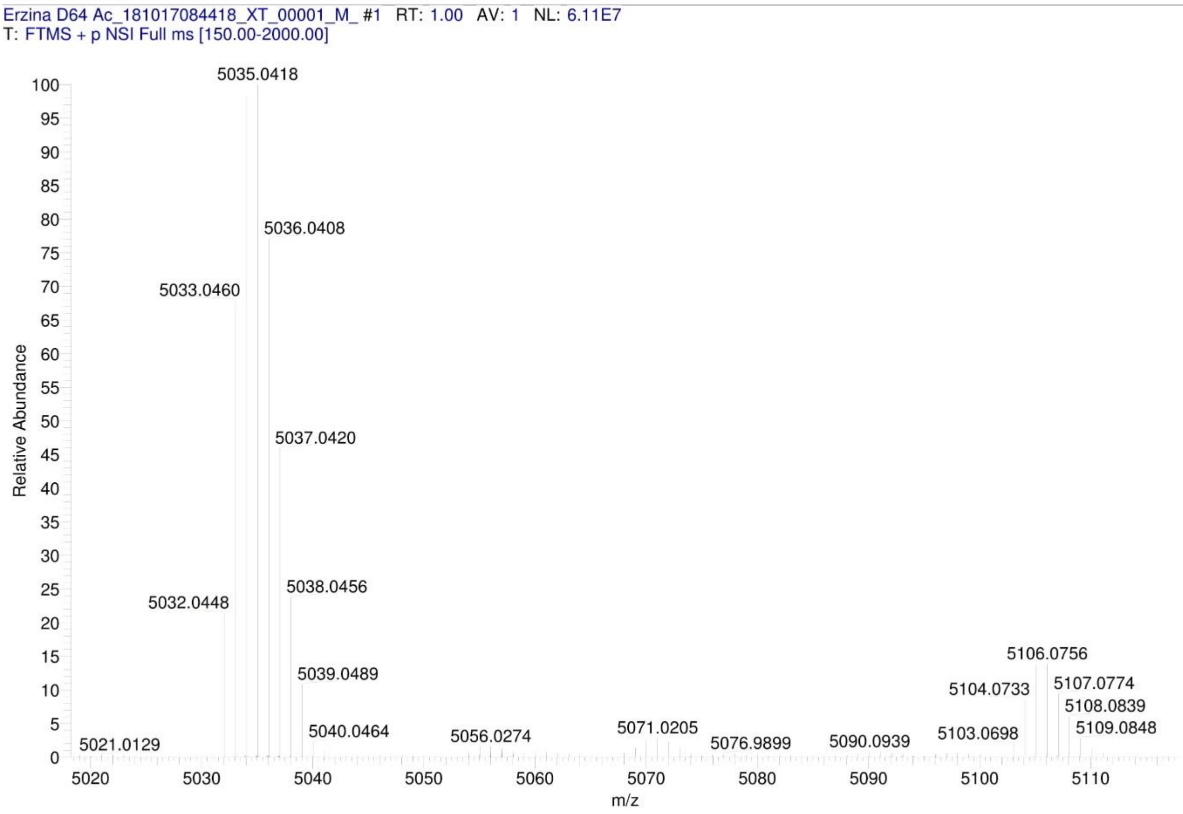

Fum**1 ((FumKA)$_8$(*K*AK)$_4$(*K*EKA)$_2$*K*AKEAYCA-NH$_2$)** was obtained from the CEM Liberty Blue synthetiser as foamy colourless solid after preparative RP-HPLC (47.6 mg, 3.1 µmol, 22.9%). Analytical RP-HPLC: t_R=1.47 min (100% A to 100% D in 5 min, λ= 214 nm). $C_{258}H_{435}N_{67}O_{75}S$ calc./obs. 5704.2005/5704.2057 Da [M].

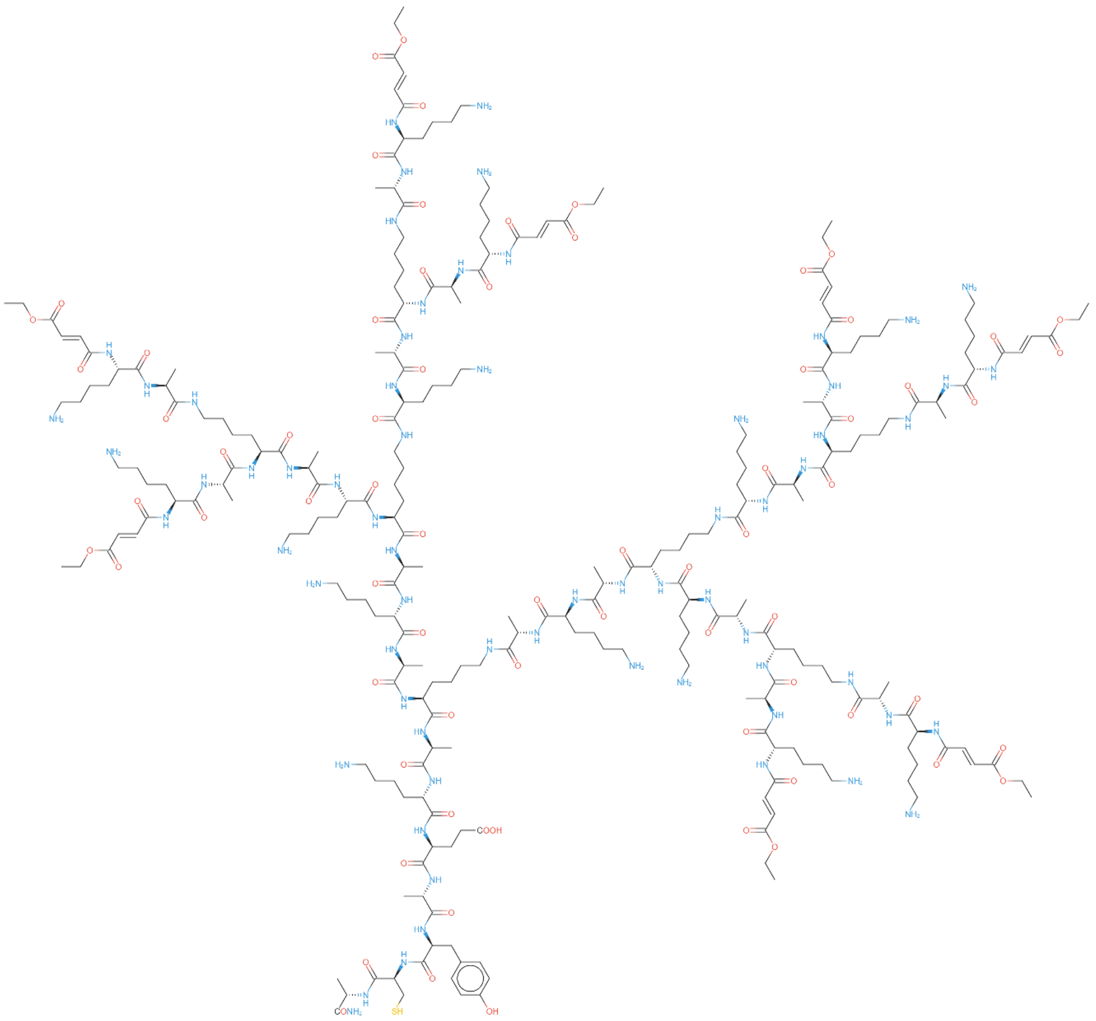

Analytical RP-HPLC:

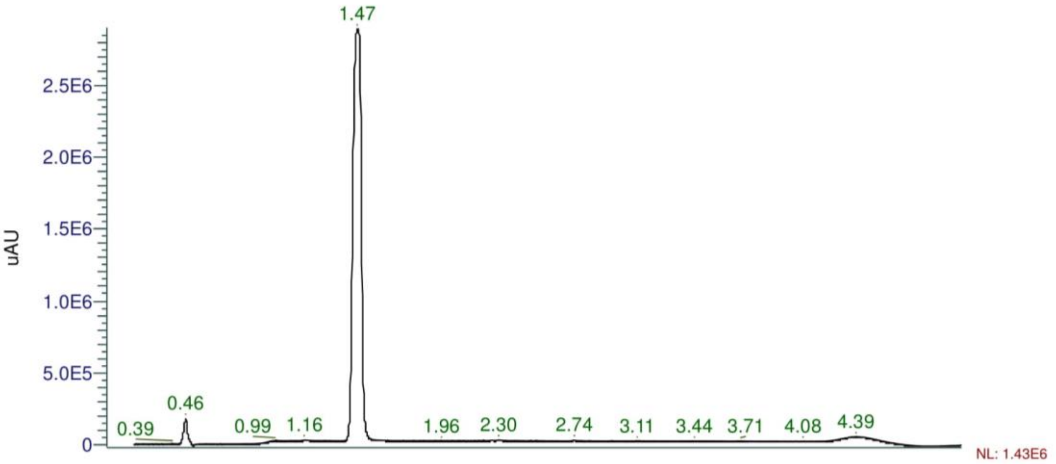

HRMS (NSI+):

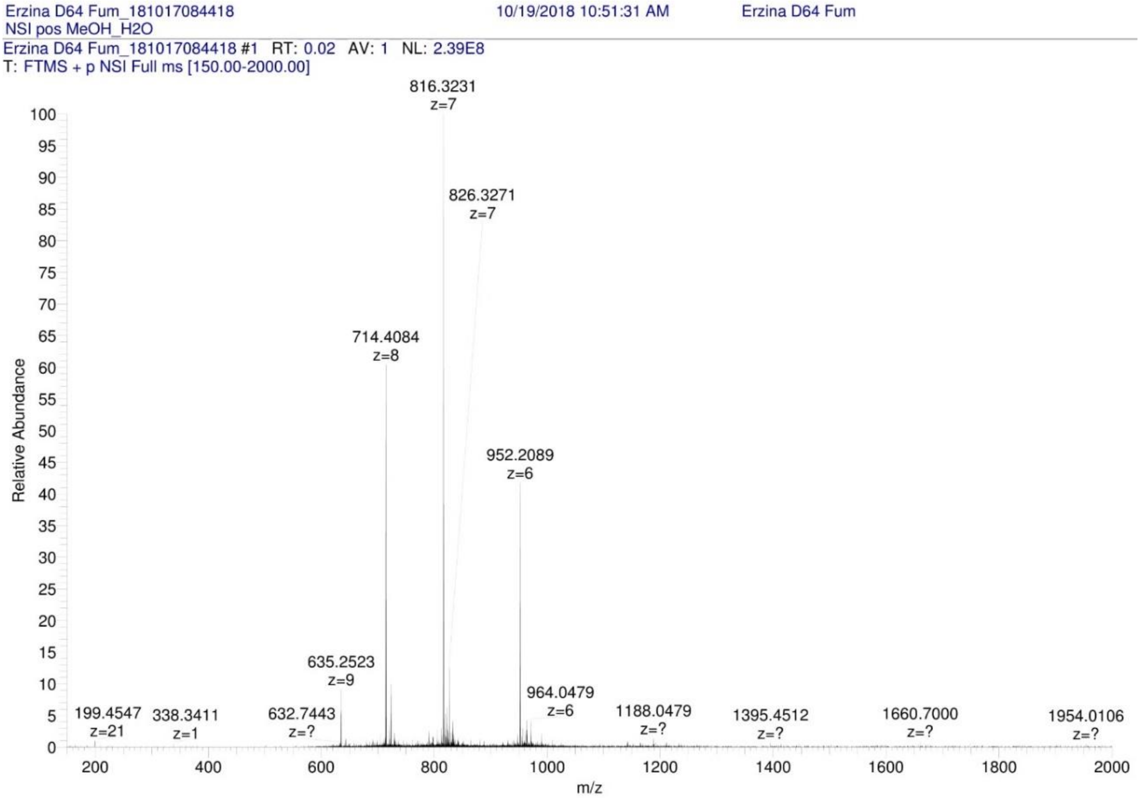
Erzina D64 Fum_181017084418
10/19/2018 10:51:31 AM
Erzina D64 Fum
NSI pos MeOH_H2O
Erzina D64 Fum_181017084418 #1 RT: 0.02 AV: 1 NL: 2.39E8
T: FTMS + p NSI Full ms [150.00-2000.00]
816.3231 z=7
826.3271 z=7
714.4084 z=8
952.2089 z=6
635.2523 z=9
964.0479 z=6
199.4547 z=21
338.3411 z=1
632.7443 z=?
1188.0479 z=?
1395.4512 z=?
1660.7000 z=?
1954.0106 z=?
Relative Abundance
m/z

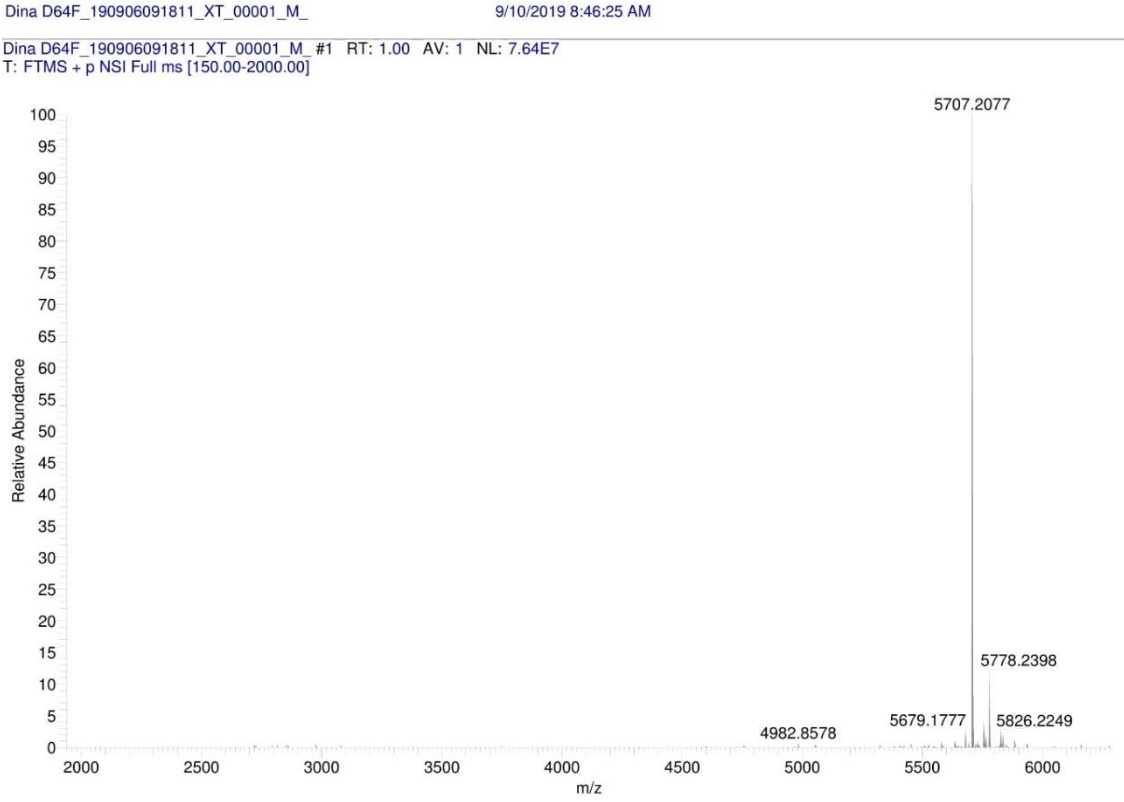
Dina D64F_190906091811_XT_00001_M_
9/10/2019 8:46:25 AM
Dina D64F_190906091811_XT_00001_M_ #1 RT: 1.00 AV: 1 NL: 7.64E7
T: FTMS + p NSI Full ms [150.00-2000.00]
5707.2077
5778.2398
5679.1777
5826.2249
4982.8578
Relative Abundance
m/z

Erzina D64 Fum_181017084418_XT_00001_M_ 10/19/2018 10:51:55 AM

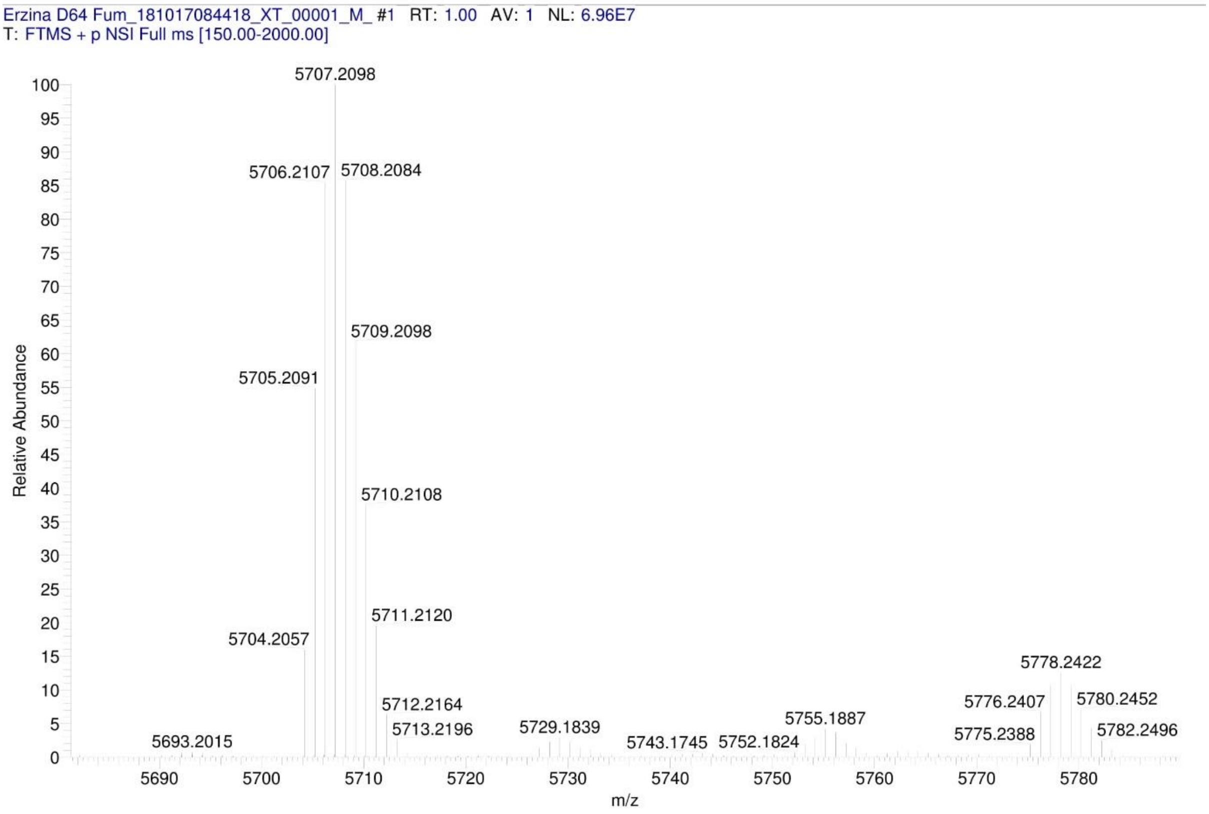

2 ((KAEKAYA)$_4$(*K*EKYAKA)$_2$*K*EKYKA-NH$_2$) was obtained f after manual synthesis as foamy colourless solid after preparative RP-HPLC (211.0 mg, 25.6 µmol, 25.6%). Analytical RP-HPLC: t_R=1.29 min (100% A to 100% D in 5 min, λ= 214 nm). HRMS (ESI+): $C_{251}H_{404}N_{66}O_{69}$ calc./obs. 5447.0133/5447.0332 Da [M].

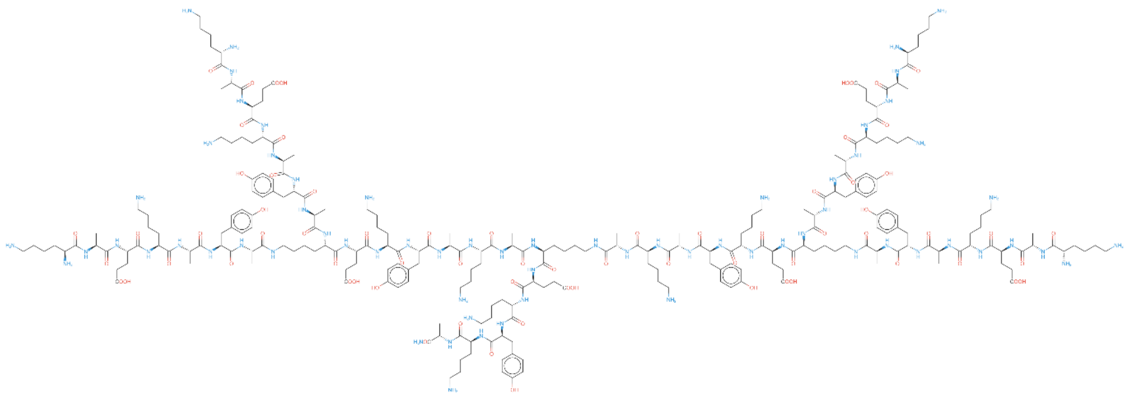

Analytical RP-HPLC:

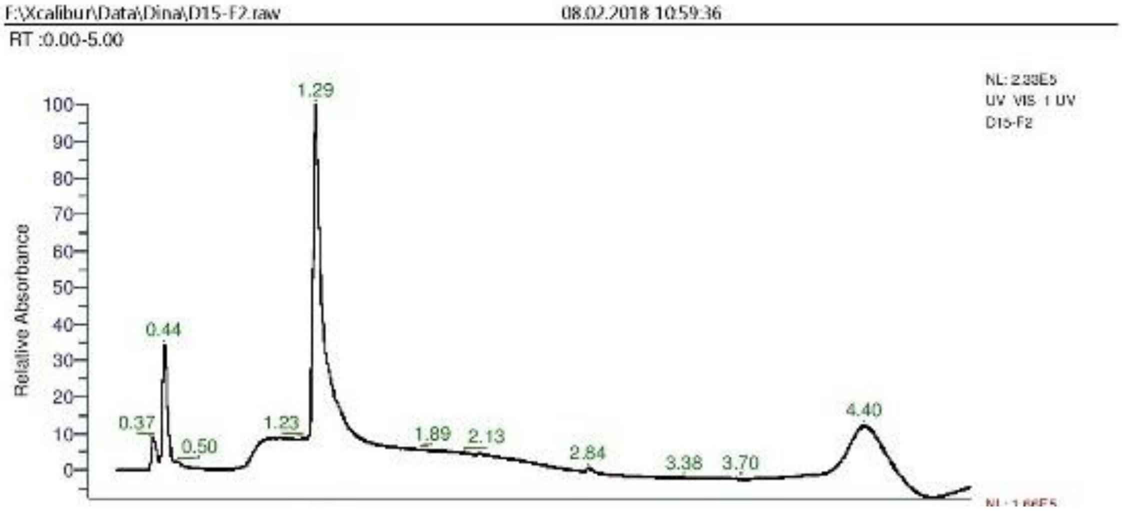

HRMS (NSI+):

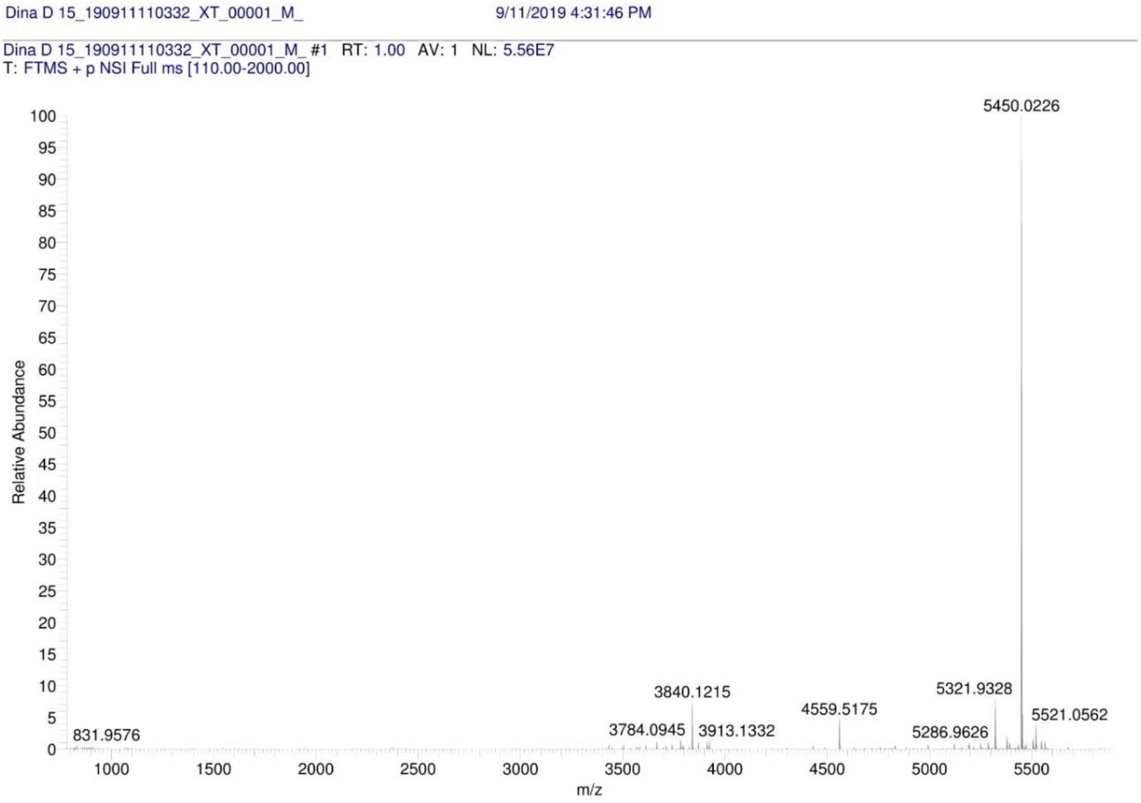
Dina D 15_190911110332_XT_00001_M_
9/11/2019 4:31:46 PM
Dina D 15_190911110332_XT_00001_M_ #1 RT: 1.00 AV: 1 NL: 5.56E7
T: FTMS + p NSI Full ms [110.00-2000.00]
5450.0226
3840.1215
4559.5175
5321.9328
5521.0562
3784.0945
3913.1332
5286.9626
831.9576
Relative Abundance
m/z

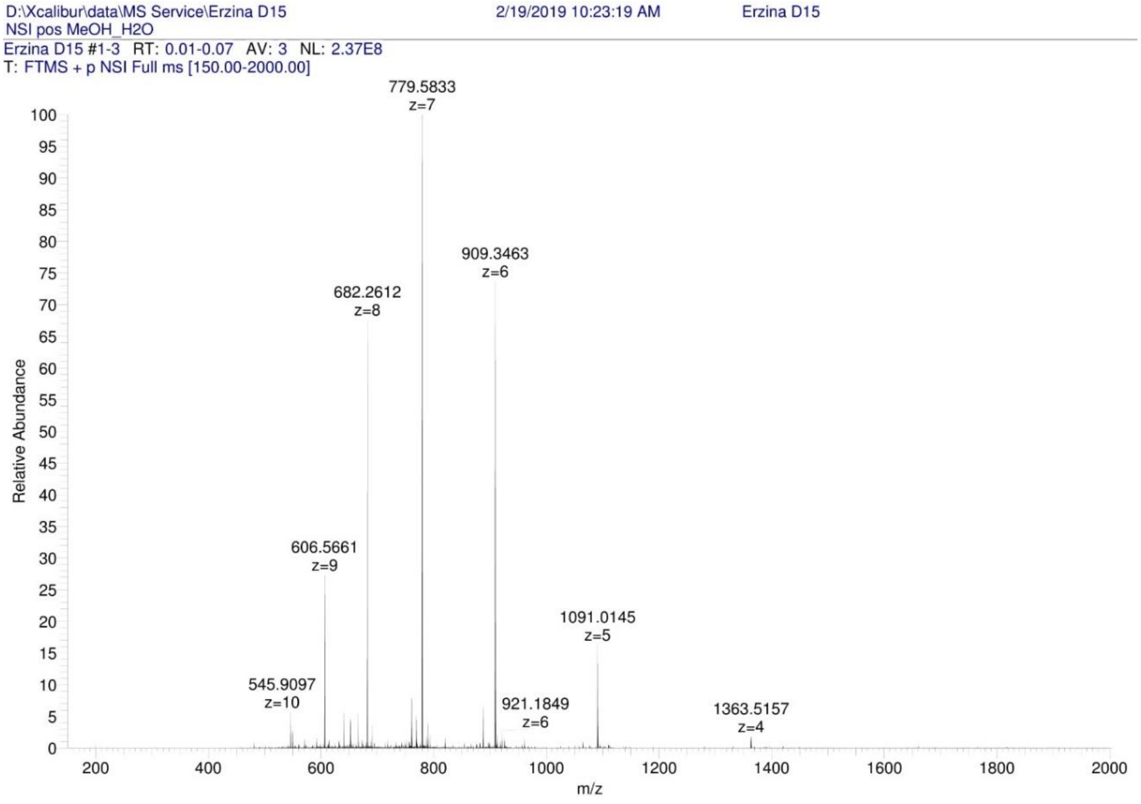
D:\Xcalibur\data\MS Service\Erzina D15
2/19/2019 10:23:19 AM
Erzina D15
NSI pos MeOH_H2O
Erzina D15 #1-3 RT: 0.01-0.07 AV: 3 NL: 2.37E8
T: FTMS + p NSI Full ms [150.00-2000.00]
779.5833 z=7
909.3463 z=6
682.2612 z=8
606.5661 z=9
1091.0145 z=5
545.9097 z=10
921.1849 z=6
1363.5157 z=4
Relative Abundance
m/z

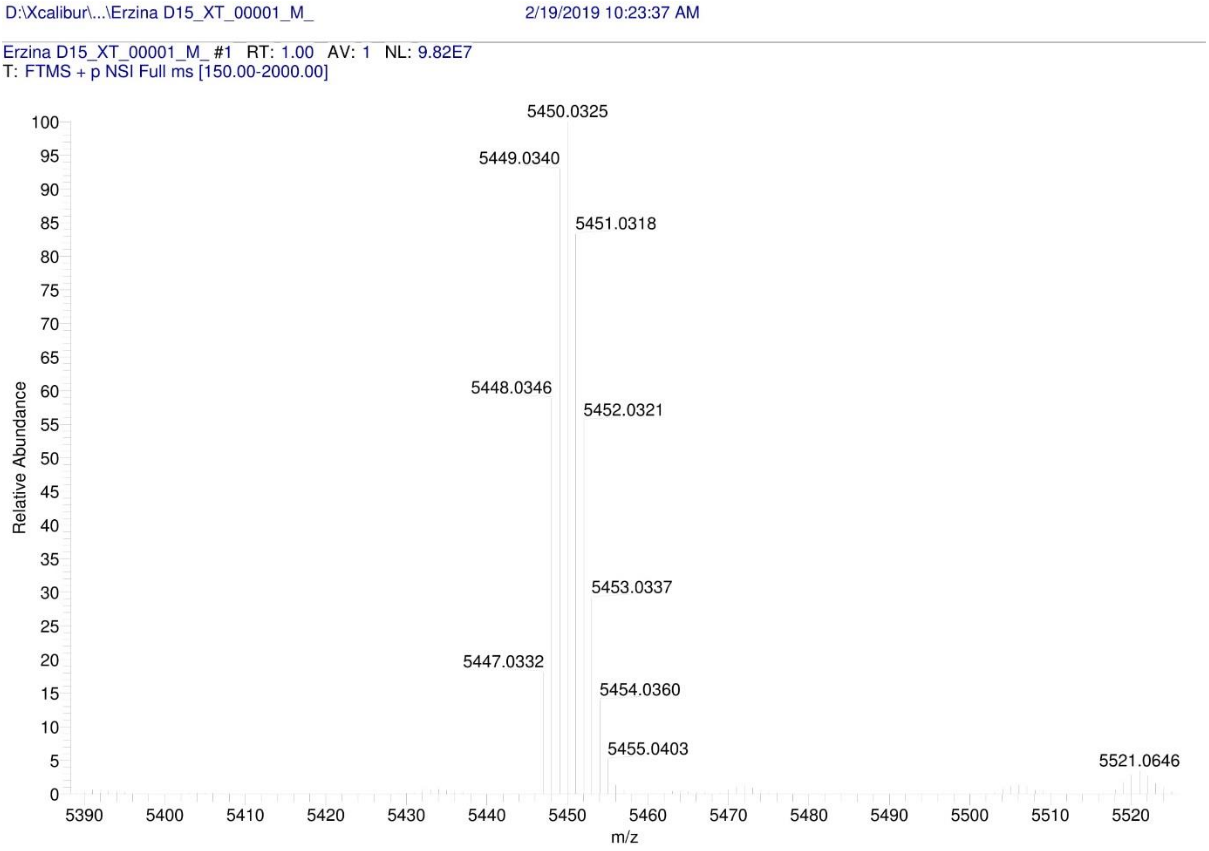
D:\Xcalibur\...\Erzina D15_XT_00001_M_
2/19/2019 10:23:37 AM
Erzina D15_XT_00001_M_ #1 RT: 1.00 AV: 1 NL: 9.82E7
T: FTMS + p NSI Full ms [150.00-2000.00]
5450.0325
5449.0340
5451.0318
5448.0346
5452.0321
5453.0337
5447.0332
5454.0360
5455.0403
5521.0646
Relative Abundance
m/z

3 ((AKA)$_8$(*K*YEK)$_4$(*K*EKA)$_2$*K*AKY-OH) was obtained from the CEM Liberty Blue synthetiser as foamy colourless solid after preparative RP-HPLC (23.7 mg, 1.1 µmol, 9.4%). Analytical RP-HPLC: t_R=1.24 min (100% A to 100% D in 5 min, λ= 214 nm). HRMS (ESI+): $C_{264}H_{448}N_{74}O_{70}$ calc./obs. 5775.3771/5775.3854 Da [M].

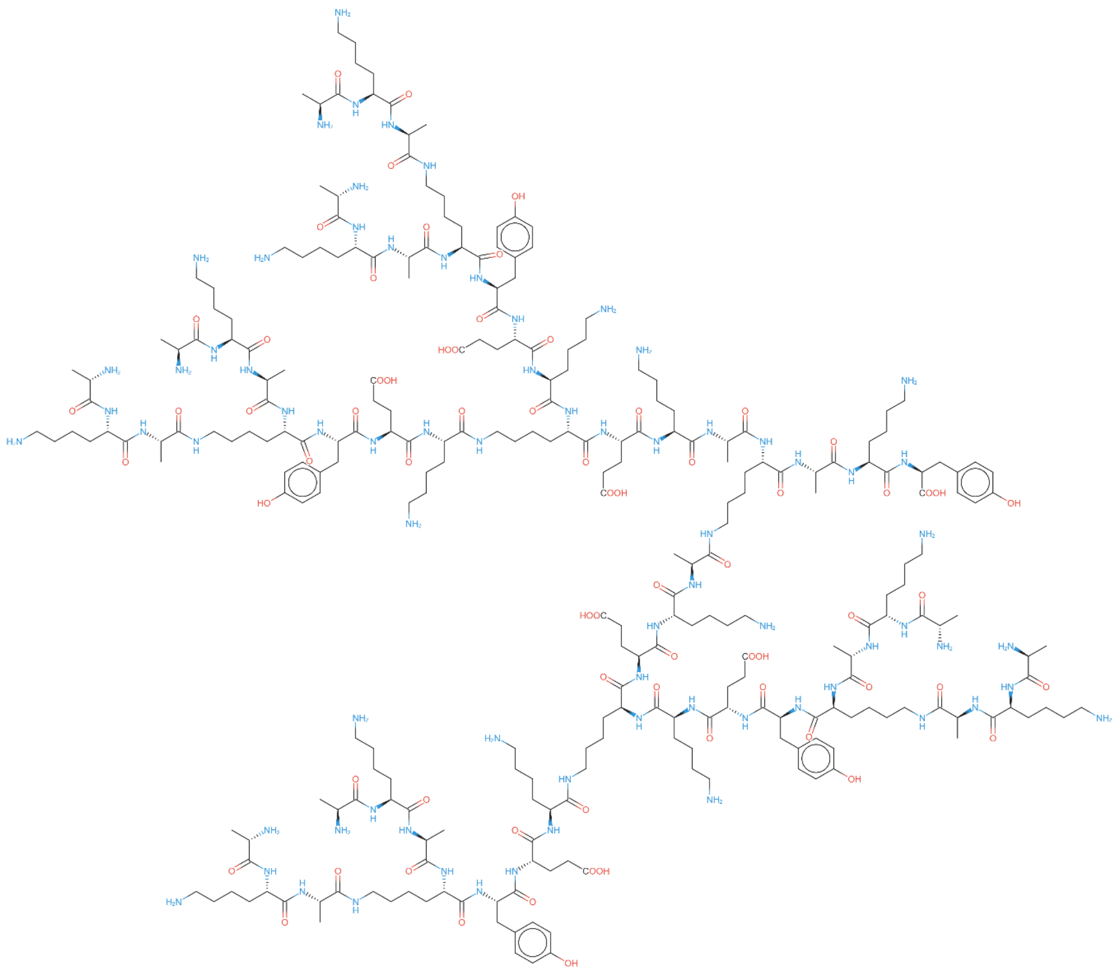

Analytical RP-HPLC:

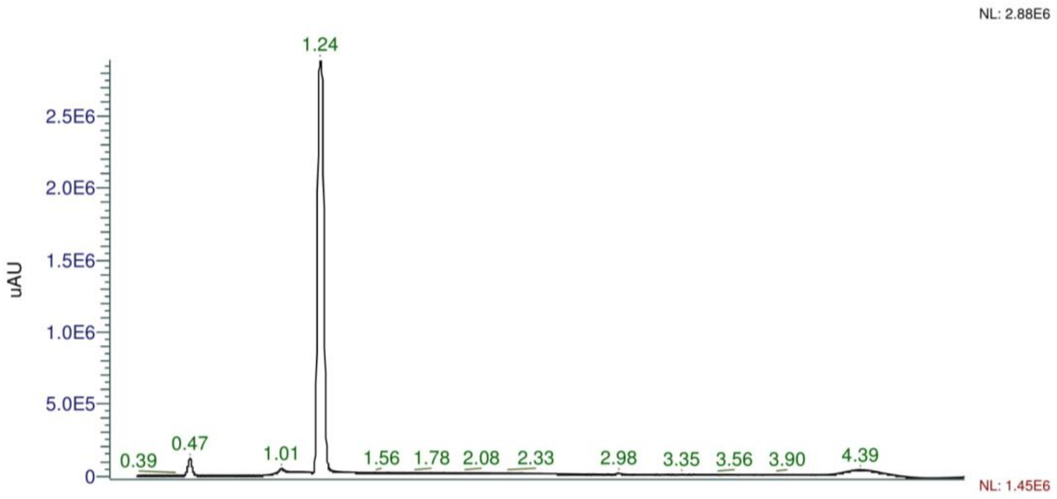

HRMS (NSI+):

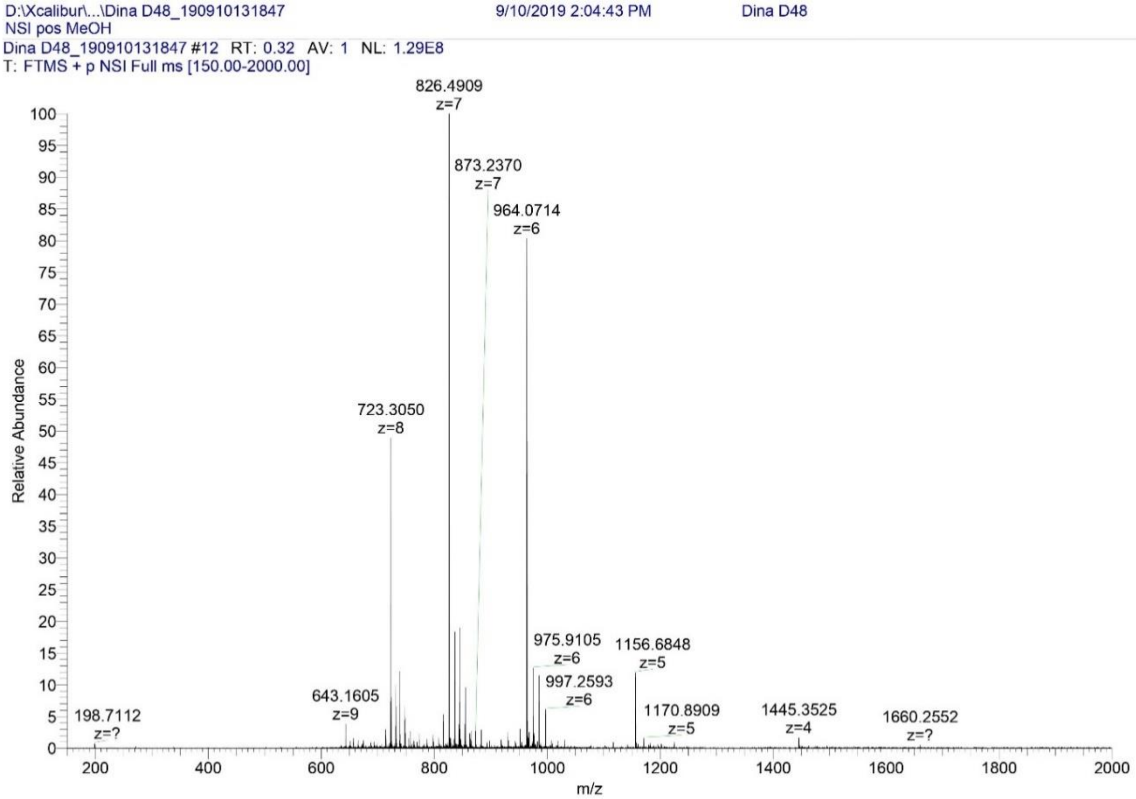
D:\Xcalibur\...\Dina D48_190910131847
9/10/2019 2:04:43 PM
Dina D48
NSI pos MeOH
Dina D48_190910131847 #12 RT: 0.32 AV: 1 NL: 1.29E8
T: FTMS + p NSI Full ms [150.00-2000.00]
826.4909 z=7
873.2370 z=7
964.0714 z=6
723.3050 z=8
975.9105 z=6
1156.6848 z=5
997.2593 z=6
643.1605 z=9
198.7112 z=?
1170.8909 z=5
1445.3525 z=4
1660.2552 z=?
Relative Abundance
m/z

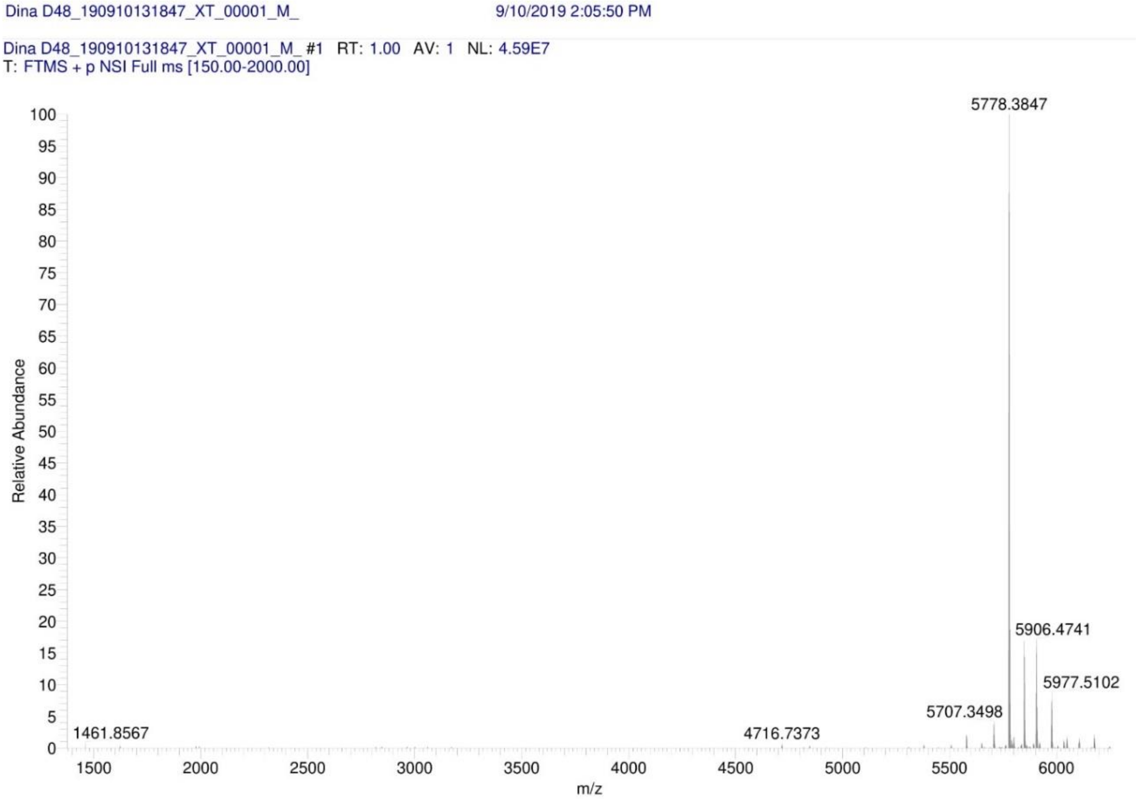
Dina D48_190910131847_XT_00001_M_
9/10/2019 2:05:50 PM
Dina D48_190910131847_XT_00001_M_ #1 RT: 1.00 AV: 1 NL: 4.59E7
T: FTMS + p NSI Full ms [150.00-2000.00]
5778.3847
5906.4741
5977.5102
5707.3498
1461.8567
4716.7373
Relative Abundance
m/z

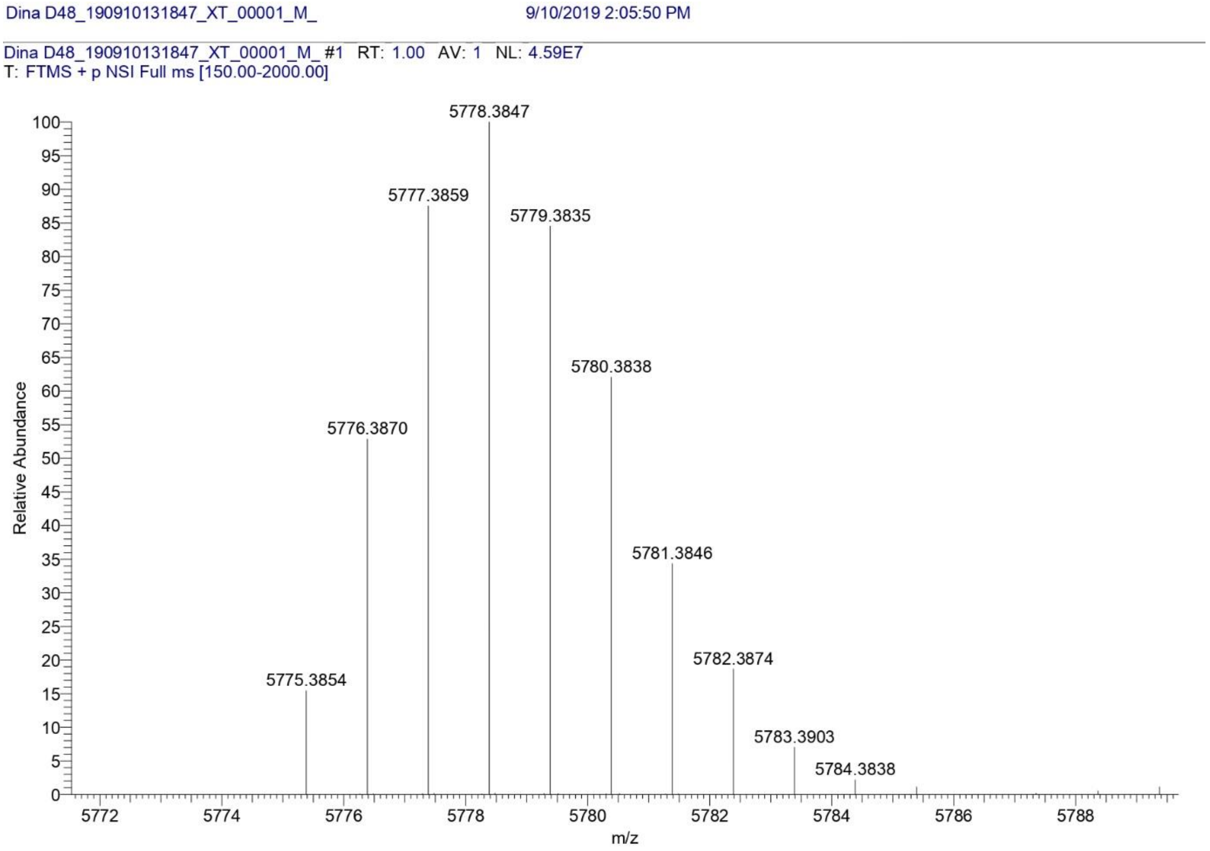
Dina D48_190910131847_XT_00001_M_
9/10/2019 2:05:50 PM
Dina D48_190910131847_XT_00001_M_ #1 RT: 1.00 AV: 1 NL: 4.59E7
T: FTMS + p NSI Full ms [150.00-2000.00]
5778.3847
5777.3859
5779.3835
5780.3838
5776.3870
5781.3846
5782.3874
5775.3854
5783.3903
5784.3838
Relative Abundance
m/z

Ac**3** **((AcAKA)$_8$(*K*YEK)$_4$(*K*EKA)$_2$*K*AKY-OH)** was obtained from the CEM Liberty Blue synthetiser as foamy colourless solid after preparative RP-HPLC (20.3 mg, 1.1 µmol, 8.7%). Analytical RP-HPLC: t_R=1.31 min (100% A to 100% D in 5 min, λ= 214 nm). HRMS (ESI+): $C_{280}H_{464}N_{74}O_{78}$ calc./obs. 6111.4616/6111.4644 Da [M].

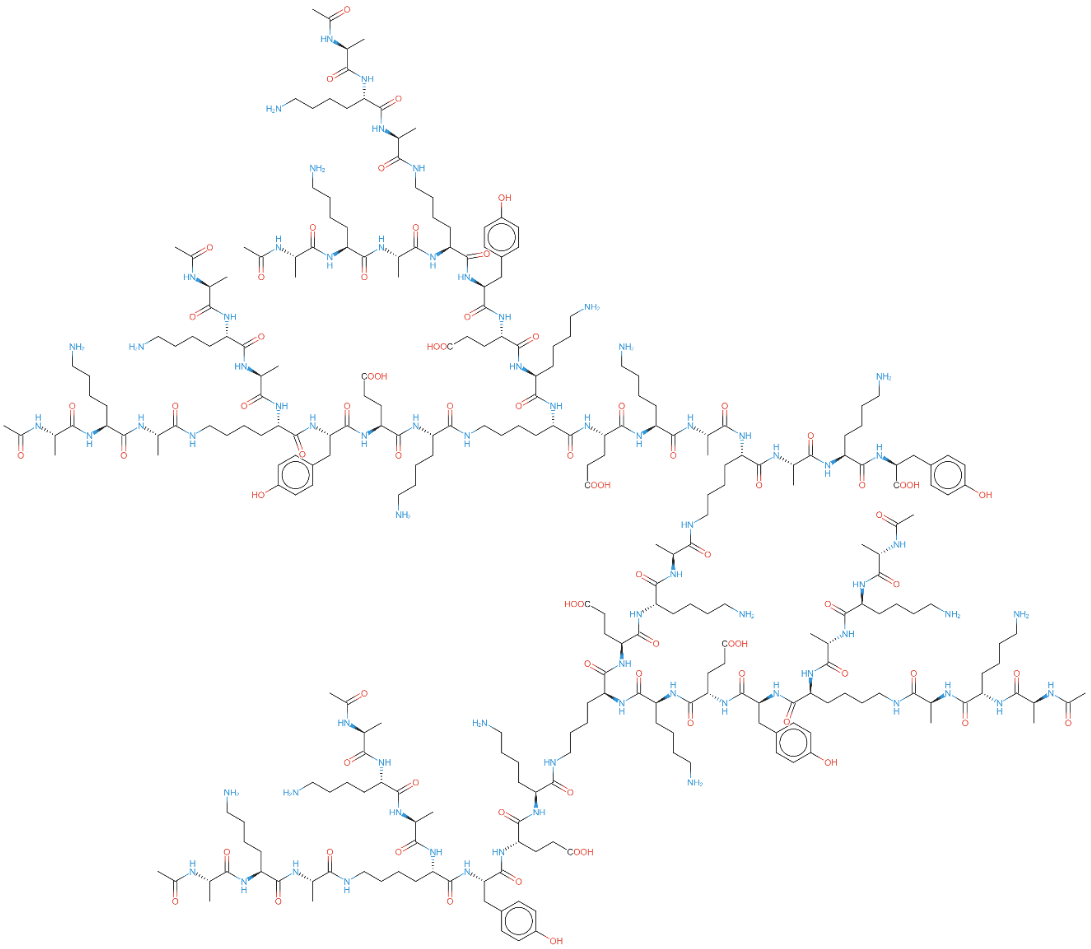

Analytical RP-HPLC:

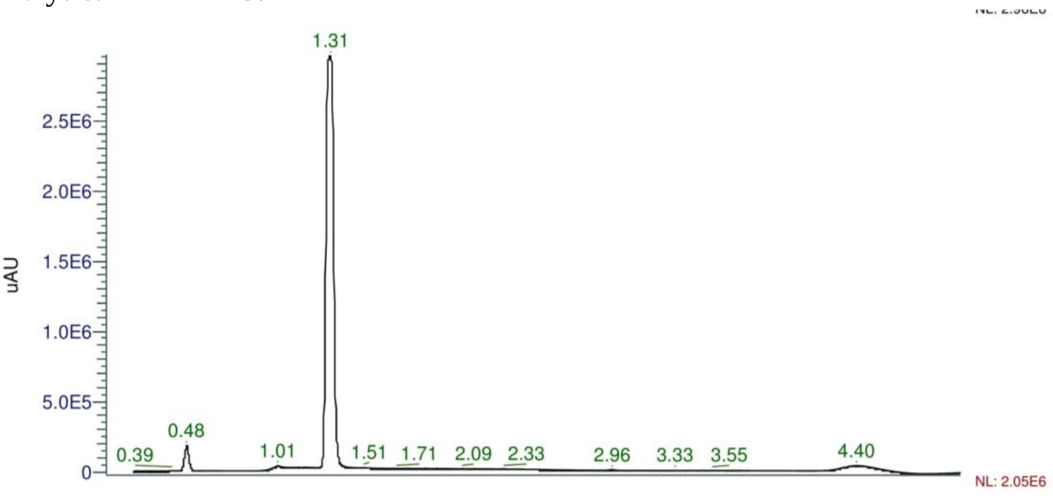

HRMS (NSI+):

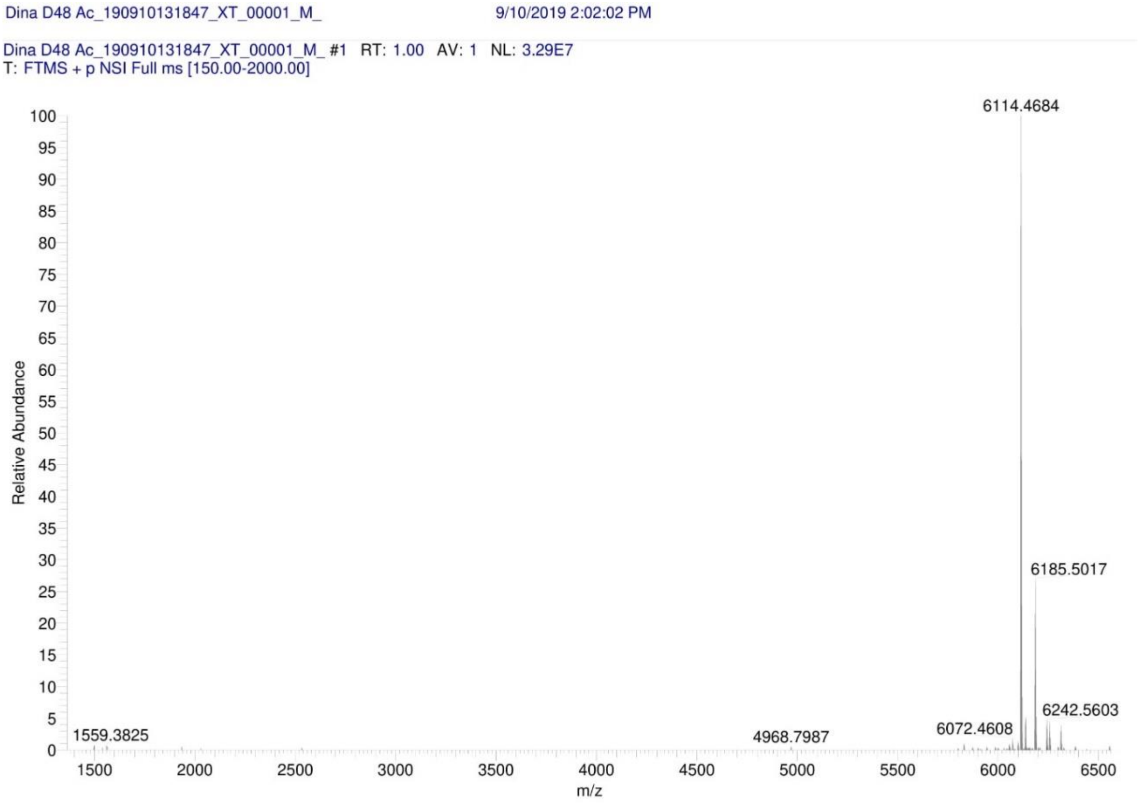

Dina D48 Ac_190910131847_XT_00001_M_
9/10/2019 2:02:02 PM
Dina D48 Ac_190910131847_XT_00001_M_ #1 RT: 1.00 AV: 1 NL: 3.29E7
T: FTMS + p NSI Full ms [150.00-2000.00]
6114.4684
6185.5017
6242.5603
6072.4608
4968.7987
1559.3825
Relative Abundance
m/z

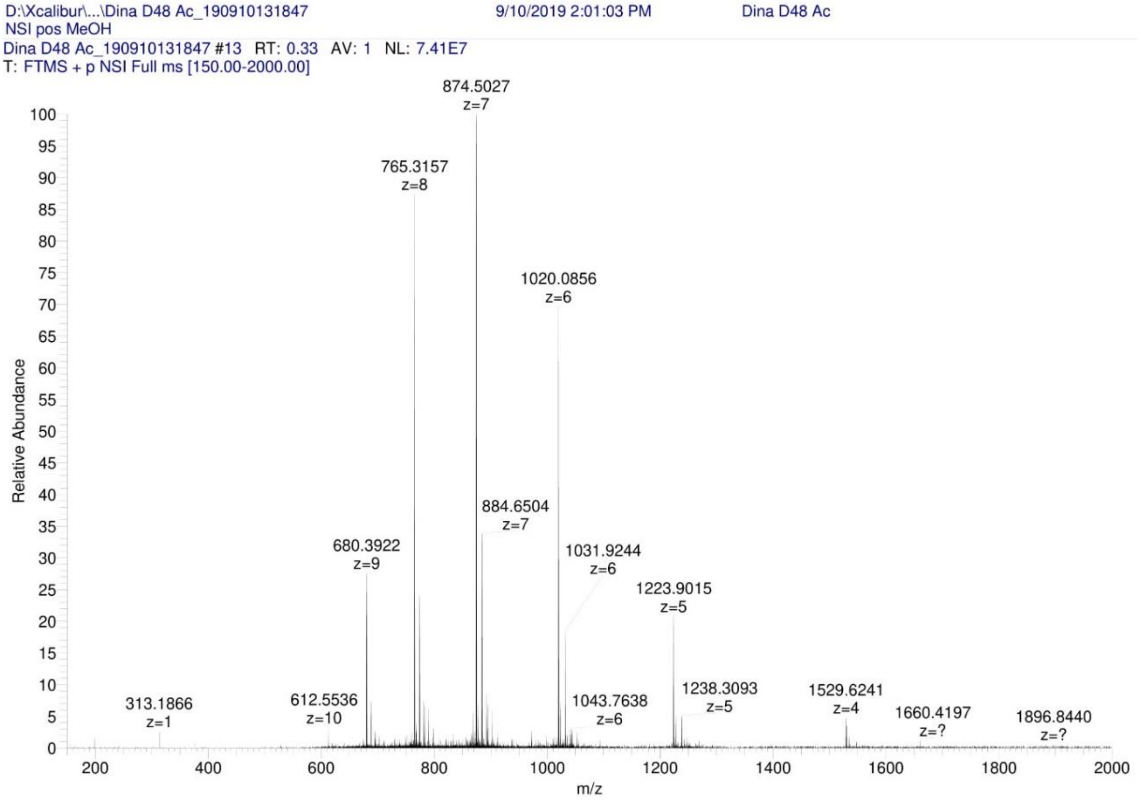

D:\Xcalibur\...\Dina D48 Ac_190910131847
9/10/2019 2:01:03 PM
Dina D48 Ac
NSI pos MeOH
Dina D48 Ac_190910131847 #13 RT: 0.33 AV: 1 NL: 7.41E7
T: FTMS + p NSI Full ms [150.00-2000.00]
874.5027 z=7
765.3157 z=8
1020.0856 z=6
884.6504 z=7
680.3922 z=9
1031.9244 z=6
1223.9015 z=5
1238.3093 z=5
1529.6241 z=4
313.1866 z=1
612.5536 z=10
1043.7638 z=6
1660.4197 z=?
1896.8440 z=?
Relative Abundance
m/z

Dina D48 Ac_190910131847_XT_00001_M_ 9/10/2019 2:02:02 PM

Dina D48 Ac_190910131847_XT_00001_M_ #1 RT: 1.00 AV: 1 NL: 3.29E7
T: FTMS + p NSI Full ms [150.00-2000.00]

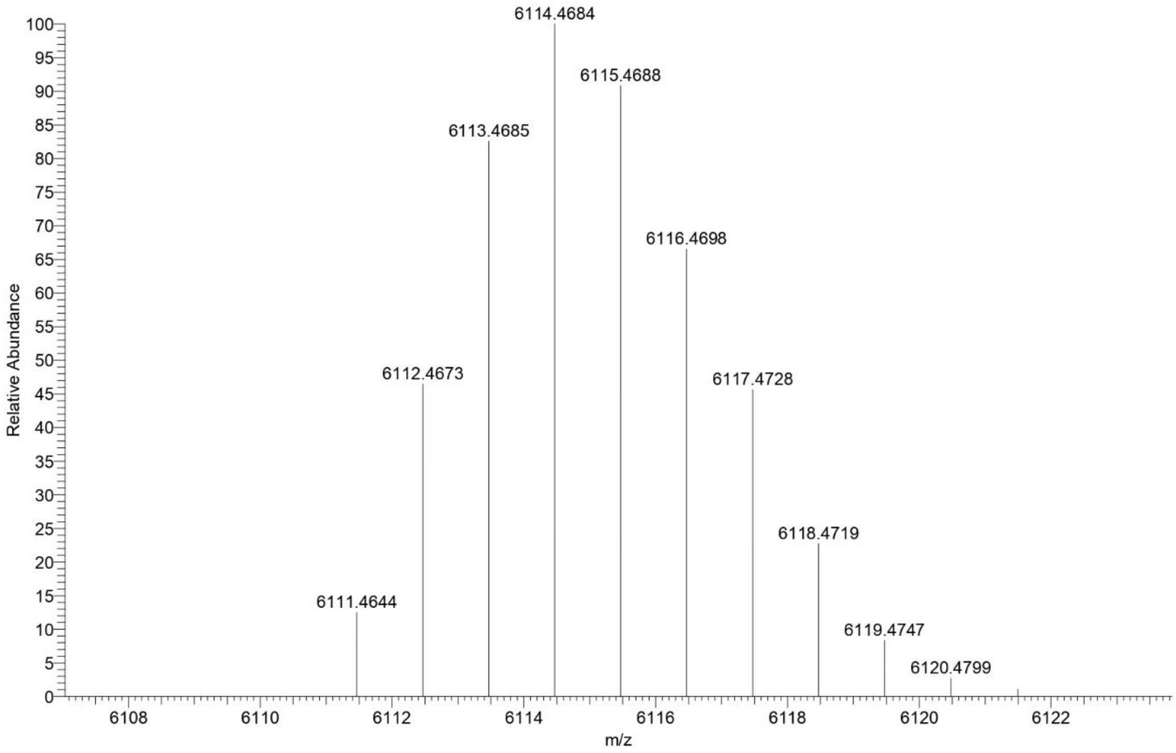

ClAc**3** **((ClAcAKA)$_8$(*K*YEK)$_4$(*K*EKA)$_2$*K*AKY-OH)** was obtained from the CEM Liberty Blue synthetiser as foamy colourless solid after preparative RP-HPLC (20.9 mg, 1.1 µmol, 8.6%). Analytical RP-HPLC: t_R=1.37 min (100% A to 100% D in 5 min, λ= 214 nm). $C_{280}H_{464}Cl_8N_{74}O_{78}$ calc./obs. 6383.1499/6384.1461 Da [M].

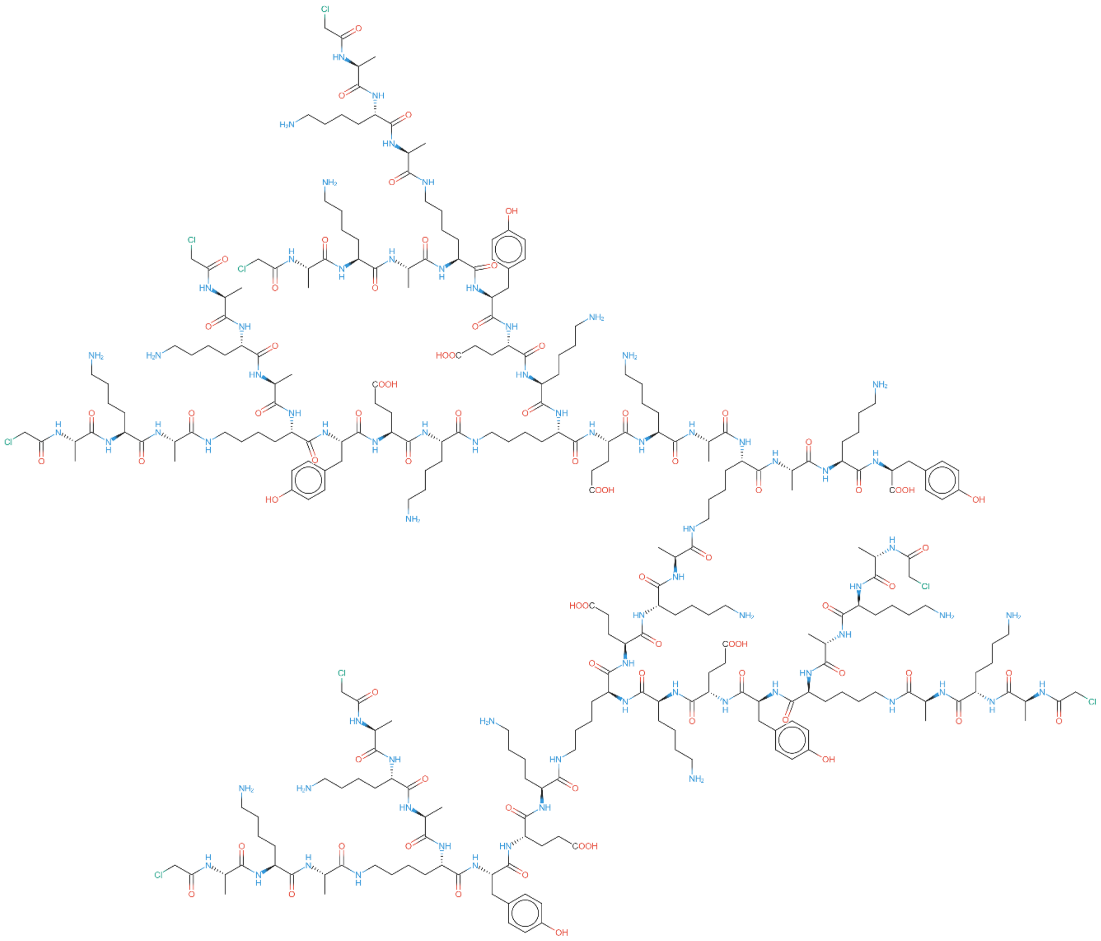

Analytical RP-HPLC:

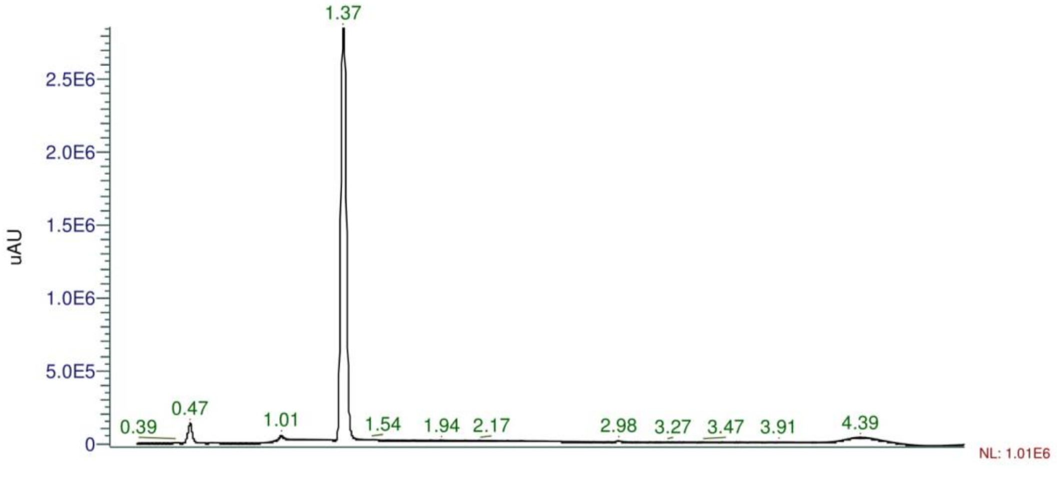

HRMS (NSI+):

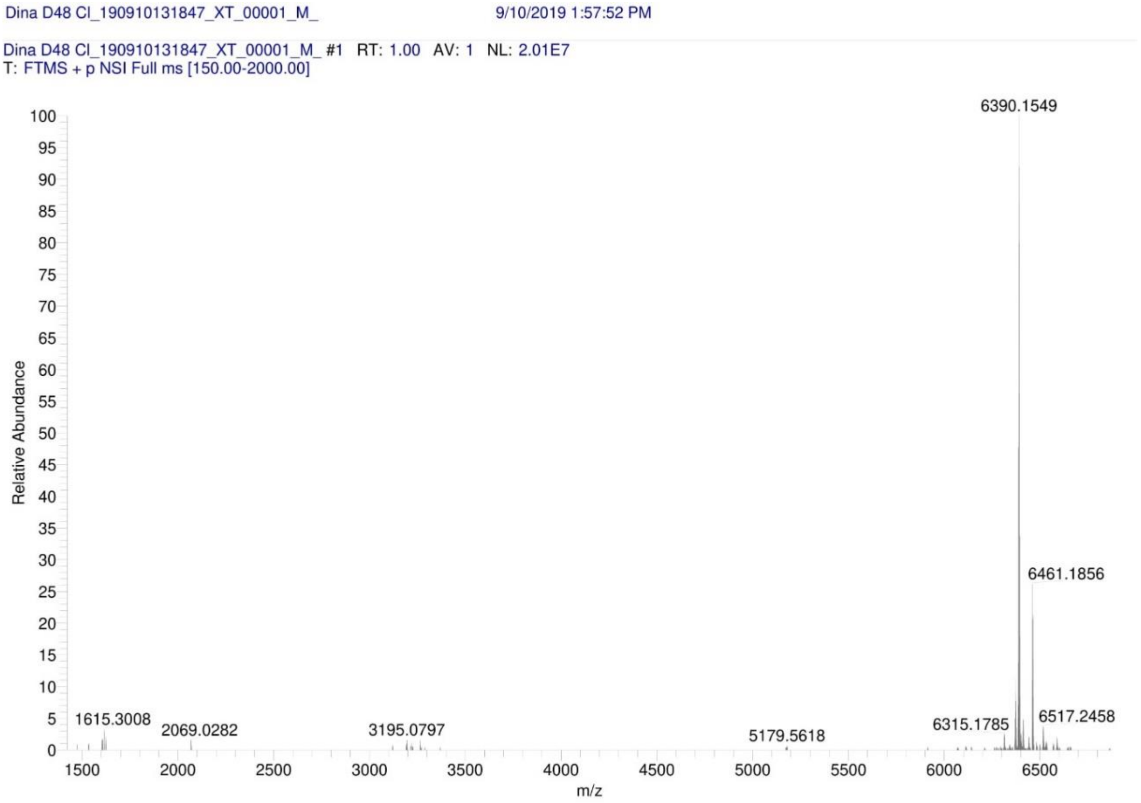

Dina D48 Cl_190910131847_XT_00001_M_
9/10/2019 1:57:52 PM
Dina D48 Cl_190910131847_XT_00001_M_ #1 RT: 1.00 AV: 1 NL: 2.01E7
T: FTMS + p NSI Full ms [150.00-2000.00]
6390.1549
6461.1856
1615.3008
2069.0282
3195.0797
5179.5618
6315.1785
6517.2458
Relative Abundance
m/z

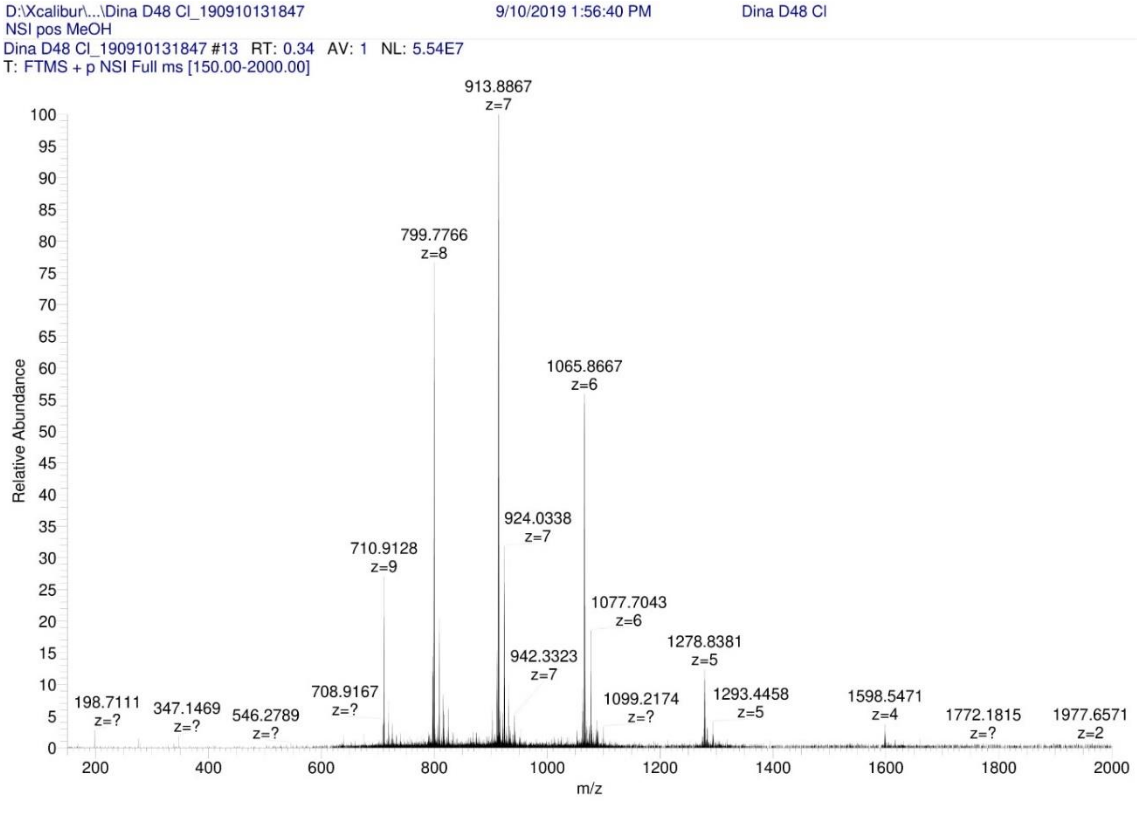

D:\Xcalibur\...\Dina D48 Cl_190910131847
NSI pos MeOH
9/10/2019 1:56:40 PM
Dina D48 Cl
Dina D48 Cl_190910131847 #13 RT: 0.34 AV: 1 NL: 5.54E7
T: FTMS + p NSI Full ms [150.00-2000.00]
913.8867 z=7
799.7766 z=8
1065.8667 z=6
924.0338 z=7
710.9128 z=9
1077.7043 z=6
1278.8381 z=5
942.3323 z=7
708.9167 z=?
1293.4458 z=5
1598.5471 z=4
1099.2174 z=?
198.7111 z=?
347.1469 z=?
546.2789 z=?
1772.1815 z=?
1977.6571 z=2
Relative Abundance
m/z

Dina D48 Cl_190910131847_XT_00001_M_ 9/10/2019 1:57:52 PM

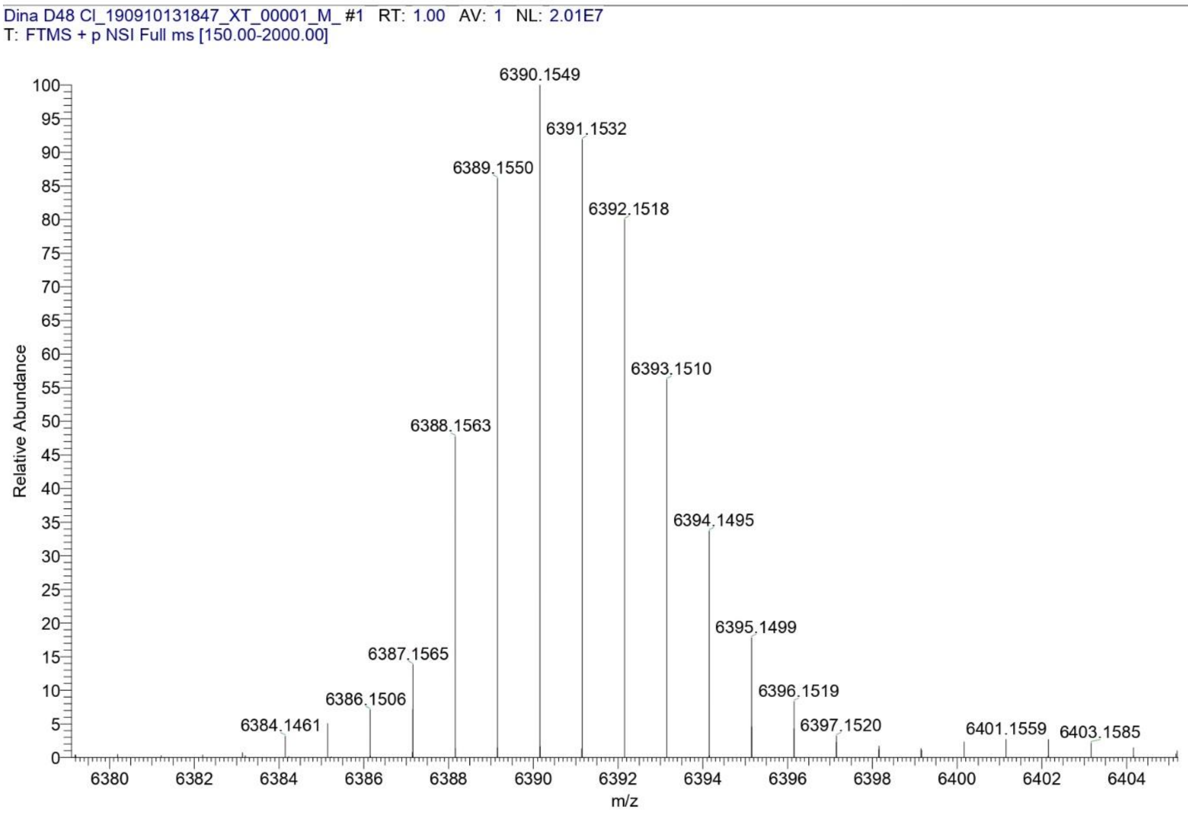

4 ((KA)$_8$(*K*KAKE)$_4$(*K*YKAKA)$_2$*K*AYKKA-OH) was obtained after manual synthesis as foamy colourless solid after preparative RP-HPLC (127 mg, 1.0 µmol, 9.0%). Analytical RP-HPLC: t_R=1.20 min (100% A to 100% D in 5 min, λ= 214 nm). HRMS (ESI+): $C_{275}H_{495}N_{83}O_{66}$ calc./obs. 6016.7929/6016.7972 Da [M].

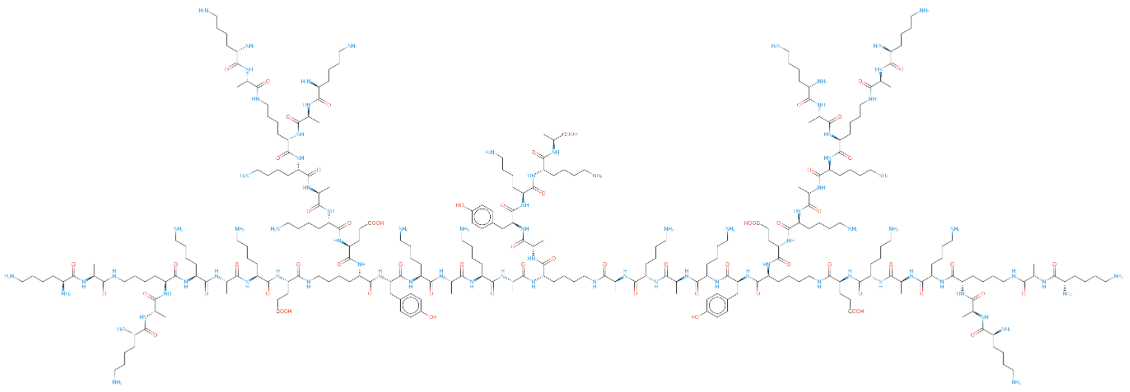

Analytical RP-HPLC:

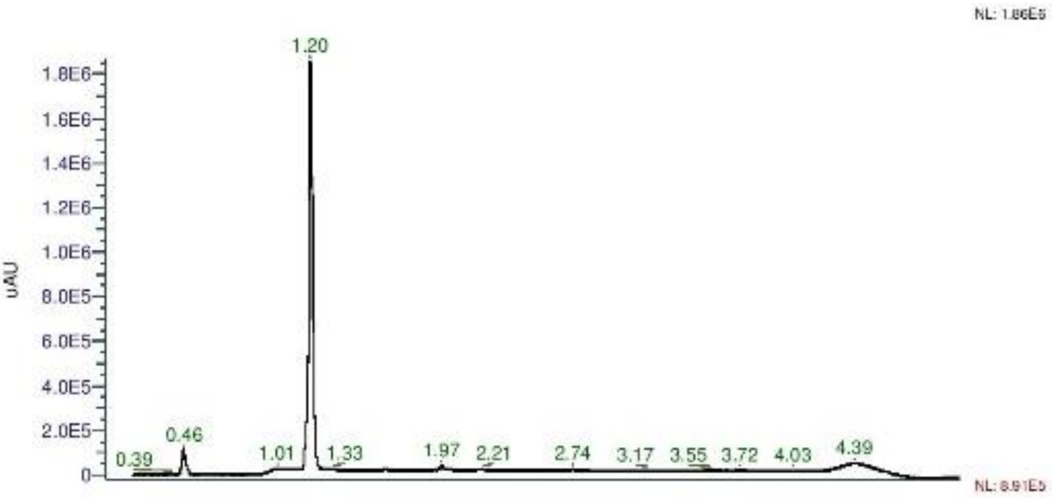

HRMS (NSI+):

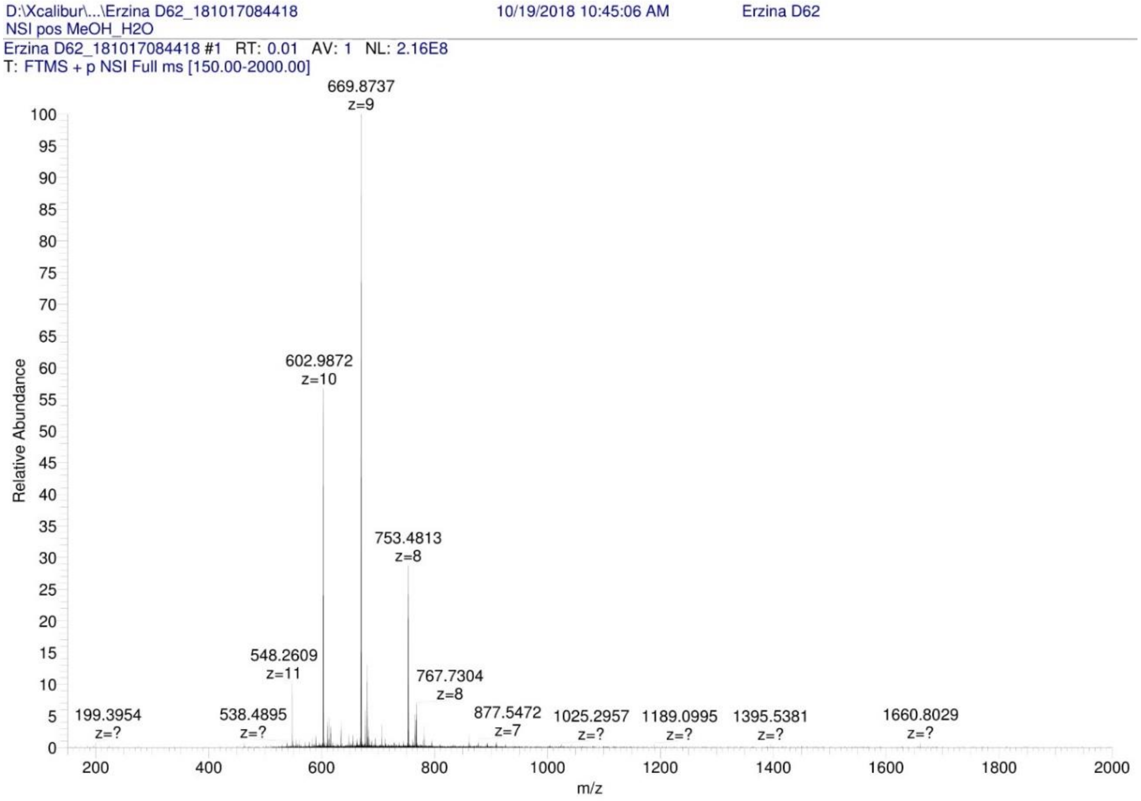
D:\Xcalibur\...\Erzina D62_181017084418
10/19/2018 10:45:06 AM
Erzina D62
NSI pos MeOH_H2O
Erzina D62_181017084418 #1 RT: 0.01 AV: 1 NL: 2.16E8
T: FTMS + p NSI Full ms [150.00-2000.00]
669.8737
z=9
602.9872
z=10
753.4813
z=8
548.2609
z=11
767.7304
z=8
199.3954
z=?
538.4895
z=?
877.5472
z=7
1025.2957
z=?
1189.0995
z=?
1395.5381
z=?
1660.8029
z=?
Relative Abundance
m/z

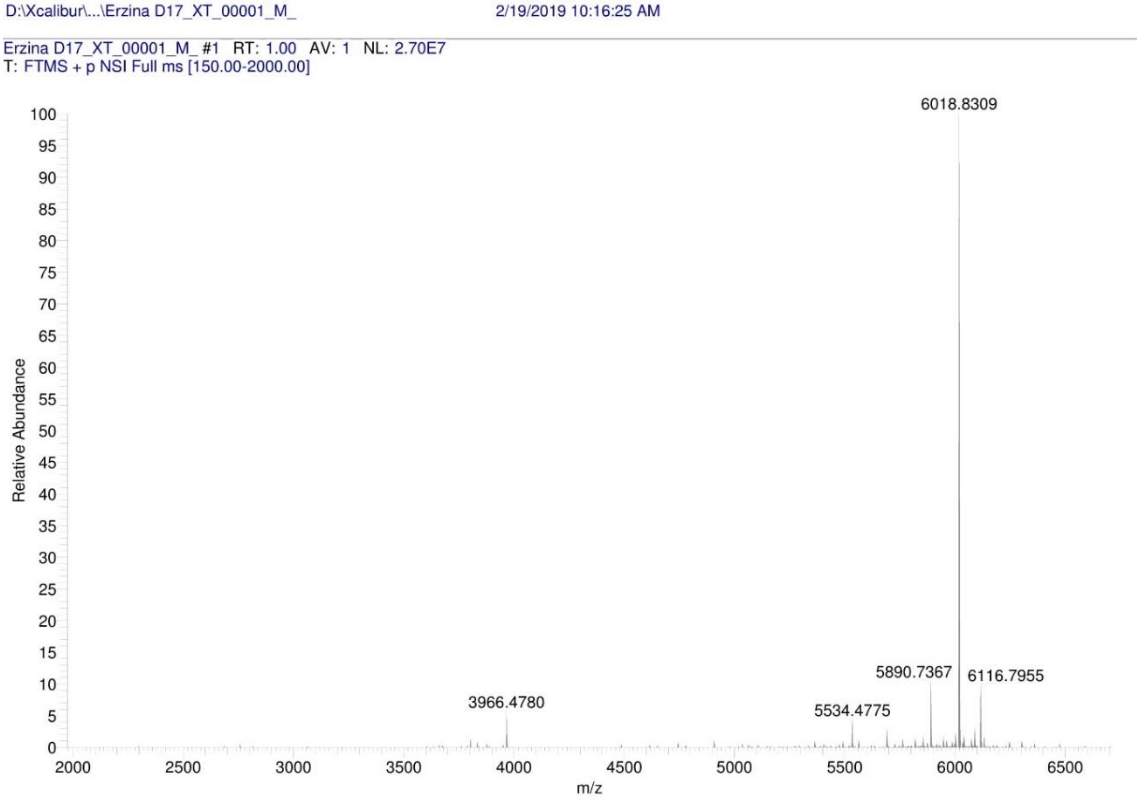
D:\Xcalibur\...\Erzina D17_XT_00001_M_
2/19/2019 10:16:25 AM
Erzina D17_XT_00001_M_ #1 RT: 1.00 AV: 1 NL: 2.70E7
T: FTMS + p NSI Full ms [150.00-2000.00]
6018.8309
5890.7367
6116.7955
3966.4780
5534.4775
Relative Abundance
m/z

Dina D62_190910103111_XT_00001_M_ 9/10/2019 10:50:53 AM

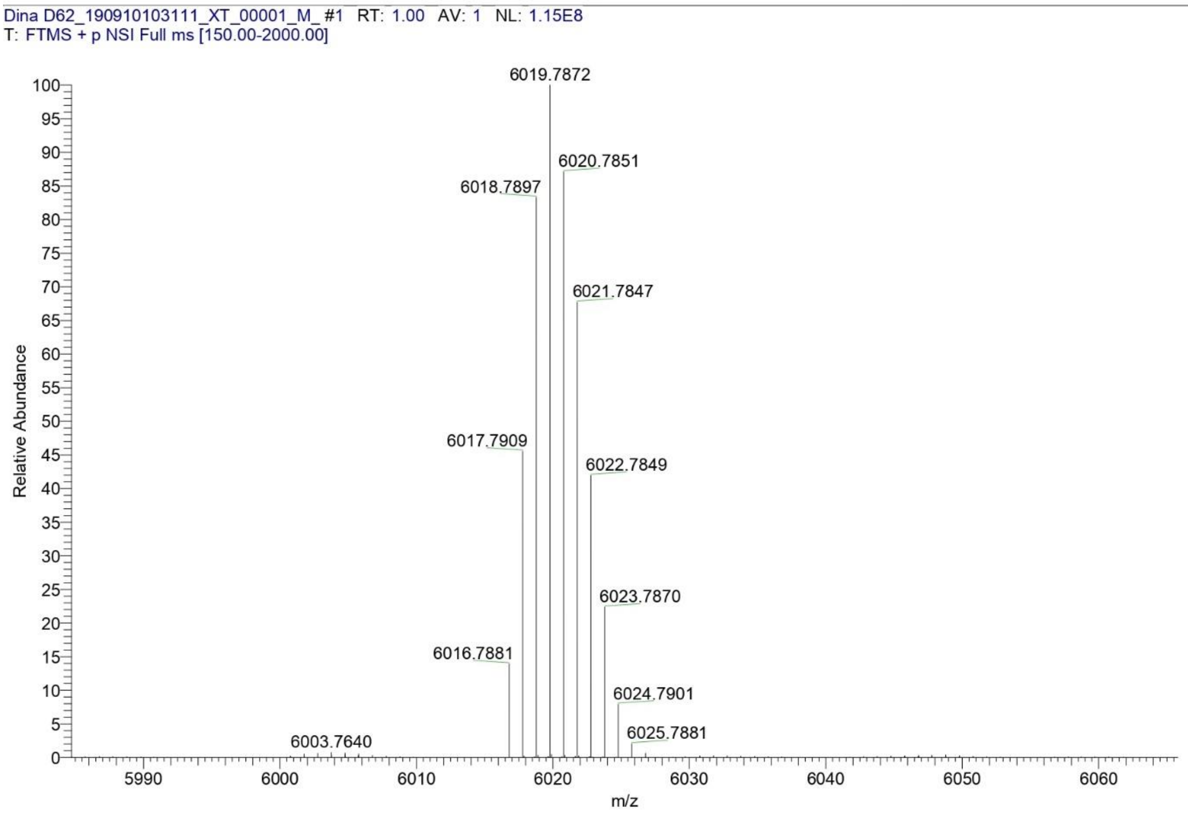

Ac**4 ((AcKA)$_8$(*K*KAKE)$_4$(*K*YKAKA)$_2$*K*AYKKA-OH)** was obtained from the CEM Liberty Blue synthetiser as foamy colourless solid after preparative RP-HPLC (52.8 mg, 2.4 µmol, 19.9%). Analytical RP-HPLC: t_R=1.25 min (100% A to 100% D in 5 min, λ= 214 nm). HRMS (ESI+): $C_{291}H_{511}N_{83}O_{74}$ calc./obs. 6352.8774/6352.8913 Da [M].

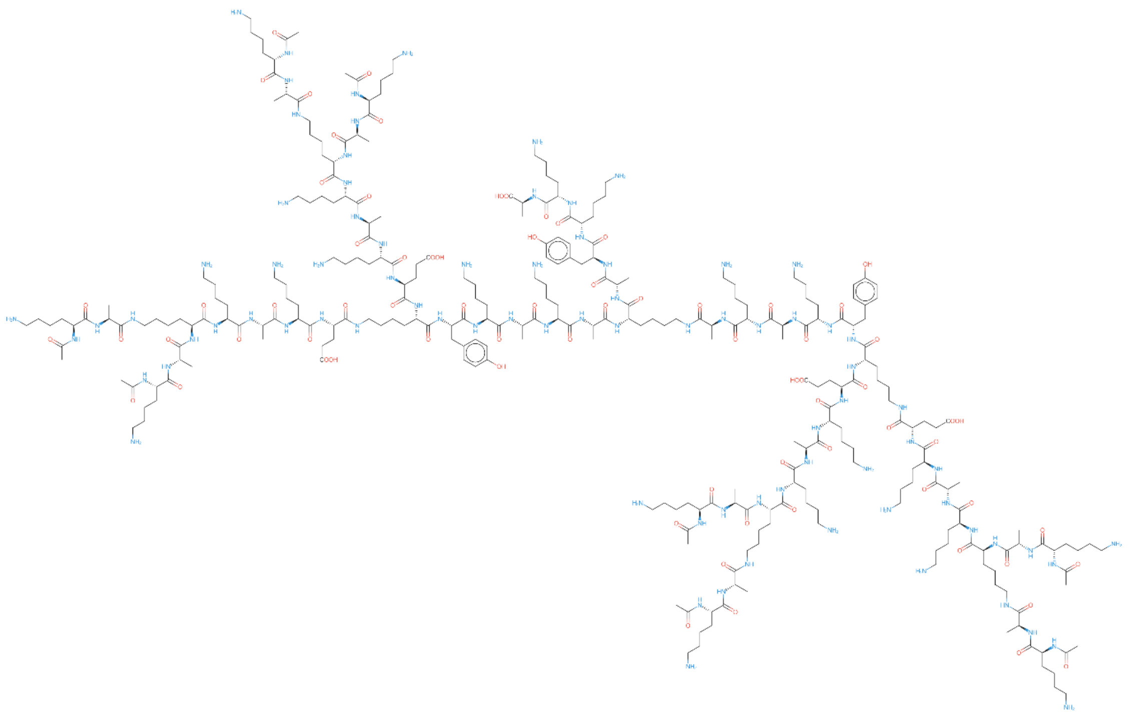

Analytical RP-HPLC:

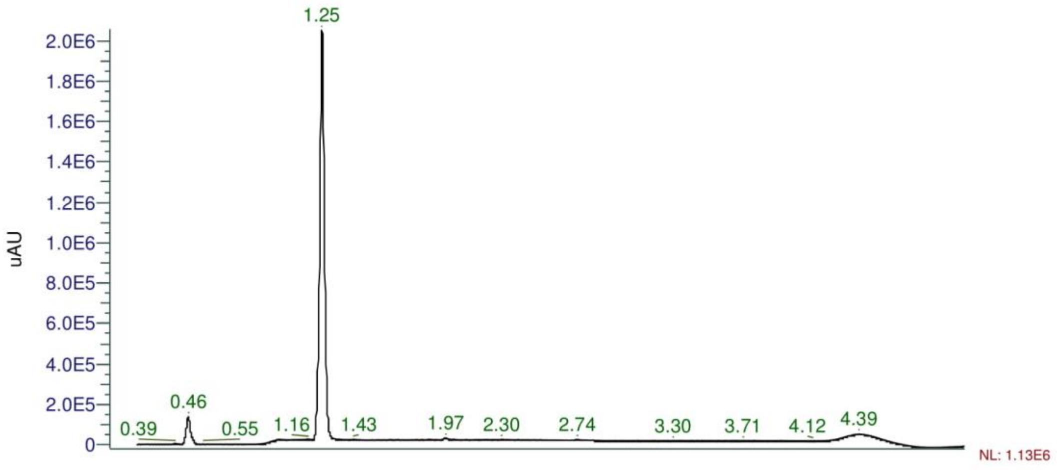

HRMS (NSI+):

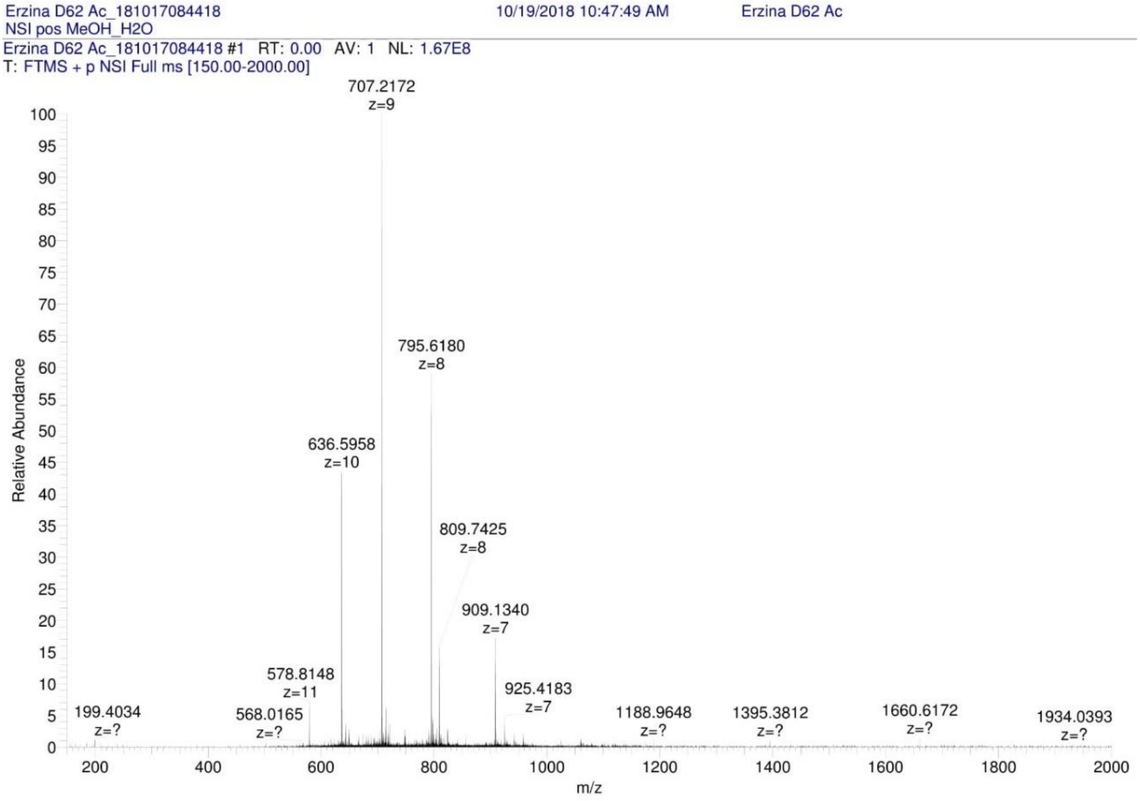
Erzina D62 Ac_181017084418
10/19/2018 10:47:49 AM
Erzina D62 Ac
NSI pos MeOH_H2O
Erzina D62 Ac_181017084418 #1 RT: 0.00 AV: 1 NL: 1.67E8
T: FTMS + p NSI Full ms [150.00-2000.00]
707.2172 z=9
795.6180 z=8
636.5958 z=10
809.7425 z=8
909.1340 z=7
578.8148 z=11
925.4183 z=7
199.4034 z=?
568.0165 z=?
1188.9648 z=?
1395.3812 z=?
1660.6172 z=?
1934.0393 z=?
Relative Abundance
m/z

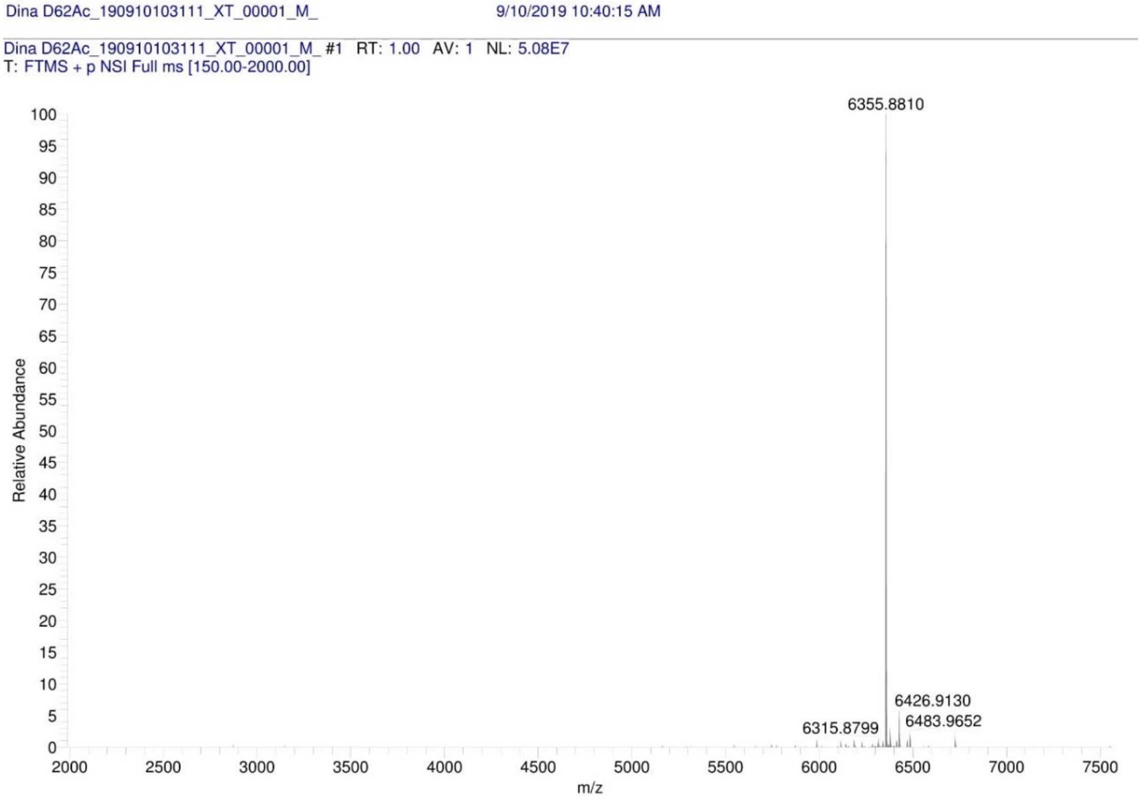
Dina D62Ac_190910103111_XT_00001_M_
9/10/2019 10:40:15 AM
Dina D62Ac_190910103111_XT_00001_M_ #1 RT: 1.00 AV: 1 NL: 5.08E7
T: FTMS + p NSI Full ms [150.00-2000.00]
6355.8810
6426.9130
6483.9652
6315.8799
Relative Abundance
m/z

Dina D62Ac_190910103111_XT_00001_M_ 9/10/2019 10:40:15 AM

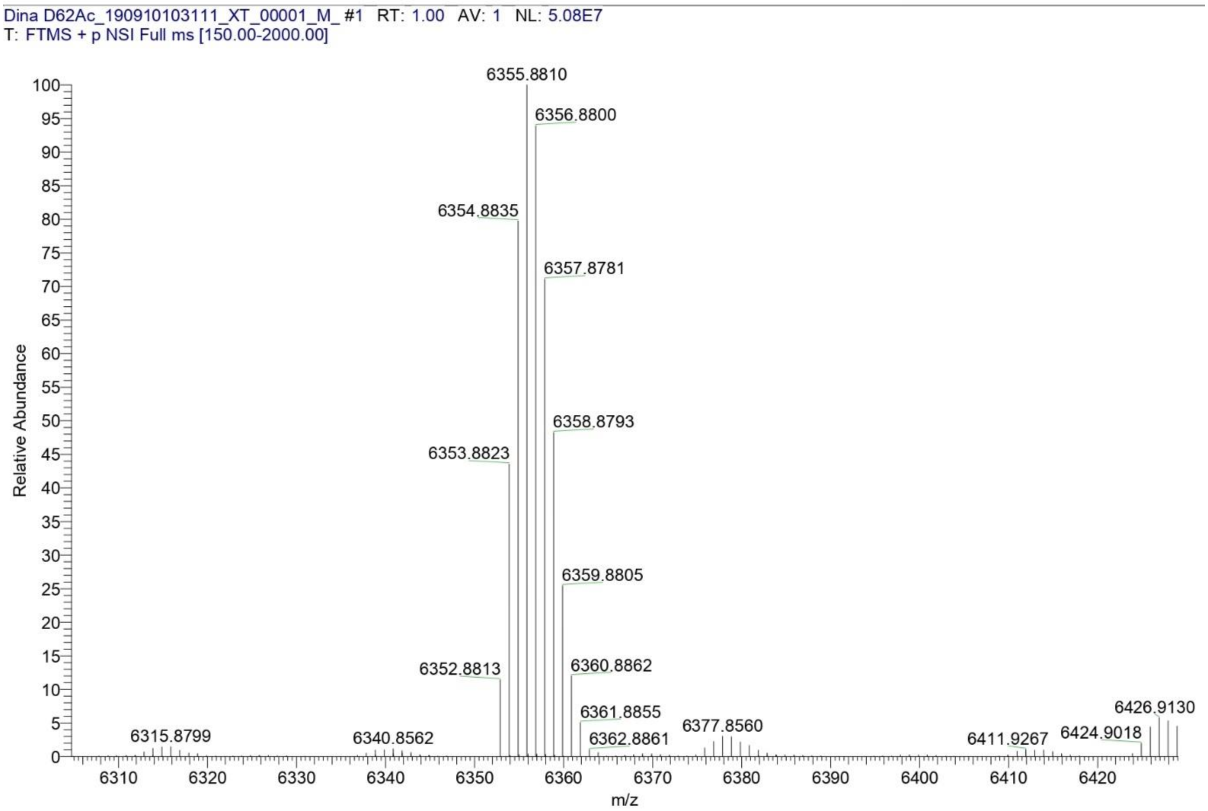

Fum**4 ((FumKA)$_8$(*K*KAKE)$_4$(*K*YKAKA)$_2$*K*AYKKA-OH)** was obtained from the CEM Liberty Blue synthetiser as foamy colourless solid after preparative RP-HPLC (51.8 mg, 1.9 µmol, 18.1%). Analytical RP-HPLC: t_R=1.42 min (100% A to 100% D in 5 min, λ= 214 nm). HRMS (ESI+): $C_{323}H_{543}N_{83}O_{90}$ calc./obs. 7025.0465/7025.0531 Da [M].

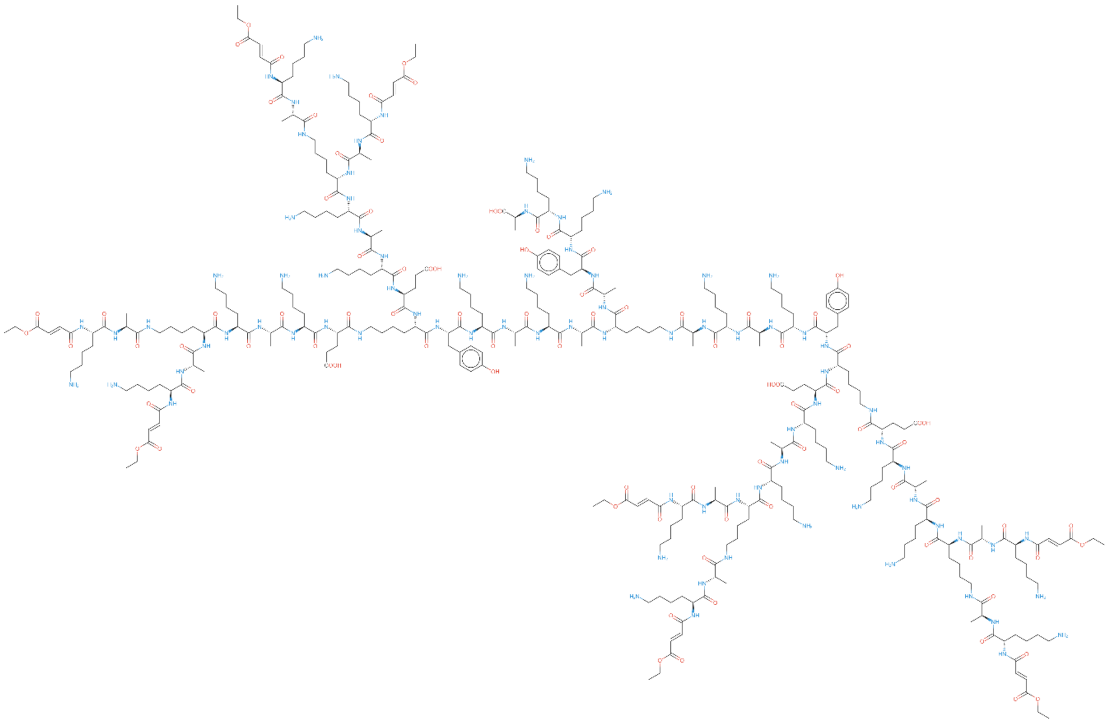

Analytical RP-HPLC:

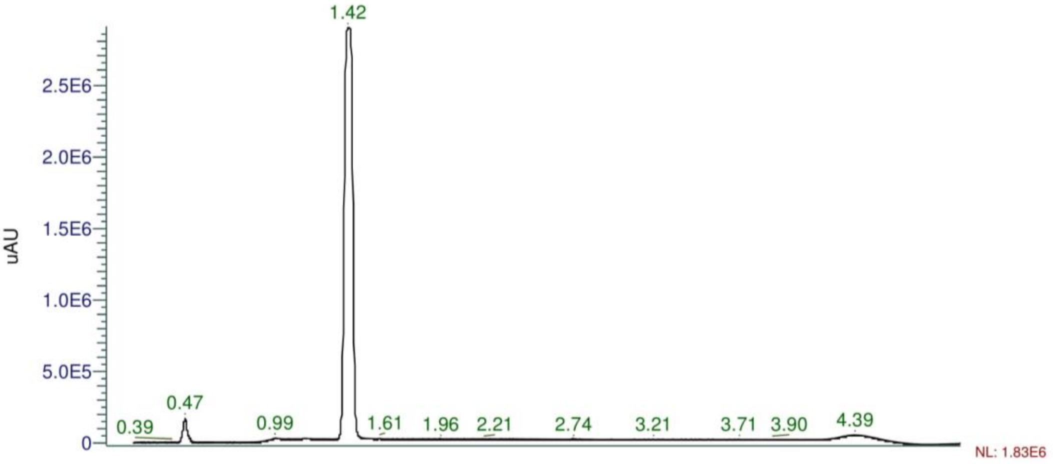

HRMS (NSI+):

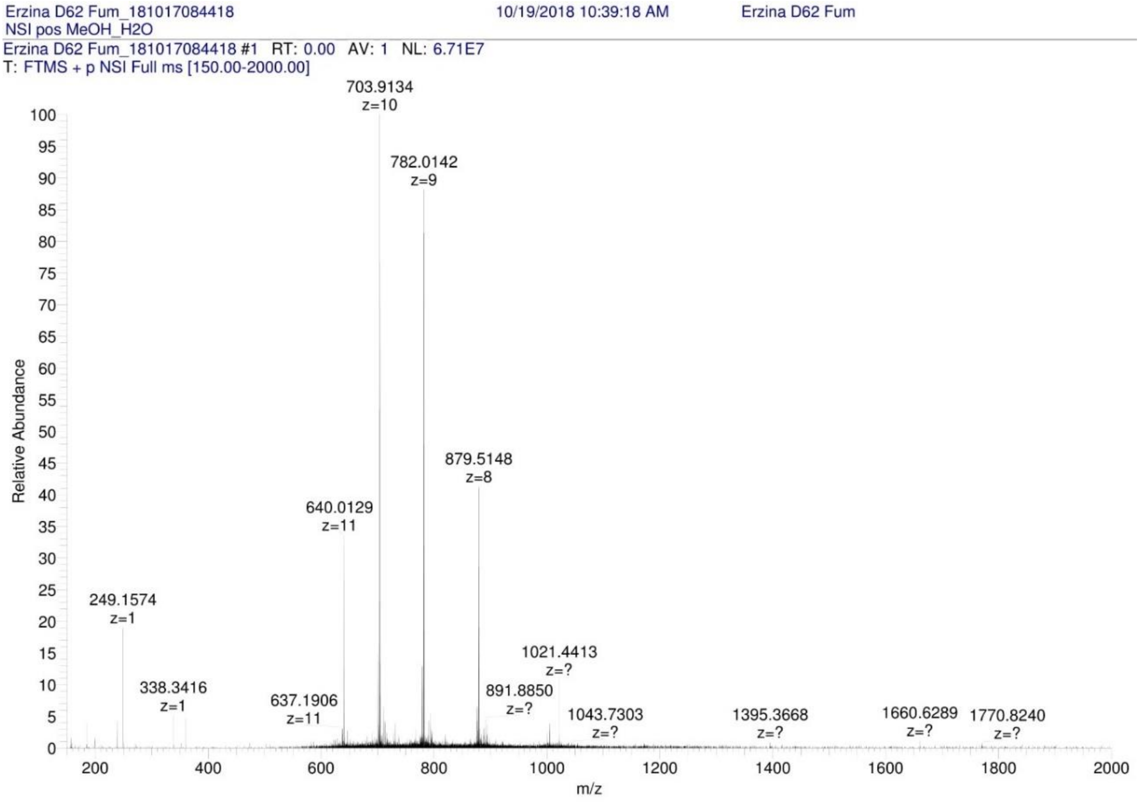
Erzina D62 Fum_181017084418
10/19/2018 10:39:18 AM
Erzina D62 Fum
NSI pos MeOH_H2O
Erzina D62 Fum_181017084418 #1 RT: 0.00 AV: 1 NL: 6.71E7
T: FTMS + p NSI Full ms [150.00-2000.00]
703.9134 z=10
782.0142 z=9
879.5148 z=8
640.0129 z=11
249.1574 z=1
338.3416 z=1
637.1906 z=11
891.8850 z=?
1021.4413 z=?
1043.7303 z=?
1395.3668 z=?
1660.6289 z=?
1770.8240 z=?
Relative Abundance
m/z

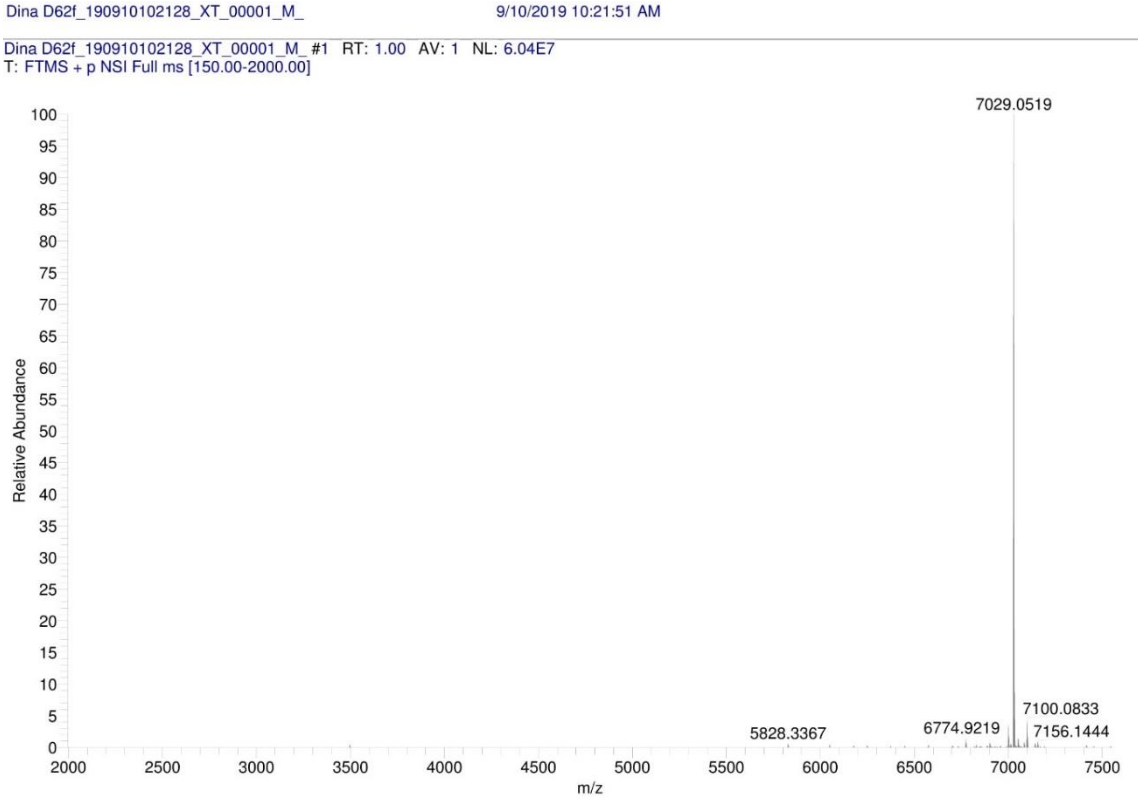
Dina D62f_190910102128_XT_00001_M_
9/10/2019 10:21:51 AM
Dina D62f_190910102128_XT_00001_M_ #1 RT: 1.00 AV: 1 NL: 6.04E7
T: FTMS + p NSI Full ms [150.00-2000.00]
7029.0519
5828.3367
6774.9219
7100.0833
7156.1444
Relative Abundance
m/z

Dina D62f_190910102128_XT_00001_M_ 9/10/2019 10:21:51 AM

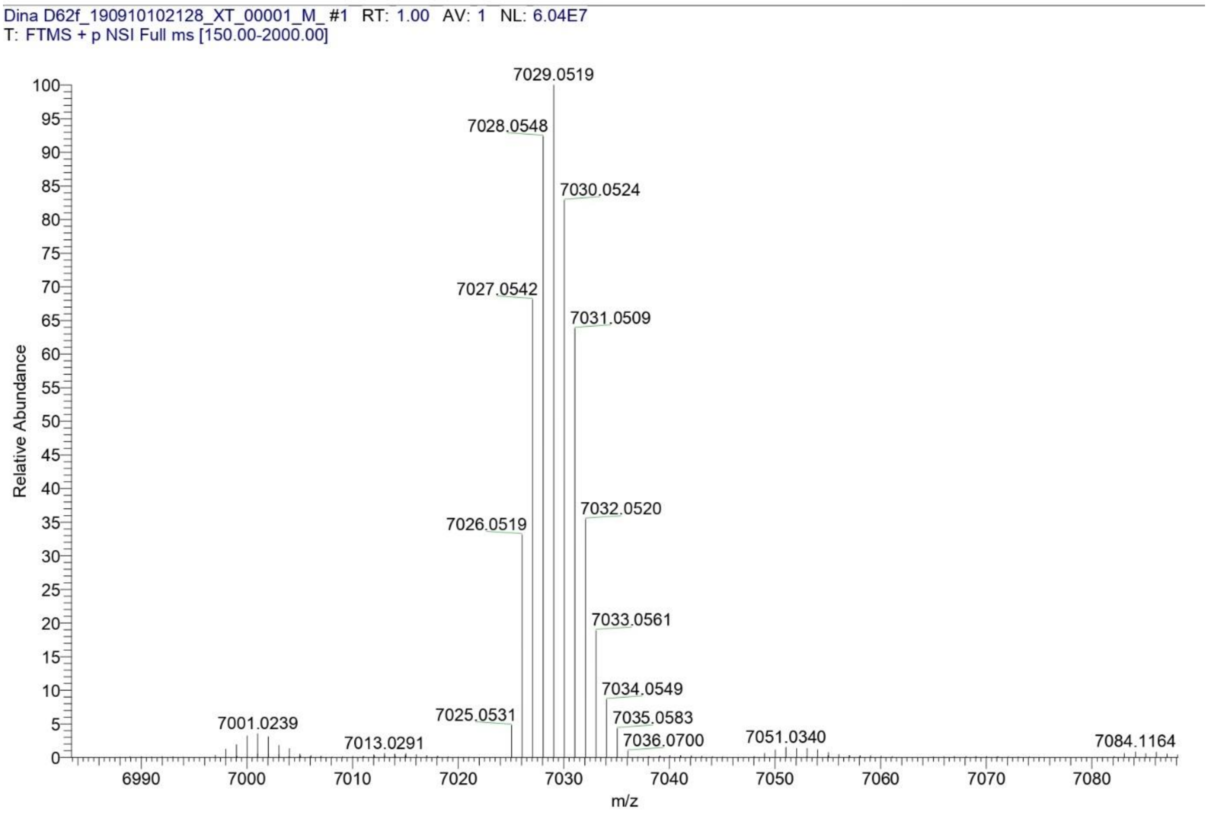

5 ((AK)$_8$(*K*AKAKY)$_4$(*K*AKEYEY)$_2$*K*AKEYEY-NH$_2$) was obtained after manual synthesis as foamy colourless solid after preparative RP-HPLC (51.3 mg, 0.6 µmol, 5.8%). Analytical RP-HPLC: t_R=1.25 min (100% A to 100% D in 5 min, λ= 214 nm). HRMS (ESI+): $C_{323}H_{544}N_{90}O_{77}$ calc./obs. 6916.1419/6916.1596 Da [M].

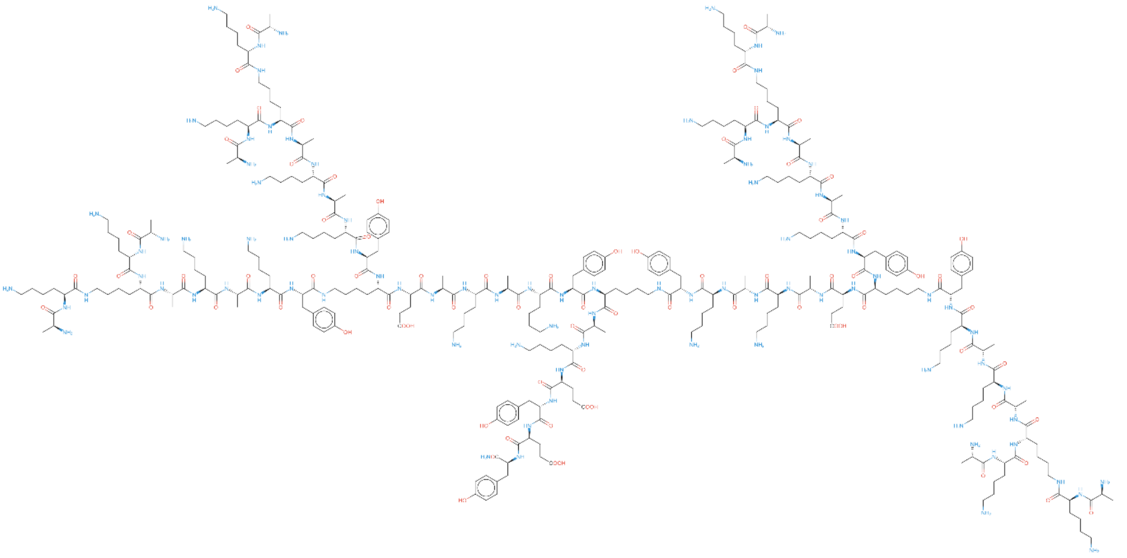

Analytical RP-HPLC:

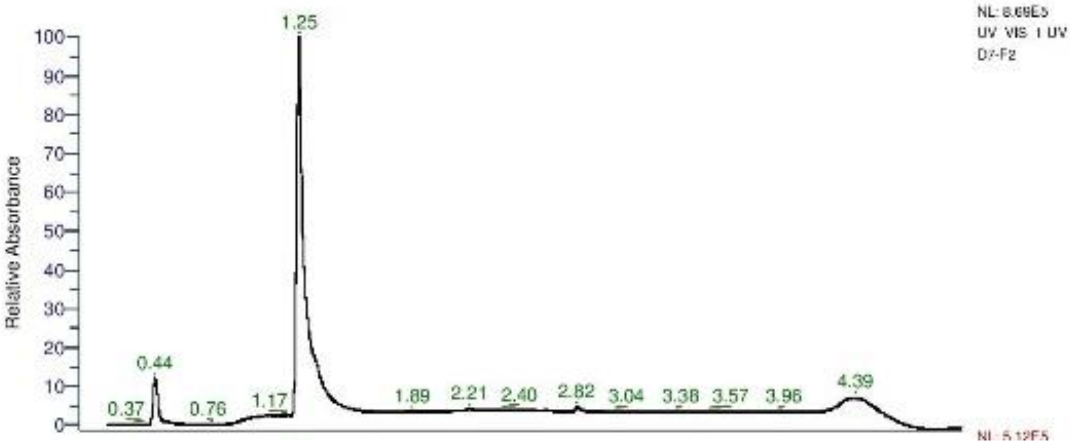

HRMS (NSI+):

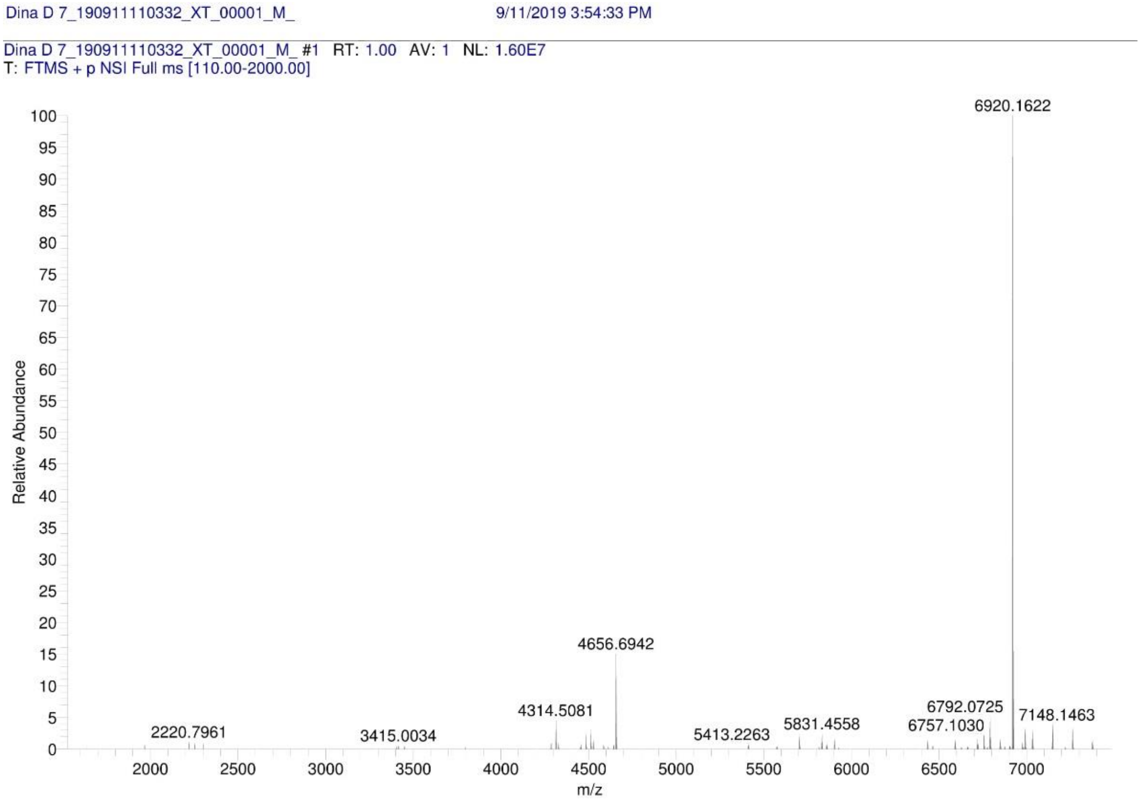
Dina D 7_190911110332_XT_00001_M_
9/11/2019 3:54:33 PM
Dina D 7_190911110332_XT_00001_M_ #1 RT: 1.00 AV: 1 NL: 1.60E7
T: FTMS + p NSI Full ms [110.00-2000.00]
6920.1622
4656.6942
4314.5081
2220.7961
3415.0034
5413.2263
5831.4558
6792.0725
6757.1030
7148.1463
Relative Abundance
m/z

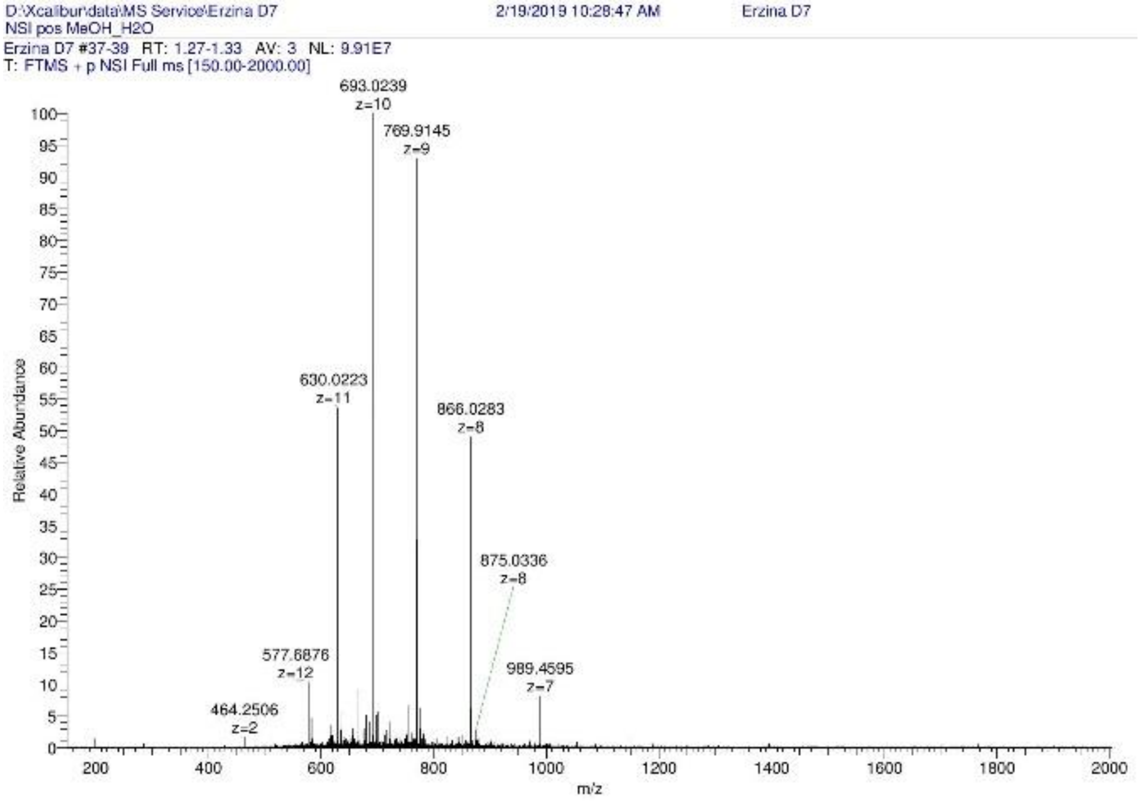
D:\Xcalibur\data\MS Service\Erzina D7
2/19/2019 10:28:47 AM
Erzina D7
NSI pos MeOH_H2O
Erzina D7 #37-39 RT: 1.27-1.33 AV: 3 NL: 9.91E7
T: FTMS + p NSI Full ms [150.00-2000.00]
693.0239 z=10
769.9145 z=9
630.0223 z=11
866.0283 z=8
875.0336 z=8
577.6876 z=12
989.4595 z=7
464.2506 z=2
Relative Abundance
m/z

D:\Xcalibur\...\Erzina D7_XT_00001_M_ 2/19/2019 10:30:24 AM

Erzina D7_XT_00001_M_ #1 RT: 1.00 AV: 1 NL: 3.22E7
T: FTMS + p NSI Full ms [150.00-2000.00]

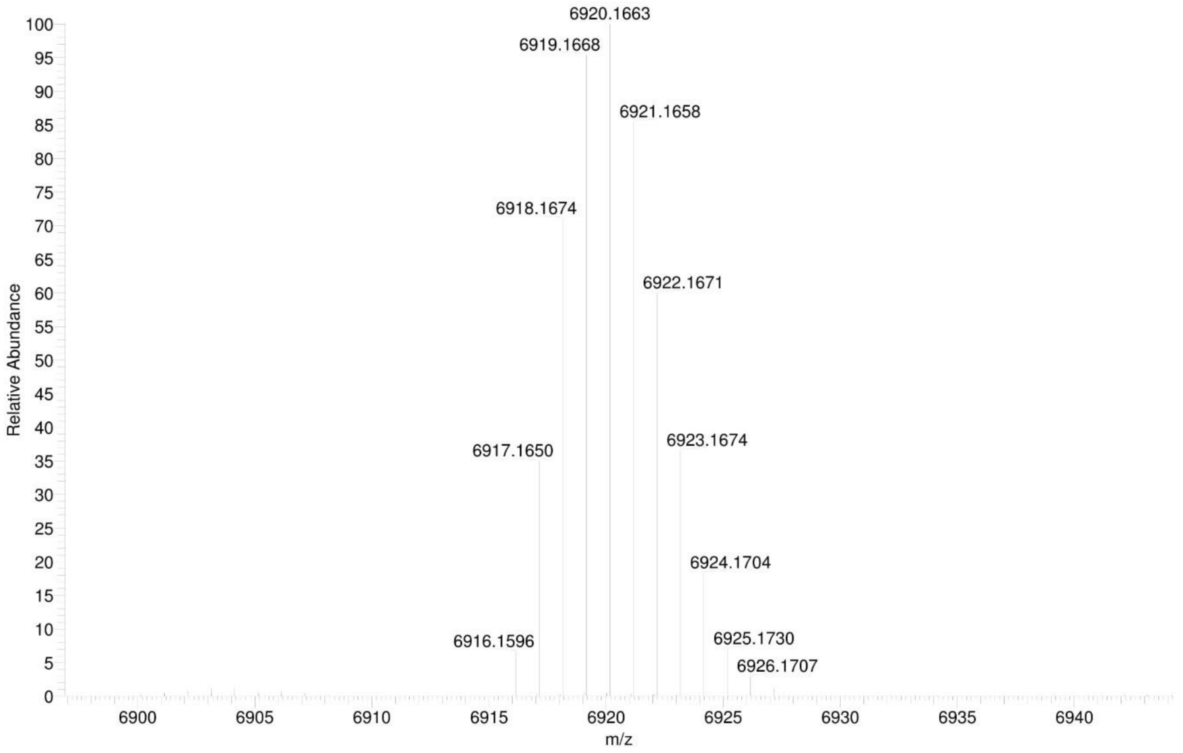

6 ((KA)$_4$(*K*AYE)$_2$*K*K-OH) was obtained from the CEM Liberty Blue synthetiser as foamy colourless solid after preparative RP-HPLC (77.2 mg, 11.3 µmol, 34.8%). Analytical RP-HPLC: t_R=1.02 min (100% A to 100% D in 5 min, λ= 214 nm). HRMS (ESI+): $C_{94}H_{160}N_{26}O_{25}$ calc./obs. 2053.2048/2053.2054 Da [M].

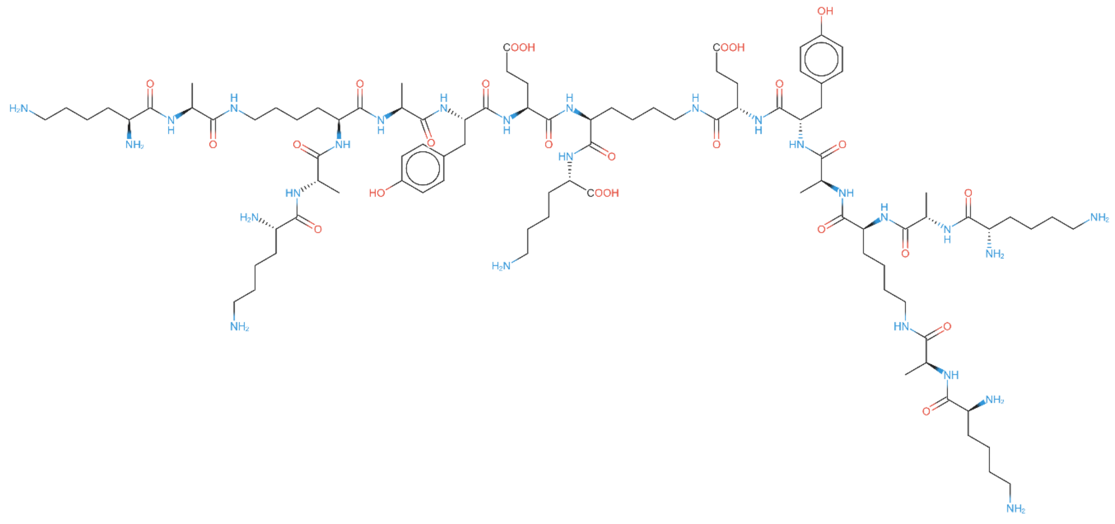

Analytical RP-HPLC:

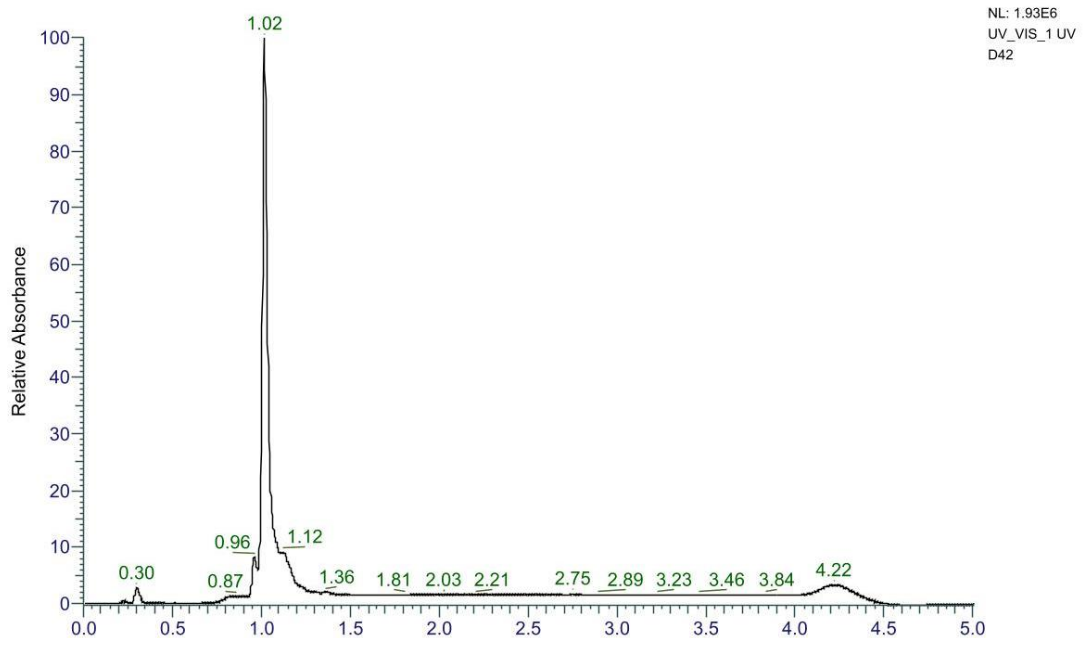

HRMS (NSI+):

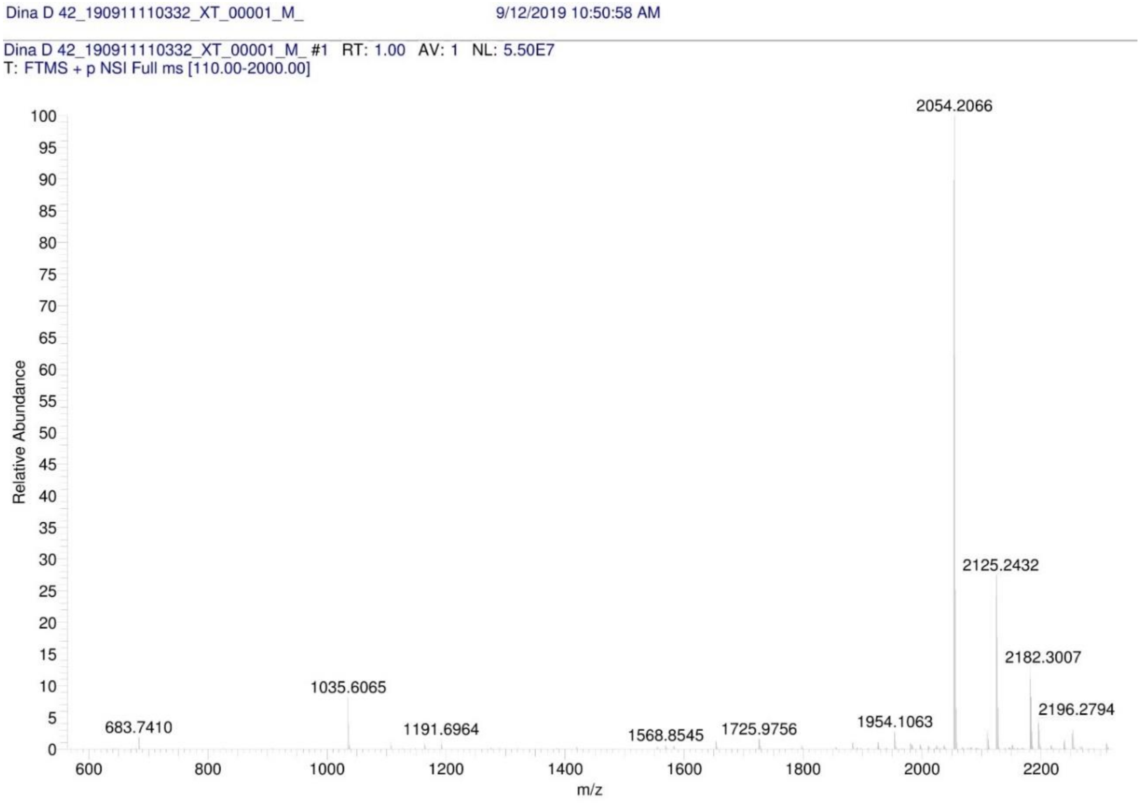

Dina D 42_190911110332_XT_00001_M_
9/12/2019 10:50:58 AM
Dina D 42_190911110332_XT_00001_M_ #1 RT: 1.00 AV: 1 NL: 5.50E7
T: FTMS + p NSI Full ms [110.00-2000.00]
2054.2066
2125.2432
2182.3007
2196.2794
1035.6065
683.7410
1191.6964
1568.8545
1725.9756
1954.1063
Relative Abundance
m/z

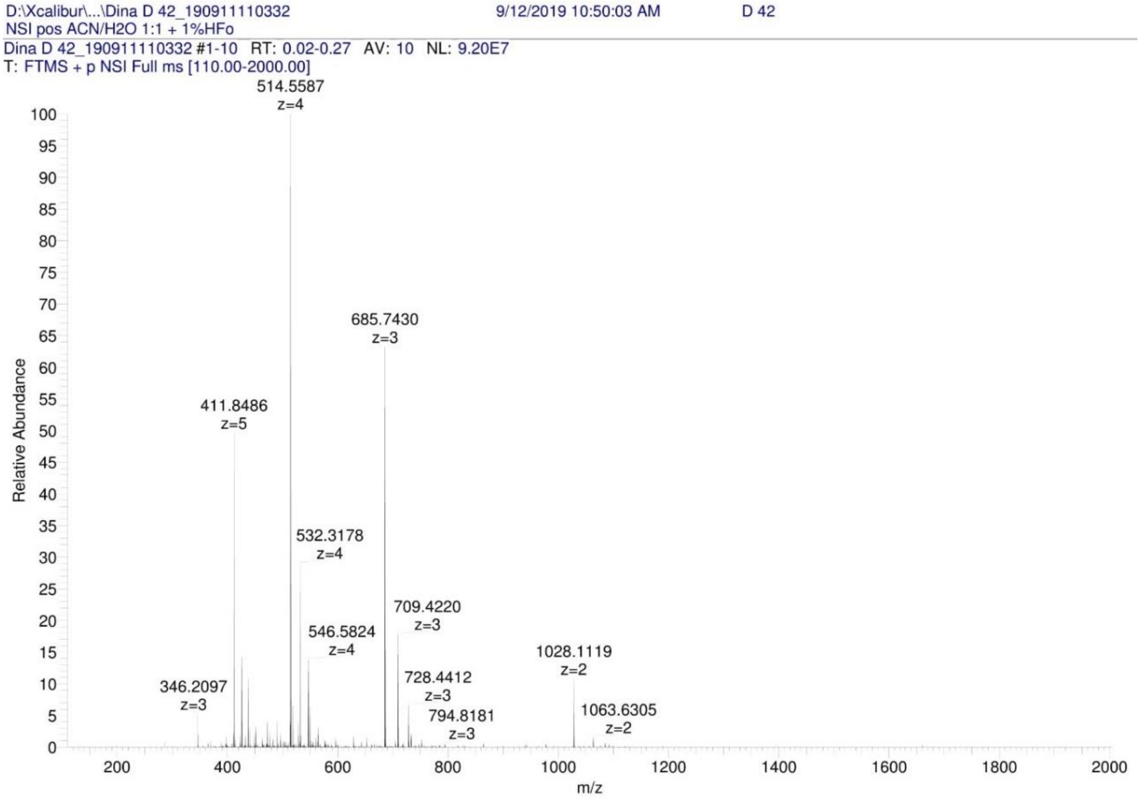

D:\Xcalibur\...\Dina D 42_190911110332
9/12/2019 10:50:03 AM
D 42
NSI pos ACN/H2O 1:1 + 1%HFo
Dina D 42_190911110332 #1-10 RT: 0.02-0.27 AV: 10 NL: 9.20E7
T: FTMS + p NSI Full ms [110.00-2000.00]
514.5587 z=4
685.7430 z=3
411.8486 z=5
532.3178 z=4
709.4220 z=3
546.5824 z=4
1028.1119 z=2
728.4412 z=3
346.2097 z=3
1063.6305 z=2
794.8181 z=3
Relative Abundance
m/z

Dina D 42_190911110332_XT_00001_M_ 9/12/2019 10:50:58 AM

Dina D 42_190911110332_XT_00001_M_ #1 RT: 1.00 AV: 1 NL: 5.50E7
T: FTMS + p NSI Full ms [110.00-2000.00]

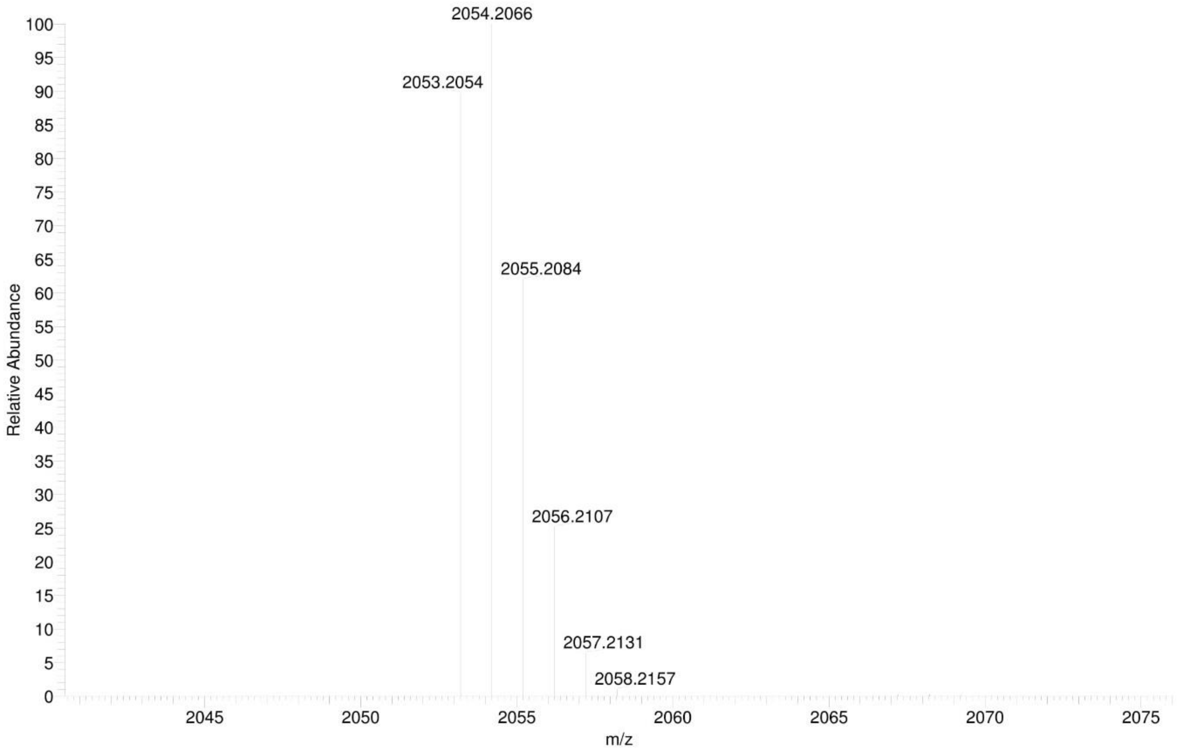

Ac**6 ((AcKA)$_4$(*K*AYE)$_2$*K*K-OH)** was obtained from the CEM Liberty Blue synthetiser as foamy colourless solid after preparative RP-HPLC (23.6 mg, 6.3 µmol, 17.6%). Analytical RP-HPLC: t_R=1.29 min (100% A to 100% D in 5 min, λ= 214 nm). HRMS (ESI+): $C_{102}H_{168}N_{26}O_{29}$ calc./obs. 2221.2471/2221.2492 Da [M].

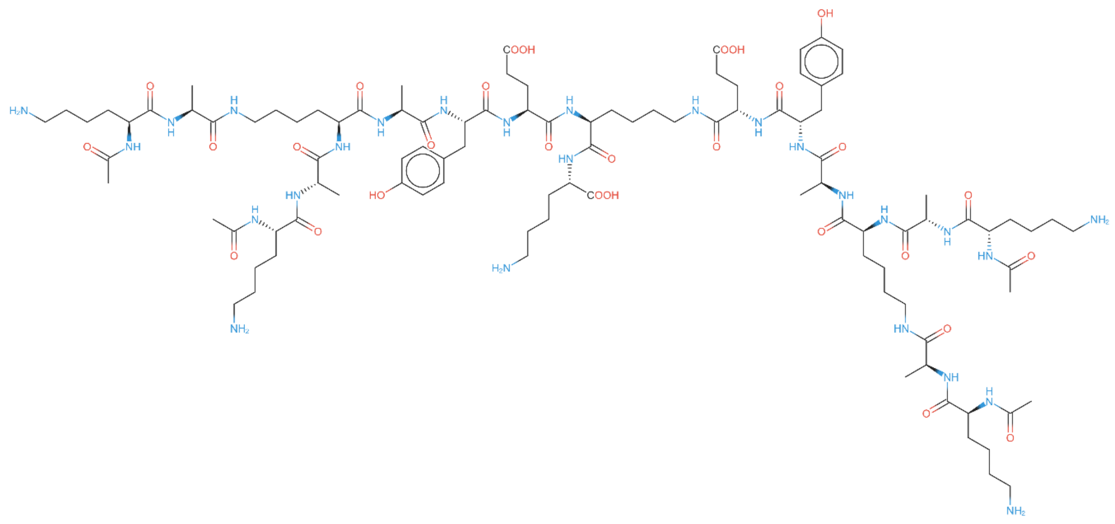

Analytical RP-HPLC:

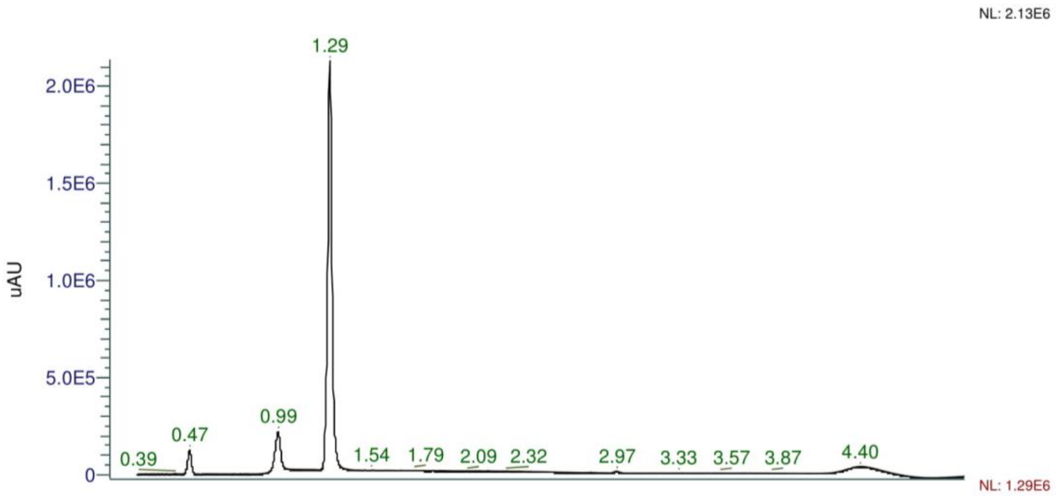

HRMS (NSI+):

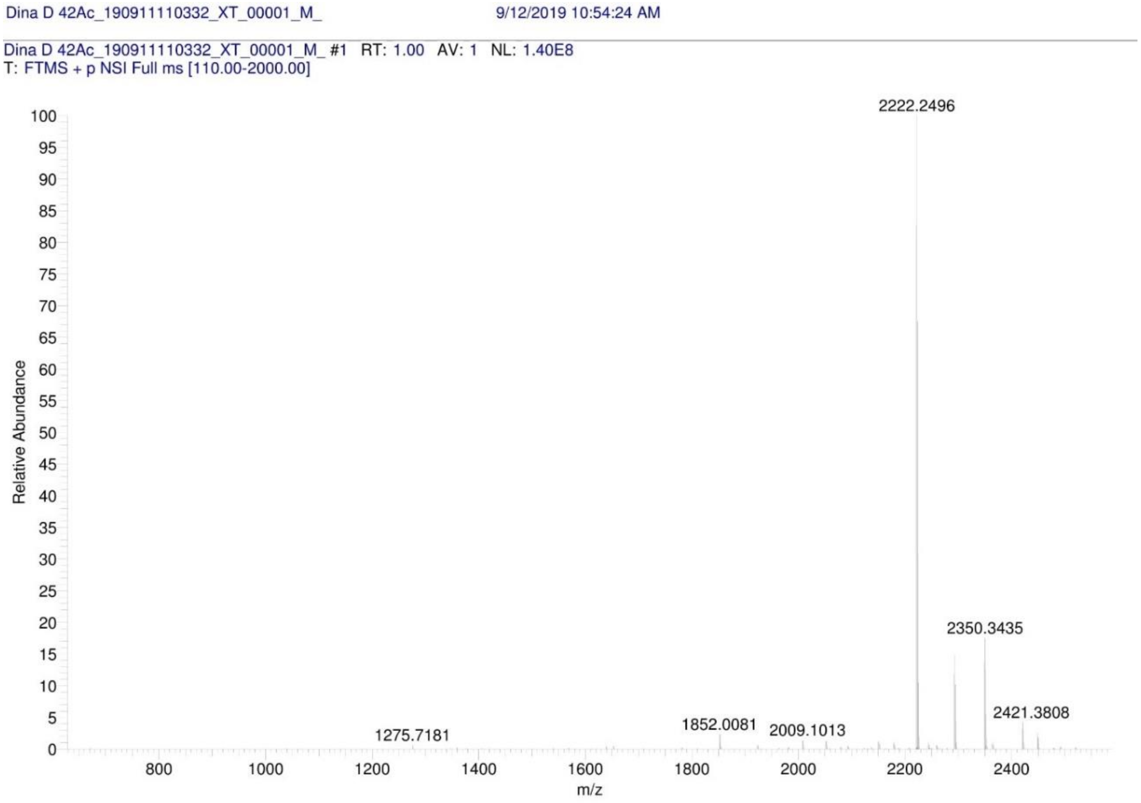
Dina D 42Ac_190911110332_XT_00001_M_
9/12/2019 10:54:24 AM
Dina D 42Ac_190911110332_XT_00001_M_ #1 RT: 1.00 AV: 1 NL: 1.40E8
T: FTMS + p NSI Full ms [110.00-2000.00]
2222.2496
2350.3435
2421.3808
1852.0081
2009.1013
1275.7181
Relative Abundance
m/z

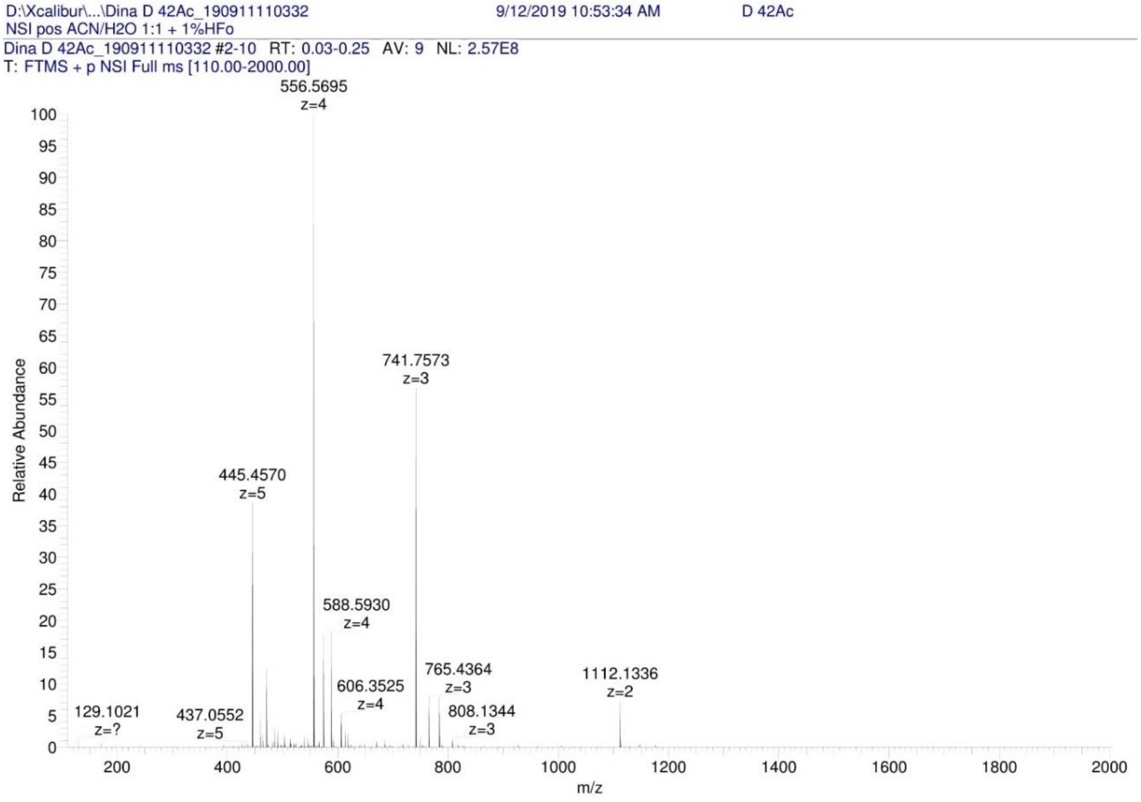
D:\Xcalibur\...\Dina D 42Ac_190911110332
9/12/2019 10:53:34 AM
D 42Ac
NSI pos ACN/H2O 1:1 + 1%HFo
Dina D 42Ac_190911110332 #2-10 RT: 0.03-0.25 AV: 9 NL: 2.57E8
T: FTMS + p NSI Full ms [110.00-2000.00]
556.5695 z=4
741.7573 z=3
445.4570 z=5
588.5930 z=4
765.4364 z=3
606.3525 z=4
808.1344 z=3
1112.1336 z=2
129.1021 z=?
437.0552 z=5
Relative Abundance
m/z

Dina D 42Ac_190911110332_XT_00001_M_ 9/12/2019 10:54:24 AM

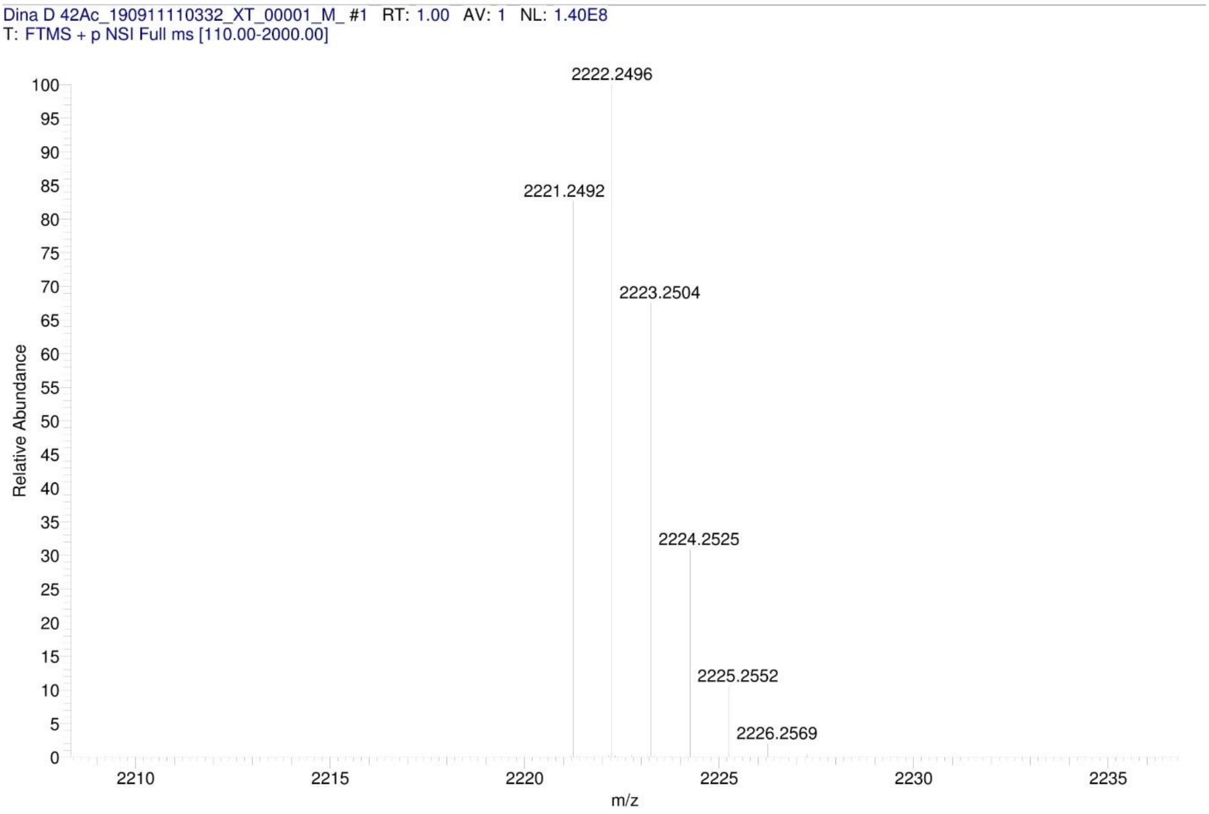

ClAc**6** **((ClAcKA)$_4$(*K*AYE)$_2$*K*K-OH)** was obtained from the CEM Liberty Blue synthetiser as foamy colourless solid after preparative RP-HPLC (106.0 mg, 13.7 µmol, 40.2%). Analytical RP-HPLC: t_R=1.37 min (100% A to 100% D in 5 min, λ= 214 nm). HRMS (ESI+): $C_{102}H_{164}Cl_4N_{26}O_{29}$ calc./obs. 2357.0912/2358.0922 Da [M].

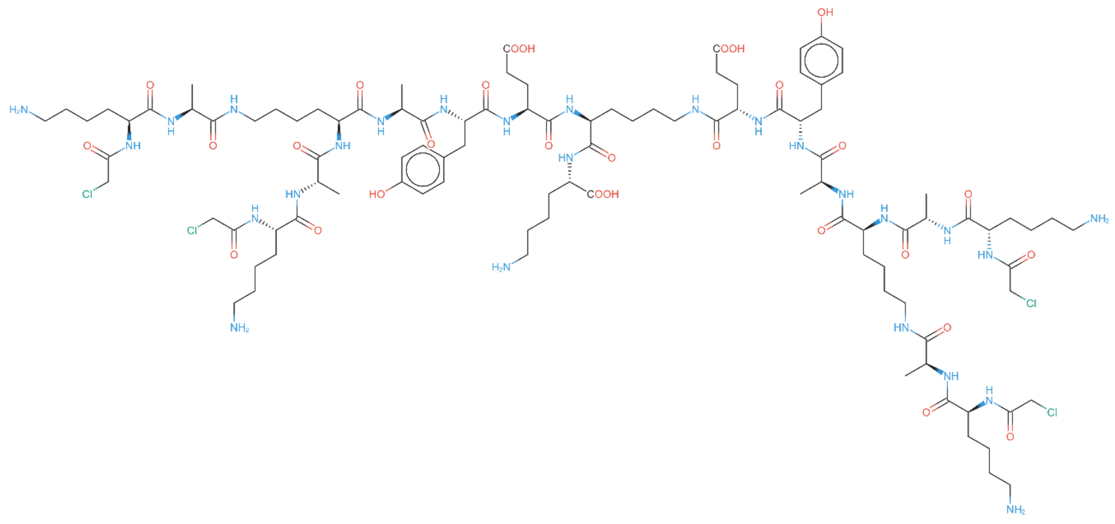

Analytical RP-HPLC:

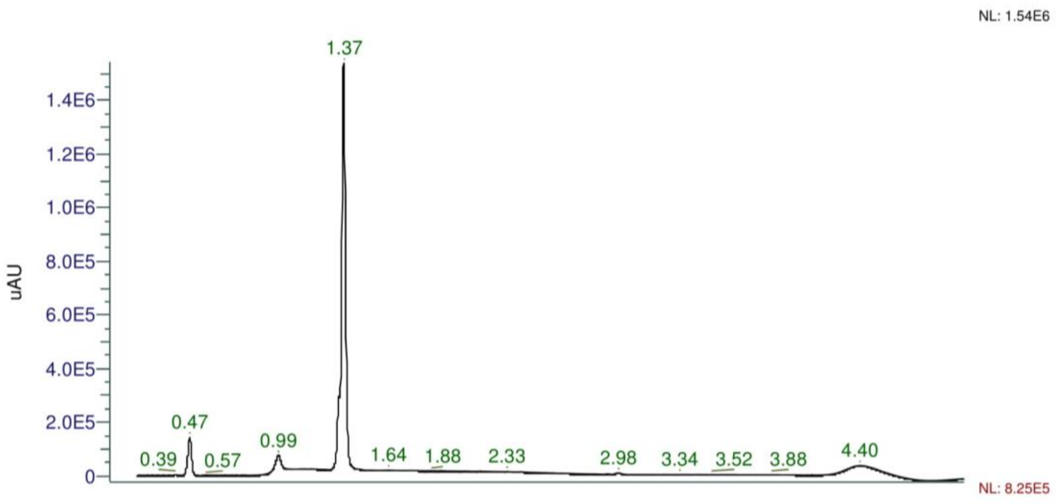

HRMS (NSI+):

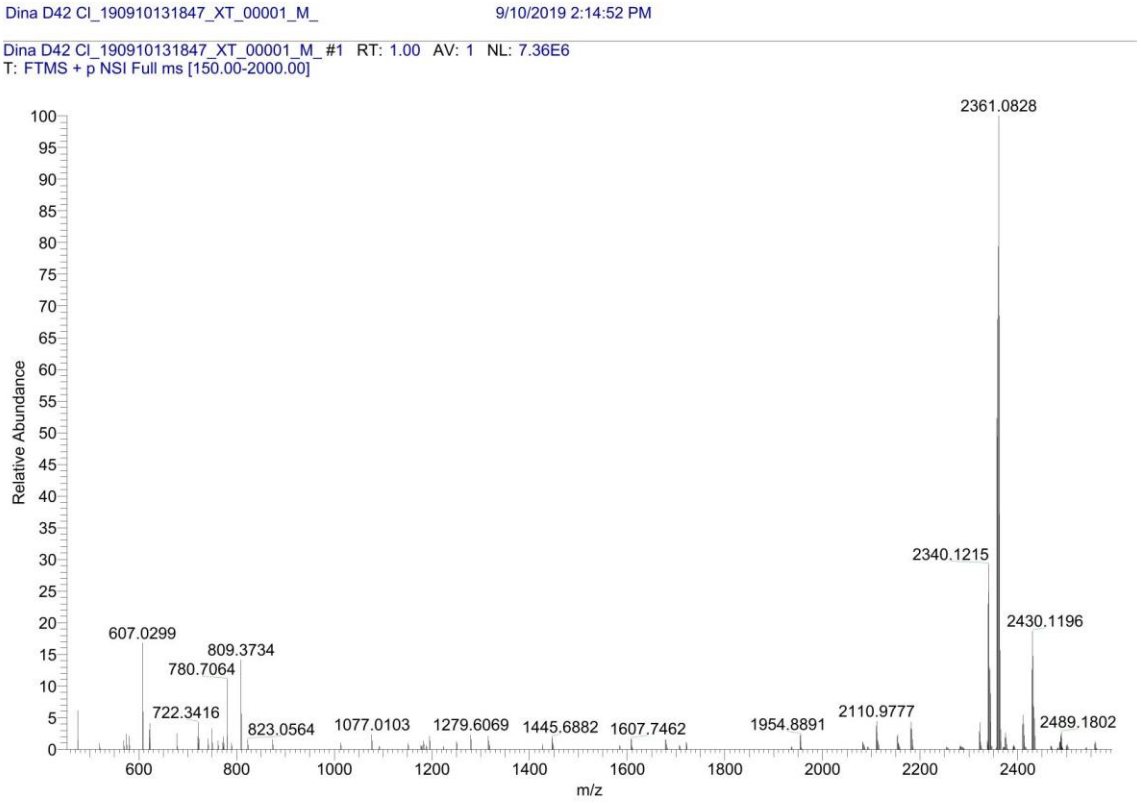
Dina D42 Cl_190910131847_XT_00001_M_
9/10/2019 2:14:52 PM
Dina D42 Cl_190910131847_XT_00001_M_ #1 RT: 1.00 AV: 1 NL: 7.36E6
T: FTMS + p NSI Full ms [150.00-2000.00]
2361.0828
2340.1215
2430.1196
607.0299
809.3734
780.7064
722.3416
823.0564
1077.0103
1279.6069
1445.6882
1607.7462
1954.8891
2110.9777
2489.1802
Relative Abundance
m/z

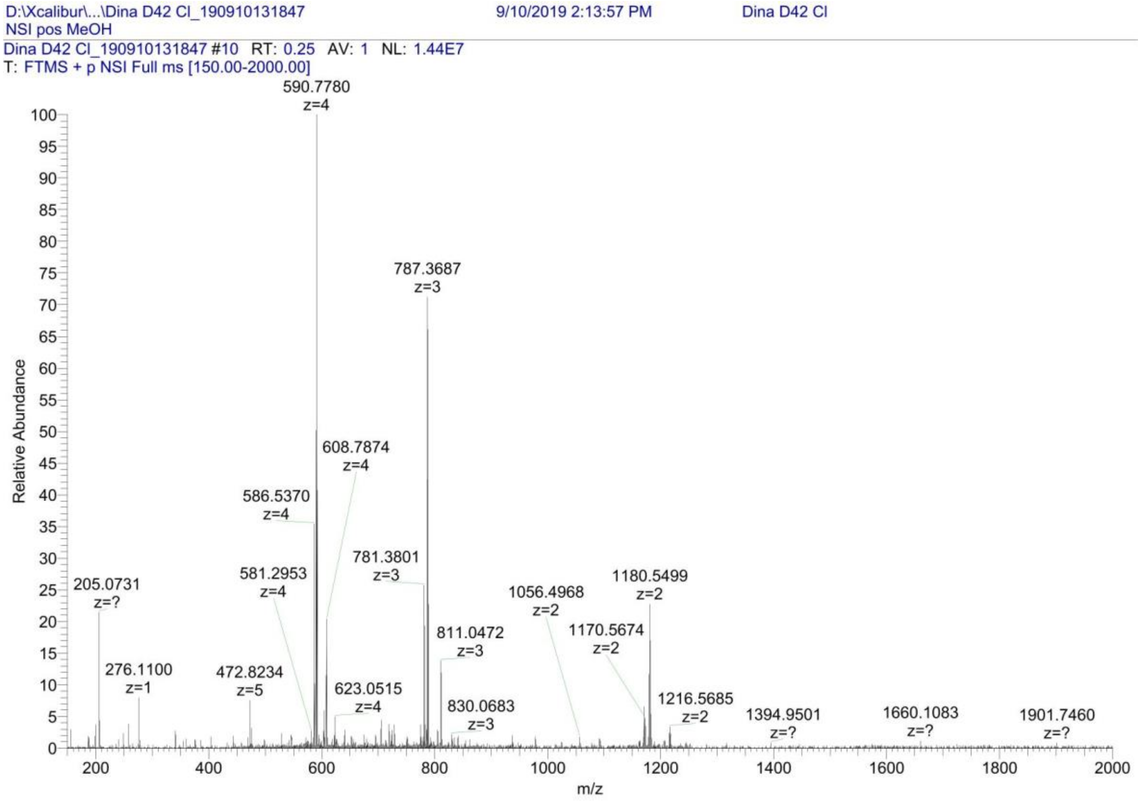
D:\Xcalibur\...\Dina D42 Cl_190910131847
9/10/2019 2:13:57 PM
Dina D42 Cl
NSI pos MeOH
Dina D42 Cl_190910131847 #10 RT: 0.25 AV: 1 NL: 1.44E7
T: FTMS + p NSI Full ms [150.00-2000.00]
590.7780 z=4
787.3687 z=3
608.7874 z=4
586.5370 z=4
781.3801 z=3
581.2953 z=4
205.0731 z=?
1180.5499 z=2
1056.4968 z=2
811.0472 z=3
1170.5674 z=2
276.1100 z=1
472.8234 z=5
623.0515 z=4
1216.5685 z=2
830.0683 z=3
1394.9501 z=?
1660.1083 z=?
1901.7460 z=?
Relative Abundance
m/z

Erzina D42 Cl_181017084418_XT_00001_M_ 10/19/2018 10:22:21 AM

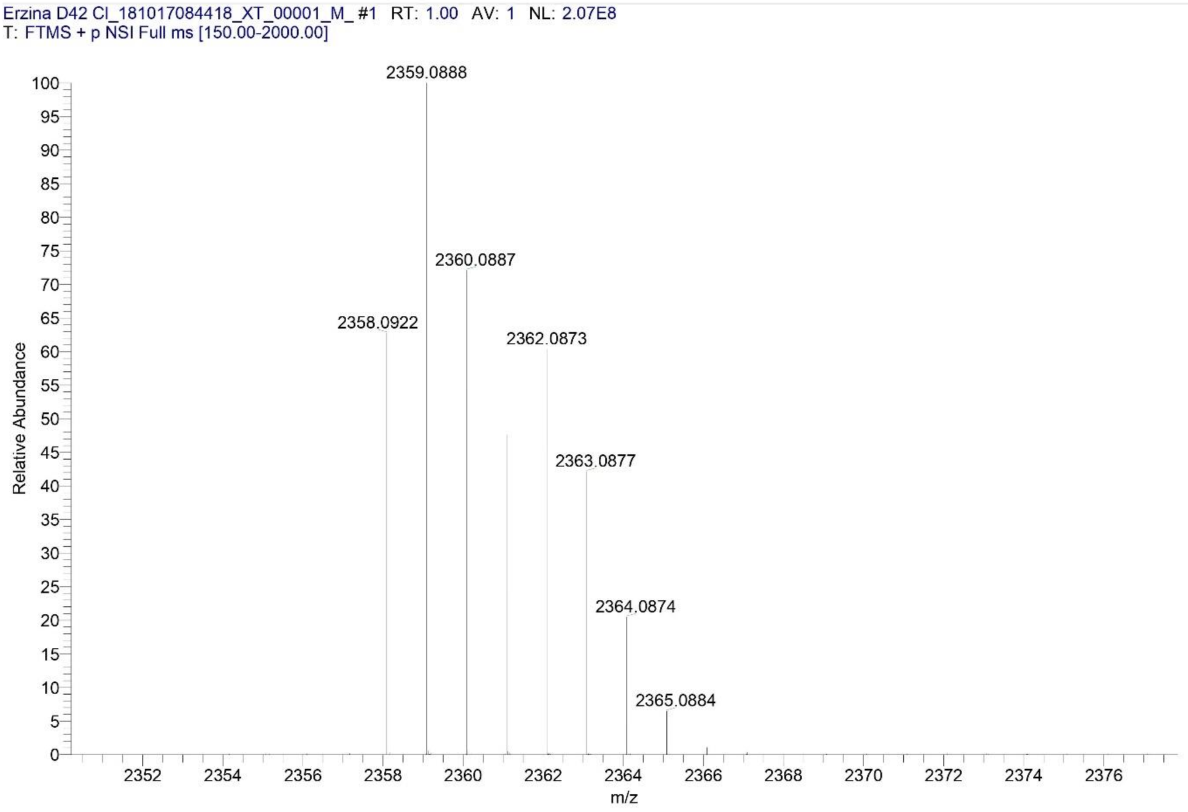

7 ((KA)$_4$(*K*EYA)$_2$*K*KAK-OH) was obtained from the CEM Liberty Blue synthetiser as foamy colourless solid after preparative RP-HPLC (25.8 mg, 6.0 µmol, 20.3%). Analytical RP-HPLC: t_R=1.19 min (100% A to 100% D in 5 min, λ= 214 nm). HRMS (ESI+): $C_{103}H_{177}N_{29}O_{27}$ calc./obs. 2252.3369/2252.3399 Da [M].

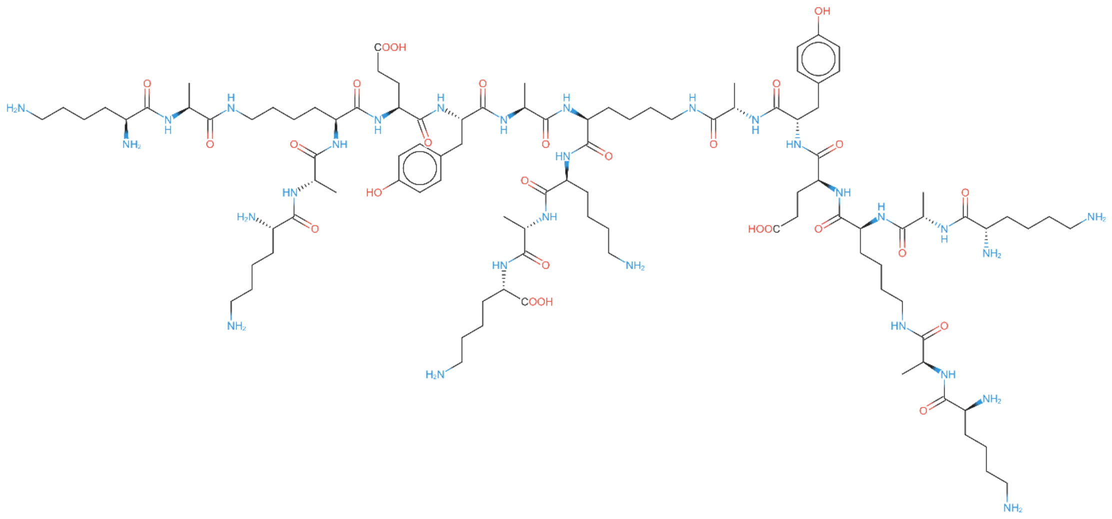

Analytical RP-HPLC:

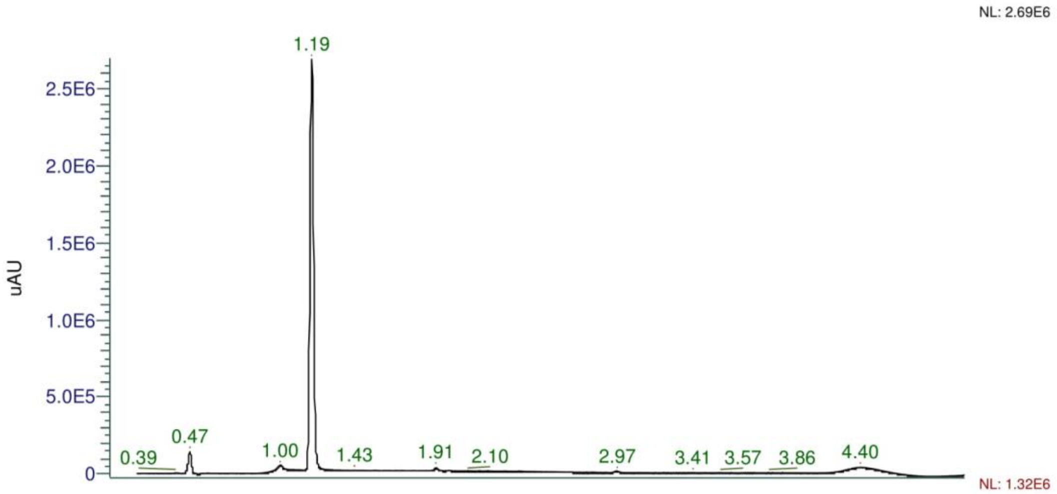

HRMS (NSI+):

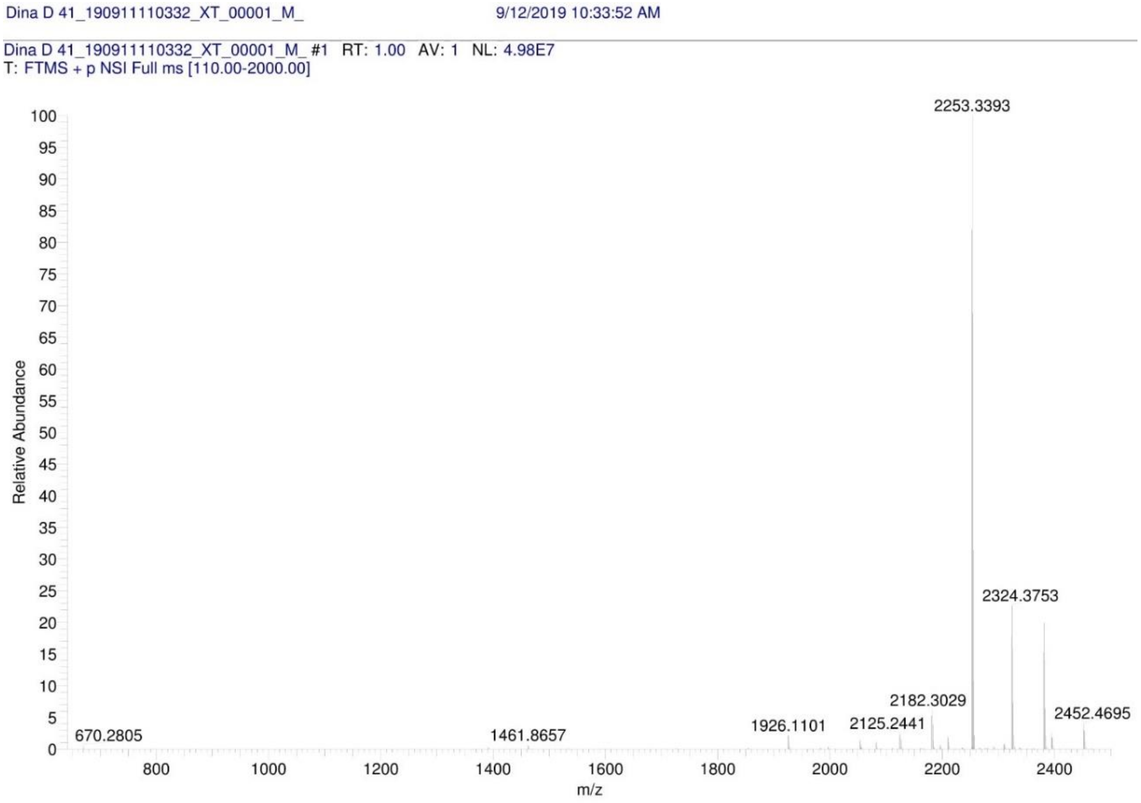

Dina D 41_190911110332_XT_00001_M_
9/12/2019 10:33:52 AM
Dina D 41_190911110332_XT_00001_M_ #1 RT: 1.00 AV: 1 NL: 4.98E7
T: FTMS + p NSI Full ms [110.00-2000.00]
2253.3393
2324.3753
2182.3029
2452.4695
1926.1101
2125.2441
670.2805
1461.8657
Relative Abundance
m/z

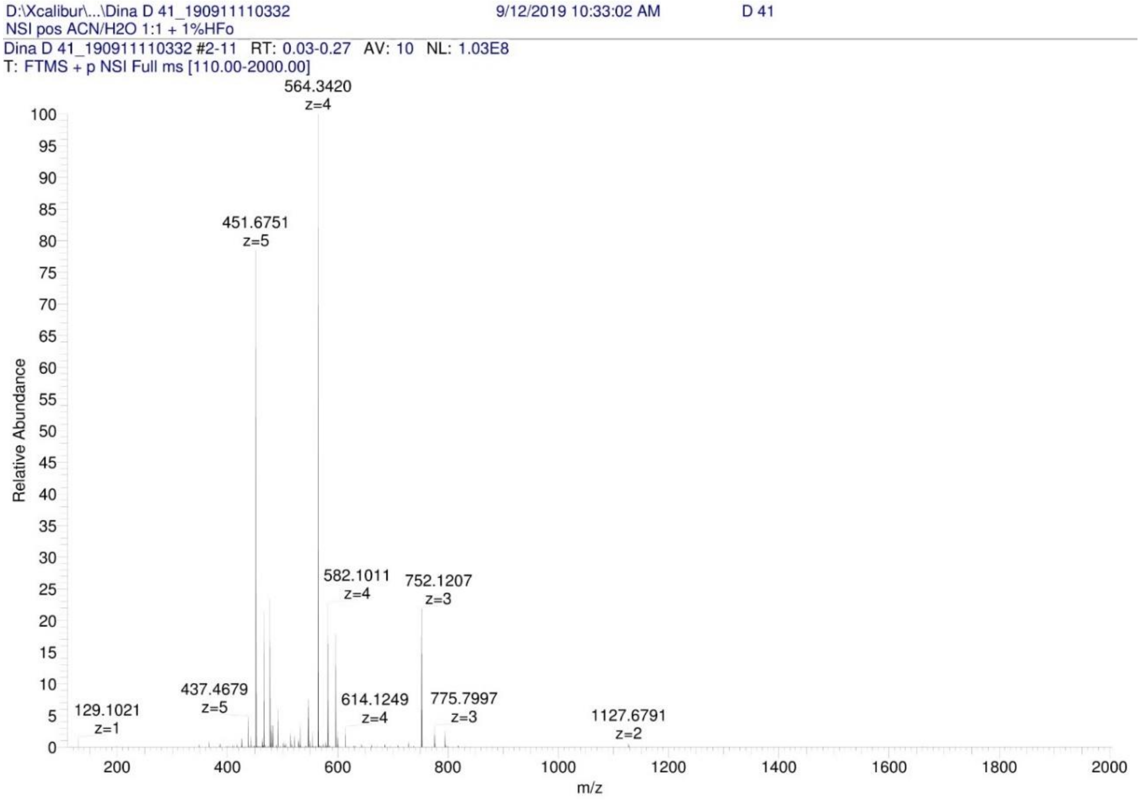

D:\Xcalibur\...\Dina D 41_190911110332
9/12/2019 10:33:02 AM
D 41
NSI pos ACN/H2O 1:1 + 1%HFo
Dina D 41_190911110332 #2-11 RT: 0.03-0.27 AV: 10 NL: 1.03E8
T: FTMS + p NSI Full ms [110.00-2000.00]
564.3420 z=4
451.6751 z=5
582.1011 z=4
752.1207 z=3
437.4679 z=5
614.1249 z=4
775.7997 z=3
129.1021 z=1
1127.6791 z=2
Relative Abundance
m/z

Dina D 41_190911110332_XT_00001_M_ 9/12/2019 10:33:52 AM

Dina D 41_190911110332_XT_00001_M_ #1 RT: 1.00 AV: 1 NL: 4.98E7
T: FTMS + p NSI Full ms [110.00-2000.00]

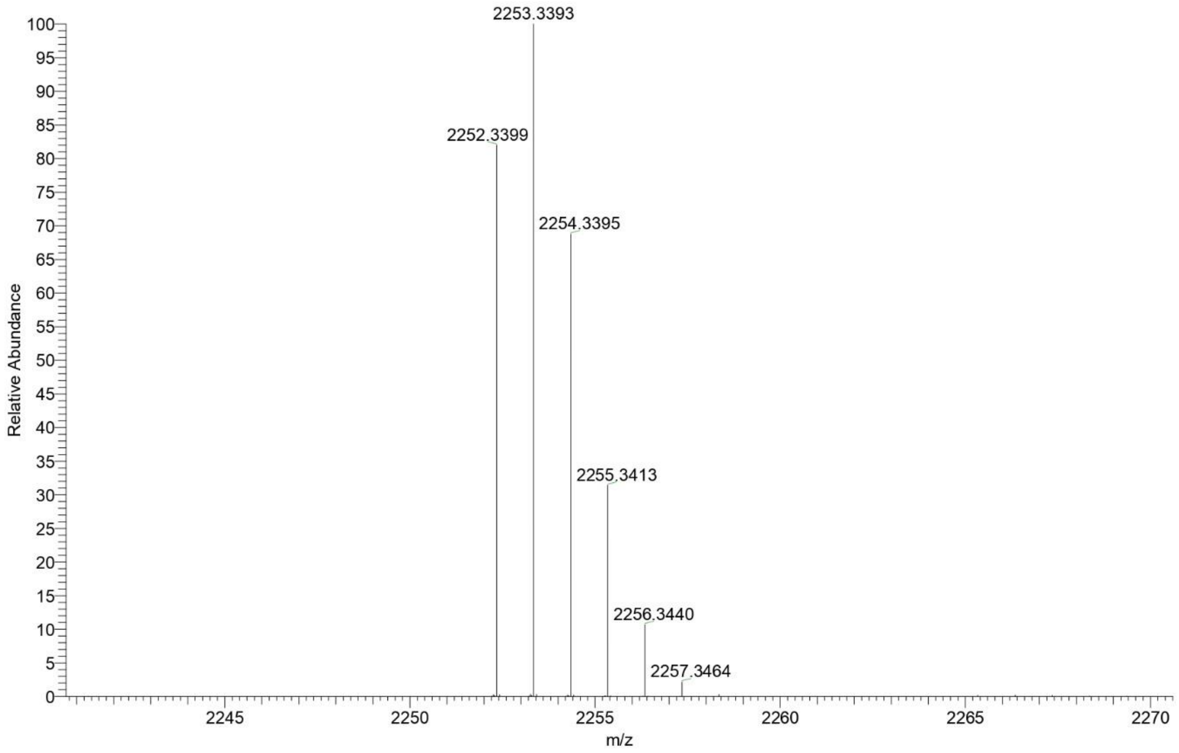

Ac**7 ((AcKA)$_4$(*K*EYA)$_2$*K*KAK-OH)** was obtained from the CEM Liberty Blue synthetiser as foamy colourless solid after preparative RP-HPLC (10.8 mg, 3.1 µmol, 9.7%). Analytical RP-HPLC: t_R=1.13 min (100% A to 100% D in 5 min, λ= 214 nm). HRMS (ESI+): $C_{111}H_{185}N_{29}O_{31}$ calc./obs. 2420.3791/2420.3812 Da [M].

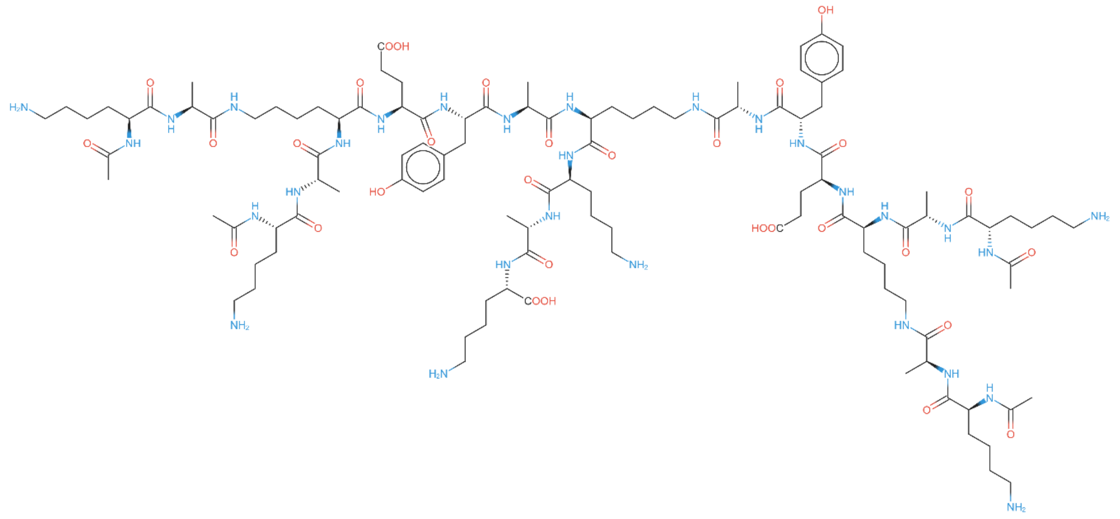

Analytical RP-HPLC:

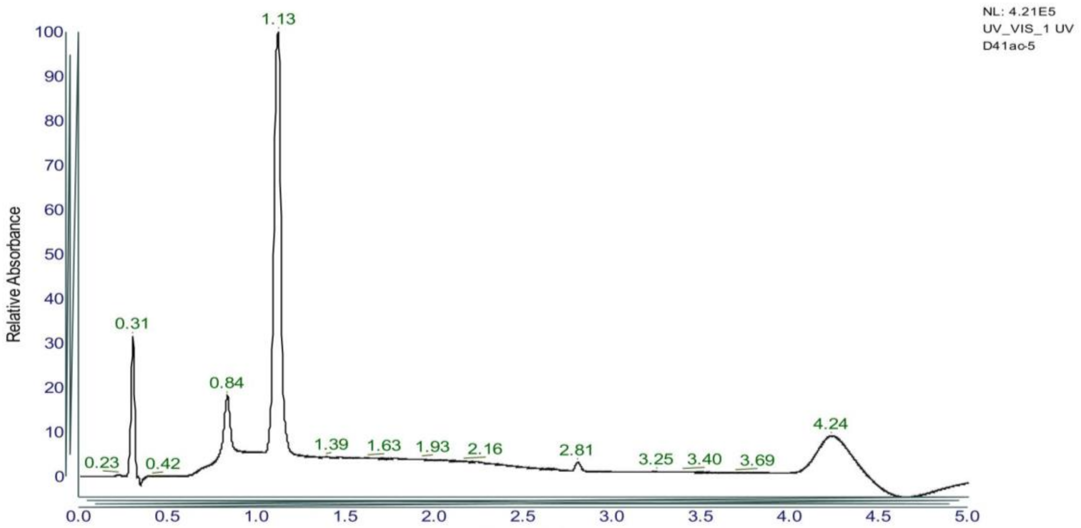

HRMS (NSI+):

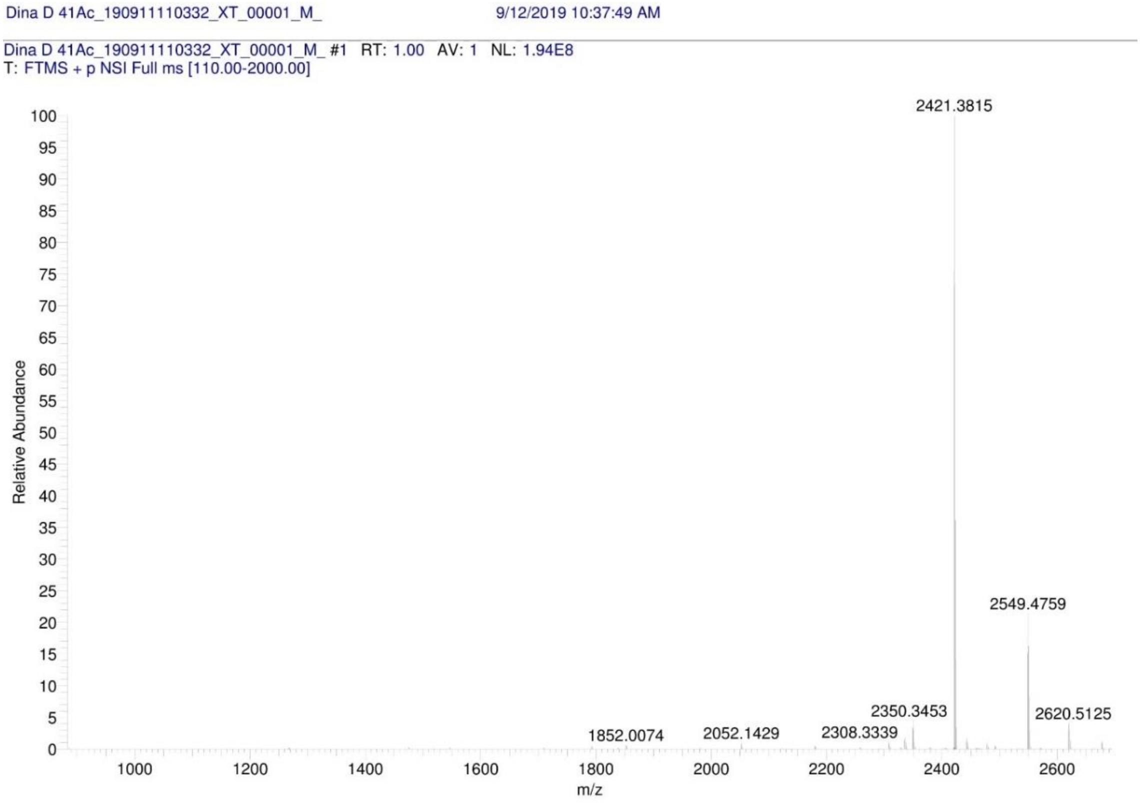
Dina D 41Ac_190911110332_XT_00001_M_
9/12/2019 10:37:49 AM
Dina D 41Ac_190911110332_XT_00001_M_ #1 RT: 1.00 AV: 1 NL: 1.94E8
T: FTMS + p NSI Full ms [110.00-2000.00]
2421.3815
2549.4759
2350.3453
2620.5125
1852.0074
2052.1429
2308.3339
Relative Abundance
m/z

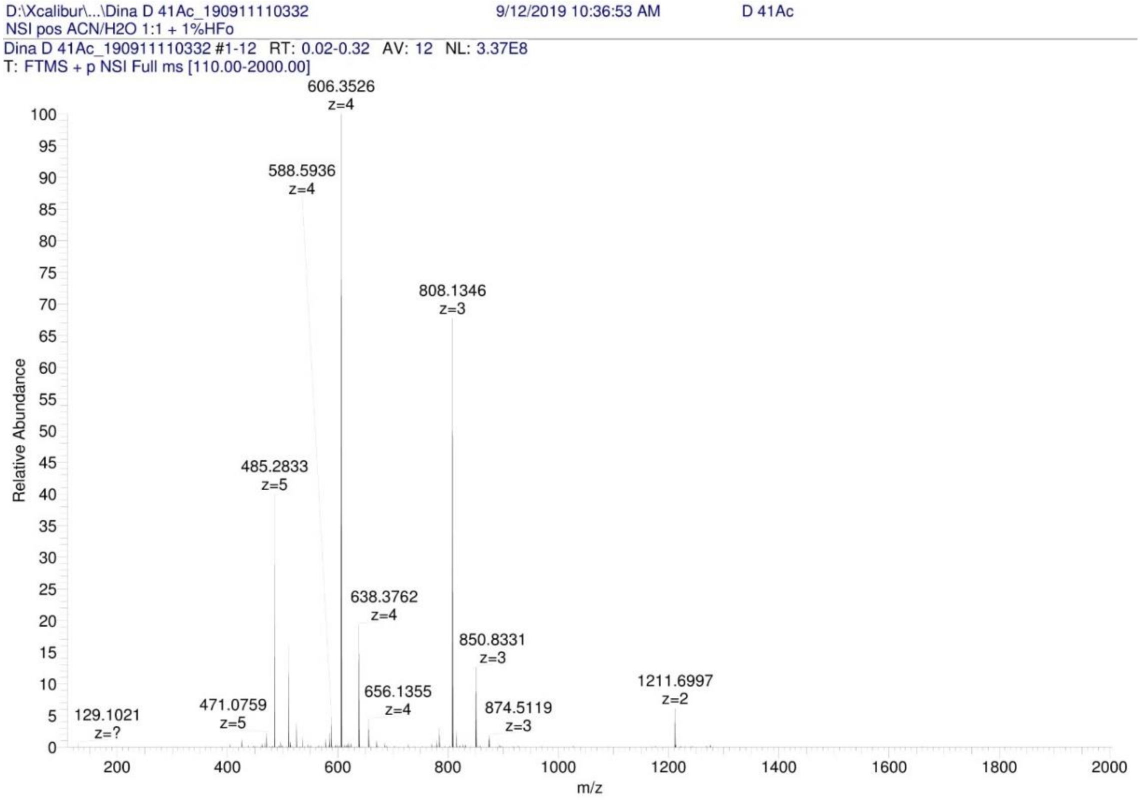
D:\Xcalibur\...\Dina D 41Ac_190911110332
9/12/2019 10:36:53 AM
D 41Ac
NSI pos ACN/H2O 1:1 + 1%HFo
Dina D 41Ac_190911110332 #1-12 RT: 0.02-0.32 AV: 12 NL: 3.37E8
T: FTMS + p NSI Full ms [110.00-2000.00]
606.3526 z=4
588.5936 z=4
808.1346 z=3
485.2833 z=5
638.3762 z=4
850.8331 z=3
1211.6997 z=2
656.1355 z=4
874.5119 z=3
471.0759 z=5
129.1021 z=?
Relative Abundance
m/z

Dina D 41Ac_190911110332_XT_00001_M_ 9/12/2019 10:37:49 AM

Dina D 41Ac_190911110332_XT_00001_M_ #1 RT: 1.00 AV: 1 NL: 1.94E8
T: FTMS + p NSI Full ms [110.00-2000.00]

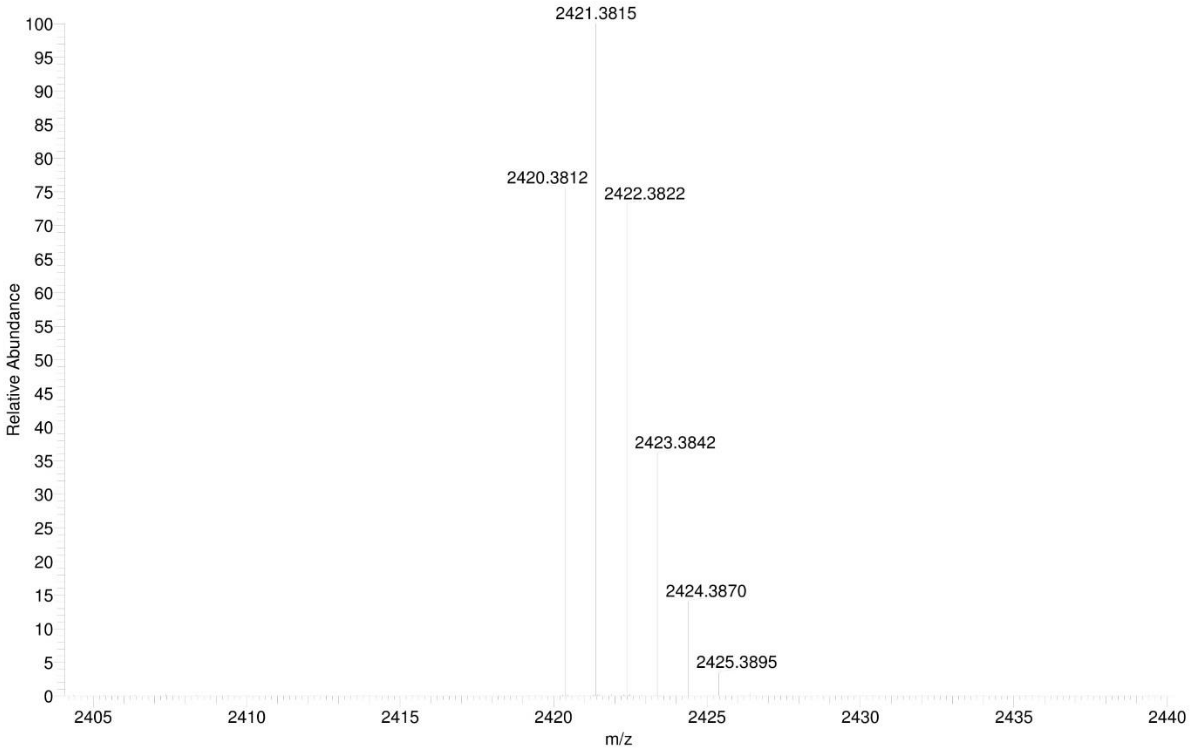

ClAc**7** **((ClAcKA)$_4$(*K*EYA)$_2$*K*KAK-OH)** was obtained from the CEM Liberty Blue synthetiser as foamy colourless solid after preparative RP-HPLC (9.3 mg, 2.5 µmol, 8.0%). Analytical RP-HPLC: t_R=1.16 min (100% A to 100% D in 5 min, λ= 214 nm). HRMS (ESI+): $C_{111}H_{181}Cl_4N_{29}O_{31}$ calc./obs. 2556.2232/2556.2228 Da [M].

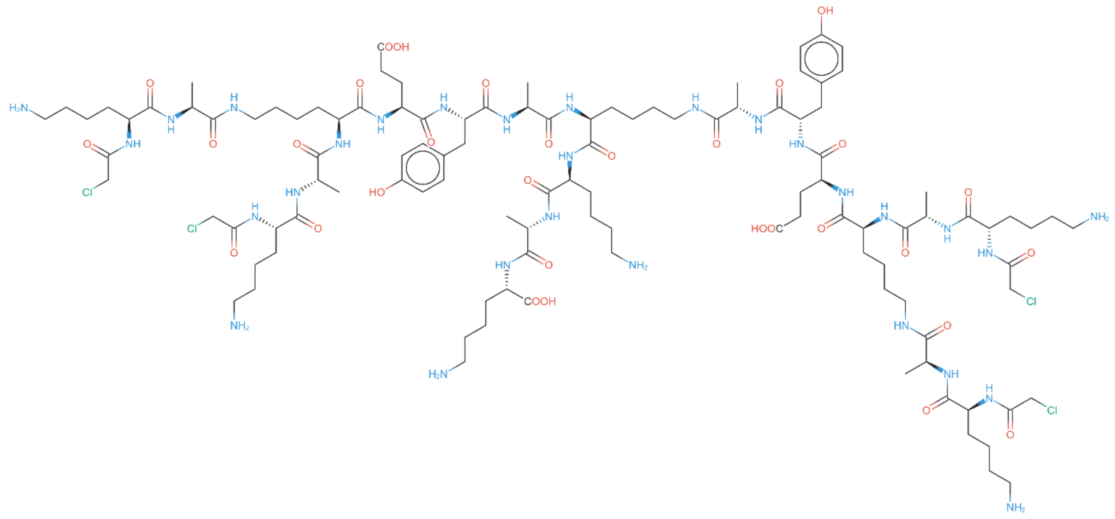

Analytical RP-HPLC:

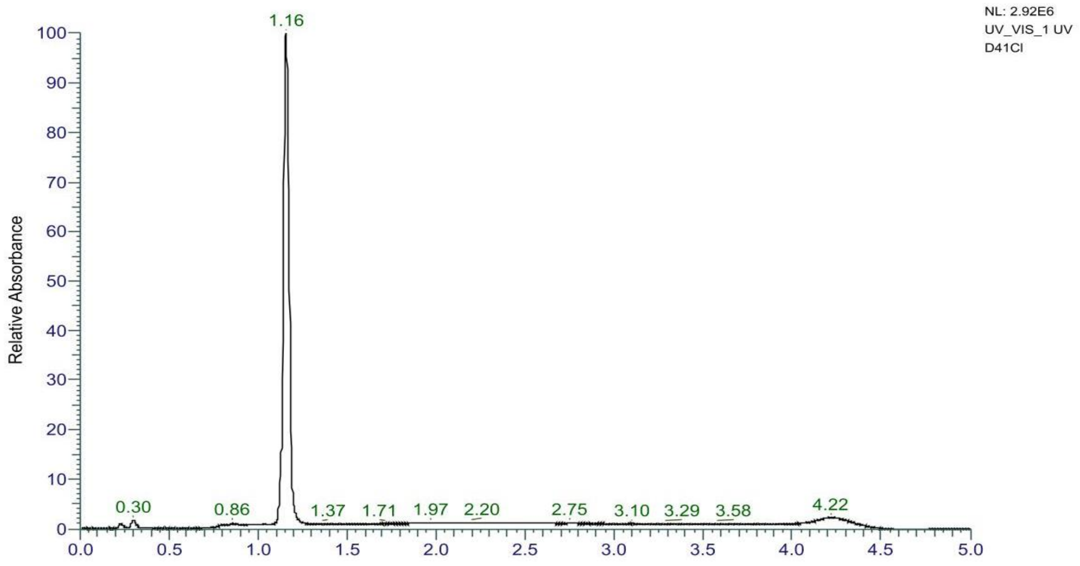

HRMS (NSI+):

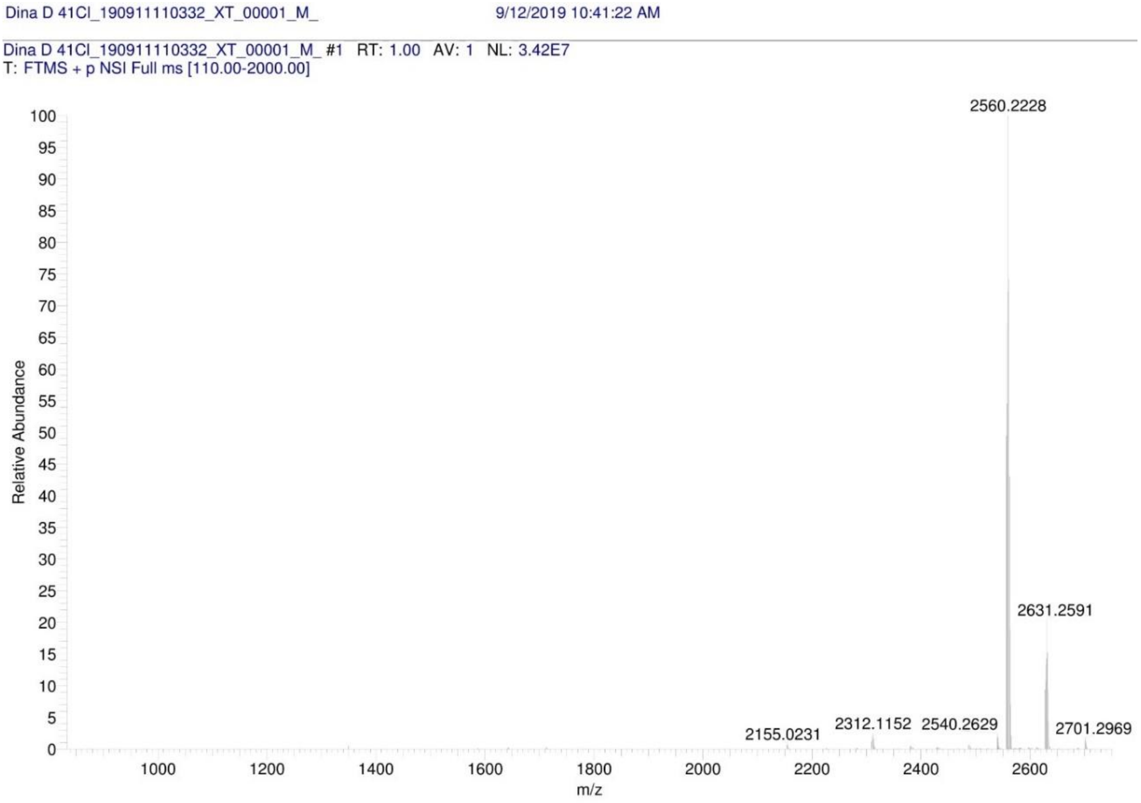

Dina D 41Cl_190911110332_XT_00001_M_
9/12/2019 10:41:22 AM
Dina D 41Cl_190911110332_XT_00001_M_ #1 RT: 1.00 AV: 1 NL: 3.42E7
T: FTMS + p NSI Full ms [110.00-2000.00]
2560.2228
2631.2591
2155.0231
2312.1152
2540.2629
2701.2969
Relative Abundance
m/z

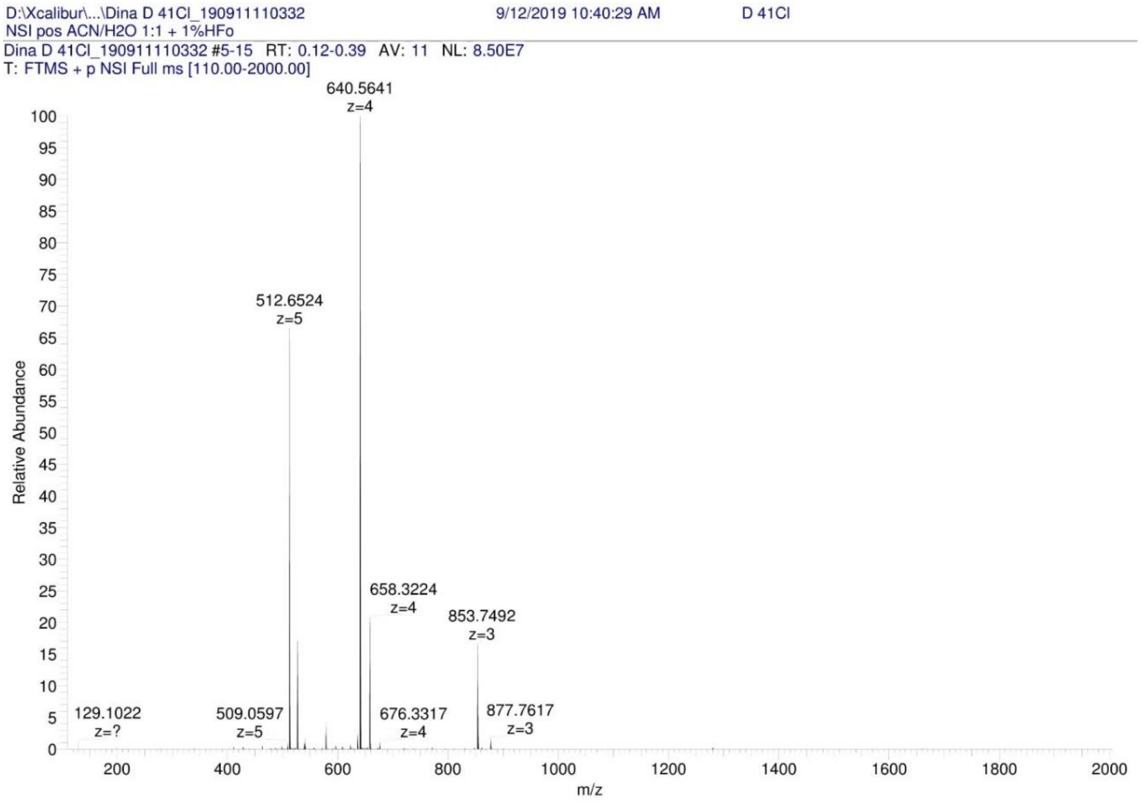

D:\Xcalibur\...\Dina D 41Cl_190911110332
9/12/2019 10:40:29 AM
D 41Cl
NSI pos ACN/H2O 1:1 + 1%HFo
Dina D 41Cl_190911110332 #5-15 RT: 0.12-0.39 AV: 11 NL: 8.50E7
T: FTMS + p NSI Full ms [110.00-2000.00]
640.5641 z=4
512.6524 z=5
658.3224 z=4
853.7492 z=3
129.1022 z=?
509.0597 z=5
676.3317 z=4
877.7617 z=3
Relative Abundance
m/z

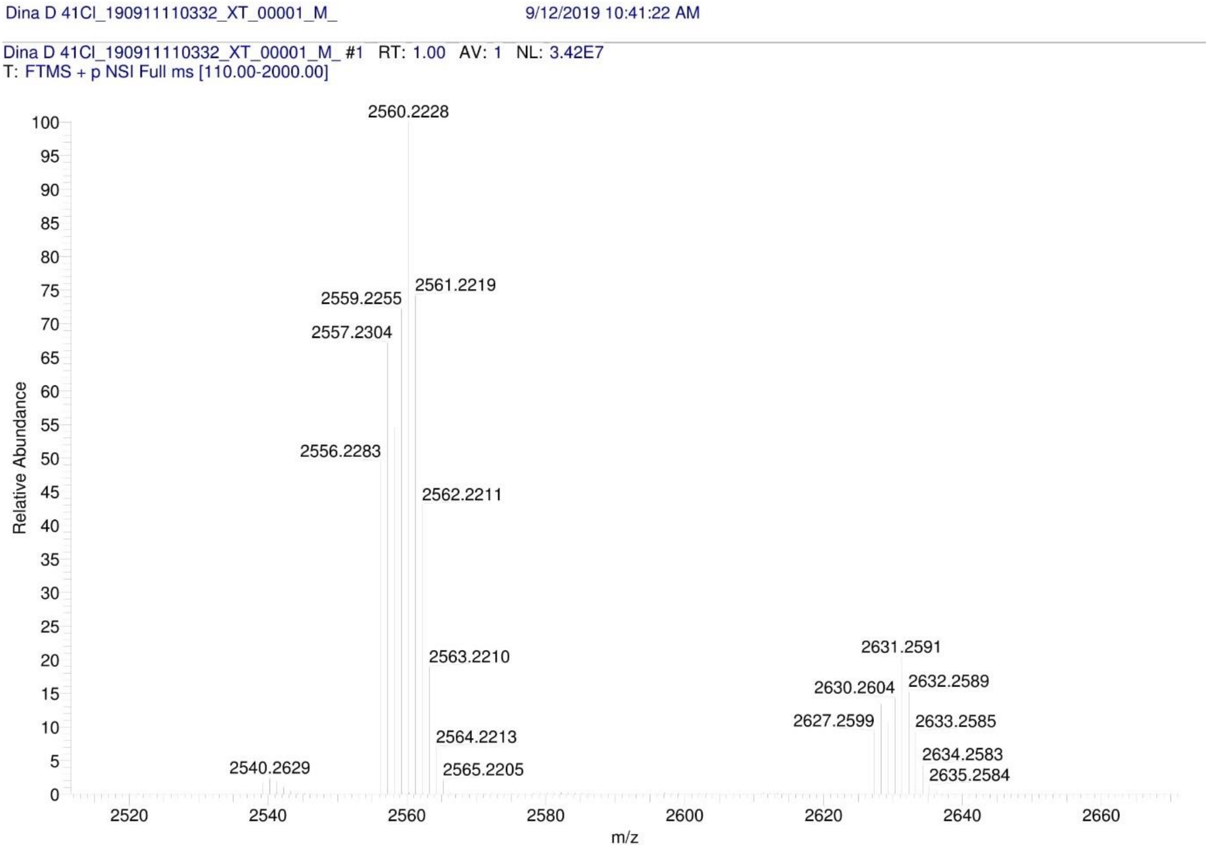
Dina D 41Cl_190911110332_XT_00001_M_
9/12/2019 10:41:22 AM
Dina D 41Cl_190911110332_XT_00001_M_ #1 RT: 1.00 AV: 1 NL: 3.42E7
T: FTMS + p NSI Full ms [110.00-2000.00]
2560.2228
2561.2219
2559.2255
2557.2304
2556.2283
2562.2211
2563.2210
2564.2213
2565.2205
2540.2629
2631.2591
2630.2604
2632.2589
2627.2599
2633.2585
2634.2583
2635.2584
Relative Abundance
m/z

8 ((AK)$_4$(*K*KYE)$_2$*K*AAA-OH) was obtained from the CEM Liberty Blue synthetiser as foamy colourless solid after preparative RP-HPLC (12.2 mg, 2.8 µmol, 9.6%). Analytical RP-HPLC: t_R=1.20 min (100% A to 100% D in 5 min, λ= 214 nm). HRMS (ESI+): $C_{103}H_{177}N_{29}O_{27}$ calc./obs. 2252.3369/2252.3332 Da [M].

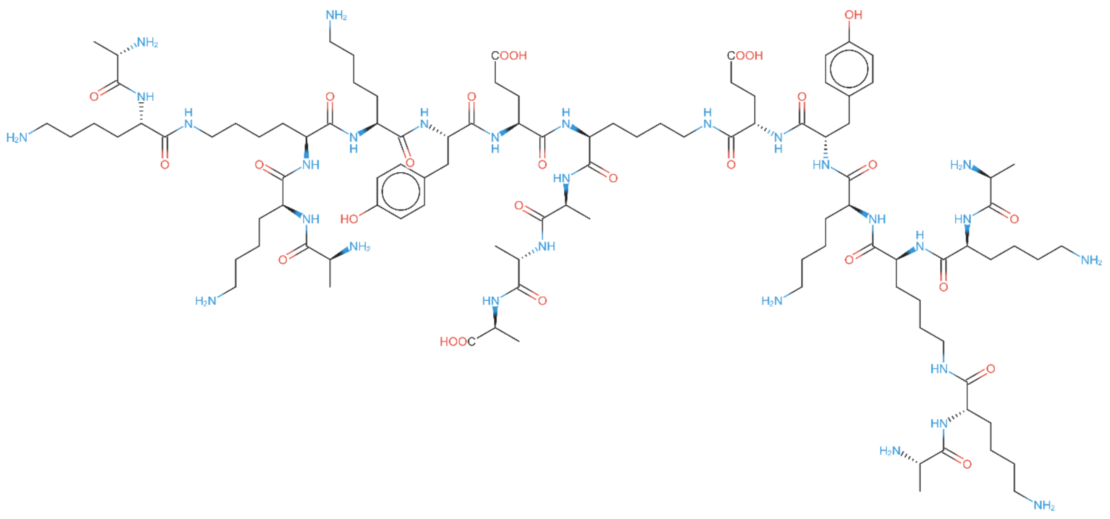

Analytical RP-HPLC:

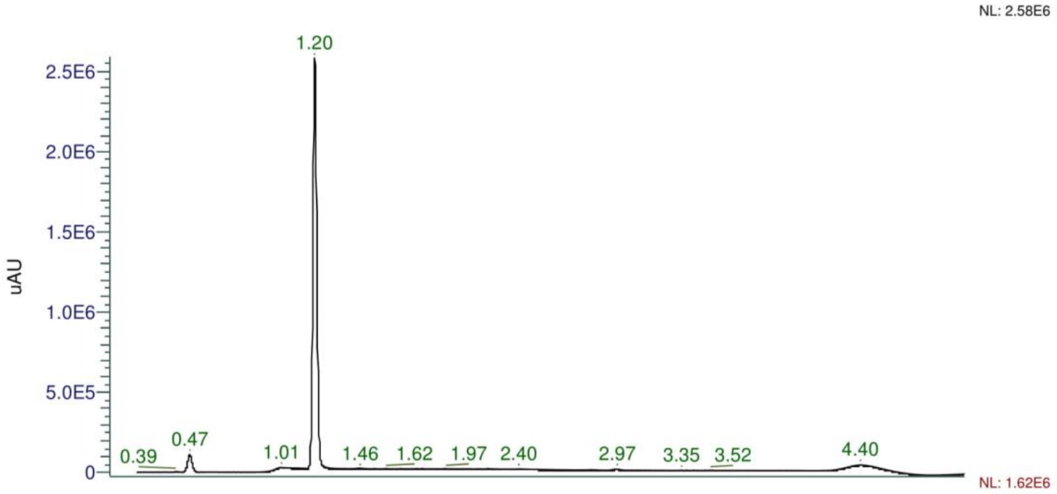

HRMS (NSI+):

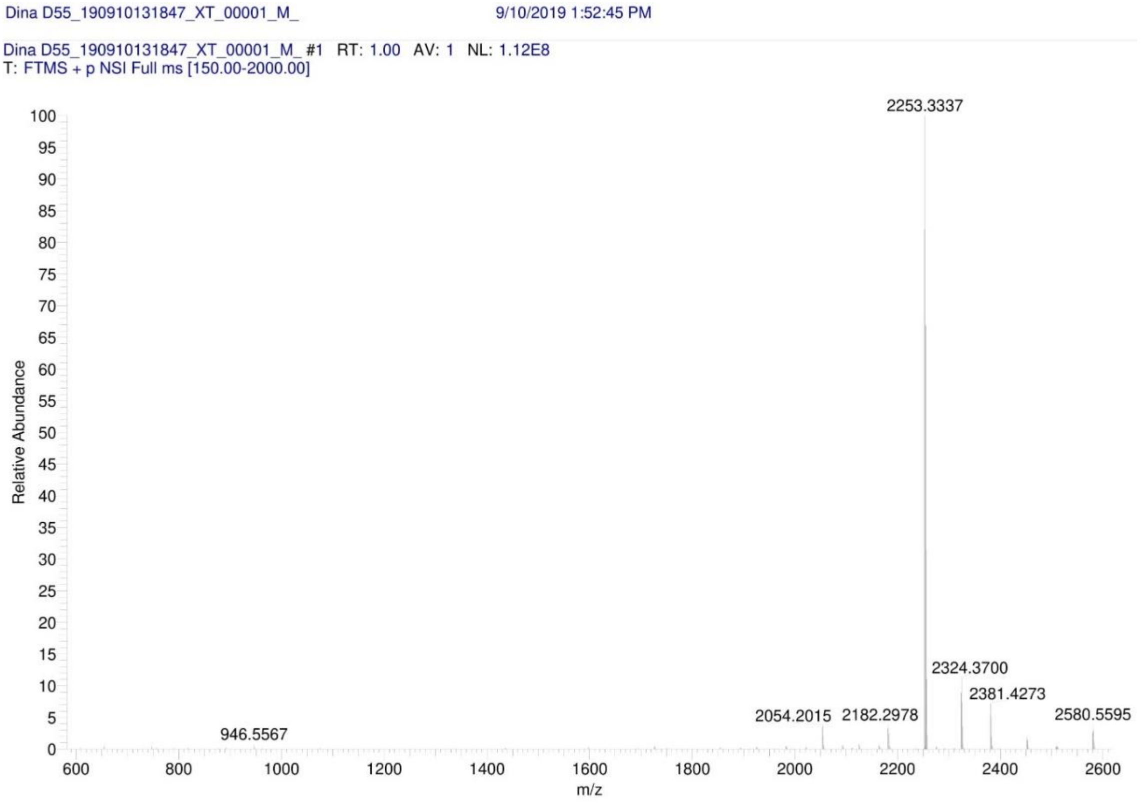
Dina D55_190910131847_XT_00001_M_ 9/10/2019 1:52:45 PM
Dina D55_190910131847_XT_00001_M_ #1 RT: 1.00 AV: 1 NL: 1.12E8
T: FTMS + p NSI Full ms [150.00-2000.00]
2253.3337
2324.3700
2381.4273
2054.2015
2182.2978
2580.5595
946.5567
Relative Abundance
m/z

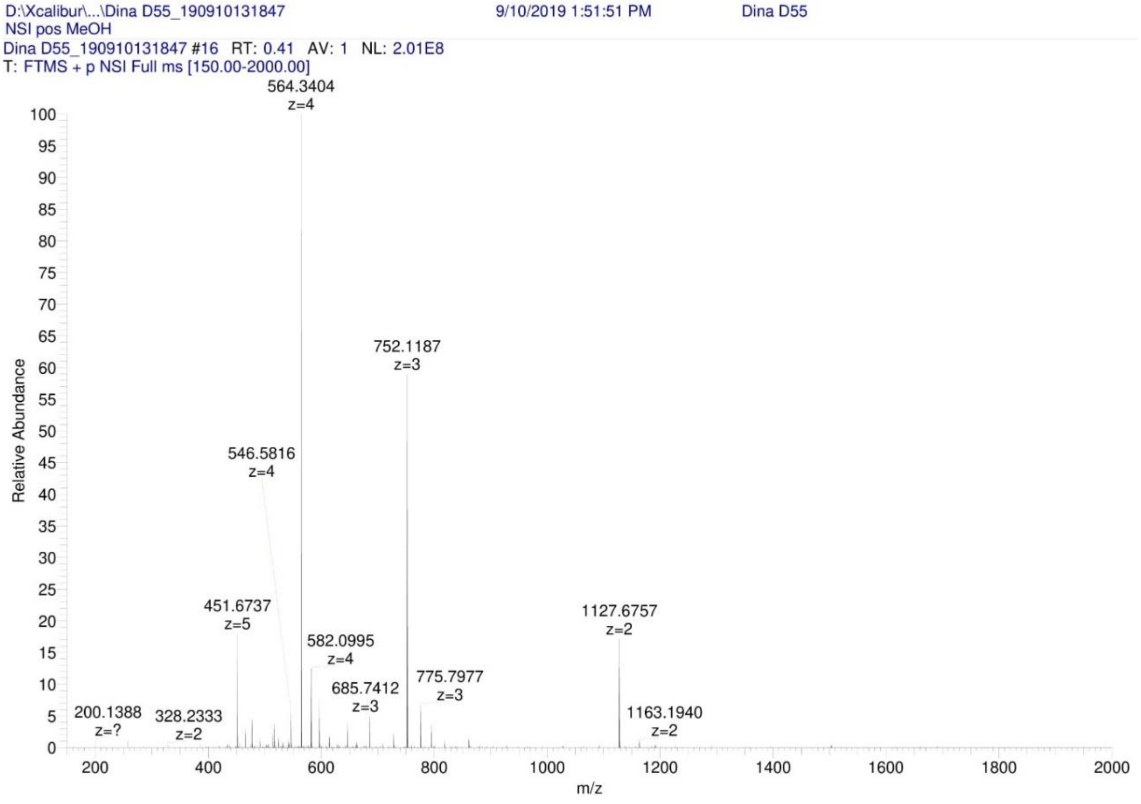
D:\Xcalibur\...\Dina D55_190910131847 9/10/2019 1:51:51 PM Dina D55
NSI pos MeOH
Dina D55_190910131847 #16 RT: 0.41 AV: 1 NL: 2.01E8
T: FTMS + p NSI Full ms [150.00-2000.00]
564.3404 z=4
752.1187 z=3
546.5816 z=4
451.6737 z=5
1127.6757 z=2
582.0995 z=4
775.7977 z=3
685.7412 z=3
200.1388 z=?
328.2333 z=2
1163.1940 z=2
Relative Abundance
m/z

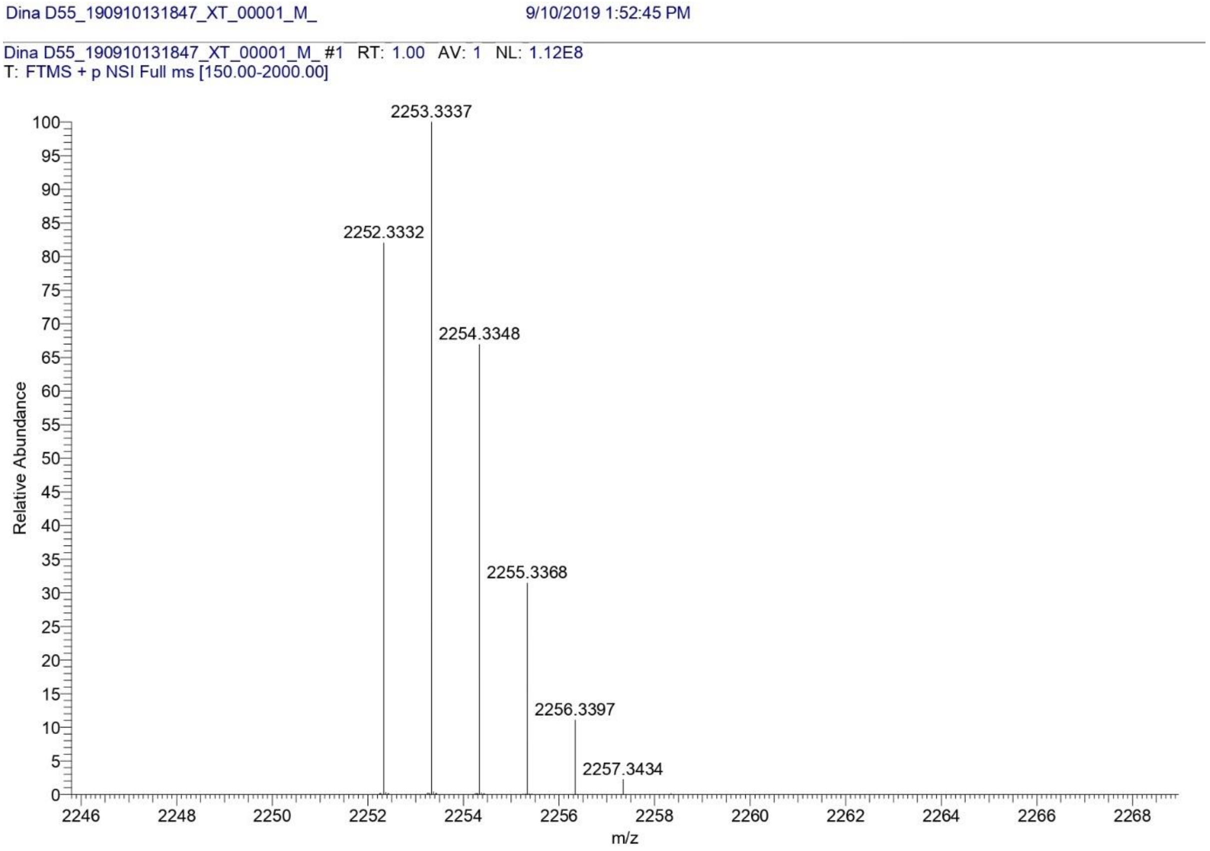

Dina D55_190910131847_XT_00001_M_
9/10/2019 1:52:45 PM
Dina D55_190910131847_XT_00001_M_ #1 RT: 1.00 AV: 1 NL: 1.12E8
T: FTMS + p NSI Full ms [150.00-2000.00]
2253.3337
2252.3332
2254.3348
2255.3368
2256.3397
2257.3434
Relative Abundance
m/z

Ac**8 ((AcAK)4(*K*KYE)2*K*AAA-OH)** was obtained from the CEM Liberty Blue synthetiser as foamy colourless solid after preparative RP-HPLC (10.4 mg, 2.9 µmol, 8.9%). Analytical RP-HPLC: t_R=1.28 min (100% A to 100% D in 5 min, λ= 214 nm). HRMS (ESI+): $C_{111}H_{185}N_{29}O_{31}$ calc./obs. 2420.3791/2420.3770 Da [M].

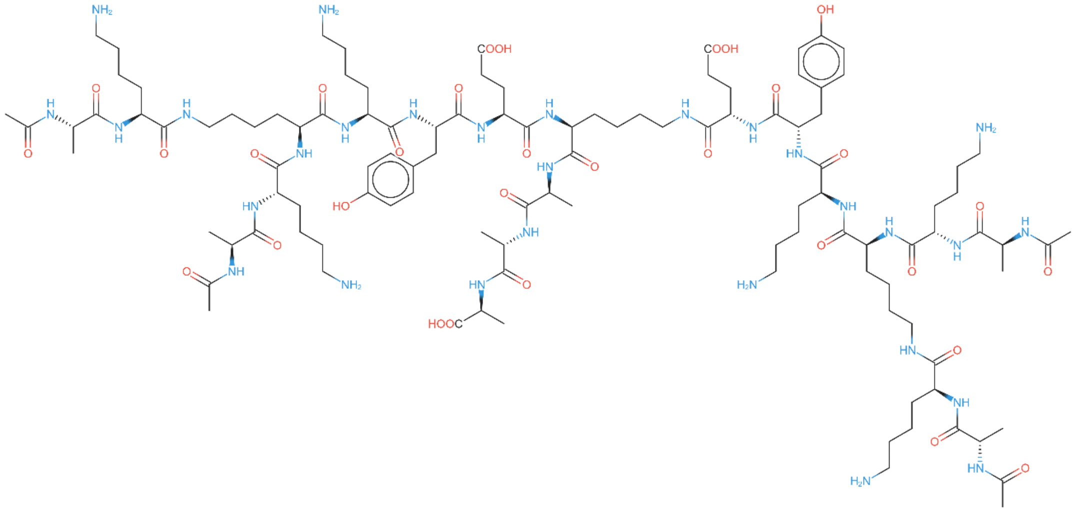

Analytical RP-HPLC:

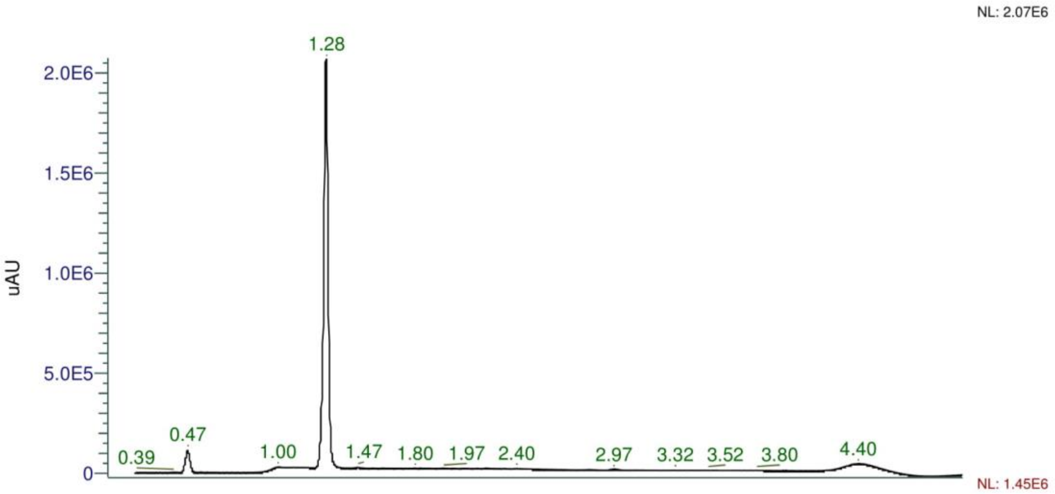

HRMS (NSI+):

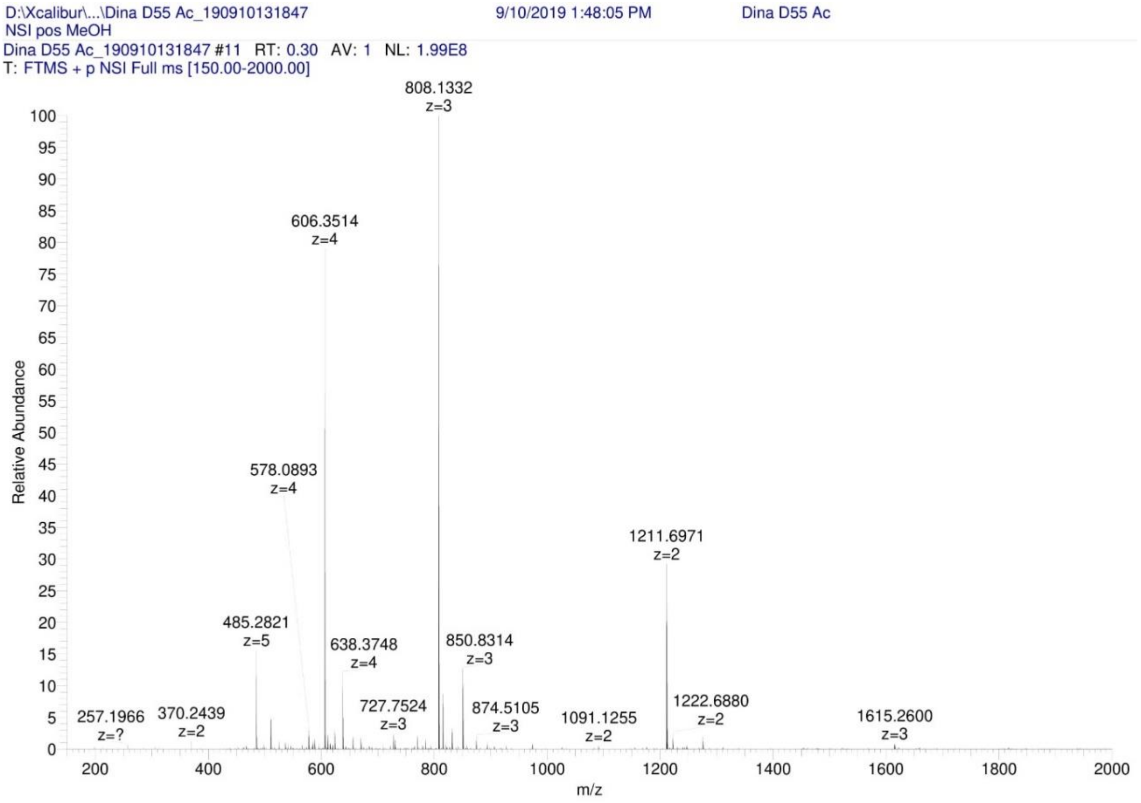
D:\Xcalibur\...\Dina D55 Ac_190910131847
9/10/2019 1:48:05 PM
Dina D55 Ac
NSI pos MeOH
Dina D55 Ac_190910131847 #11 RT: 0.30 AV: 1 NL: 1.99E8
T: FTMS + p NSI Full ms [150.00-2000.00]
808.1332
z=3
606.3514
z=4
578.0893
z=4
1211.6971
z=2
485.2821
z=5
638.3748
z=4
850.8314
z=3
727.7524
z=3
874.5105
z=3
1222.6880
z=2
257.1966
z=?
370.2439
z=2
1091.1255
z=2
1615.2600
z=3
Relative Abundance
m/z

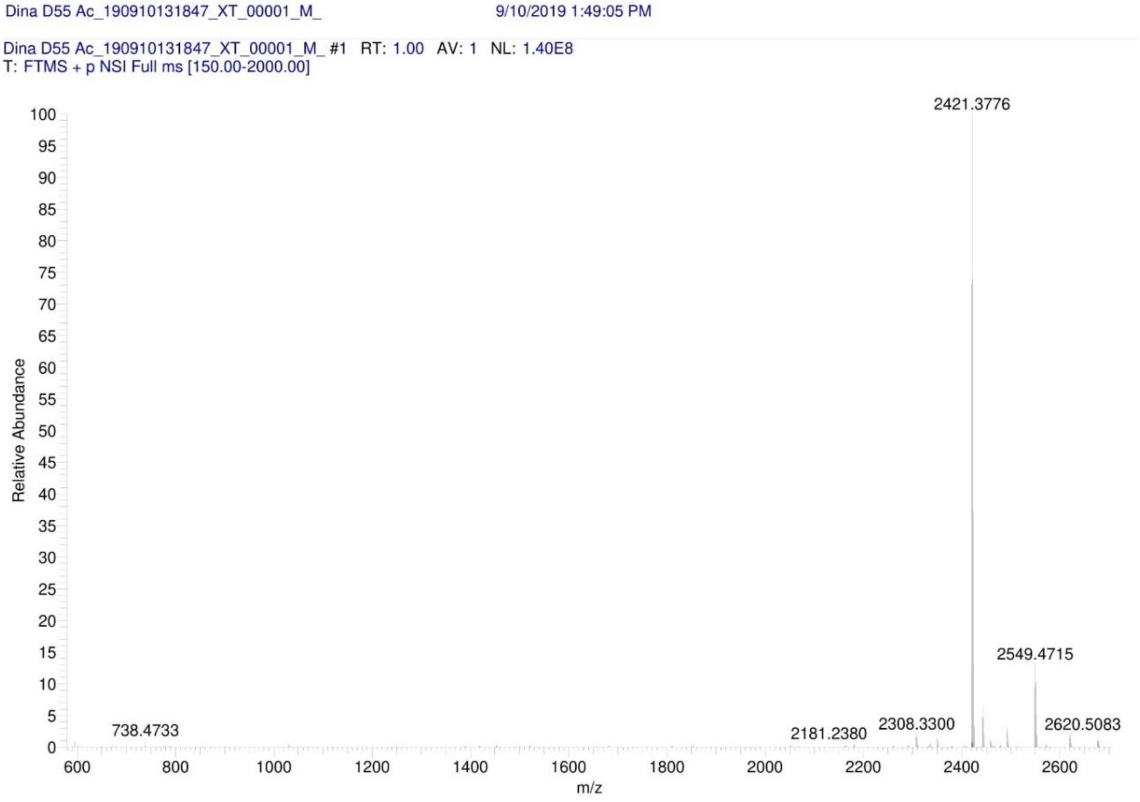
Dina D55 Ac_190910131847_XT_00001_M_
9/10/2019 1:49:05 PM
Dina D55 Ac_190910131847_XT_00001_M_ #1 RT: 1.00 AV: 1 NL: 1.40E8
T: FTMS + p NSI Full ms [150.00-2000.00]
2421.3776
2549.4715
738.4733
2181.2380
2308.3300
2620.5083
Relative Abundance
m/z

Dina D55 Ac_190910131847_XT_00001_M_ 9/10/2019 1:49:05 PM

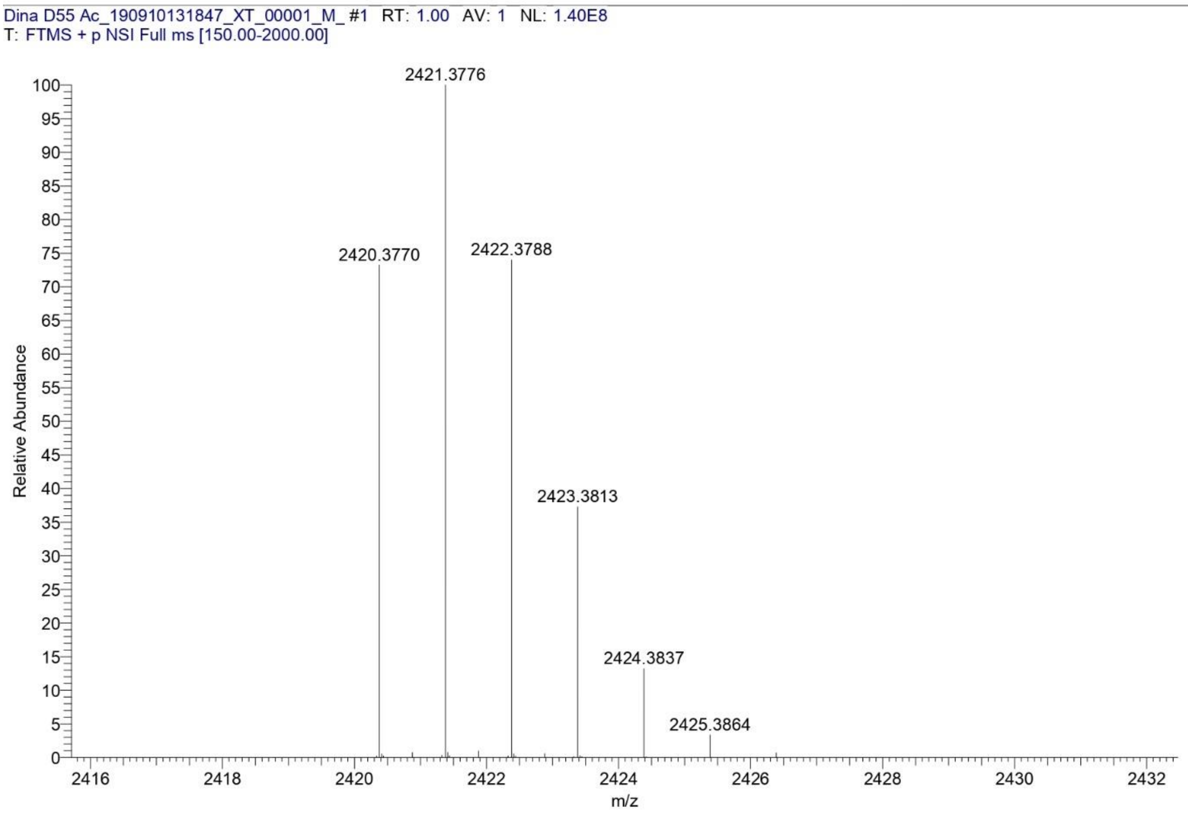

9 ((AYK)$_4$(*K*E)$_2$*K*KAA-OH) was obtained after manual synthesis as foamy colourless solid after preparative RP-HPLC (34.8 mg, 8.3 µmol, 28.4%). Analytical RP-HPLC: t_R=1.23 min (100% A to 100% D in 5 min, λ= 214 nm). HRMS (ESI+): $C_{112}H_{178}N_{28}O_{29}$ calc./obs. 2379.3315/2379.3345 Da [M].

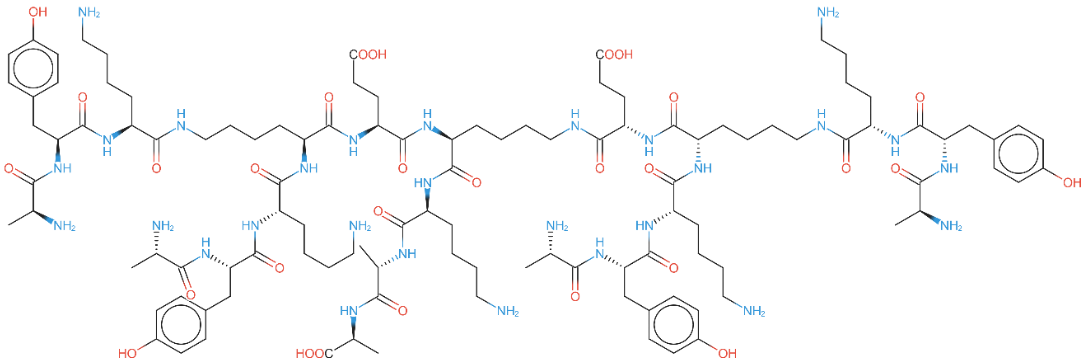

Analytical RP-HPLC:

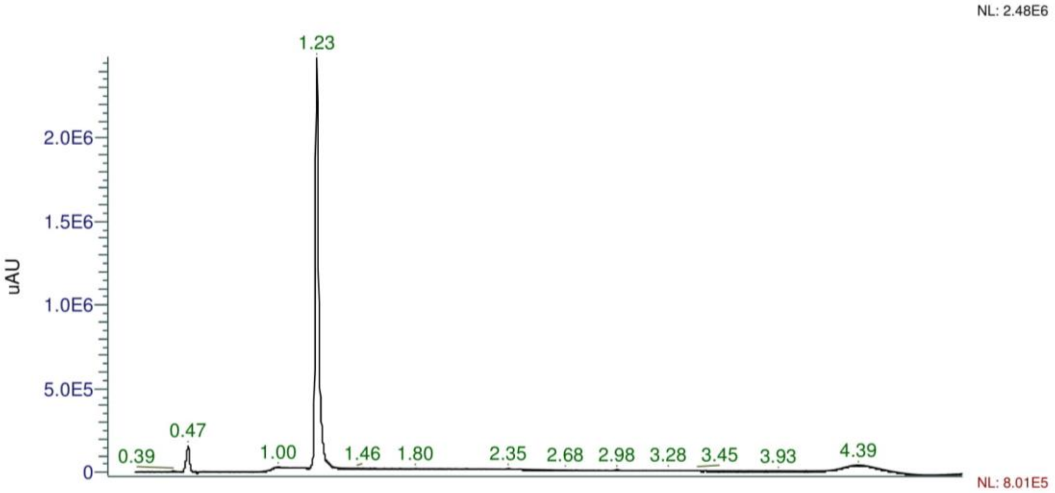

HRMS (NSI+):

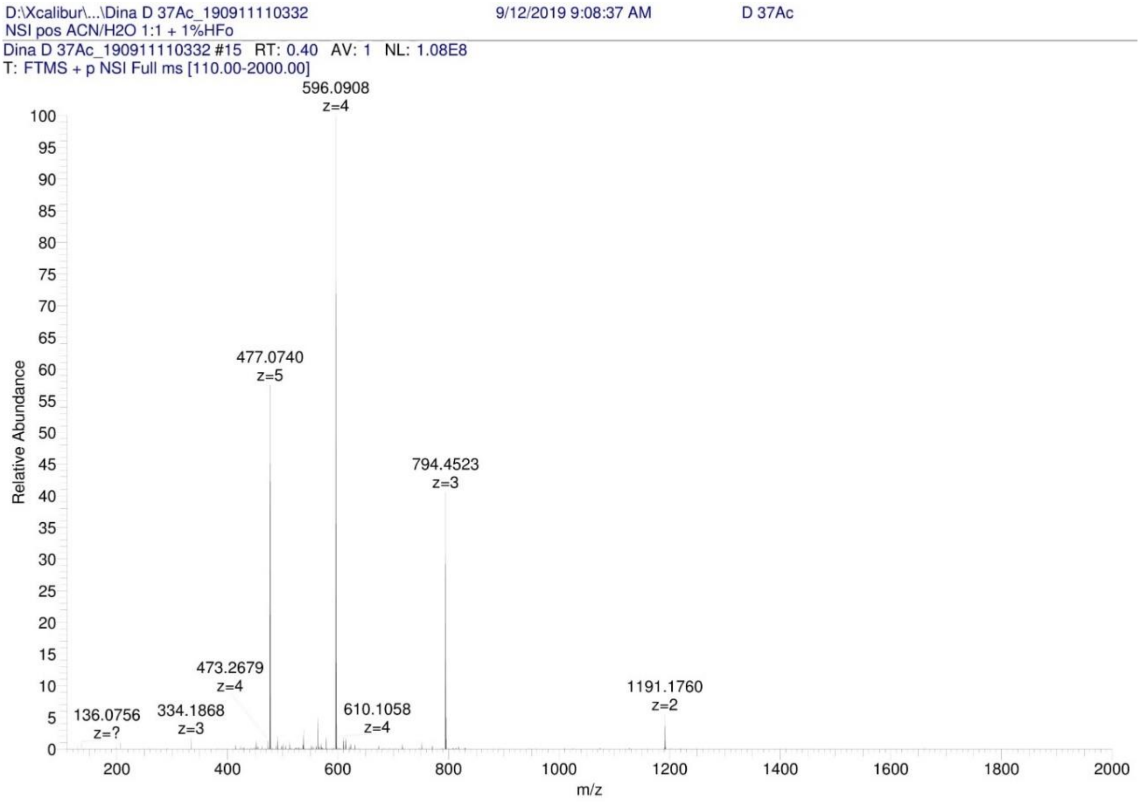
D:\Xcalibur\...\Dina D 37Ac_190911110332
9/12/2019 9:08:37 AM
D 37Ac
NSI pos ACN/H2O 1:1 + 1%HFo
Dina D 37Ac_190911110332 #15 RT: 0.40 AV: 1 NL: 1.08E8
T: FTMS + p NSI Full ms [110.00-2000.00]
596.0908 z=4
477.0740 z=5
794.4523 z=3
473.2679 z=4
1191.1760 z=2
136.0756 z=?
334.1868 z=3
610.1058 z=4
Relative Abundance
m/z

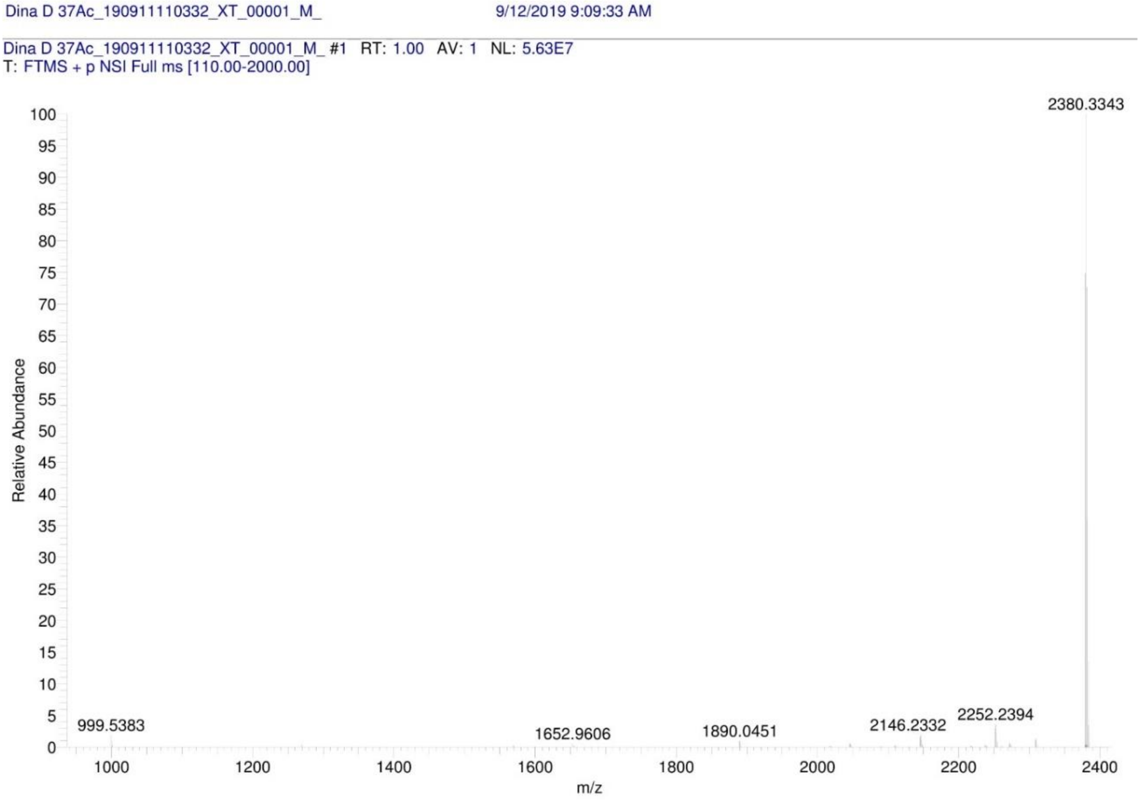
Dina D 37Ac_190911110332_XT_00001_M_
9/12/2019 9:09:33 AM
Dina D 37Ac_190911110332_XT_00001_M_ #1 RT: 1.00 AV: 1 NL: 5.63E7
T: FTMS + p NSI Full ms [110.00-2000.00]
2380.3343
999.5383
1652.9606
1890.0451
2146.2332
2252.2394
Relative Abundance
m/z

Dina D 37Ac_190911110332_XT_00001_M_ 9/12/2019 9:09:33 AM

Dina D 37Ac_190911110332_XT_00001_M_ #1 RT: 1.00 AV: 1 NL: 5.63E7
T: FTMS + p NSI Full ms [110.00-2000.00]

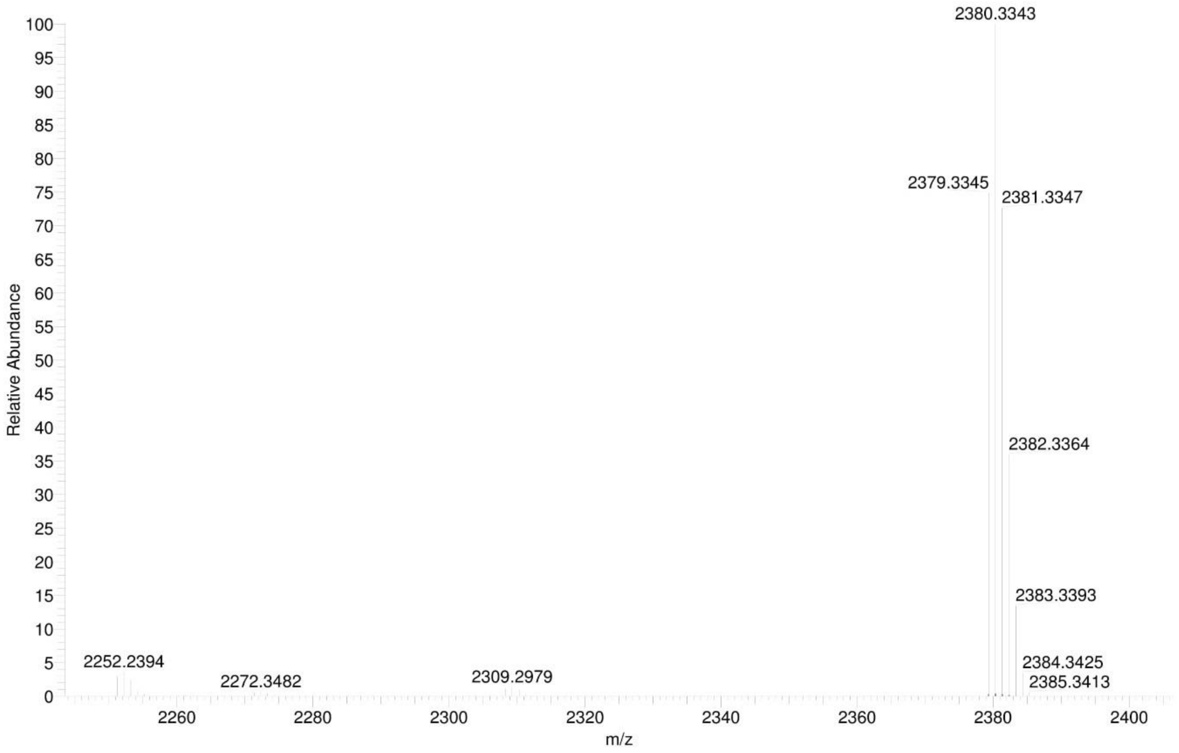

Ac**9 ((AcAYK)$_4$(*K*E)$_2$*K*KAA-OH)** was obtained after manual synthesis as foamy colourless solid after preparative RP-HPLC (50.2 mg, 9.6 µmol, 28.4%). Analytical RP-HPLC: t_R=1.36 min (100% A to 100% D in 5 min, λ= 214 nm). HRMS (ESI+): $C_{120}H_{186}N_{28}O_{33}$ calc./obs. 2547.3315/2547.3775 Da [M].

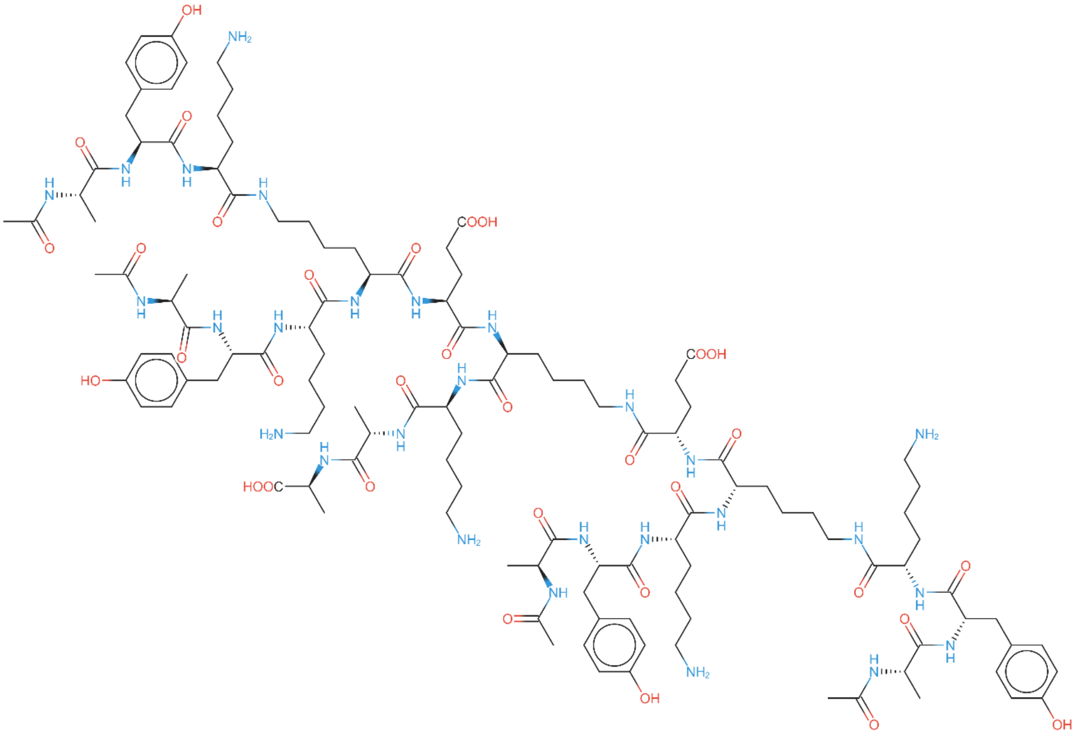

Analytical RP-HPLC:

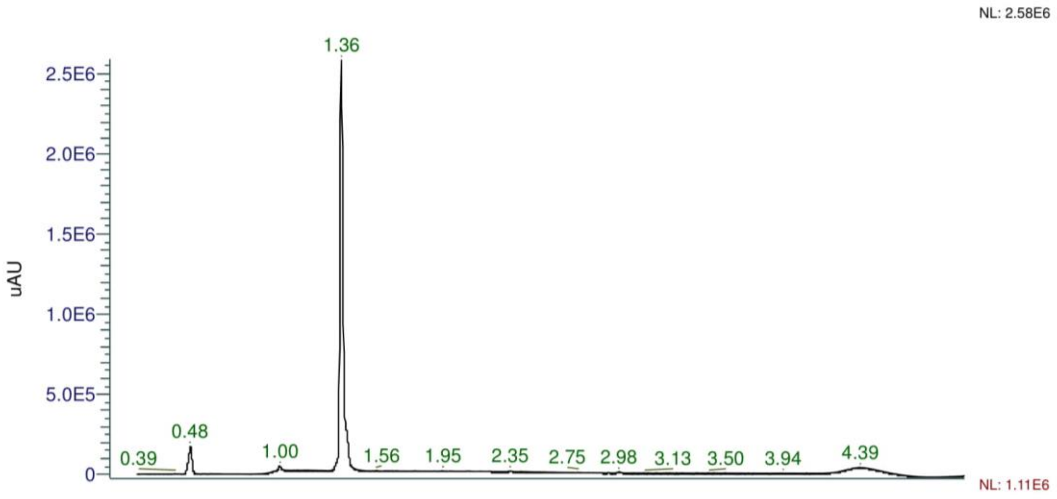

HRMS (NSI+):

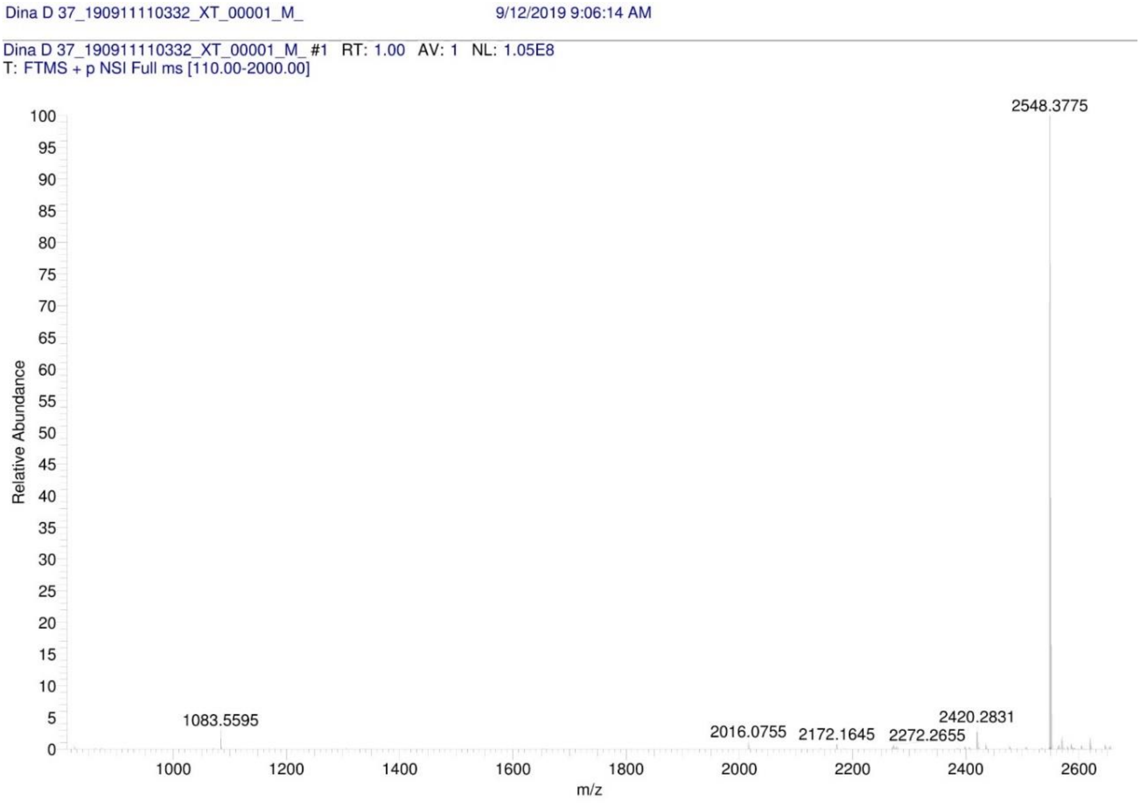
Dina D 37_190911110332_XT_00001_M_
9/12/2019 9:06:14 AM
Dina D 37_190911110332_XT_00001_M_ #1 RT: 1.00 AV: 1 NL: 1.05E8
T: FTMS + p NSI Full ms [110.00-2000.00]
2548.3775
1083.5595
2016.0755
2172.1645
2272.2655
2420.2831
Relative Abundance
m/z

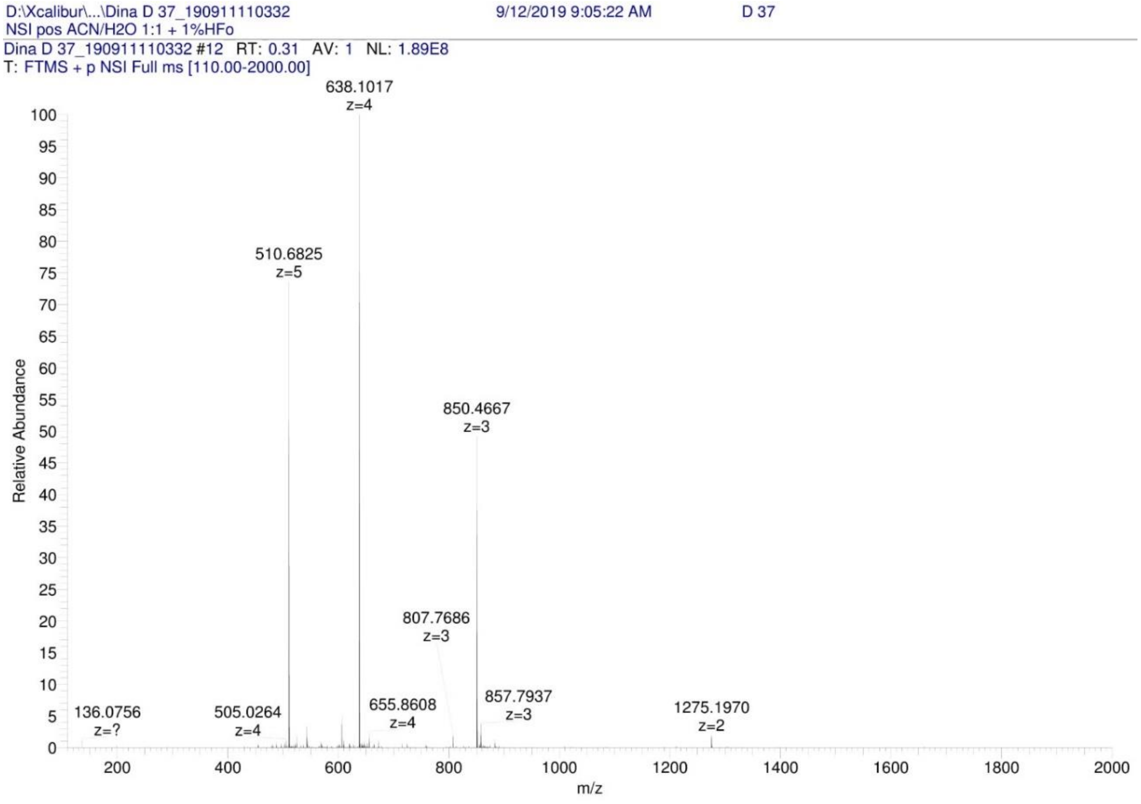
D:\Xcalibur\...\Dina D 37_190911110332
9/12/2019 9:05:22 AM
D 37
NSI pos ACN/H2O 1:1 + 1%HFo
Dina D 37_190911110332 #12 RT: 0.31 AV: 1 NL: 1.89E8
T: FTMS + p NSI Full ms [110.00-2000.00]
638.1017 z=4
510.6825 z=5
850.4667 z=3
807.7686 z=3
136.0756 z=?
505.0264 z=4
655.8608 z=4
857.7937 z=3
1275.1970 z=2
Relative Abundance
m/z

Dina D 37_190911110332_XT_00001_M_ 9/12/2019 9:06:14 AM

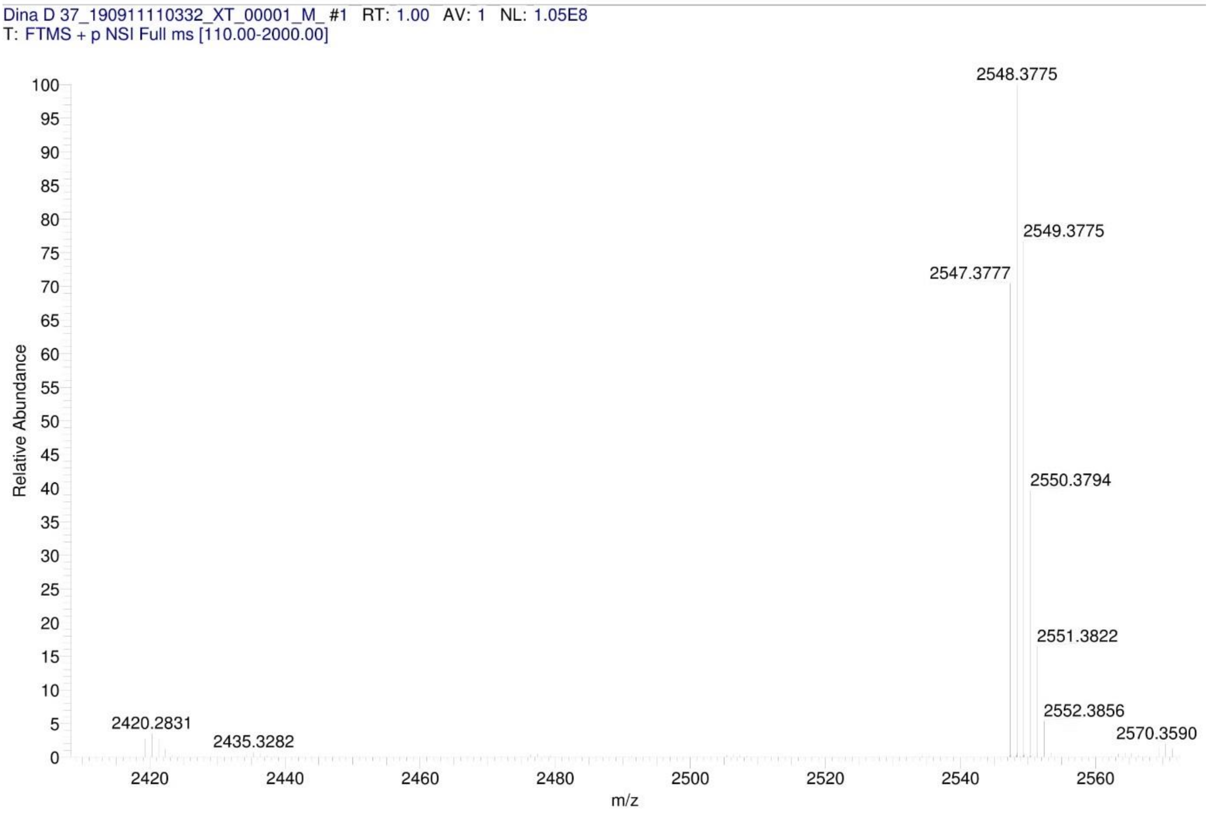

10 ((AKA)$_4$(*K*KEY)$_2$*K*E-OH) was obtained after manual synthesis as foamy colourless solid after preparative RP-HPLC (24.3 mg, 4.9 µmol, 17.6%). Analytical RP-HPLC: t_R=1.20 min (100% A to 100% D in 5 min, λ= 214 nm). HRMS (ESI+): $C_{111}H_{189}N_{31}O_{31}$ calc./obs. 2452.4166/2452.4170 Da [M].

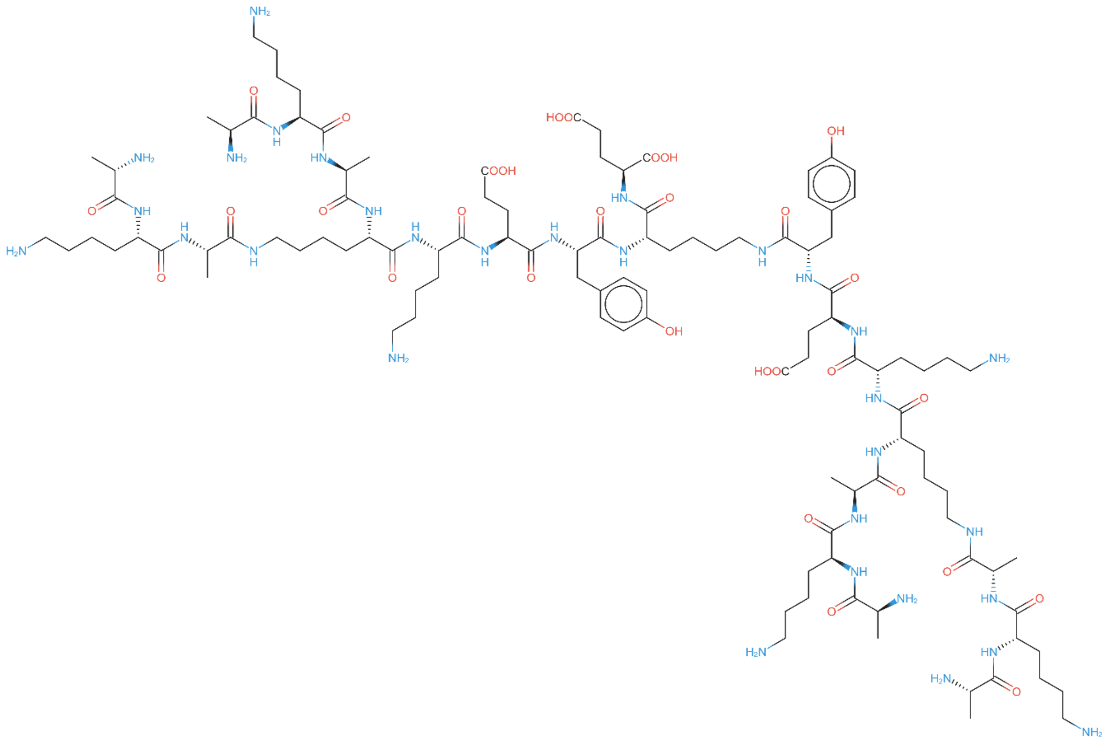

Analytical RP-HPLC:

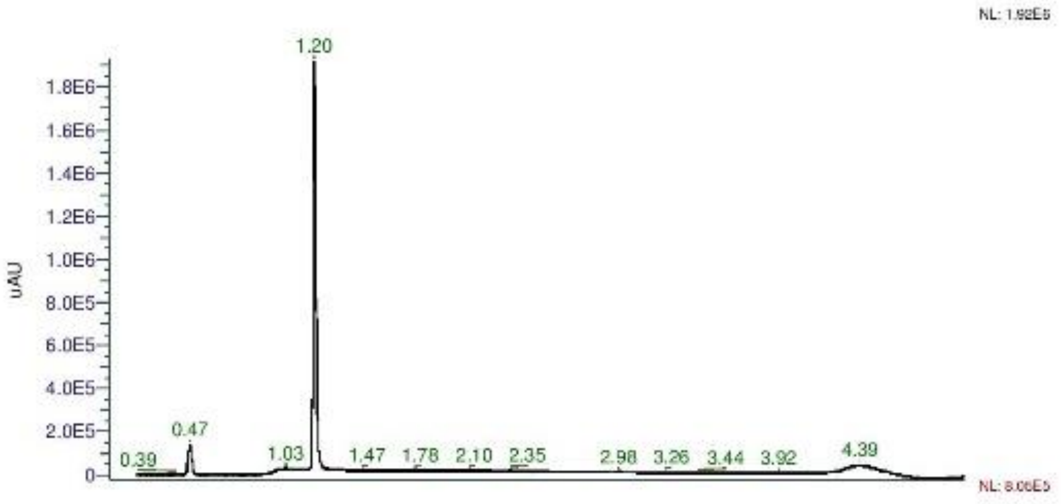

HRMS (NSI+):

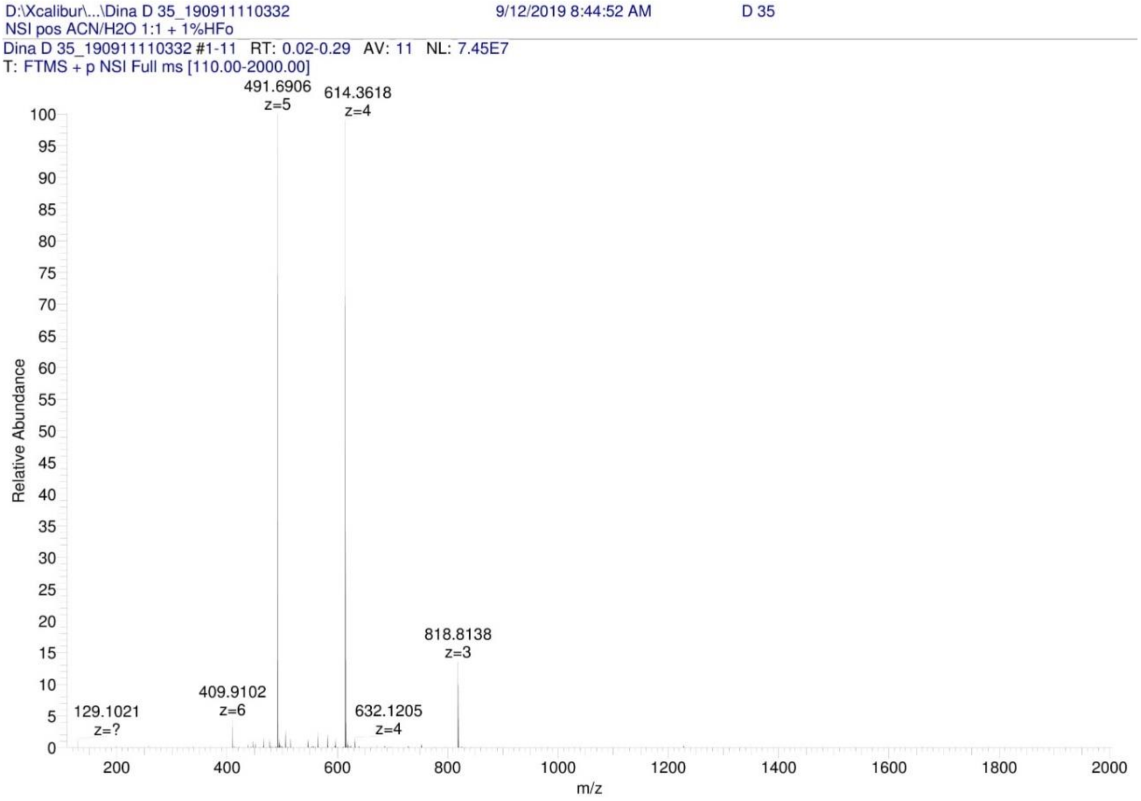

D:\Xcalibur\...\Dina D 35_190911110332
9/12/2019 8:44:52 AM
D 35
NSI pos ACN/H2O 1:1 + 1%HFo
Dina D 35_190911110332 #1-11 RT: 0.02-0.29 AV: 11 NL: 7.45E7
T: FTMS + p NSI Full ms [110.00-2000.00]
491.6906 z=5
614.3618 z=4
818.8138 z=3
409.9102 z=6
632.1205 z=4
129.1021 z=?
Relative Abundance
m/z

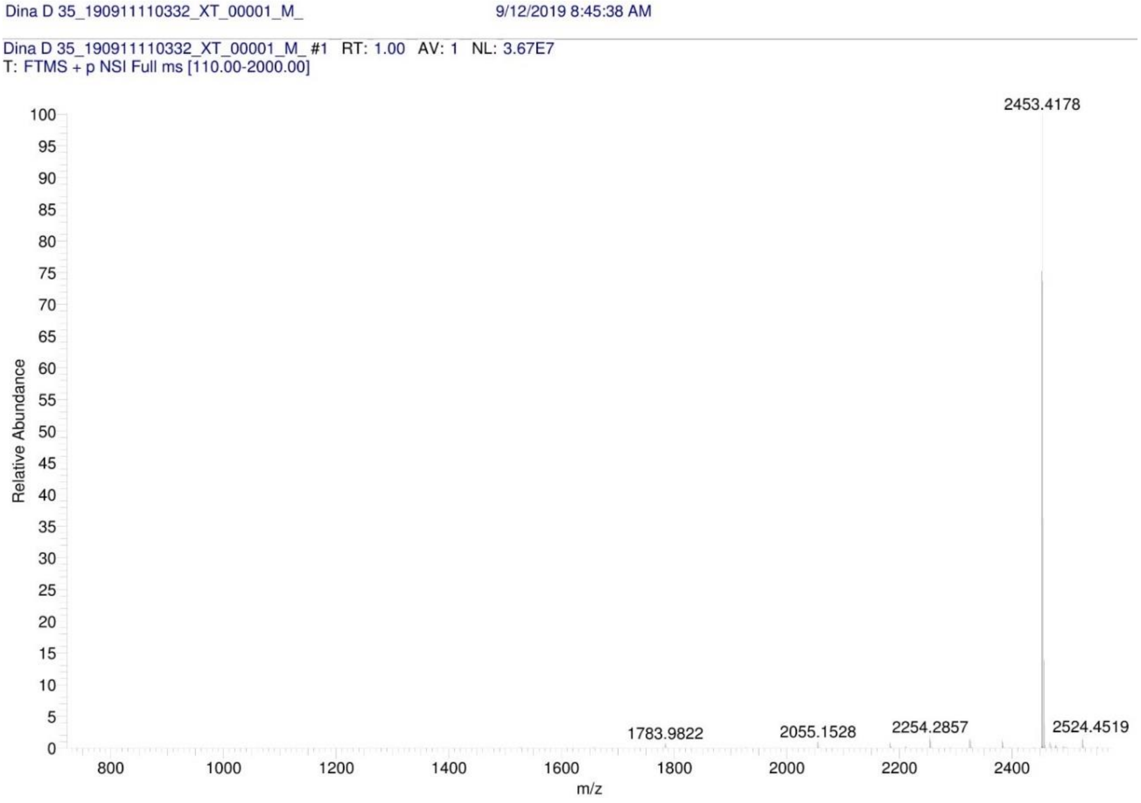

Dina D 35_190911110332_XT_00001_M_
9/12/2019 8:45:38 AM
Dina D 35_190911110332_XT_00001_M_ #1 RT: 1.00 AV: 1 NL: 3.67E7
T: FTMS + p NSI Full ms [110.00-2000.00]
2453.4178
1783.9822
2055.1528
2254.2857
2524.4519
Relative Abundance
m/z

Dina D 35_190911110332_XT_00001_M_ 9/12/2019 8:45:38 AM

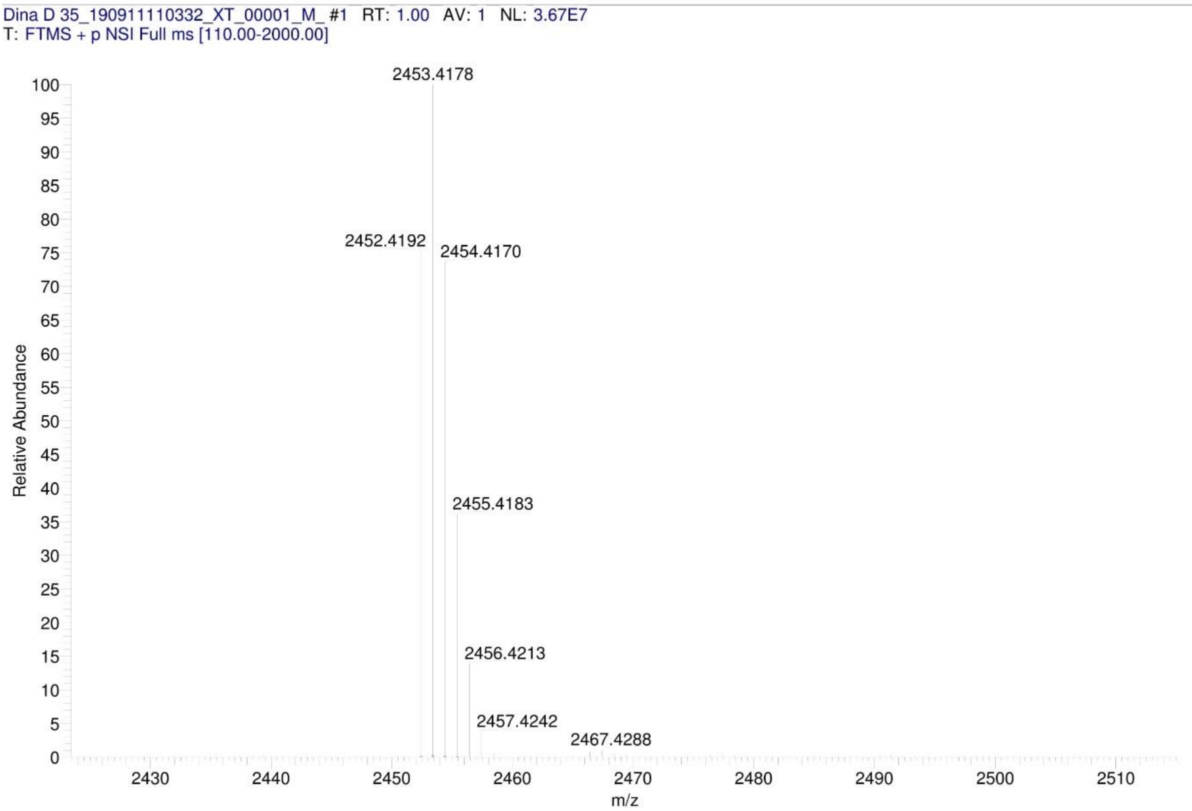

Ac**10 ((AcAKA)$_4$(*K*KEY)$_2$*K*E-OH)** was obtained after manual synthesis as foamy colourless solid after preparative RP-HPLC (49.3 mg, 9.4 µmol, 31.1%). Analytical RP-HPLC: t_R=1.30 min (100% A to 100% D in 5 min, λ= 214 nm). HRMS (ESI+): $C_{119}H_{197}N_{31}O_{35}$ calc./obs. 2620.4588/2620.4615 Da [M].

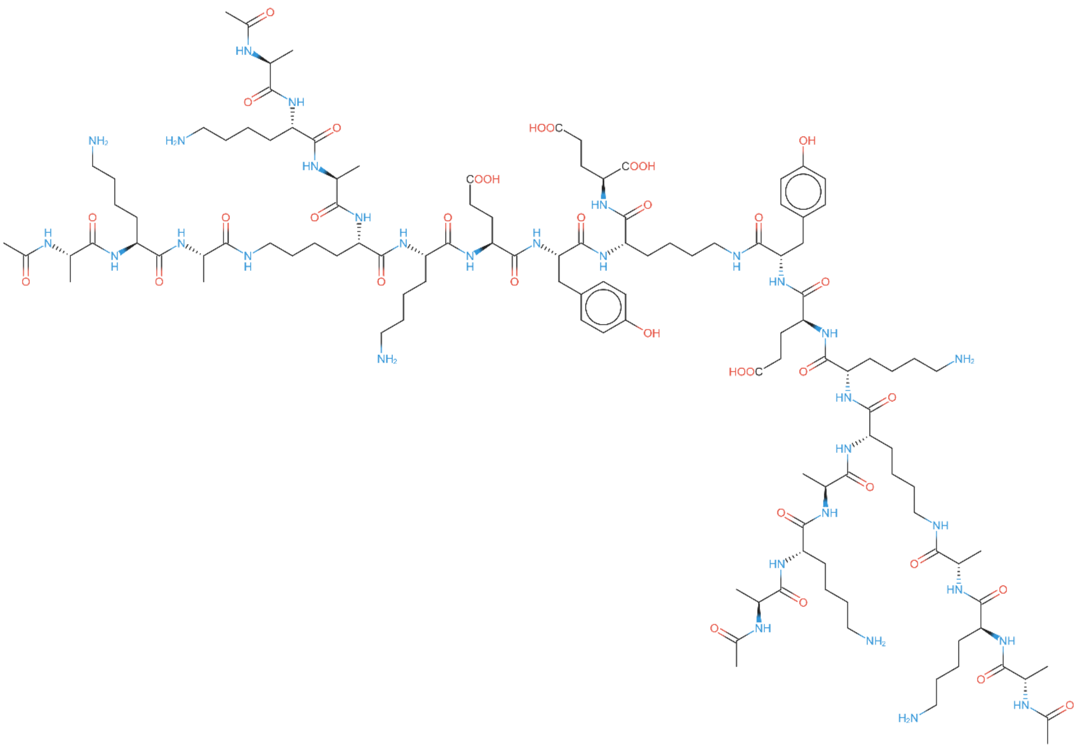

Analytical RP-HPLC:

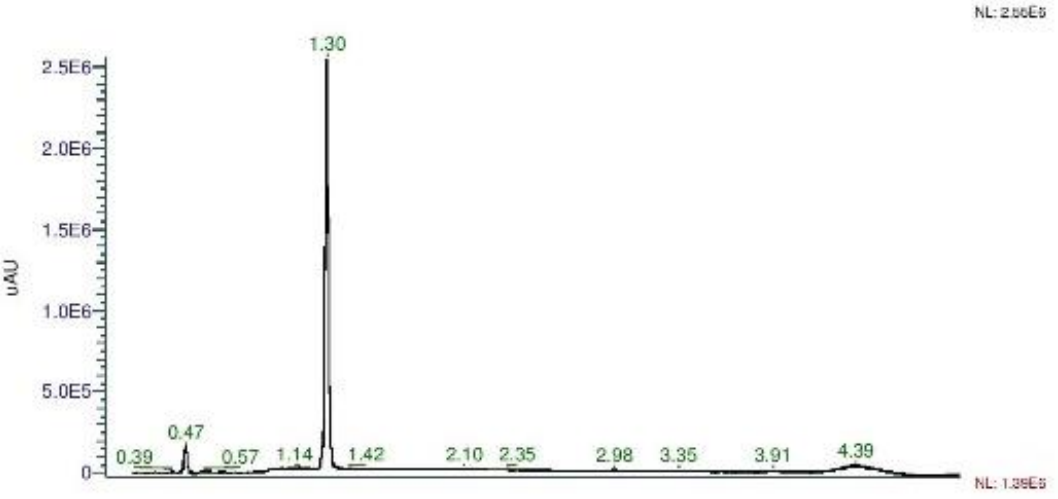

HRMS (NSI+):

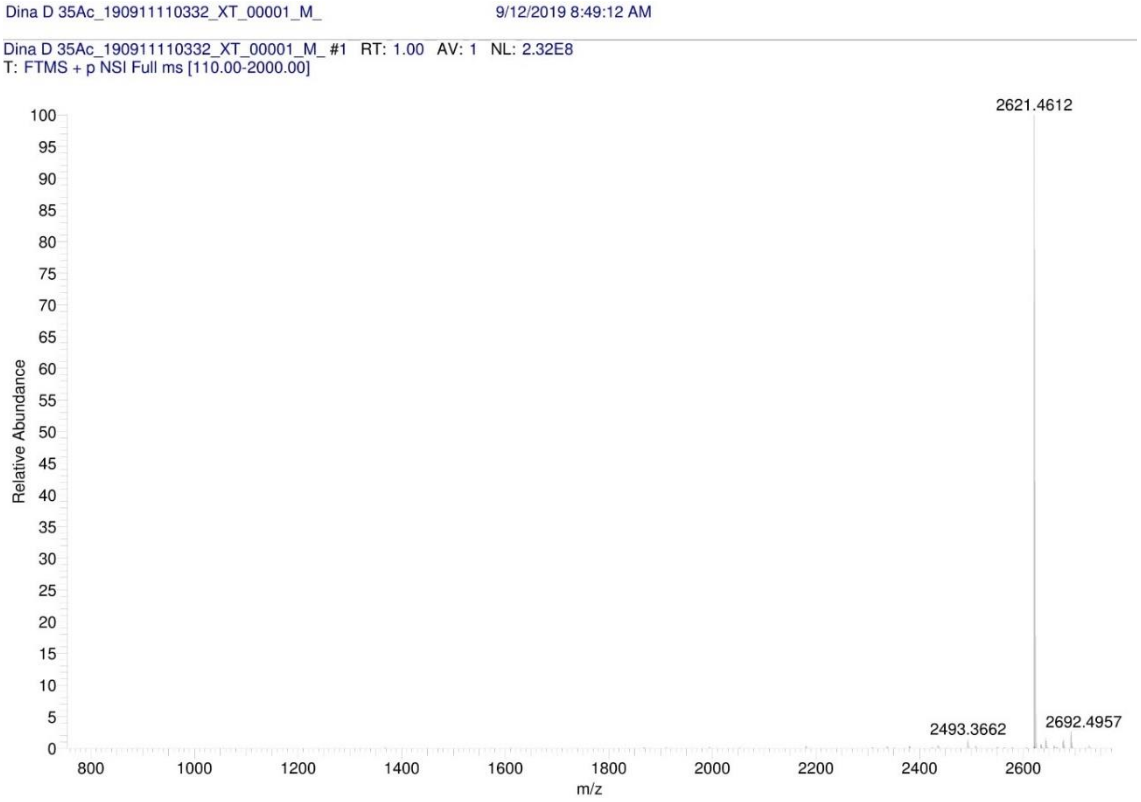
Dina D 35Ac_190911110332_XT_00001_M_ 9/12/2019 8:49:12 AM
Dina D 35Ac_190911110332_XT_00001_M_ #1 RT: 1.00 AV: 1 NL: 2.32E8
T: FTMS + p NSI Full ms [110.00-2000.00]
2621.4612
2493.3662
2692.4957
Relative Abundance
m/z

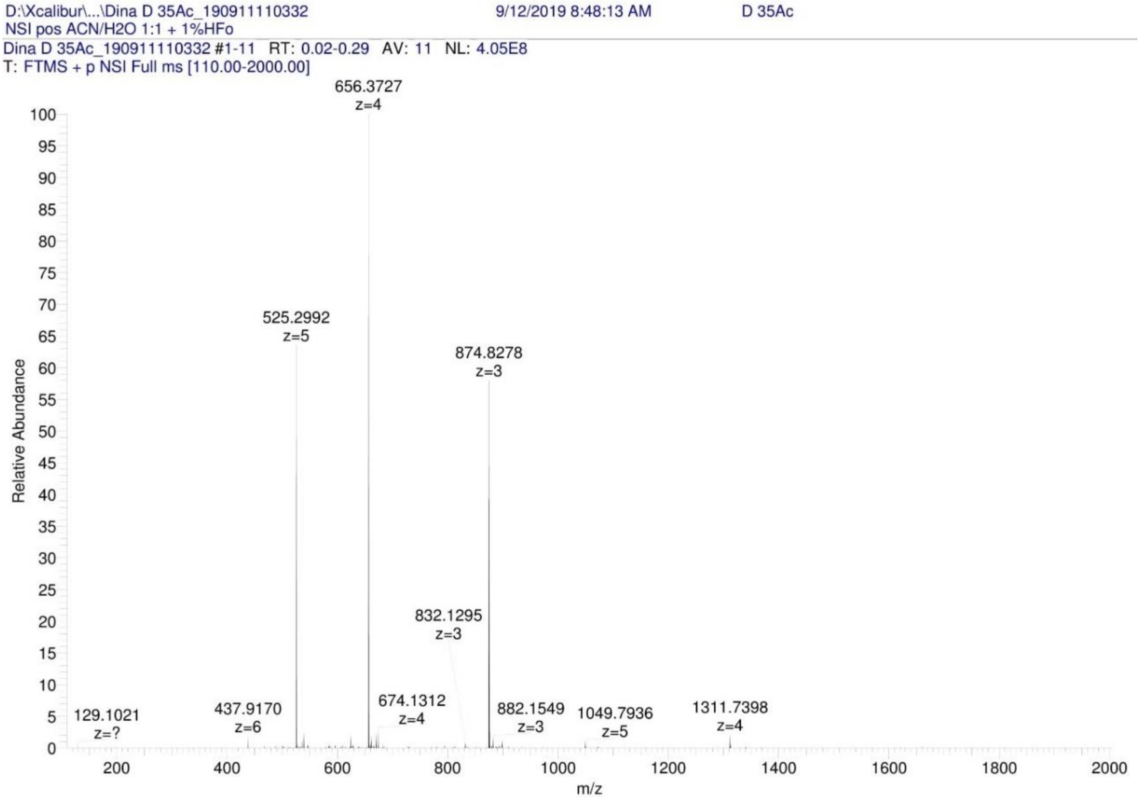
D:\Xcalibur\...\Dina D 35Ac_190911110332 9/12/2019 8:48:13 AM D 35Ac
NSI pos ACN/H2O 1:1 + 1%HFo
Dina D 35Ac_190911110332 #1-11 RT: 0.02-0.29 AV: 11 NL: 4.05E8
T: FTMS + p NSI Full ms [110.00-2000.00]
656.3727 z=4
525.2992 z=5
874.8278 z=3
832.1295 z=3
129.1021 z=?
437.9170 z=6
674.1312 z=4
882.1549 z=3
1049.7936 z=5
1311.7398 z=4
Relative Abundance
m/z

Dina D 35Ac_190911110332_XT_00001_M_ 9/12/2019 8:49:12 AM

Dina D 35Ac_190911110332_XT_00001_M_ #1 RT: 1.00 AV: 1 NL: 2.32E8
T: FTMS + p NSI Full ms [110.00-2000.00]

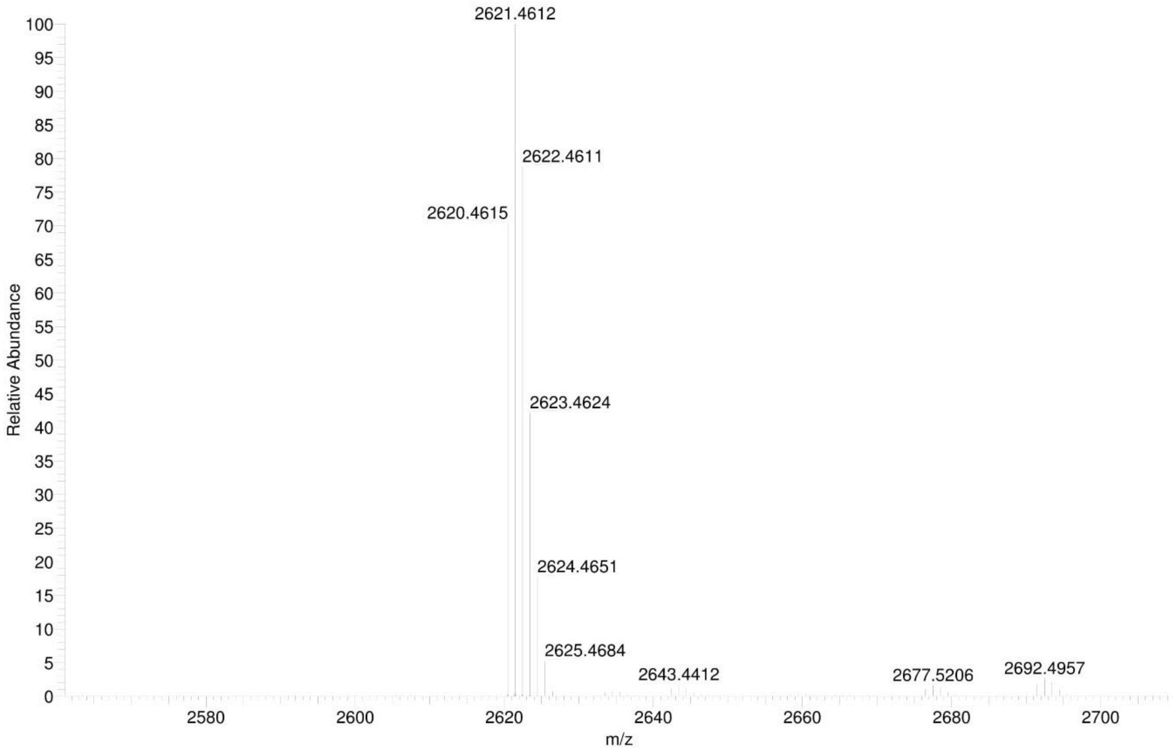

ClAc**10 ((ClAcAKA)$_4$(*K*KEY)$_2$*K*E-OH)** was obtained after manual synthesis as foamy colourless solid after preparative RP-HPLC (43.4 mg, 9.4 µmol, 32.3%). Analytical RP-HPLC: t_R=1.37 min (100% A to 100% D in 5 min, λ= 214 nm). HRMS (ESI+): $C_{119}H_{193}Cl_4N_{31}O_{35}$ calc./obs. 2756.3029/2756.3066 Da [M].

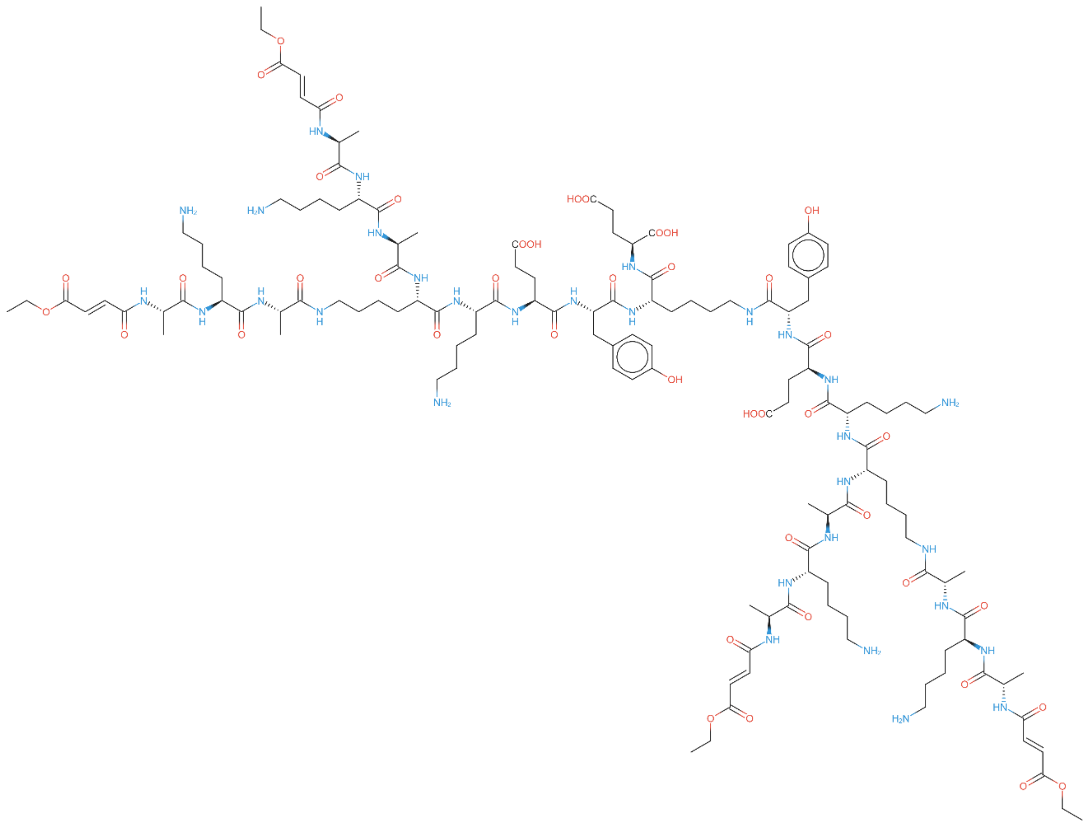

Analytical RP-HPLC:

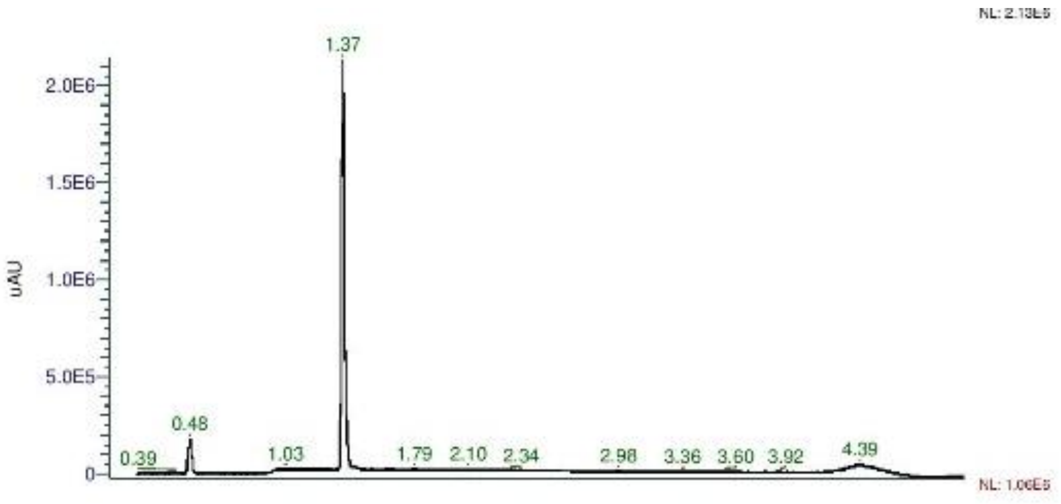

HRMS (NSI+):

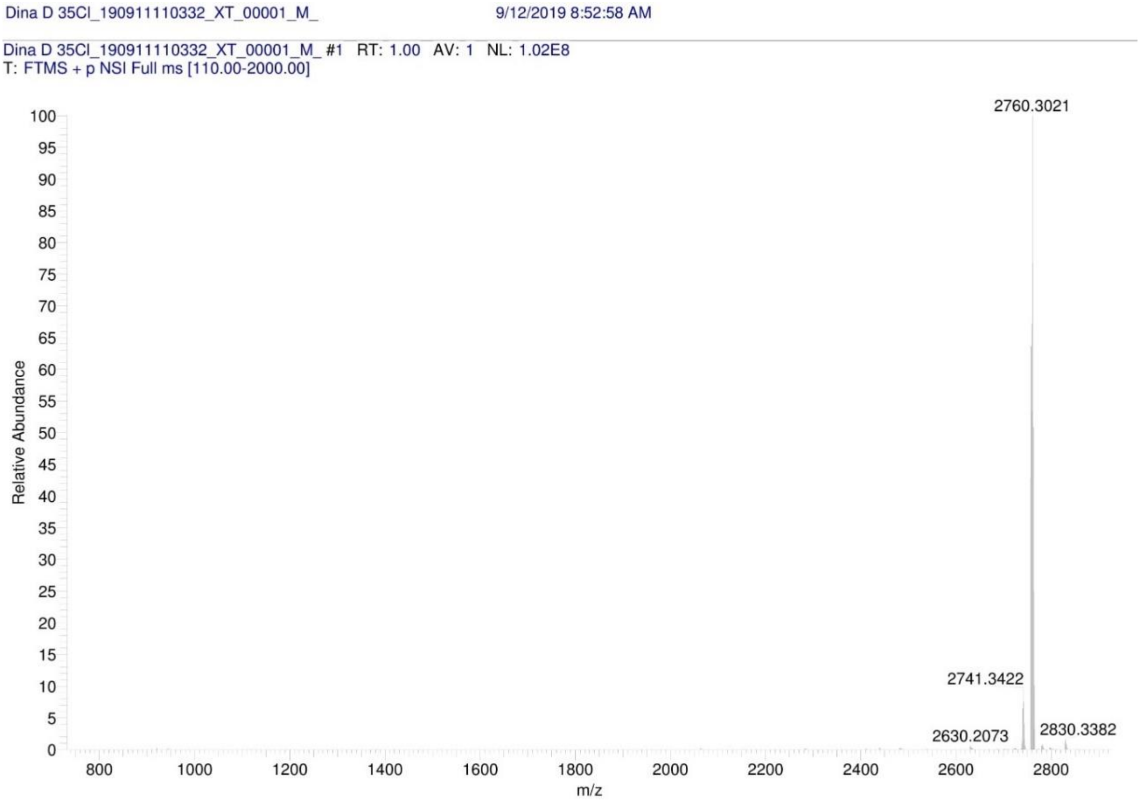

Dina D 35Cl_190911110332_XT_00001_M_
9/12/2019 8:52:58 AM
Dina D 35Cl_190911110332_XT_00001_M_ #1 RT: 1.00 AV: 1 NL: 1.02E8
T: FTMS + p NSI Full ms [110.00-2000.00]
2760.3021
2741.3422
2630.2073
2830.3382
Relative Abundance
m/z

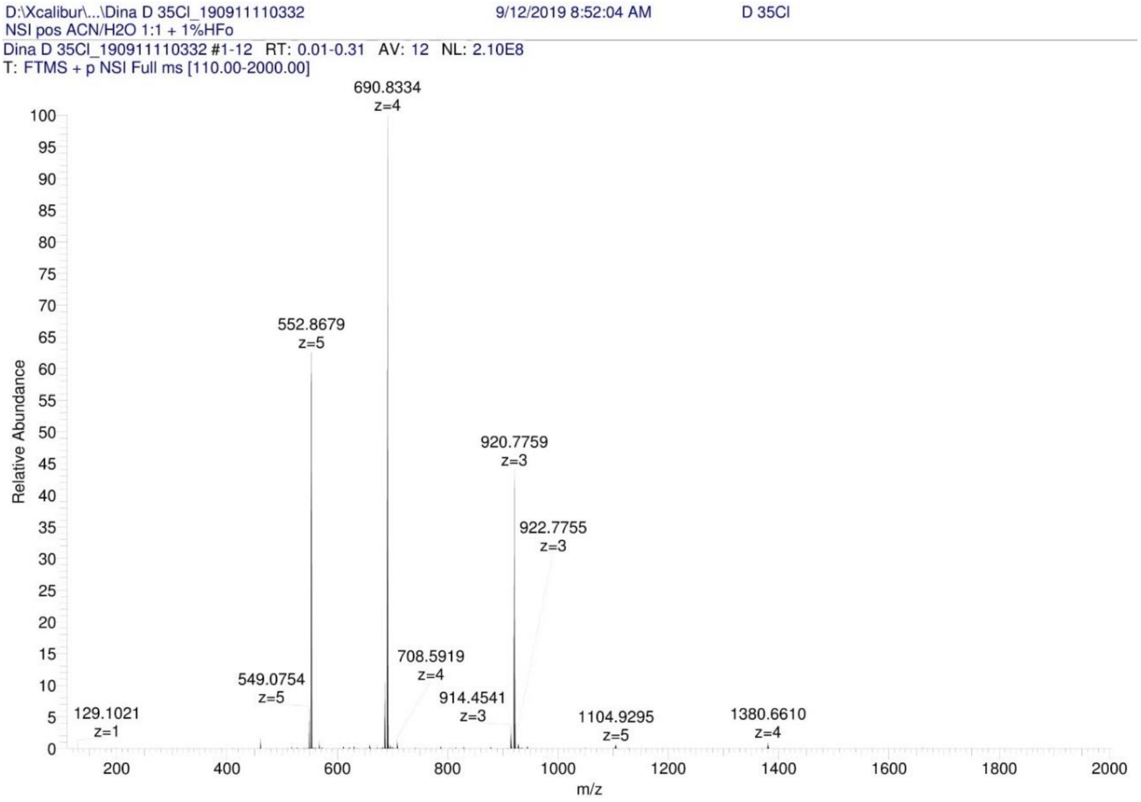

D:\Xcalibur\...\Dina D 35Cl_190911110332
9/12/2019 8:52:04 AM
D 35Cl
NSI pos ACN/H2O 1:1 + 1%HFo
Dina D 35Cl_190911110332 #1-12 RT: 0.01-0.31 AV: 12 NL: 2.10E8
T: FTMS + p NSI Full ms [110.00-2000.00]
690.8334 z=4
552.8679 z=5
920.7759 z=3
922.7755 z=3
708.5919 z=4
549.0754 z=5
914.4541 z=3
129.1021 z=1
1104.9295 z=5
1380.6610 z=4
Relative Abundance
m/z

Dina D 35Cl_190911110332_XT_00001_M_ 9/12/2019 8:52:58 AM

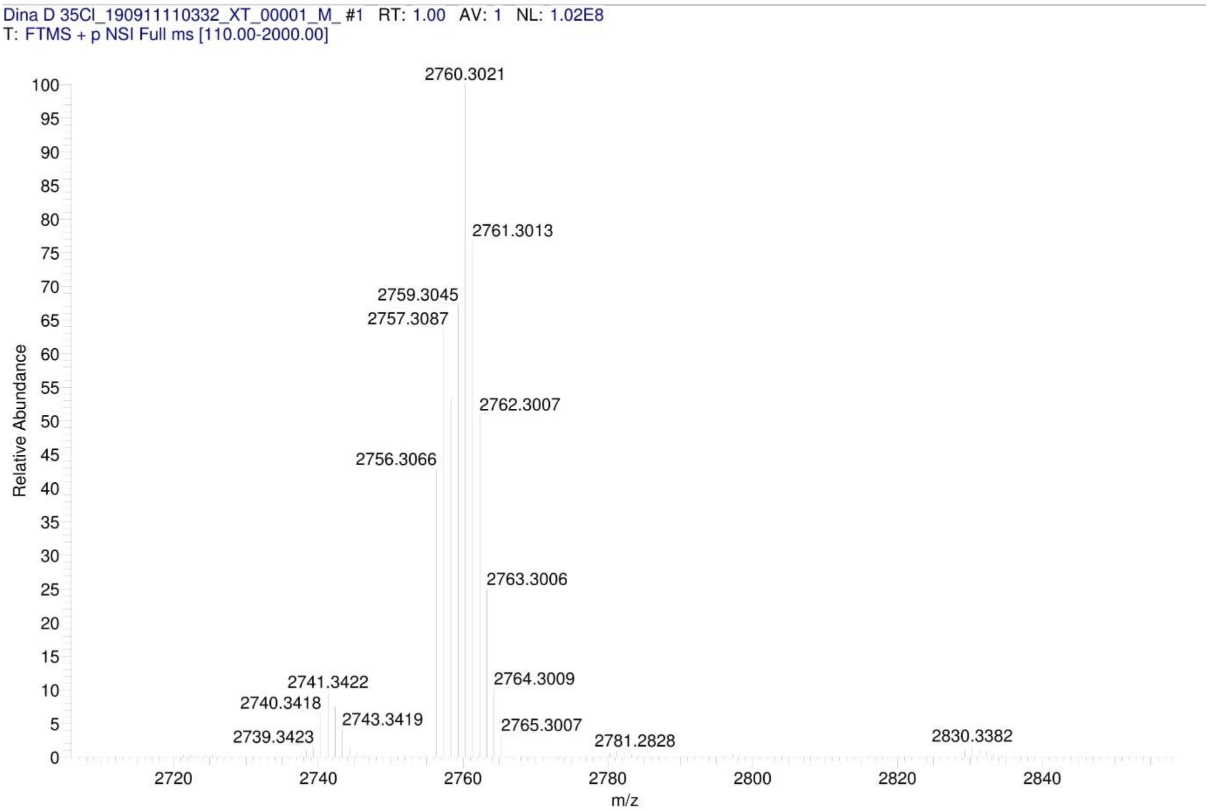

11 ((AKA)$_4$(*K*EK)$_2$*K*YEY-OH) was obtained from the CEM Liberty Blue synthetiser as foamy colourless solid after preparative RP-HPLC (34.2 mg, 7.4 µmol, 26.4%). Analytical RP-HPLC: t_R=1.23 min (100% A to 100% D in 5 min, λ= 214 nm). HRMS (ESI+): $C_{111}H_{189}N_{31}O_{31}$ calc./obs. 2452.4166/2452.4208 Da [M].

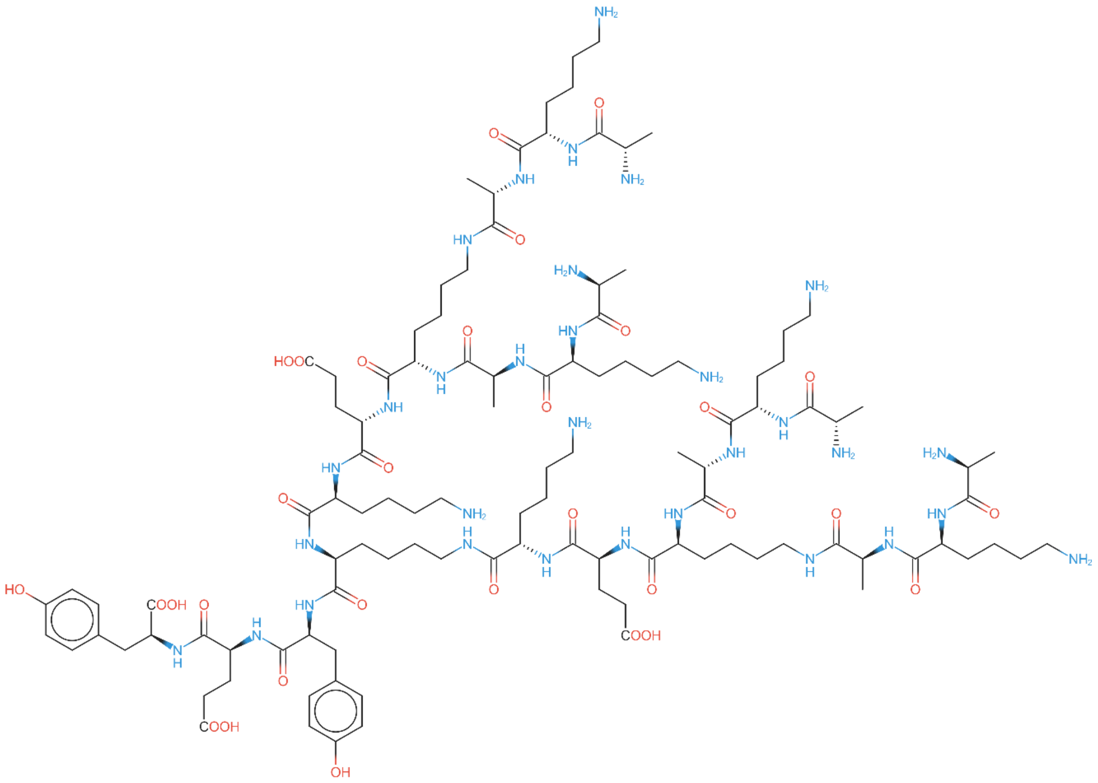

Analytical RP-HPLC:

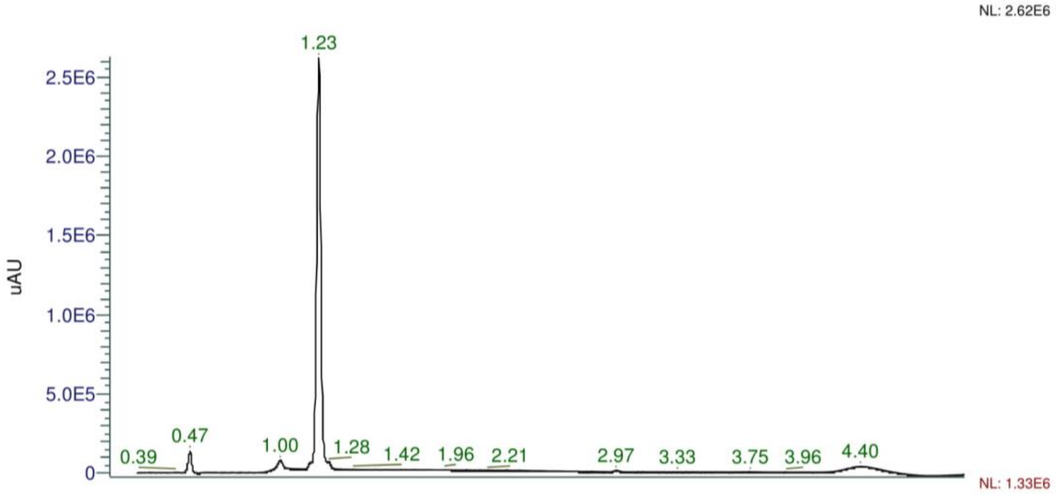

HRMS (NSI+):

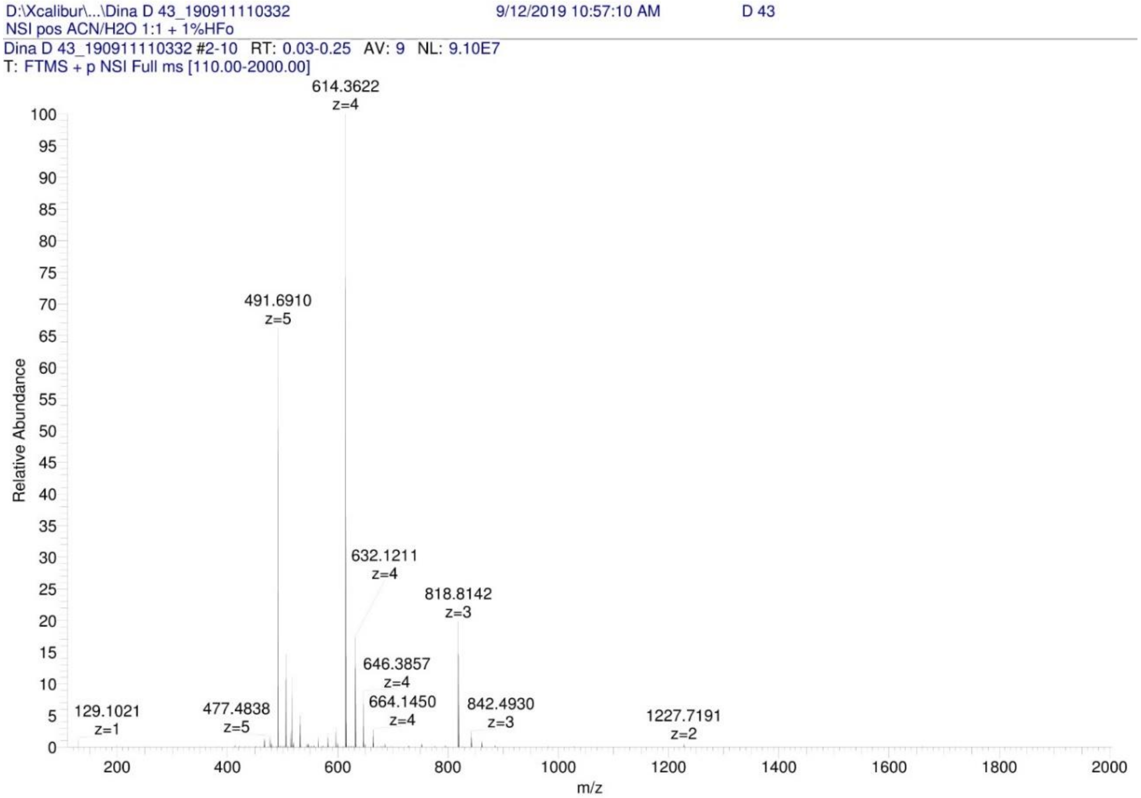
D:\Xcalibur\...\Dina D 43_190911110332
9/12/2019 10:57:10 AM
D 43
NSI pos ACN/H2O 1:1 + 1%HFo
Dina D 43_190911110332 #2-10 RT: 0.03-0.25 AV: 9 NL: 9.10E7
T: FTMS + p NSI Full ms [110.00-2000.00]
614.3622
z=4
491.6910
z=5
632.1211
z=4
818.8142
z=3
646.3857
z=4
664.1450
z=4
842.4930
z=3
129.1021
z=1
477.4838
z=5
1227.7191
z=2
Relative Abundance
m/z

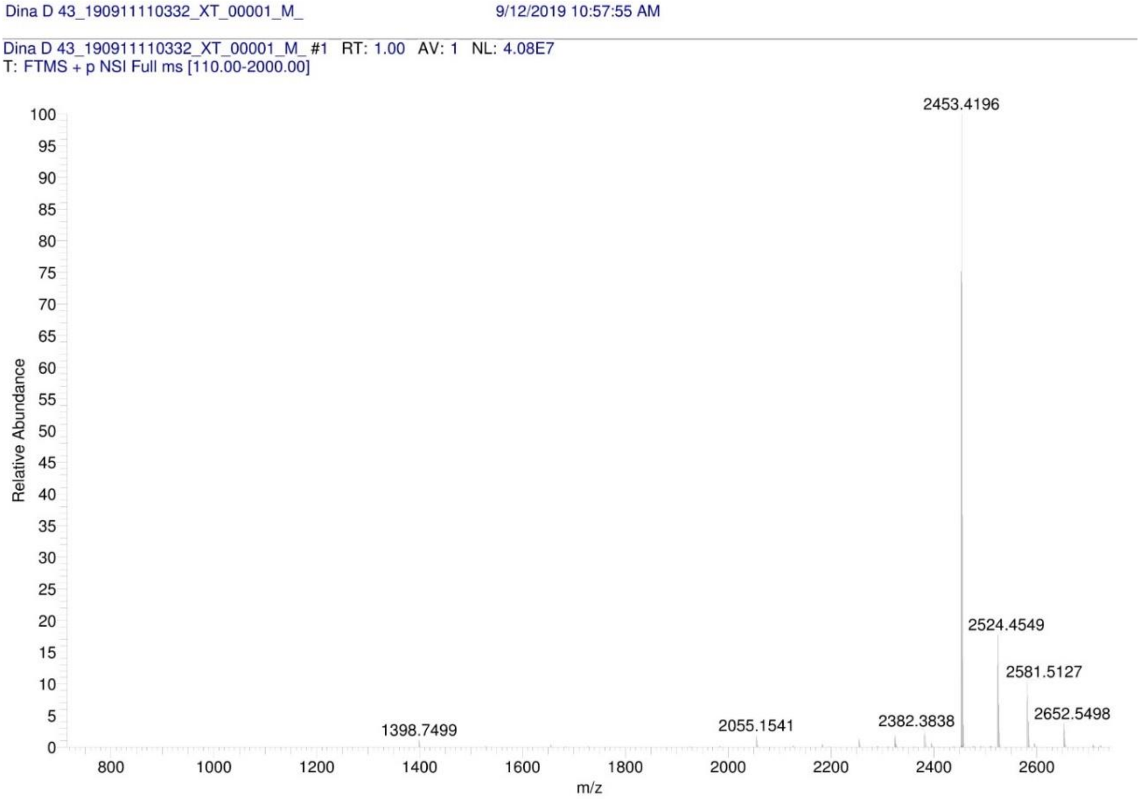
Dina D 43_190911110332_XT_00001_M_
9/12/2019 10:57:55 AM
Dina D 43_190911110332_XT_00001_M_ #1 RT: 1.00 AV: 1 NL: 4.08E7
T: FTMS + p NSI Full ms [110.00-2000.00]
2453.4196
2524.4549
2581.5127
2652.5498
2382.3838
2055.1541
1398.7499
Relative Abundance
m/z

Dina D 43_190911110332_XT_00001_M_ 9/12/2019 10:57:55 AM

Dina D 43_190911110332_XT_00001_M_ #1 RT: 1.00 AV: 1 NL: 4.08E7
T: FTMS + p NSI Full ms [110.00-2000.00]

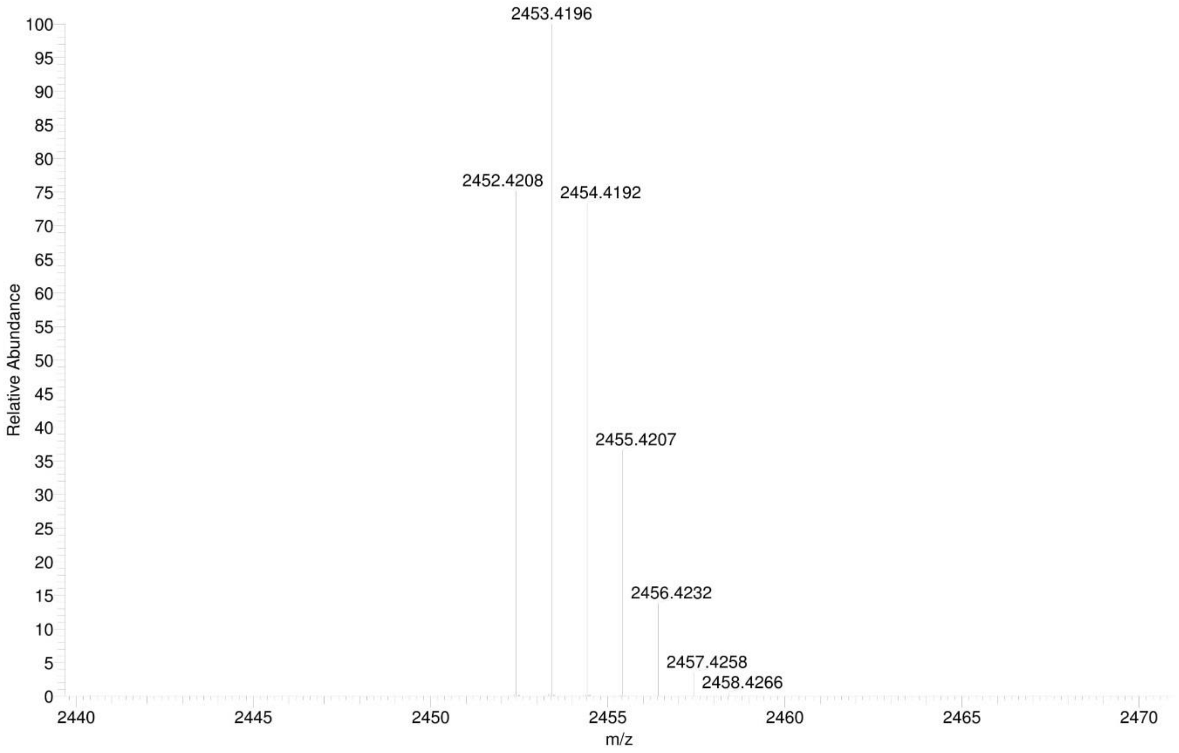

Ac**11 ((AcAKA)$_4$(*K*EK)$_2$*K*YEY-OH)** was obtained from the CEM Liberty Blue synthetiser as foamy colourless solid after preparative RP-HPLC (25.0 mg, 6.4 µmol, 21.0%). Analytical RP-HPLC: t_R=1.31 min (100% A to 100% D in 5 min, λ= 214 nm). HRMS (ESI+): $C_{119}H_{197}N_{31}O_{35}$ calc./obs. 2620.4588/2620.4627 Da [M].

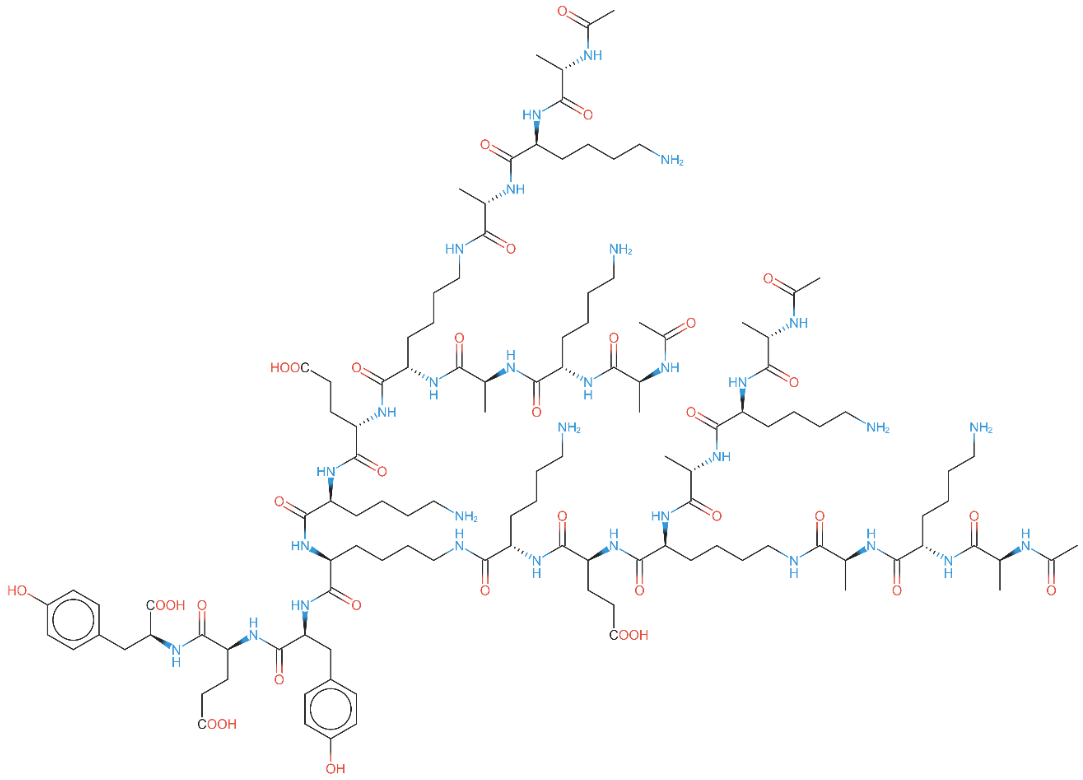

Analytical RP-HPLC:

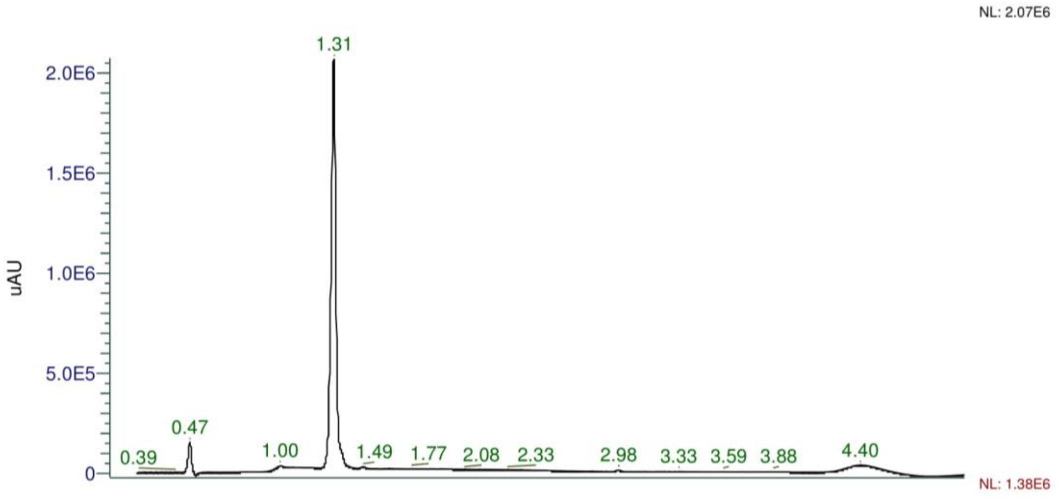

HRMS (NSI+):

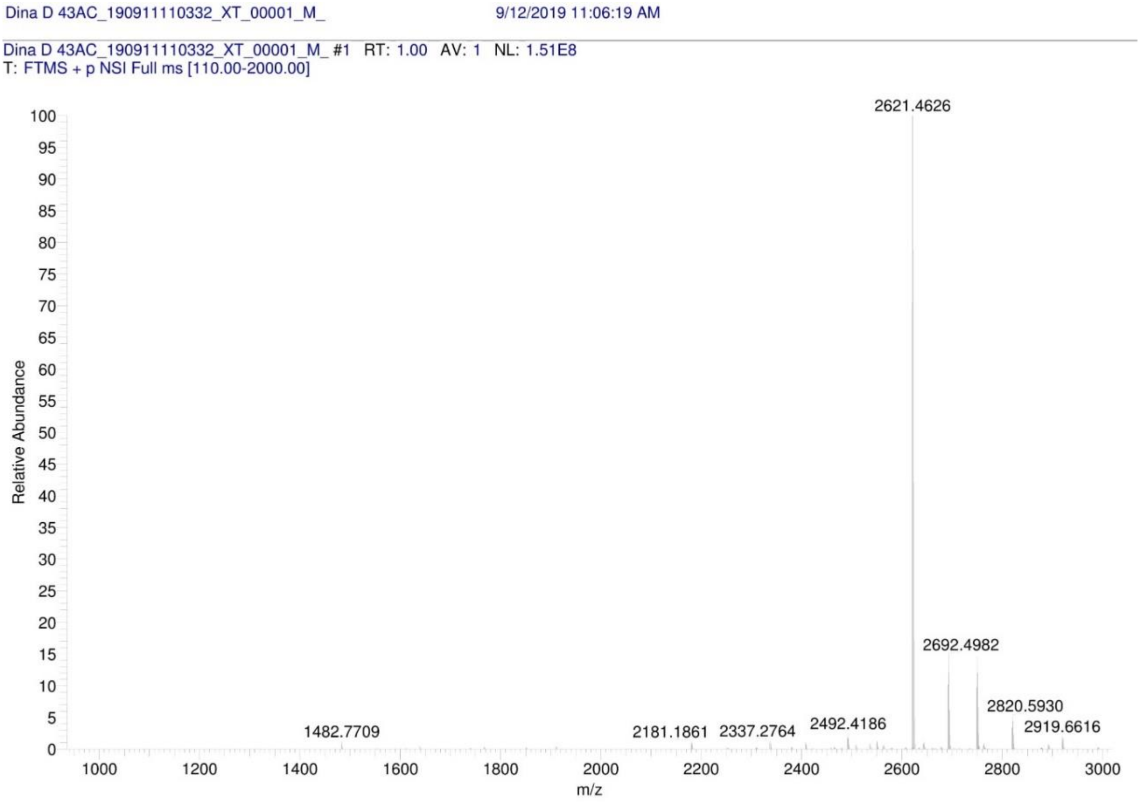
Dina D 43AC_190911110332_XT_00001_M_
9/12/2019 11:06:19 AM
Dina D 43AC_190911110332_XT_00001_M_ #1 RT: 1.00 AV: 1 NL: 1.51E8
T: FTMS + p NSI Full ms [110.00-2000.00]
2621.4626
2692.4982
2820.5930
2919.6616
2492.4186
2337.2764
2181.1861
1482.7709
Relative Abundance
m/z

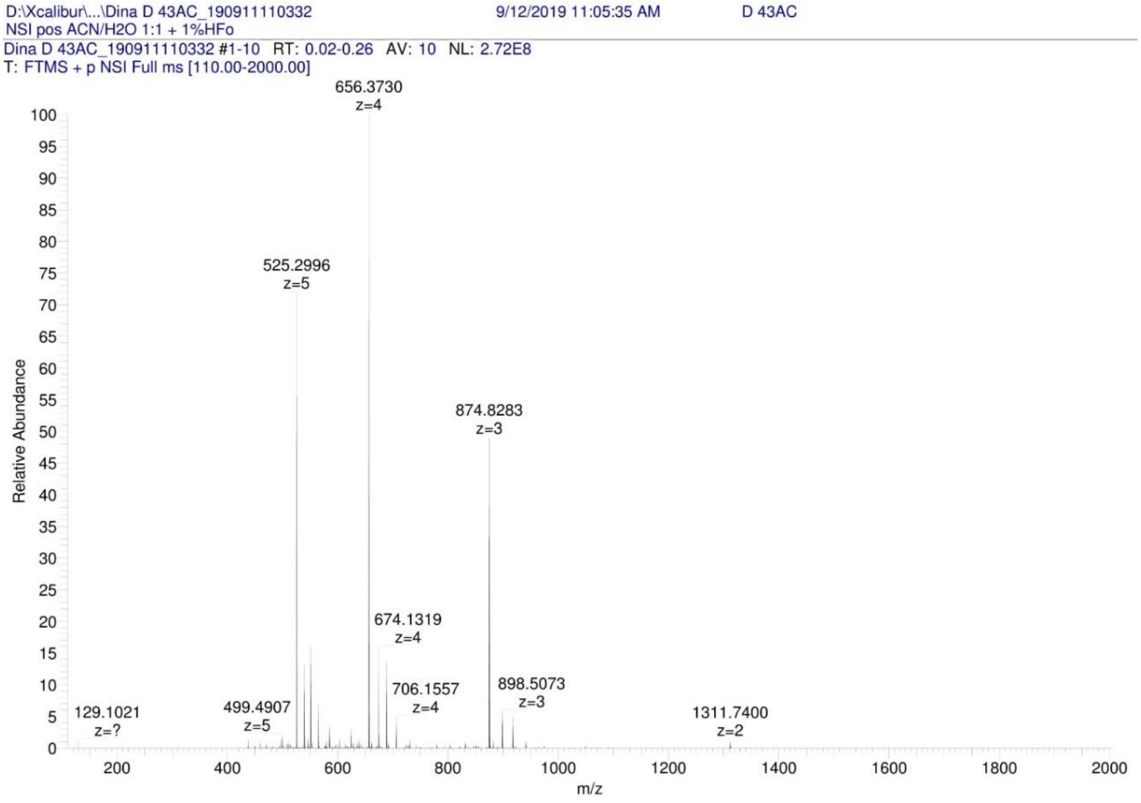
D:\Xcalibur\...\Dina D 43AC_190911110332
9/12/2019 11:05:35 AM
D 43AC
NSI pos ACN/H2O 1:1 + 1%HFo
Dina D 43AC_190911110332 #1-10 RT: 0.02-0.26 AV: 10 NL: 2.72E8
T: FTMS + p NSI Full ms [110.00-2000.00]
656.3730
z=4
525.2996
z=5
874.8283
z=3
674.1319
z=4
706.1557
z=4
898.5073
z=3
499.4907
z=5
129.1021
z=?
1311.7400
z=2
Relative Abundance
m/z

Dina D 43AC_190911110332_XT_00001_M_ 9/12/2019 11:06:19 AM

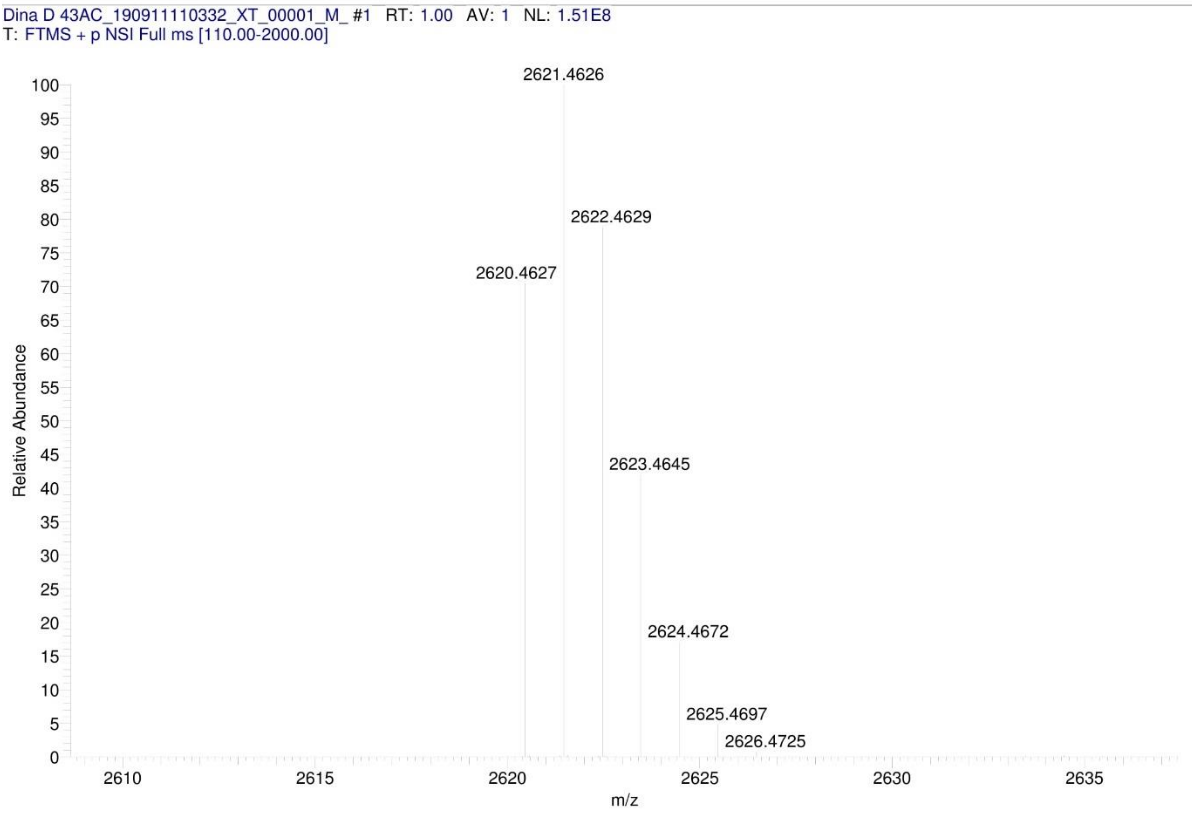

ClAc**11** **((ClAcAKA)$_4$(*K*EK)$_2$*K*YEY-OH)** was obtained from the CEM Liberty Blue synthetiser as foamy colourless solid after preparative RP-HPLC (10.2 mg, 2.3 µmol, 8.2%). Analytical RP-HPLC: t_R=1.39 min (100% A to 100% D in 5 min, λ= 214 nm). HRMS (ESI+): $C_{119}H_{193}Cl_4N_{31}O_{35}$ calc./obs. 2756.3029/2756.3050 Da [M].

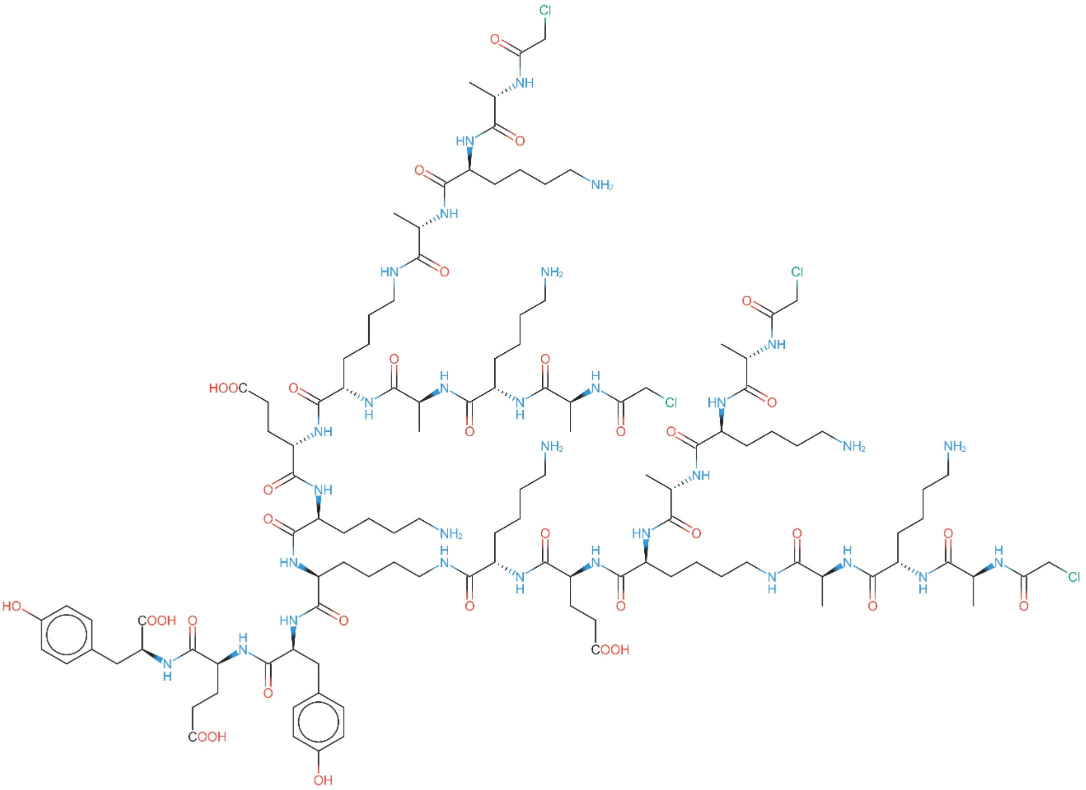

Analytical RP-HPLC:

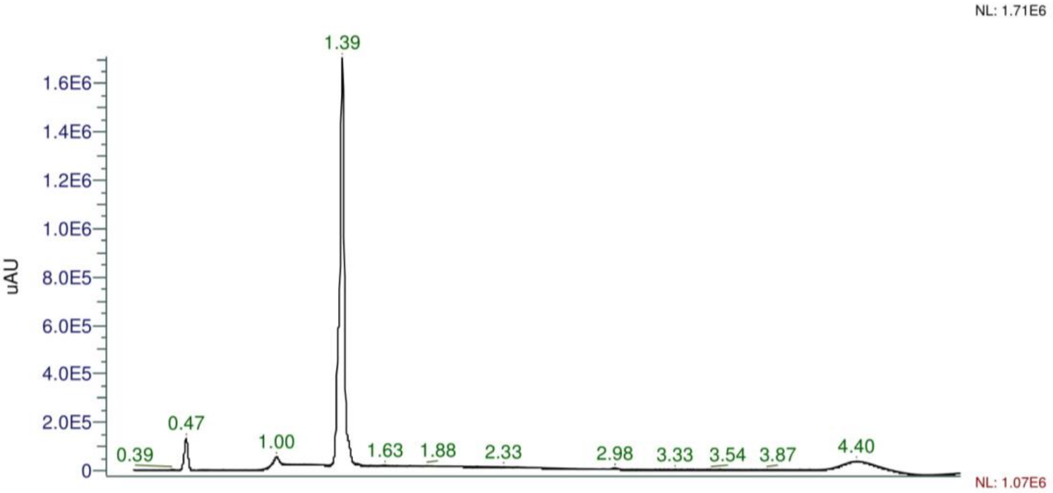

HRMS (NSI+):

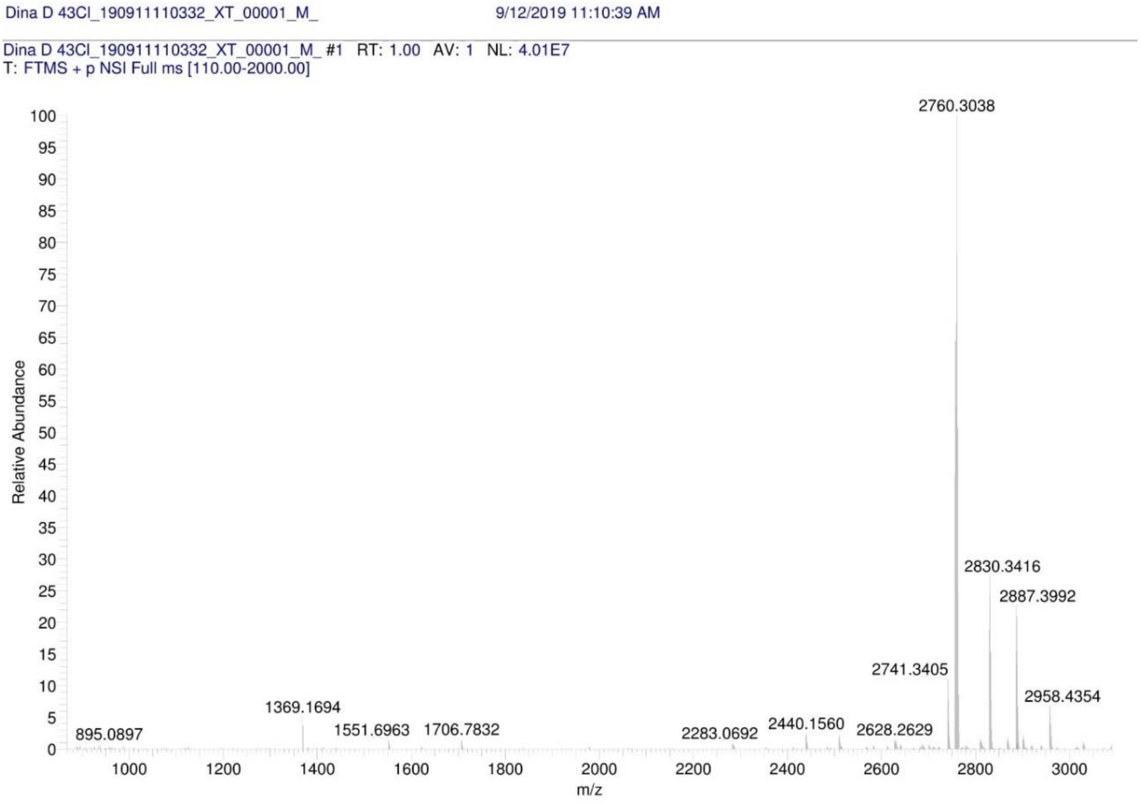
Dina D 43Cl_190911110332_XT_00001_M_
9/12/2019 11:10:39 AM
Dina D 43Cl_190911110332_XT_00001_M_ #1 RT: 1.00 AV: 1 NL: 4.01E7
T: FTMS + p NSI Full ms [110.00-2000.00]
2760.3038
2830.3416
2887.3992
2741.3405
2958.4354
1369.1694
895.0897
1551.6963
1706.7832
2283.0692
2440.1560
2628.2629
Relative Abundance
m/z

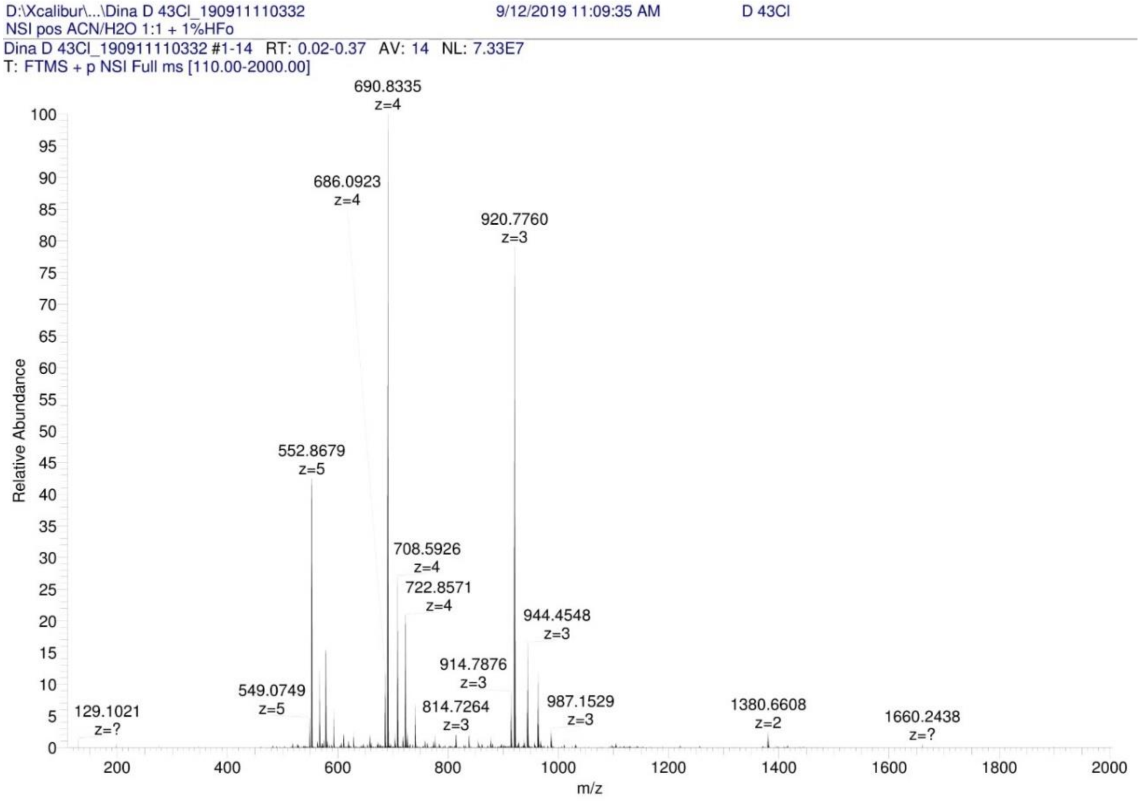
D:\Xcalibur\...\Dina D 43Cl_190911110332
9/12/2019 11:09:35 AM
D 43Cl
NSI pos ACN/H2O 1:1 + 1%HFo
Dina D 43Cl_190911110332 #1-14 RT: 0.02-0.37 AV: 14 NL: 7.33E7
T: FTMS + p NSI Full ms [110.00-2000.00]
690.8335 z=4
686.0923 z=4
920.7760 z=3
552.8679 z=5
708.5926 z=4
722.8571 z=4
944.4548 z=3
914.7876 z=3
549.0749 z=5
987.1529 z=3
814.7264 z=3
129.1021 z=?
1380.6608 z=2
1660.2438 z=?
Relative Abundance
m/z

Dina D 43Cl_190911110332_XT_00001_M_ 9/12/2019 11:10:39 AM

Dina D 43Cl_190911110332_XT_00001_M_ #1 RT: 1.00 AV: 1 NL: 4.01E7
T: FTMS + p NSI Full ms [110.00-2000.00]

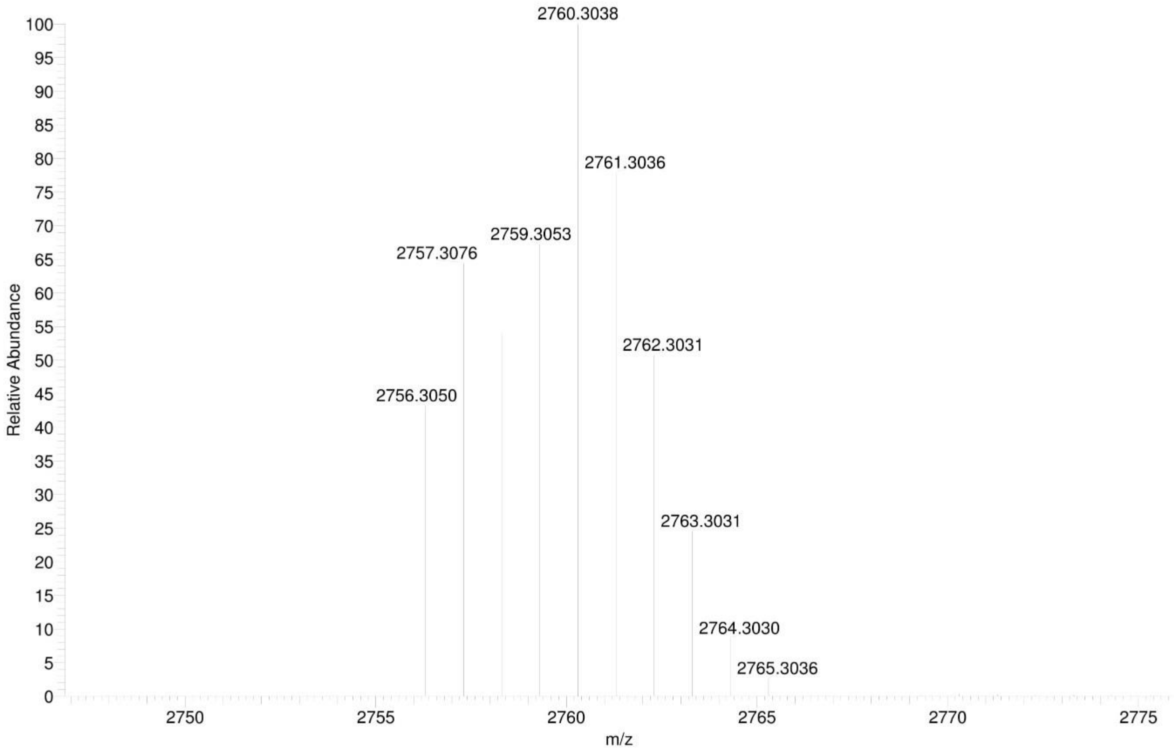

12 ((KAA)$_4$(*K*EKA)$_2$*K*EKA-NH$_2$) was obtained from the CEM Liberty Blue synthetiser as foamy colourless solid after preparative RP-HPLC (169.5 mg, 12.6 µmol, 49.3%). Analytical RP-HPLC: t_R=1.21 min (100% A to 100% D in 5 min, λ= 214 nm). HRMS (ESI+): $C_{120}H_{207}Cl_4N_{35}O_{32}$ calc./obs. 2650.5646/2650.5679 Da [M].

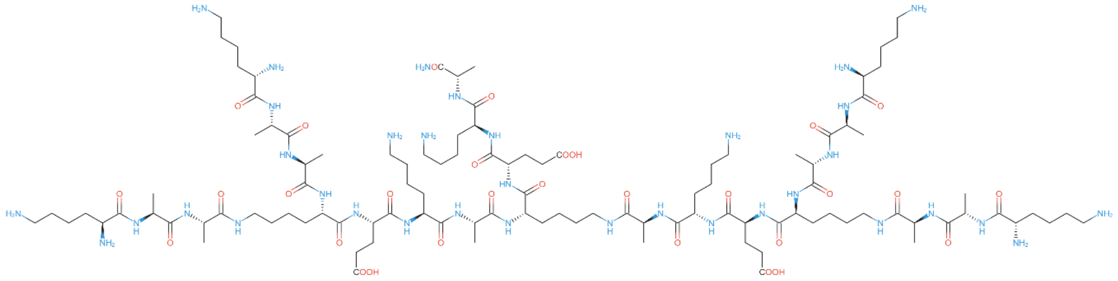

Analytical RP-HPLC:

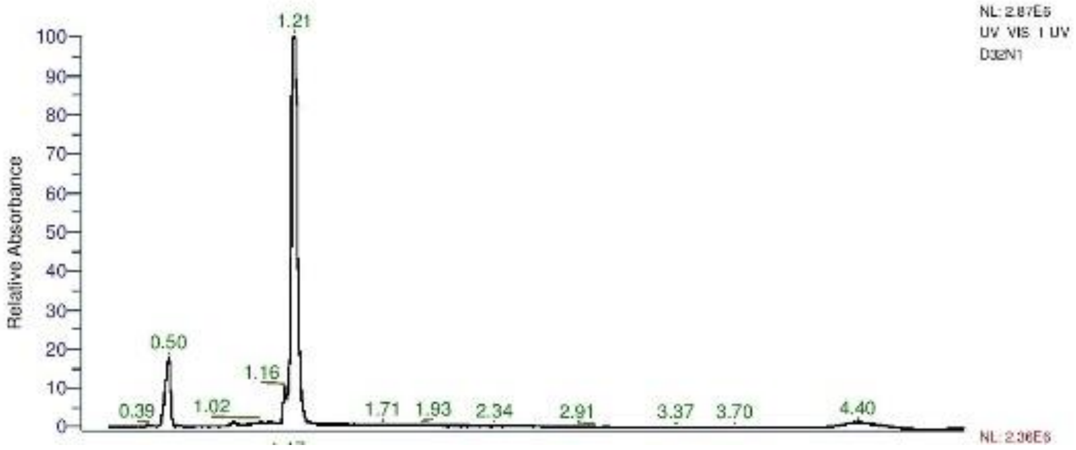

HRMS (NSI+):

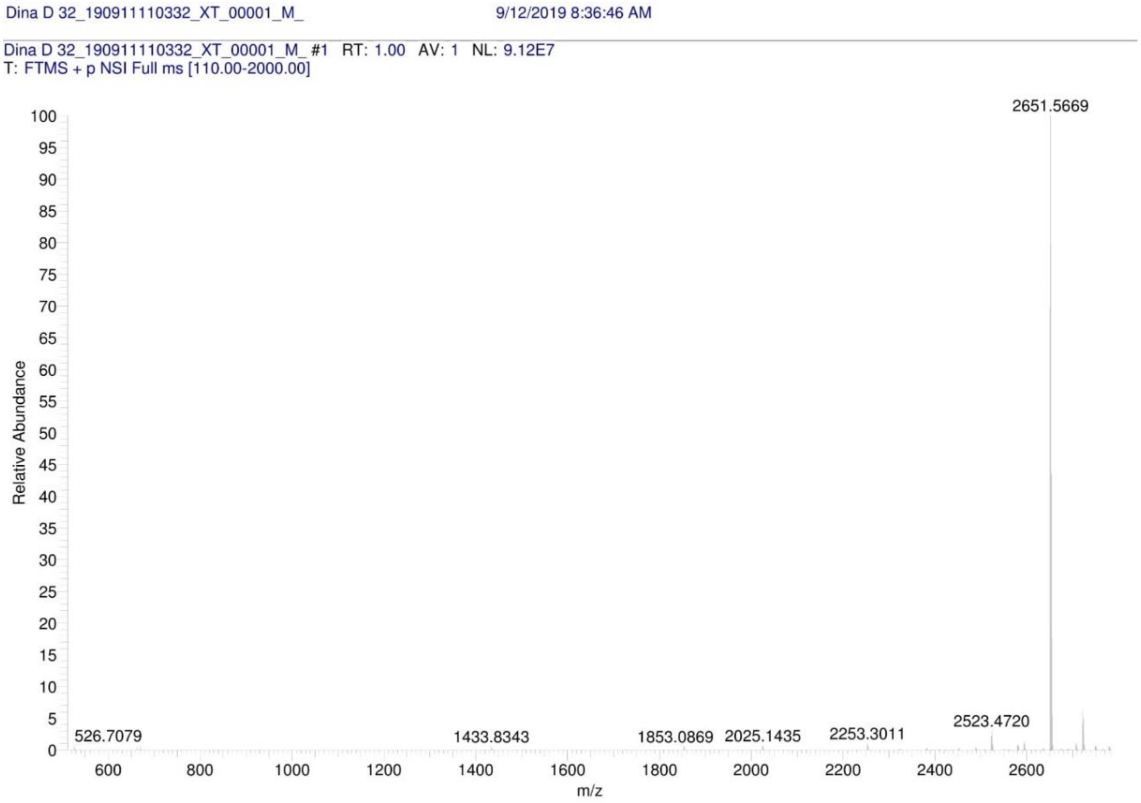
Dina D 32_190911110332_XT_00001_M_
9/12/2019 8:36:46 AM
Dina D 32_190911110332_XT_00001_M_ #1 RT: 1.00 AV: 1 NL: 9.12E7
T: FTMS + p NSI Full ms [110.00-2000.00]
2651.5669
526.7079
1433.8343
1853.0869
2025.1435
2253.3011
2523.4720
Relative Abundance
m/z

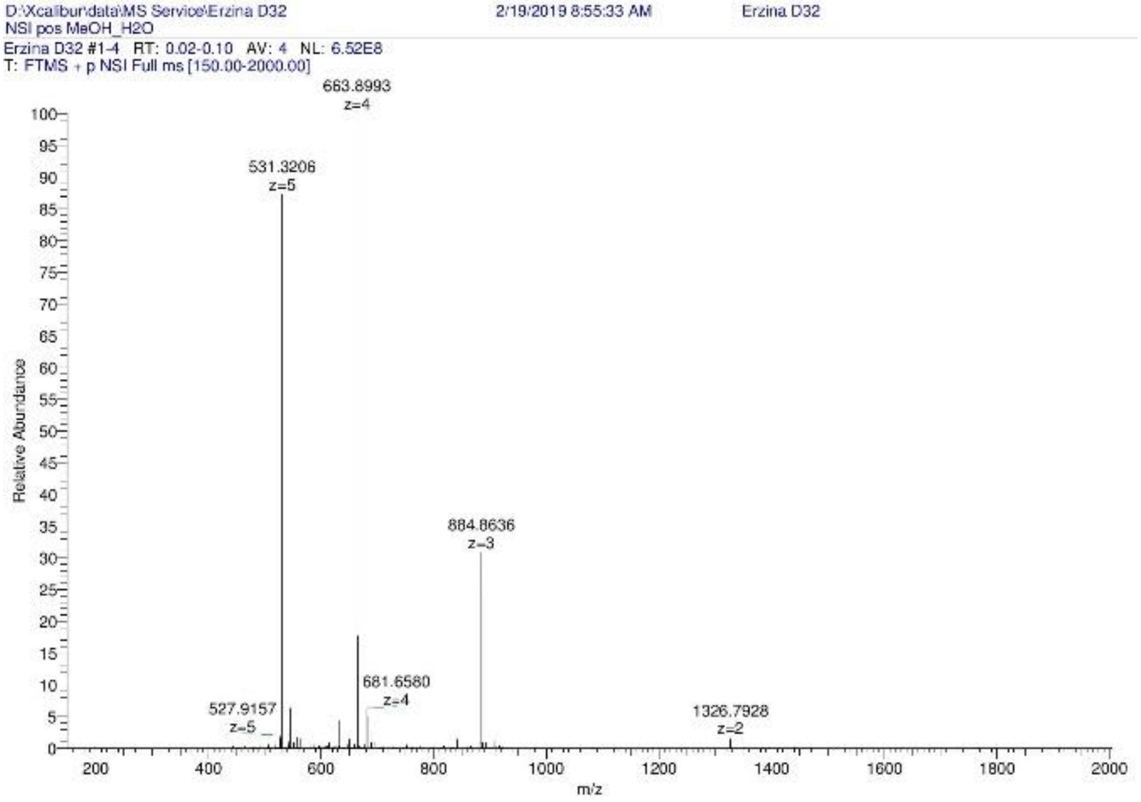
D:\Xcalibur\data\MS Service\Erzina D32
NSI pos MeOH_H2O
2/19/2019 8:55:33 AM
Erzina D32
Erzina D32 #1-4 RT: 0.02-0.10 AV: 4 NL: 6.52E8
T: FTMS + p NSI Full ms [150.00-2000.00]
663.8993
z=4
531.3206
z=5
884.8636
z=3
681.6580
z=4
527.9157
z=5
1326.7928
z=2
Relative Abundance
m/z

Dina D 32_190911110332_XT_00001_M_ 9/12/2019 8:36:46 AM

Dina D 32_190911110332_XT_00001_M_ #1 RT: 1.00 AV: 1 NL: 9.12E7
T: FTMS + p NSI Full ms [110.00-2000.00]

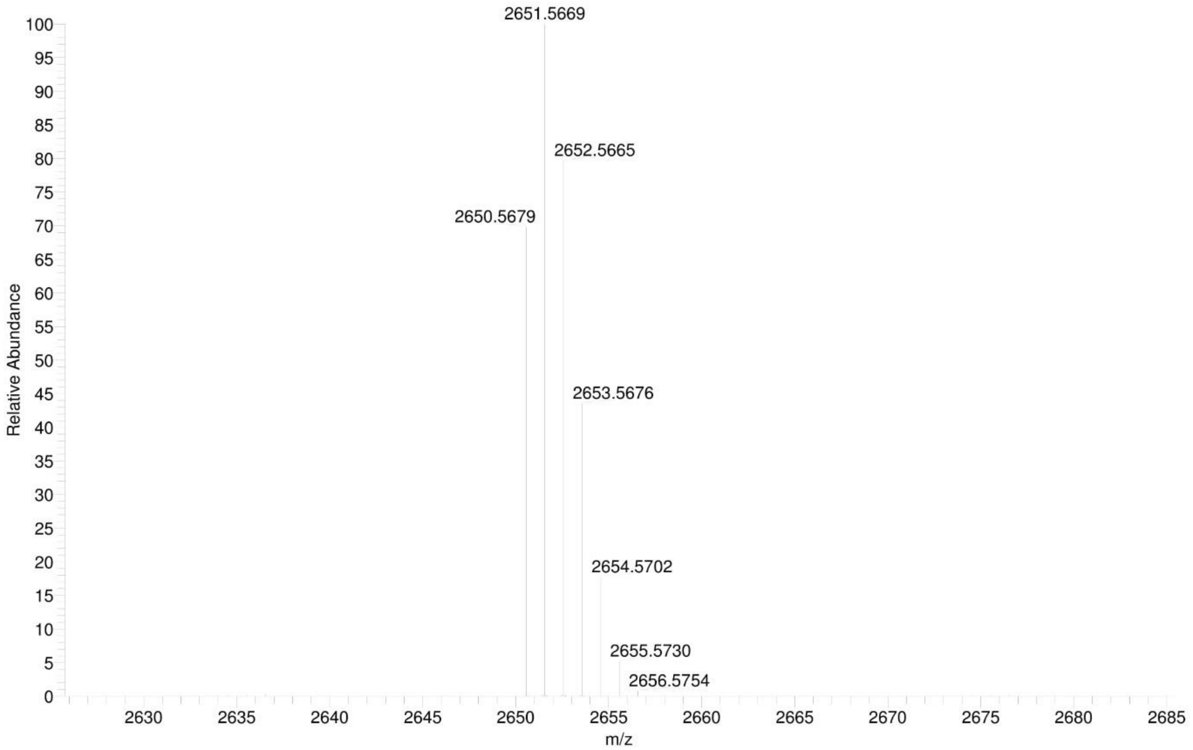

ClAc**13 ((ClAcK)$_4$(*K*AYE)$_2$*K*AKYAEA-NH$_2$)** was obtained from the CEM Liberty Blue synthetiser as foamy colourless solid after preparative RP-HPLC (159.6 mg, 14.6 µmol, 46.1%). Analytical RP-HPLC: t_R=1.44 min (100% A to 100% D in 5 min, λ= 214 nm). HRMS (ESI+): $C_{113}H_{178}Cl_4N_{30}O_{30}$ calc./obs. 2575.2079/2577.1828 Da [M].

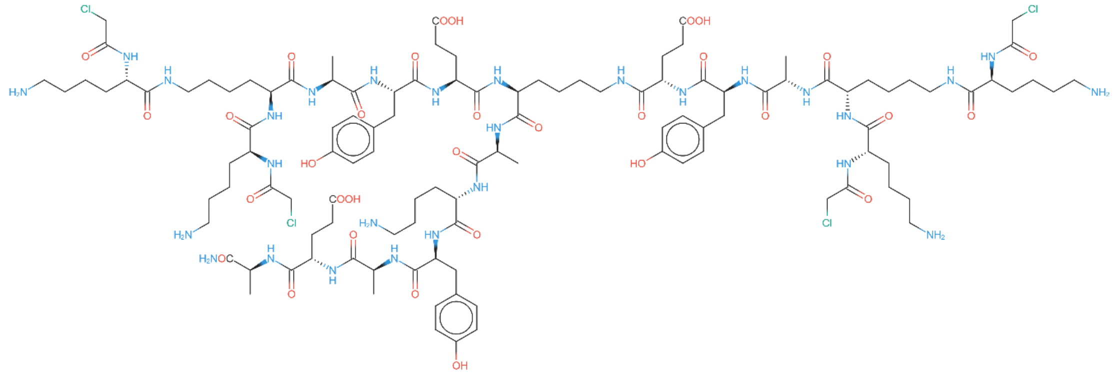

Analytical RP-HPLC:

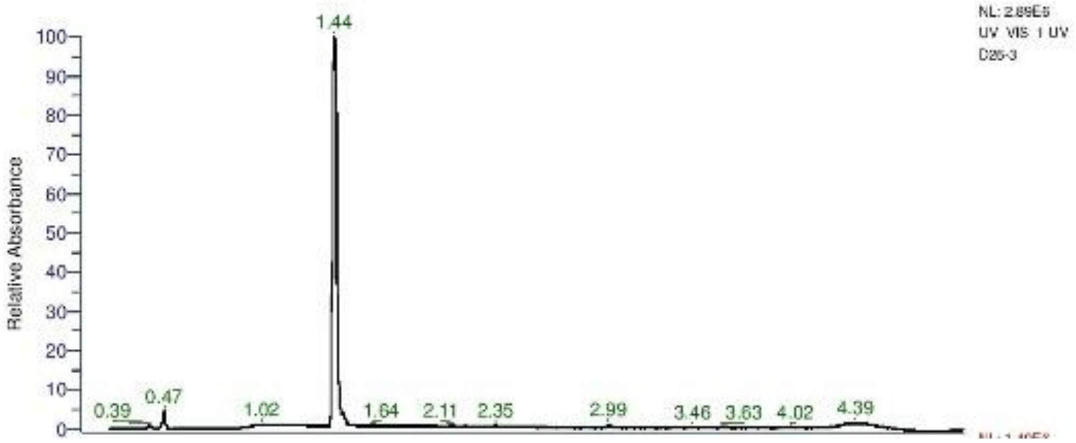

HRMS (NSI+):

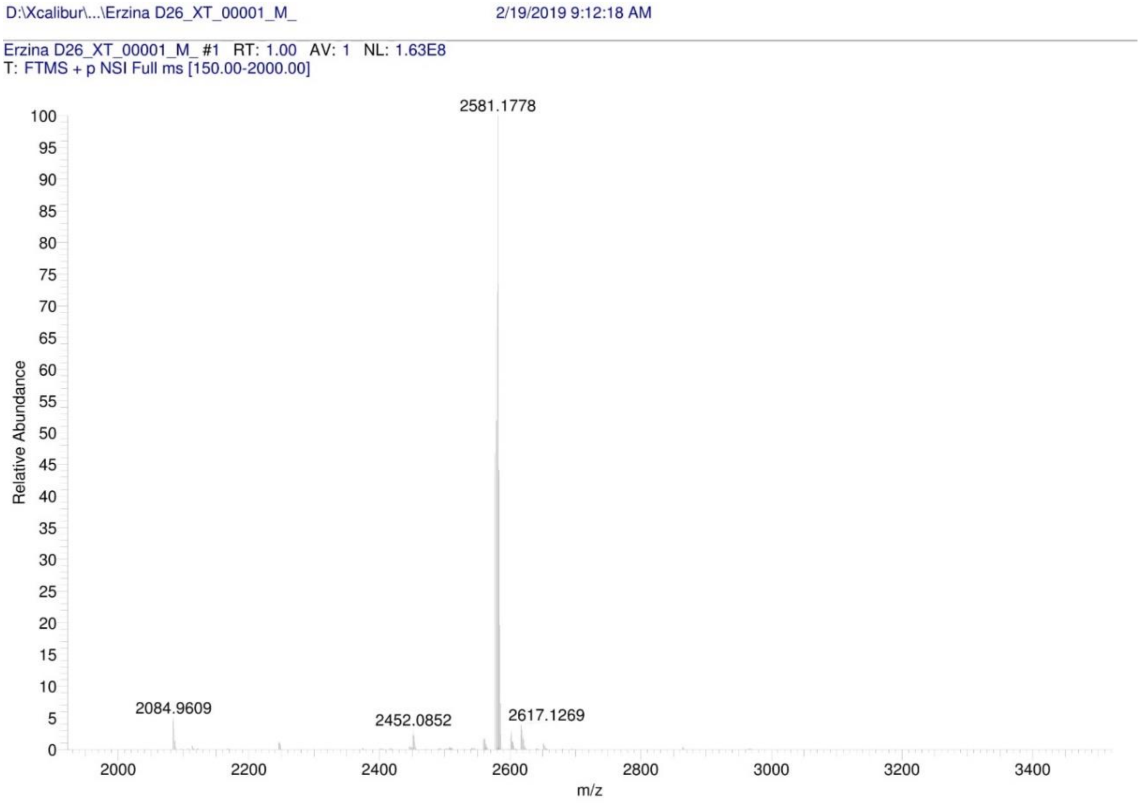

D:\Xcalibur\...\Erzina D26_XT_00001_M_
2/19/2019 9:12:18 AM
Erzina D26_XT_00001_M_ #1 RT: 1.00 AV: 1 NL: 1.63E8
T: FTMS + p NSI Full ms [150.00-2000.00]
2581.1778
2084.9609
2452.0852
2617.1269
Relative Abundance
m/z

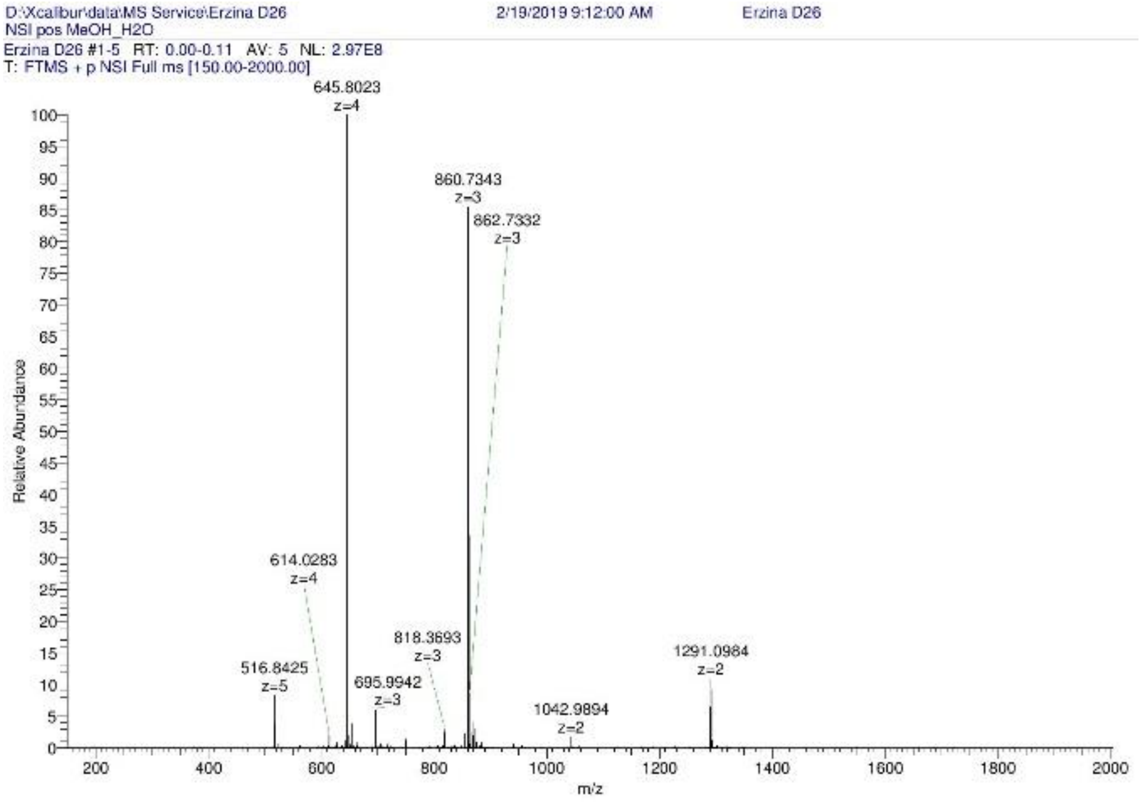

D:\Xcalibur\data\MS Service\Erzina D26
2/19/2019 9:12:00 AM
Erzina D26
NSI pos MeOH_H2O
Erzina D26 #1-5 RT: 0.00-0.11 AV: 5 NL: 2.97E8
T: FTMS + p NSI Full ms [150.00-2000.00]
645.8023 z=4
860.7343 z=3
862.7332 z=3
614.0283 z=4
818.3693 z=3
516.8425 z=5
695.9942 z=3
1291.0984 z=2
1042.9894 z=2
Relative Abundance
m/z

Dina D 26_190911110332_XT_00001_M_ 9/12/2019 8:03:05 AM

Dina D 26_190911110332_XT_00001_M_ #1 RT: 1.00 AV: 1 NL: 1.65E8
T: FTMS + p NSI Full ms [110.00-2000.00]

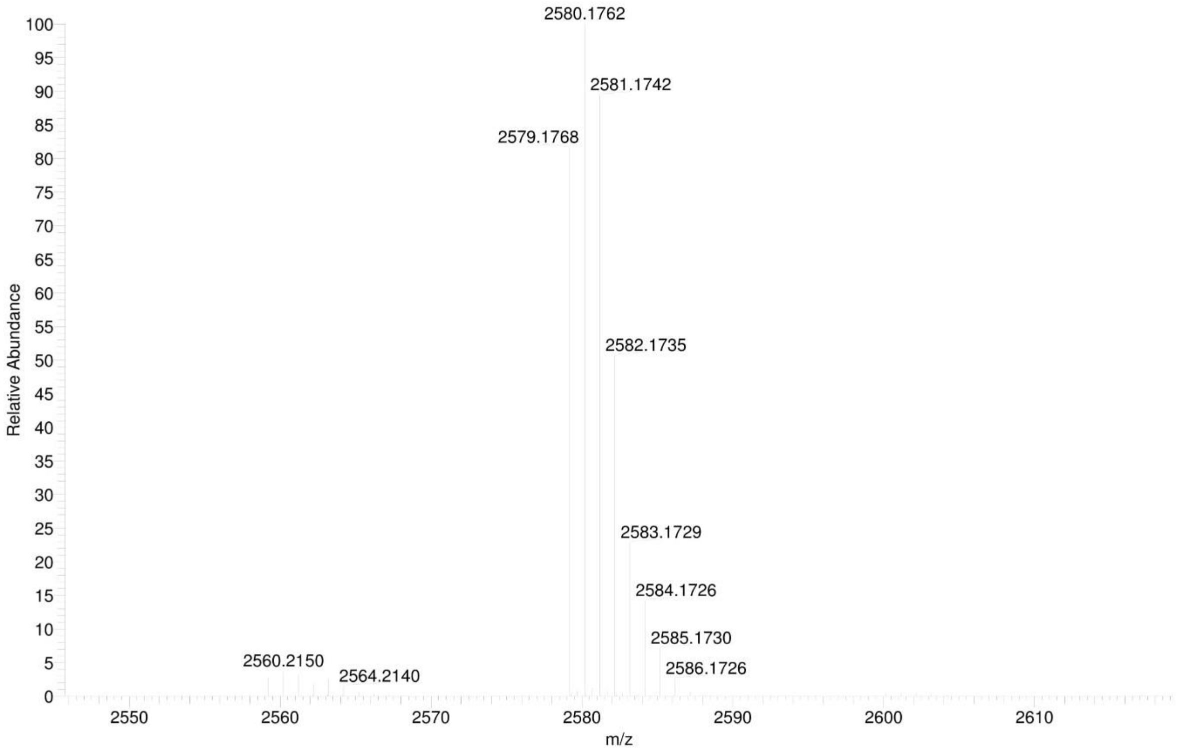

14 ((KYA)$_4$(*K*EA)$_2$*K*KKA-OH) was obtained after manual synthesis as foamy colourless solid after preparative RP-HPLC (87.6 mg, 10.6 µmol, 39.2%). Analytical RP-HPLC: t_R=1.24 min (100% A to 100% D in 5 min, λ= 214 nm). HRMS (ESI+): $C_{121}H_{195}N_{31}O_{301}$ calc./obs. 2578.4635/2578.4656 Da [M].

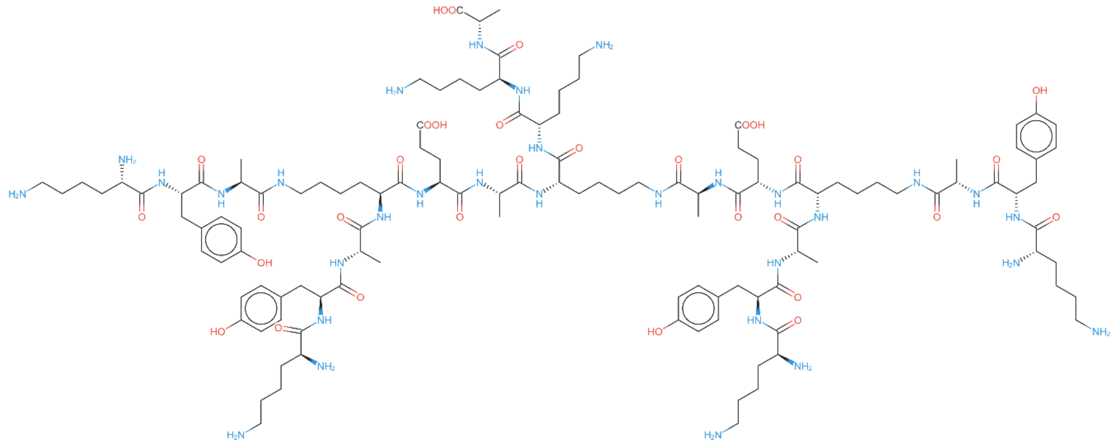

Analytical RP-HPLC:

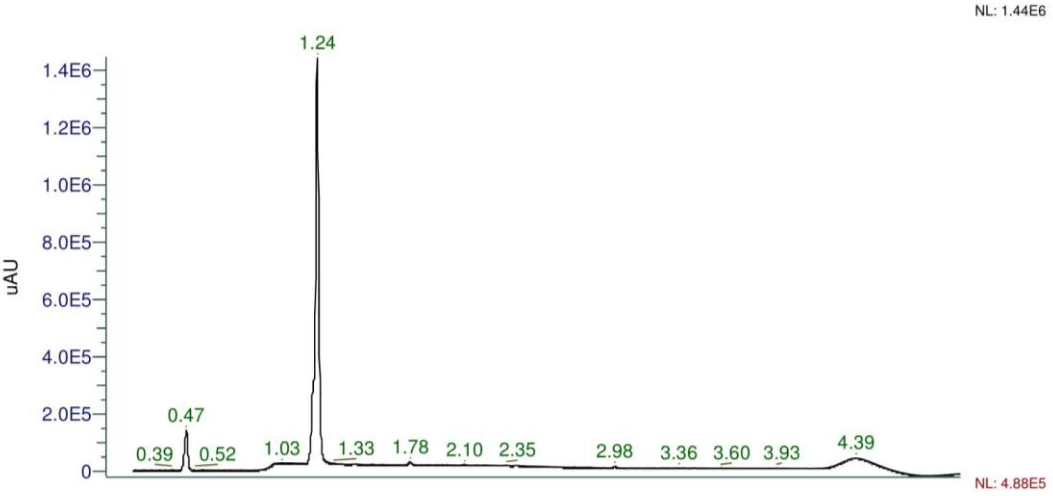

HRMS (NSI+):

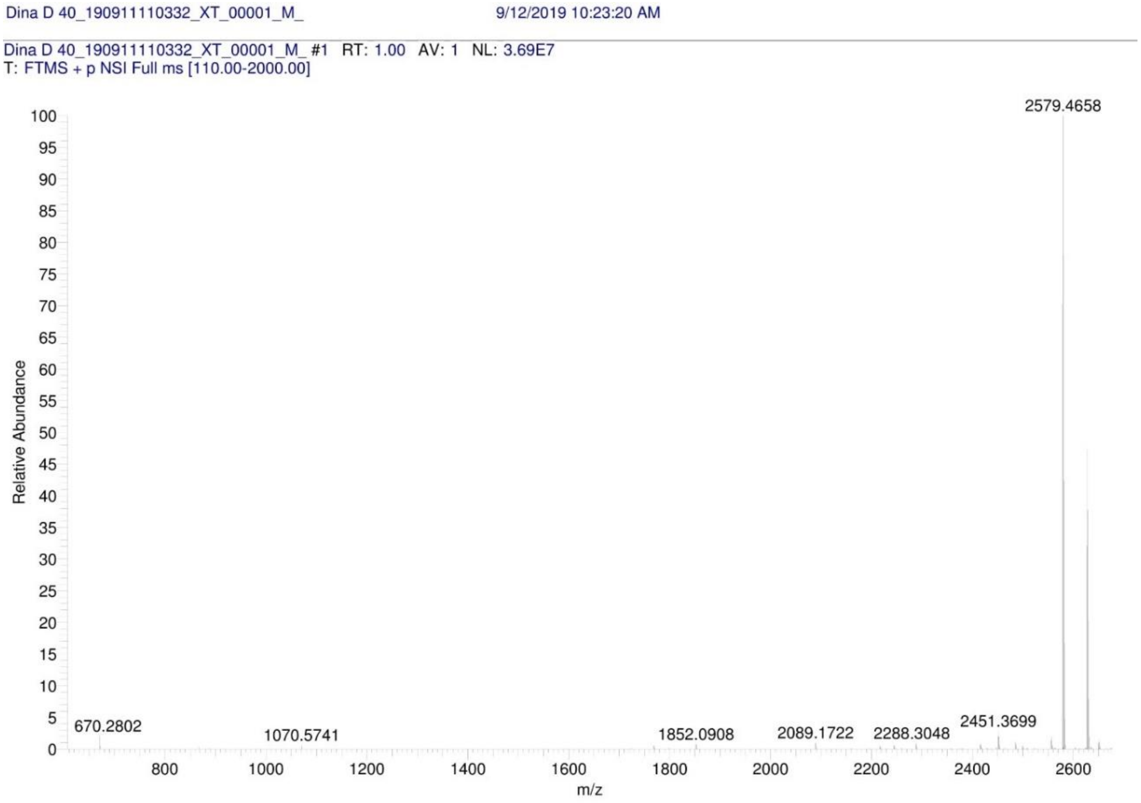
Dina D 40_190911110332_XT_00001_M_
9/12/2019 10:23:20 AM
Dina D 40_190911110332_XT_00001_M_ #1 RT: 1.00 AV: 1 NL: 3.69E7
T: FTMS + p NSI Full ms [110.00-2000.00]
Relative Abundance
m/z
2579.4658
670.2802
1070.5741
1852.0908
2089.1722
2288.3048
2451.3699

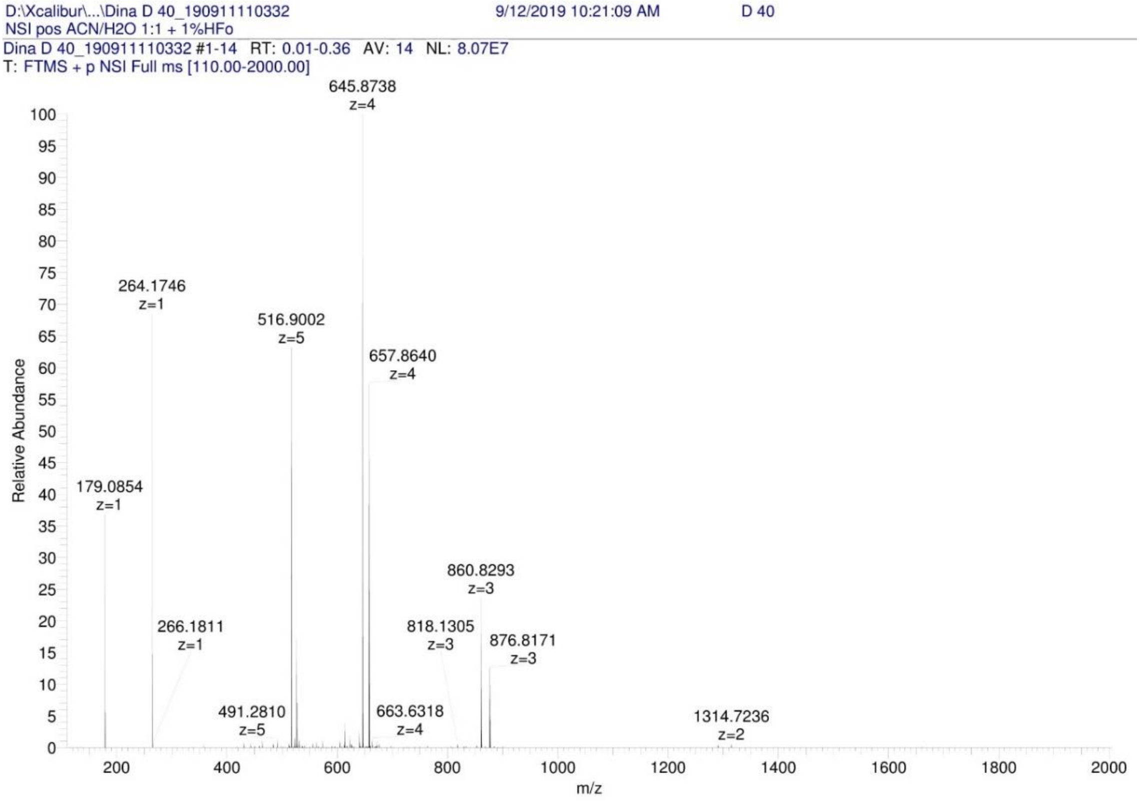
D:\Xcalibur\...\Dina D 40_190911110332
9/12/2019 10:21:09 AM
D 40
NSI pos ACN/H2O 1:1 + 1%HFo
Dina D 40_190911110332 #1-14 RT: 0.01-0.36 AV: 14 NL: 8.07E7
T: FTMS + p NSI Full ms [110.00-2000.00]
Relative Abundance
m/z
645.8738 z=4
264.1746 z=1
516.9002 z=5
657.8640 z=4
179.0854 z=1
860.8293 z=3
266.1811 z=1
818.1305 z=3
876.8171 z=3
491.2810 z=5
663.6318 z=4
1314.7236 z=2

Dina D 40_190911110332_XT_00001_M_ 9/12/2019 10:23:20 AM

Dina D 40_190911110332_XT_00001_M_ #1 RT: 1.00 AV: 1 NL: 3.69E7
T: FTMS + p NSI Full ms [110.00-2000.00]

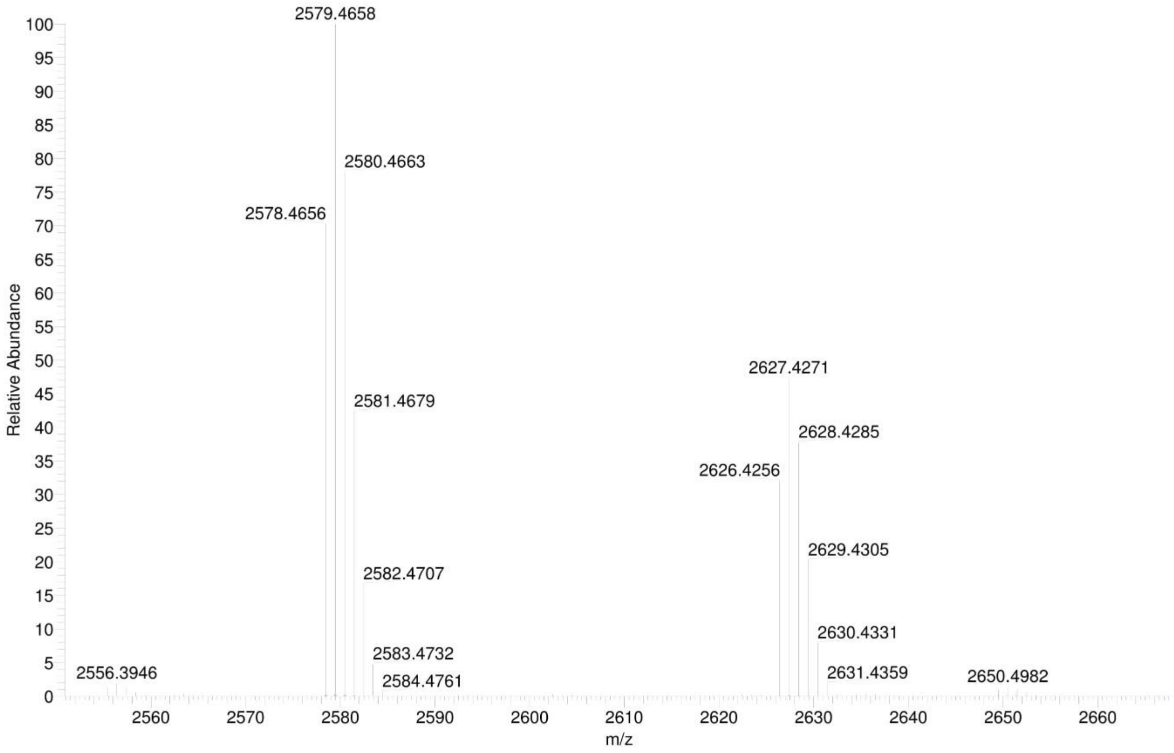

Ac**14** **((AcKYA)$_4$(*K*EA)$_2$*K*KKA-OH)** was obtained after manual synthesis as foamy colourless solid after preparative RP-HPLC (88.0 mg, 12.5 µmol, 42.7%). Analytical RP-HPLC: t_R=1.19 min (100% A to 100% D in 5 min, λ= 214 nm). HRMS (ESI+): $C_{129}H_{203}N_{31}O_{35}$ calc./obs. 2746.5058/2746.5094 Da [M].

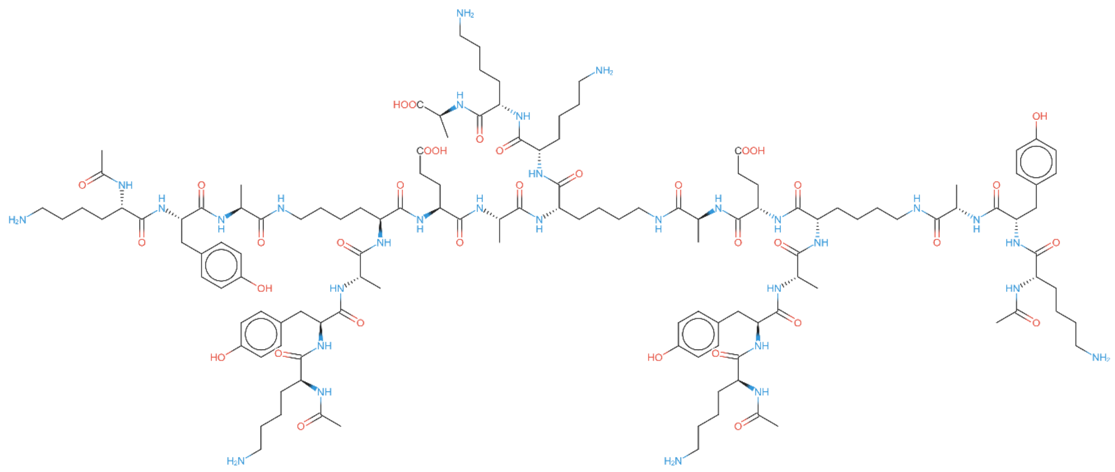

Analytical RP-HPLC:

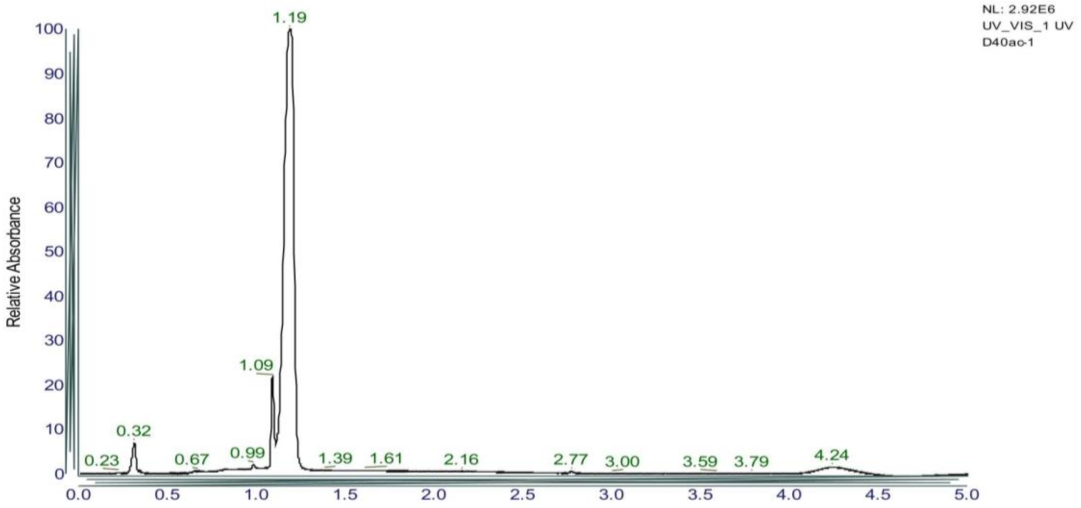

HRMS (NSI+):

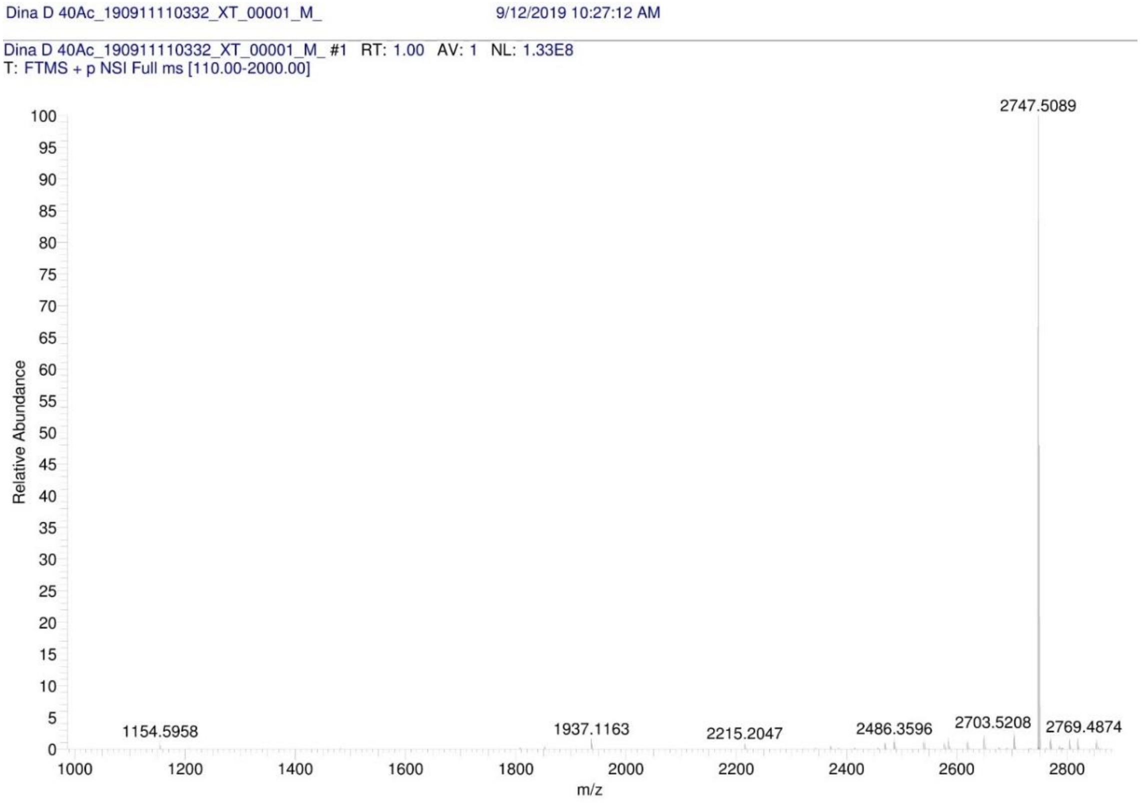
Dina D 40Ac_190911110332_XT_00001_M_ 9/12/2019 10:27:12 AM
Dina D 40Ac_190911110332_XT_00001_M_ #1 RT: 1.00 AV: 1 NL: 1.33E8
T: FTMS + p NSI Full ms [110.00-2000.00]
2747.5089
1154.5958
1937.1163
2215.2047
2486.3596
2703.5208
2769.4874
Relative Abundance
m/z

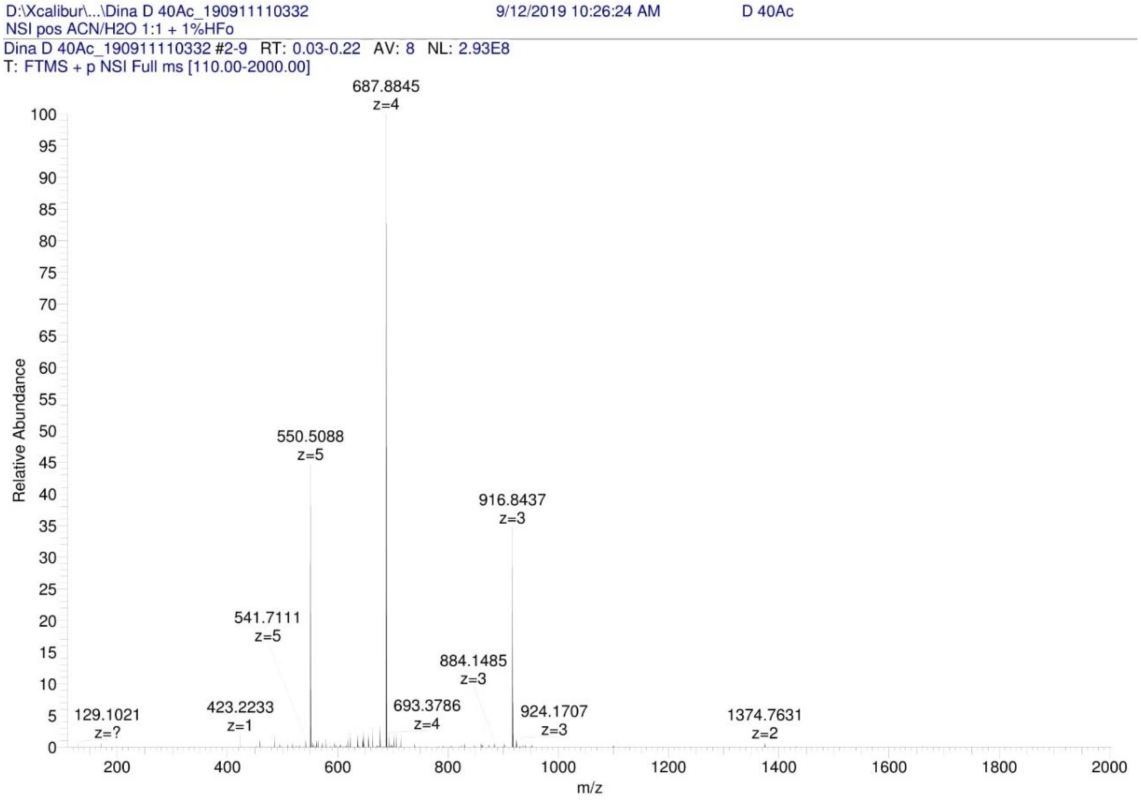
D:\Xcalibur\...\Dina D 40Ac_190911110332 9/12/2019 10:26:24 AM D 40Ac
NSI pos ACN/H2O 1:1 + 1%HFo
Dina D 40Ac_190911110332 #2-9 RT: 0.03-0.22 AV: 8 NL: 2.93E8
T: FTMS + p NSI Full ms [110.00-2000.00]
687.8845 z=4
550.5088 z=5
916.8437 z=3
541.7111 z=5
884.1485 z=3
129.1021 z=?
423.2233 z=1
693.3786 z=4
924.1707 z=3
1374.7631 z=2
Relative Abundance
m/z

Dina D 40Ac_190911110332_XT_00001_M_ 9/12/2019 10:27:12 AM

Dina D 40Ac_190911110332_XT_00001_M_ #1 RT: 1.00 AV: 1 NL: 1.33E8
T: FTMS + p NSI Full ms [110.00-2000.00]

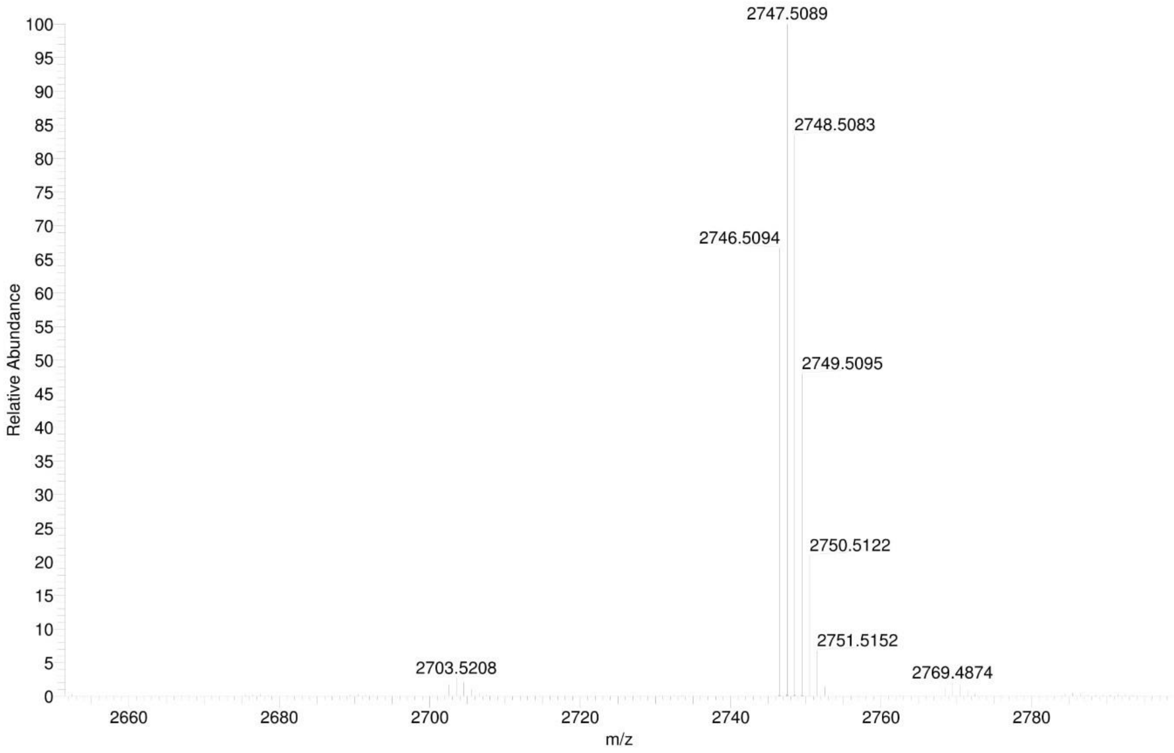

ClAc**14 ((ClAcKYA)$_4$(*K*EA)$_2$*K*KKA-OH)** was obtained after manual synthesis as foamy colourless solid after preparative RP-HPLC (65.3 mg, 8.5 µmol, 30.5%). Analytical RP-HPLC: t_R=1.42 min (100% A to 100% D in 5 min, λ= 214 nm). HRMS (ESI+): $C_{129}H_{199}Cl_4N_{31}O_{35}$ calc./obs. 2882.3499/2882.3549 Da [M].

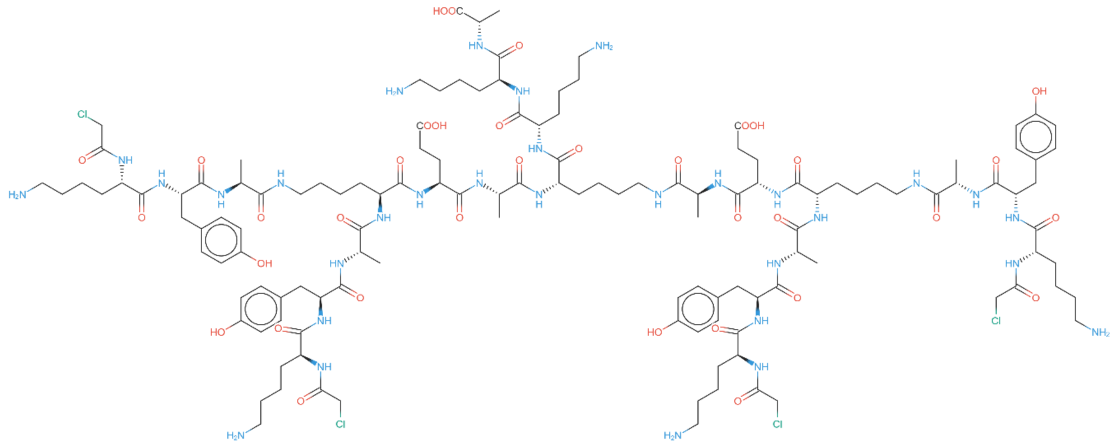

Analytical RP-HPLC:

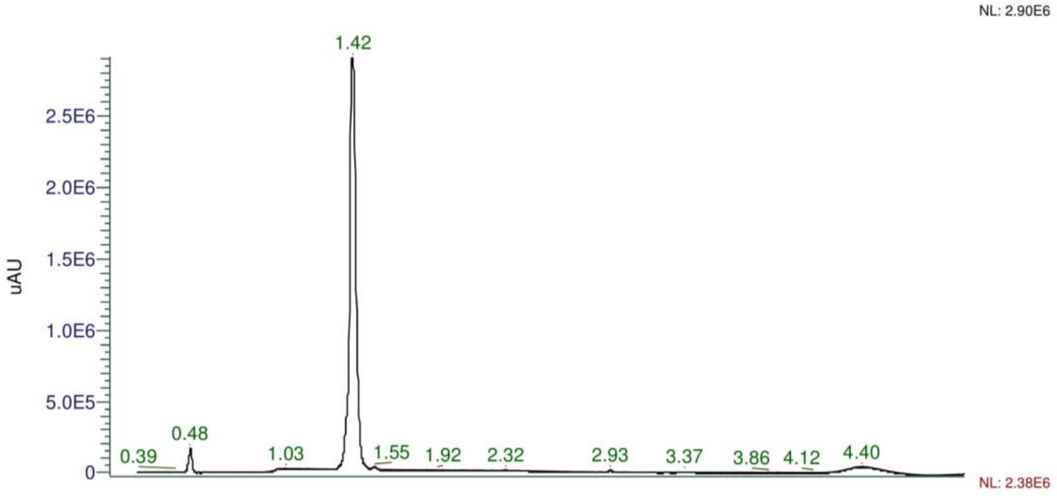

HRMS (NSI+):

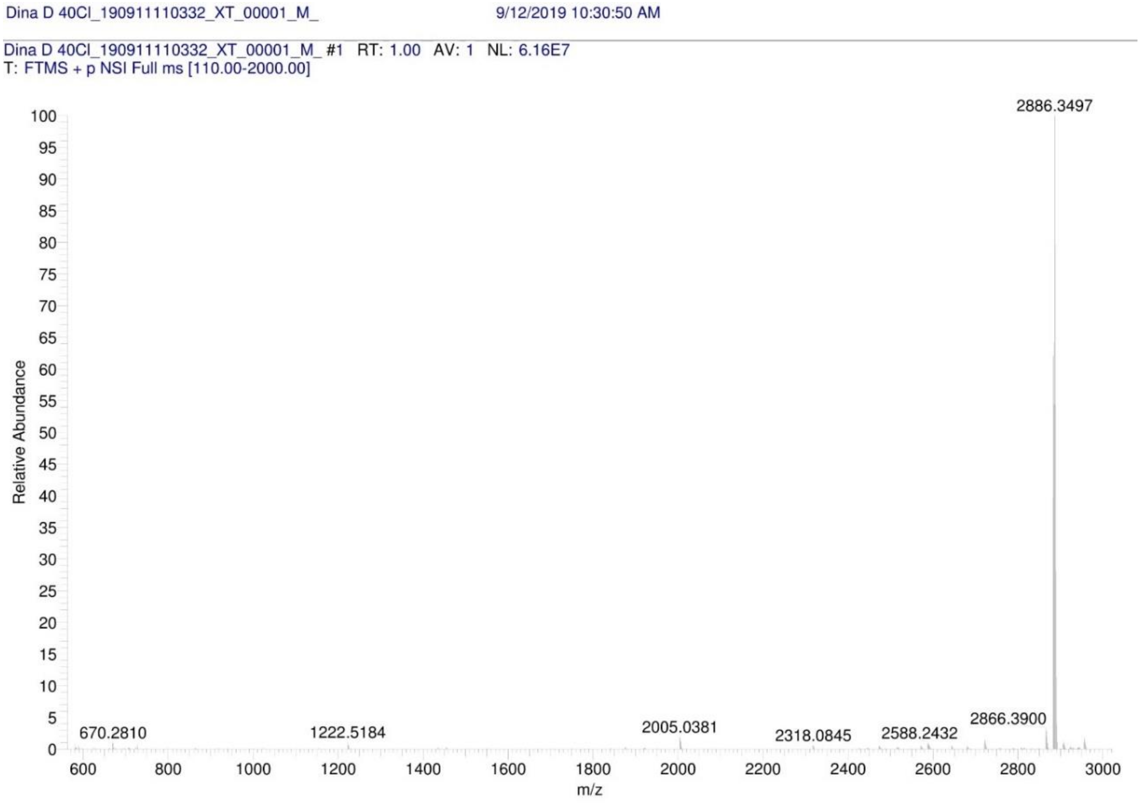

Dina D 40Cl_190911110332_XT_00001_M_
9/12/2019 10:30:50 AM
Dina D 40Cl_190911110332_XT_00001_M_ #1 RT: 1.00 AV: 1 NL: 6.16E7
T: FTMS + p NSI Full ms [110.00-2000.00]
2886.3497
670.2810
1222.5184
2005.0381
2318.0845
2588.2432
2866.3900
Relative Abundance
m/z

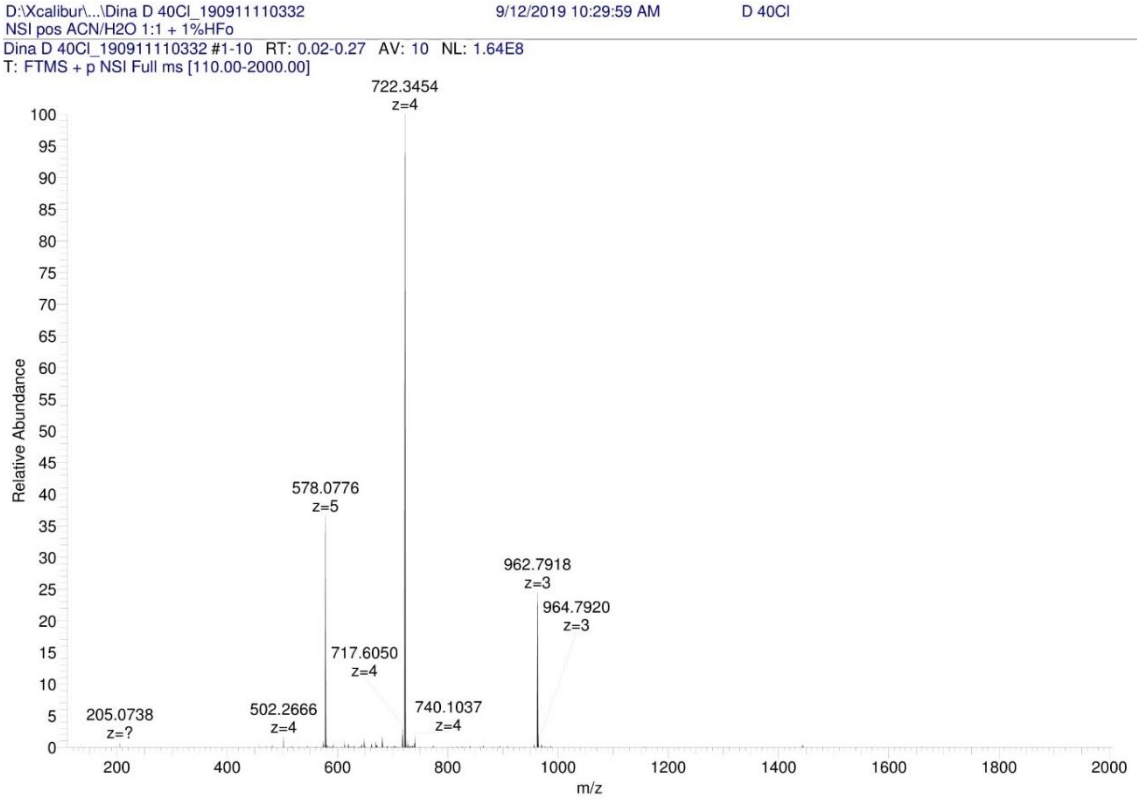

D:\Xcalibur\...\Dina D 40Cl_190911110332
9/12/2019 10:29:59 AM
D 40Cl
NSI pos ACN/H2O 1:1 + 1%HFo
Dina D 40Cl_190911110332 #1-10 RT: 0.02-0.27 AV: 10 NL: 1.64E8
T: FTMS + p NSI Full ms [110.00-2000.00]
722.3454 z=4
578.0776 z=5
962.7918 z=3
964.7920 z=3
717.6050 z=4
740.1037 z=4
502.2666 z=4
205.0738 z=?
Relative Abundance
m/z

Dina D 40Cl_190911110332_XT_00001_M_ 9/12/2019 10:30:50 AM

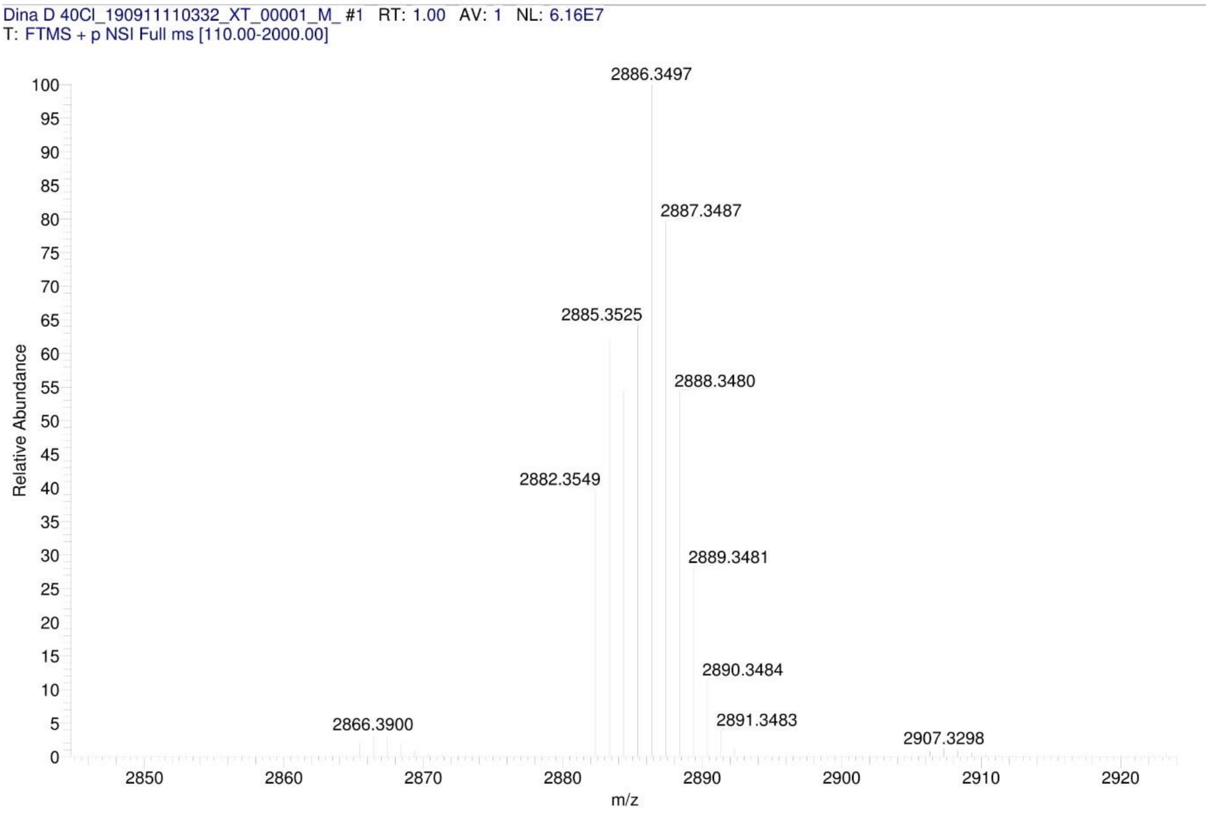

Ac**15 ((AcAKA)$_4$(*K*EY)$_2$*K*YKK-NH$_2$)** was obtained from the CEM Liberty Blue synthetiser as foamy colourless solid after preparative RP-HPLC (31.7 mg, 13.4 µmol, 49.3%). Analytical RP-HPLC: t_R=1.31 min (100% A to 100% D in 5 min, λ= 214 nm). HRMS (ESI+): $C_{123}H_{200}N_{32}O_{33}$ calc./obs. 2653.4956/2653.4904 Da [M].

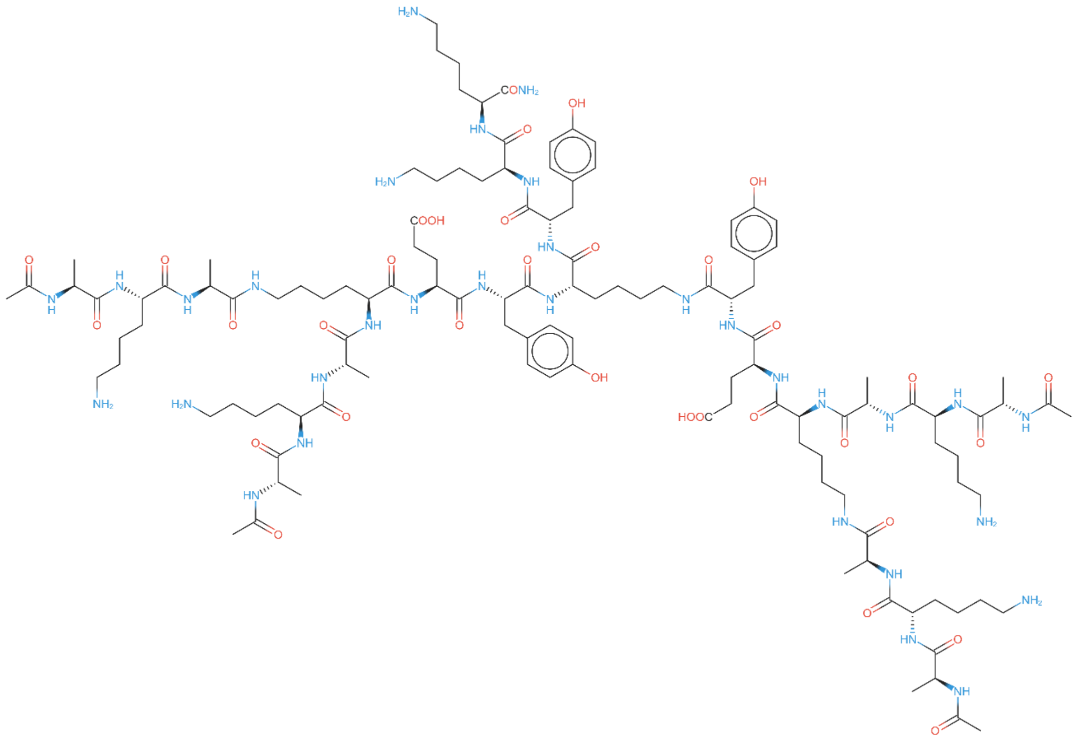

Analytical RP-HPLC:

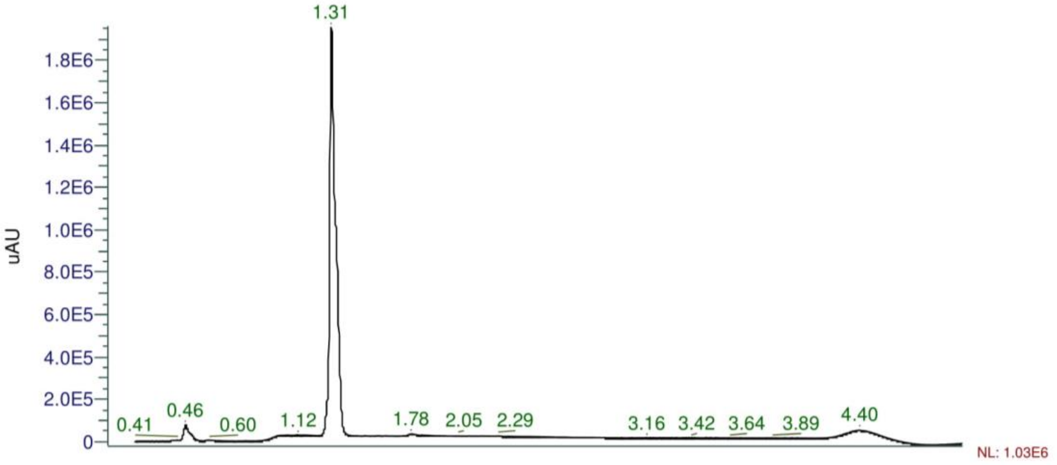

HRMS (NSI+):

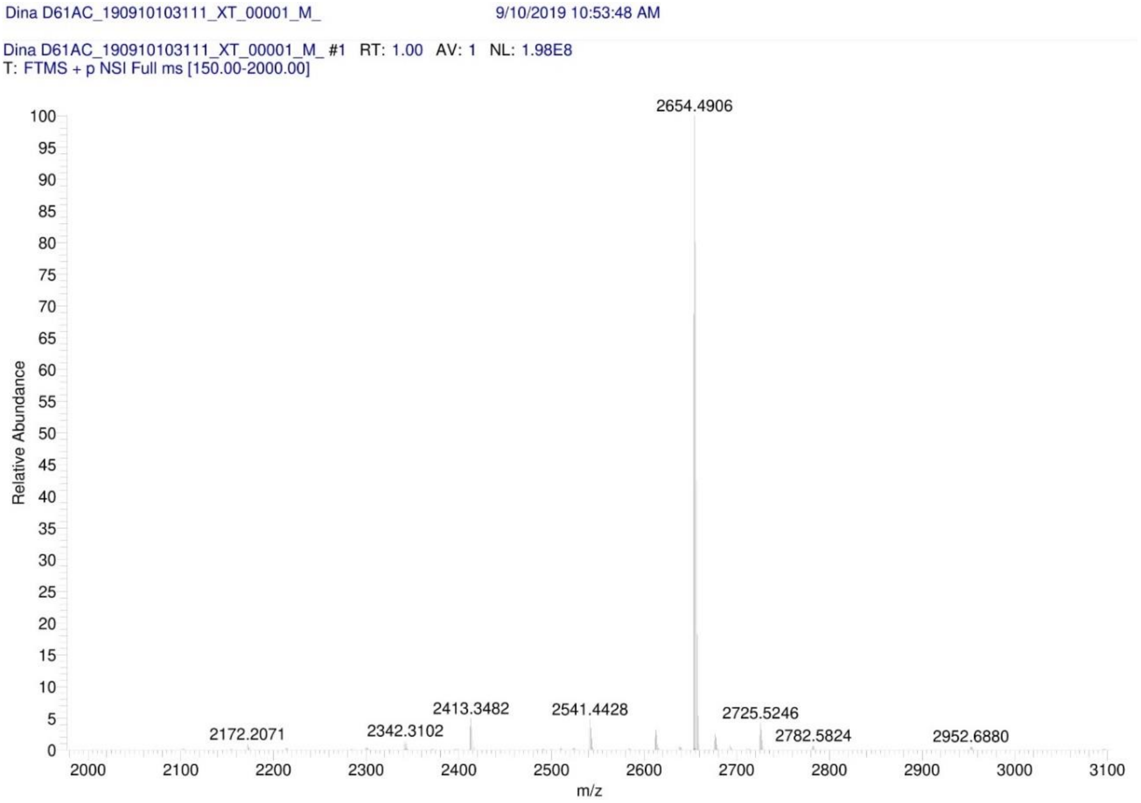

Dina D61AC_190910103111_XT_00001_M_
9/10/2019 10:53:48 AM
Dina D61AC_190910103111_XT_00001_M_ #1 RT: 1.00 AV: 1 NL: 1.98E8
T: FTMS + p NSI Full ms [150.00-2000.00]
2654.4906
2172.2071
2342.3102
2413.3482
2541.4428
2725.5246
2782.5824
2952.6880
Relative Abundance
m/z

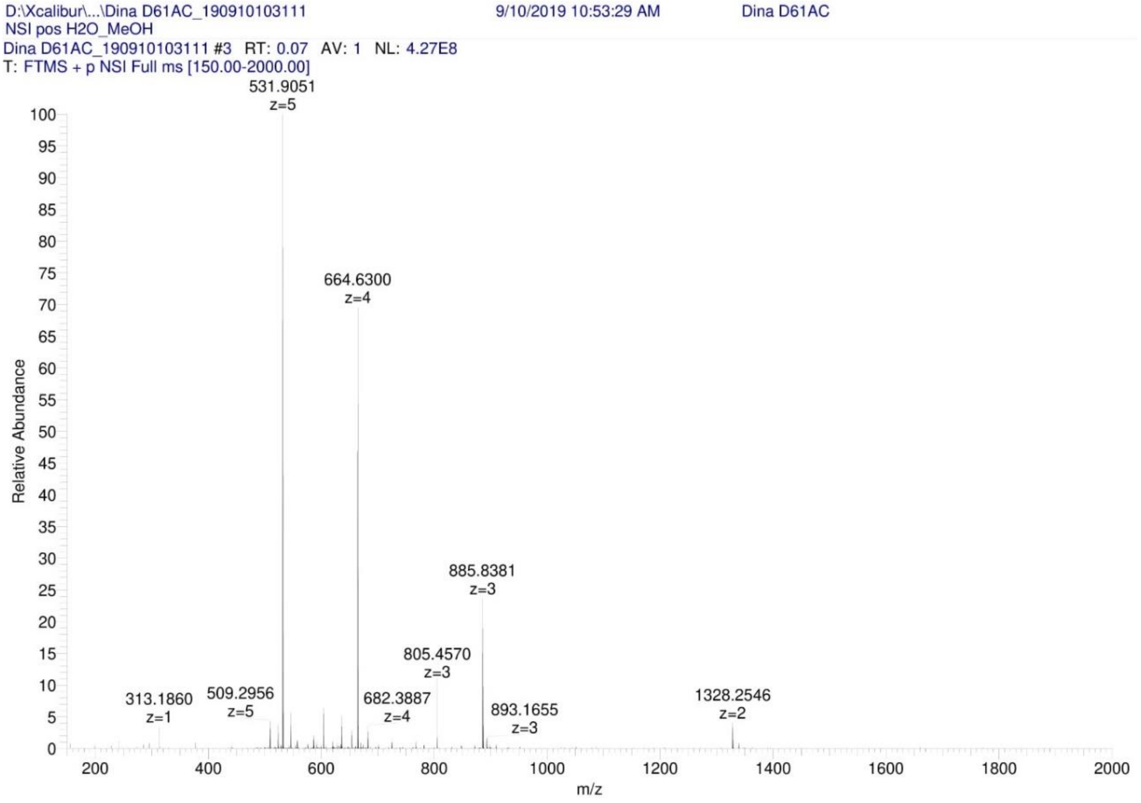

D:\Xcalibur\...\Dina D61AC_190910103111
NSI pos H2O_MeOH
9/10/2019 10:53:29 AM
Dina D61AC
Dina D61AC_190910103111 #3 RT: 0.07 AV: 1 NL: 4.27E8
T: FTMS + p NSI Full ms [150.00-2000.00]
531.9051 z=5
664.6300 z=4
885.8381 z=3
805.4570 z=3
313.1860 z=1
509.2956 z=5
682.3887 z=4
893.1655 z=3
1328.2546 z=2
Relative Abundance
m/z

Dina D61AC_190910103111_XT_00001_M_ 9/10/2019 10:53:48 AM

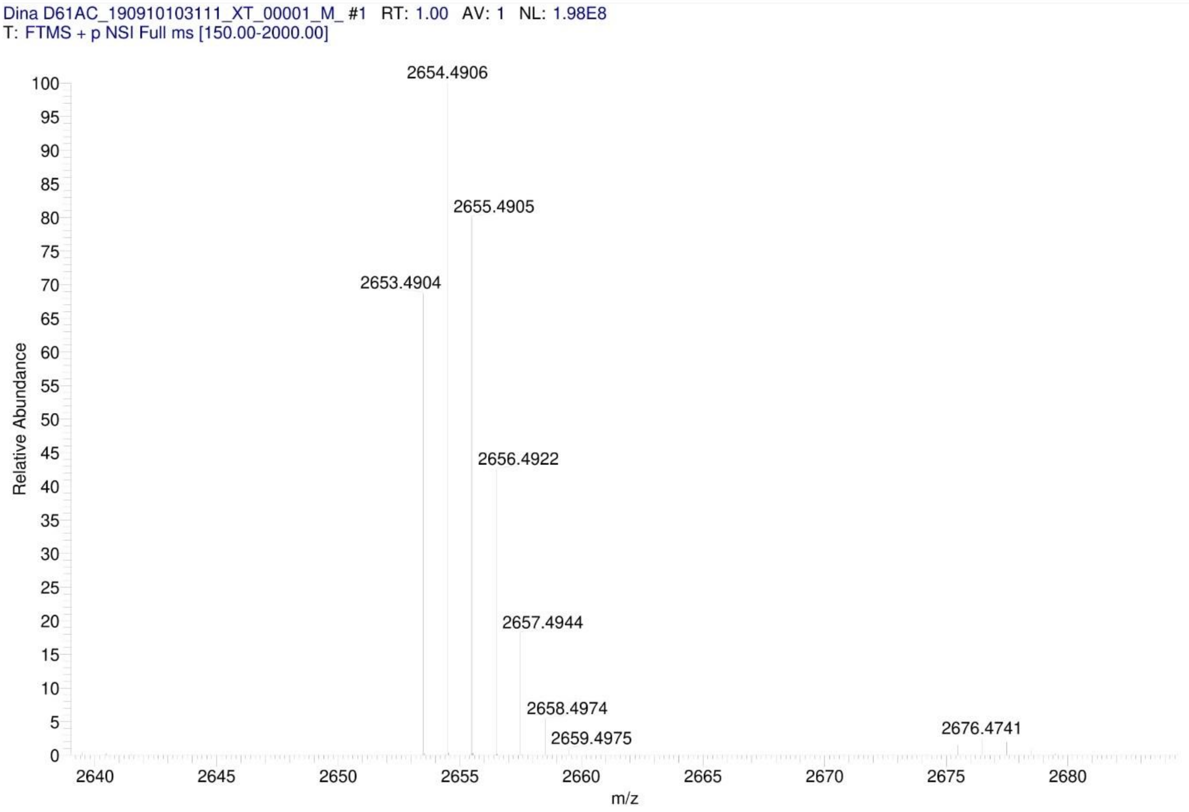

Fum**15** **((FumAKA)$_4$(*K*EY)$_2$*K*YKK-NH$_2$)** was obtained from the CEM Liberty Blue synthetiser as foamy colourless solid after preparative RP-HPLC (15.0 mg, 6.5 µmol, 23.6%). Analytical RP-HPLC: t_R=1.21 min (100% A to 100% D in 5 min, λ= 214 nm). HRMS (ESI+): $C_{115}H_{192}N_{32}O_{29}$ calc./obs. 2485.4533/2485.4561 Da [M].

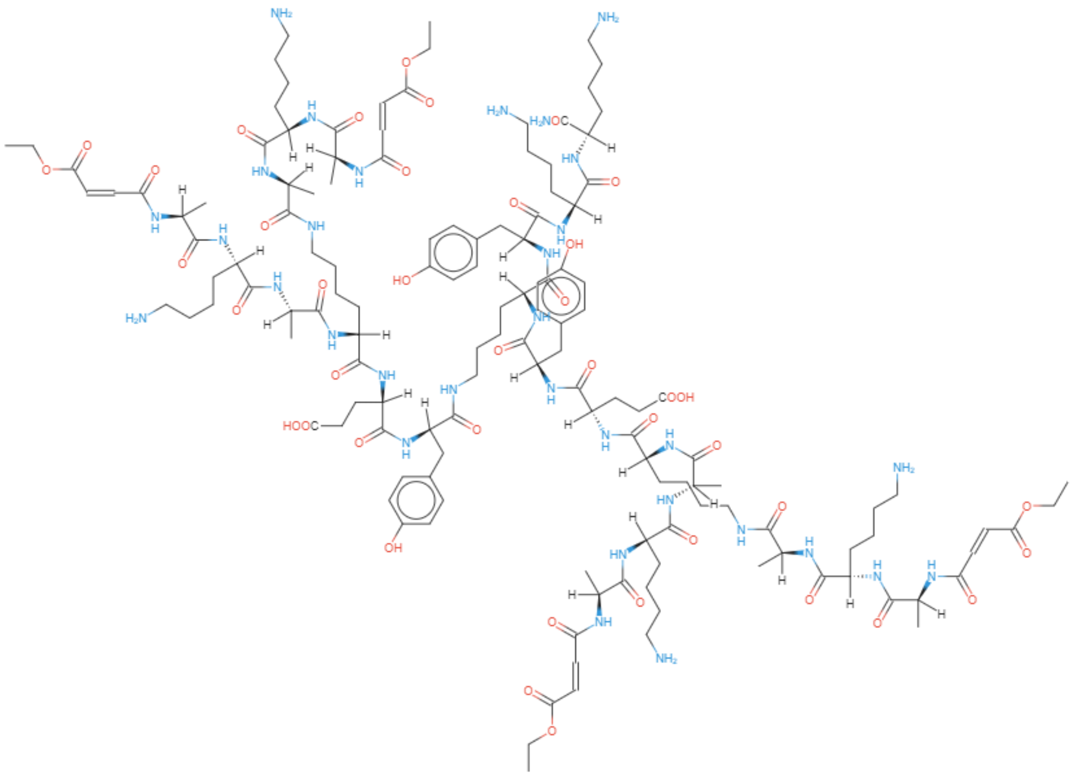

Analytical RP-HPLC:

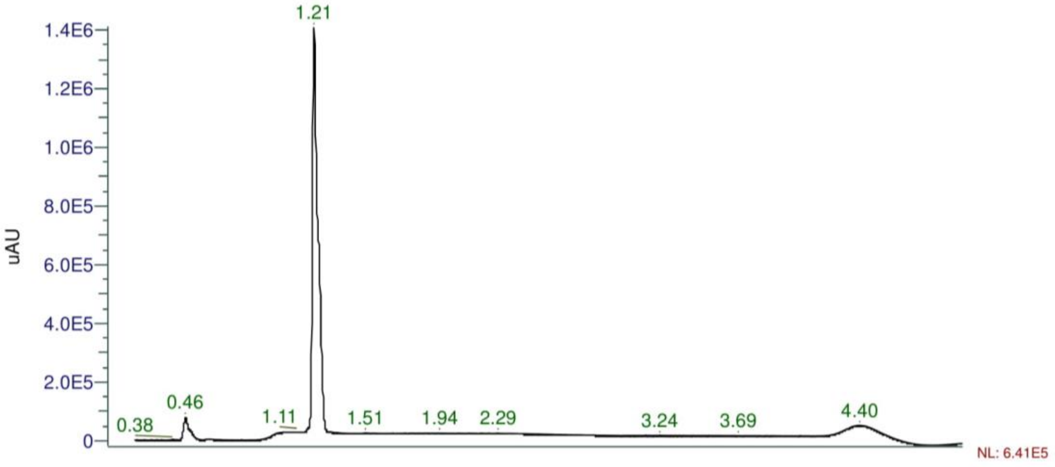

HRMS (NSI+):

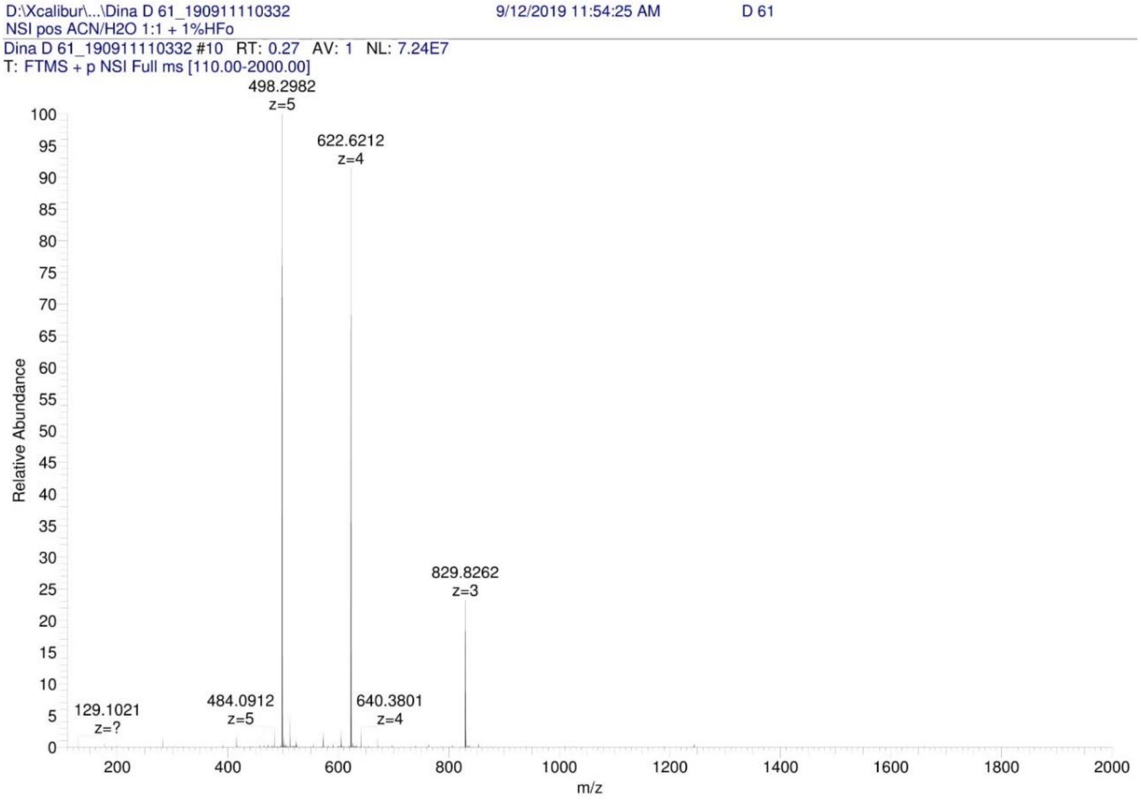
D:\Xcalibur\...\Dina D 61_190911110332
9/12/2019 11:54:25 AM
D 61
NSI pos ACN/H2O 1:1 + 1%HFo
Dina D 61_190911110332 #10 RT: 0.27 AV: 1 NL: 7.24E7
T: FTMS + p NSI Full ms [110.00-2000.00]
498.2982
z=5
622.6212
z=4
829.8262
z=3
129.1021
z=?
484.0912
z=5
640.3801
z=4
Relative Abundance
m/z

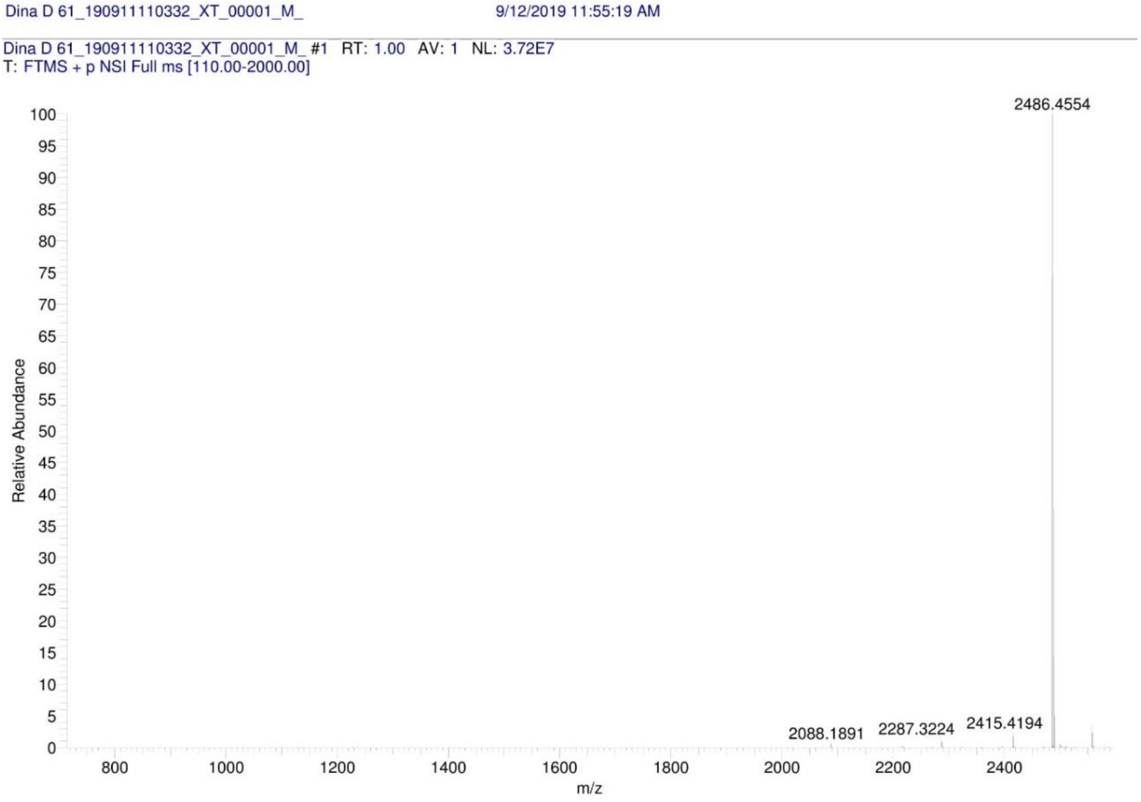
Dina D 61_190911110332_XT_00001_M_
9/12/2019 11:55:19 AM
Dina D 61_190911110332_XT_00001_M_ #1 RT: 1.00 AV: 1 NL: 3.72E7
T: FTMS + p NSI Full ms [110.00-2000.00]
2486.4554
2088.1891
2287.3224
2415.4194
Relative Abundance
m/z

Dina D 61_190911110332_XT_00001_M_ 9/12/2019 11:55:19 AM

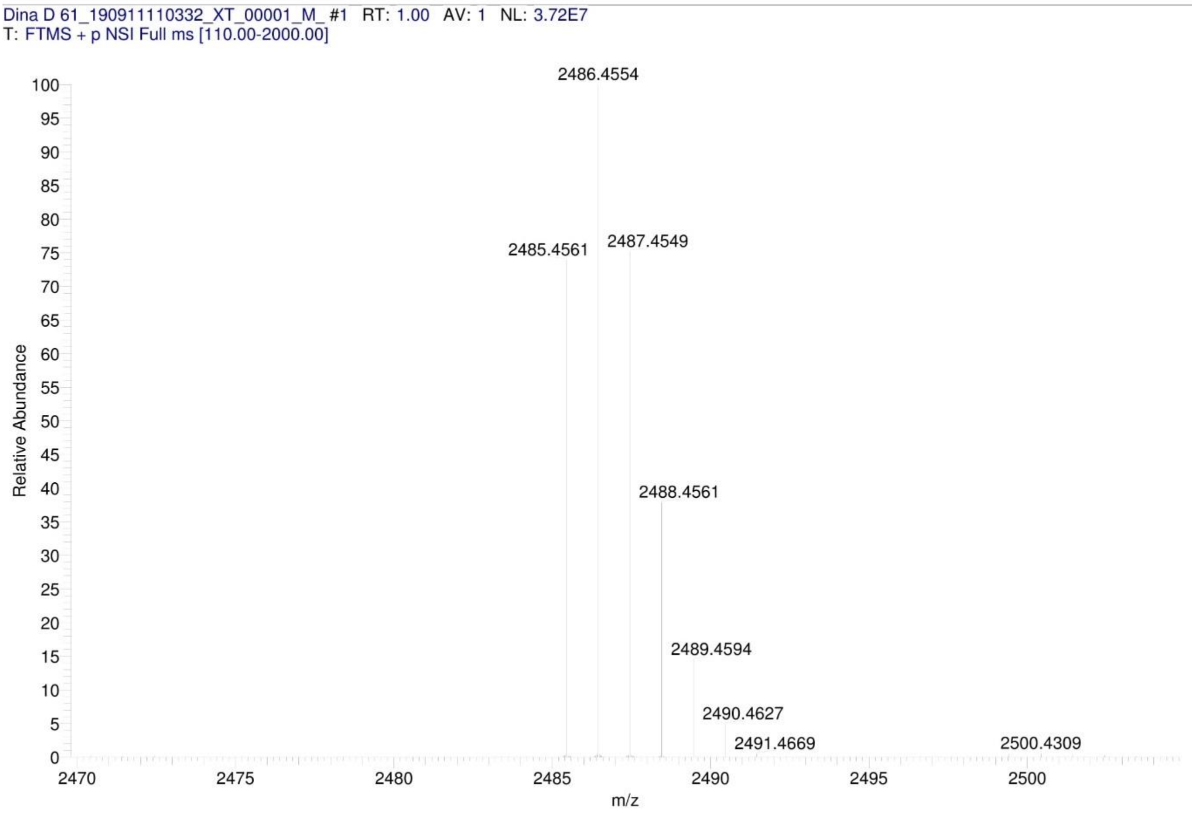

16 ((AKE)$_4$(*K*AYA)$_2$*K*KKK-NH$_2$) was obtained from the CEM Liberty Blue synthetiser as foamy colourless solid after preparative RP-HPLC (13.5 mg, 2.9 µmol, 11.4%). Analytical RP-HPLC: t_R=1.18 min (100% A to 100% D in 5 min, λ= 214 nm). HRMS (ESI+): $C_{122}H_{209}N_{35}O_{34}$ calc./obs. 2708.5701/2708.5633 Da [M].

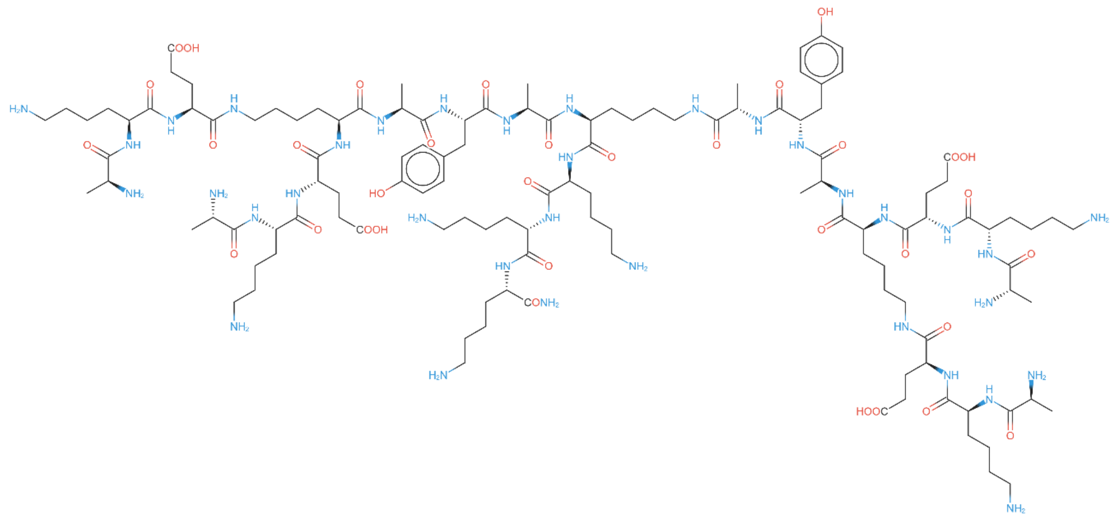

Analytical RP-HPLC:

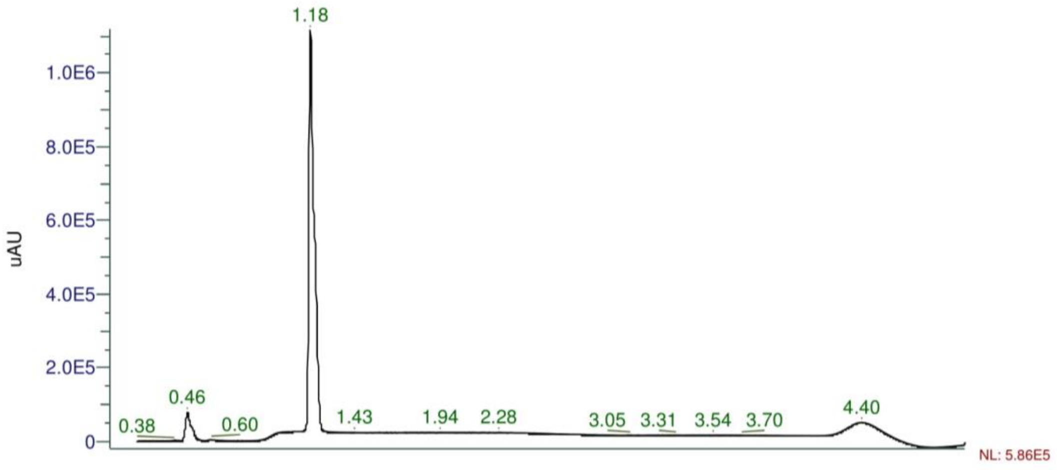

HRMS (NSI+):

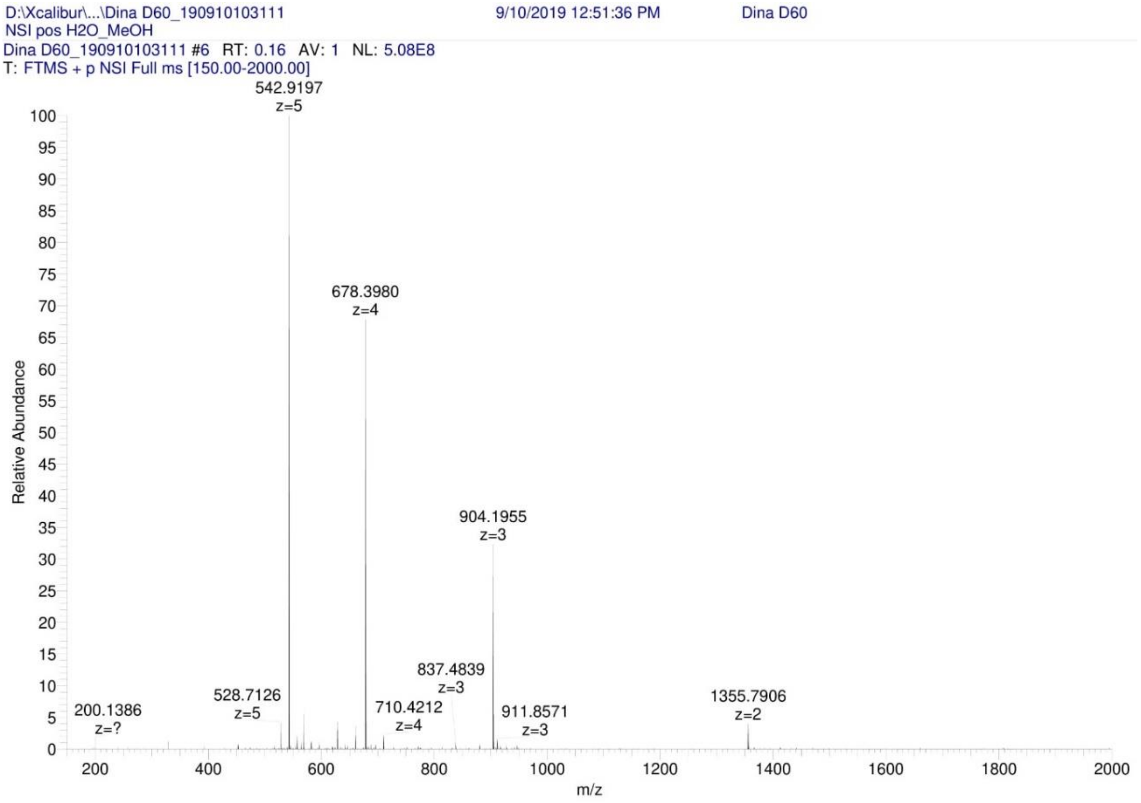
D:\Xcalibur\...\Dina D60_190910103111
9/10/2019 12:51:36 PM
Dina D60
NSI pos H2O_MeOH
Dina D60_190910103111 #6 RT: 0.16 AV: 1 NL: 5.08E8
T: FTMS + p NSI Full ms [150.00-2000.00]
542.9197 z=5
678.3980 z=4
904.1955 z=3
837.4839 z=3
528.7126 z=5
710.4212 z=4
911.8571 z=3
200.1386 z=?
1355.7906 z=2
Relative Abundance
m/z

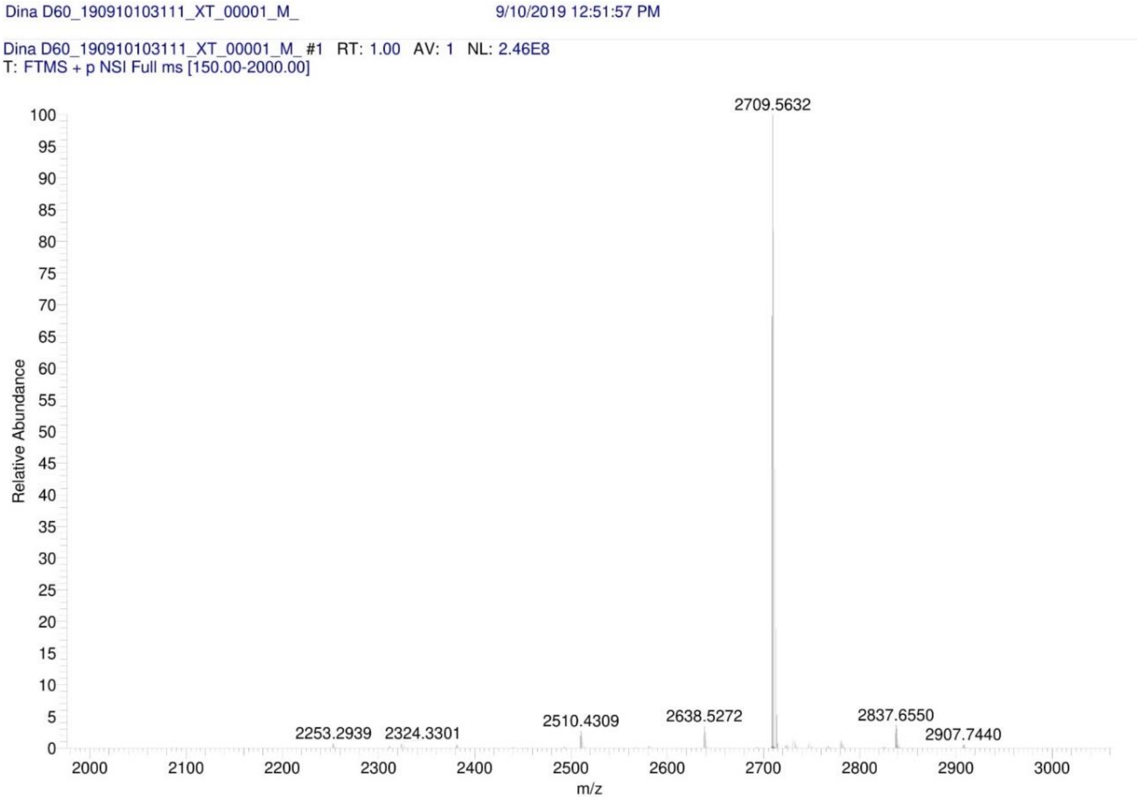
Dina D60_190910103111_XT_00001_M_
9/10/2019 12:51:57 PM
Dina D60_190910103111_XT_00001_M_ #1 RT: 1.00 AV: 1 NL: 2.46E8
T: FTMS + p NSI Full ms [150.00-2000.00]
2709.5632
2253.2939
2324.3301
2510.4309
2638.5272
2837.6550
2907.7440
Relative Abundance
m/z

Dina D60_190910103111_XT_00001_M_ 9/10/2019 12:51:57 PM

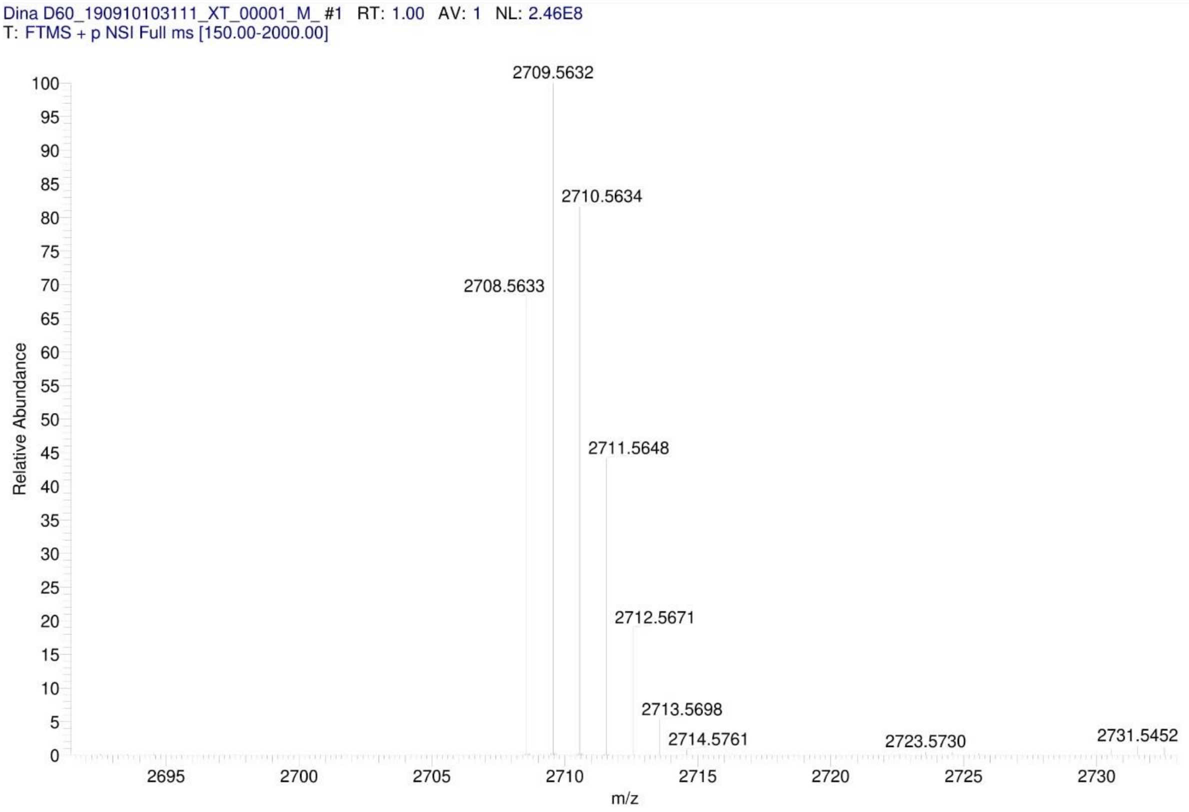

Ac**16 ((AcAKE)$_4$(*K*AYA)$_2$*K*KKK-NH$_2$)** was obtained from the CEM Liberty Blue synthetiser as foamy colourless solid after preparative RP-HPLC (13.8 mg, 3.4 µmol, 12.5%). Analytical RP-HPLC: t_R=1.27 min (100% A to 100% D in 5 min, λ= 214 nm). HRMS (ESI+): $C_{130}H_{217}N_{35}O_{38}$ calc./obs. 2876.6124/2876.6340 Da [M].

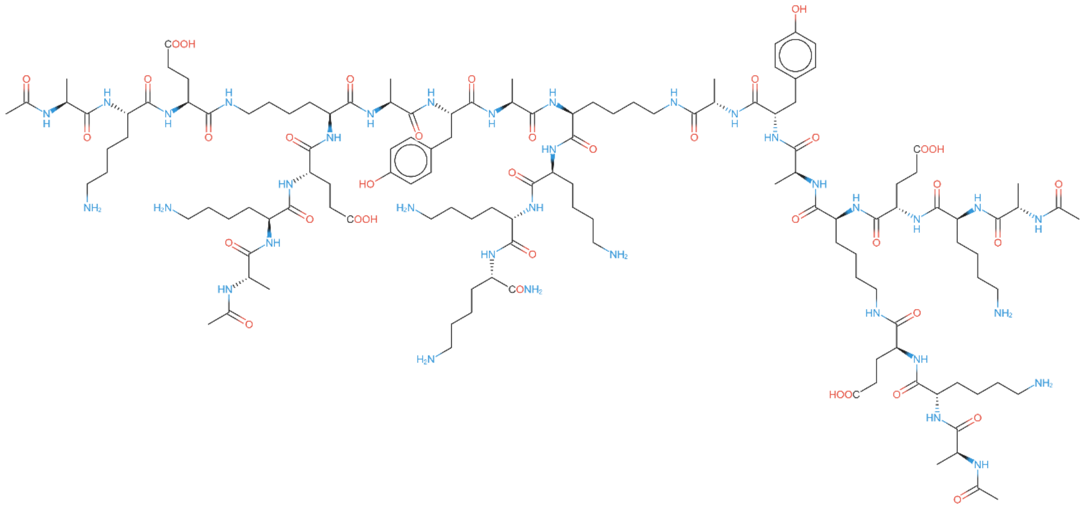

Analytical RP-HPLC:

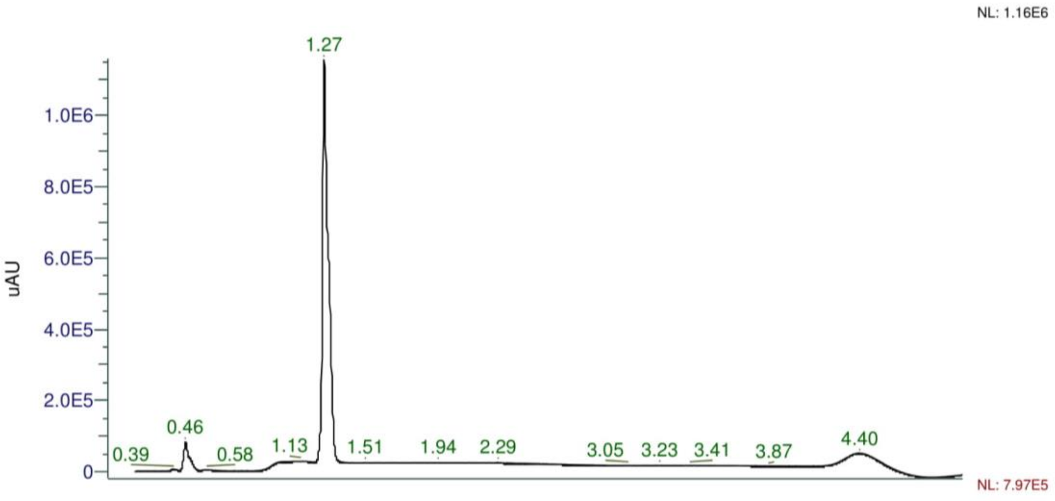

HRMS (NSI+):

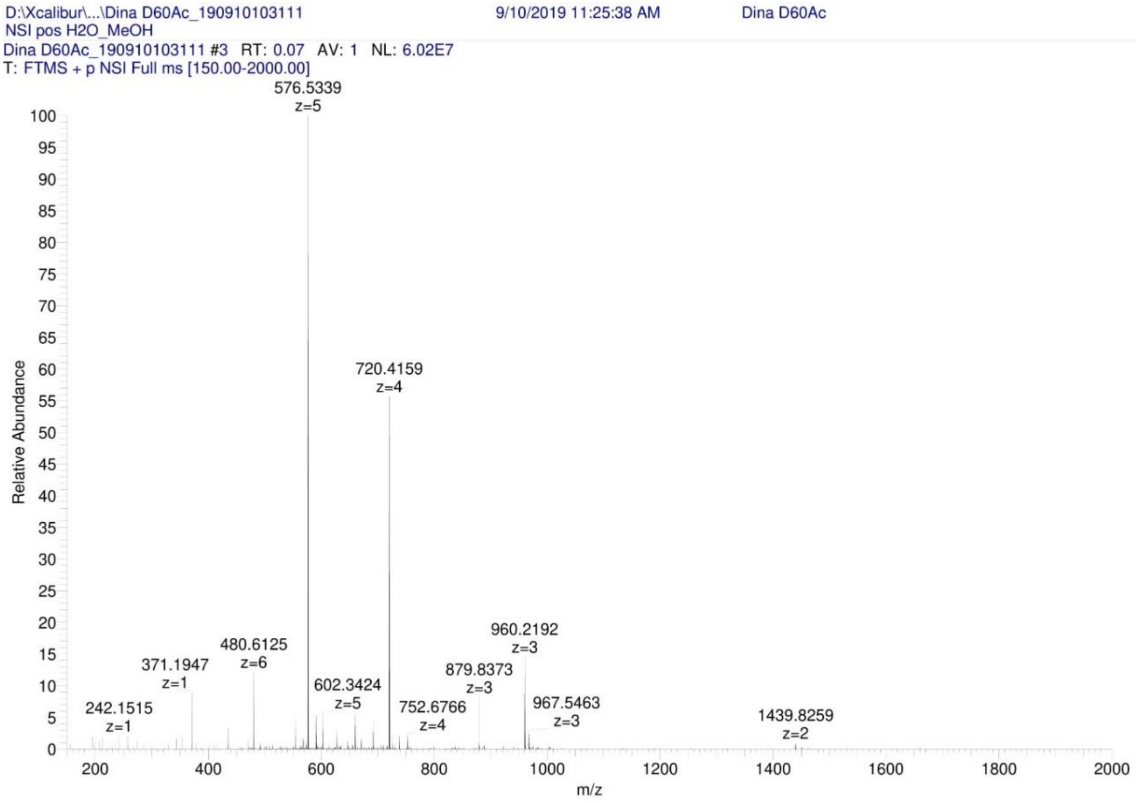
D:\Xcalibur\...\Dina D60Ac_190910103111
9/10/2019 11:25:38 AM
Dina D60Ac
NSI pos H2O_MeOH
Dina D60Ac_190910103111 #3 RT: 0.07 AV: 1 NL: 6.02E7
T: FTMS + p NSI Full ms [150.00-2000.00]
576.5339
z=5
720.4159
z=4
960.2192
z=3
480.6125
z=6
371.1947
z=1
879.8373
z=3
602.3424
z=5
967.5463
z=3
242.1515
z=1
752.6766
z=4
1439.8259
z=2
Relative Abundance
m/z

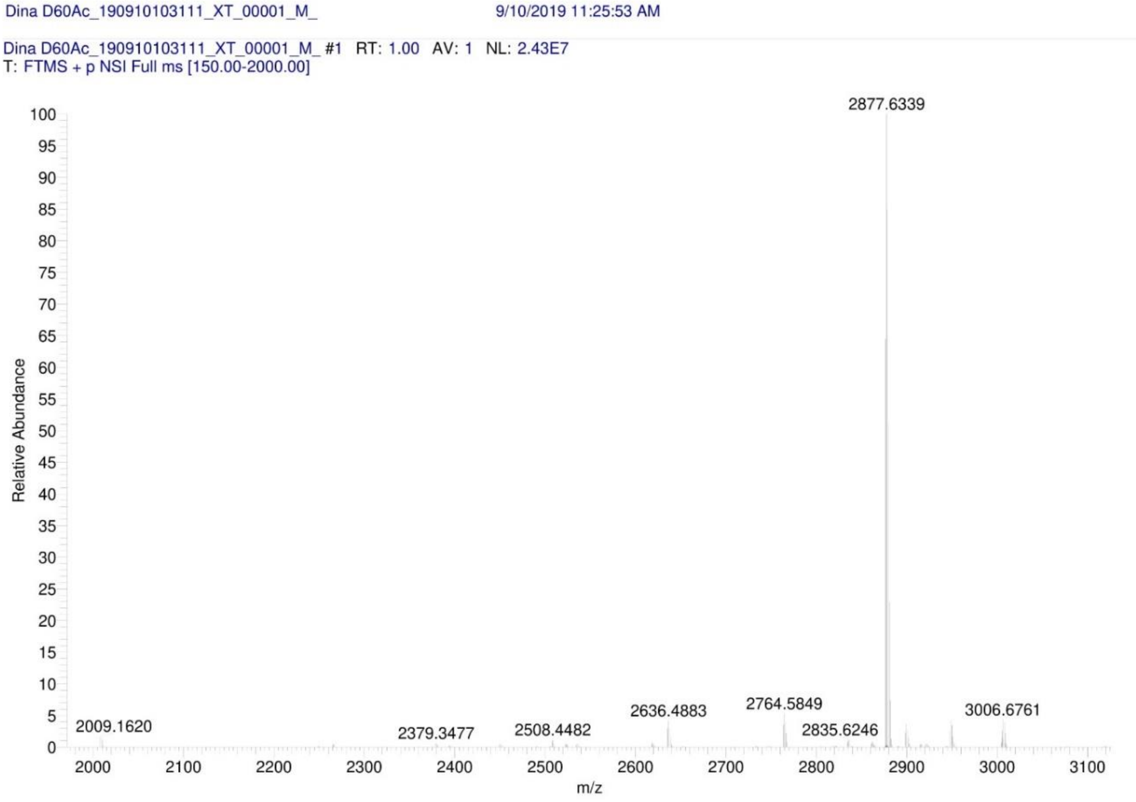
Dina D60Ac_190910103111_XT_00001_M_
9/10/2019 11:25:53 AM
Dina D60Ac_190910103111_XT_00001_M_ #1 RT: 1.00 AV: 1 NL: 2.43E7
T: FTMS + p NSI Full ms [150.00-2000.00]
2877.6339
2009.1620
2379.3477
2508.4482
2636.4883
2764.5849
2835.6246
3006.6761
Relative Abundance
m/z

Dina D60Ac_190910103111_XT_00001_M_ 9/10/2019 11:25:53 AM

Dina D60Ac_190910103111_XT_00001_M_ #1 RT: 1.00 AV: 1 NL: 2.43E7
T: FTMS + p NSI Full ms [150.00-2000.00]

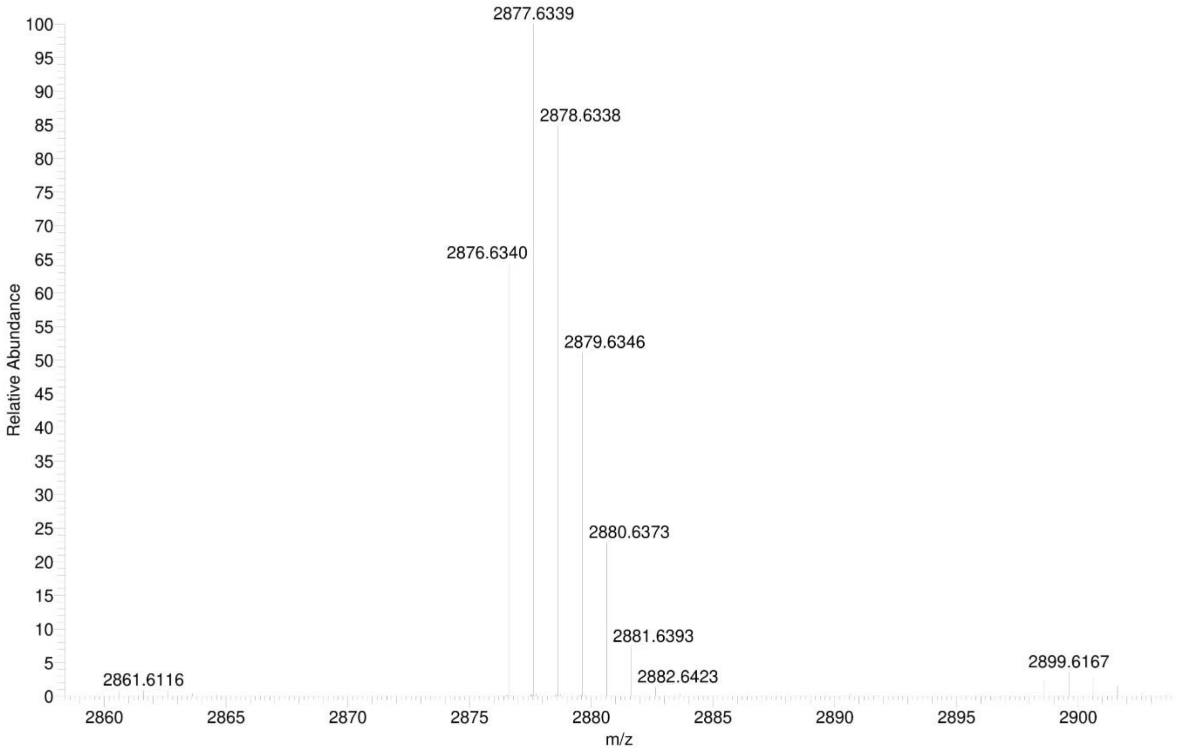

Fum**16** **((FumAKE)$_4$(*K*AYA)$_2$*K*KKK-NH$_2$)** was obtained from the CEM Liberty Blue synthetiser as foamy colourless solid after preparative RP-HPLC (13.5 mg, 6.5 µmol, 23.6%). Analytical RP-HPLC: t_R=1.48 min (100% A to 100% D in 5 min, λ= 214 nm). $C_{146}H_{233}N_{35}O_{46}$ calc./obs. 3212.6969/3212.7355 Da [M].

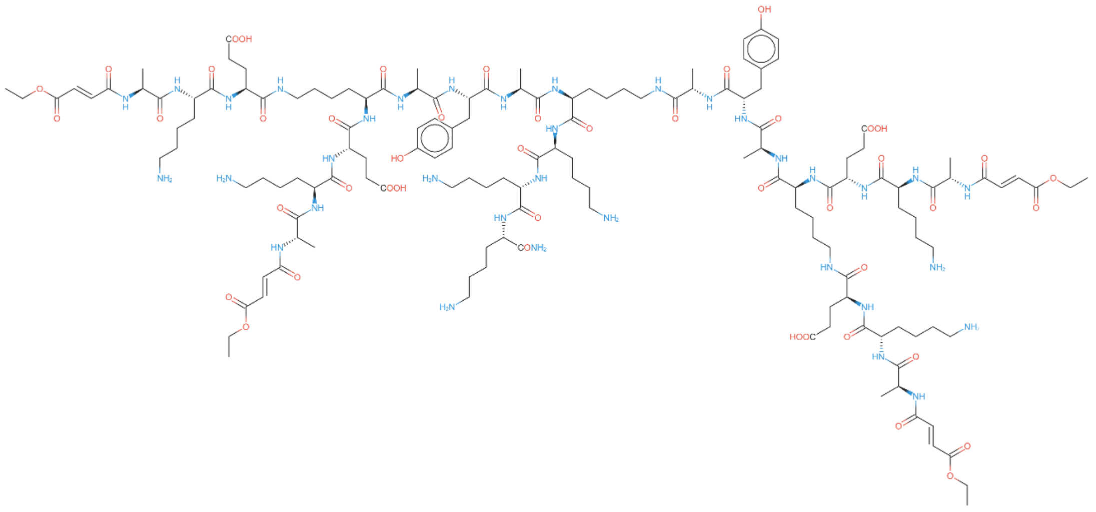

Analytical RP-HPLC:

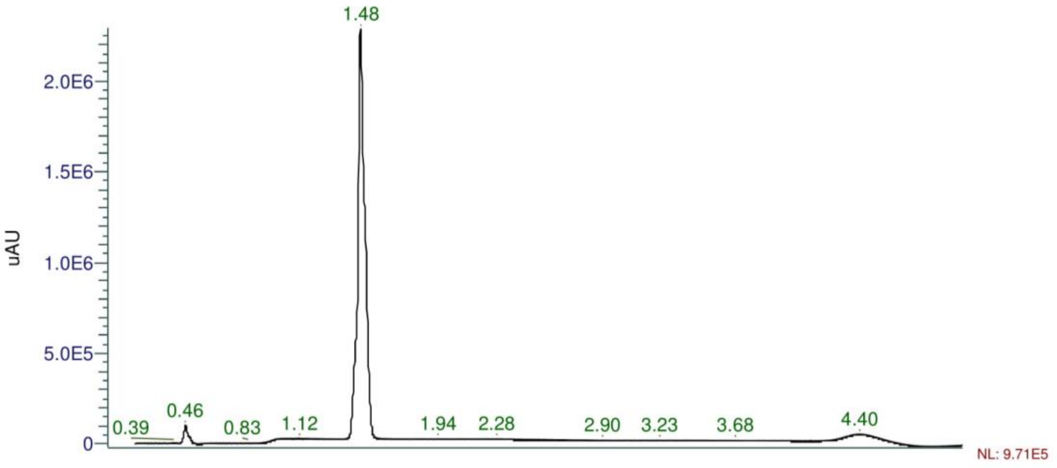

HRMS (NSI+):

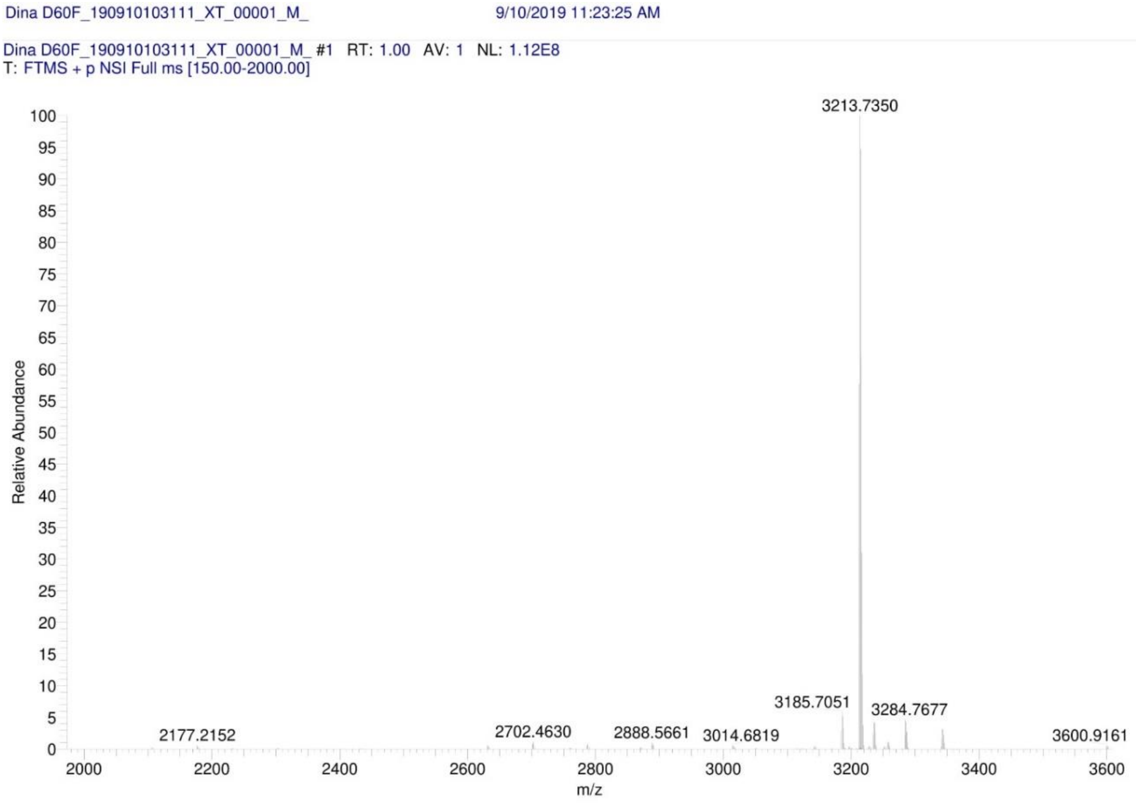
Dina D60F_190910103111_XT_00001_M_
9/10/2019 11:23:25 AM
Dina D60F_190910103111_XT_00001_M_ #1 RT: 1.00 AV: 1 NL: 1.12E8
T: FTMS + p NSI Full ms [150.00-2000.00]
3213.7350
3185.7051
3284.7677
2177.2152
2702.4630
2888.5661
3014.6819
3600.9161
Relative Abundance
m/z

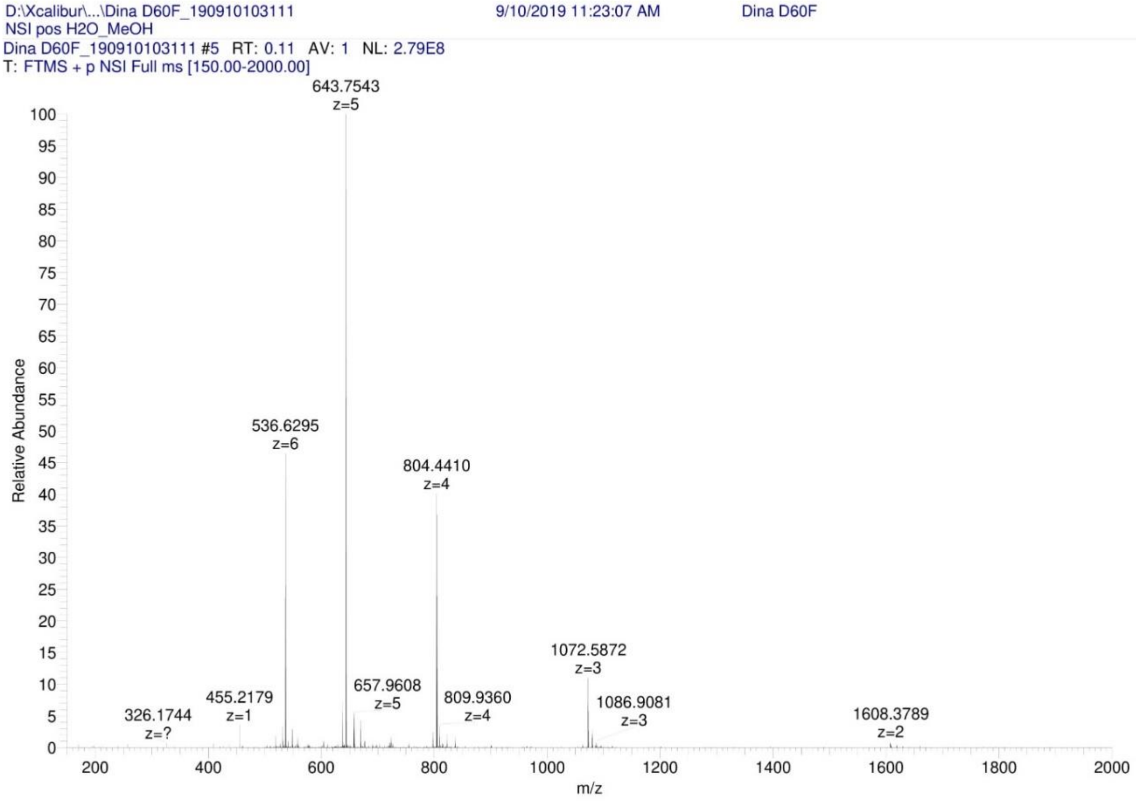
D:\Xcalibur\...\Dina D60F_190910103111
9/10/2019 11:23:07 AM
Dina D60F
NSI pos H2O_MeOH
Dina D60F_190910103111 #5 RT: 0.11 AV: 1 NL: 2.79E8
T: FTMS + p NSI Full ms [150.00-2000.00]
643.7543
z=5
536.6295
z=6
804.4410
z=4
1072.5872
z=3
657.9608
z=5
809.9360
z=4
1086.9081
z=3
455.2179
z=1
326.1744
z=?
1608.3789
z=2
Relative Abundance
m/z

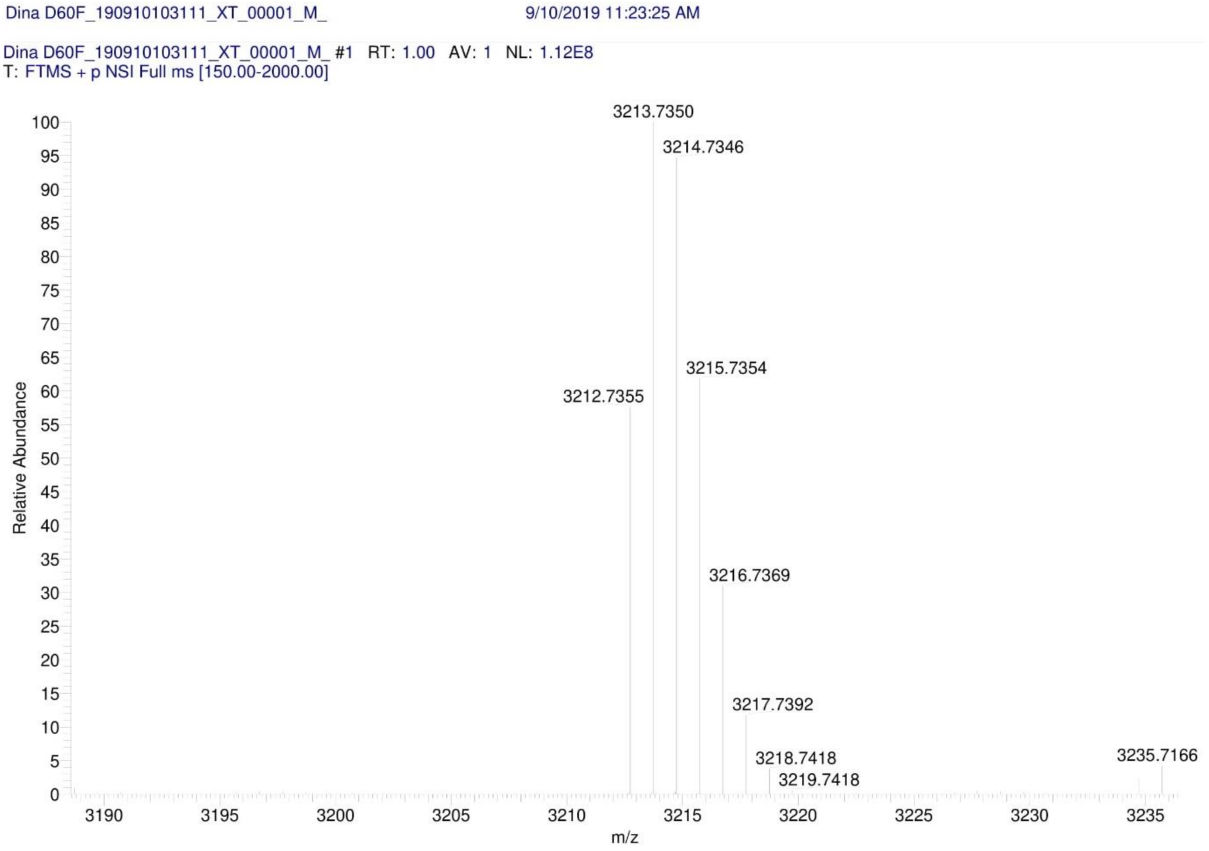
Dina D60F_190910103111_XT_00001_M_
9/10/2019 11:23:25 AM
Dina D60F_190910103111_XT_00001_M_ #1 RT: 1.00 AV: 1 NL: 1.12E8
T: FTMS + p NSI Full ms [150.00-2000.00]
3213.7350
3214.7346
3215.7354
3212.7355
3216.7369
3217.7392
3218.7418
3219.7418
3235.7166
Relative Abundance
m/z

17 ((K)$_4$(*K*AYAEY)$_2$*K*AKAEYA-NH$_2$) was obtained from the CEM Liberty Blue synthetiser as foamy colourless solid after preparative RP-HPLC (108.2 mg, 11.5 µmol, 43.5%). Analytical RP-HPLC: t_R=1.31 min (100% A to 100% D in 5 min, λ= 214 nm). HRMS (ESI+): $C_{129}H_{200}N_{32}O_{34}$ calc./obs. 2741.4905/2741.4961 Da [M].

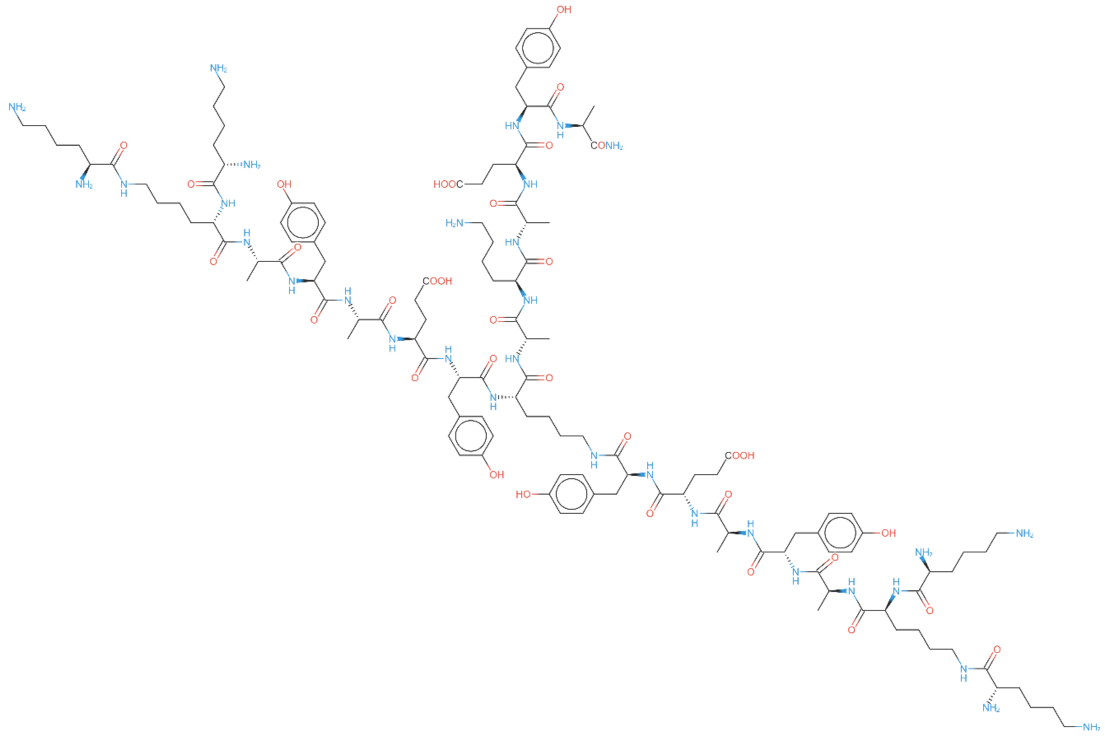

Analytical RP-HPLC:

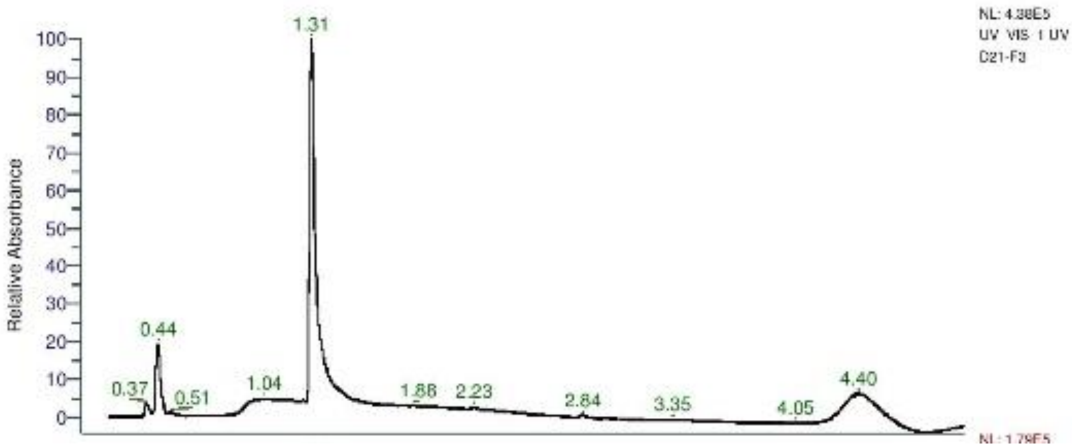

HRMS (NSI+):

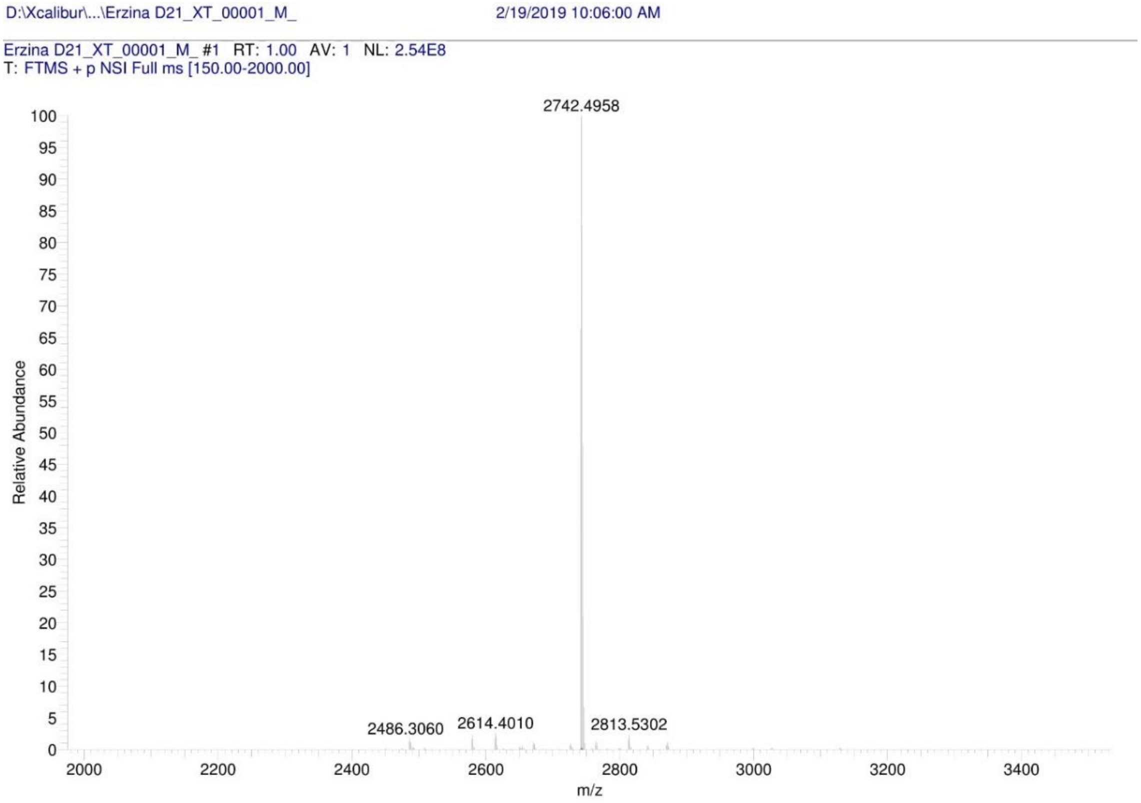
D:\Xcalibur\...\Erzina D21_XT_00001_M_
2/19/2019 10:06:00 AM
Erzina D21_XT_00001_M_ #1 RT: 1.00 AV: 1 NL: 2.54E8
T: FTMS + p NSI Full ms [150.00-2000.00]
2742.4958
2486.3060
2614.4010
2813.5302
Relative Abundance
m/z

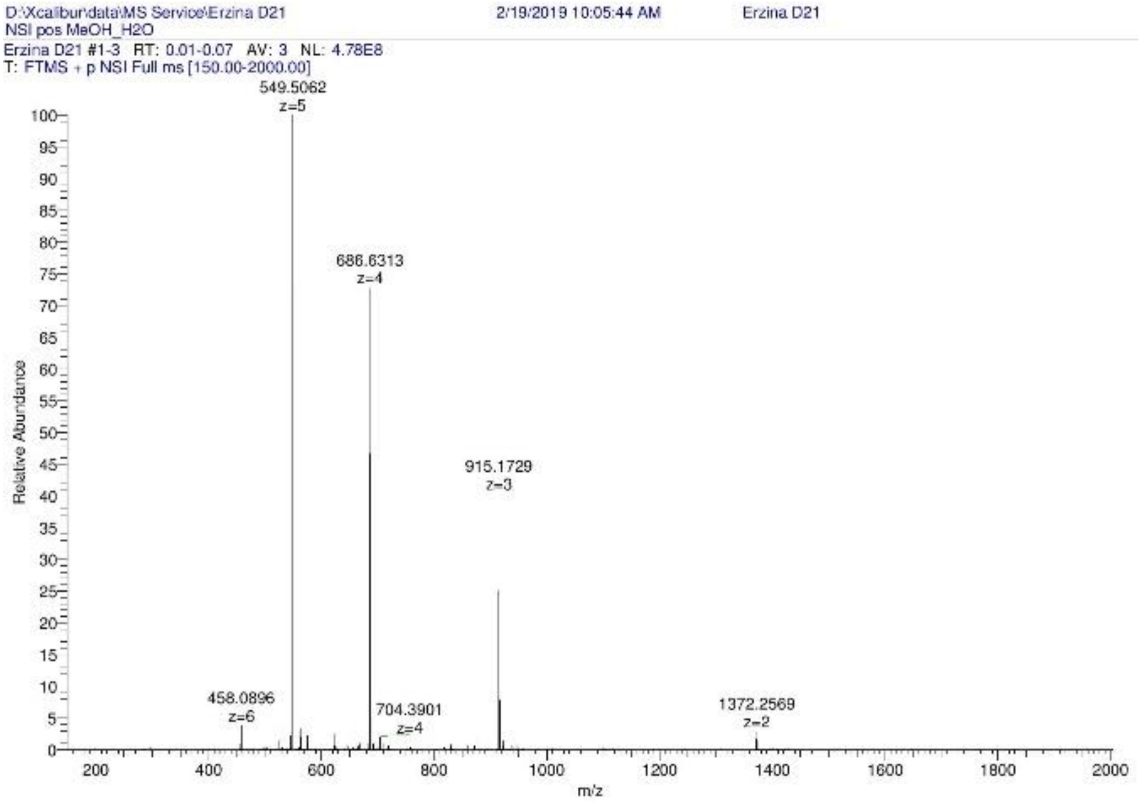
D:\Xcalibur\data\MS Service\Erzina D21
2/19/2019 10:05:44 AM
Erzina D21
NSI pos MeOH_H2O
Erzina D21 #1-3 RT: 0.01-0.07 AV: 3 NL: 4.78E8
T: FTMS + p NSI Full ms [150.00-2000.00]
549.5062
z=5
686.6313
z=4
915.1729
z=3
458.0896
z=6
704.3901
z=4
1372.2569
z=2
Relative Abundance
m/z

Dina D 21_190911110332_XT_00001_M_ 9/12/2019 7:56:38 AM

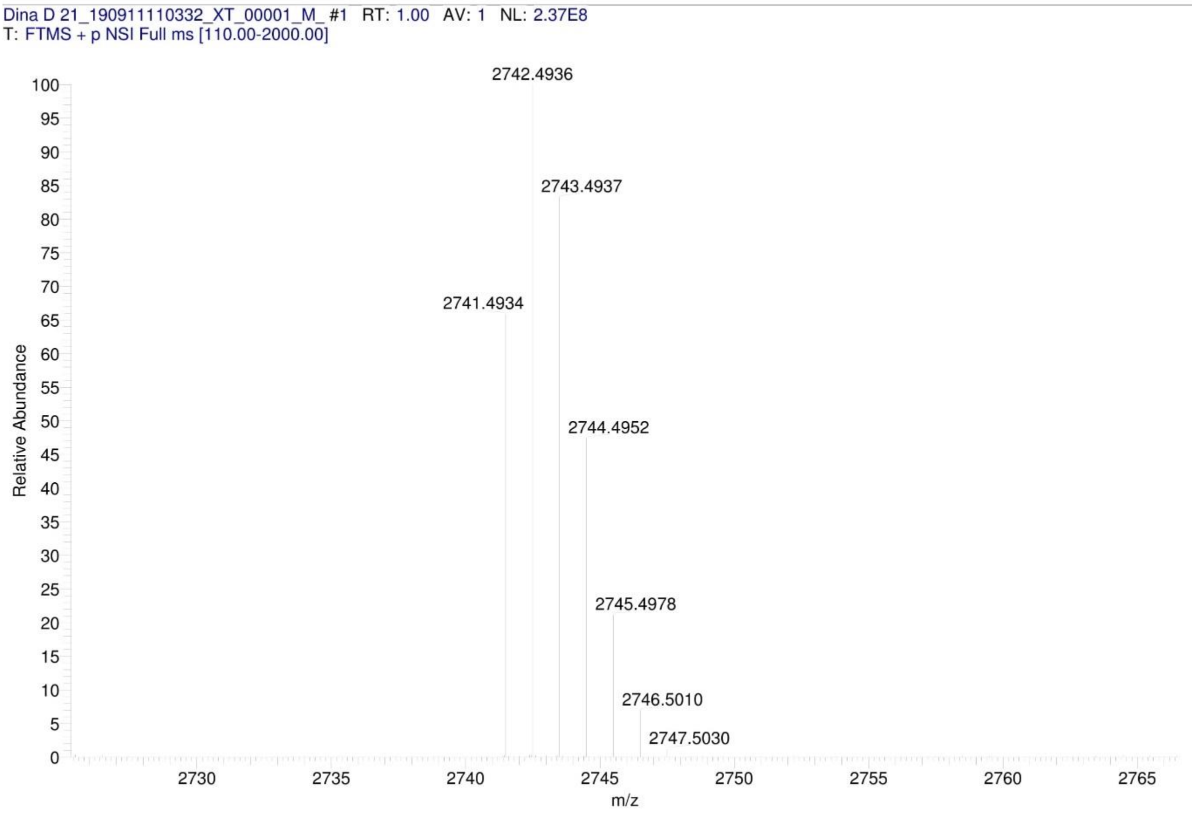

18 ((KAE)$_4$(*K*AEK)$_2$*K*YYA-OH) was obtained after manual synthesis as foamy colourless solid after preparative RP-HPLC (49.3 mg, 7.7 µmol, 30.0%). Analytical RP-HPLC: t_R=1.23 min (100% A to 100% D in 5 min, λ= 214 nm). HRMS (ESI+): $C_{123}H_{205}N_{33}O_{39}$ calc./obs. 2768.5072/2768.5122 Da [M].

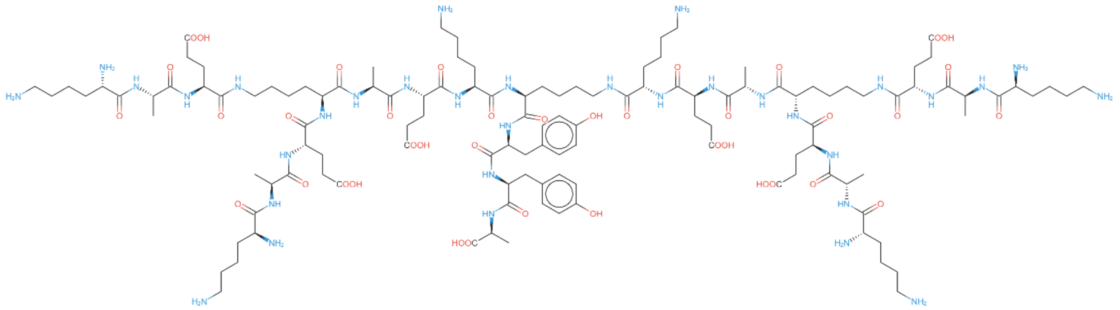

Analytical RP-HPLC:

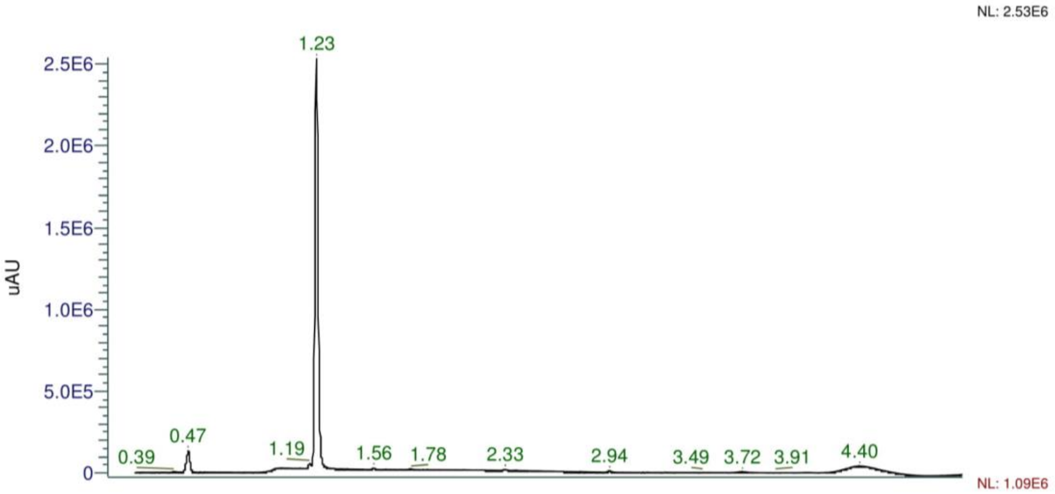

HRMS (NSI+):

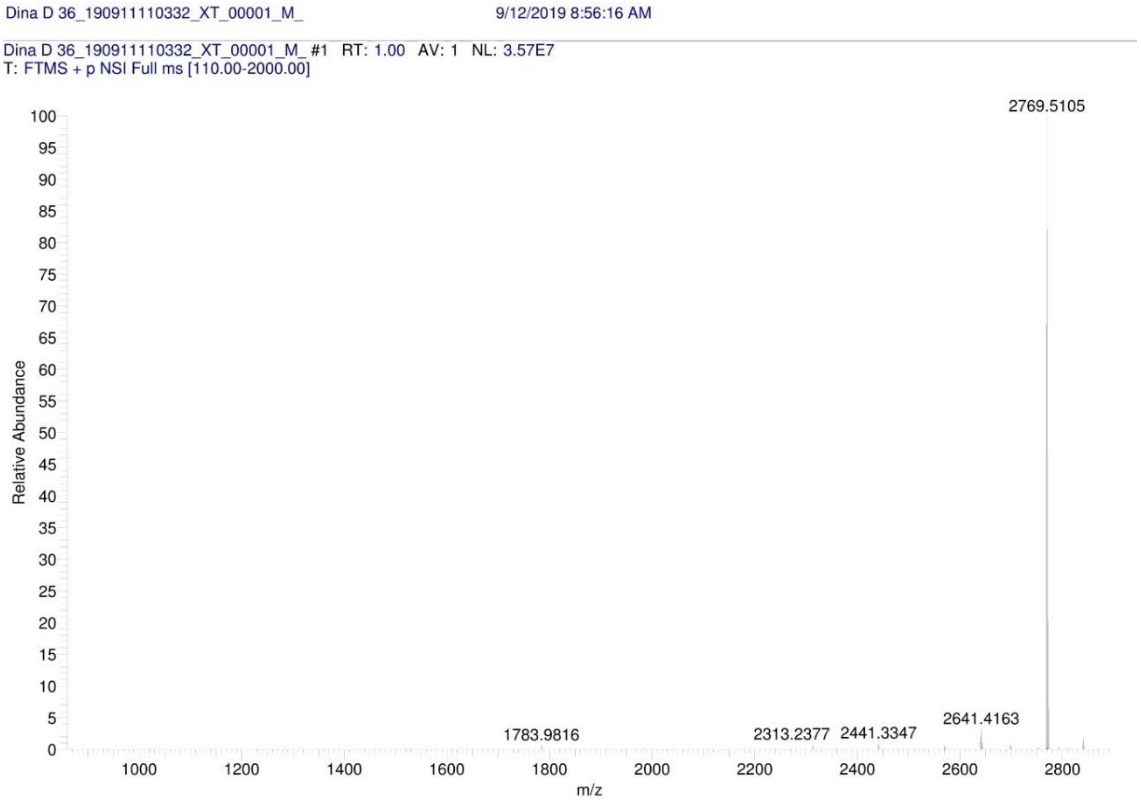
Dina D 36_190911110332_XT_00001_M_
9/12/2019 8:56:16 AM
Dina D 36_190911110332_XT_00001_M_ #1 RT: 1.00 AV: 1 NL: 3.57E7
T: FTMS + p NSI Full ms [110.00-2000.00]
2769.5105
2641.4163
1783.9816
2313.2377
2441.3347
Relative Abundance
m/z

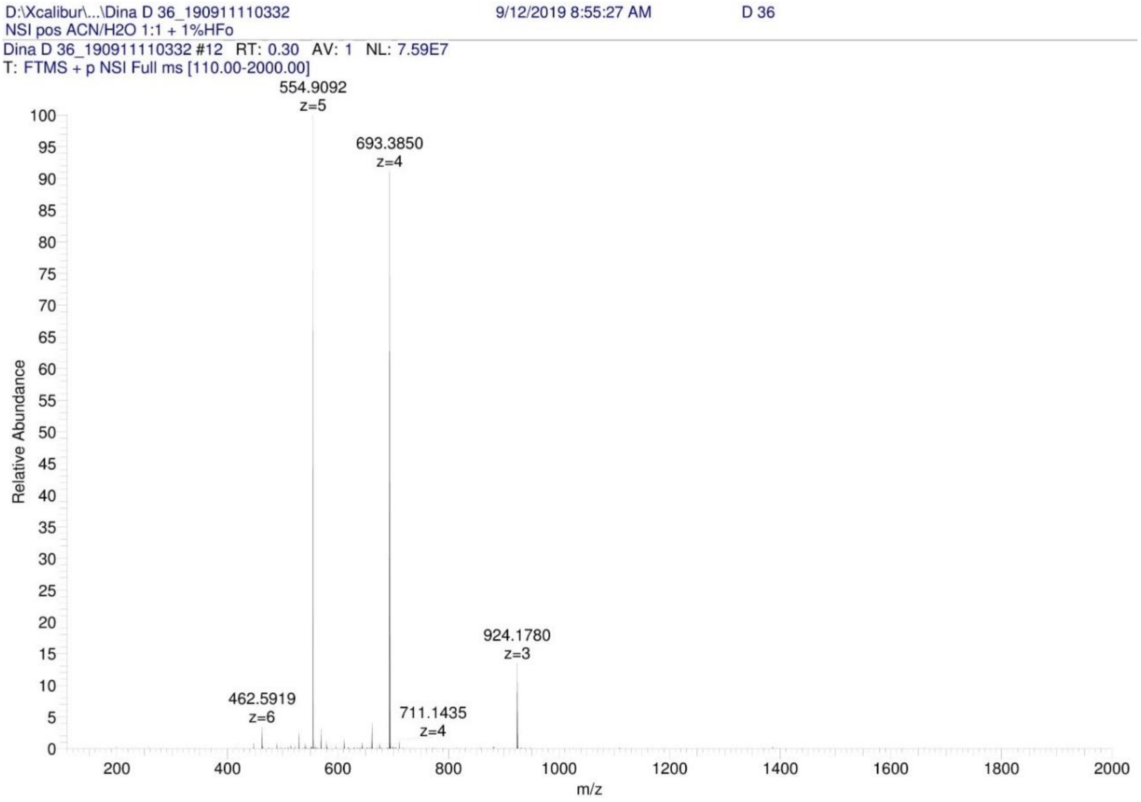
D:\Xcalibur\...\Dina D 36_190911110332
9/12/2019 8:55:27 AM
D 36
NSI pos ACN/H2O 1:1 + 1%HFo
Dina D 36_190911110332 #12 RT: 0.30 AV: 1 NL: 7.59E7
T: FTMS + p NSI Full ms [110.00-2000.00]
554.9092
z=5
693.3850
z=4
924.1780
z=3
462.5919
z=6
711.1435
z=4
Relative Abundance
m/z

Dina D 36_190911110332_XT_00001_M_ 9/12/2019 8:56:16 AM

Dina D 36_190911110332_XT_00001_M_ #1 RT: 1.00 AV: 1 NL: 3.57E7
T: FTMS + p NSI Full ms [110.00-2000.00]

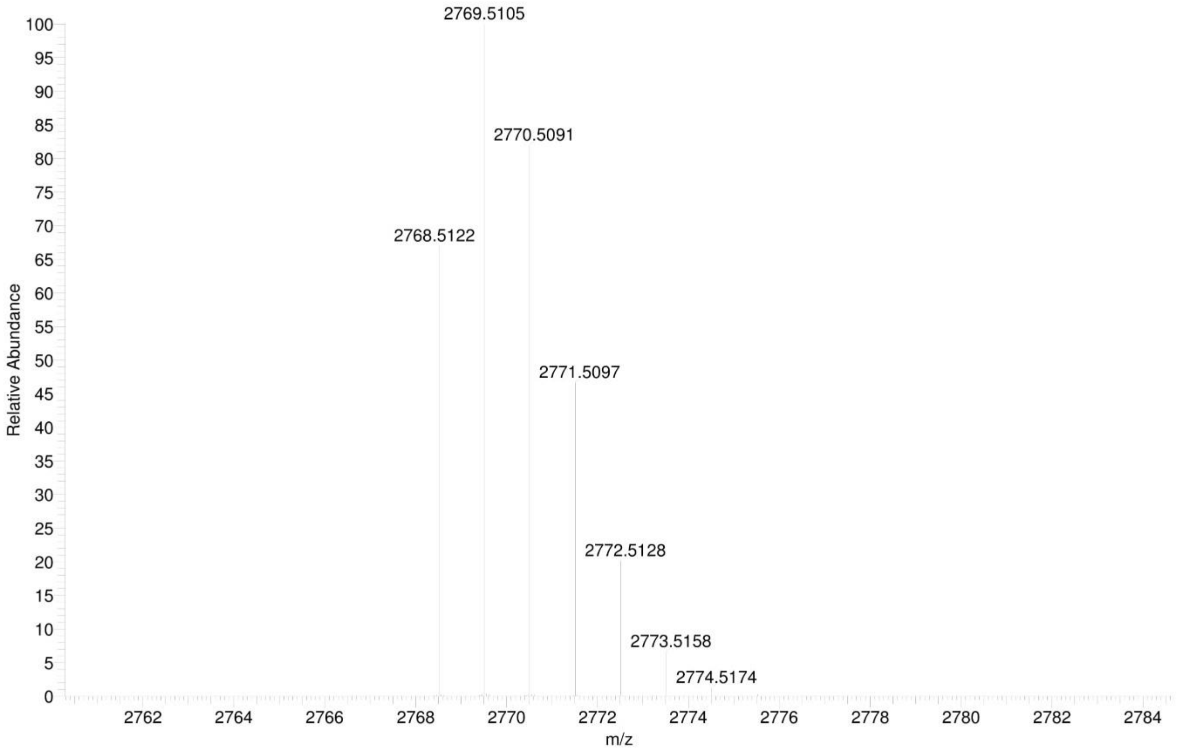

Ac**18** **((AcKAE)$_4$(*K*AEK)$_2$*K*YYA-OH)** was obtained after manual synthesis as foamy colourless solid after preparative RP-HPLC (4.1 mg, 9.4 µmol, 34.1%). Analytical RP-HPLC: t_R=1.29 min (100% A to 100% D in 5 min, λ= 214 nm). HRMS (ESI+): $C_{131}H_{213}N_{33}O_{43}$ calc./obs. 2936.5495/2936.5543 Da [M].

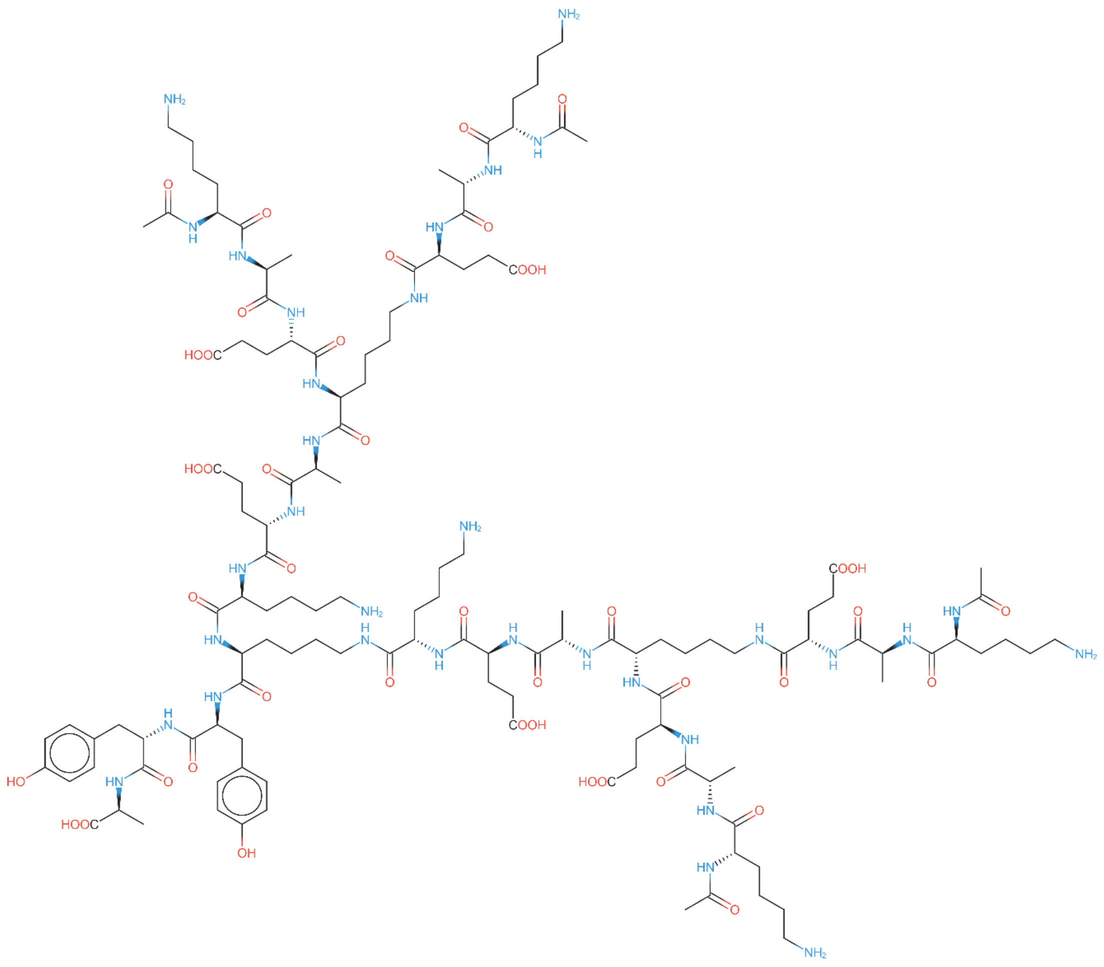

Analytical RP-HPLC:

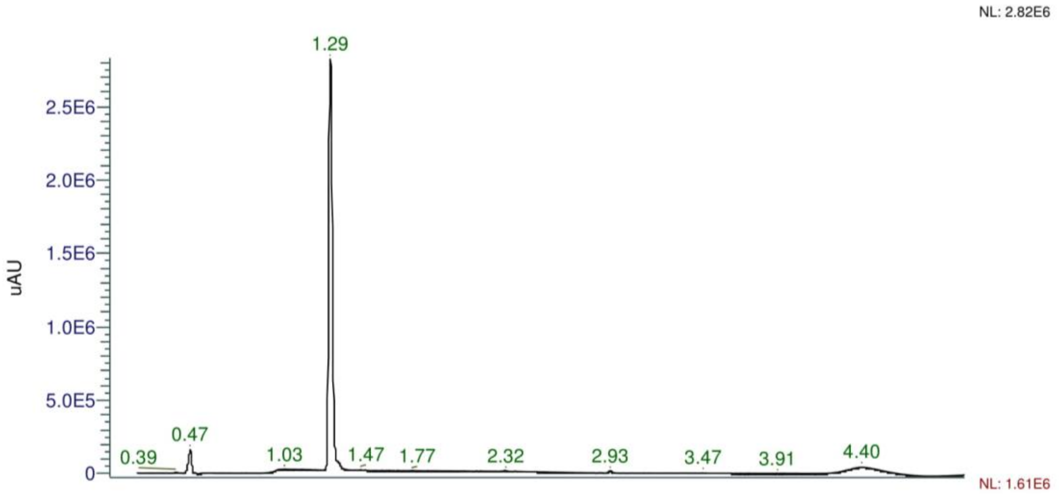

HRMS (NSI+):

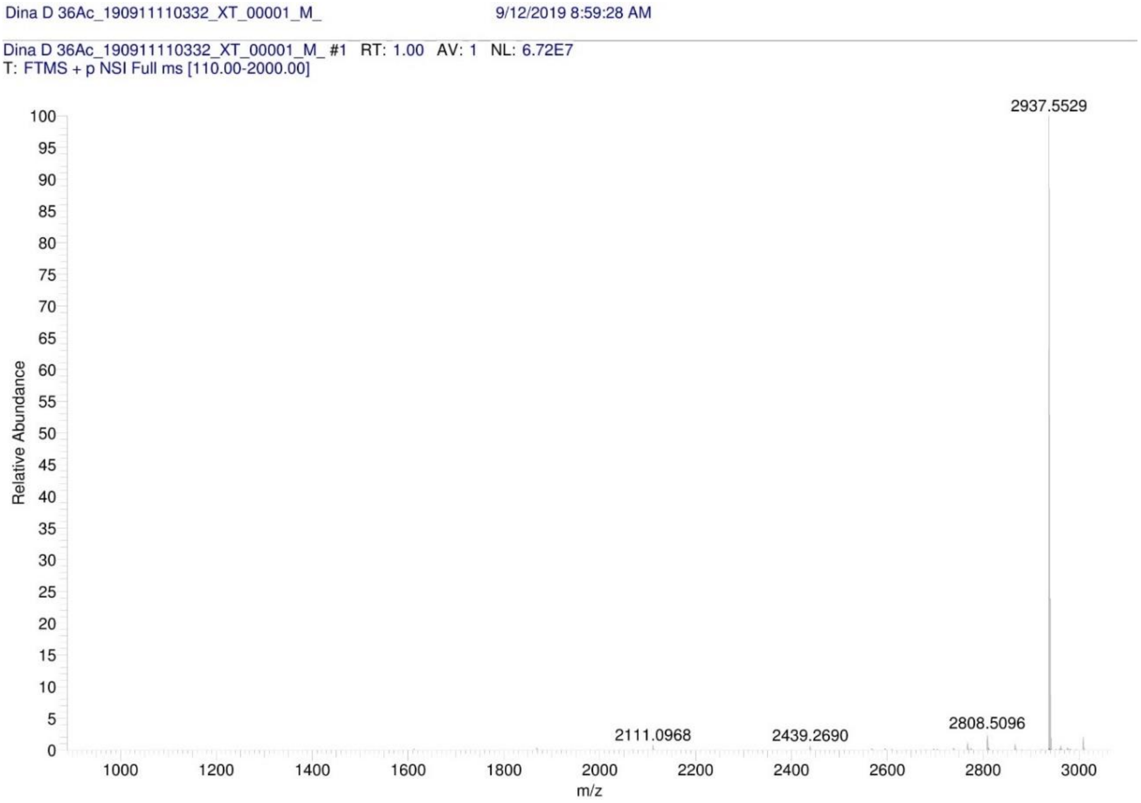

Dina D 36Ac_190911110332_XT_00001_M_
9/12/2019 8:59:28 AM
Dina D 36Ac_190911110332_XT_00001_M_ #1 RT: 1.00 AV: 1 NL: 6.72E7
T: FTMS + p NSI Full ms [110.00-2000.00]
2937.5529
2111.0968
2439.2690
2808.5096
Relative Abundance
m/z

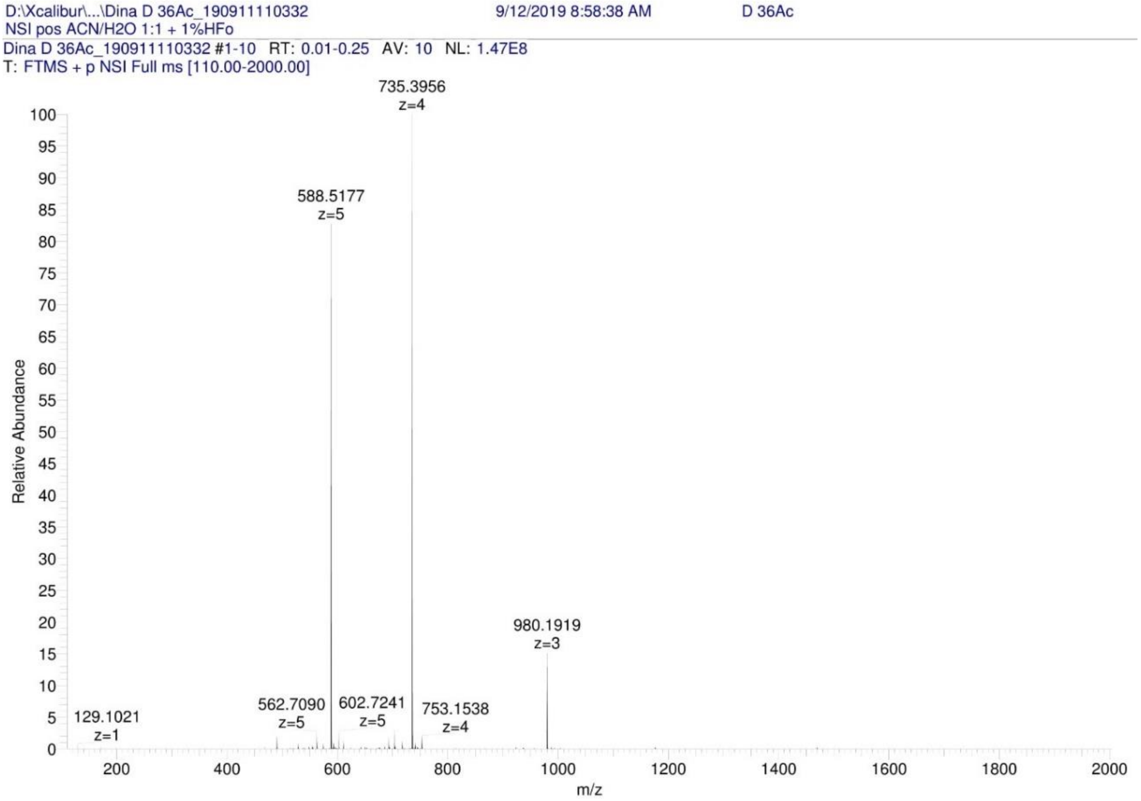

D:\Xcalibur\...\Dina D 36Ac_190911110332
9/12/2019 8:58:38 AM
D 36Ac
NSI pos ACN/H2O 1:1 + 1%HFo
Dina D 36Ac_190911110332 #1-10 RT: 0.01-0.25 AV: 10 NL: 1.47E8
T: FTMS + p NSI Full ms [110.00-2000.00]
735.3956 z=4
588.5177 z=5
980.1919 z=3
129.1021 z=1
562.7090 z=5
602.7241 z=5
753.1538 z=4
Relative Abundance
m/z

Dina D 36Ac_190911110332_XT_00001_M_ 9/12/2019 8:59:28 AM

Dina D 36Ac_190911110332_XT_00001_M_ #1 RT: 1.00 AV: 1 NL: 6.72E7
T: FTMS + p NSI Full ms [110.00-2000.00]

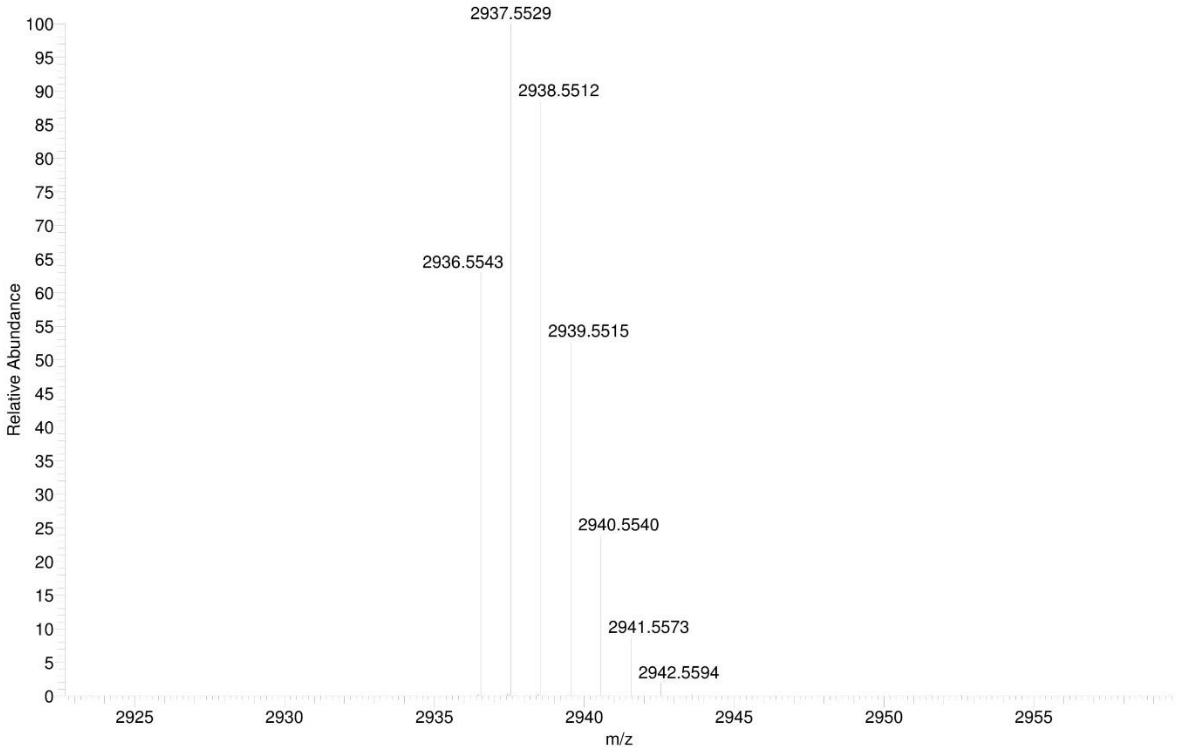

ClAc**18 ((ClAcKAE)$_4$(*K*AEK)$_2$*K*YYA-OH)** was obtained after manual synthesis as foamy colourless solid after preparative RP-HPLC (91.6 mg, 8.9 µmol, 33.8%7). Analytical RP-HPLC: t_R=1.35 min (100% A to 100% D in 5 min, λ= 214 nm). HRMS (ESI+): $C_{131}H_{209}Cl_4N_{33}O_{43}$ calc./obs. 3072.3936/3072.3961 Da [M].

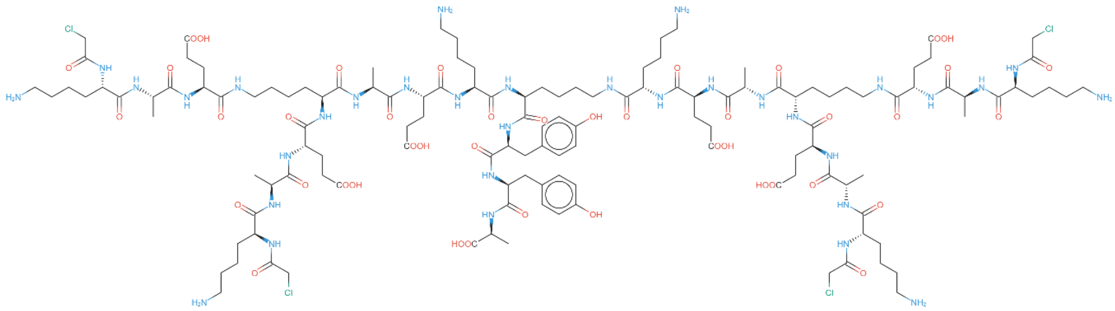

Analytical RP-HPLC:

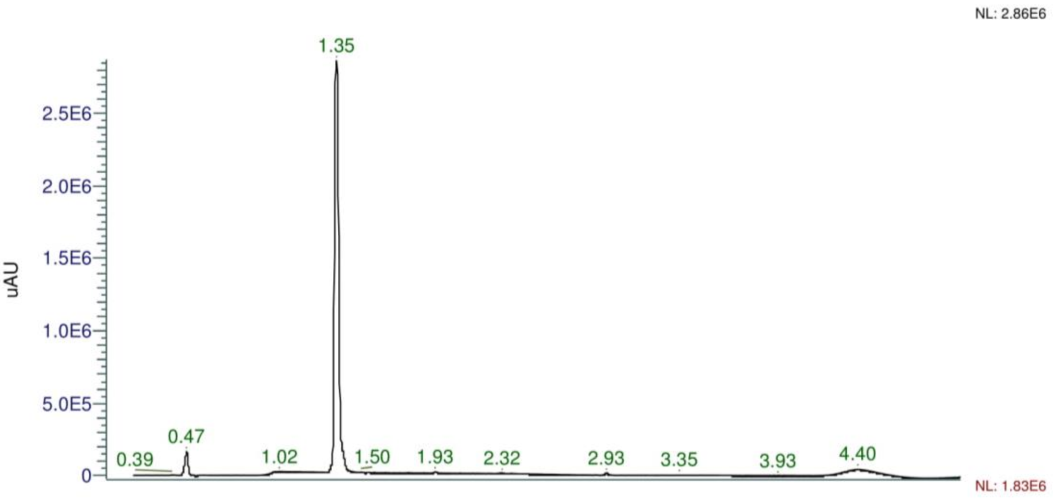

HRMS (NSI+):

Dina D 36Cl_190911110332_XT_00001_M_ 9/12/2019 9:03:04 AM

Dina D 36Cl_190911110332_XT_00001_M_ #1 RT: 1.00 AV: 1 NL: 1.18E8
T: FTMS + p NSI Full ms [110.00-2000.00]

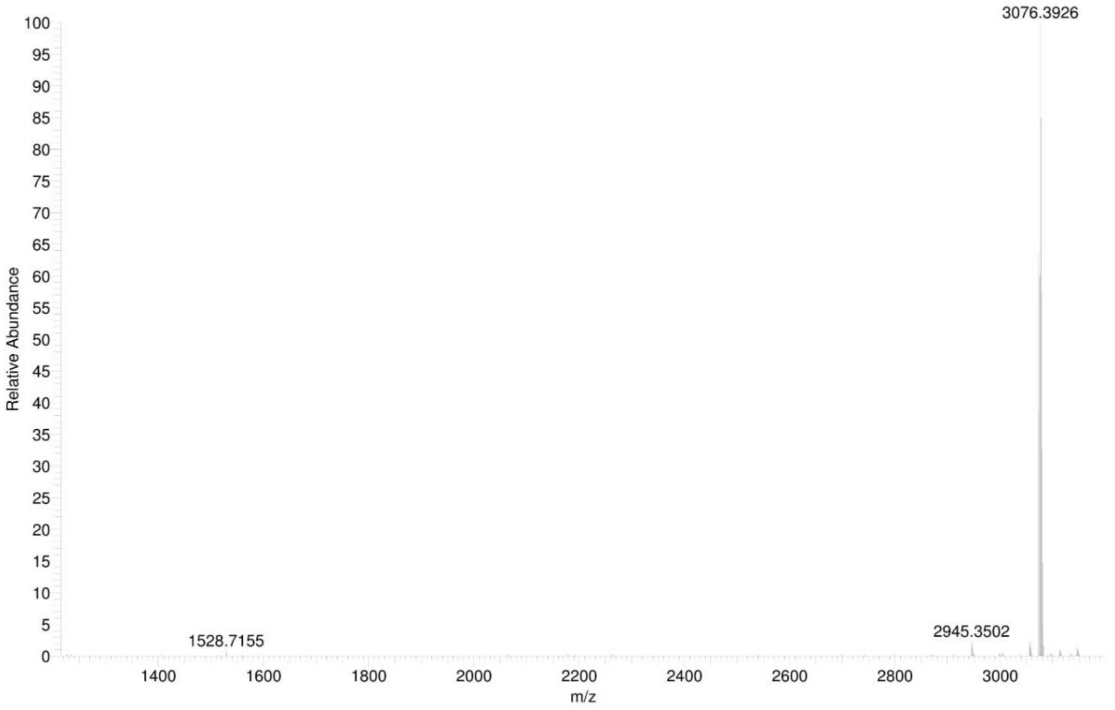

D:\Xcalibur\...\Dina D 36Cl_190911110332 9/12/2019 9:02:18 AM D 36Cl
NSI pos ACN/H2O 1:1 + 1%HFo
Dina D 36Cl_190911110332 #9 RT: 0.24 AV: 1 NL: 2.40E8
T: FTMS + p NSI Full ms [110.00-2000.00]

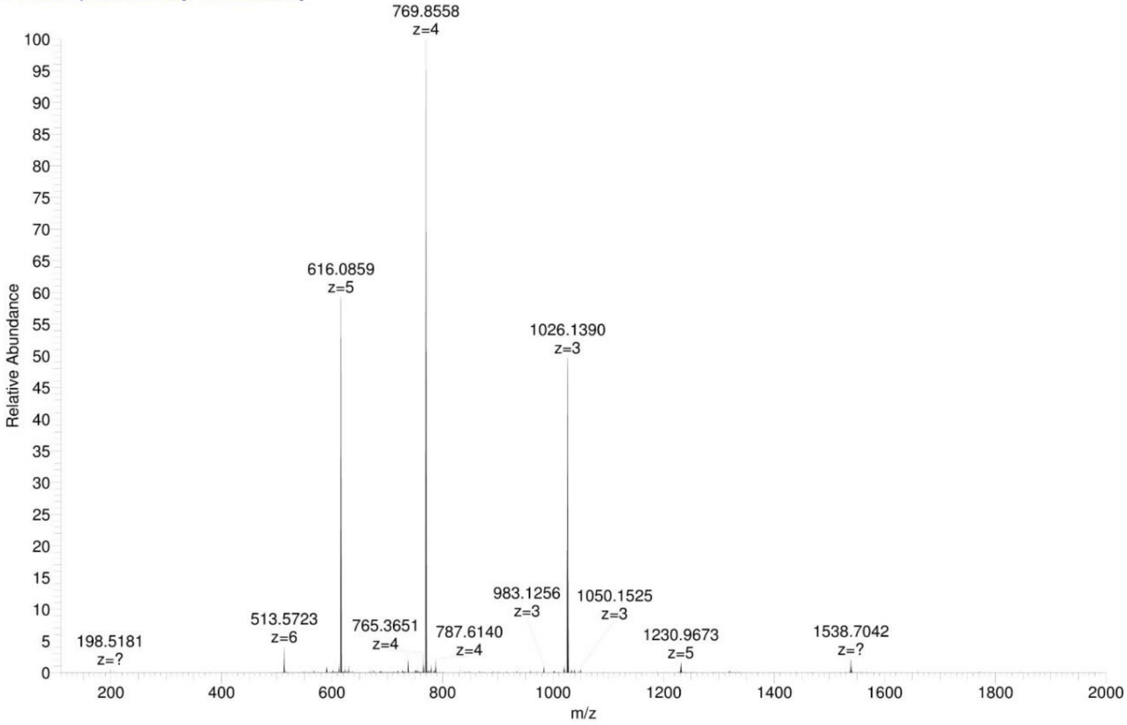

Dina D 36Cl_190911110332_XT_00001_M_ 9/12/2019 9:03:04 AM

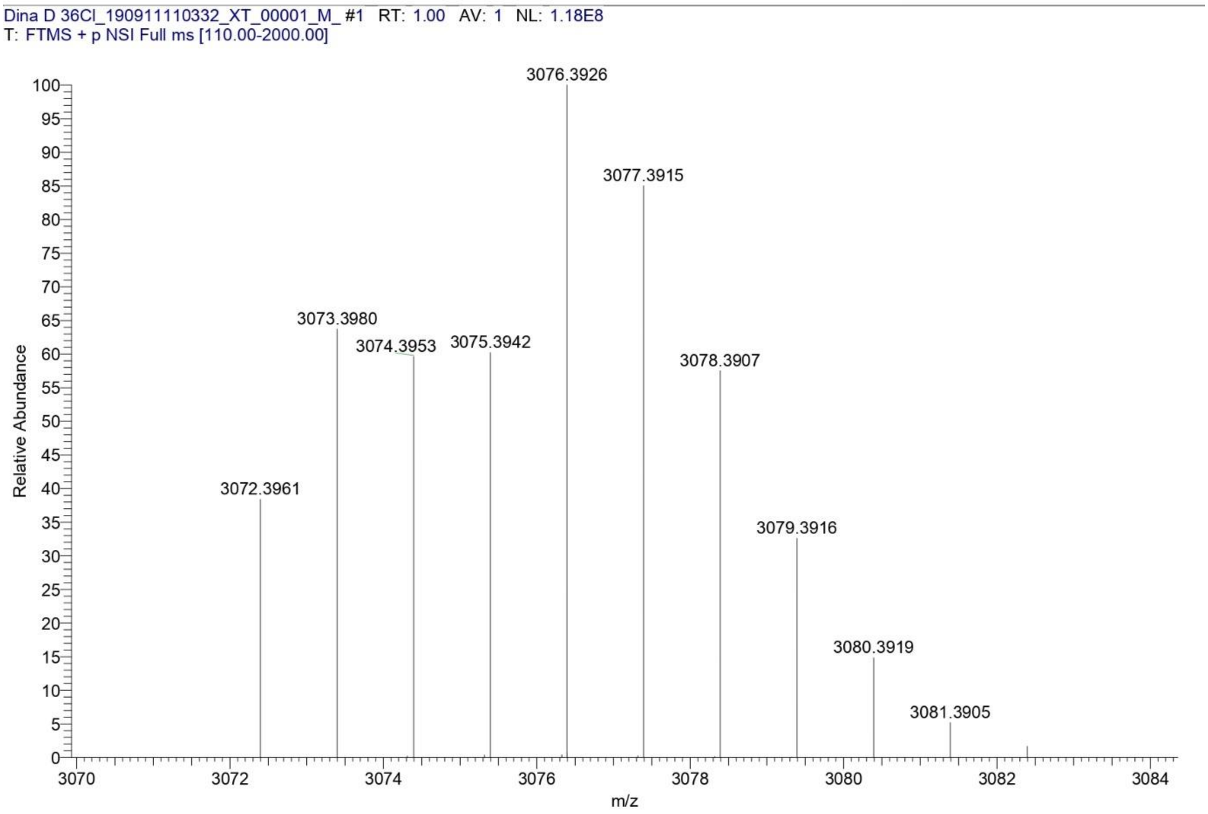

Ac**19** **((AcKAA)$_4$(*K*YYE)$_2$*K*AY-NH$_2$)** was obtained after manual synthesis as foamy colourless solid after preparative RP-HPLC (31.7 mg, 9.1 µmol, 29.5%). Analytical RP-HPLC: t_R=1.47 min (100% A to 100% D in 5 min, λ= 214 nm). HRMS (ESI+): $C_{132}H_{199}N_{31}O_{36}$ calc./obs. 2794.4694/2794.4734 Da [M].

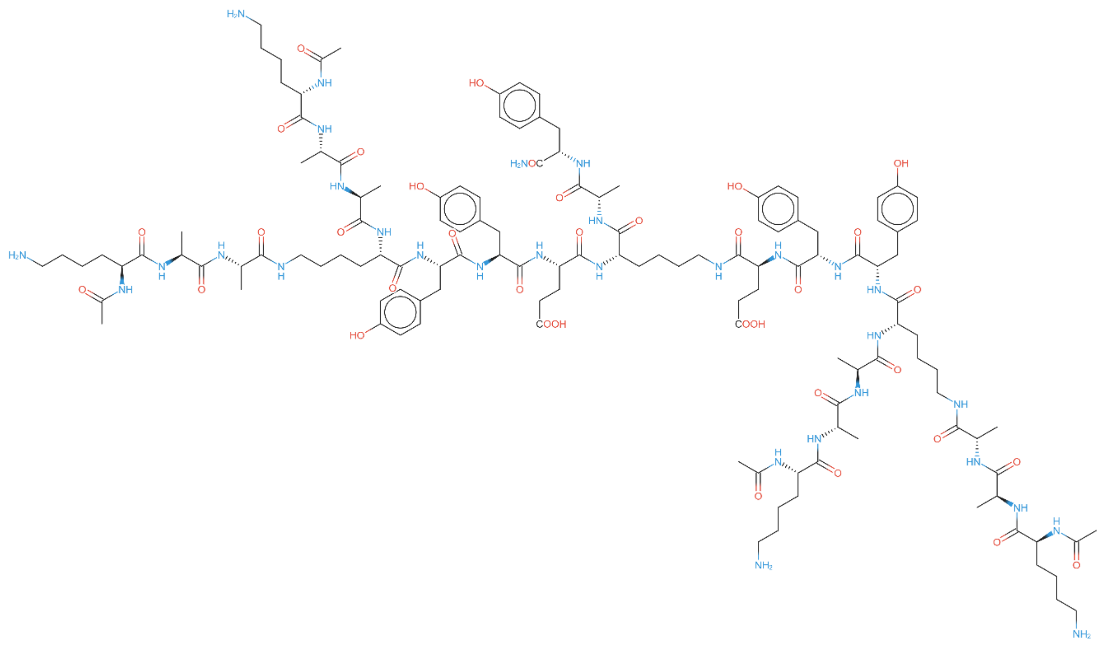

Analytical RP-HPLC:

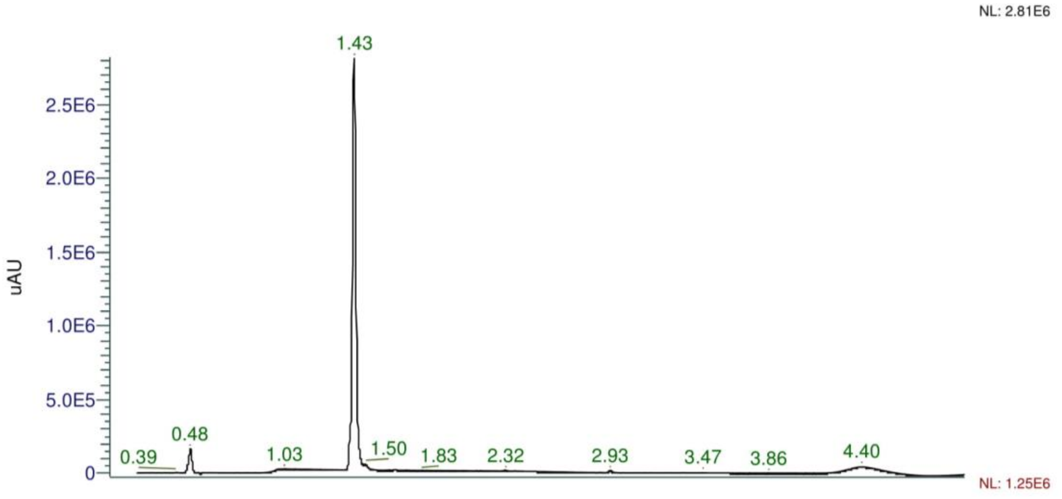

HRMS (NSI+):

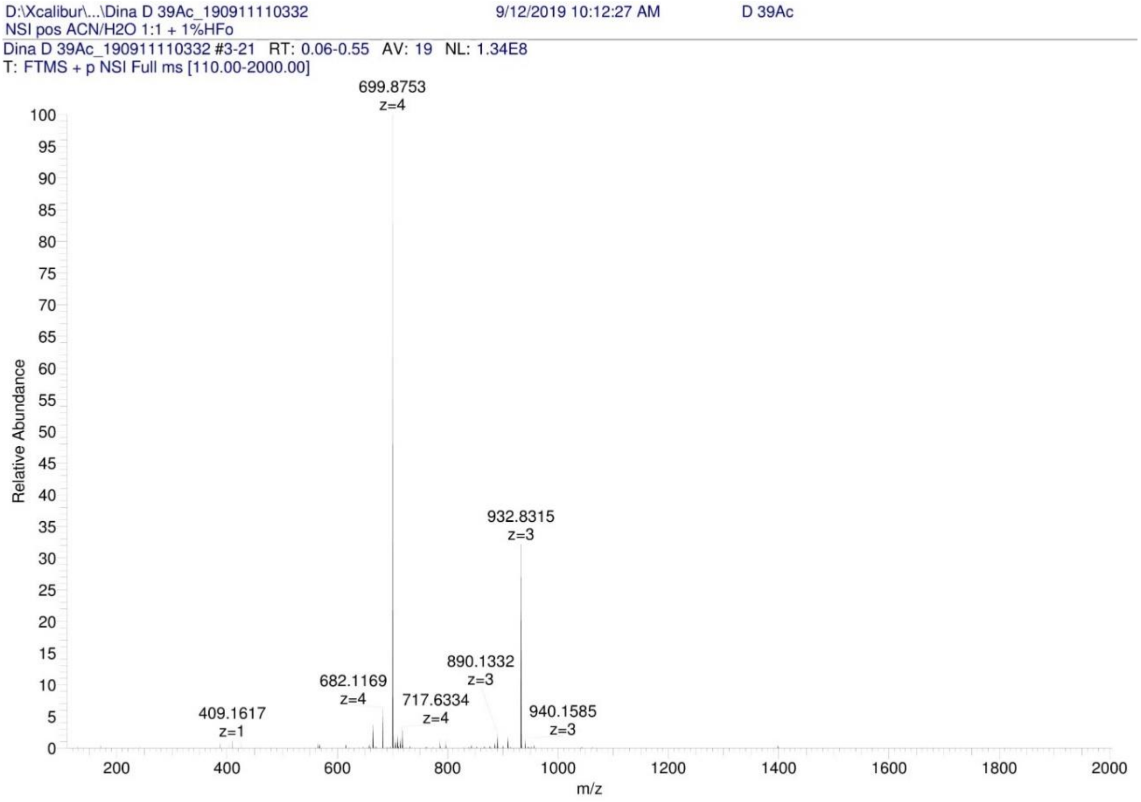
D:\Xcalibur\...\Dina D 39Ac_190911110332
9/12/2019 10:12:27 AM
D 39Ac
NSI pos ACN/H2O 1:1 + 1%HFo
Dina D 39Ac_190911110332 #3-21 RT: 0.06-0.55 AV: 19 NL: 1.34E8
T: FTMS + p NSI Full ms [110.00-2000.00]
699.8753
z=4
932.8315
z=3
890.1332
z=3
682.1169
z=4
717.6334
z=4
409.1617
z=1
940.1585
z=3
Relative Abundance
m/z

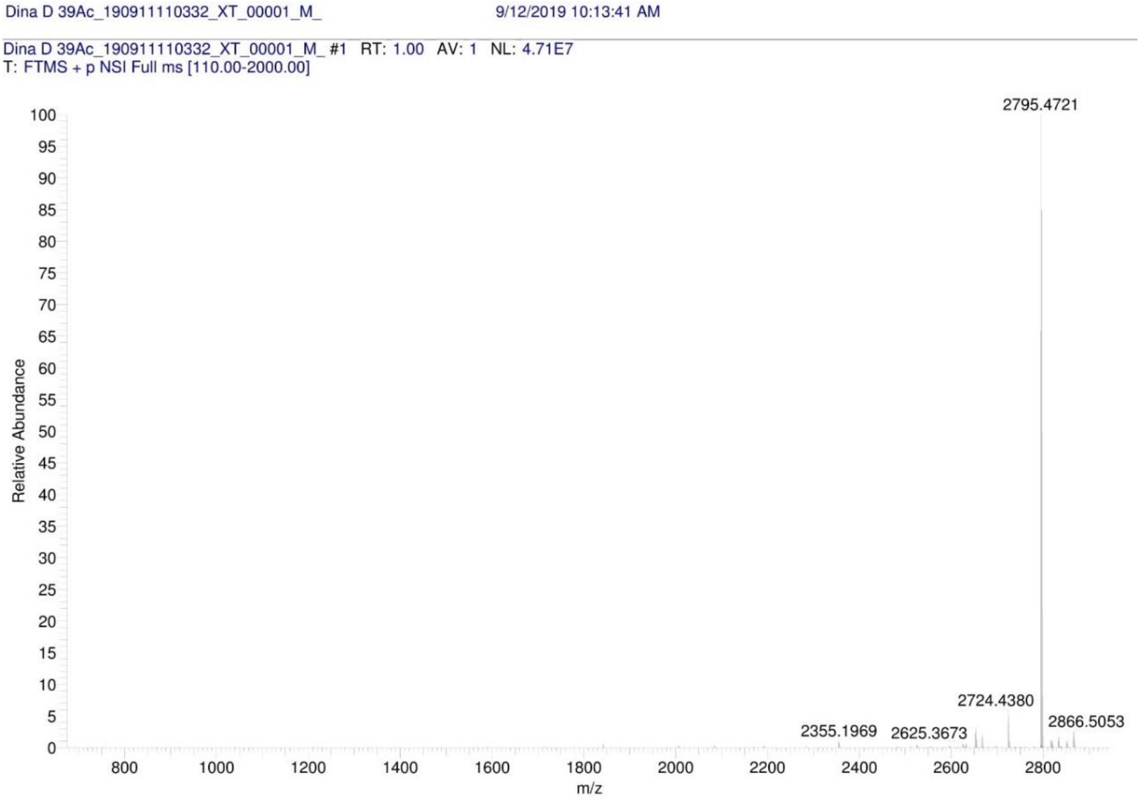
Dina D 39Ac_190911110332_XT_00001_M_
9/12/2019 10:13:41 AM
Dina D 39Ac_190911110332_XT_00001_M_ #1 RT: 1.00 AV: 1 NL: 4.71E7
T: FTMS + p NSI Full ms [110.00-2000.00]
2795.4721
2724.4380
2355.1969
2625.3673
2866.5053
Relative Abundance
m/z

Dina D 39Ac_190911110332_XT_00001_M_ 9/12/2019 10:13:41 AM

Dina D 39Ac_190911110332_XT_00001_M_ #1 RT: 1.00 AV: 1 NL: 4.71E7
T: FTMS + p NSI Full ms [110.00-2000.00]

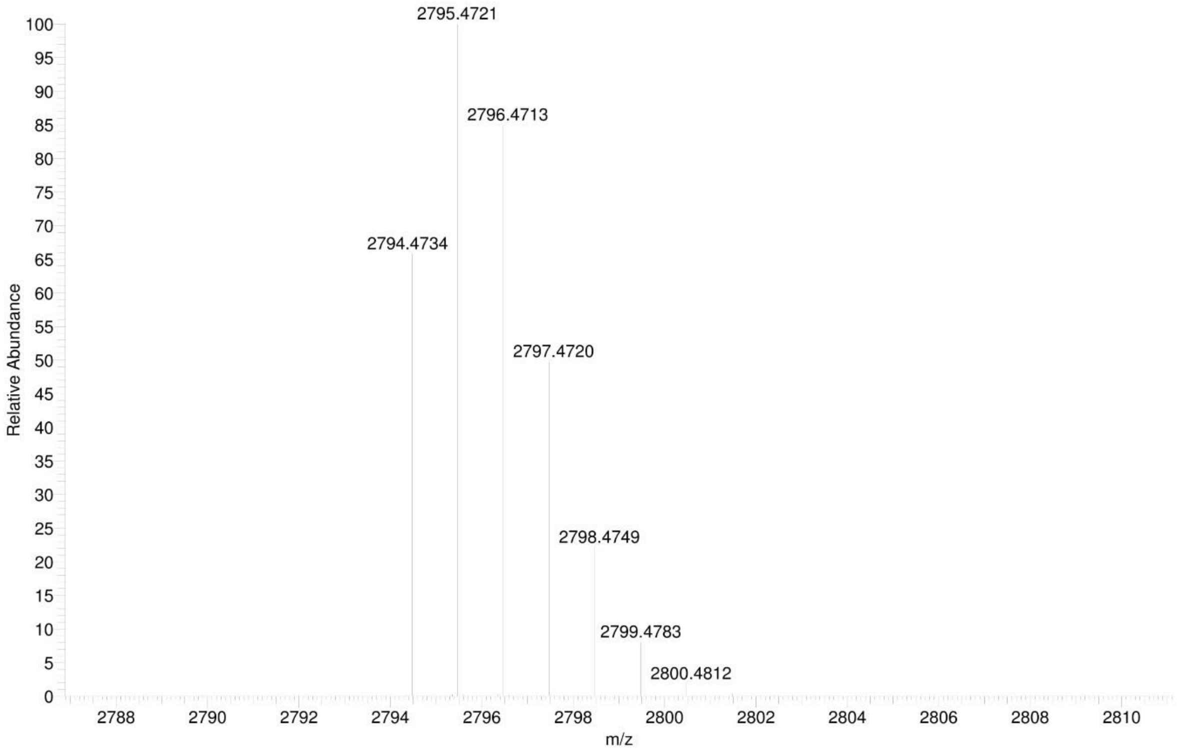

ClAc**19 ((ClAcKAA)$_4$(*K*YYE)$_2$*K*AY-NH$_2$)** was obtained after manual synthesis as foamy colourless solid after preparative RP-HPLC (29.4 mg, 7.8 µmol, 26.3%). Analytical RP-HPLC: t_R=1.51 min (100% A to 100% D in 5 min, λ= 214 nm). HRMS (ESI+): $C_{132}H_{195}Cl_4N_{31}O_{36}$ calc./obs. 2930.3135/2930.3170 Da [M].

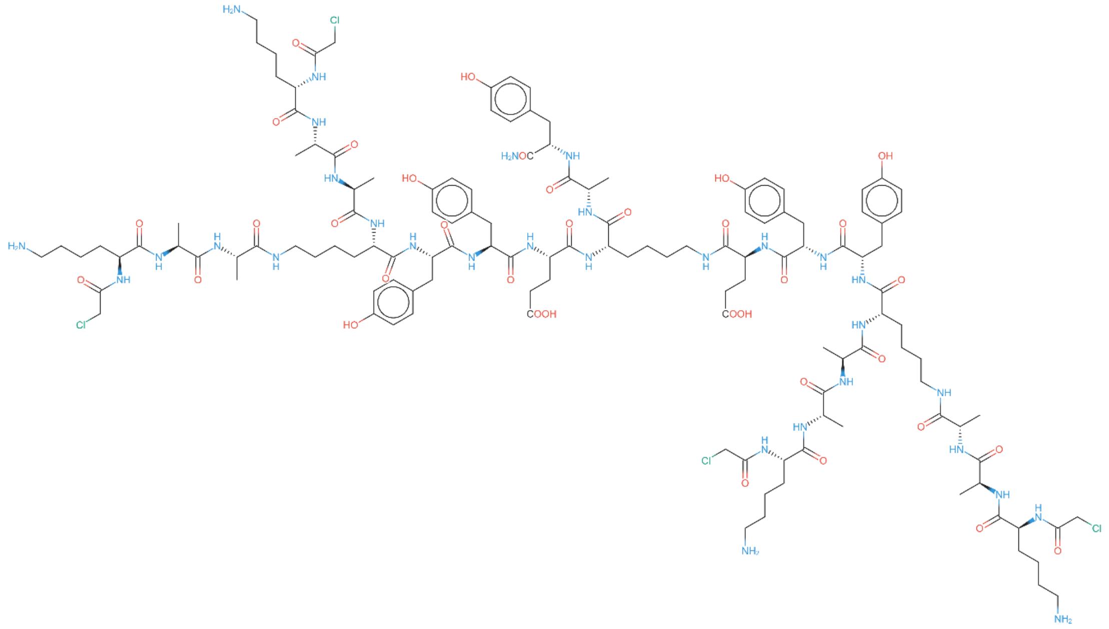

Analytical RP-HPLC:

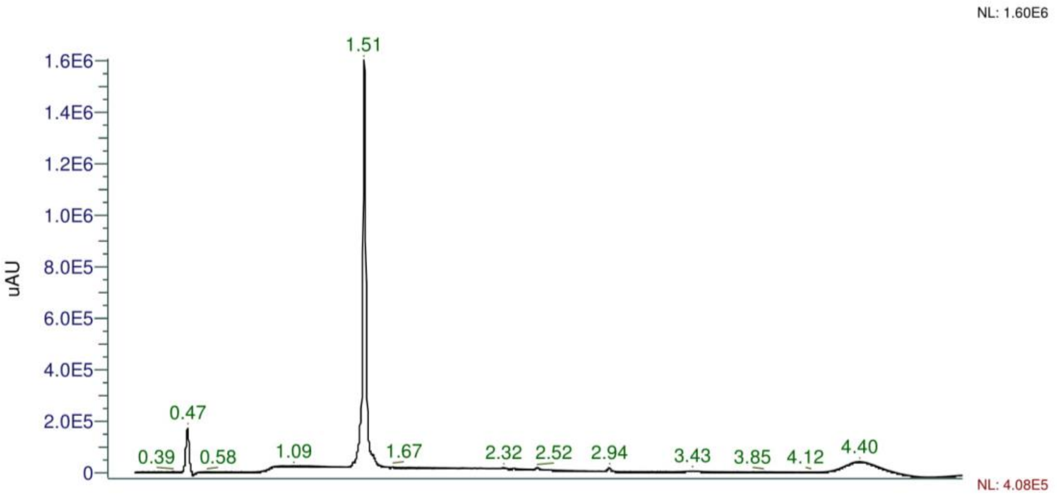

HRMS (NSI+):

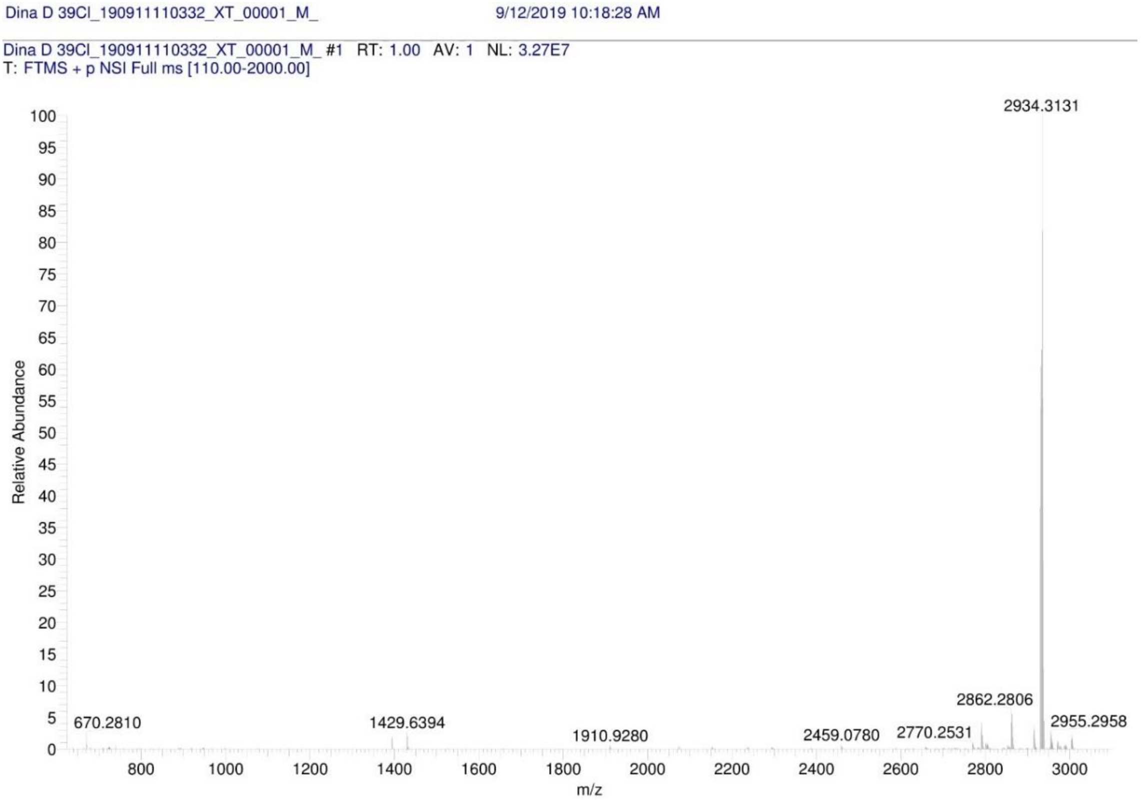
Dina D 39Cl_190911110332_XT_00001_M_
9/12/2019 10:18:28 AM
Dina D 39Cl_190911110332_XT_00001_M_ #1 RT: 1.00 AV: 1 NL: 3.27E7
T: FTMS + p NSI Full ms [110.00-2000.00]
2934.3131
2862.2806
2955.2958
670.2810
1429.6394
1910.9280
2459.0780
2770.2531
Relative Abundance
m/z

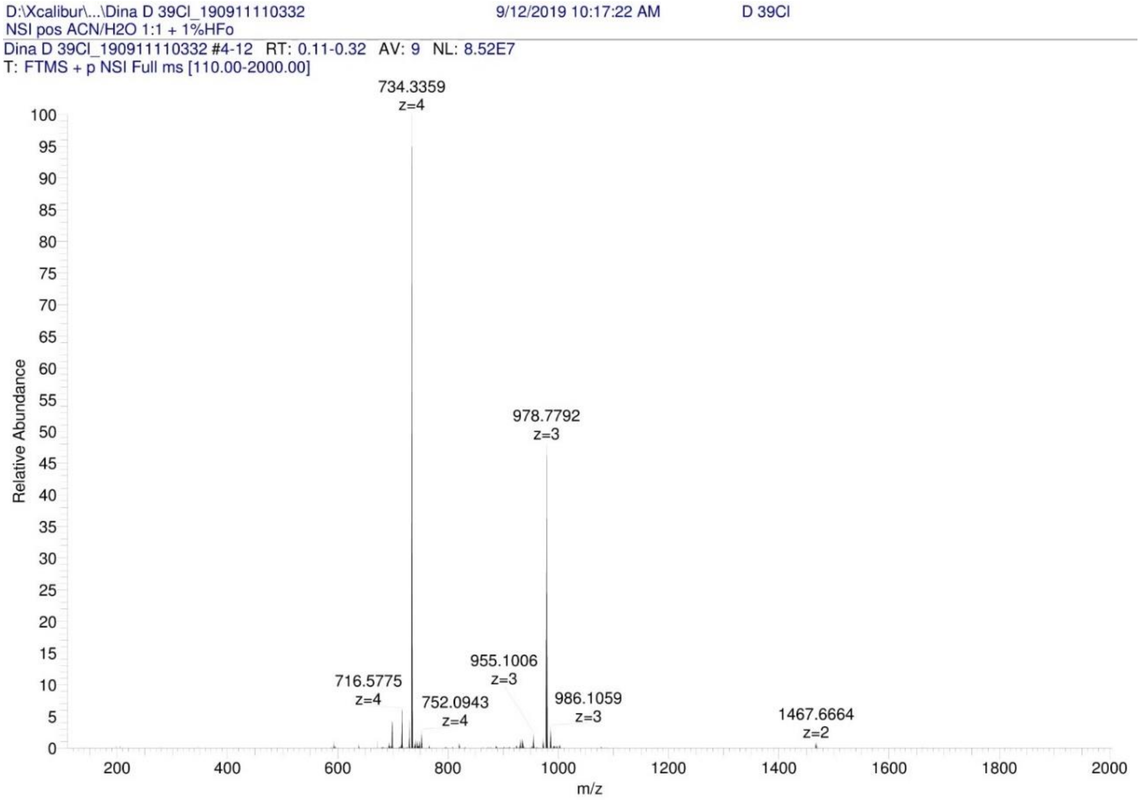
D:\Xcalibur\...\Dina D 39Cl_190911110332
9/12/2019 10:17:22 AM
D 39Cl
NSI pos ACN/H2O 1:1 + 1%HFo
Dina D 39Cl_190911110332 #4-12 RT: 0.11-0.32 AV: 9 NL: 8.52E7
T: FTMS + p NSI Full ms [110.00-2000.00]
734.3359 z=4
978.7792 z=3
955.1006 z=3
716.5775 z=4
752.0943 z=4
986.1059 z=3
1467.6664 z=2
Relative Abundance
m/z

Dina D 39Cl_190911110332_XT_00001_M_ 9/12/2019 10:18:28 AM

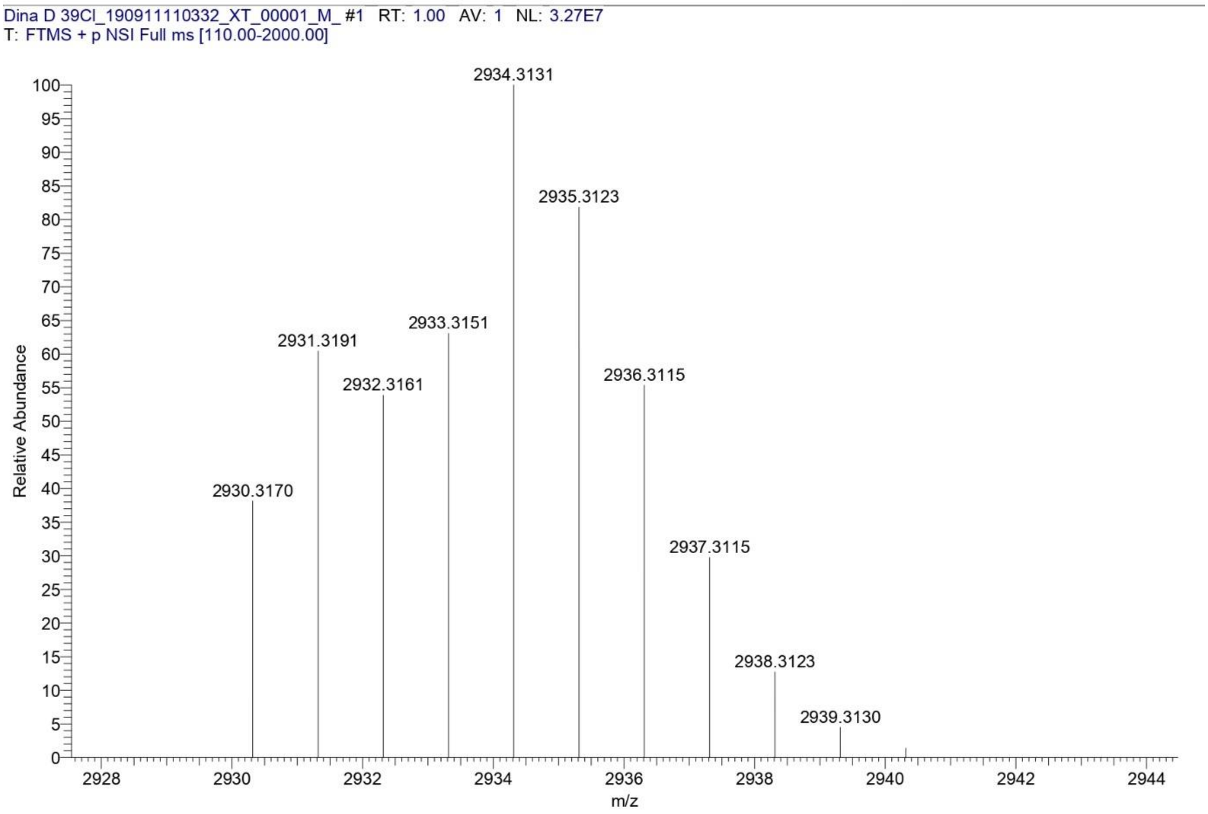

Ac**20 ((AcKAK)$_4$(*K*AEY)$_2$*K*AEA-OH)** was obtained from the CEM Liberty Blue synthetiser as foamy colourless solid after preparative RP-HPLC (3.0 mg, 0.7 µmol, 2.3%). Analytical RP-HPLC: t_R=1.27 min (100% A to 100% D in 5 min, λ= 214 nm). HRMS (ESI+): $C_{131}H_{221}N_{35}O_{37}$ calc./obs. 2876.6488/2876.6463 Da [M].

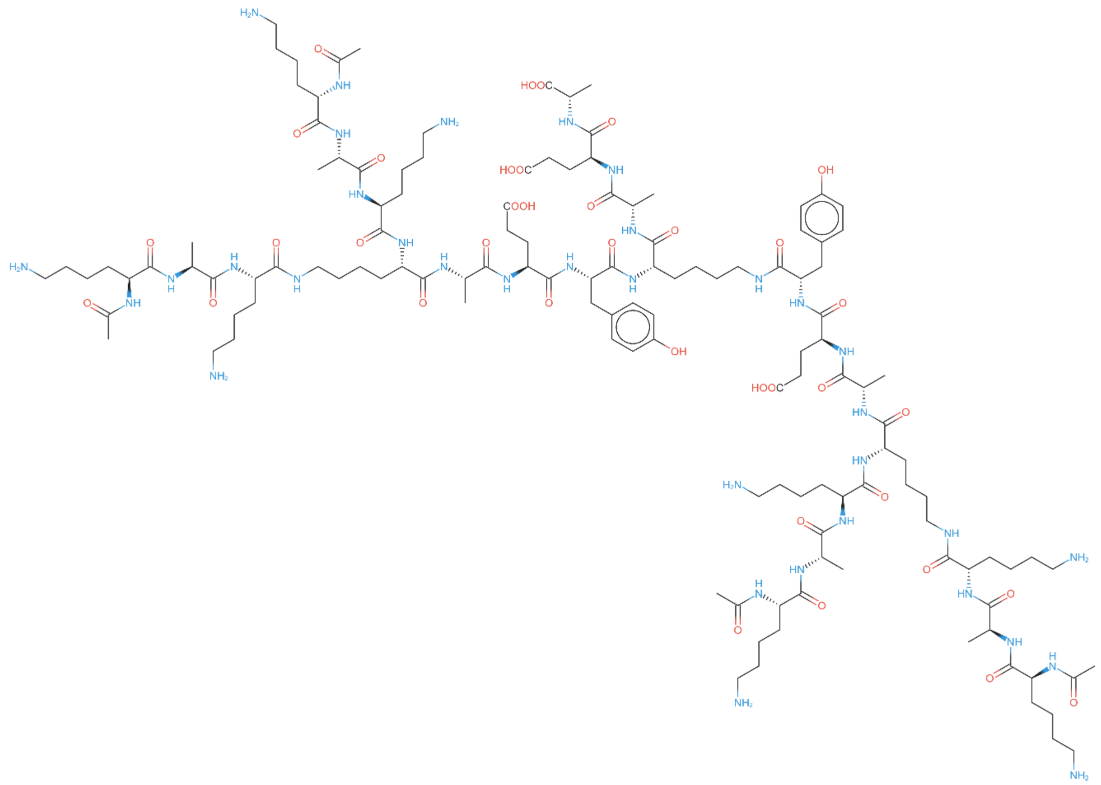

Analytical RP-HPLC:

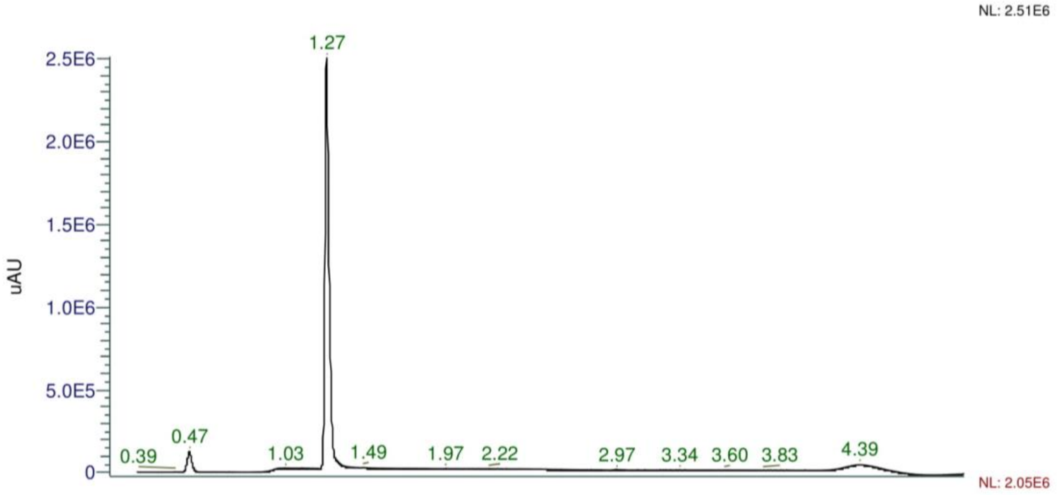

HRMS (NSI+):

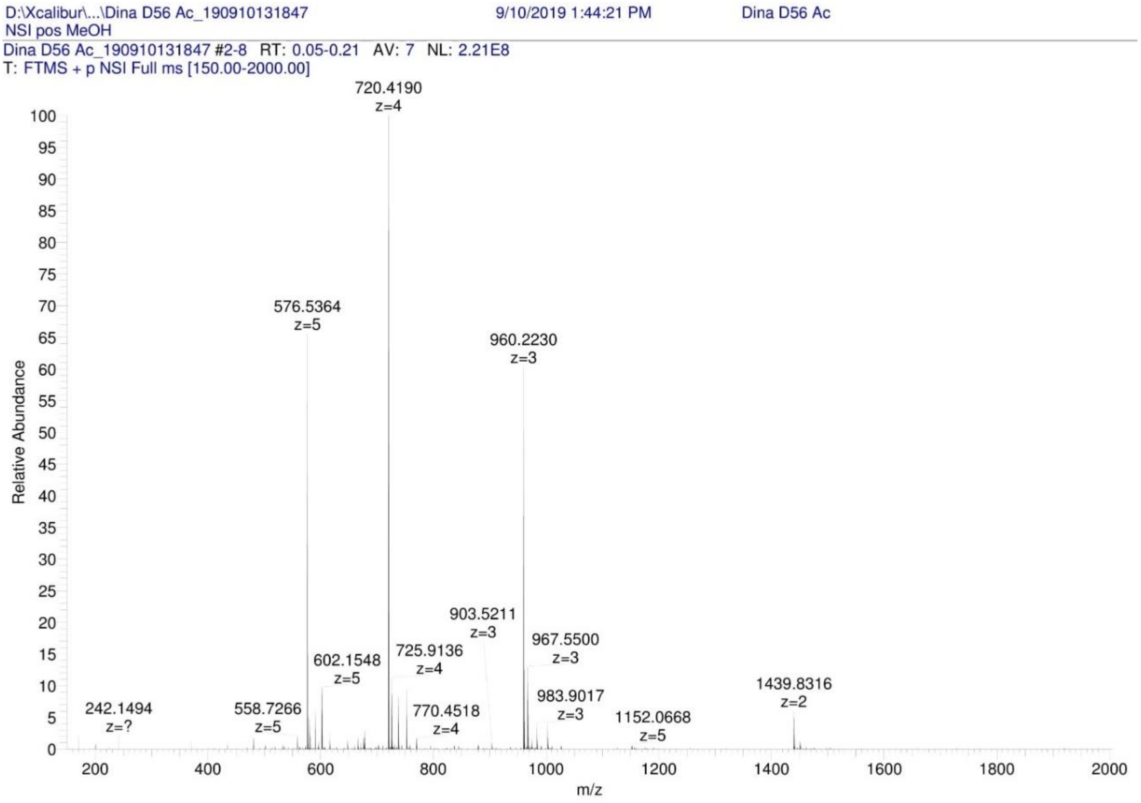
D:\Xcalibur\...\Dina D56 Ac_190910131847
9/10/2019 1:44:21 PM
Dina D56 Ac
NSI pos MeOH
Dina D56 Ac_190910131847 #2-8 RT: 0.05-0.21 AV: 7 NL: 2.21E8
T: FTMS + p NSI Full ms [150.00-2000.00]
720.4190 z=4
576.5364 z=5
960.2230 z=3
903.5211 z=3
967.5500 z=3
602.1548 z=5
725.9136 z=4
1439.8316 z=2
983.9017 z=3
242.1494 z=?
558.7266 z=5
770.4518 z=4
1152.0668 z=5
Relative Abundance
m/z

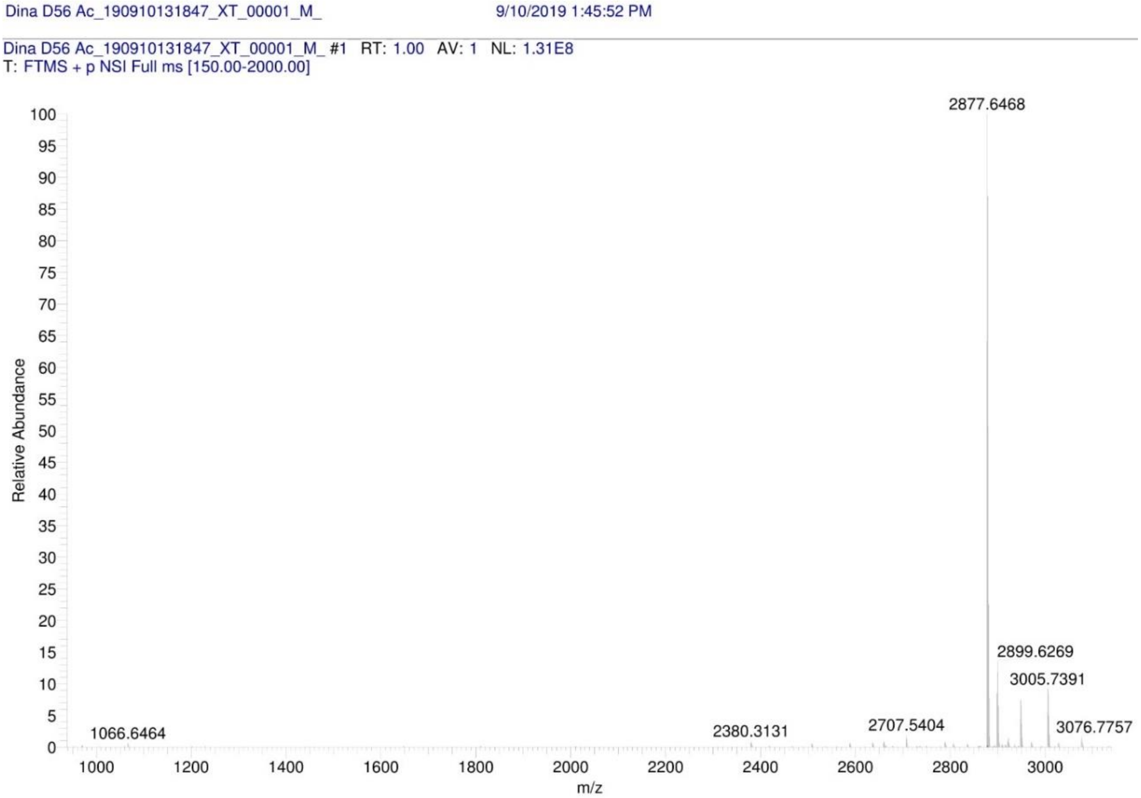
Dina D56 Ac_190910131847_XT_00001_M_
9/10/2019 1:45:52 PM
Dina D56 Ac_190910131847_XT_00001_M_ #1 RT: 1.00 AV: 1 NL: 1.31E8
T: FTMS + p NSI Full ms [150.00-2000.00]
2877.6468
2899.6269
3005.7391
1066.6464
2380.3131
2707.5404
3076.7757
Relative Abundance
m/z

Dina D56 Ac_190910131847_XT_00001_M_ 9/10/2019 1:45:52 PM

Dina D56 Ac_190910131847_XT_00001_M_ #1 RT: 1.00 AV: 1 NL: 1.31E8
T: FTMS + p NSI Full ms [150.00-2000.00]

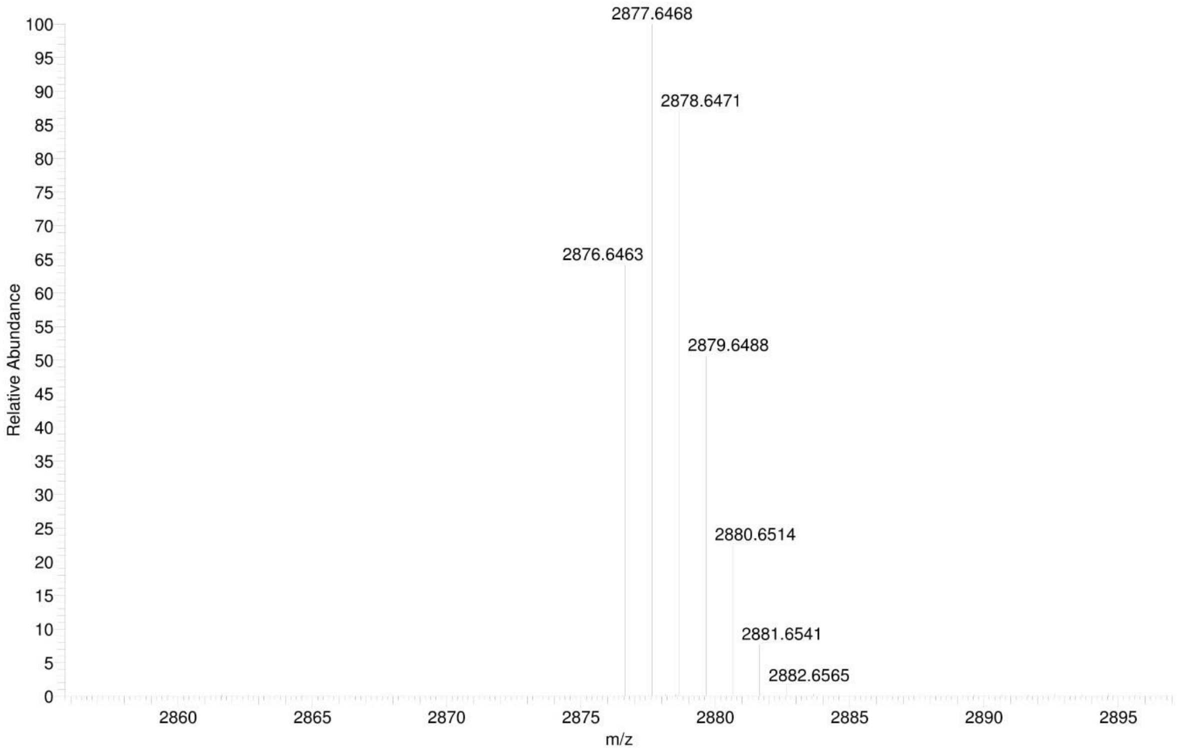

21 ((AKK)$_4$(*K*YEK)$_2$*K*KEA-NH$_2$) was obtained from the CEM Liberty Blue synthetiser as foamy colourless solid after preparative RP-HPLC (168.3 mg, 12.6 µmol, 49.3%). Analytical RP-HPLC: t_R=1.17 min (100% A to 100% D in 5 min, λ= 214 nm). HRMS (ESI+): $C_{120}H_{207}N_{35}O_{32}$ calc./obs. 2650.5646/2650.5678 Da [M].

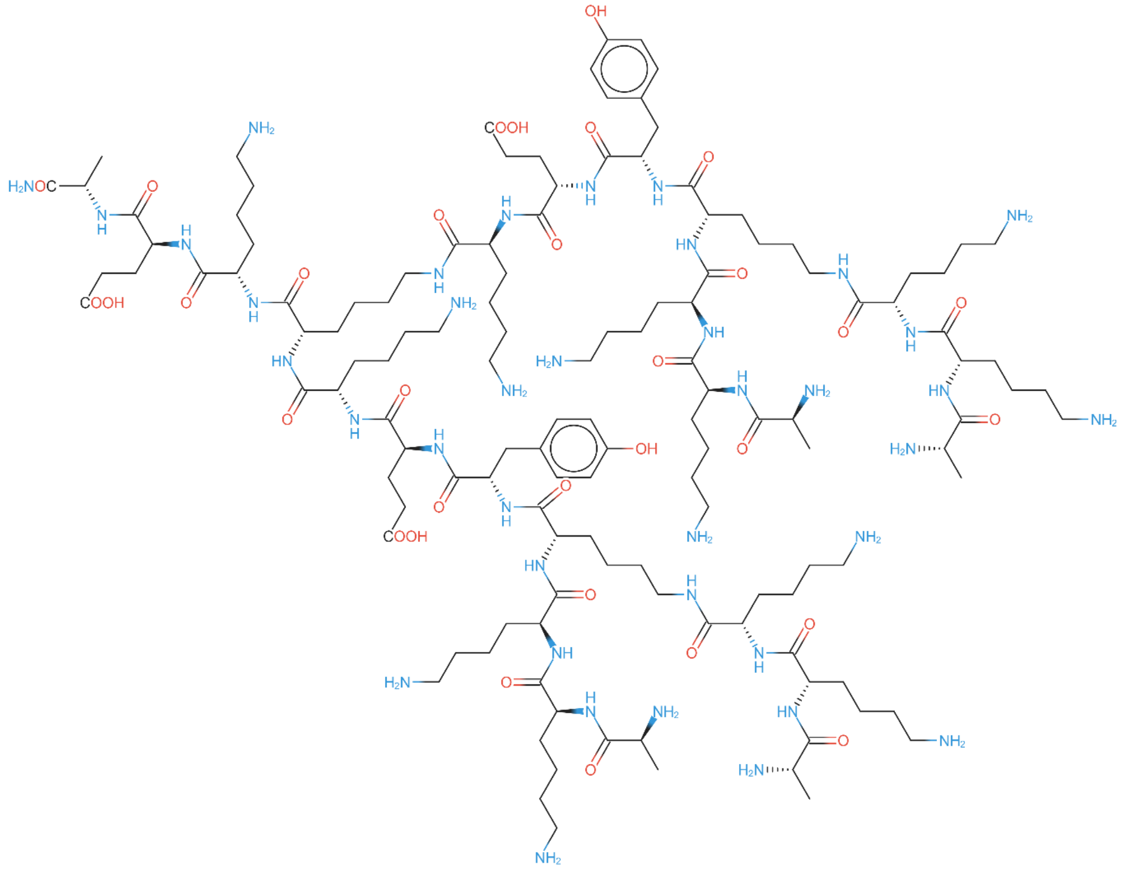

Analytical RP-HPLC:

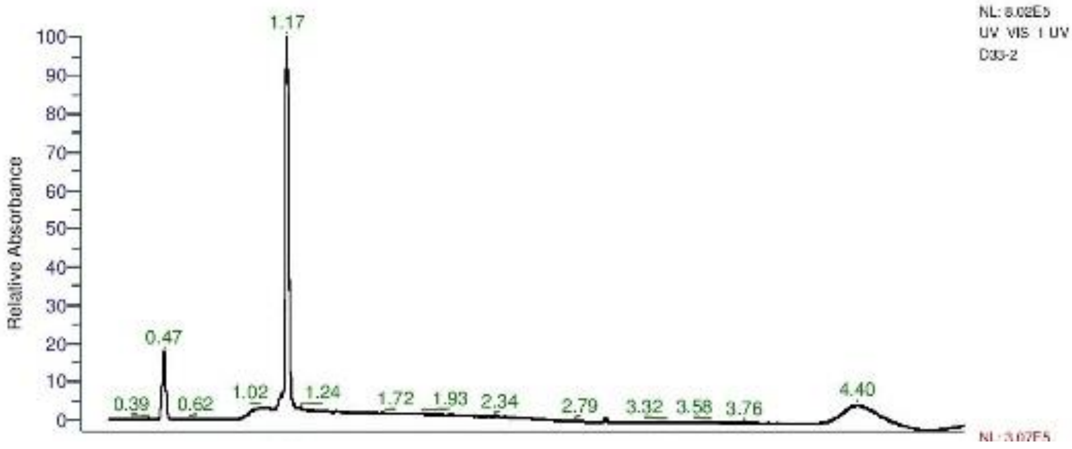

HRMS (NSI+):

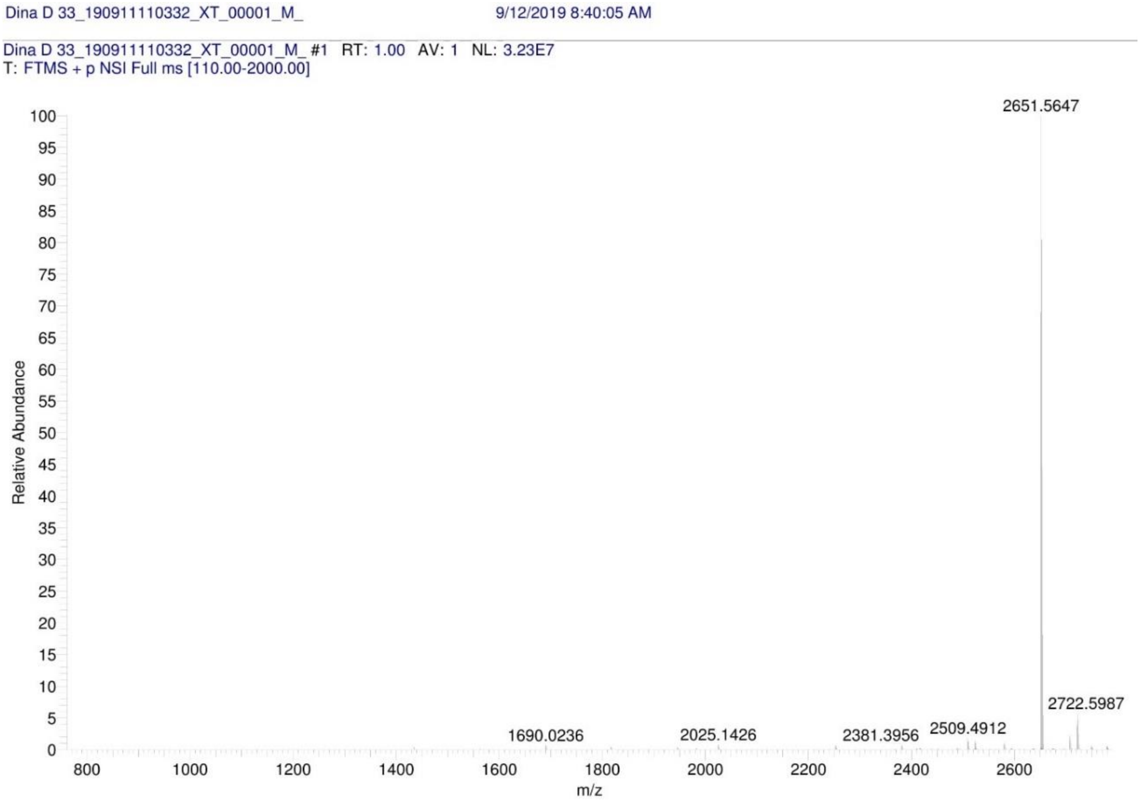
Dina D 33_190911110332_XT_00001_M_
9/12/2019 8:40:05 AM
Dina D 33_190911110332_XT_00001_M_ #1 RT: 1.00 AV: 1 NL: 3.23E7
T: FTMS + p NSI Full ms [110.00-2000.00]
2651.5647
2722.5987
1690.0236
2025.1426
2381.3956
2509.4912
Relative Abundance
m/z

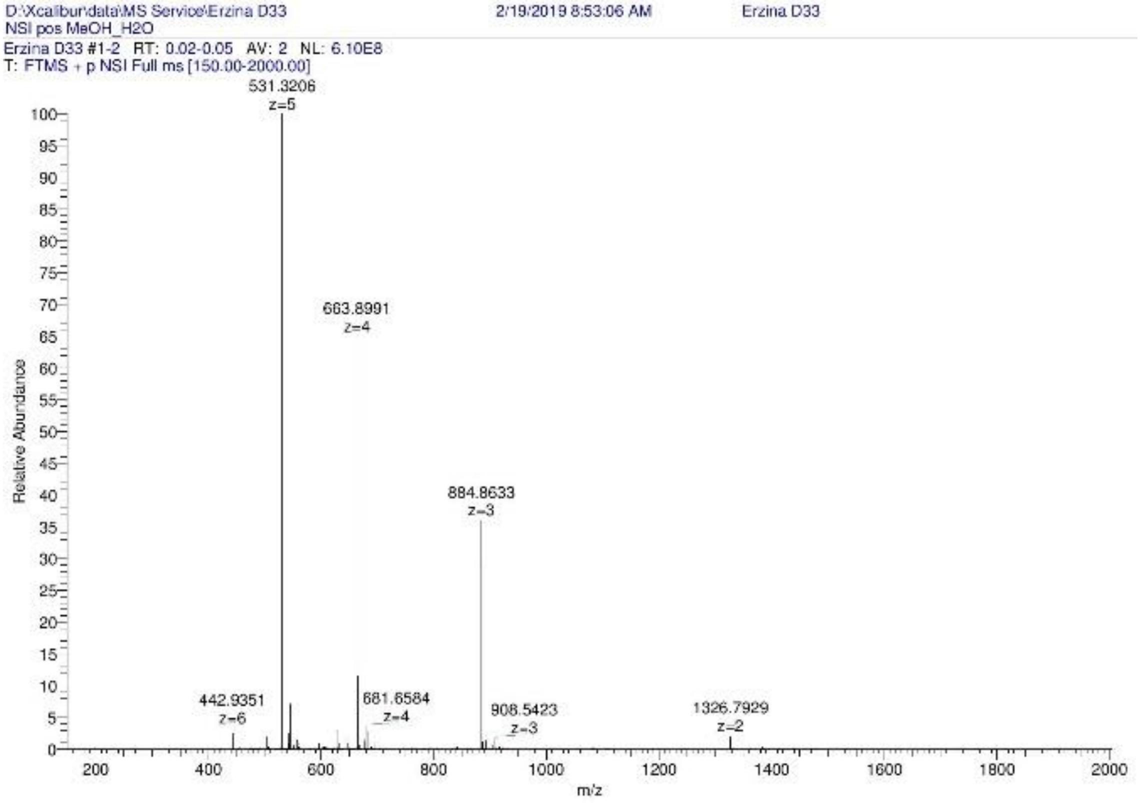
D:\Xcalibur\data\MS Service\Erzina D33
NSI pos MeOH_H2O
2/19/2019 8:53:06 AM
Erzina D33
Erzina D33 #1-2 RT: 0.02-0.05 AV: 2 NL: 6.10E8
T: FTMS + p NSI Full ms [150.00-2000.00]
531.3206
z=5
663.8991
z=4
884.8633
z=3
442.9351
z=6
681.6584
z=4
908.5423
z=3
1326.7929
z=2
Relative Abundance
m/z

Dina D 33_190911110332_XT_00001_M_ 9/12/2019 8:40:05 AM

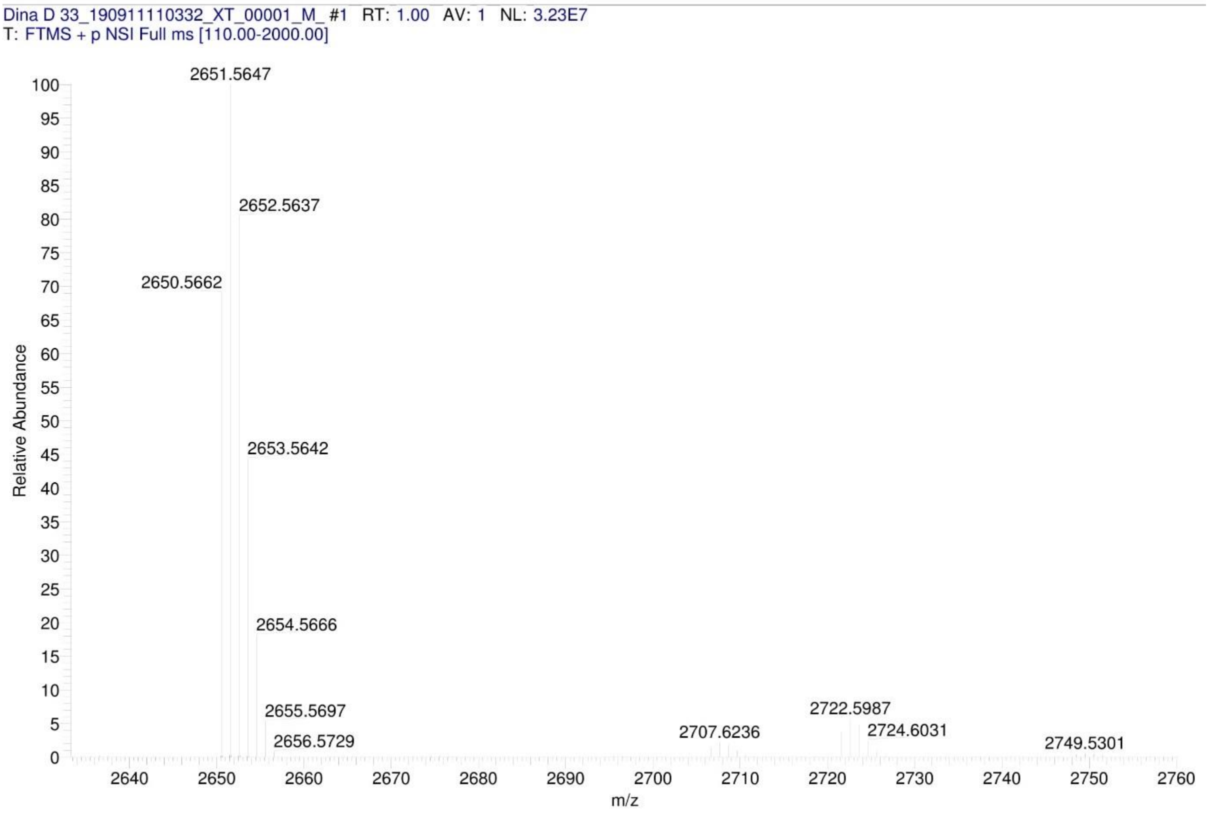

22 ((AKE)$_4$(*K*KAY)$_2$*K*YKY-NH$_2$) was obtained after manual synthesis as foamy colourless solid after preparative RP-HPLC (25.4 mg, 4.8 µmol, 18.6%). Analytical RP-HPLC: t_R=1.25 min (100% A to 100% D in 5 min, λ= 214 nm). HRMS (ESI+): $C_{134}H_{217}N_{35}O_{36}$ calc./obs. 2892.6225/2892.6243 Da [M].

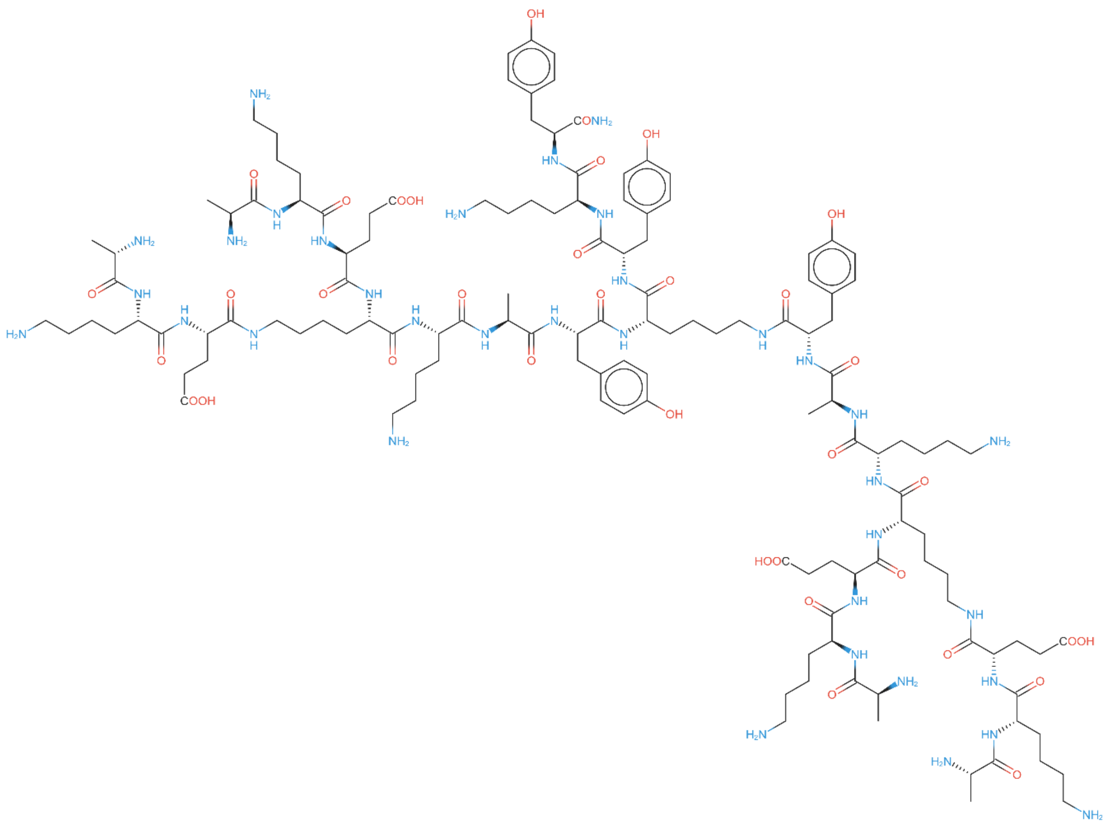

Analytical RP-HPLC:

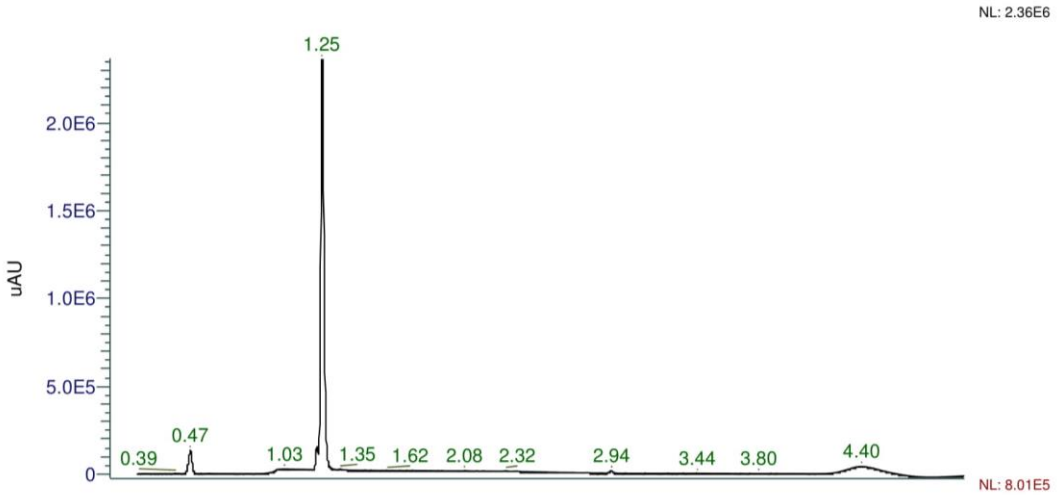

HRMS (NSI+):

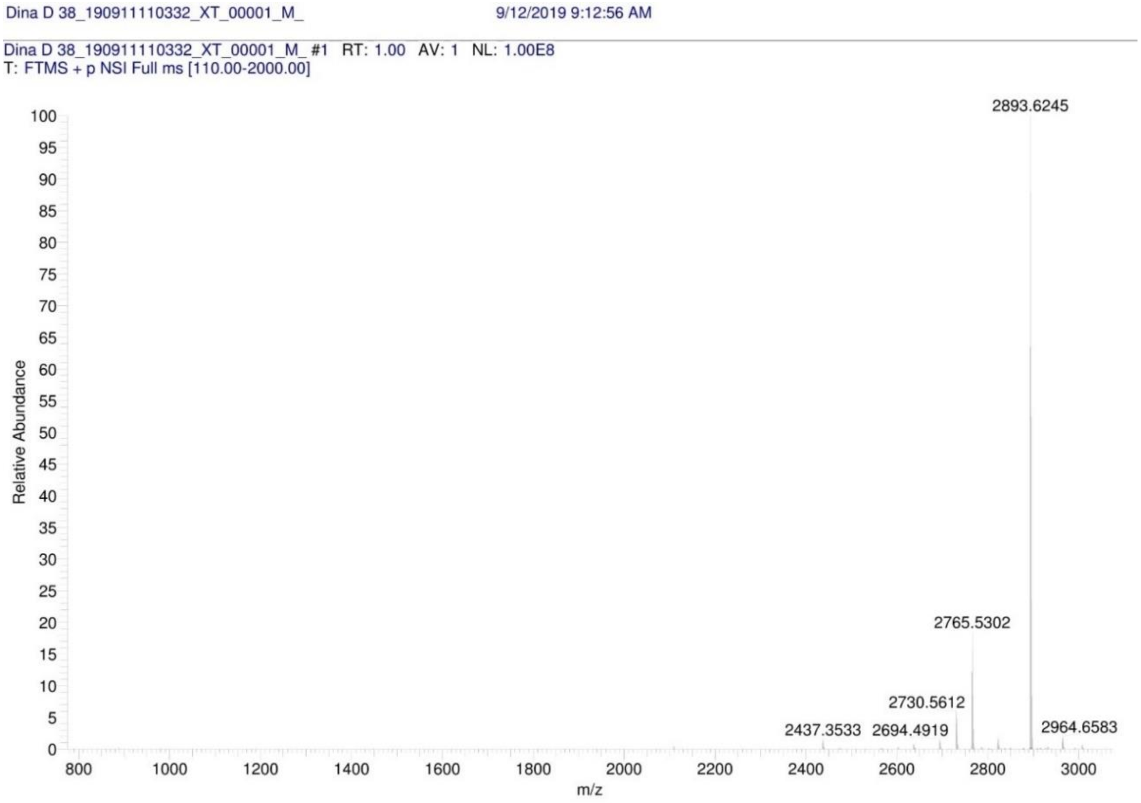
Dina D 38_190911110332_XT_00001_M_
9/12/2019 9:12:56 AM
Dina D 38_190911110332_XT_00001_M_ #1 RT: 1.00 AV: 1 NL: 1.00E8
T: FTMS + p NSI Full ms [110.00-2000.00]
2893.6245
2765.5302
2730.5612
2437.3533
2694.4919
2964.6583
Relative Abundance
m/z

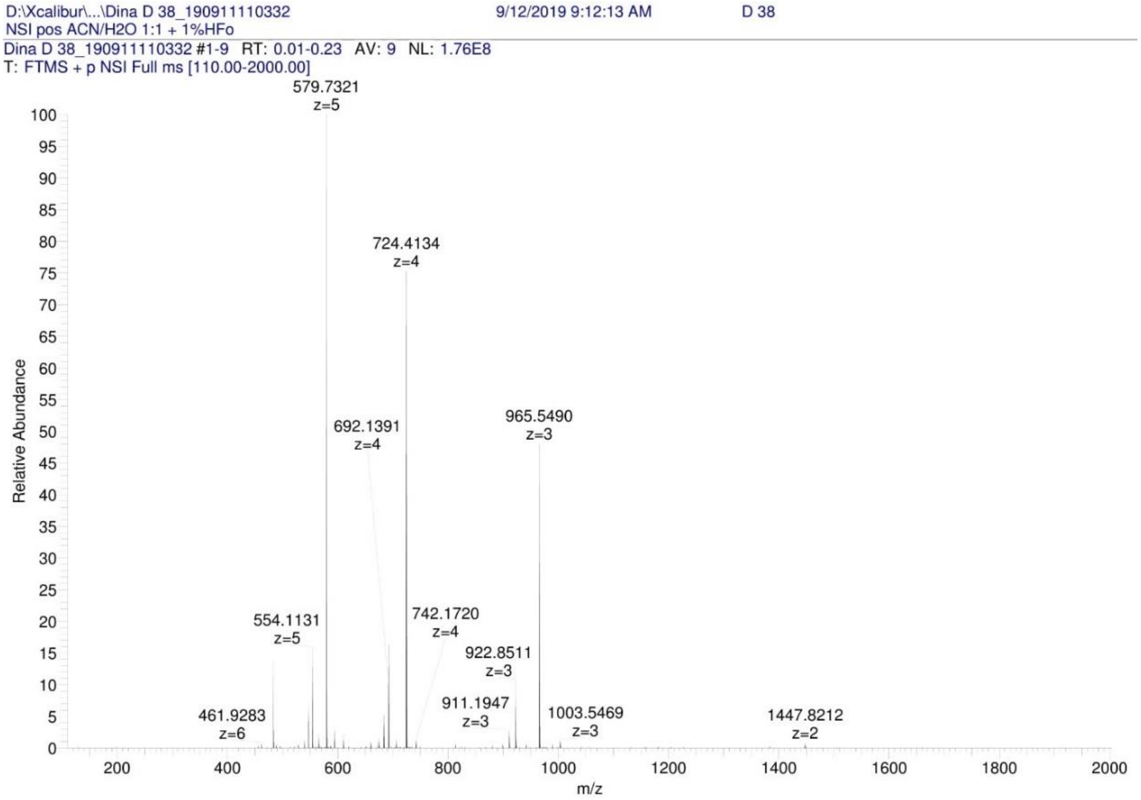
D:\Xcalibur\...\Dina D 38_190911110332
9/12/2019 9:12:13 AM
D 38
NSI pos ACN/H2O 1:1 + 1%HFo
Dina D 38_190911110332 #1-9 RT: 0.01-0.23 AV: 9 NL: 1.76E8
T: FTMS + p NSI Full ms [110.00-2000.00]
579.7321 z=5
724.4134 z=4
692.1391 z=4
965.5490 z=3
554.1131 z=5
742.1720 z=4
922.8511 z=3
911.1947 z=3
461.9283 z=6
1003.5469 z=3
1447.8212 z=2
Relative Abundance
m/z

Dina D 38_190911110332_XT_00001_M_ 9/12/2019 9:12:56 AM

Dina D 38_190911110332_XT_00001_M_ #1 RT: 1.00 AV: 1 NL: 1.00E8
T: FTMS + p NSI Full ms [110.00-2000.00]

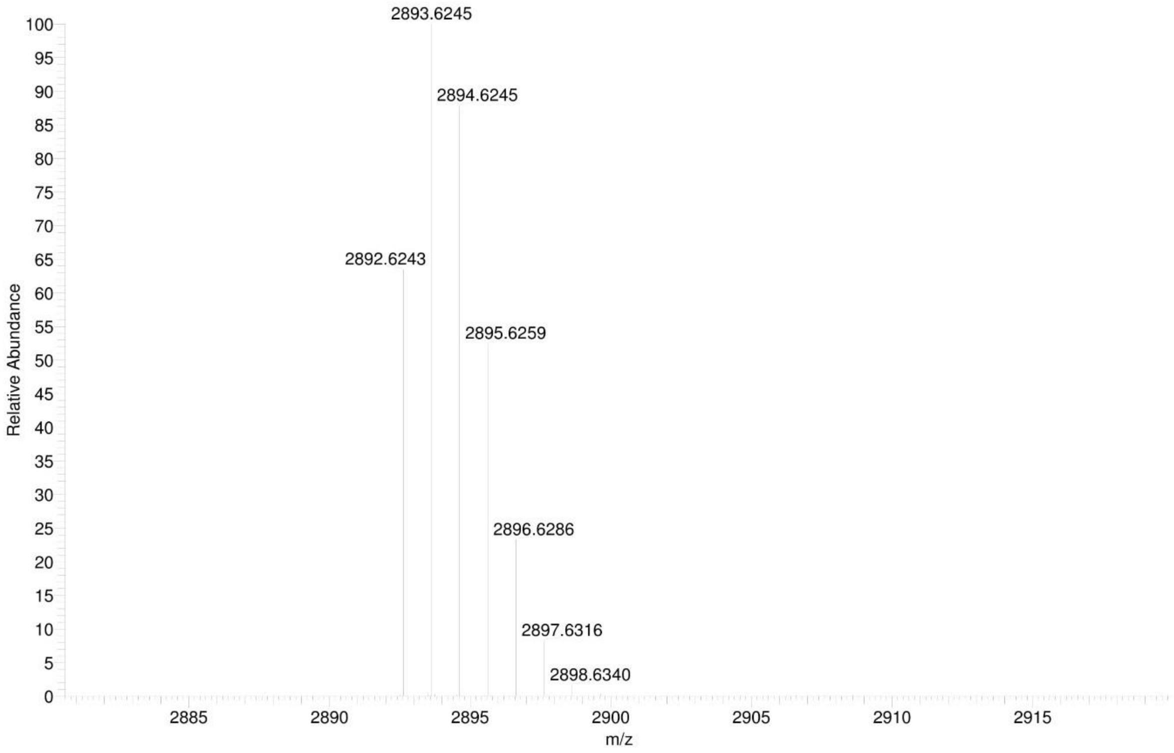

Ac**22** **((AcAKE)$_4$(*K*KAY)$_2$*K*YKY-NH$_2$)** was obtained after manual synthesis as foamy colourless solid after preparative RP-HPLC (35.6 mg, 7.2 µmol, 27.9%). Analytical RP-HPLC: t_R=1.32 min (100% A to 100% D in 5 min, λ= 214 nm). HRMS (ESI+): $C_{142}H_{225}N_{35}O_{40}$ calc./obs. 3060.6648/3060.6693 Da [M].

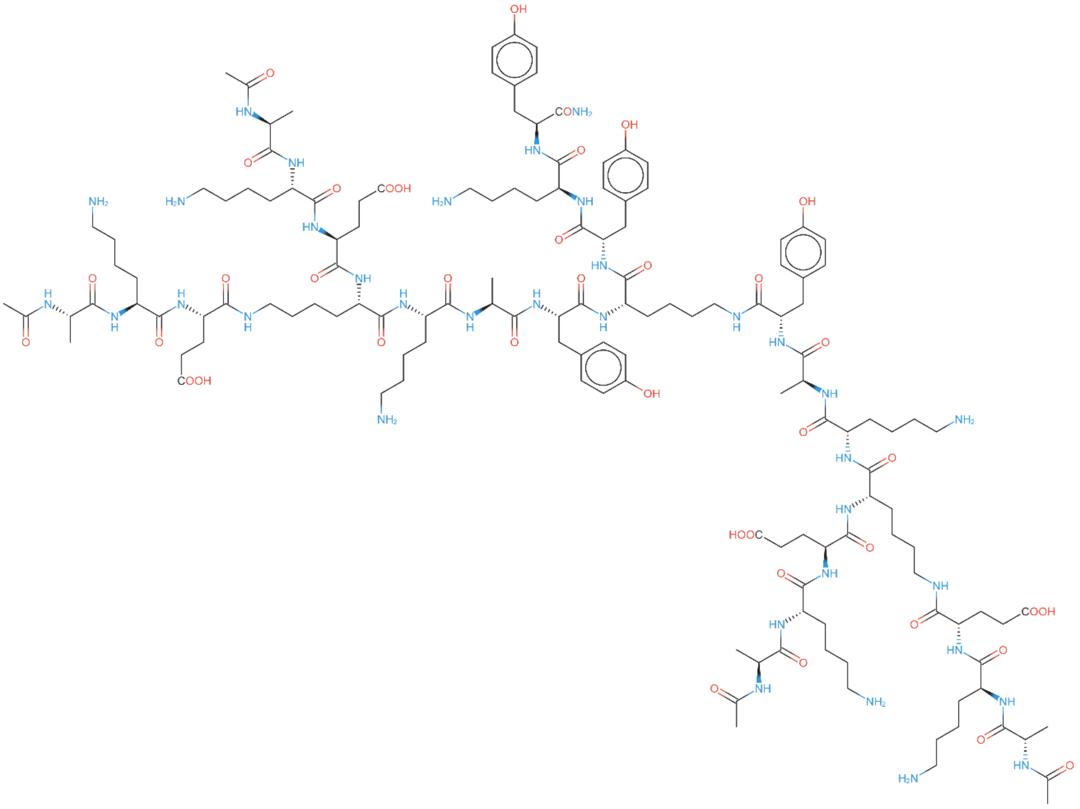

Analytical RP-HPLC:

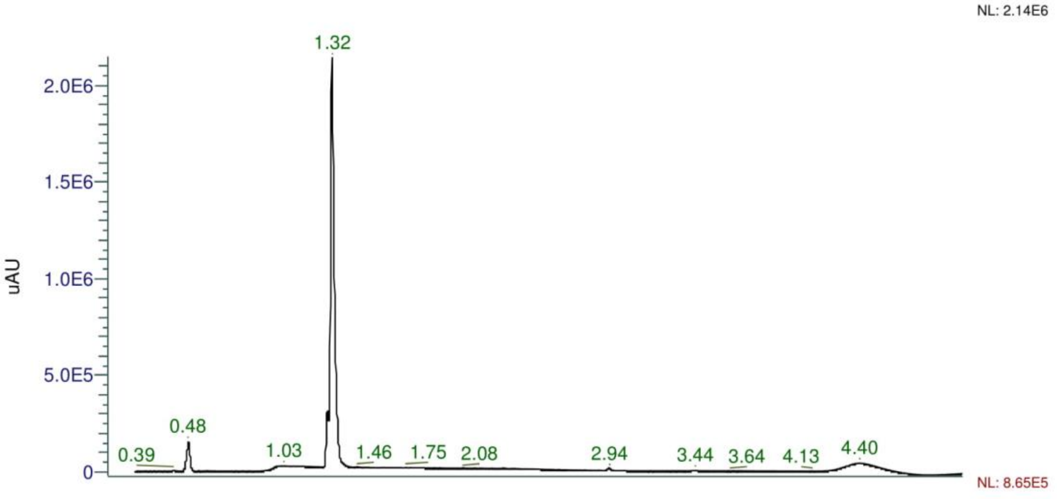

HRMS (NSI+):

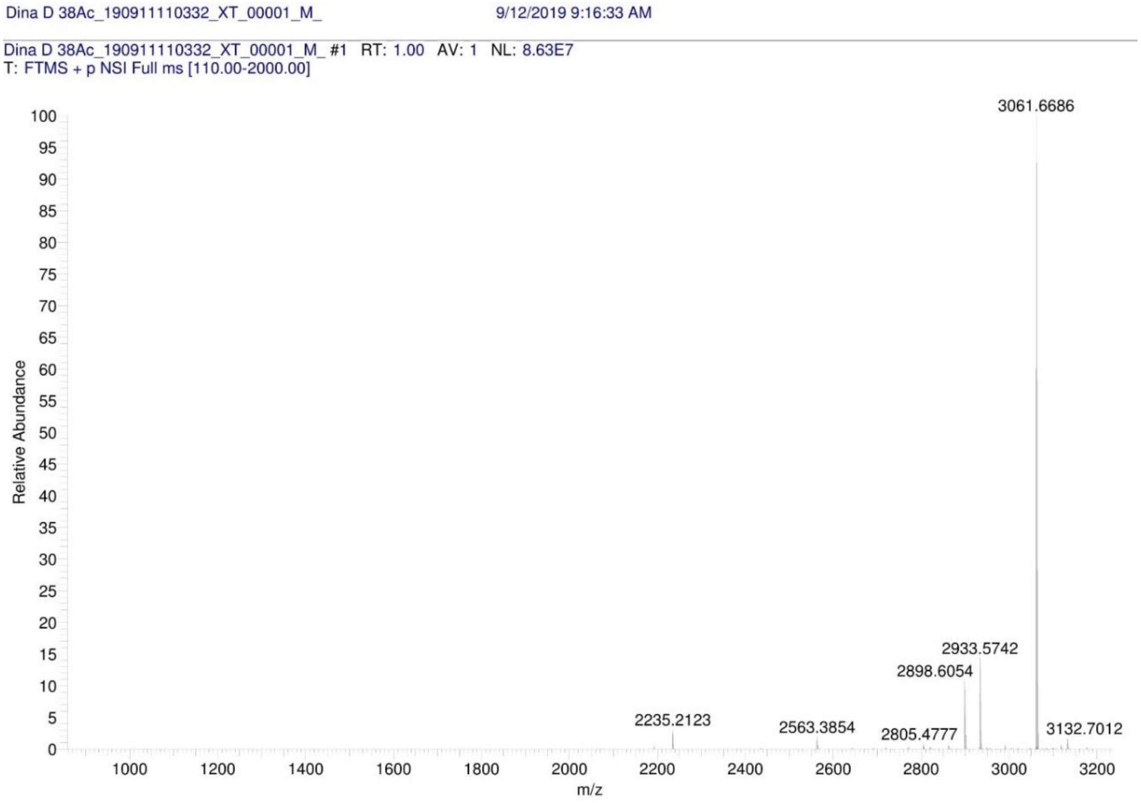
Dina D 38Ac_190911110332_XT_00001_M_
9/12/2019 9:16:33 AM
Dina D 38Ac_190911110332_XT_00001_M_ #1 RT: 1.00 AV: 1 NL: 8.63E7
T: FTMS + p NSI Full ms [110.00-2000.00]
3061.6686
2933.5742
2898.6054
2235.2123
2563.3854
2805.4777
3132.7012
Relative Abundance
m/z

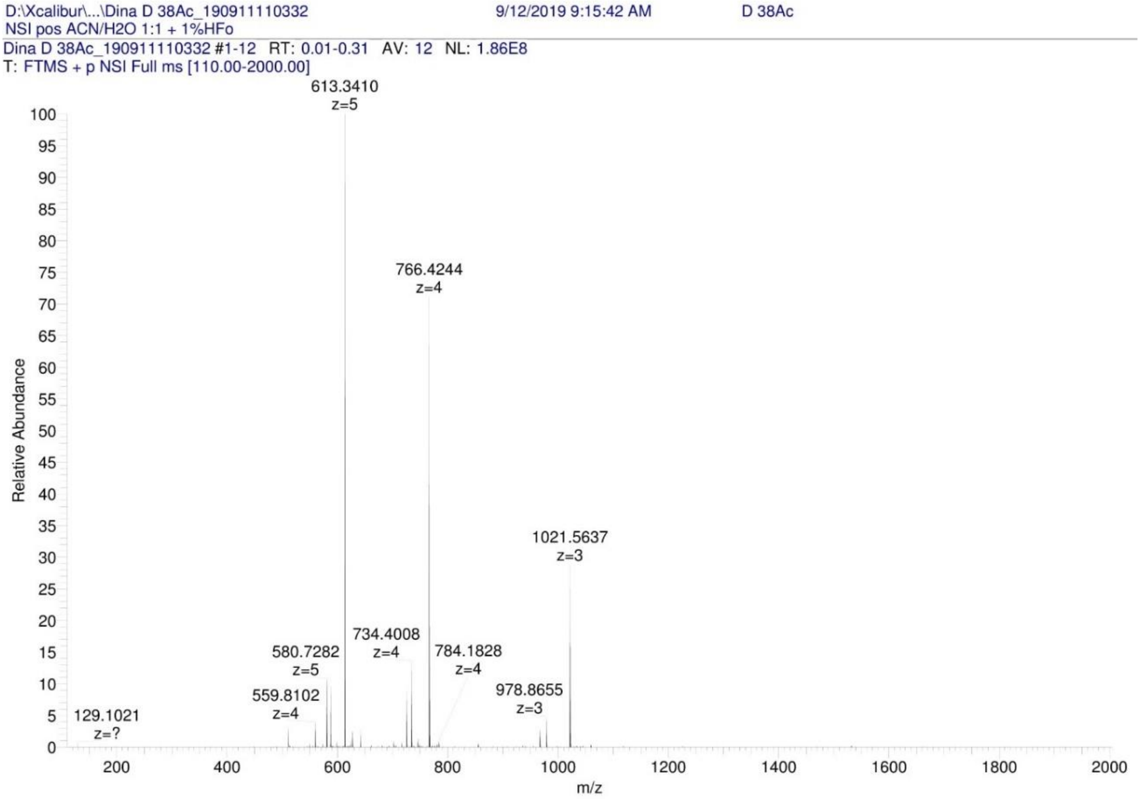
D:\Xcalibur\...\Dina D 38Ac_190911110332
9/12/2019 9:15:42 AM
D 38Ac
NSI pos ACN/H2O 1:1 + 1%HFo
Dina D 38Ac_190911110332 #1-12 RT: 0.01-0.31 AV: 12 NL: 1.86E8
T: FTMS + p NSI Full ms [110.00-2000.00]
613.3410 z=5
766.4244 z=4
1021.5637 z=3
734.4008 z=4
580.7282 z=5
784.1828 z=4
978.8655 z=3
559.8102 z=4
129.1021 z=?
Relative Abundance
m/z

Dina D 38Ac_190911110332_XT_00001_M_ 9/12/2019 9:16:33 AM

Dina D 38Ac_190911110332_XT_00001_M_ #1 RT: 1.00 AV: 1 NL: 8.63E7
T: FTMS + p NSI Full ms [110.00-2000.00]

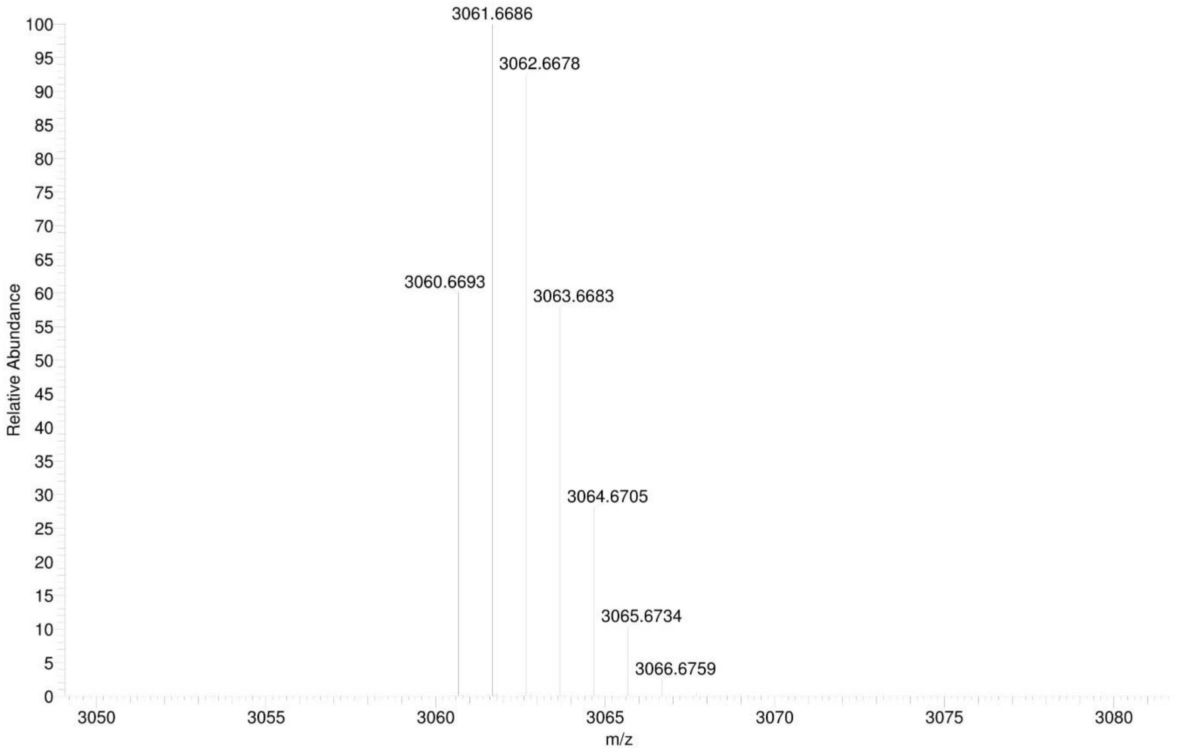

ClAc**22 ((ClAcAKE)$_4$(*K*KAY)$_2$*K*YKY-NH$_2$)** was obtained after manual synthesis as foamy colourless solid after preparative RP-HPLC (8.9 mg, 1.7 µmol, 6.7%). Analytical RP-HPLC: t_R=1.37 min (100% A to 100% D in 5 min, λ= 214 nm). HRMS (ESI+): $C_{142}H_{221}Cl_4N_{35}O_{40}$ calc./obs. 3196.5059/3196.5148 Da [M].

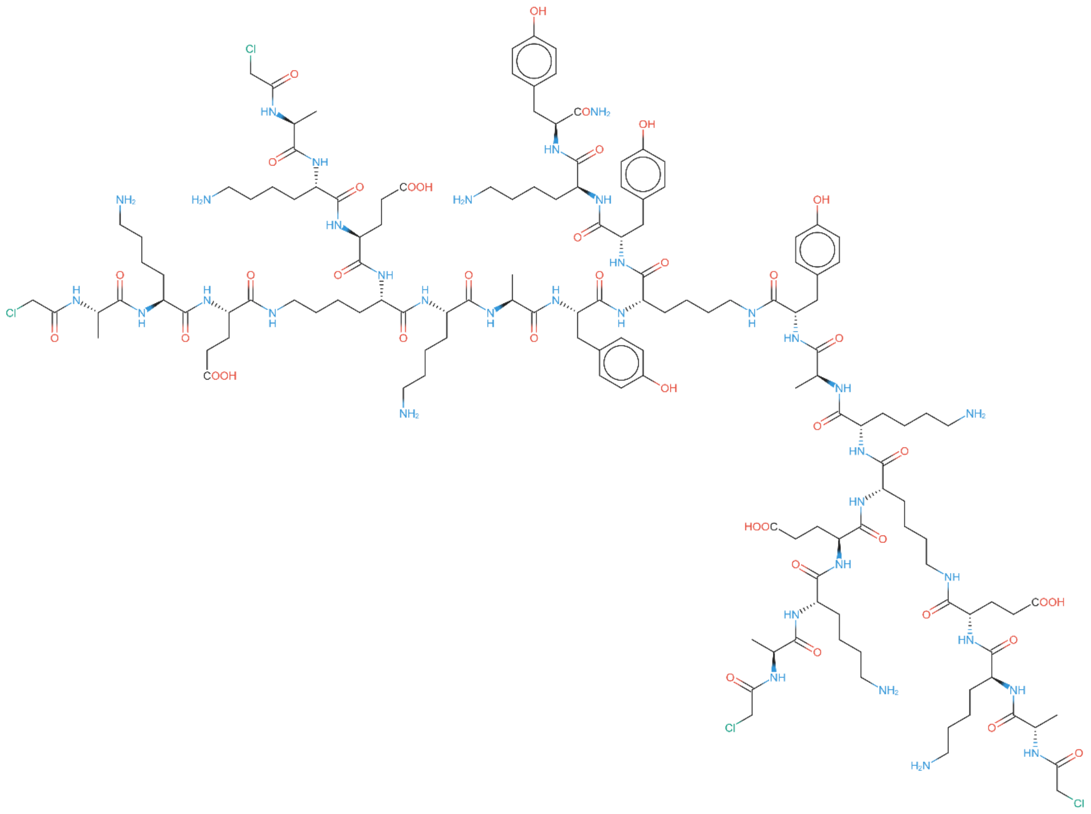

Analytical RP-HPLC:

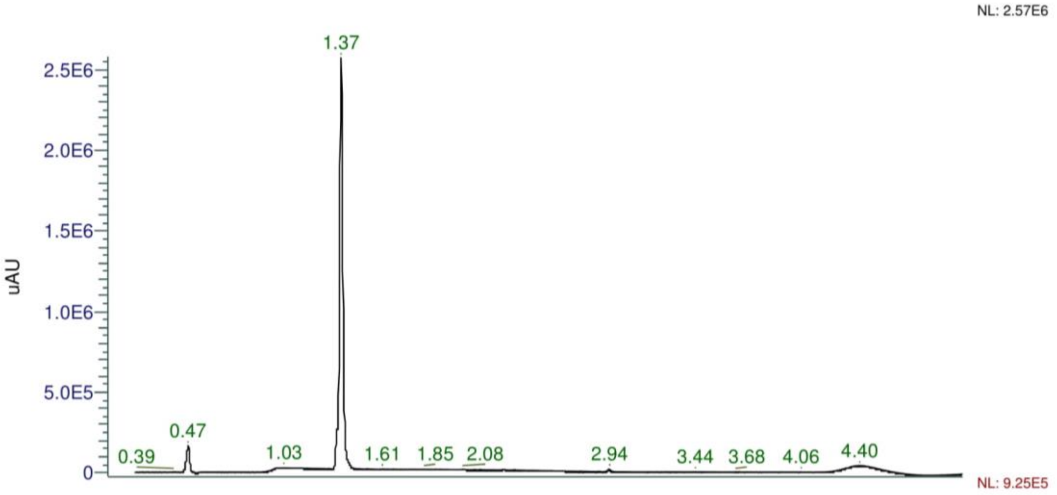

HRMS (NSI+):

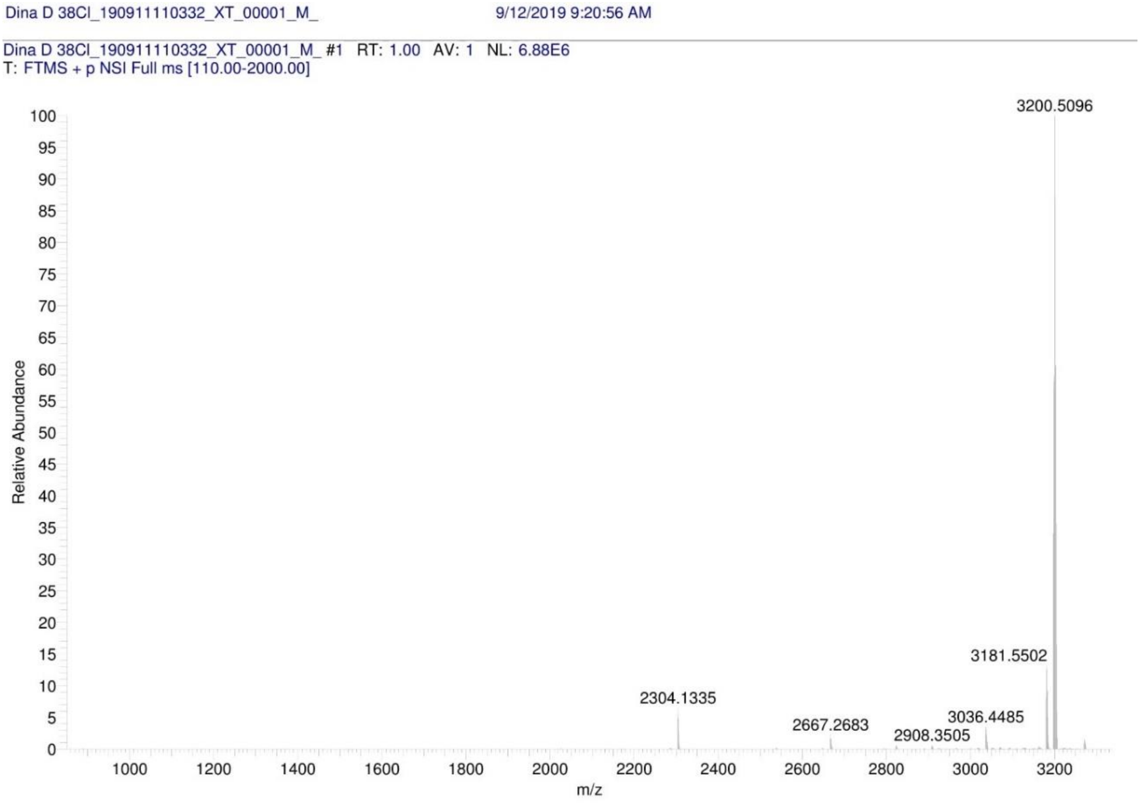
Dina D 38Cl_190911110332_XT_00001_M_ 9/12/2019 9:20:56 AM
Dina D 38Cl_190911110332_XT_00001_M_ #1 RT: 1.00 AV: 1 NL: 6.88E6
T: FTMS + p NSI Full ms [110.00-2000.00]
3200.5096
3181.5502
2304.1335
2667.2683
2908.3505
3036.4485
Relative Abundance
m/z

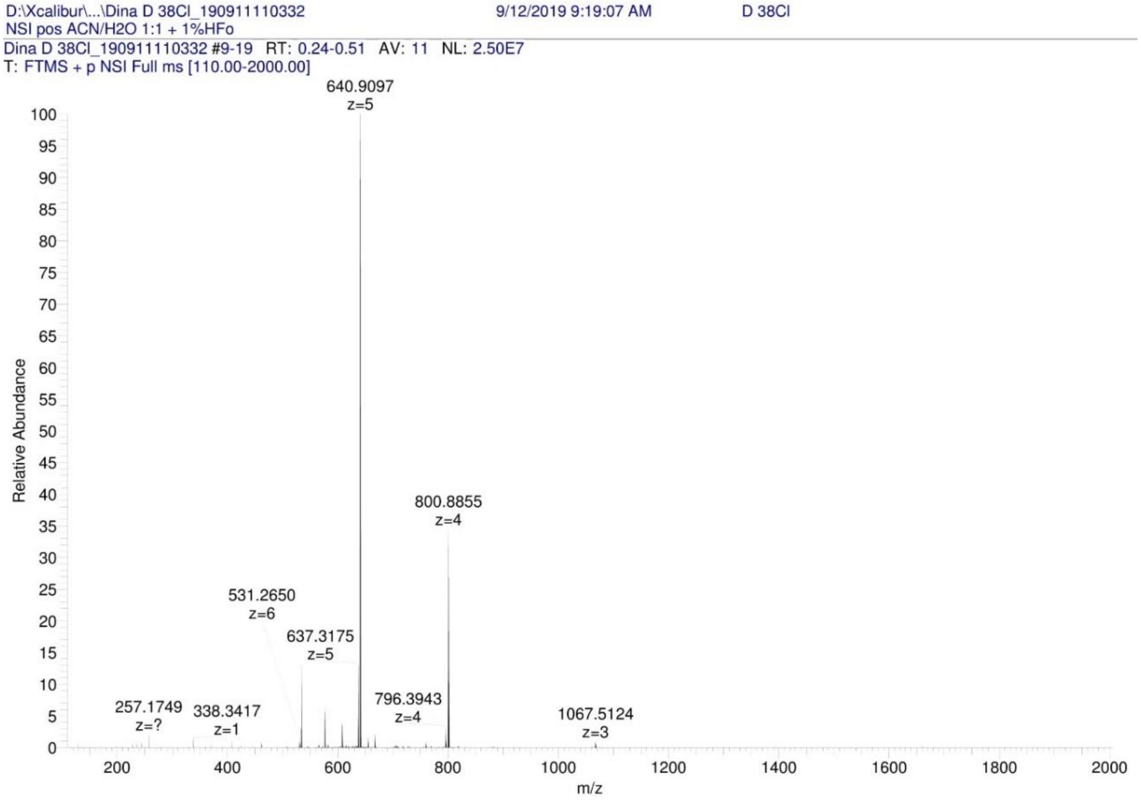
D:\Xcalibur\...\Dina D 38Cl_190911110332 9/12/2019 9:19:07 AM D 38Cl
NSI pos ACN/H2O 1:1 + 1%HFo
Dina D 38Cl_190911110332 #9-19 RT: 0.24-0.51 AV: 11 NL: 2.50E7
T: FTMS + p NSI Full ms [110.00-2000.00]
640.9097 z=5
800.8855 z=4
531.2650 z=6
637.3175 z=5
257.1749 z=?
338.3417 z=1
796.3943 z=4
1067.5124 z=3
Relative Abundance
m/z

Dina D 38Cl_190911110332_XT_00001_M_ 9/12/2019 9:20:56 AM

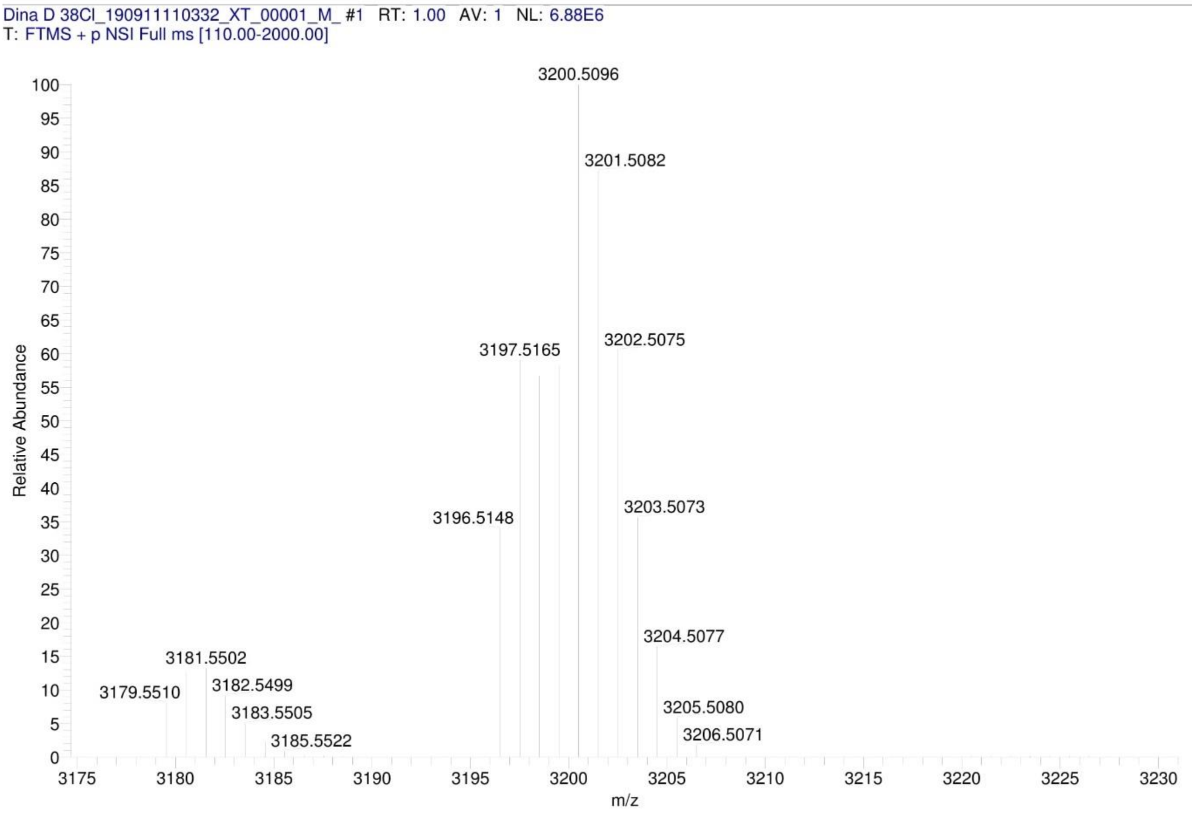

23 ((KYA)$_4$(*K*AE)$_2$*K*AEYKCA-NH$_2$) was obtained after manual synthesis as foamy colourless solid after preparative RP-HPLC (127.7 mg, 14.6 µmol, 29.5%). Analytical RP-HPLC: t_R=1.27 min (100% A to 100% D in 5 min, λ= 214 nm). HRMS (ESI+): $C_{135}H_{212}N_{36}O_{34}S$ calc./obs. 2913.5687/2915.5439 Da [M].

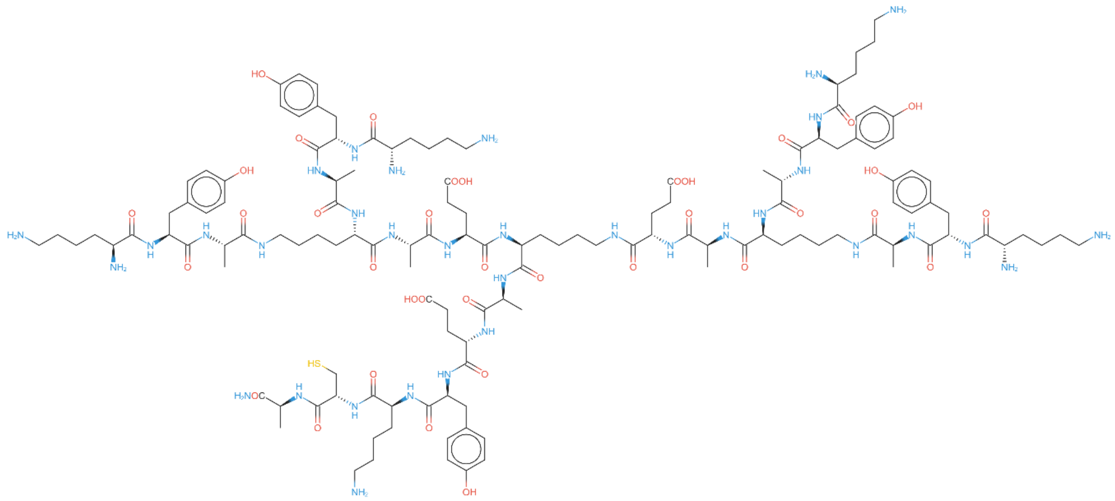

Analytical RP-HPLC:

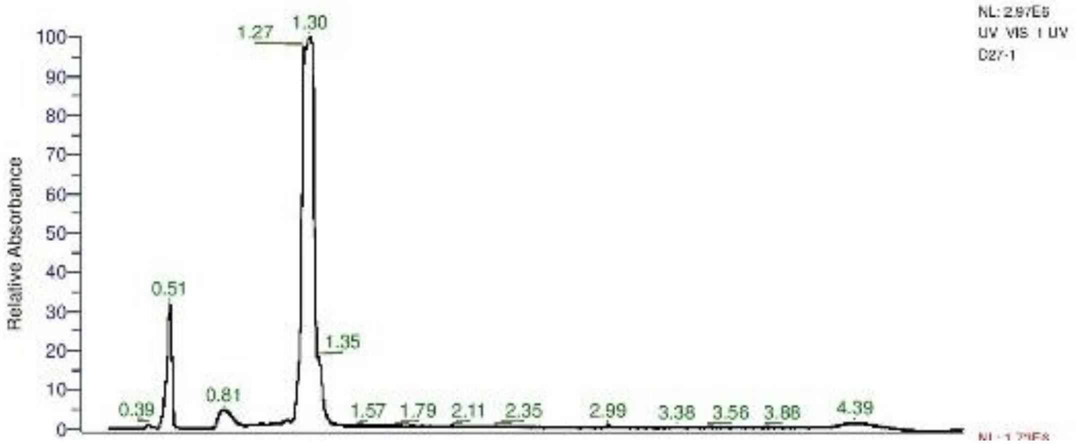

HRMS (NSI+):

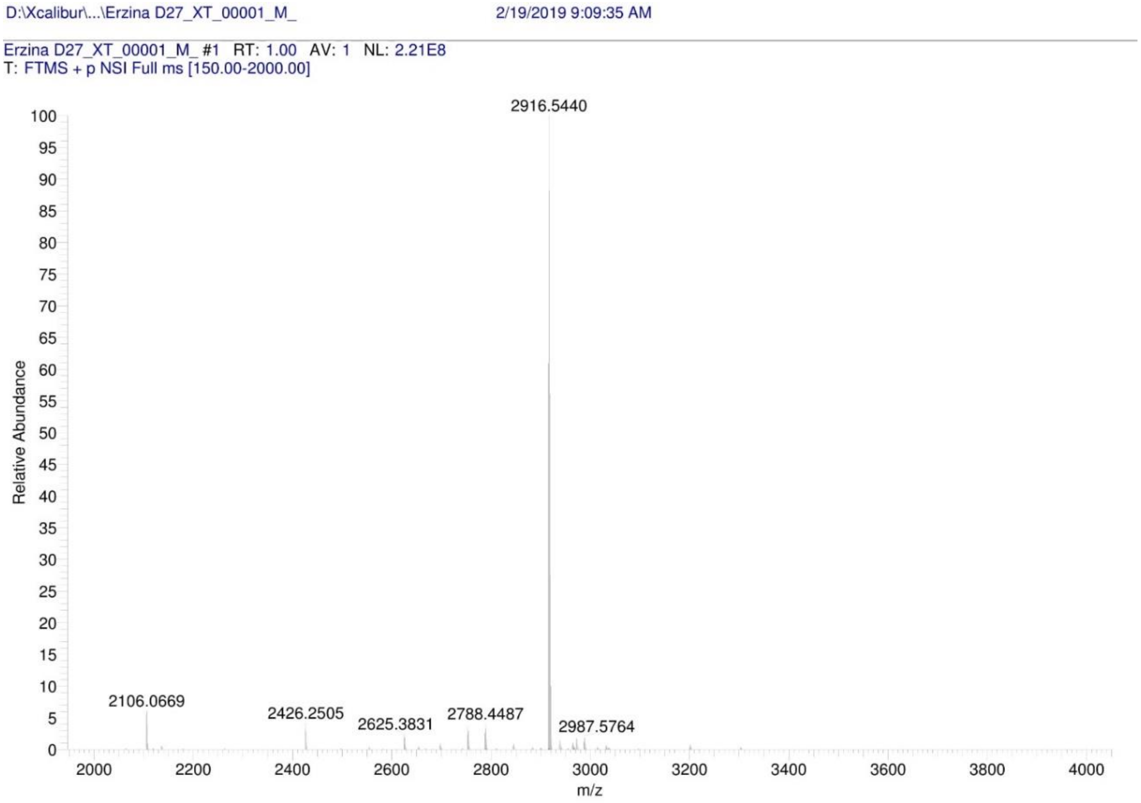
D:\Xcalibur\...\Erzina D27_XT_00001_M_
2/19/2019 9:09:35 AM
Erzina D27_XT_00001_M_ #1 RT: 1.00 AV: 1 NL: 2.21E8
T: FTMS + p NSI Full ms [150.00-2000.00]
2916.5440
2106.0669
2426.2505
2625.3831
2788.4487
2987.5764
Relative Abundance
m/z

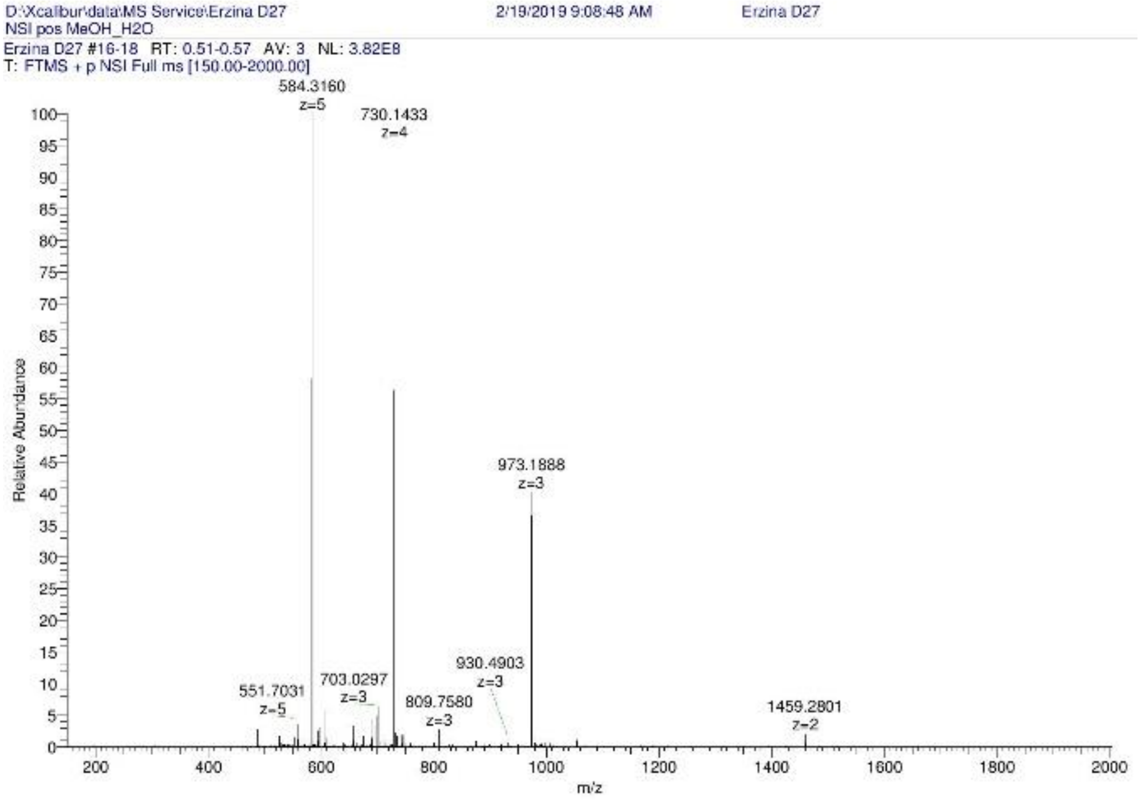
D:\Xcalibur\data\MS Service\Erzina D27
NSI pos MeOH_H2O
2/19/2019 9:08:48 AM
Erzina D27
Erzina D27 #16-18 RT: 0.51-0.57 AV: 3 NL: 3.82E8
T: FTMS + p NSI Full ms [150.00-2000.00]
584.3160 z=5
730.1433 z=4
973.1888 z=3
930.4903 z=3
703.0297 z=3
551.7031 z=5
809.7580 z=3
1459.2801 z=2
Relative Abundance
m/z

Dina D 27_190911110332_XT_00001_M_ 9/12/2019 8:07:16 AM

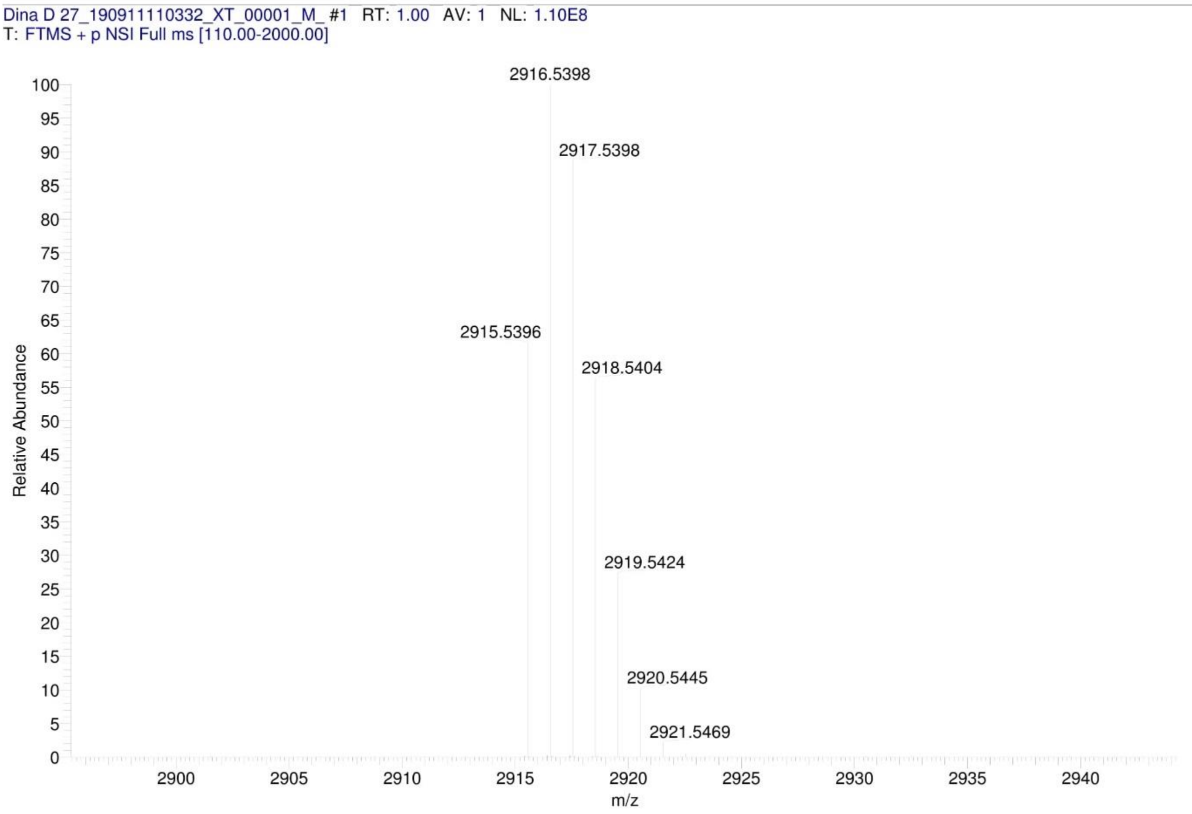

24 ((KAA)$_4$(*K*EY)$_2$*K*AKEYEYA-NH$_2$) was obtained after manual synthesis as foamy colourless solid after preparative RP-HPLC (180 mg, 10.5 µmol, 41.1%). Analytical RP-HPLC: t_R=1.25 min (100% A to 100% D in 5 min, λ= 214 nm). HRMS (ESI+): $C_{134}H_{213}N_{35}O_{38}$ calc./obs. 2920.5811/2920.5847 Da [M].

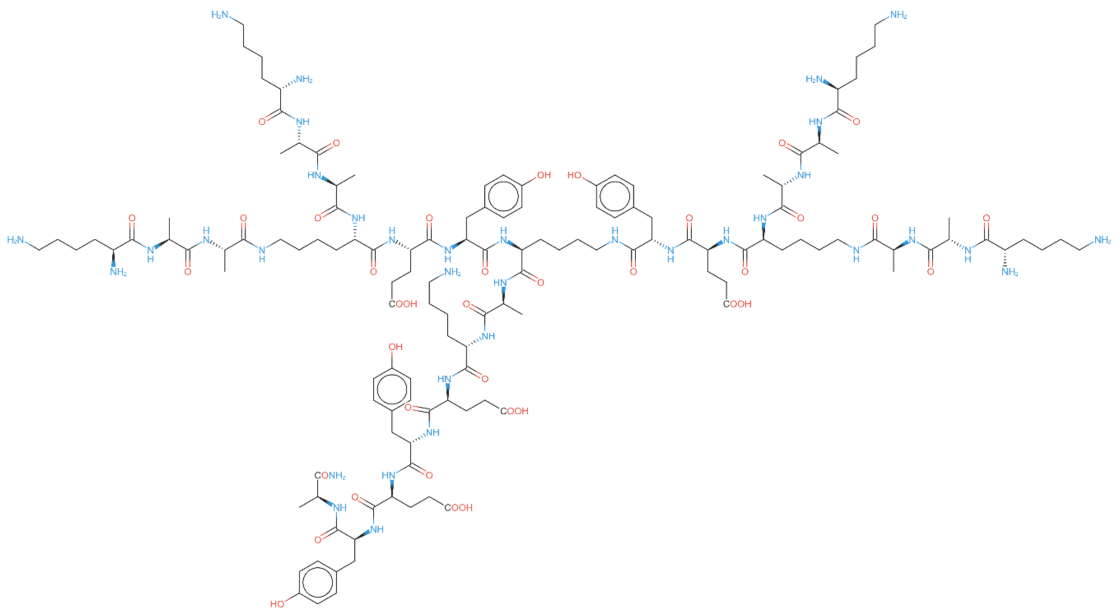

Analytical RP-HPLC:

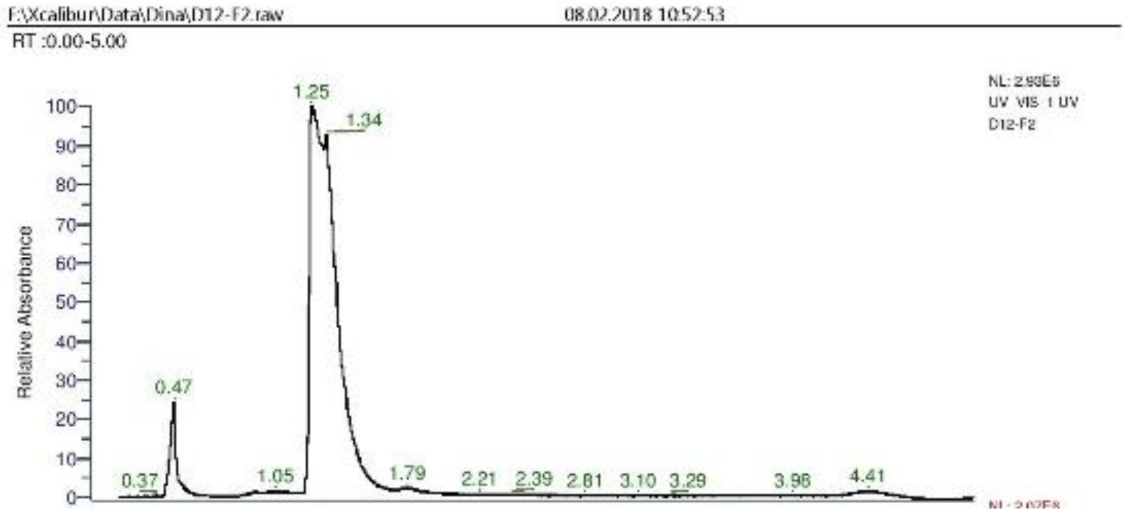

HRMS (NSI+):

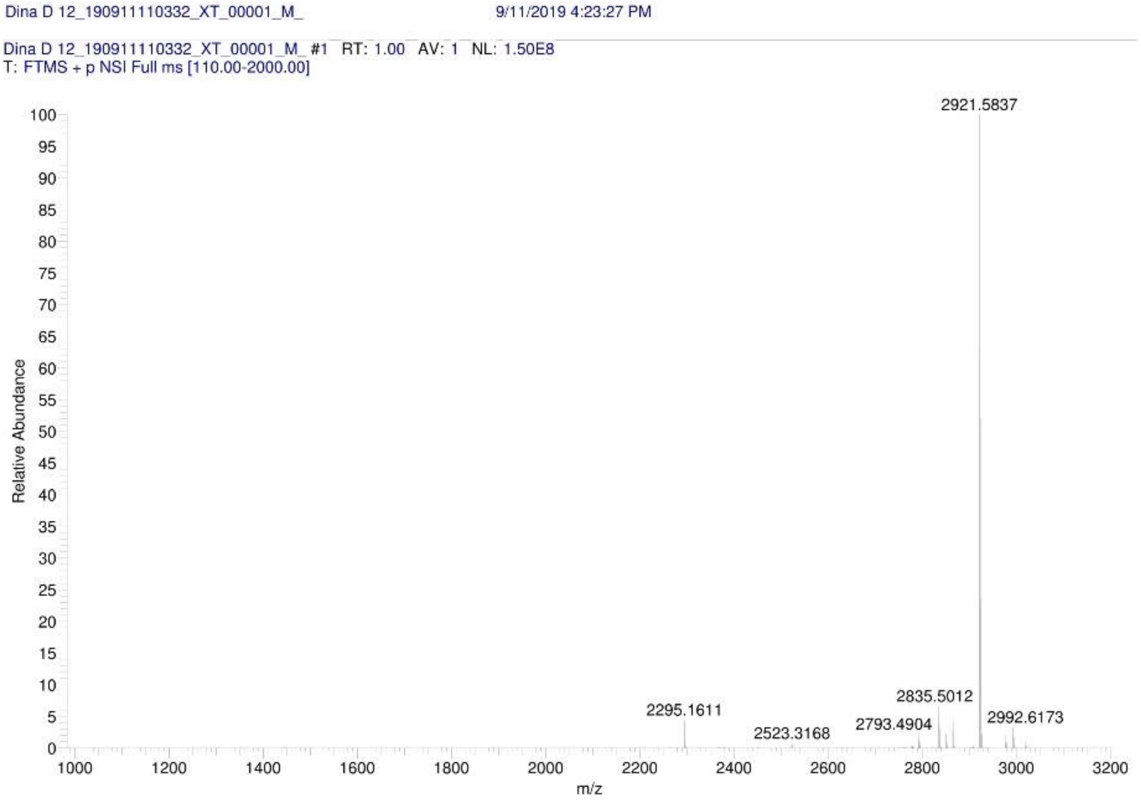

Dina D 12_190911110332_XT_00001_M_
9/11/2019 4:23:27 PM
Dina D 12_190911110332_XT_00001_M_ #1 RT: 1.00 AV: 1 NL: 1.50E8
T: FTMS + p NSI Full ms [110.00-2000.00]
2921.5837
2835.5012
2295.1611
2793.4904
2992.6173
2523.3168
Relative Abundance
m/z

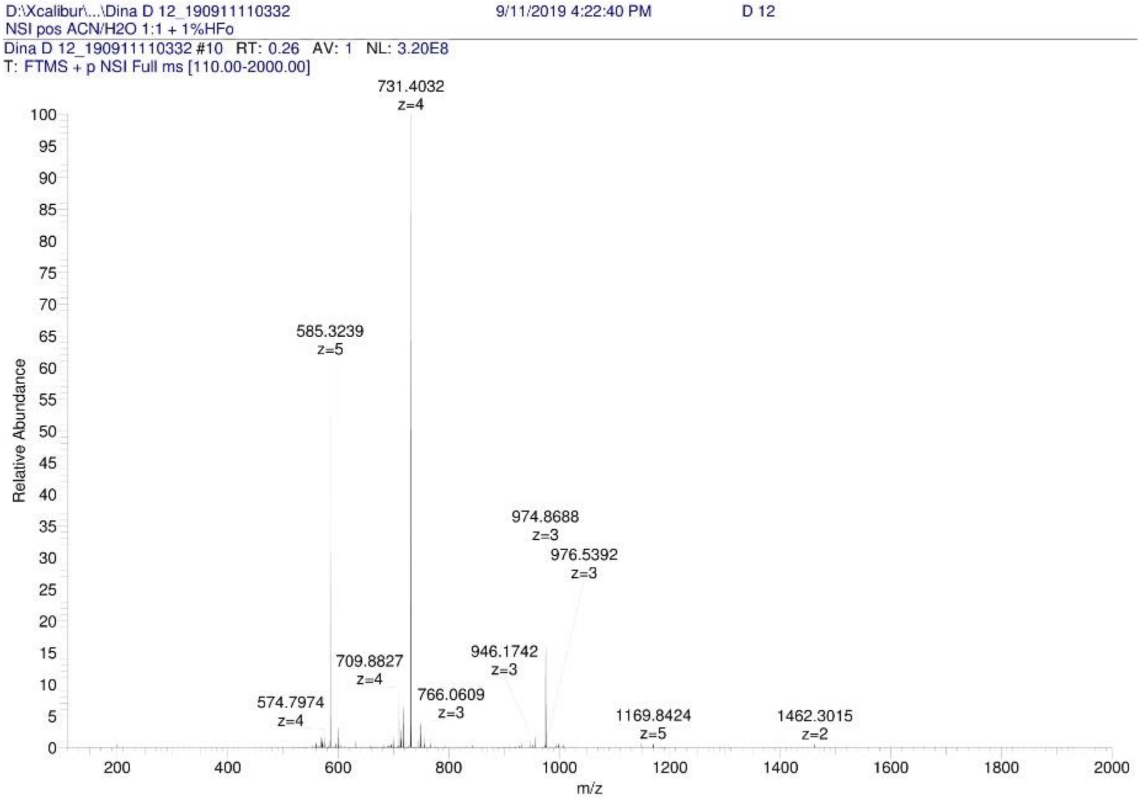

D:\Xcalibur\...\Dina D 12_190911110332
9/11/2019 4:22:40 PM
D 12
NSI pos ACN/H2O 1:1 + 1%HFo
Dina D 12_190911110332 #10 RT: 0.26 AV: 1 NL: 3.20E8
T: FTMS + p NSI Full ms [110.00-2000.00]
731.4032
z=4
585.3239
z=5
974.8688
z=3
976.5392
z=3
946.1742
z=3
709.8827
z=4
766.0609
z=3
574.7974
z=4
1169.8424
z=5
1462.3015
z=2
Relative Abundance
m/z

Dina D 12_190911110332_XT_00001_M_ 9/11/2019 4:23:27 PM

Dina D 12_190911110332_XT_00001_M_ #1 RT: 1.00 AV: 1 NL: 1.50E8
T: FTMS + p NSI Full ms [110.00-2000.00]

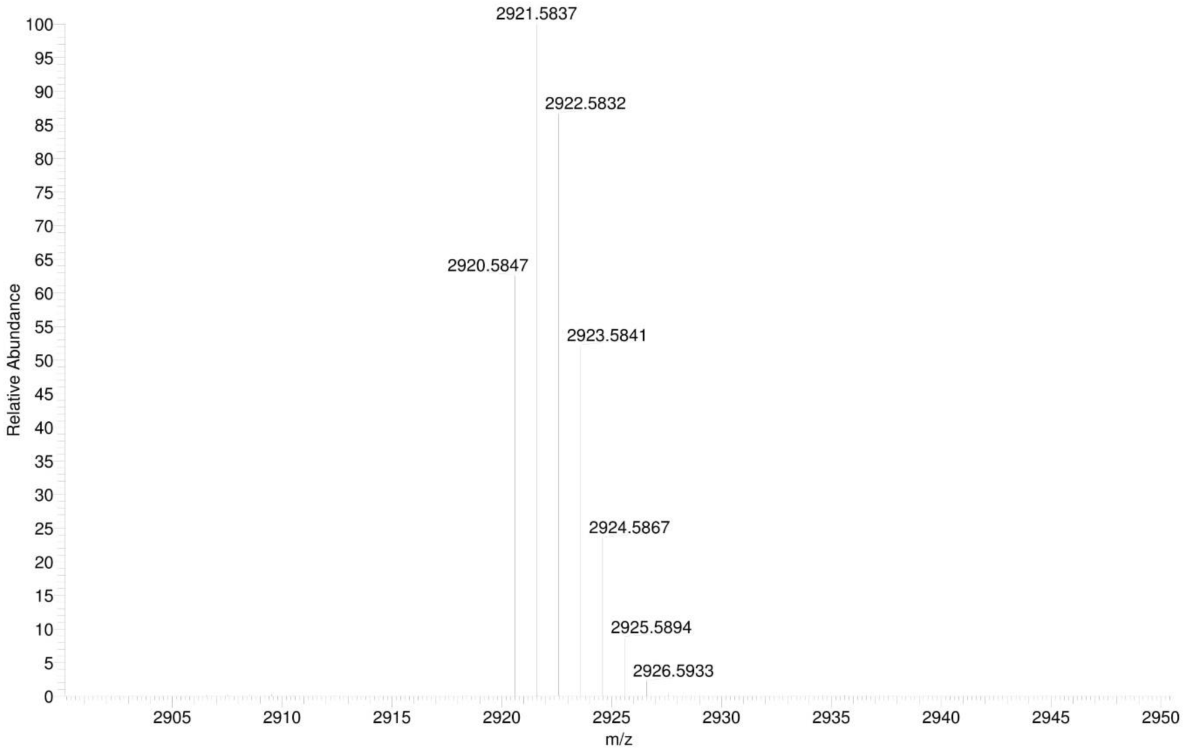

ClAc**25 ((ClAcK)$_4$(*K*EAKAKEY)$_2$*K*AKEYEY-OH)** was obtained from the CEM Liberty Blue synthetiser as foamy colourless solid after preparative RP-HPLC (159.9 mg, 4.9 µmol, 22.8%). Analytical RP-HPLC: t_R=1.34 min (100% A to 100% D in 5 min, λ= 214 nm). HRMS (ESI+): $C_{161}H_{253}Cl_4N_{39}O_{48}$ calc./obs. 3640.7309/3640.7259 Da [M].

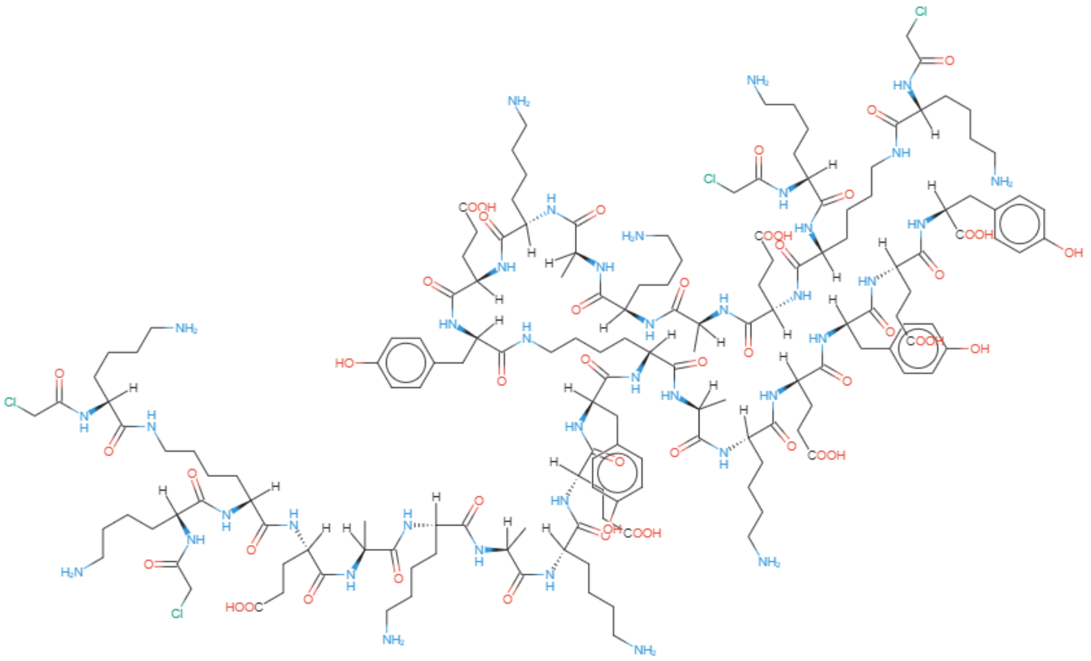

Analytical RP-HPLC:

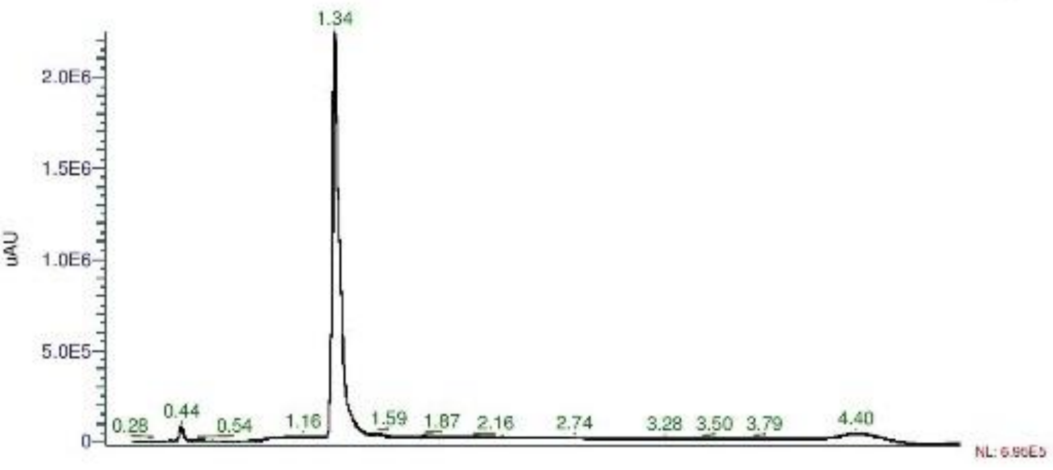

HRMS (NSI+):

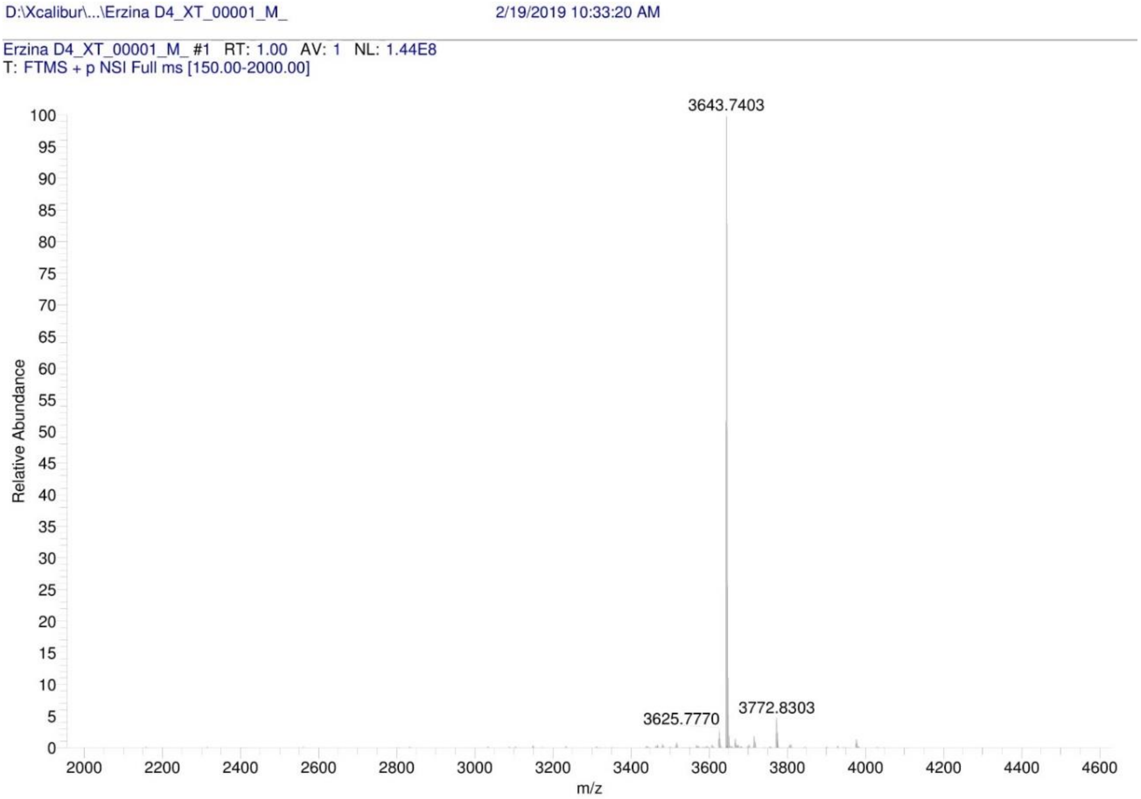
D:\Xcalibur\...\Erzina D4_XT_00001_M_
2/19/2019 10:33:20 AM
Erzina D4_XT_00001_M_ #1 RT: 1.00 AV: 1 NL: 1.44E8
T: FTMS + p NSI Full ms [150.00-2000.00]
3643.7403
3625.7770
3772.8303
Relative Abundance
m/z

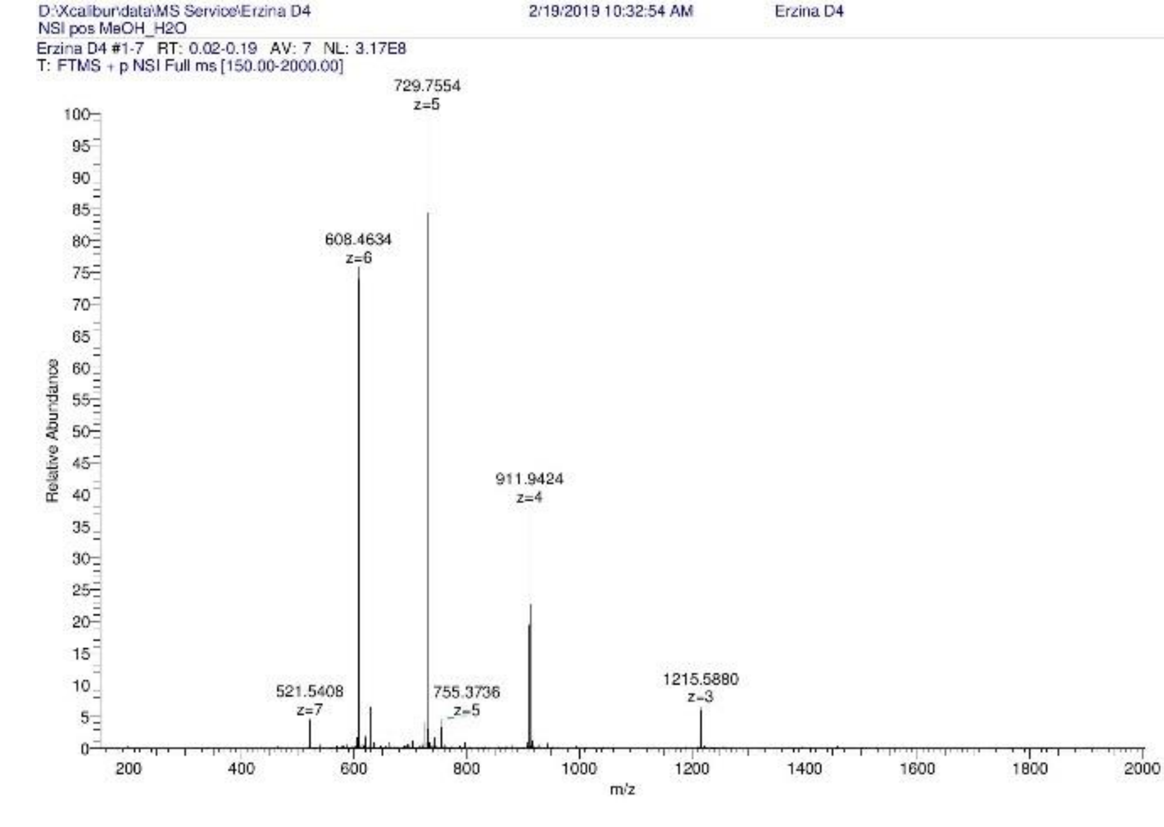
D:\Xcalibur\data\MS Service\Erzina D4
2/19/2019 10:32:54 AM
Erzina D4
NSI pos MeOH_H2O
Erzina D4 #1-7 RT: 0.02-0.19 AV: 7 NL: 3.17E8
T: FTMS + p NSI Full ms [150.00-2000.00]
729.7554
z=5
608.4634
z=6
911.9424
z=4
521.5408
z=7
755.3736
z=5
1215.5880
z=3
Relative Abundance
m/z

D:\Xcalibur\...\Erzina D4_XT_00001_M_ 2/19/2019 10:33:20 AM

Erzina D4_XT_00001_M_ #1 RT: 1.00 AV: 1 NL: 1.44E8
T: FTMS + p NSI Full ms [150.00-2000.00]

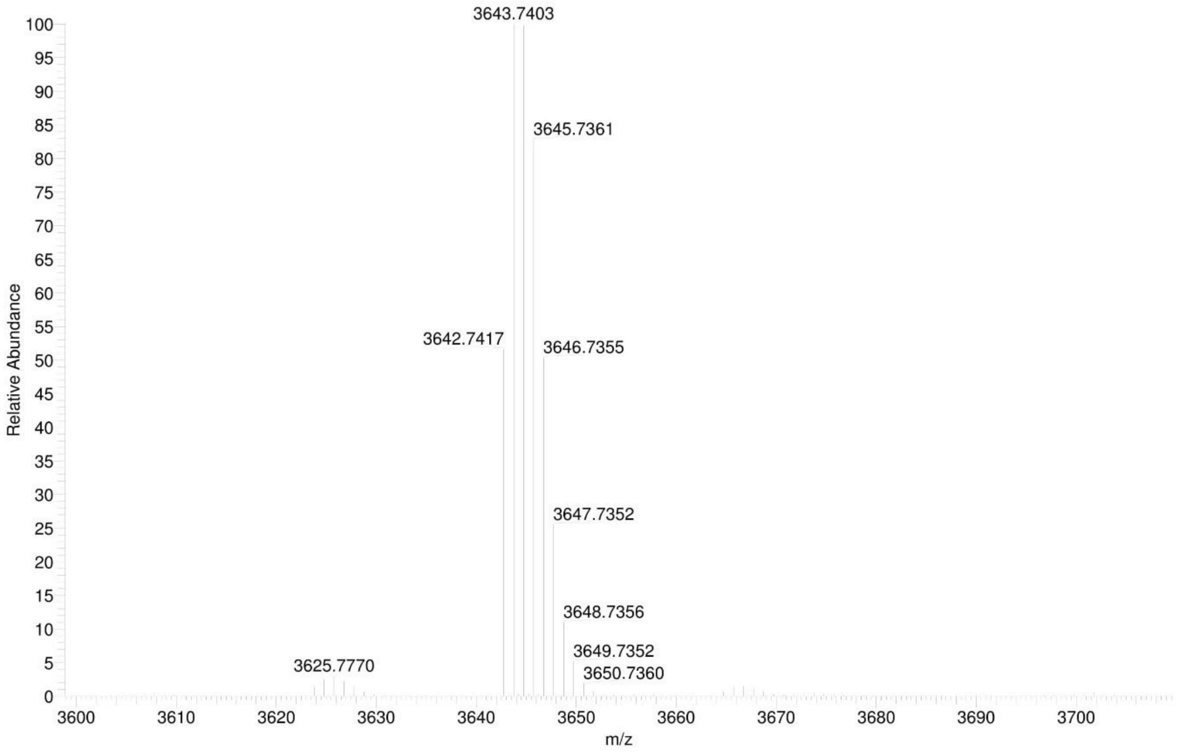

26 ((AK)$_4$(*K*AEAKAKE)$_2$*K*KEYEYCA-NH$_2$) was obtained after manual synthesis as foamy colourless solid after preparative RP-HPLC (138 mg, 7.3 µmol, 39.1%). Analytical RP-HPLC: t_R=1.30 min (100% A to 100% D in 5 min, λ= 214 nm). HRMS (ESI+): $C_{174}H_{285}N_{47}O_{50}S$ calc./obs. 3865.0924/3865.1068 Da [M].

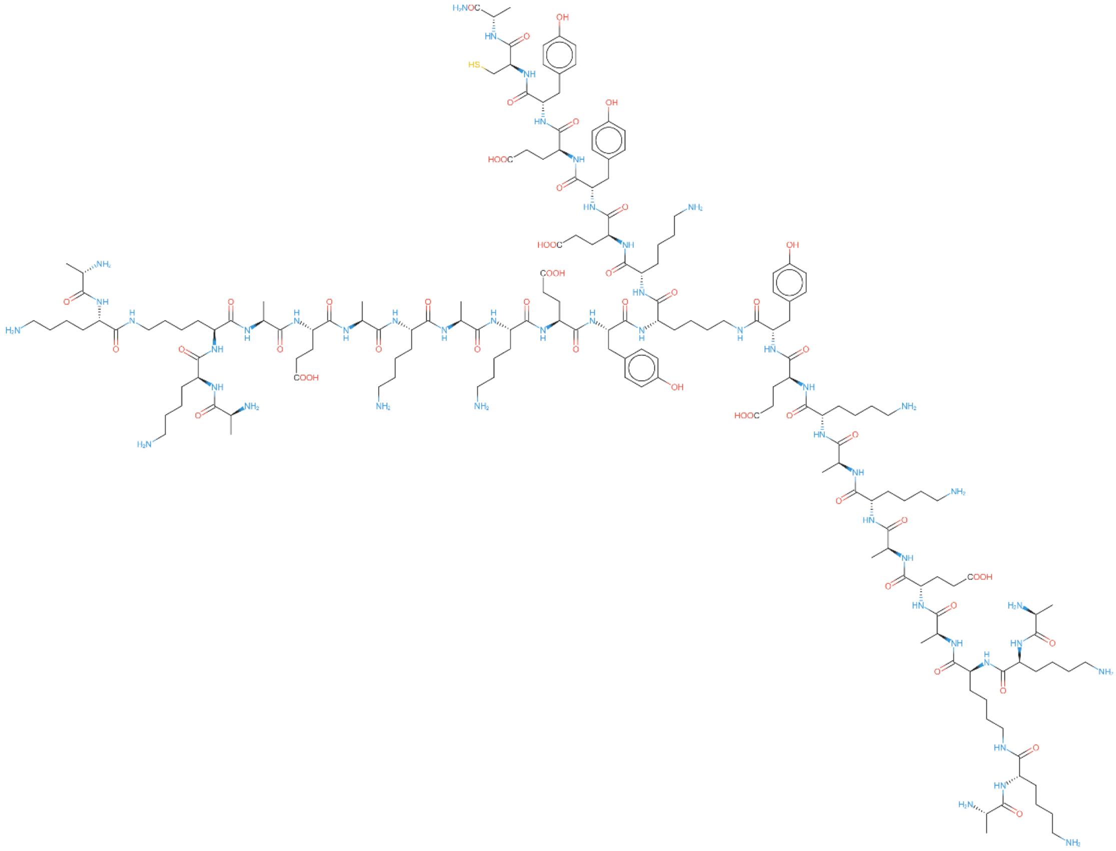

Analytical RP-HPLC:

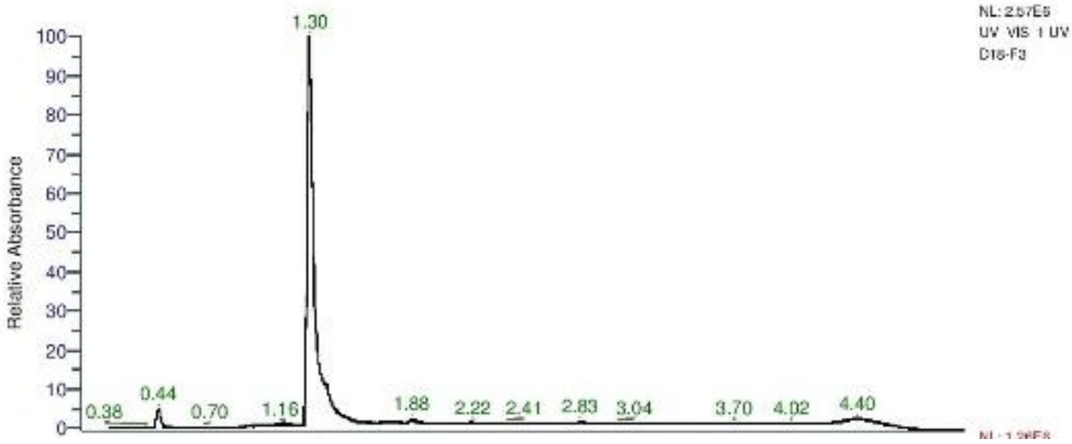

HRMS (NSI+):

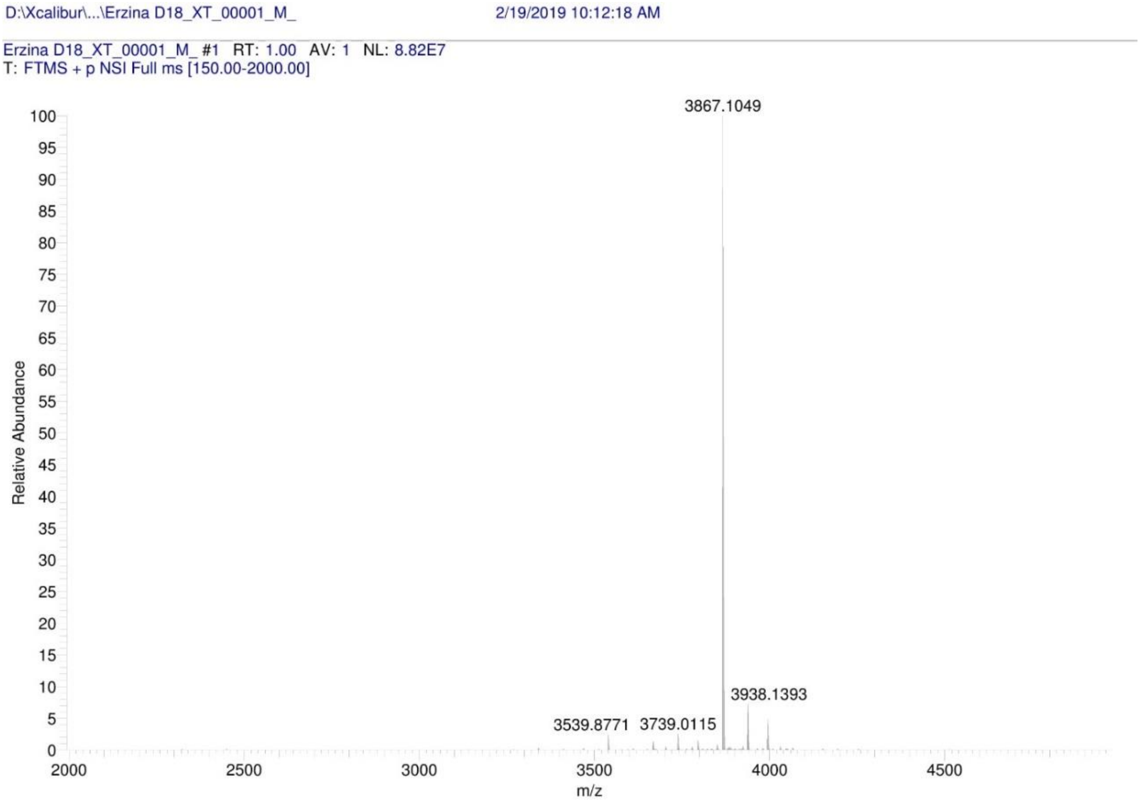

D:\Xcalibur\...\Erzina D18_XT_00001_M_
2/19/2019 10:12:18 AM
Erzina D18_XT_00001_M_ #1 RT: 1.00 AV: 1 NL: 8.82E7
T: FTMS + p NSI Full ms [150.00-2000.00]
3867.1049
3938.1393
3539.8771
3739.0115
Relative Abundance
m/z

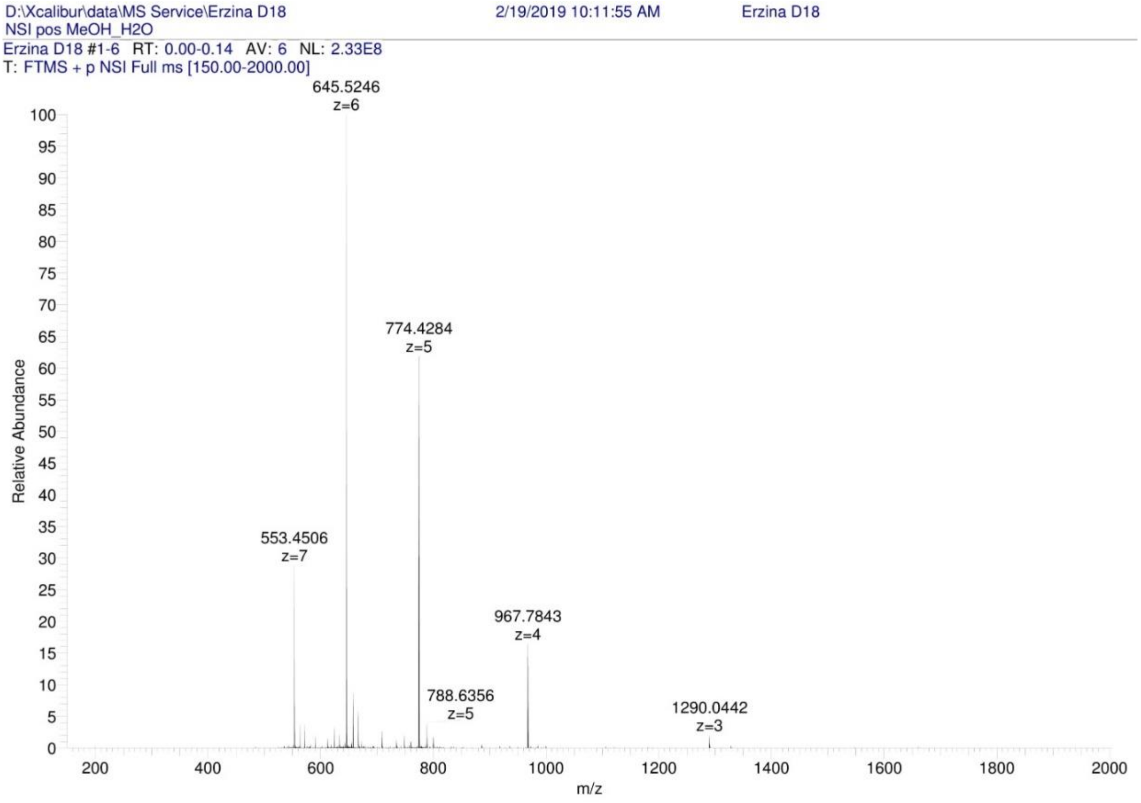

D:\Xcalibur\data\MS Service\Erzina D18
2/19/2019 10:11:55 AM
Erzina D18
NSI pos MeOH_H2O
Erzina D18 #1-6 RT: 0.00-0.14 AV: 6 NL: 2.33E8
T: FTMS + p NSI Full ms [150.00-2000.00]
645.5246
z=6
774.4284
z=5
553.4506
z=7
967.7843
z=4
788.6356
z=5
1290.0442
z=3
Relative Abundance
m/z

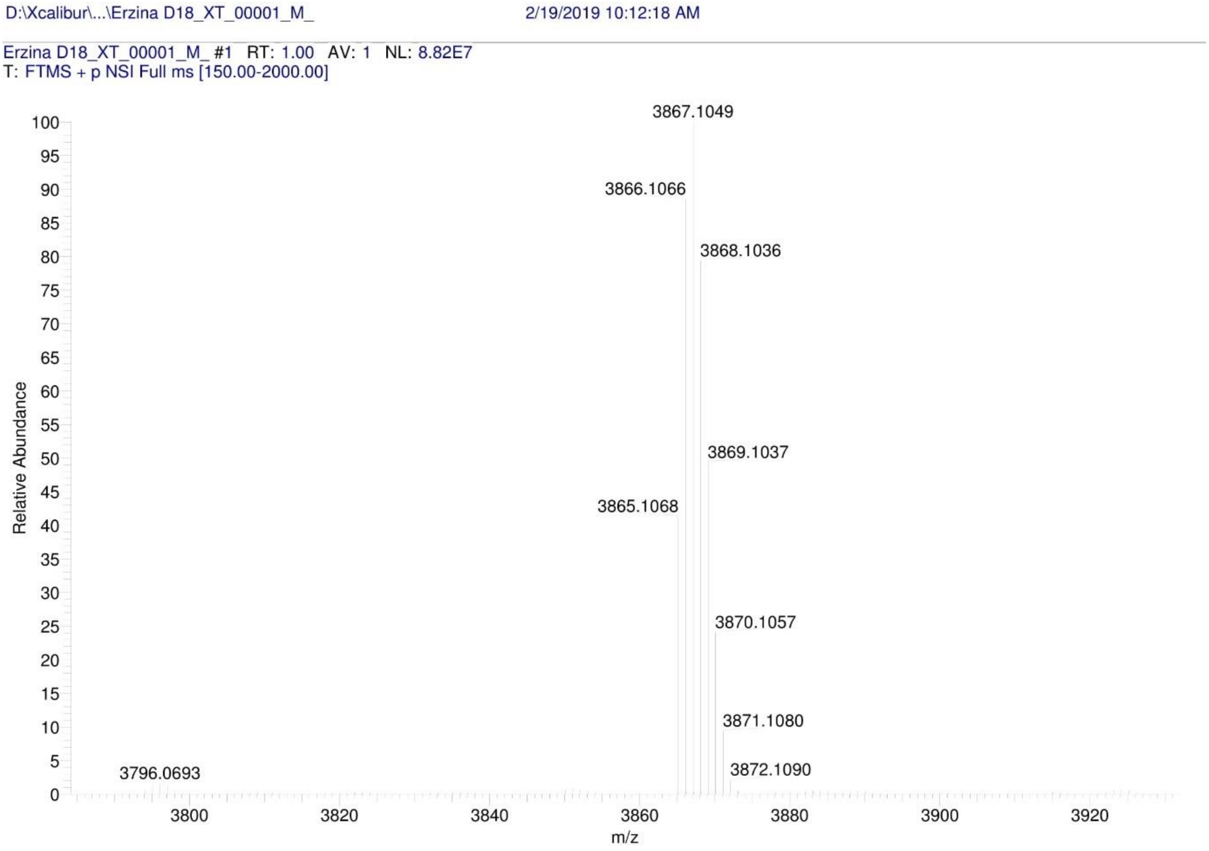
D:\Xcalibur\...\Erzina D18_XT_00001_M_
2/19/2019 10:12:18 AM
Erzina D18_XT_00001_M_ #1 RT: 1.00 AV: 1 NL: 8.82E7
T: FTMS + p NSI Full ms [150.00-2000.00]
3867.1049
3866.1066
3868.1036
3869.1037
3865.1068
3870.1057
3871.1080
3872.1090
3796.0693
Relative Abundance
m/z

26Coum **((AK)$_4$(*K*AEAKAKE)$_2$*K*KEYEYC(Coumarin)A-NH$_2$)** initial dendrimer was obtained as foamy white solid after preparative RP-HPLC, then 1eq. Of dendrimer was coupled with 1.1 eq. of 7-diethylamino-3-[4-(iodoacetamido)phenyl]-4- methylcoumarin in H_2O/ACN solution with NH_4HCO_3 50 mM buffer, pH 8. (6.1 mg, 9.6 µmol, 55.3%). Analytical RP-HPLC: t_R=1.59 min (100% A to 100% D in 5 min, λ= 214 nm). HRMS (ESI+): $C_{198}H_{307}N_{49}O_{54}S$ calc./obs. 4267.2504/4267.2627 Da [M].

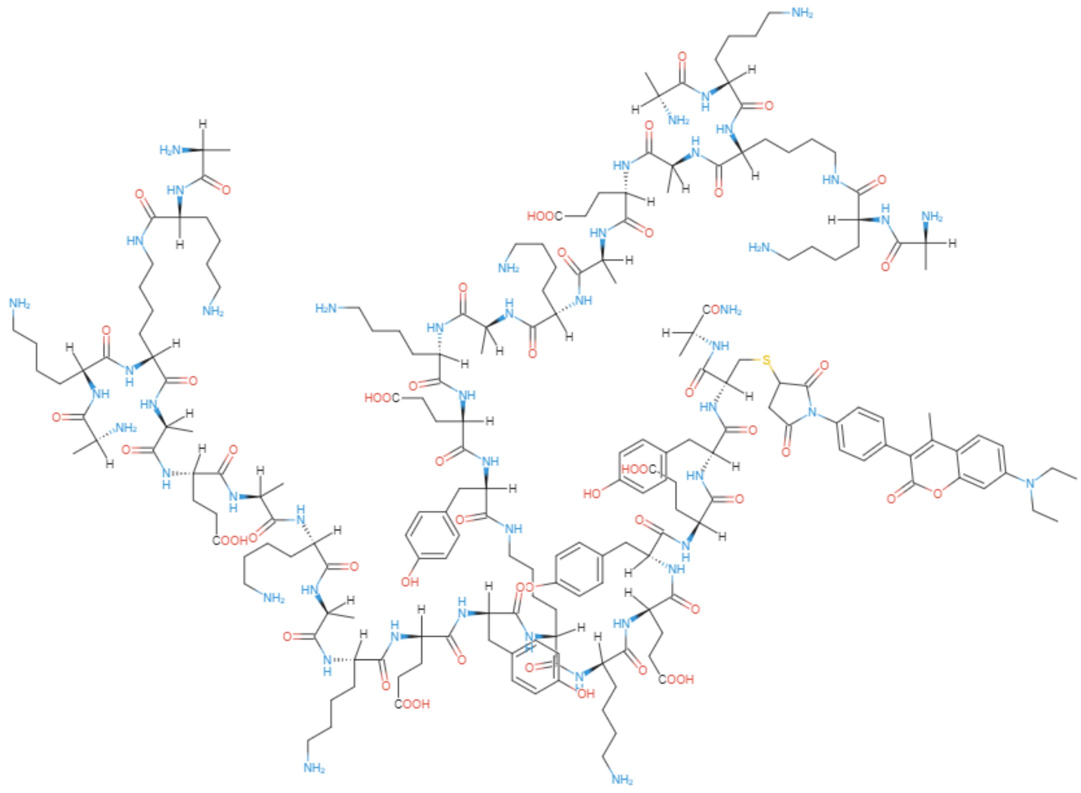

Analytical RP-HPLC:

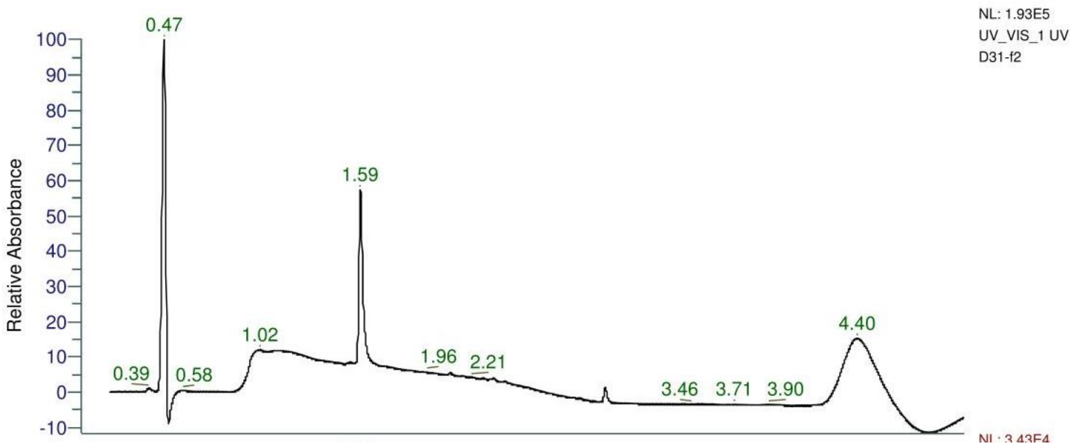

HRMS (NSI+):

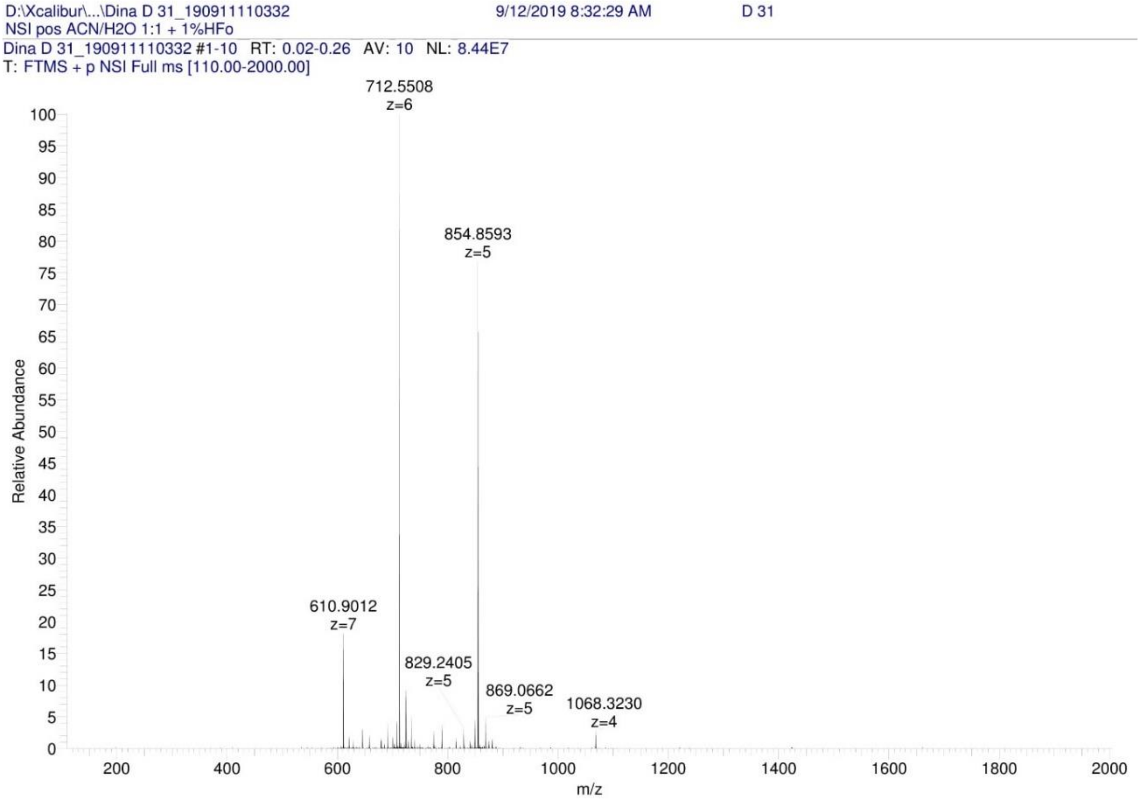
D:\Xcalibur\...\Dina D 31_190911110332
9/12/2019 8:32:29 AM
D 31
NSI pos ACN/H2O 1:1 + 1%HFo
Dina D 31_190911110332 #1-10 RT: 0.02-0.26 AV: 10 NL: 8.44E7
T: FTMS + p NSI Full ms [110.00-2000.00]
712.5508
z=6
854.8593
z=5
610.9012
z=7
829.2405
z=5
869.0662
z=5
1068.3230
z=4
Relative Abundance
m/z

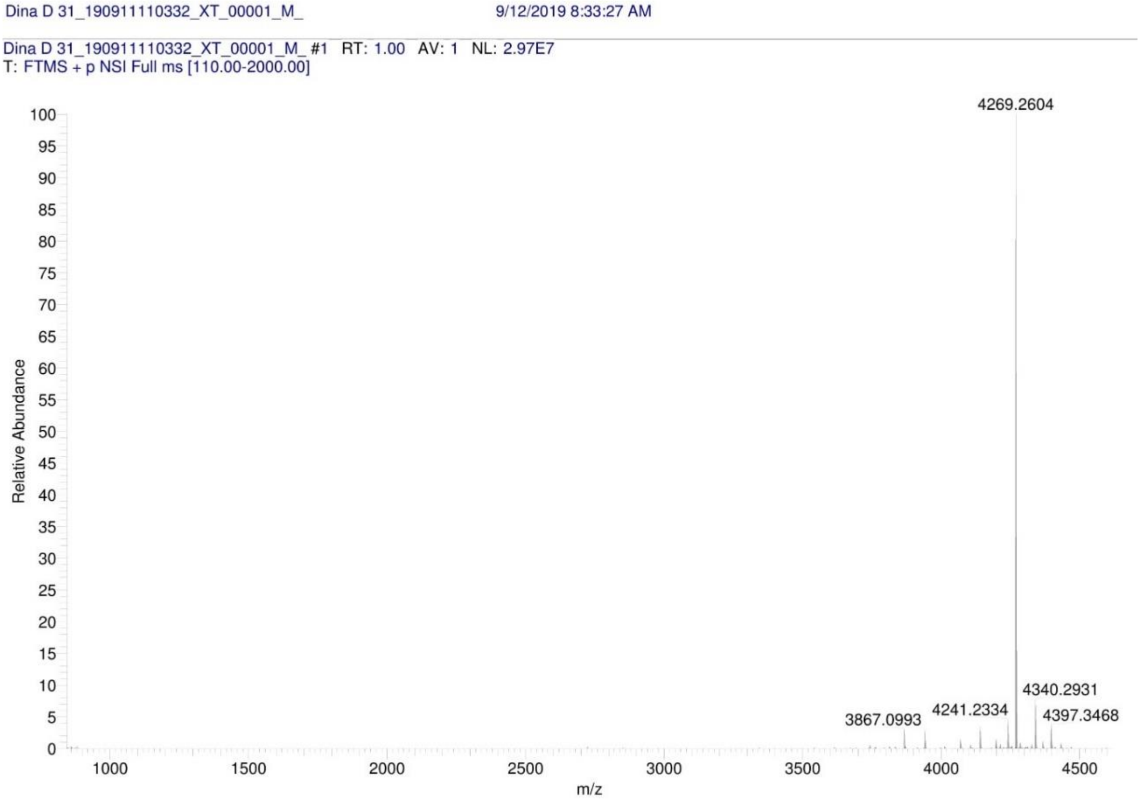
Dina D 31_190911110332_XT_00001_M_
9/12/2019 8:33:27 AM
Dina D 31_190911110332_XT_00001_M_ #1 RT: 1.00 AV: 1 NL: 2.97E7
T: FTMS + p NSI Full ms [110.00-2000.00]
4269.2604
4340.2931
3867.0993
4241.2334
4397.3468
Relative Abundance
m/z

Dina D 31_190911110332_XT_00001_M_ 9/12/2019 8:33:27 AM

Dina D 31_190911110332_XT_00001_M_ #1 RT: 1.00 AV: 1 NL: 2.97E7
T: FTMS + p NSI Full ms [110.00-2000.00]

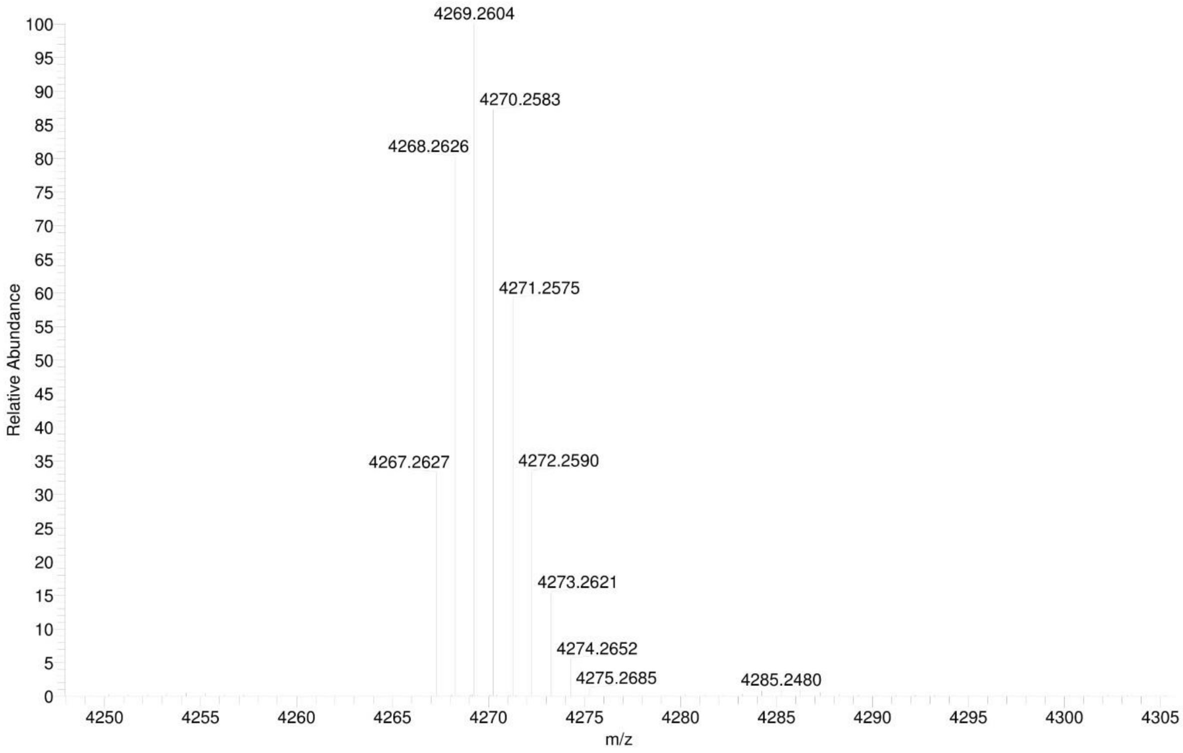

Ac**27 ((AcKEAKY)$_4$(*K*KYEA)$_2$*K*EKAYKA-NH$_2$)** was obtained from the CEM Liberty Blue synthetiser as foamy colourless solid after preparative RP-HPLC (76.6 mg, 5.6 µmol, 34.3%). Analytical RP-HPLC: t_R=1.18 min (100% A to 100% D in 5 min, λ= 214 nm). HRMS (ESI+): $C_{220}H_{343}N_{53}O_{62}$ calc./obs. 4719.5316/4719.5442 Da [M].

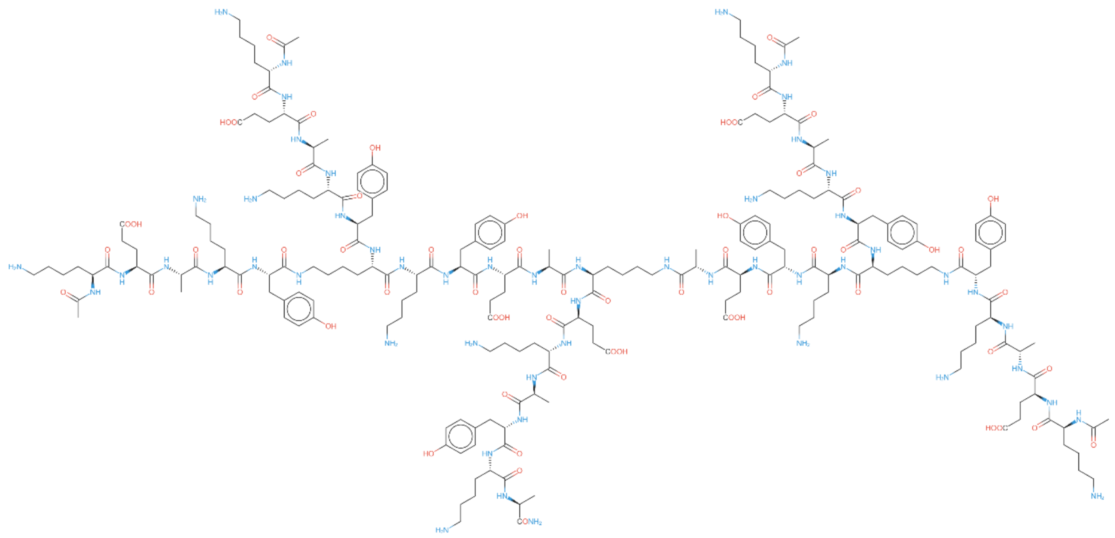

Analytical RP-HPLC:

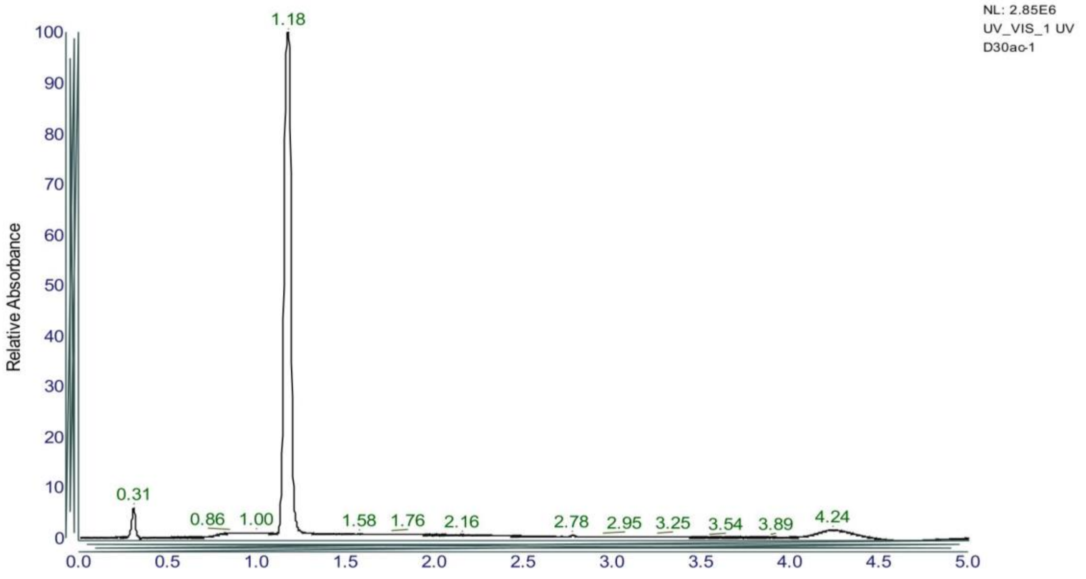

HRMS (NSI+):

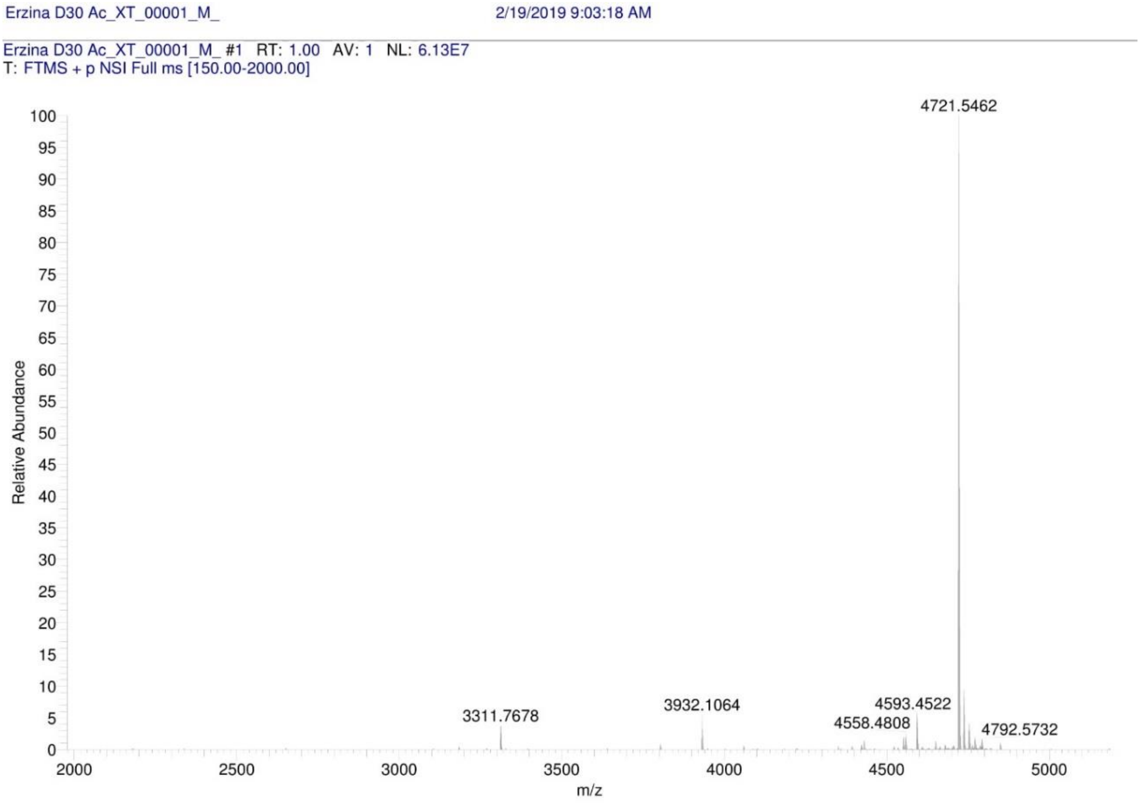
Erzina D30 Ac_XT_00001_M_
2/19/2019 9:03:18 AM
Erzina D30 Ac_XT_00001_M_ #1 RT: 1.00 AV: 1 NL: 6.13E7
T: FTMS + p NSI Full ms [150.00-2000.00]
4721.5462
3311.7678
3932.1064
4593.4522
4558.4808
4792.5732
Relative Abundance
m/z

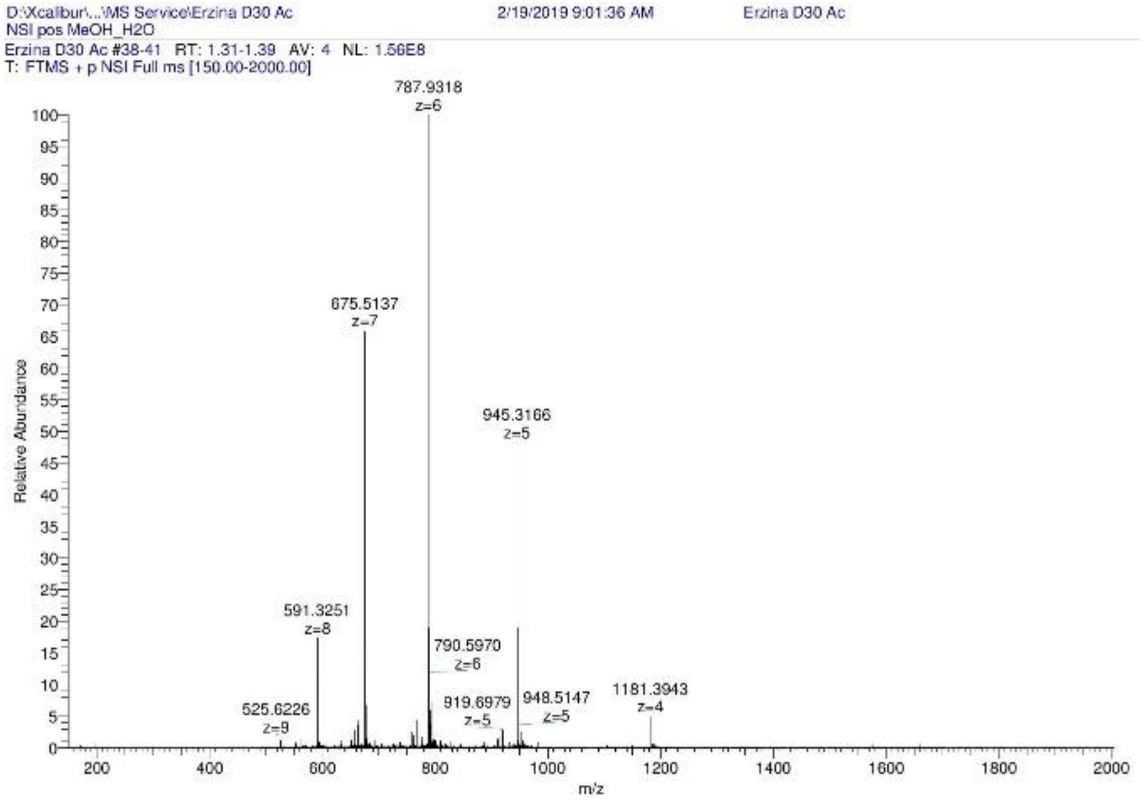
D:\Xcalibur\...\MS Service\Erzina D30 Ac
2/19/2019 9:01:36 AM
Erzina D30 Ac
NSI pos MeOH_H2O
Erzina D30 Ac #38-41 RT: 1.31-1.39 AV: 4 NL: 1.56E8
T: FTMS + p NSI Full ms [150.00-2000.00]
787.9318 z=6
675.5137 z=7
945.3166 z=5
591.3251 z=8
790.5970 z=6
525.6226 z=9
919.6979 z=5
948.5147 z=5
1181.3943 z=4
Relative Abundance
m/z

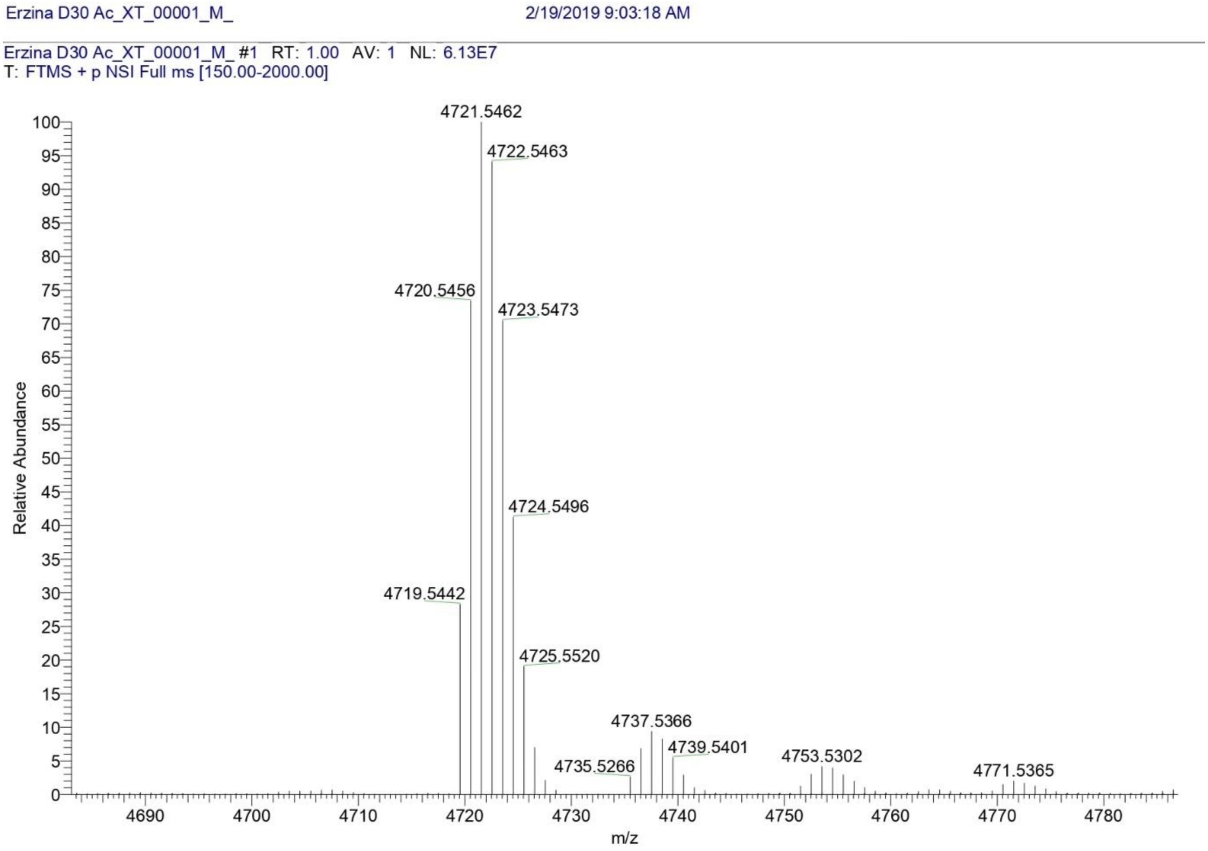
Erzina D30 Ac_XT_00001_M_
2/19/2019 9:03:18 AM
Erzina D30 Ac_XT_00001_M_ #1 RT: 1.00 AV: 1 NL: 6.13E7
T: FTMS + p NSI Full ms [150.00-2000.00]
4721.5462
4722.5463
4720.5456
4723.5473
4724.5496
4719.5442
4725.5520
4737.5366
4739.5401
4735.5266
4753.5302
4771.5365
Relative Abundance
m/z

ClAc**27 ((ClAcKEAKY)$_4$(*K*KYEA)$_2$*K*EKAYKA-NH$_2$)** was obtained from the CEM Liberty Blue synthetiser as foamy colourless solid after preparative RP-HPLC (56.7 mg, 4.0 µmol, 24.8%). Analytical RP-HPLC: t_R=1.21 min (100% A to 100% D in 5 min, λ= 214 nm). HRMS (ESI+): $C_{220}H_{339}N_{52}Cl_4O_{62}$ calc./obs. 4855.3757/4857.3959 Da [M].

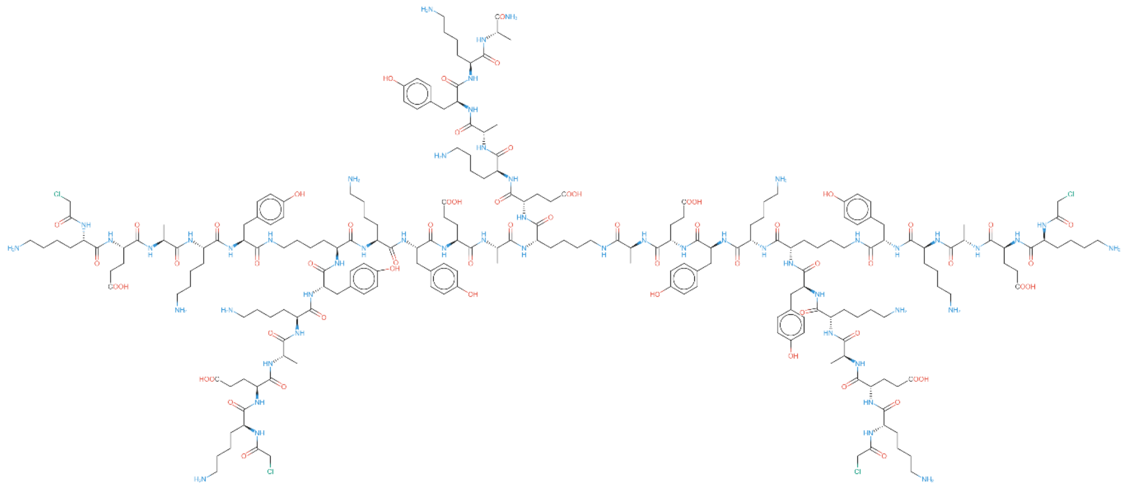

Analytical RP-HPLC:

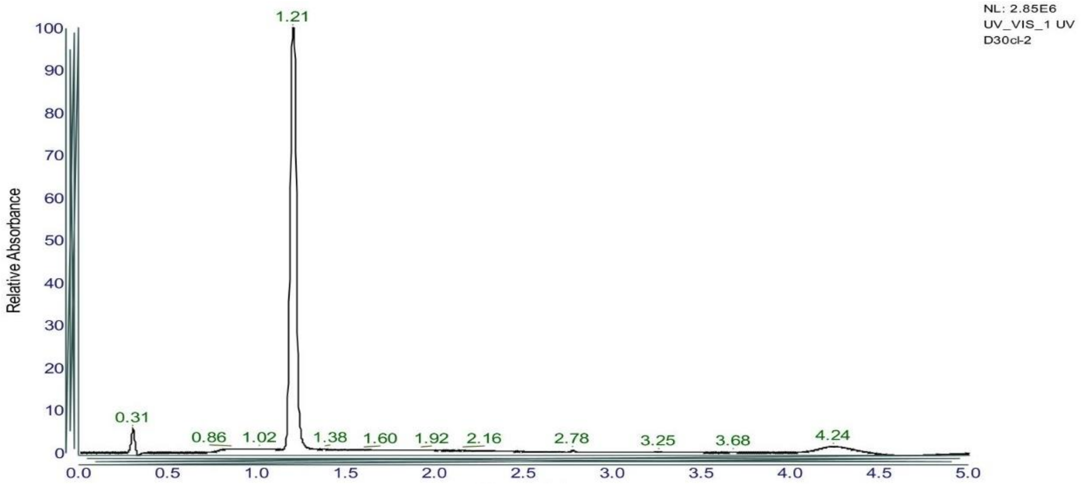

HRMS (NSI+):

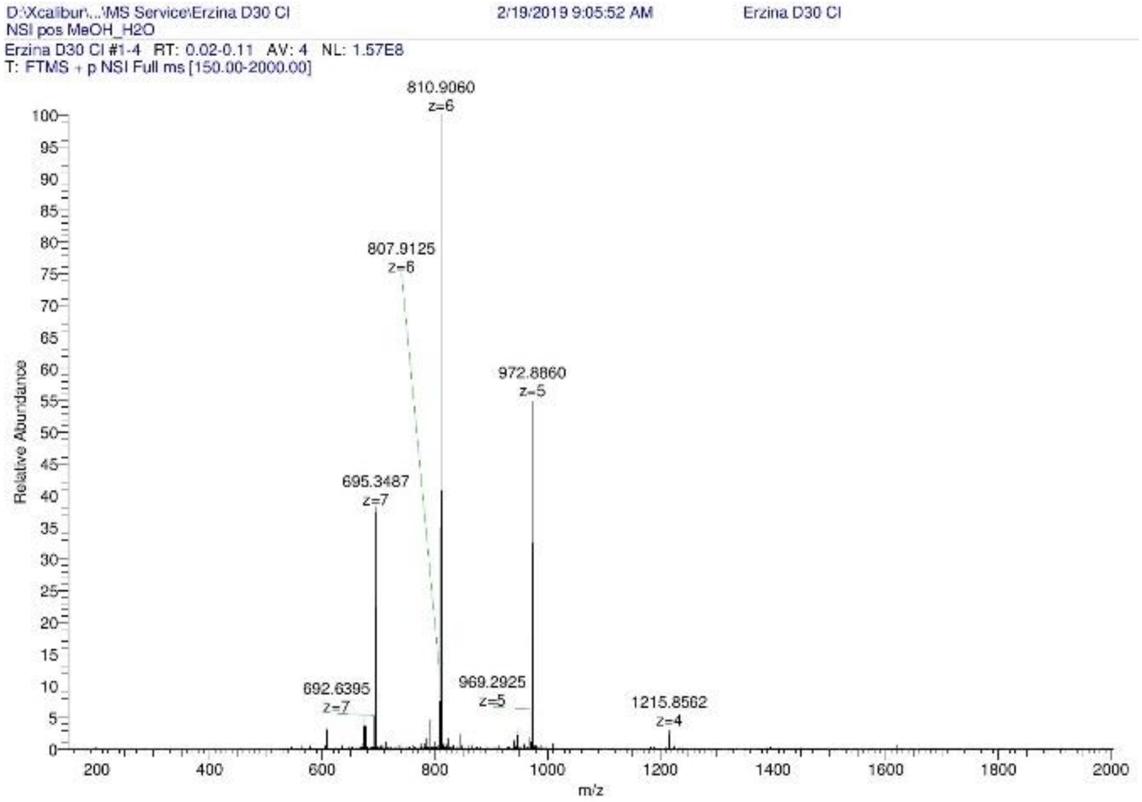
D:\Xcalibur\...\MS Service\Erzina D30 Cl
2/19/2019 9:05:52 AM
Erzina D30 Cl
NSI pos MeOH_H2O
Erzina D30 Cl #1-4 RT: 0.02-0.11 AV: 4 NL: 1.57E8
T: FTMS + p NSI Full ms [150.00-2000.00]
810.9060
z=6
807.9125
z=6
972.8860
z=5
695.3487
z=7
692.6395
z=7
969.2925
z=5
1215.8562
z=4
Relative Abundance
m/z

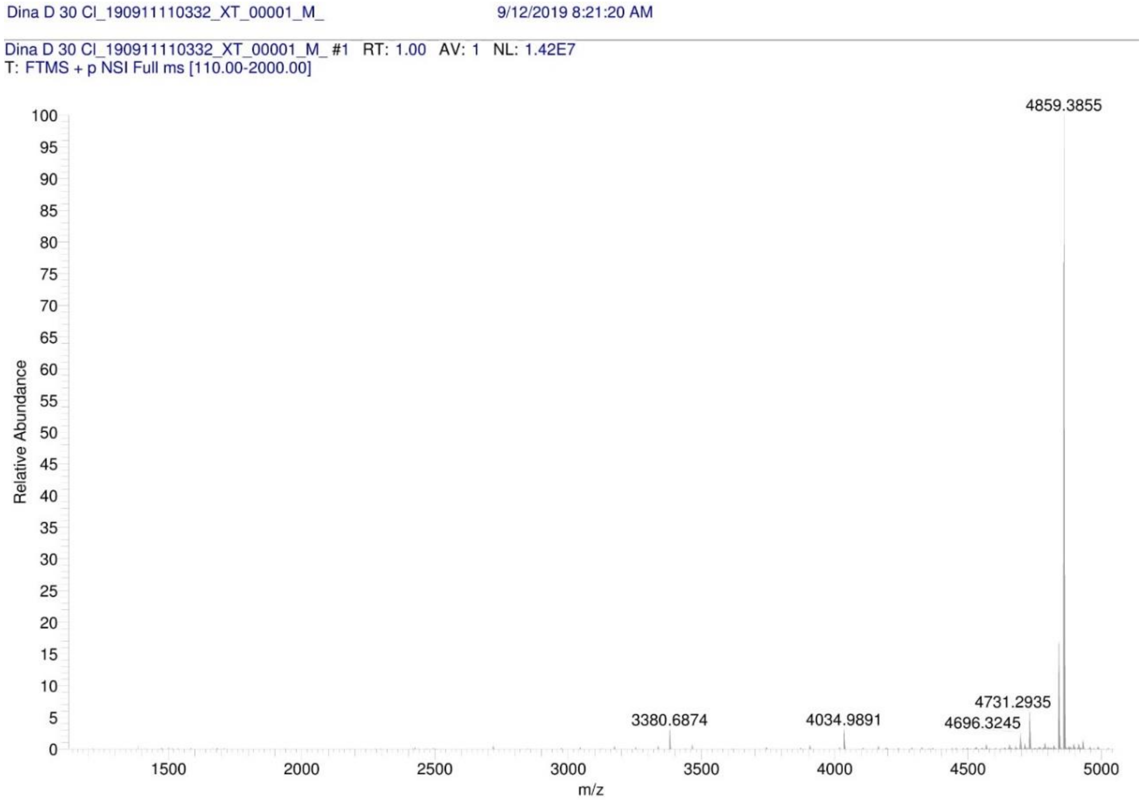
Dina D 30 Cl_190911110332_XT_00001_M_
9/12/2019 8:21:20 AM
Dina D 30 Cl_190911110332_XT_00001_M_ #1 RT: 1.00 AV: 1 NL: 1.42E7
T: FTMS + p NSI Full ms [110.00-2000.00]
4859.3855
4731.2935
4696.3245
3380.6874
4034.9891
Relative Abundance
m/z

Erzina D30 Cl_XT_00001_M_ 2/19/2019 9:06:19 AM

Erzina D30 Cl_XT_00001_M_ #1 RT: 1.00 AV: 1 NL: 5.46E7
T: FTMS + p NSI Full ms [150.00-2000.00]

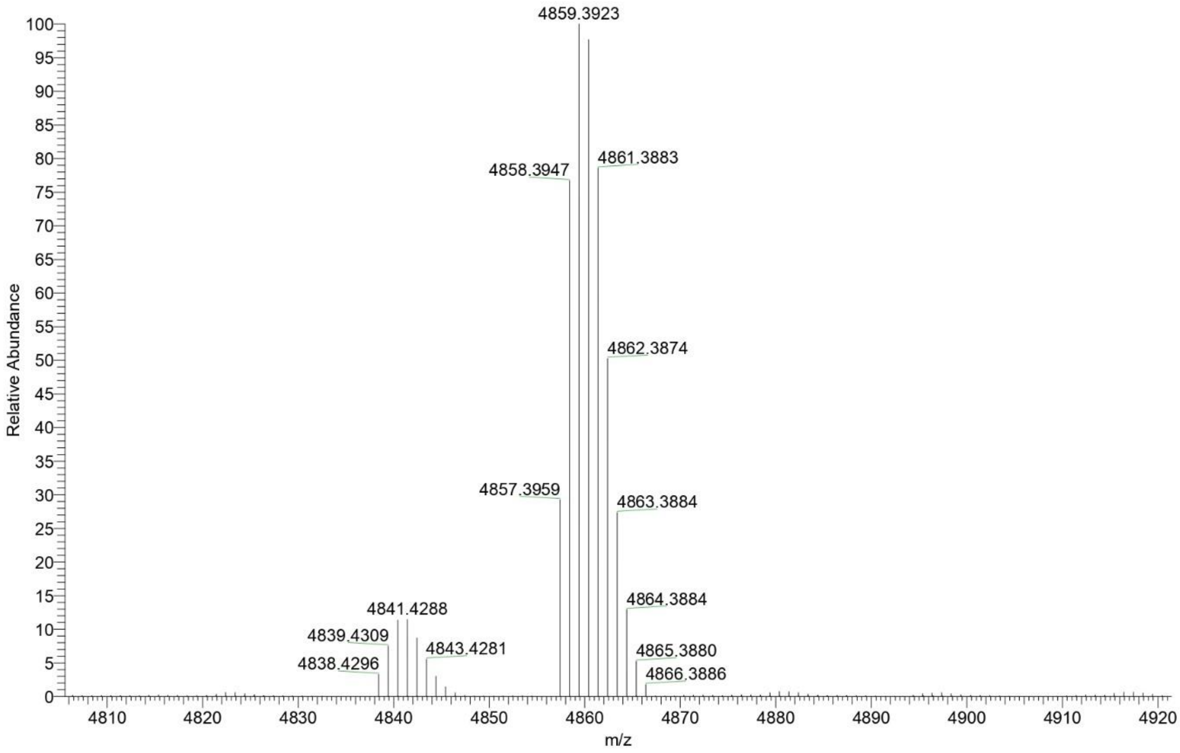

28 ((YAKAKE)$_4$(*K*AYKAKA)$_2$*K*AYKKA-NH$_2$) was obtained after manual synthesis as foamy colourless solid after preparative RP-HPLC (169.6 mg, 3.5 µmol, 23.4%). Analytical RP-HPLC: t_R=1.23 min (100% A to 100% D in 5 min, λ= 214 nm). HRMS (ESI+): $C_{233}H_{378}N_{62}O_{59}$ calc./obs. 4988.8484/4988.8659 Da [M].

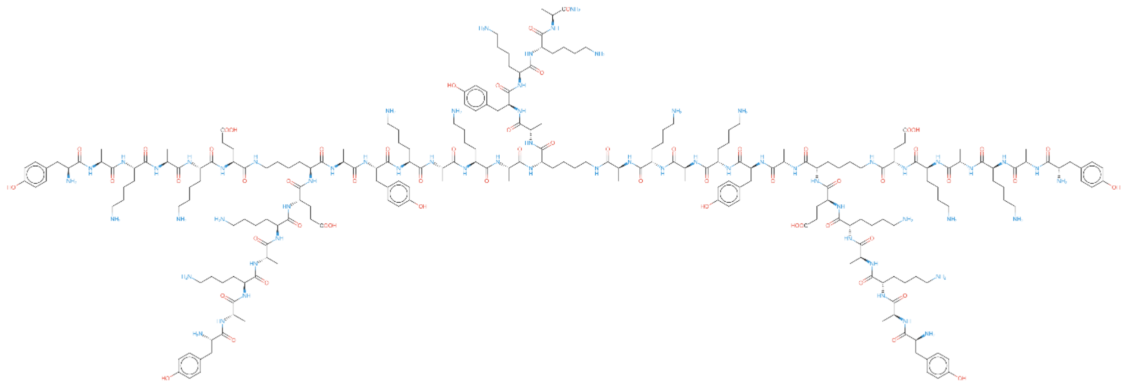

Analytical RP-HPLC:

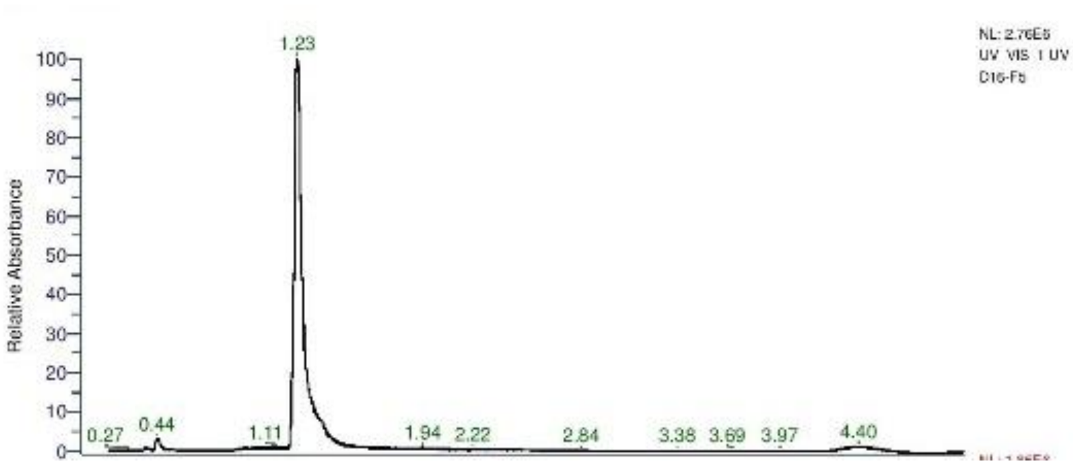

HRMS (NSI+):

Dina D 16_190911110332_XT_00001_M_ 9/11/2019 4:35:37 PM

Dina D 16_190911110332_XT_00001_M_ #1 RT: 1.00 AV: 1 NL: 4.16E7
T: FTMS + p NSI Full ms [110.00-2000.00]

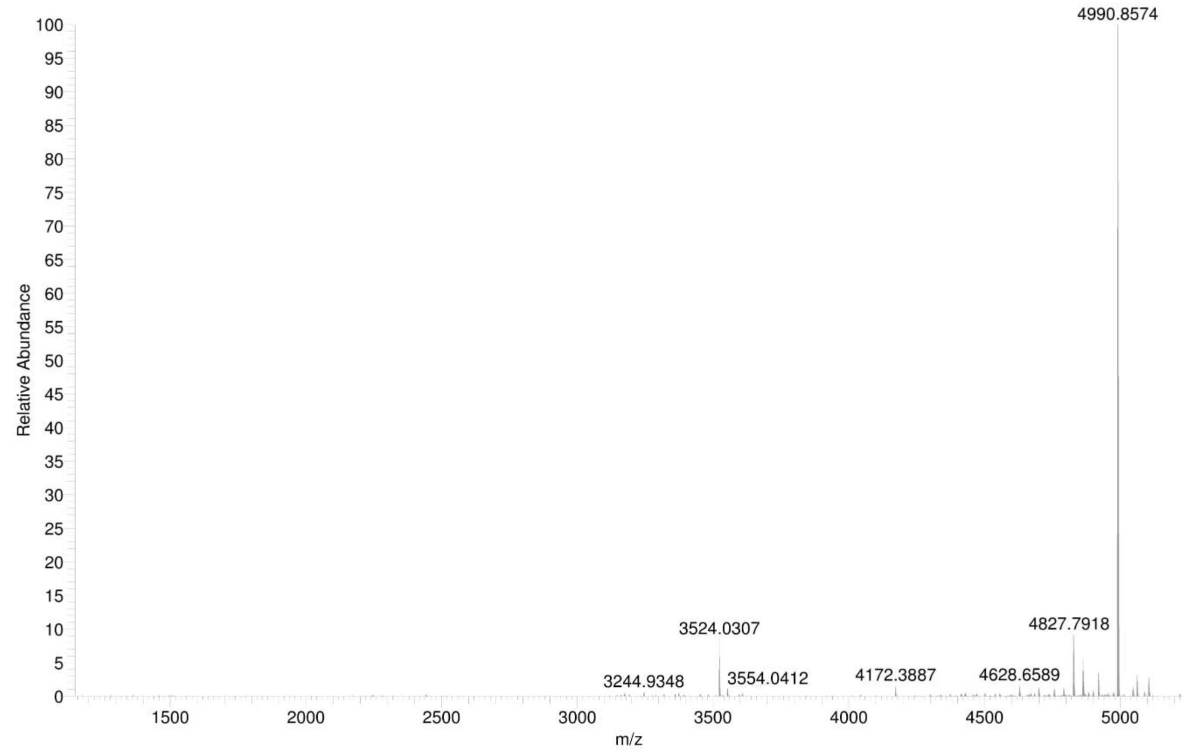

D:\Xcalibur\...\Dina D 16_190911110332 9/11/2019 4:34:34 PM D 16
NSI pos ACN/H2O 1:1 + 1%HFo

Dina D 16_190911110332 #1-11 RT: 0.02-0.30 AV: 11 NL: 1.18E8
T: FTMS + p NSI Full ms [110.00-2000.00]

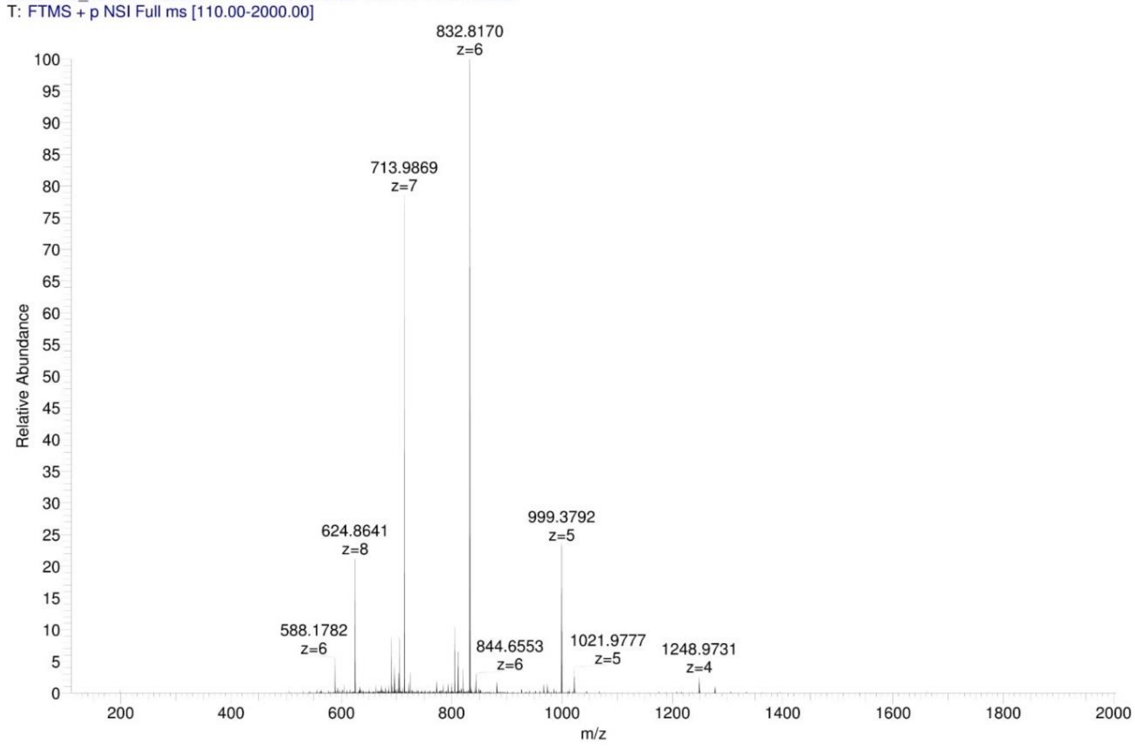

D:\Xcalibur\...\Erzina D16_XT_00001_M_ 2/19/2019 10:19:11 AM

Erzina D16_XT_00001_M_ #1 RT: 1.00 AV: 1 NL: 9.30E7
T: FTMS + p NSI Full ms [150.00-2000.00]

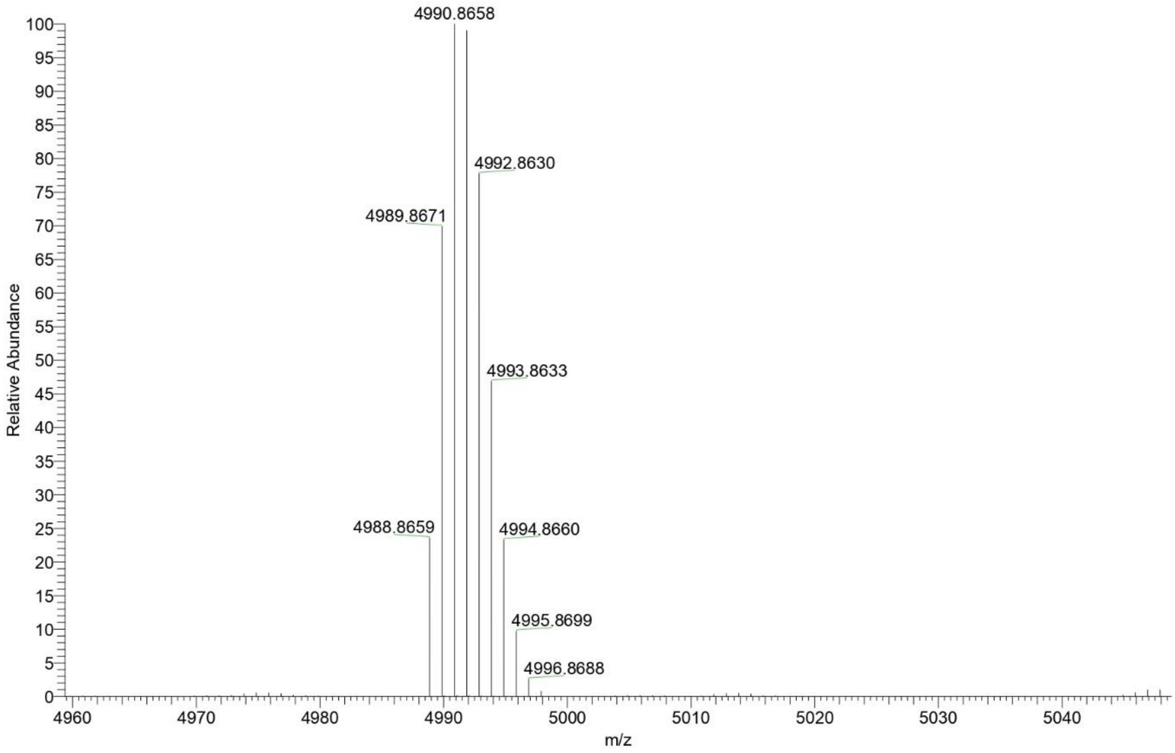

29 ((YKAKAKY)$_4$(*K*EAKAKY)$_2$*K*AKEYEY-OH) was obtained after manual synthesis as foamy colourless solid after preparative RP-HPLC (154 mg, 1.4 µmol, 12.3%). Analytical RP-HPLC: t_R=1.14 min (100% A to 100% D in 5 min, λ= 214 nm). HRMS (ESI+): $C_{287}H_{443}N_{69}O_{70}$ calc./obs. 5976.3226/5976.3381 Da [M].

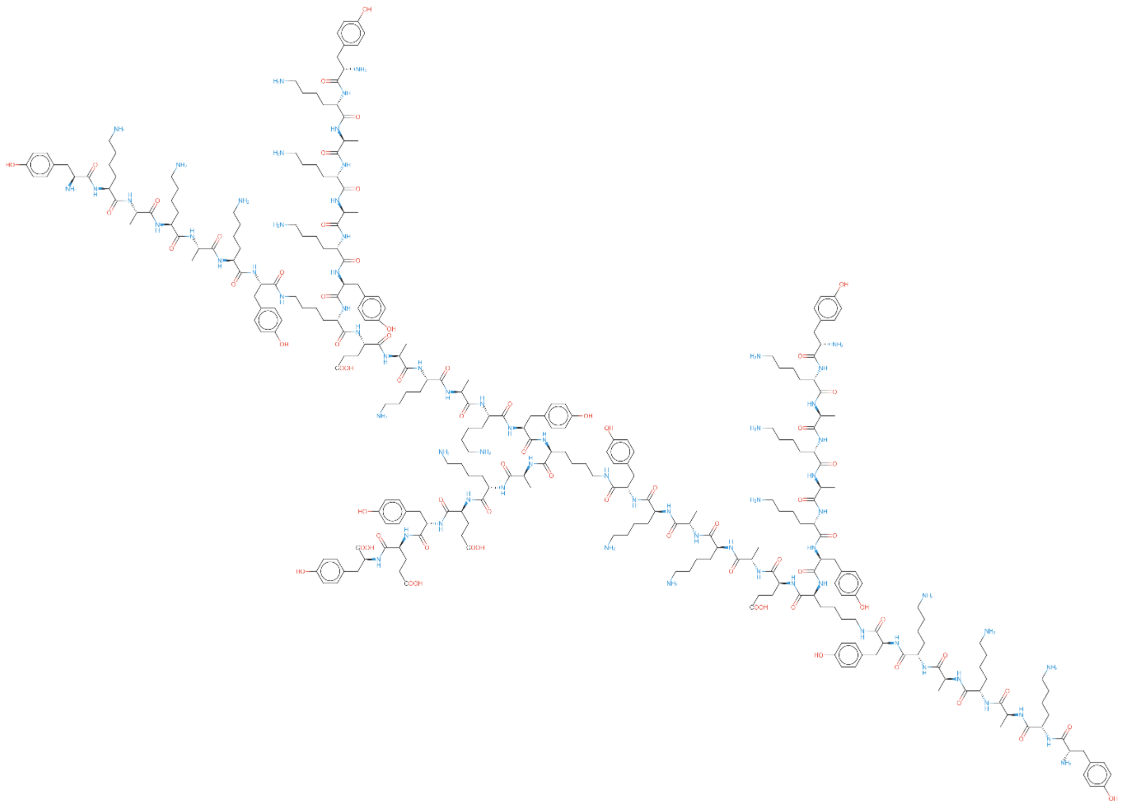

Analytical RP-HPLC:

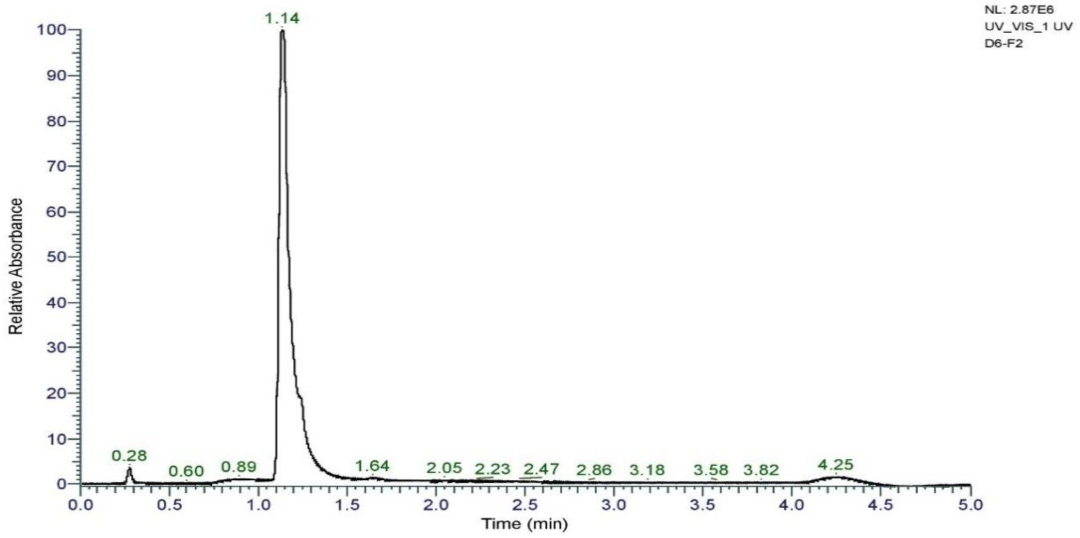

HRMS (NSI+):

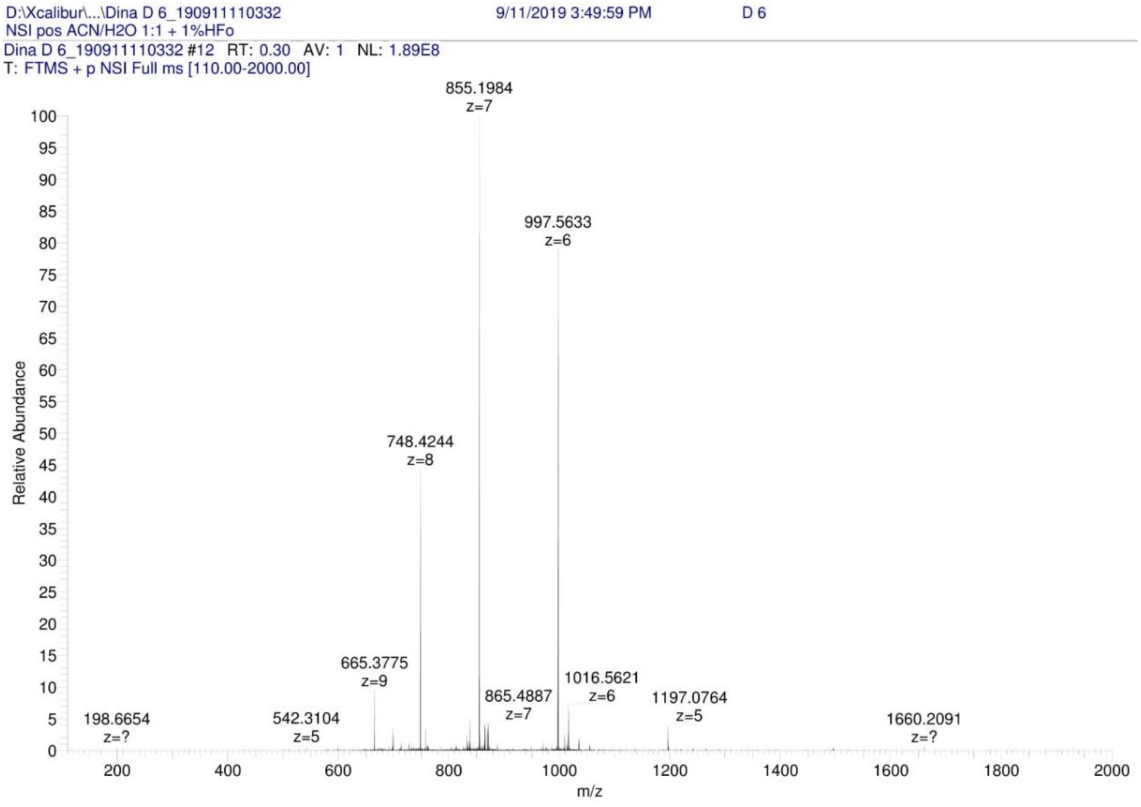

D:\Xcalibur\...\Dina D 6_190911110332
9/11/2019 3:49:59 PM
D 6
NSI pos ACN/H2O 1:1 + 1%HFo
Dina D 6_190911110332 #12 RT: 0.30 AV: 1 NL: 1.89E8
T: FTMS + p NSI Full ms [110.00-2000.00]
855.1984 z=7
997.5633 z=6
748.4244 z=8
665.3775 z=9
865.4887 z=7
1016.5621 z=6
1197.0764 z=5
198.6654 z=?
542.3104 z=5
1660.2091 z=?
Relative Abundance
m/z

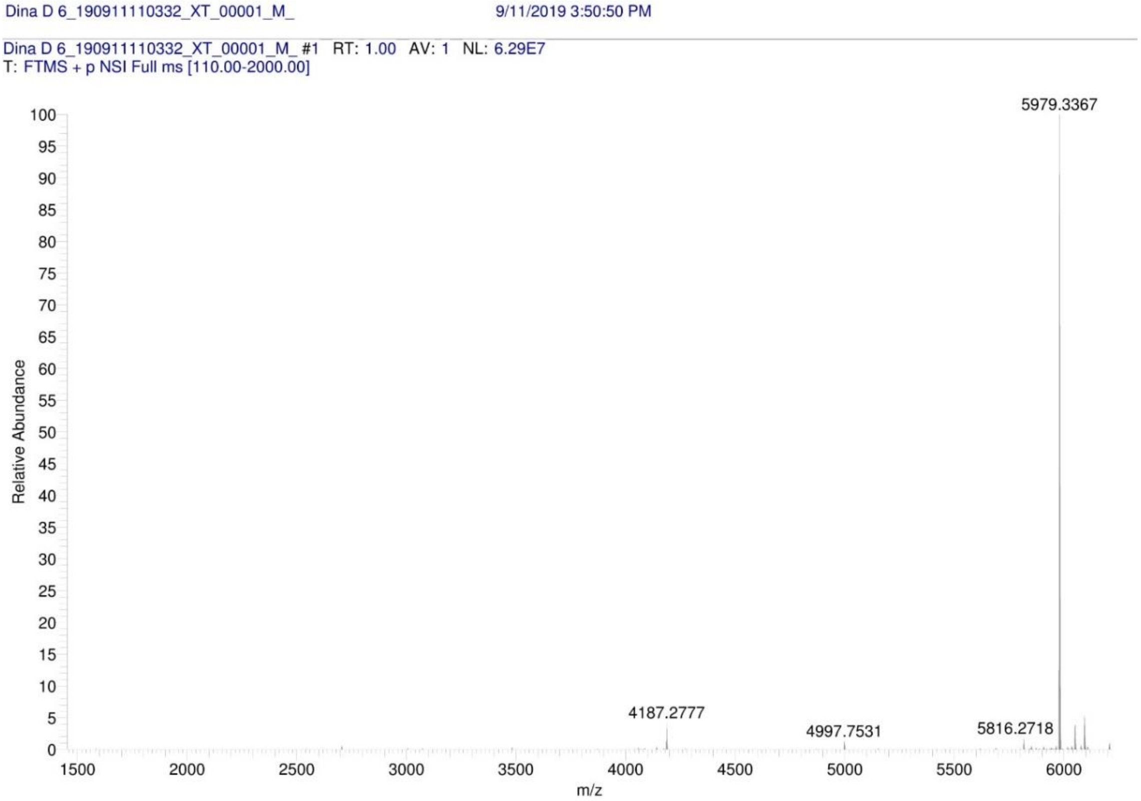

Dina D 6_190911110332_XT_00001_M_
9/11/2019 3:50:50 PM
Dina D 6_190911110332_XT_00001_M_ #1 RT: 1.00 AV: 1 NL: 6.29E7
T: FTMS + p NSI Full ms [110.00-2000.00]
5979.3367
4187.2777
4997.7531
5816.2718
Relative Abundance
m/z

Dina D 6_190911110332_XT_00001_M_ 9/11/2019 3:50:50 PM

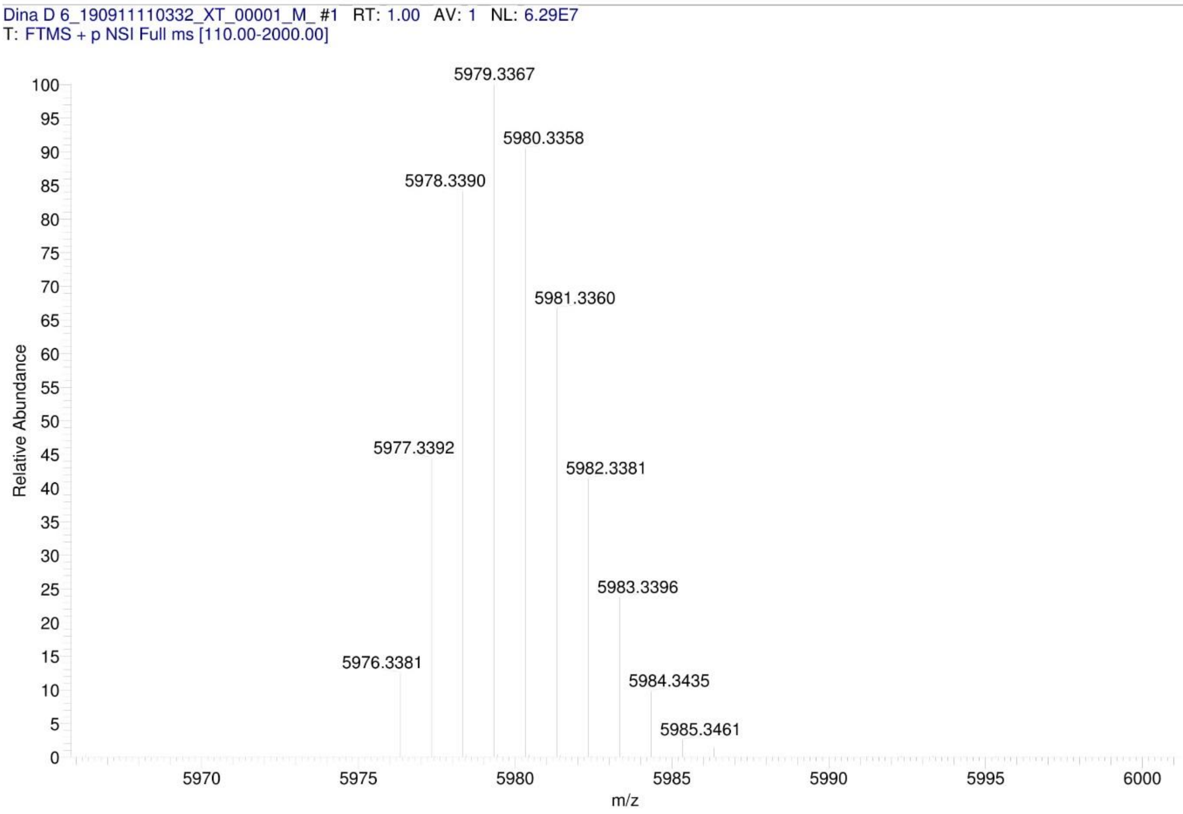

30 ((KAA)$_8$(*K*KAKAK)$_4$(*K*AAKKY)$_2$*K*EKAKCA-OH) was obtained after manual synthesis as foamy colourless solid after preparative RP-HPLC (17 mg, 0.2 µmol, 2.6%). Analytical RP-HPLC: t_R=1.21 min (100% A to 100% D in 5 min, λ= 214 nm). HRMS (ESI+): $C_{315}H_{580}N_{100}O_{71}S$ calc./obs. 6934.4362/6934.4379 Da [M].

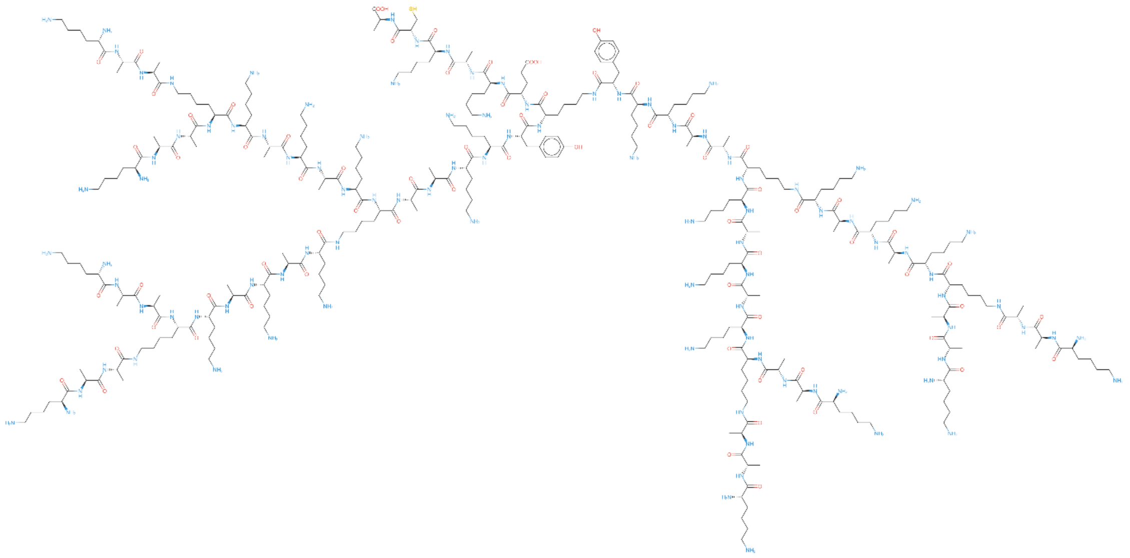

Analytical RP-HPLC:

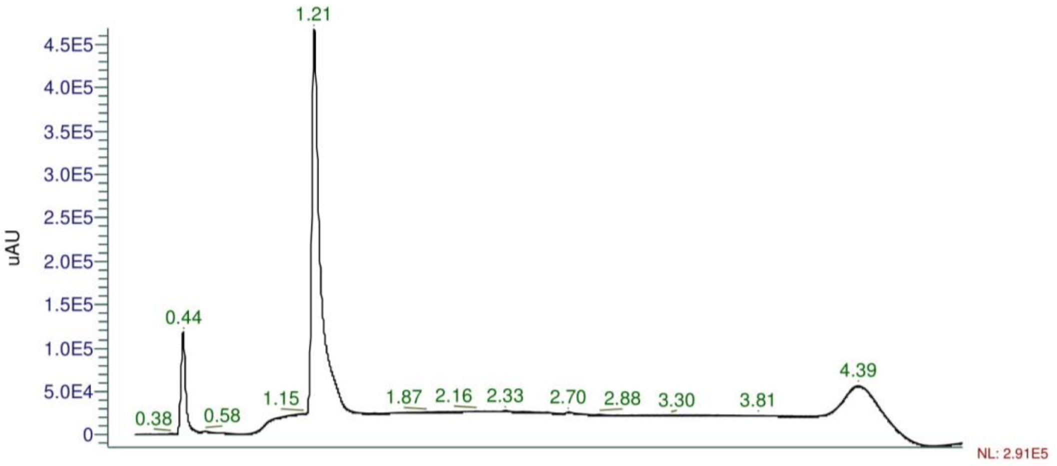

HRMS (NSI+):

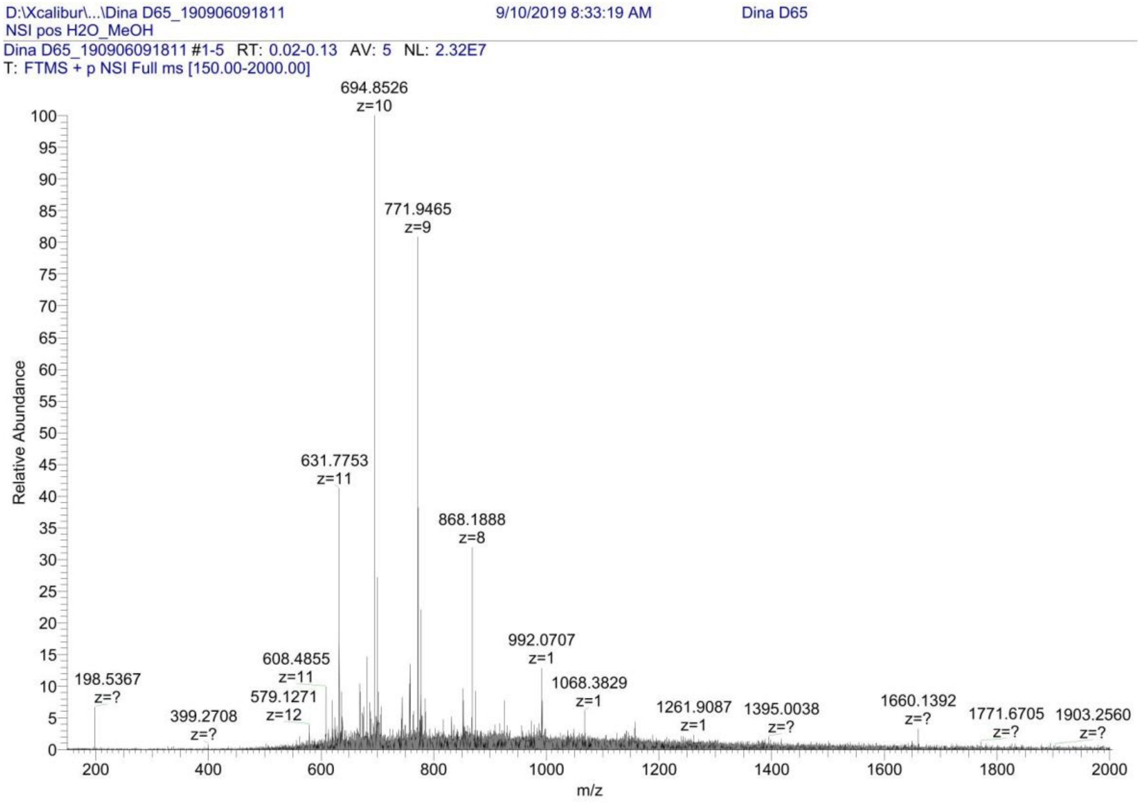
D:\Xcalibur\...\Dina D65_190906091811
9/10/2019 8:33:19 AM
Dina D65
NSI pos H2O_MeOH
Dina D65_190906091811 #1-5 RT: 0.02-0.13 AV: 5 NL: 2.32E7
T: FTMS + p NSI Full ms [150.00-2000.00]
694.8526 z=10
771.9465 z=9
631.7753 z=11
868.1888 z=8
992.0707 z=1
608.4855 z=11
1068.3829 z=1
198.5367 z=?
579.1271 z=12
399.2708 z=?
1261.9087 z=1
1395.0038 z=?
1660.1392 z=?
1771.6705 z=?
1903.2560 z=?
Relative Abundance
m/z

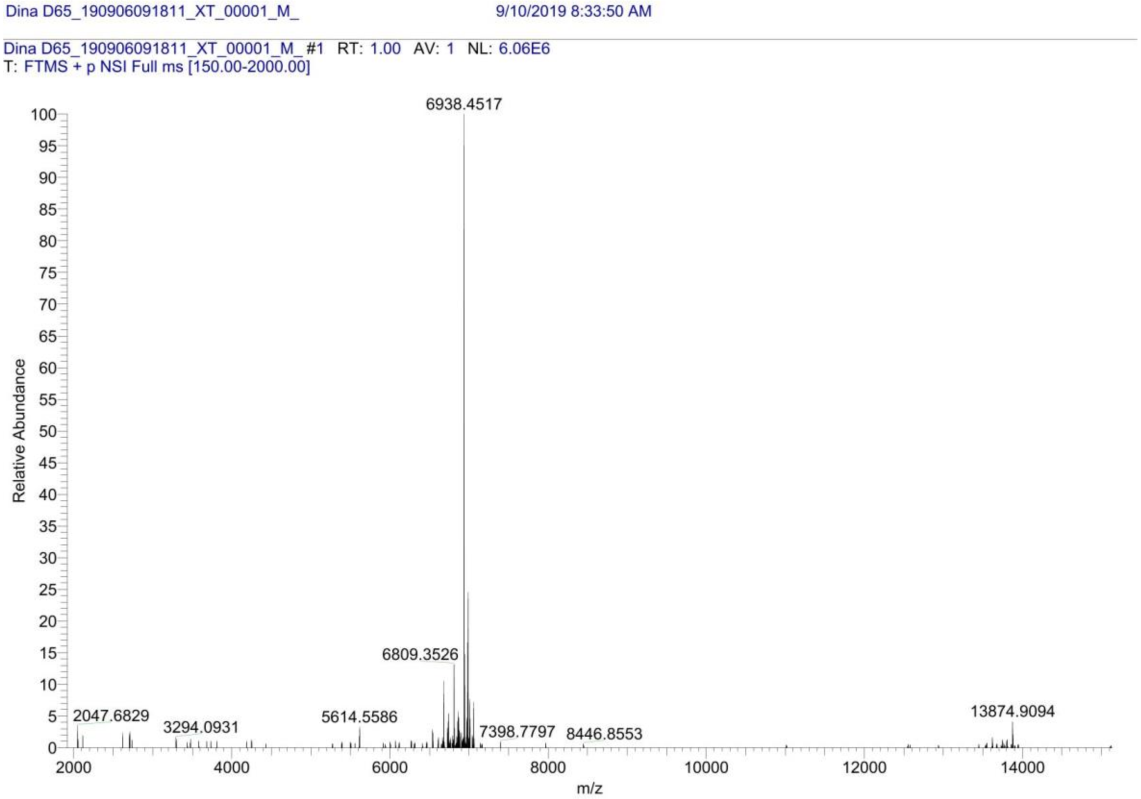
Dina D65_190906091811_XT_00001_M_
9/10/2019 8:33:50 AM
Dina D65_190906091811_XT_00001_M_ #1 RT: 1.00 AV: 1 NL: 6.06E6
T: FTMS + p NSI Full ms [150.00-2000.00]
6938.4517
6809.3526
2047.6829
3294.0931
5614.5586
7398.7797
8446.8553
13874.9094
Relative Abundance
m/z

Dina D65_190906091811_XT_00001_M_ 9/10/2019 8:33:50 AM

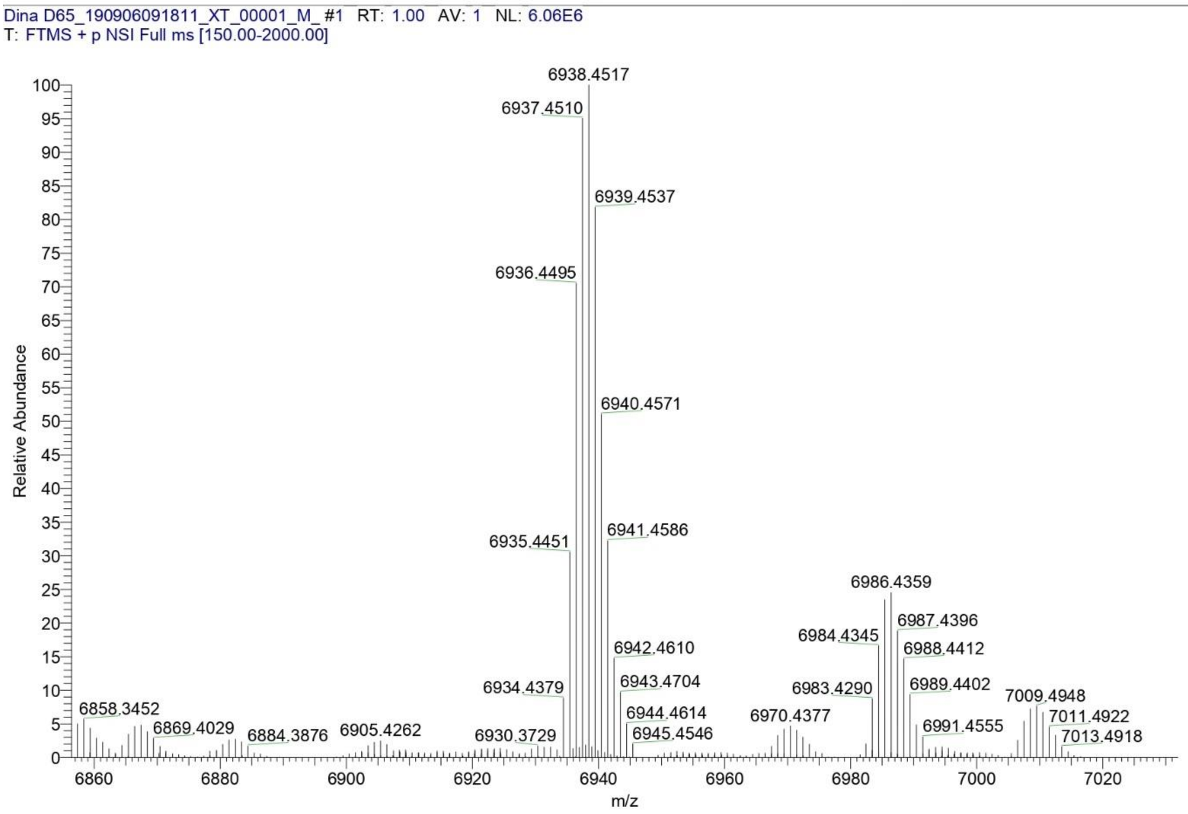

31 ((X)$_{30}$-NH$_2$) was synthetised using the CEM Liberty Blue synthetiser by mixing Fmoc-L-alanine, Fmoc-L-lysine, Fmoc-L-glutamic acid, Fmoc-L-tyrosine in ratio 4.2/3.4/1.4/1 respectively. Colourless foamy solid was obtained after preparative RP-HPLC (46.3 mg). Analytical RP-HPLC: t_R=2.51- min (100% A to 100% D in 10 min, λ= 214 nm). Amino acid analysis:

Analytical RP-HPLC:

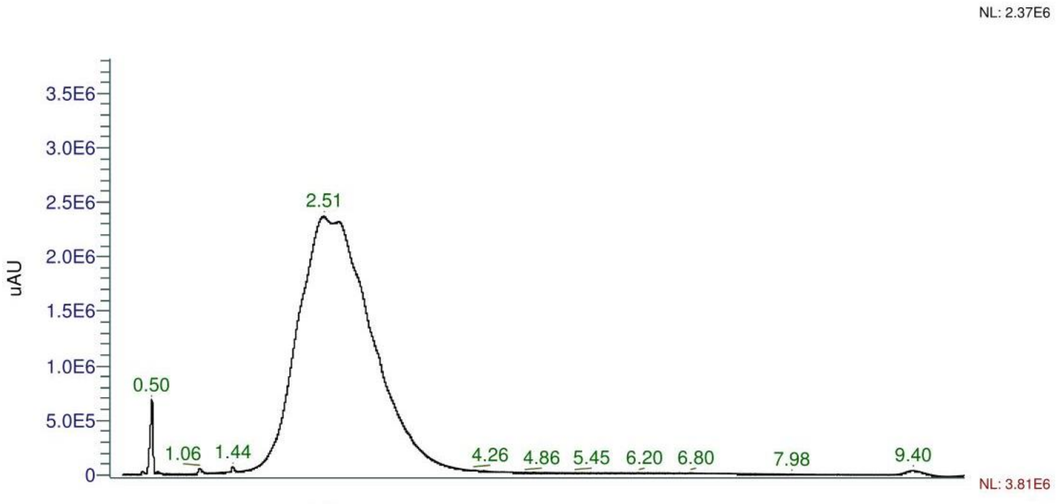

HRMS (NSI+):

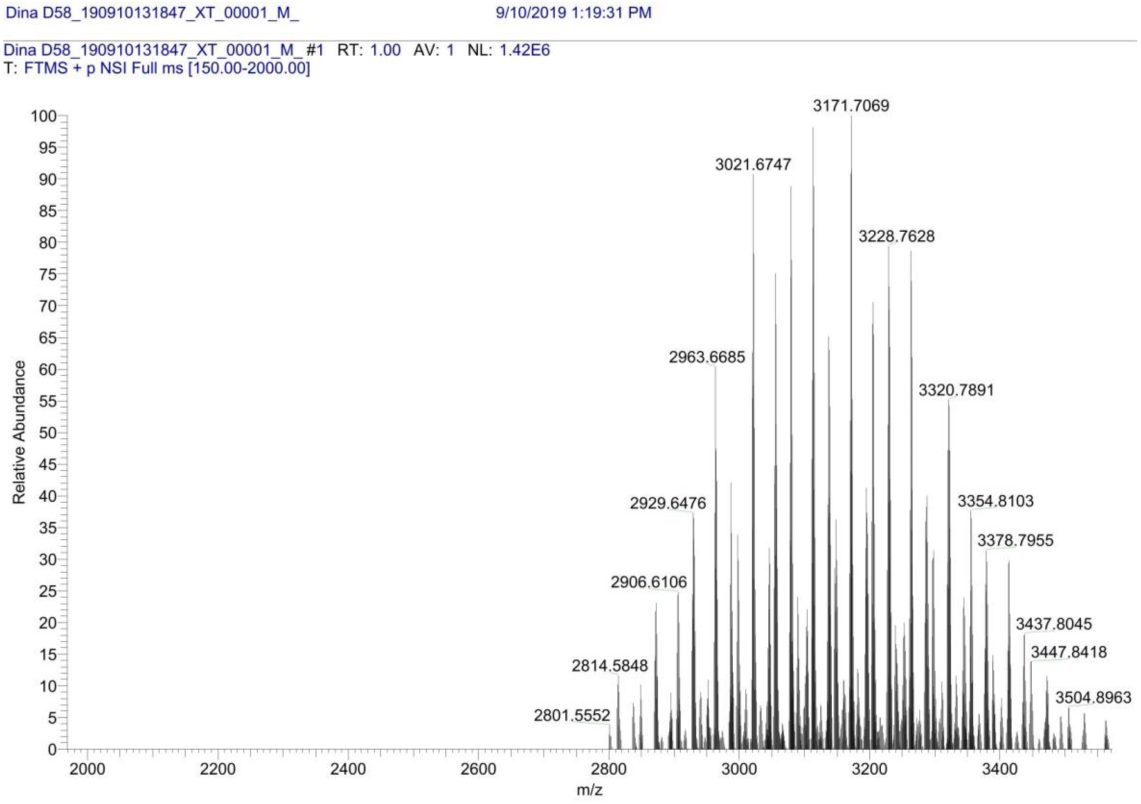

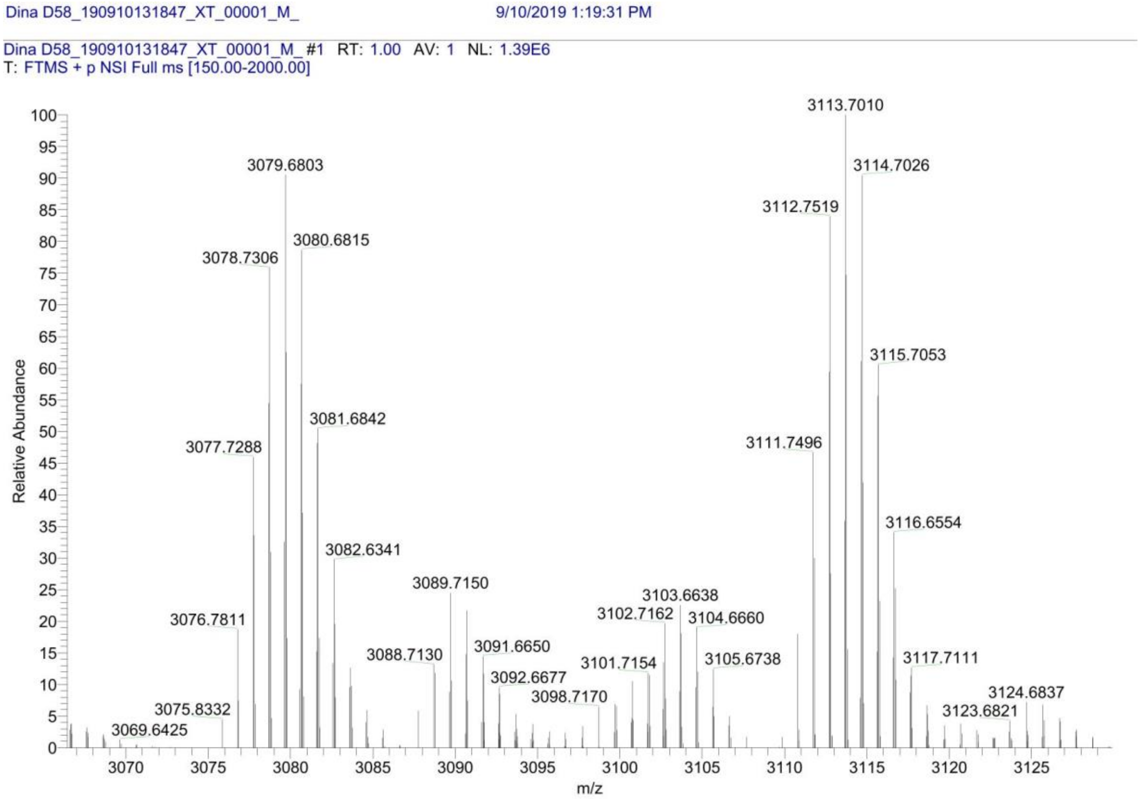

Dina D58_190910131847_XT_00001_M_
9/10/2019 1:19:31 PM
Dina D58_190910131847_XT_00001_M_ #1 RT: 1.00 AV: 1 NL: 1.39E6
T: FTMS + p NSI Full ms [150.00-2000.00]
Relative Abundance
m/z
3113.7010
3079.6803
3114.7026
3112.7519
3080.6815
3078.7306
3115.7053
3081.6842
3077.7288
3111.7496
3116.6554
3082.6341
3089.7150
3103.6638
3102.7162
3104.6660
3076.7811
3091.6650
3088.7130
3105.6738
3101.7154
3117.7111
3092.6677
3098.7170
3124.6837
3075.8332
3123.6821
3069.6425

ClAc31 ($ClAc(X)_{30}$-NH_2) was synthetised using the CEM Liberty Blue synthetiser by mixing Fmoc-L-alanine, Fmoc-L-lysine, Fmoc-L-glutamic acid, Fmoc-L-tyrosine in ratio 4.2/3.4/1.4/1 respectively. Colourless foamy solid was obtained after preparative RP-HPLC (34.0 mg). Analytical RP-HPLC: t_R=2.76- min (100% A to 100% D in 10 min, λ= 214 nm).

Analytical RP-HPLC:

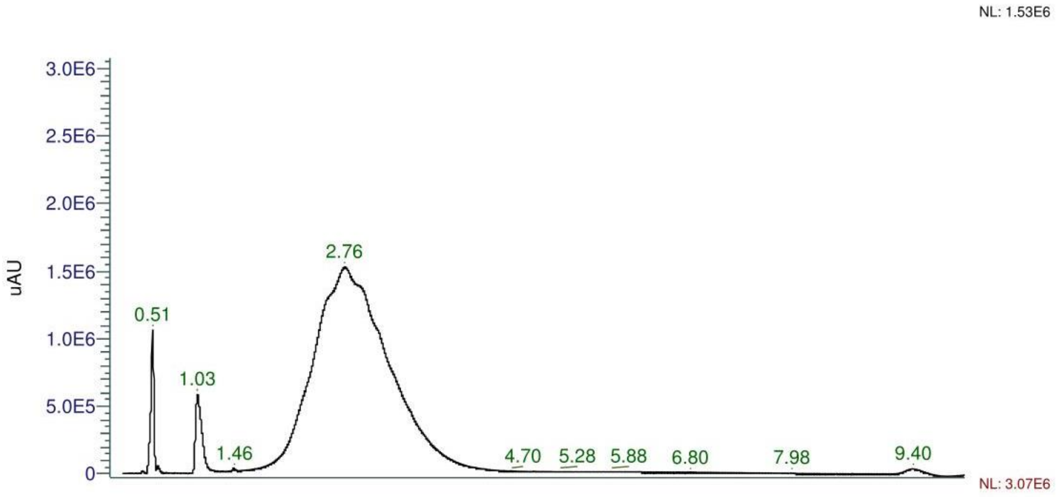

HRMS (NSI+):

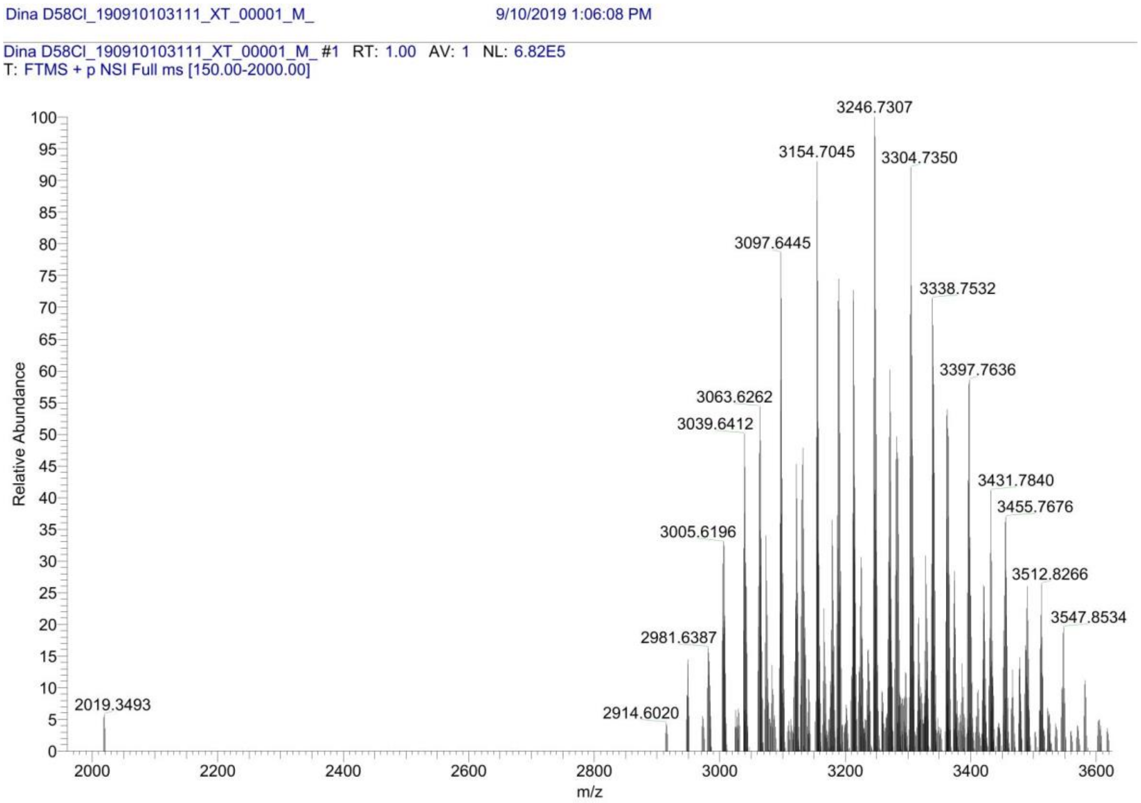

Dina D58Cl_190910103111_XT_00001_M_ 9/10/2019 1:06:08 PM

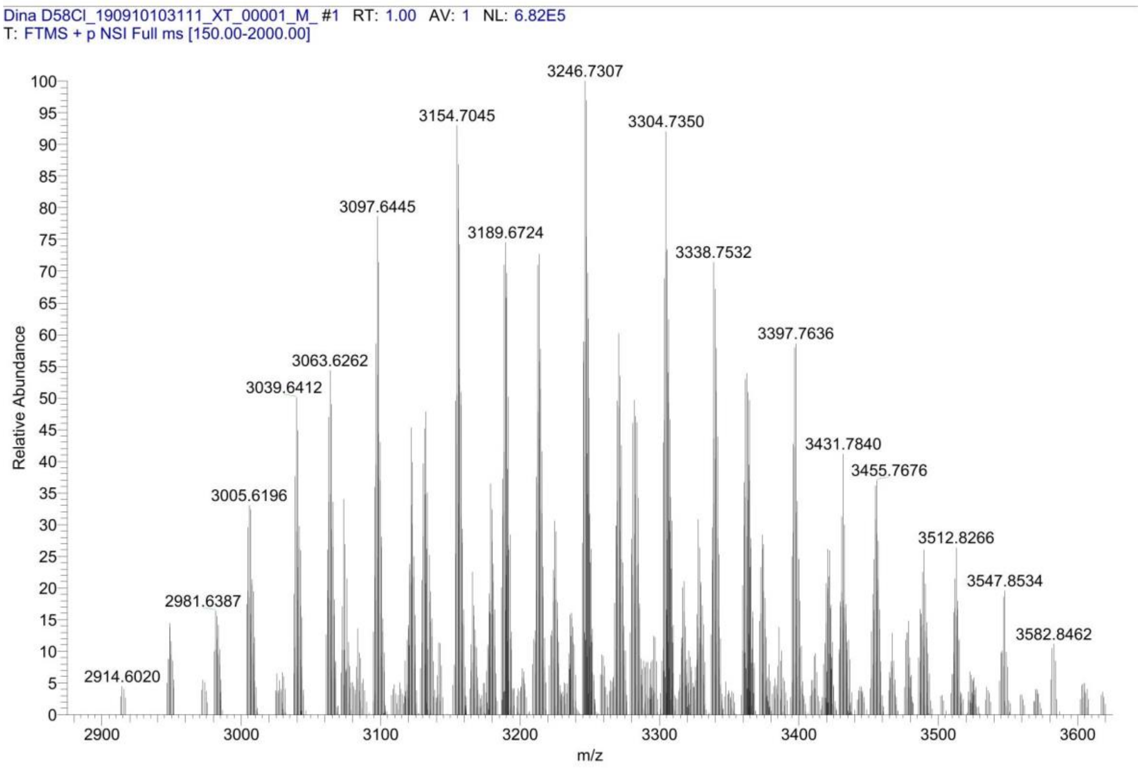

32 ((X)$_{40}$-NH$_2$) was synthetised using the CEM Liberty Blue synthetiser by mixing Fmoc-L-alanine, Fmoc-L-lysine, Fmoc-L-glutamic acid, Fmoc-L-tyrosine in ratio 4.2/3.4/1.4/1 respectively. Colourless foamy solid was obtained after preparative RP-HPLC (62.4 mg). Analytical RP-HPLC: t_R=2.72- min (100% A to 100% D in 10 min, λ= 214 nm).

Analytical RP-HPLC:

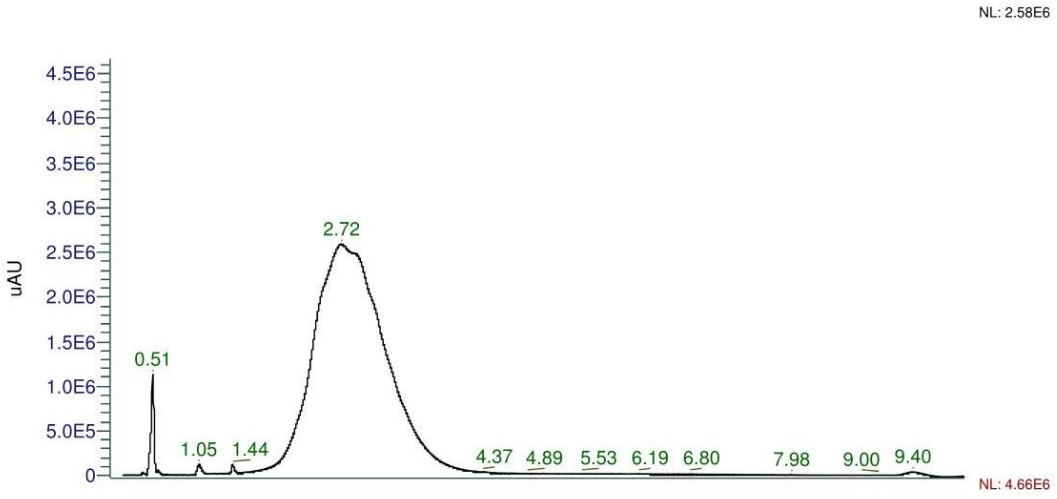

HRMS (NSI+):

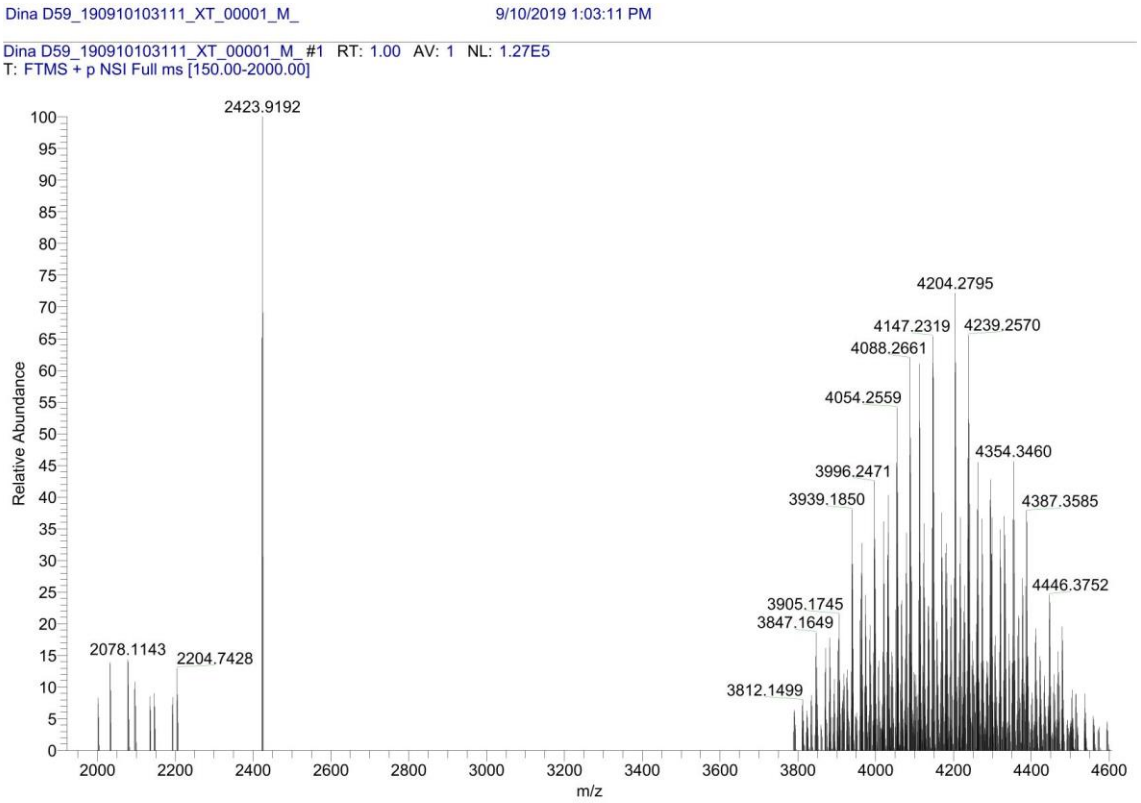

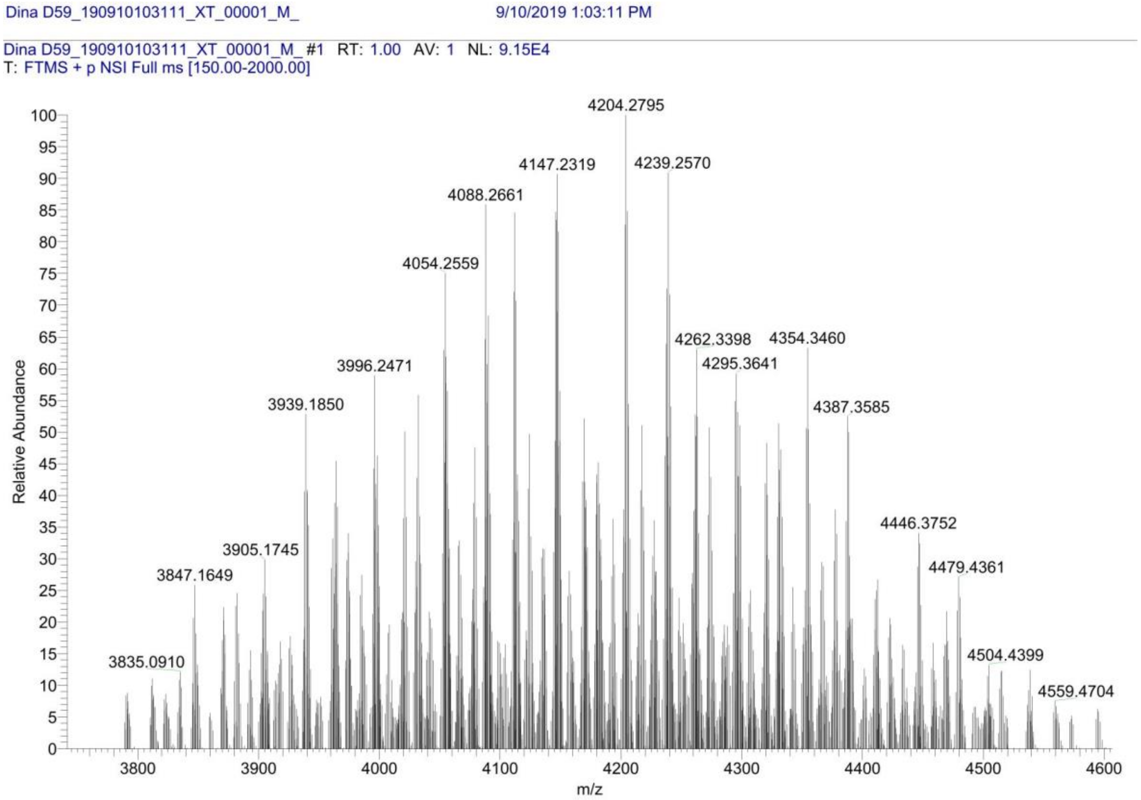
Dina D59_190910103111_XT_00001_M_
9/10/2019 1:03:11 PM
Dina D59_190910103111_XT_00001_M_ #1 RT: 1.00 AV: 1 NL: 9.15E4
T: FTMS + p NSI Full ms [150.00-2000.00]
4204.2795
4147.2319
4239.2570
4088.2661
4054.2559
4262.3398
4354.3460
3996.2471
4295.3641
3939.1850
4387.3585
4446.3752
3905.1745
4479.4361
3847.1649
4504.4399
3835.0910
4559.4704
Relative Abundance
m/z

Ac**32 (Ac(X)$_{40}$-NH$_2$)** was synthetised using the CEM Liberty Blue synthetiser by mixing Fmoc-L-alanine, Fmoc-L-lysine, Fmoc-L-glutamic acid, Fmoc-L-tyrosine in ratio 4.2/3.4/1.4/1 respectively. Colourless foamy solid was obtained after preparative RP-HPLC (51.3 mg). Analytical RP-HPLC: t_R=2.79- min (100% A to 100% D in 10 min, λ= 214 nm).

Analytical RP-HPLC:

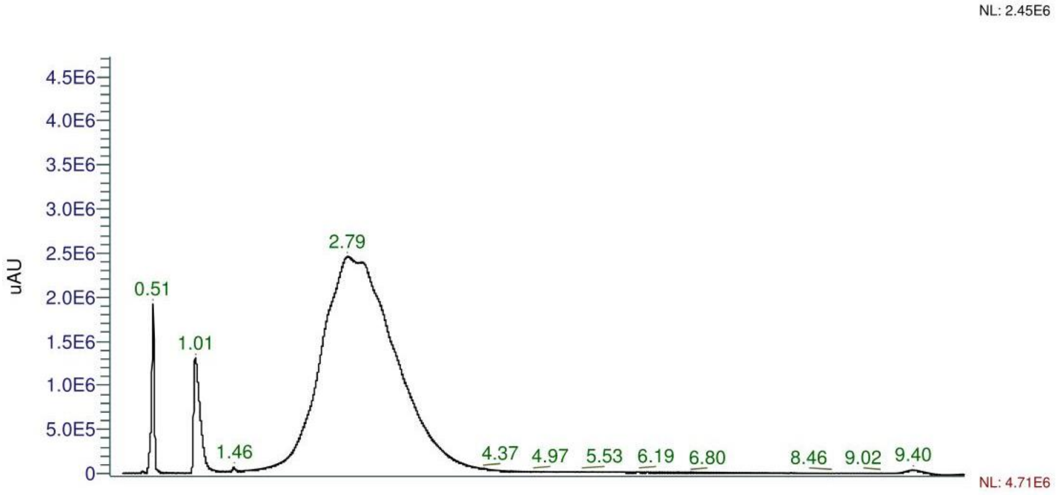

HRMS (NSI+):

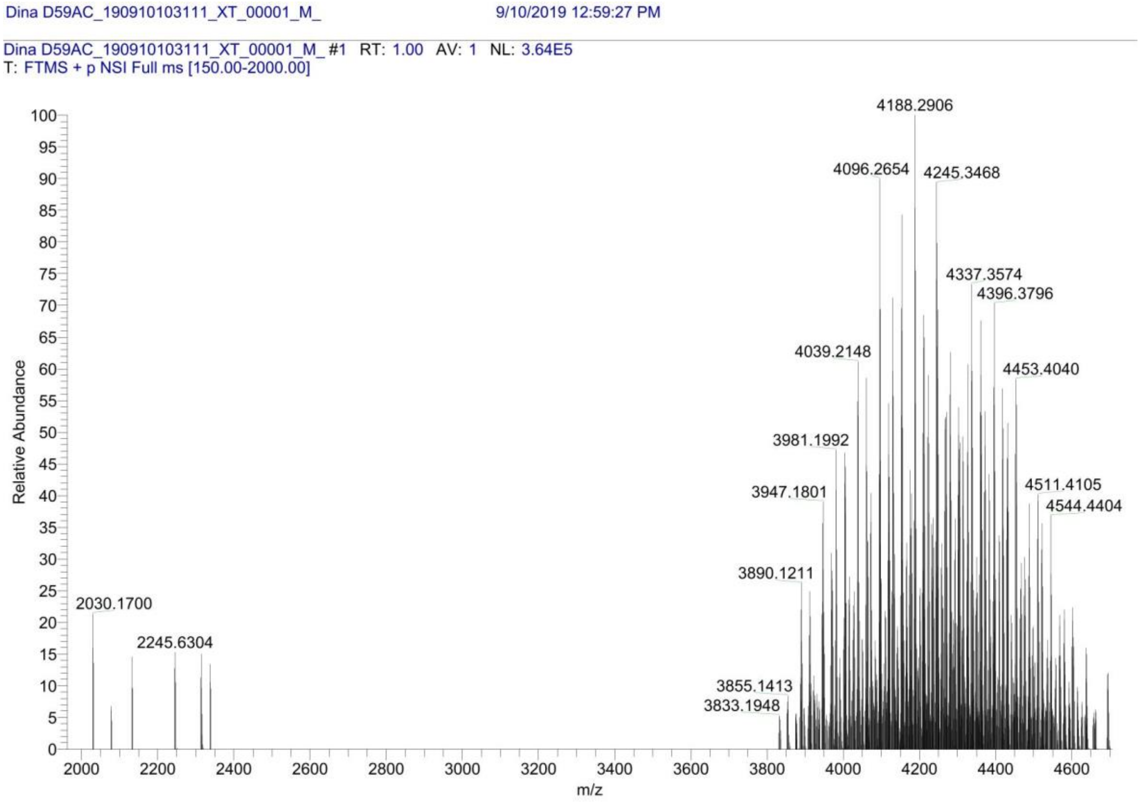

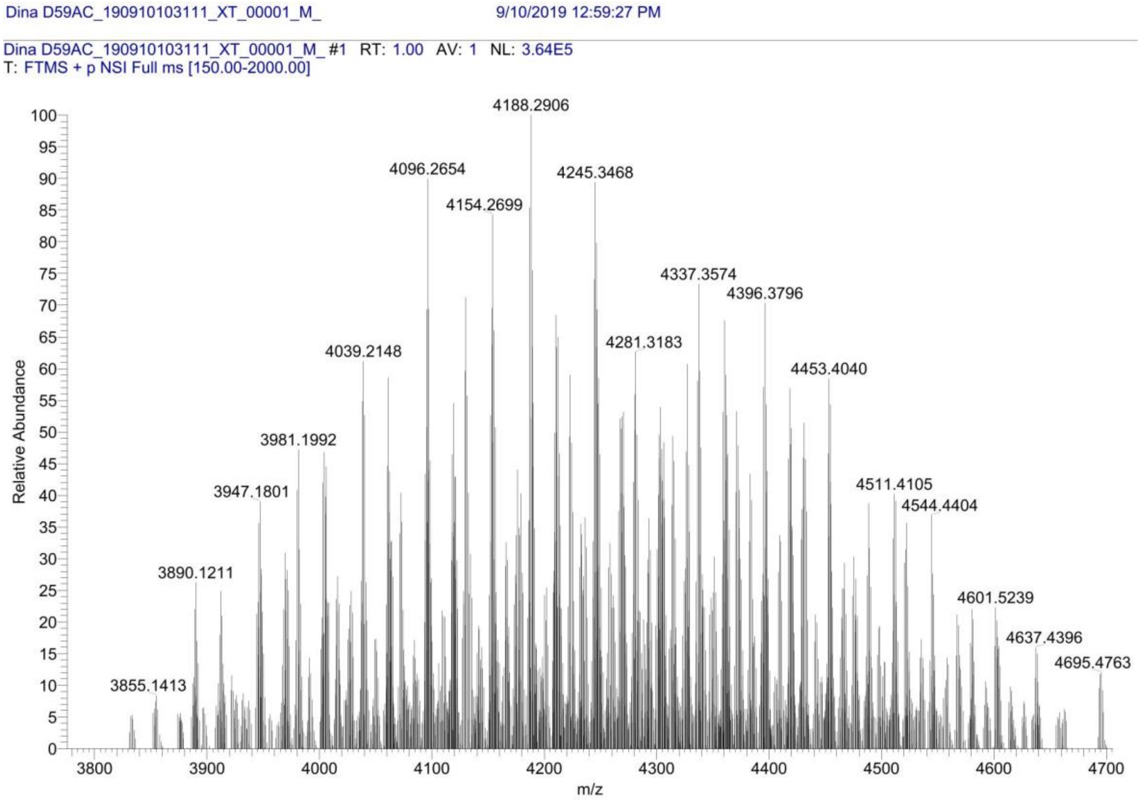
Dina D59AC_190910103111_XT_00001_M_
9/10/2019 12:59:27 PM
Dina D59AC_190910103111_XT_00001_M_ #1 RT: 1.00 AV: 1 NL: 3.64E5
T: FTMS + p NSI Full ms [150.00-2000.00]
Relative Abundance
m/z
4188.2906
4096.2654
4245.3468
4154.2699
4337.3574
4396.3796
4281.3183
4039.2148
4453.4040
3981.1992
3947.1801
4511.4105
4544.4404
3890.1211
4601.5239
4637.4396
4695.4763
3855.1413

33 ((*K*AK)$_4$(*K*EKA)$_2$*K*AKEAYCA-NH$_2$)) was obtained after manual synthesis as foamy colourless solid after preparative RP-HPLC (21.9 mg, 4.66 µmol, 14.1%). Analytical RP-HPLC: t_R=1.07 min (100% A to 100% D in 5 min, λ= 214 nm). HRMS (ESI+): $C_{138}H_{251}N_{43}O_{35}S$ calc./obs. 3102.8903/3102.8874 Da [M].

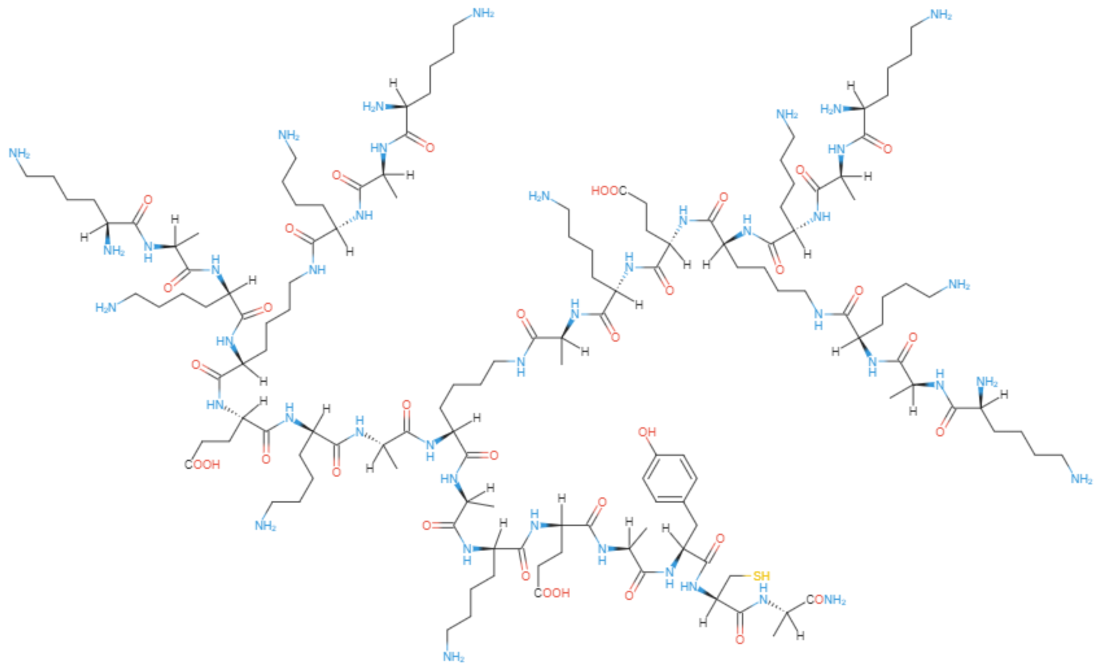

Analytical RP-HPLC:

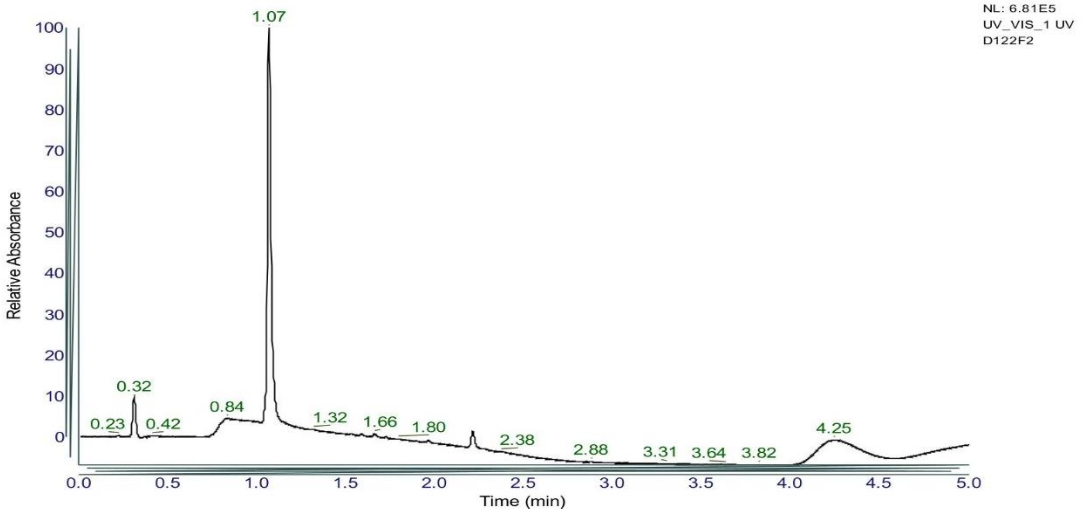

HRMS (NSI+):

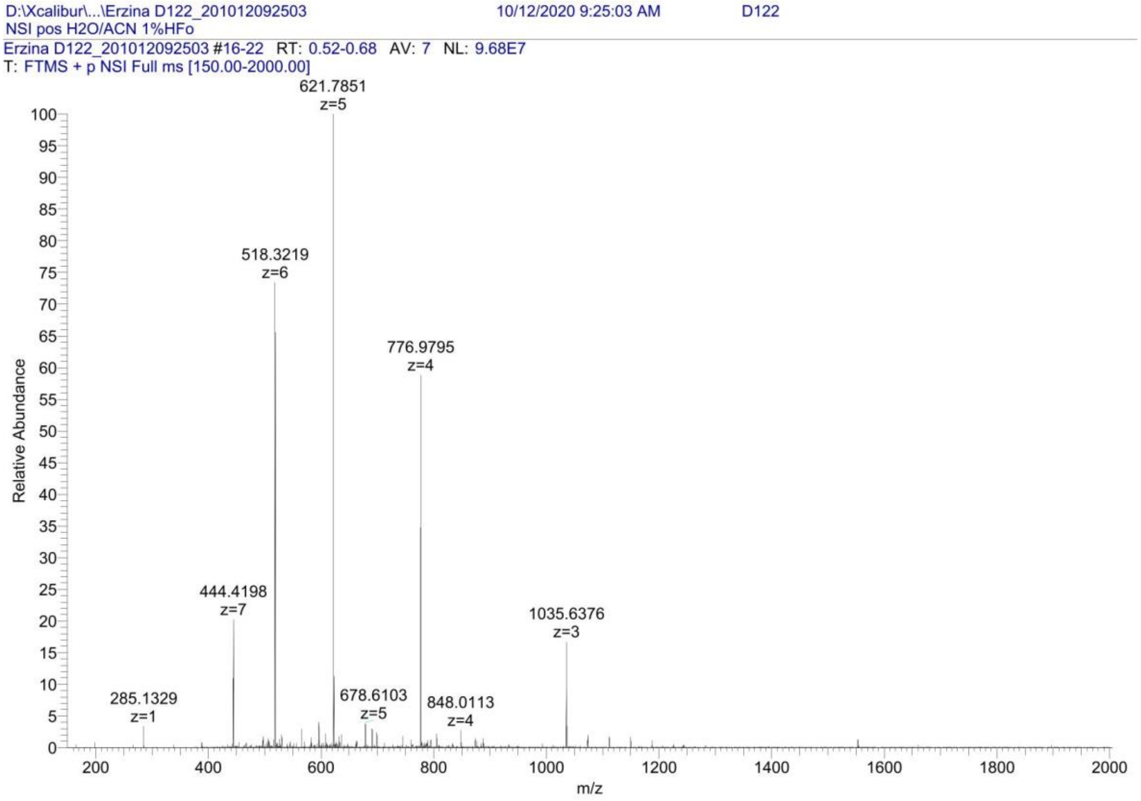
D:\Xcalibur\...\Erzina D122_201012092503
10/12/2020 9:25:03 AM
D122
NSI pos H2O/ACN 1%HFo
Erzina D122_201012092503 #16-22 RT: 0.52-0.68 AV: 7 NL: 9.68E7
T: FTMS + p NSI Full ms [150.00-2000.00]
621.7851 z=5
518.3219 z=6
776.9795 z=4
444.4198 z=7
1035.6376 z=3
285.1329 z=1
678.6103 z=5
848.0113 z=4
Relative Abundance
m/z

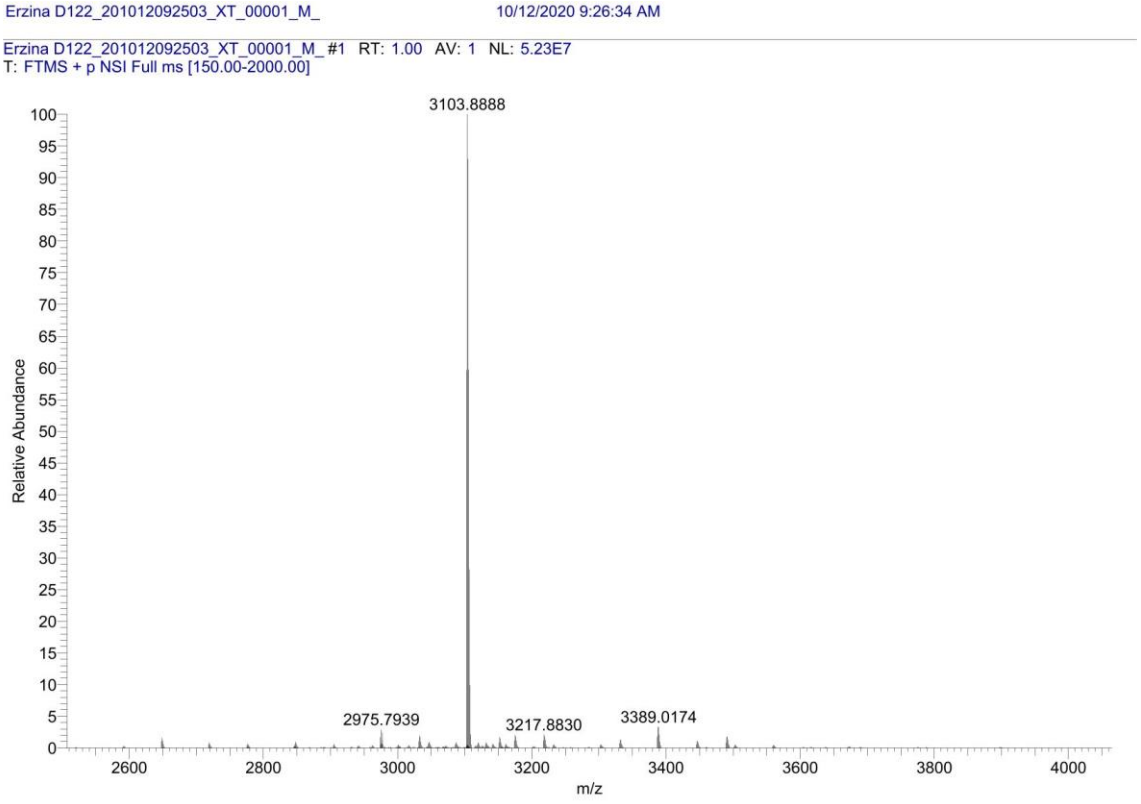
Erzina D122_201012092503_XT_00001_M_
10/12/2020 9:26:34 AM
Erzina D122_201012092503_XT_00001_M_ #1 RT: 1.00 AV: 1 NL: 5.23E7
T: FTMS + p NSI Full ms [150.00-2000.00]
3103.8888
2975.7939
3217.8830
3389.0174
Relative Abundance
m/z

Erzina D122_201012092503_XT_00001_M_ 10/12/2020 9:26:34 AM

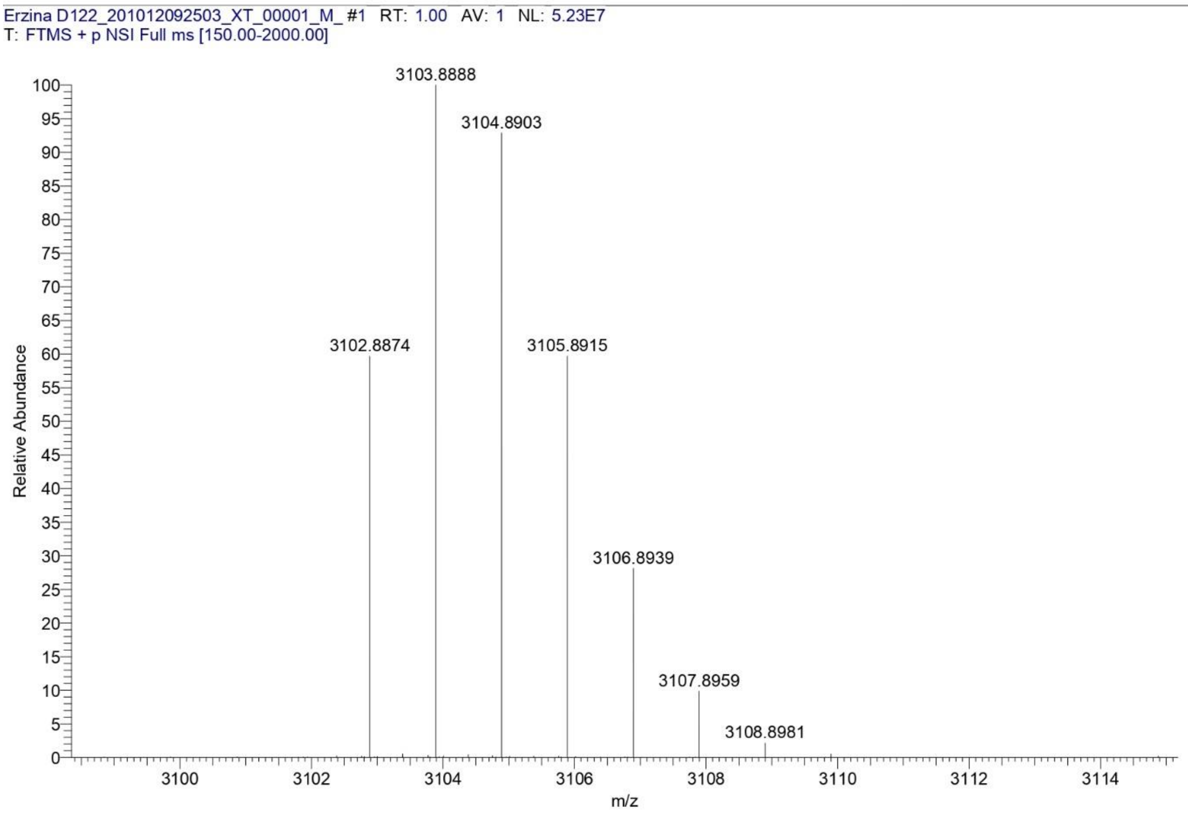

34 ((AK)$_8$(*K*AK)$_4$(*K*EKA)$_2$*K*AKEAYCA-NH$_2$)) was obtained after manual synthesis as foamy colourless solid after preparative RP-HPLC (23.2 mg, 3.2 µmol, 23.2%). Analytical RP-HPLC: t_R=1.06 min (100% A to 100% D in 5 min, λ= 214 nm). HRMS (ESI+): $C_{210}H_{387}N_{67}O_{51}S$ calc./obs. 4695.9470/4695.9469 Da [M].

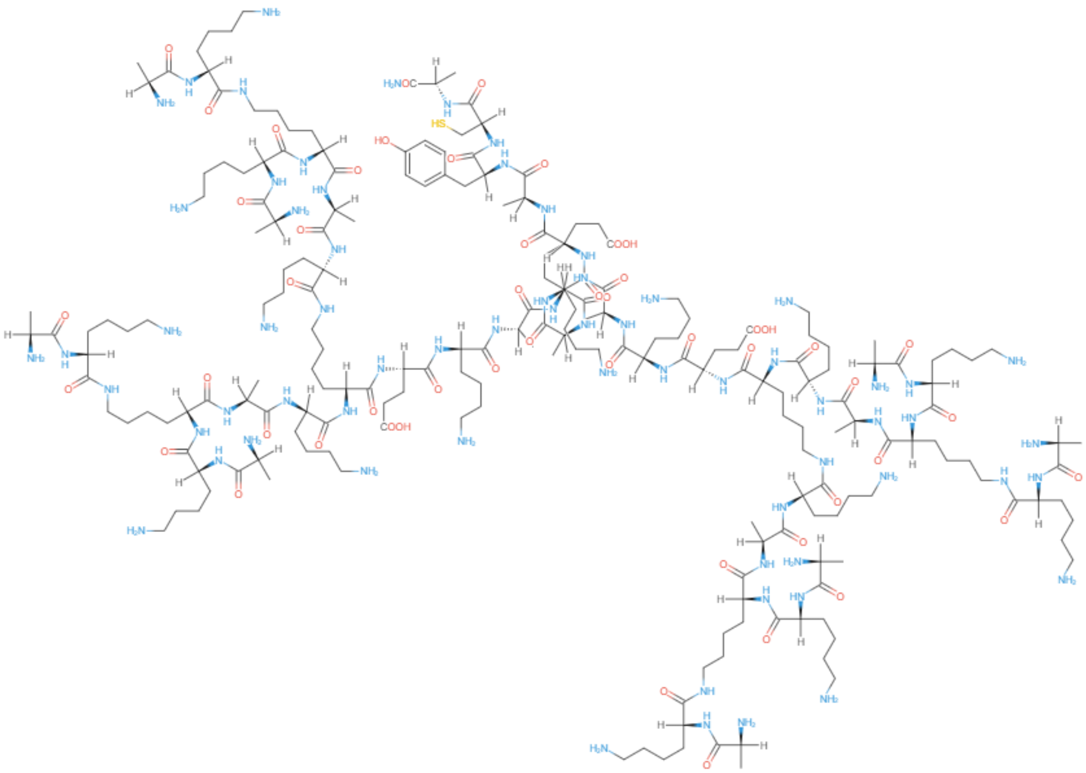

Analytical RP-HPLC:

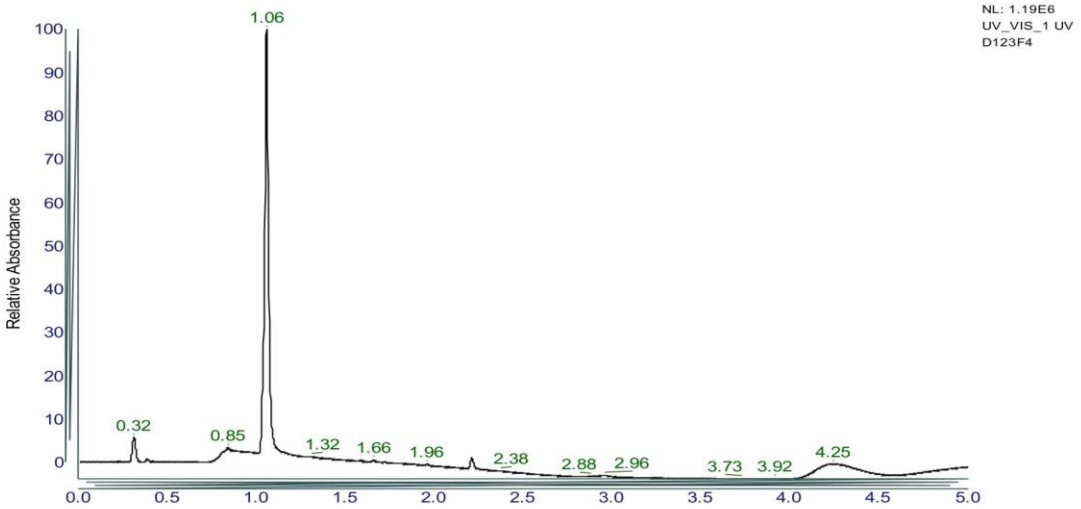

HRMS (NSI+):

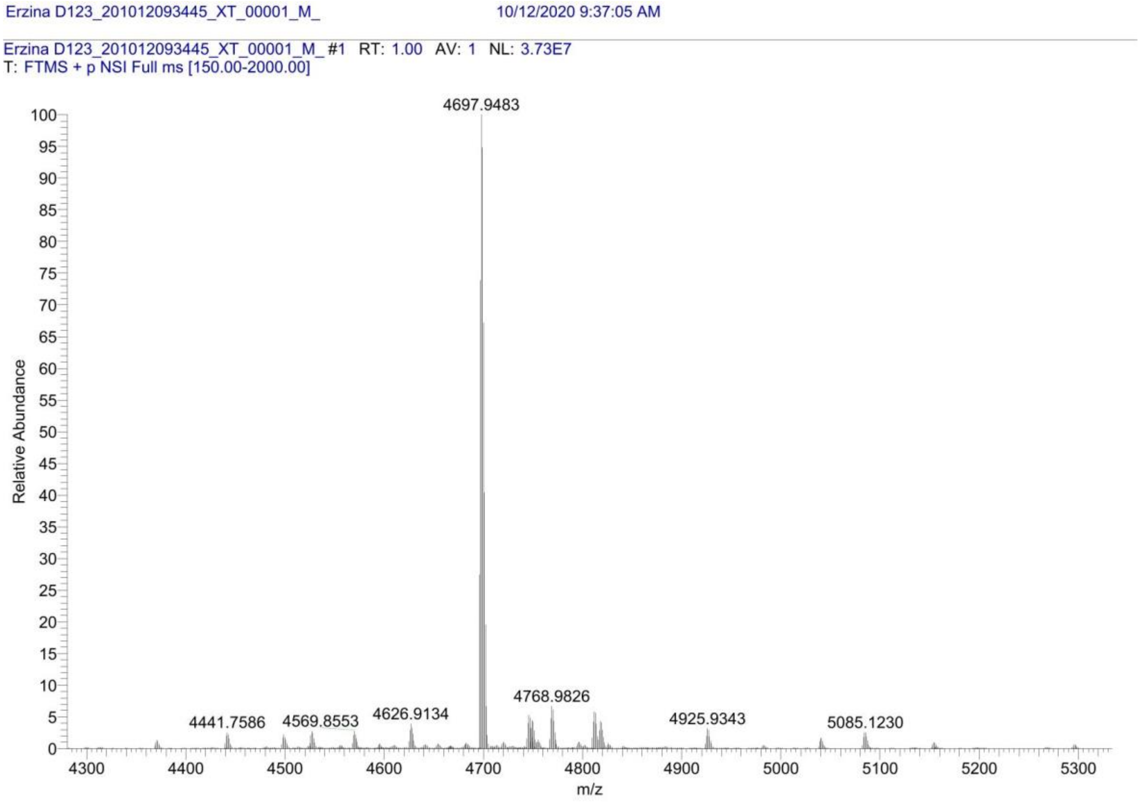
Erzina D123_201012093445_XT_00001_M_
10/12/2020 9:37:05 AM
Erzina D123_201012093445_XT_00001_M_ #1 RT: 1.00 AV: 1 NL: 3.73E7
T: FTMS + p NSI Full ms [150.00-2000.00]
4697.9483
4441.7586
4569.8553
4626.9134
4768.9826
4925.9343
5085.1230
Relative Abundance
m/z

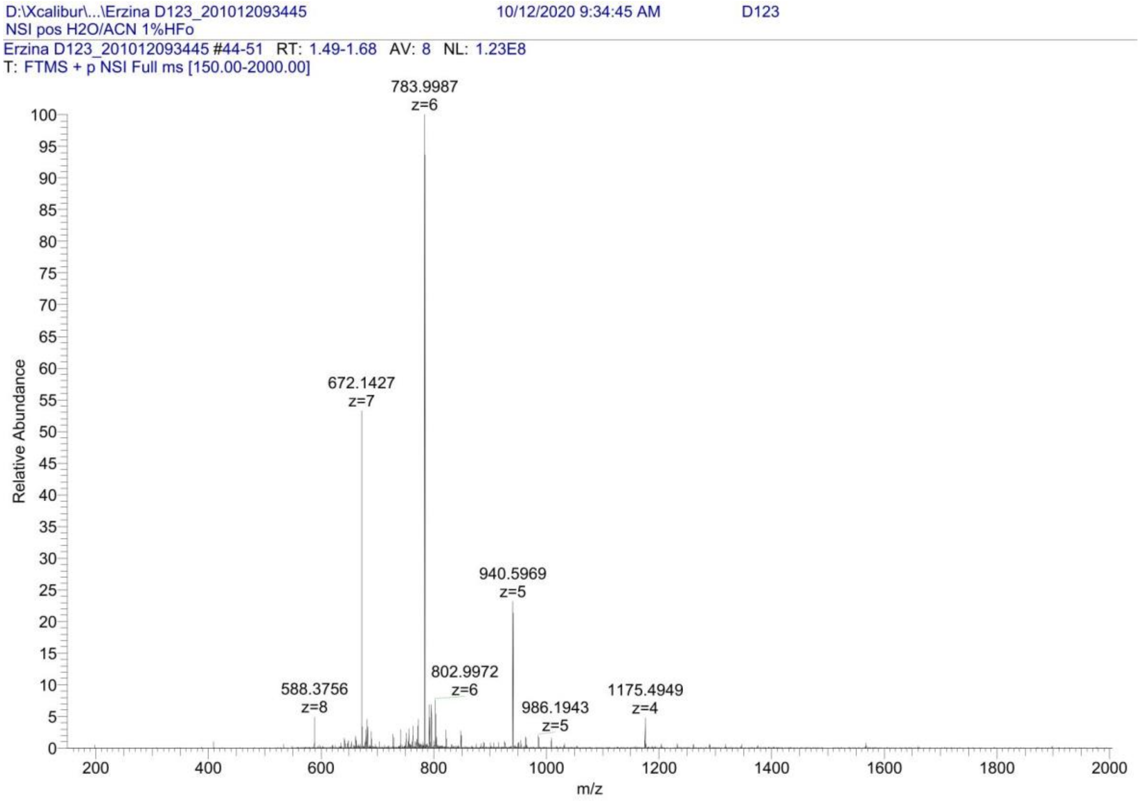
D:\Xcalibur\...\Erzina D123_201012093445
10/12/2020 9:34:45 AM
D123
NSI pos H2O/ACN 1%HFo
Erzina D123_201012093445 #44-51 RT: 1.49-1.68 AV: 8 NL: 1.23E8
T: FTMS + p NSI Full ms [150.00-2000.00]
783.9987
z=6
672.1427
z=7
940.5969
z=5
802.9972
z=6
588.3756
z=8
986.1943
z=5
1175.4949
z=4
Relative Abundance
m/z

Erzina D123_201012093445_XT_00001_M_ 10/12/2020 9:37:05 AM

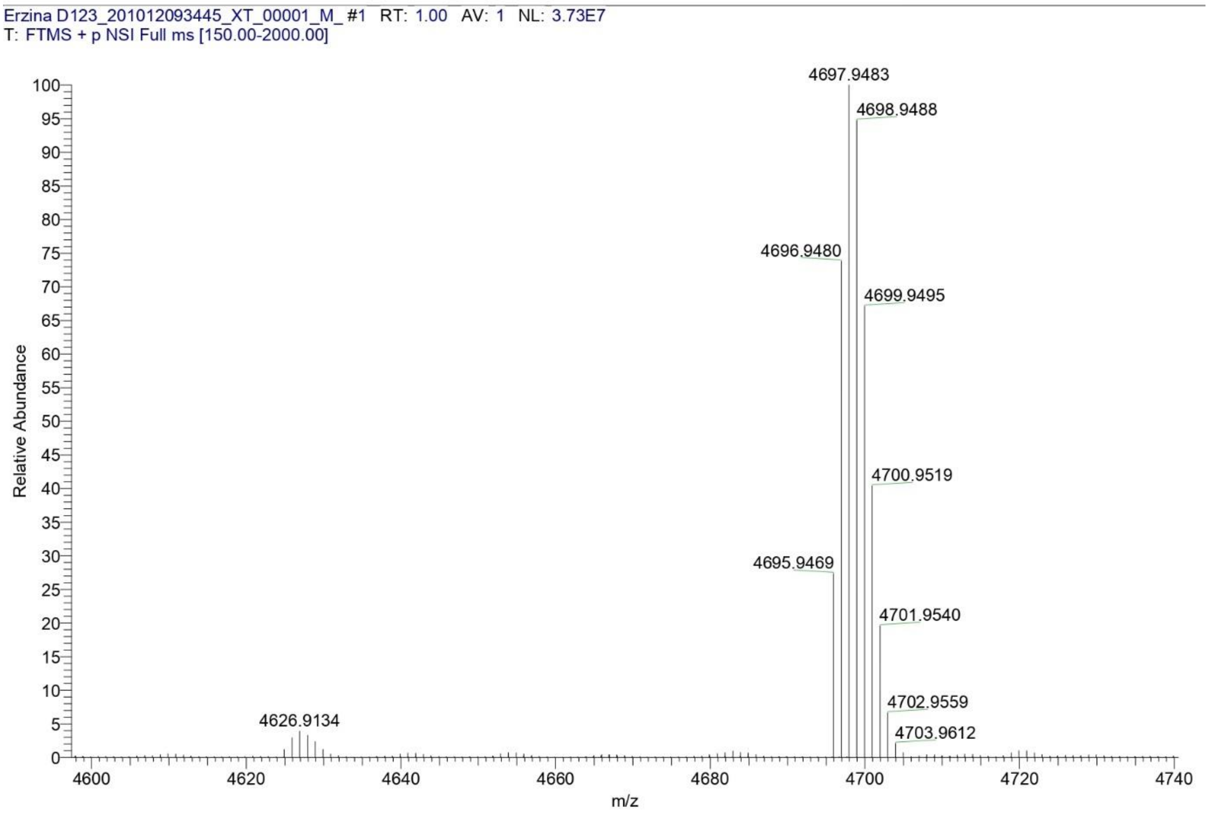

35 ((KK)$_8$(*K*AK)$_4$(*K*EKA)$_2$*K*AKEAYCA-NH$_2$) was obtained after manual synthesis as foamy colourless solid after preparative RP-HPLC (18.9 mg, 2.2 µmol, 18.9%). Analytical RP-HPLC: t_R=1.07 min (100% A to 100% D in 5 min, λ= 214 nm). HRMS (ESI+): $C_{234}H_{443}N_{75}O_{51}S$ calc./obs. 5152.4098/5152.4134 Da [M].

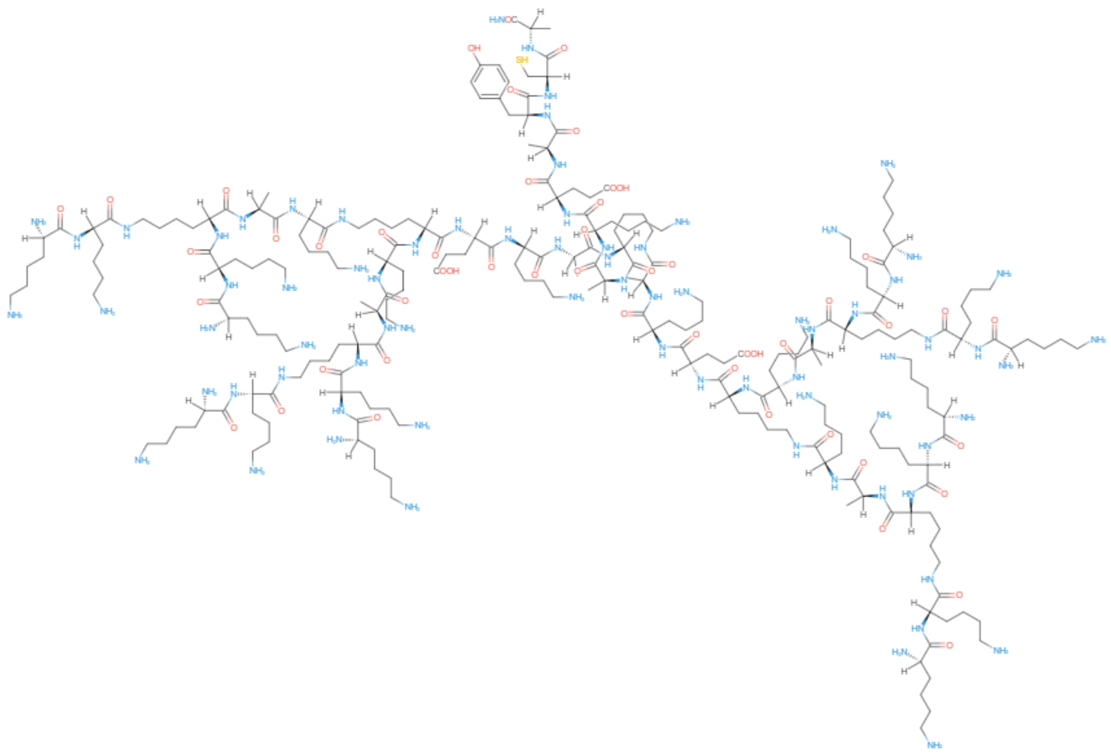

Analytical RP-HPLC:

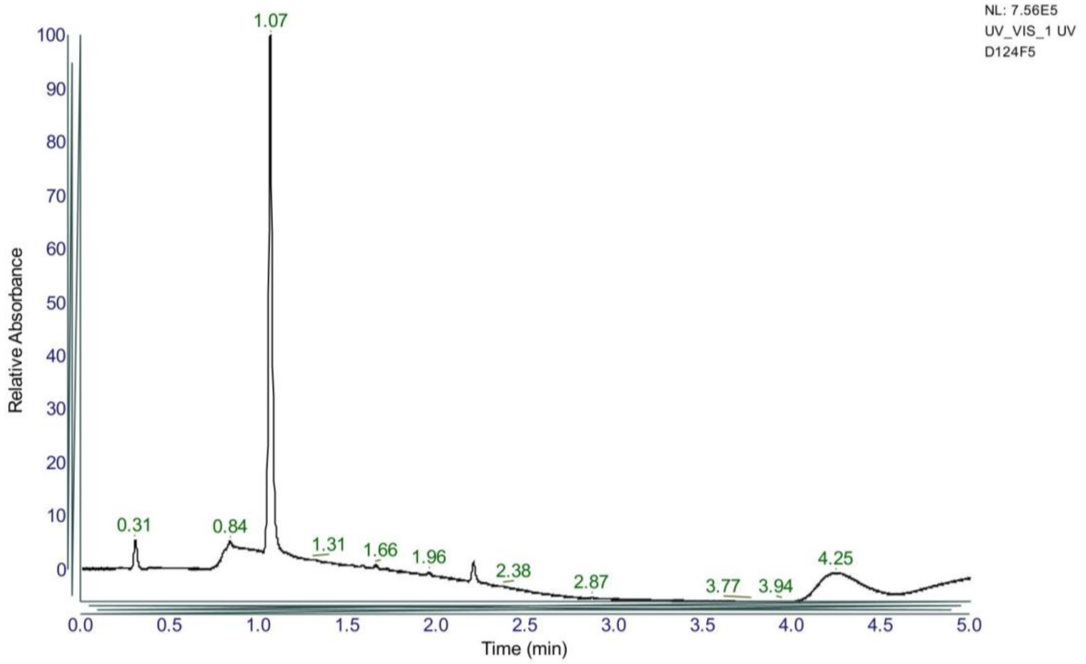

HRMS (NSI+):

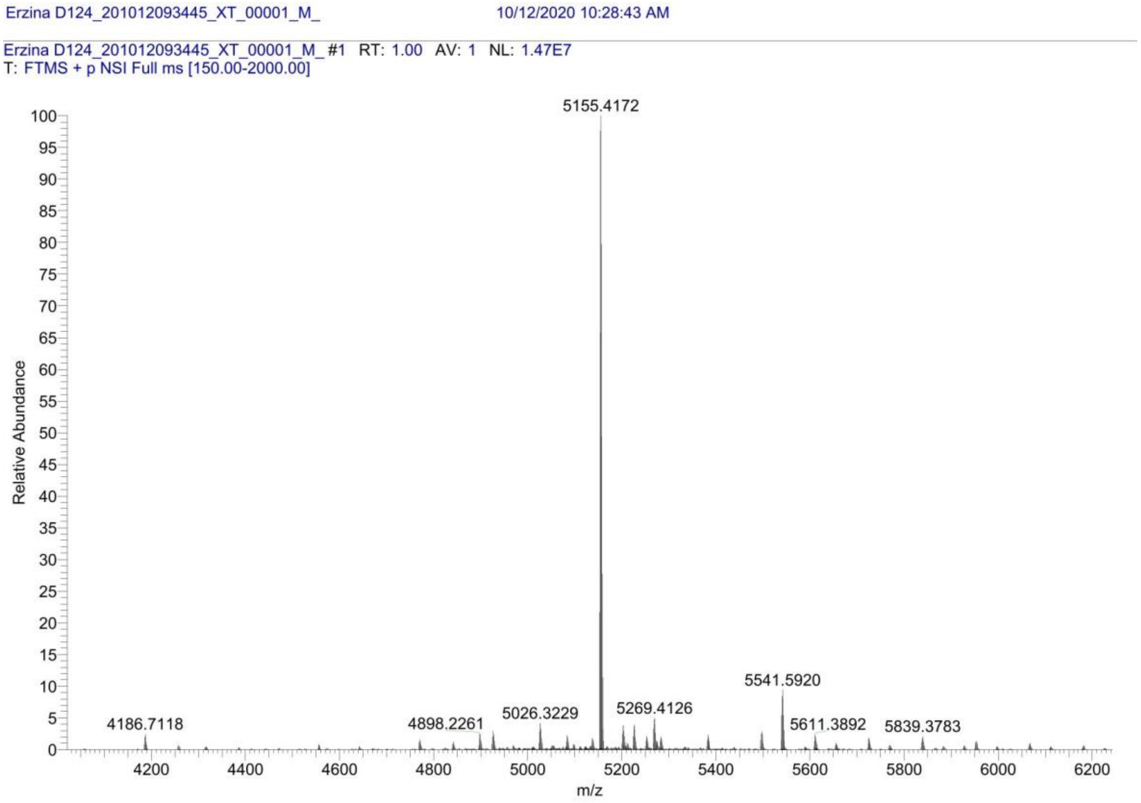
Erzina D124_201012093445_XT_00001_M_
10/12/2020 10:28:43 AM
Erzina D124_201012093445_XT_00001_M_ #1 RT: 1.00 AV: 1 NL: 1.47E7
T: FTMS + p NSI Full ms [150.00-2000.00]
5155.4172
5541.5920
4186.7118
4898.2261
5026.3229
5269.4126
5611.3892
5839.3783
Relative Abundance
m/z

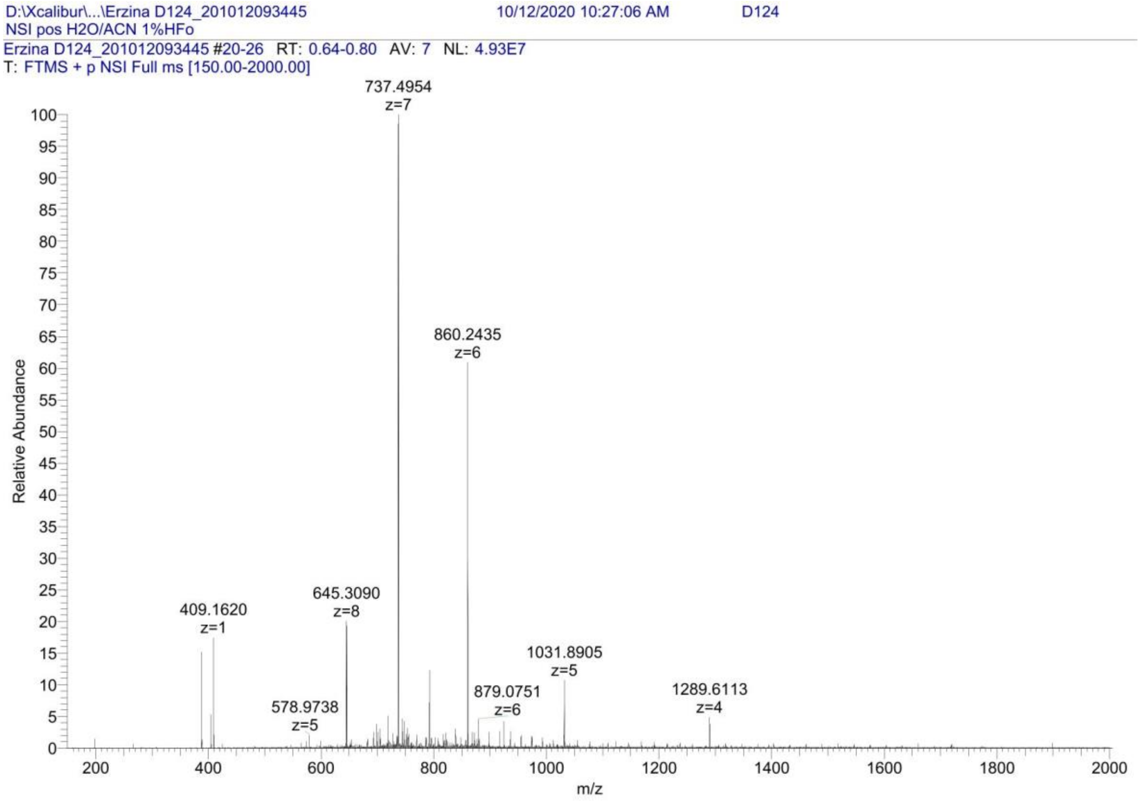
D:\Xcalibur\...\Erzina D124_201012093445
10/12/2020 10:27:06 AM
D124
NSI pos H2O/ACN 1%HFo
Erzina D124_201012093445 #20-26 RT: 0.64-0.80 AV: 7 NL: 4.93E7
T: FTMS + p NSI Full ms [150.00-2000.00]
737.4954 z=7
860.2435 z=6
409.1620 z=1
645.3090 z=8
1031.8905 z=5
879.0751 z=6
1289.6113 z=4
578.9738 z=5
Relative Abundance
m/z

Erzina D124_201012093445_XT_00001_M_ 10/12/2020 10:28:43 AM

Erzina D124_201012093445_XT_00001_M_ #1 RT: 1.00 AV: 1 NL: 1.47E7
T: FTMS + p NSI Full ms [150.00-2000.00]

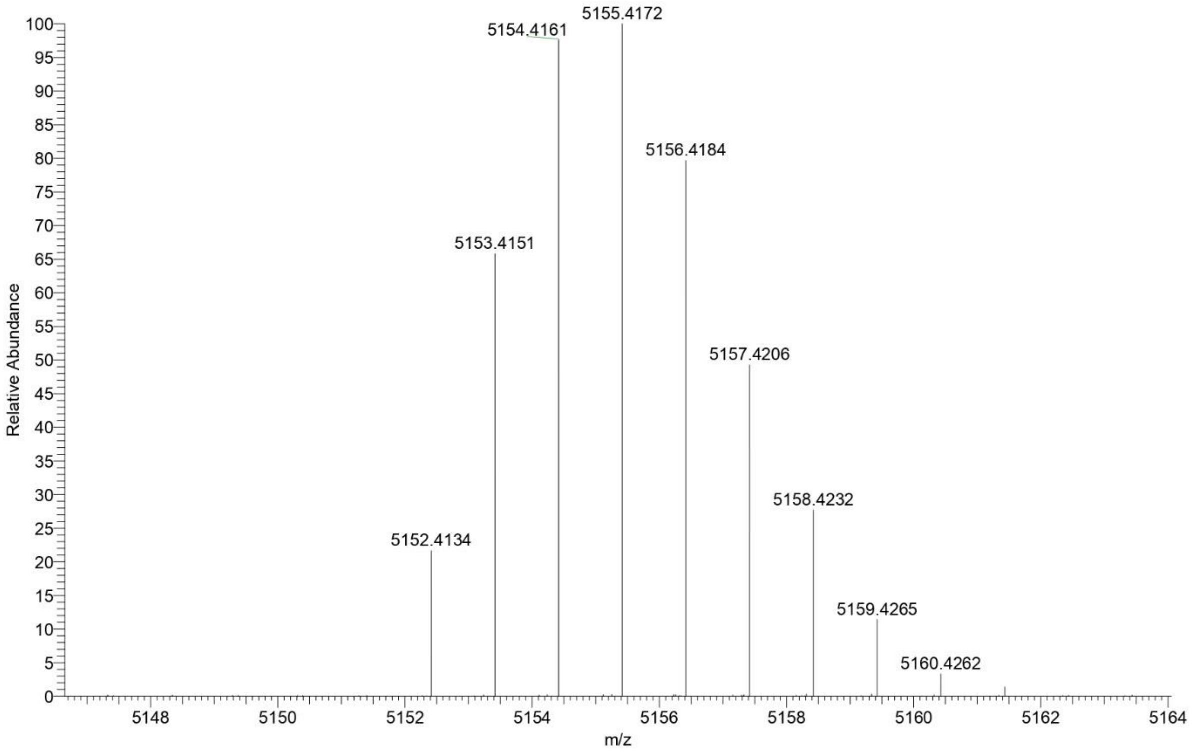

36 ((KA)$_8$(*K*KKAK)$_4$(*K*EKA)$_2$*K*AKEAYCA-NH$_2$) was obtained from the CEM Liberty Blue synthetiser as foamy colourless solid after preparative RP-HPLC (28.0 mg, 0.9 µmol, 8.6%). Analytical RP-HPLC: t_R=1.18 min (100% A to 100% D in 5 min, λ= 214 nm). HRMS (ESI+): $C_{258}H_{483}N_{83}O_{59}S$ calc./obs. 5720.7067/5720.7093 Da [M].

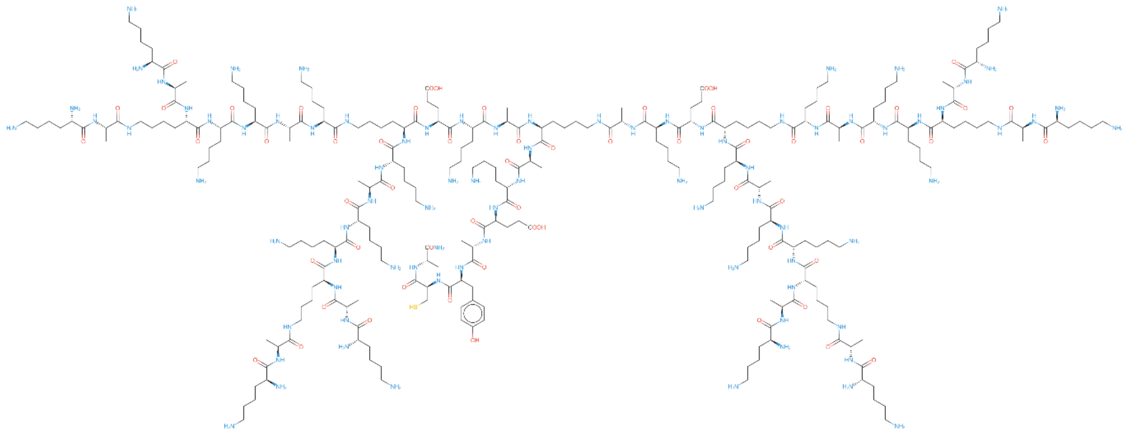

Analytical RP-HPLC:

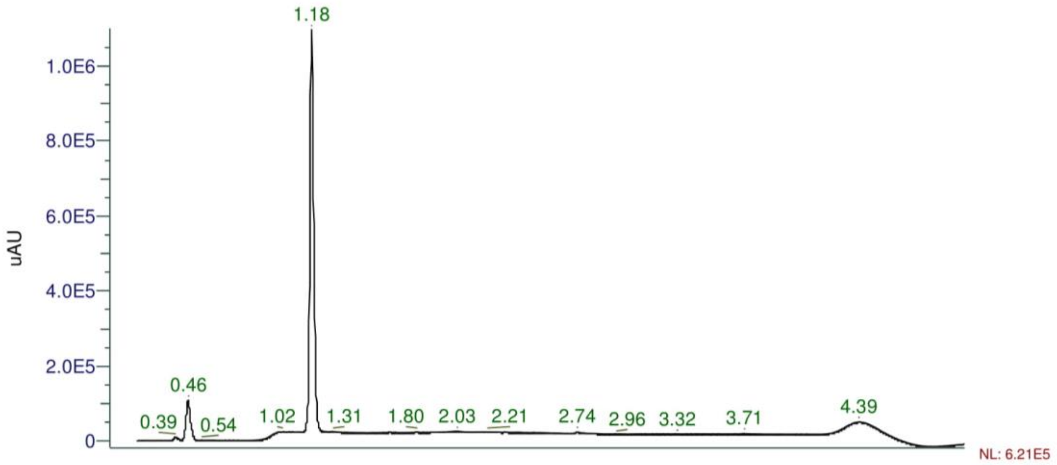

HRMS (NSI+):

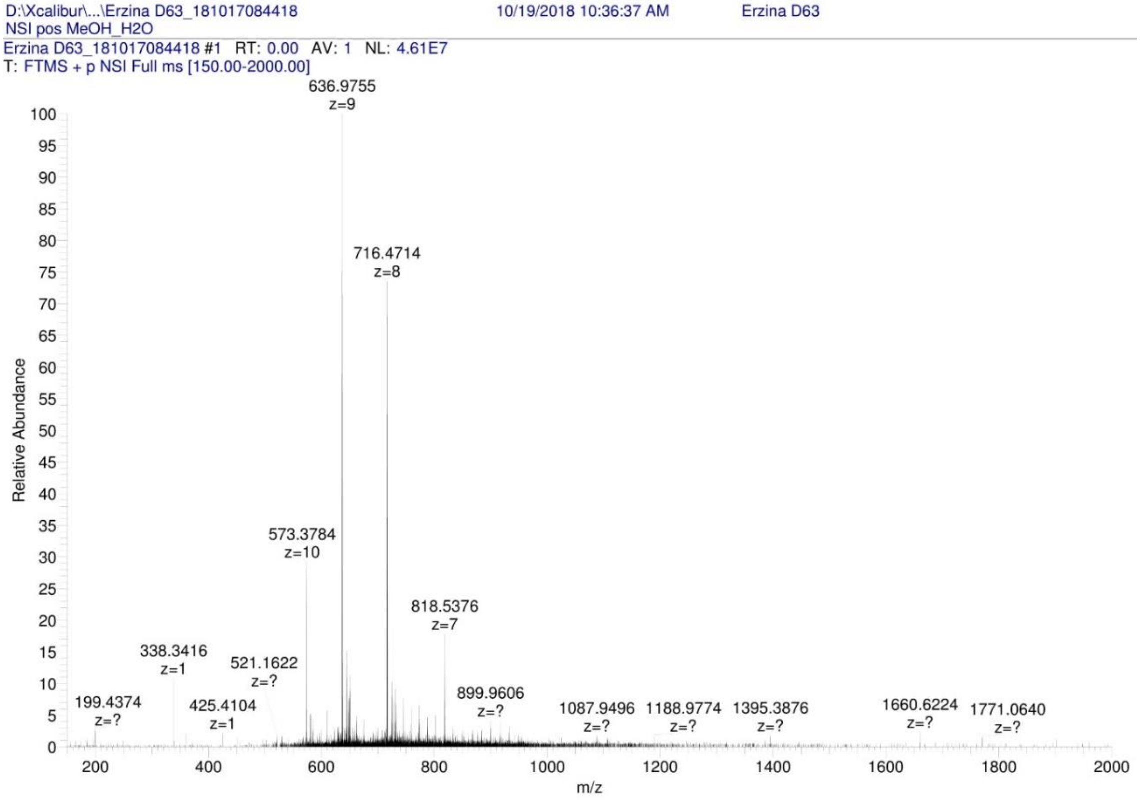
D:\Xcalibur\...\Erzina D63_181017084418
10/19/2018 10:36:37 AM
Erzina D63
NSI pos MeOH_H2O
Erzina D63_181017084418 #1 RT: 0.00 AV: 1 NL: 4.61E7
T: FTMS + p NSI Full ms [150.00-2000.00]
636.9755
z=9
716.4714
z=8
573.3784
z=10
818.5376
z=7
338.3416
z=1
521.1622
z=?
899.9606
z=?
199.4374
z=?
425.4104
z=1
1087.9496
z=?
1188.9774
z=?
1395.3876
z=?
1660.6224
z=?
1771.0640
z=?
Relative Abundance
m/z

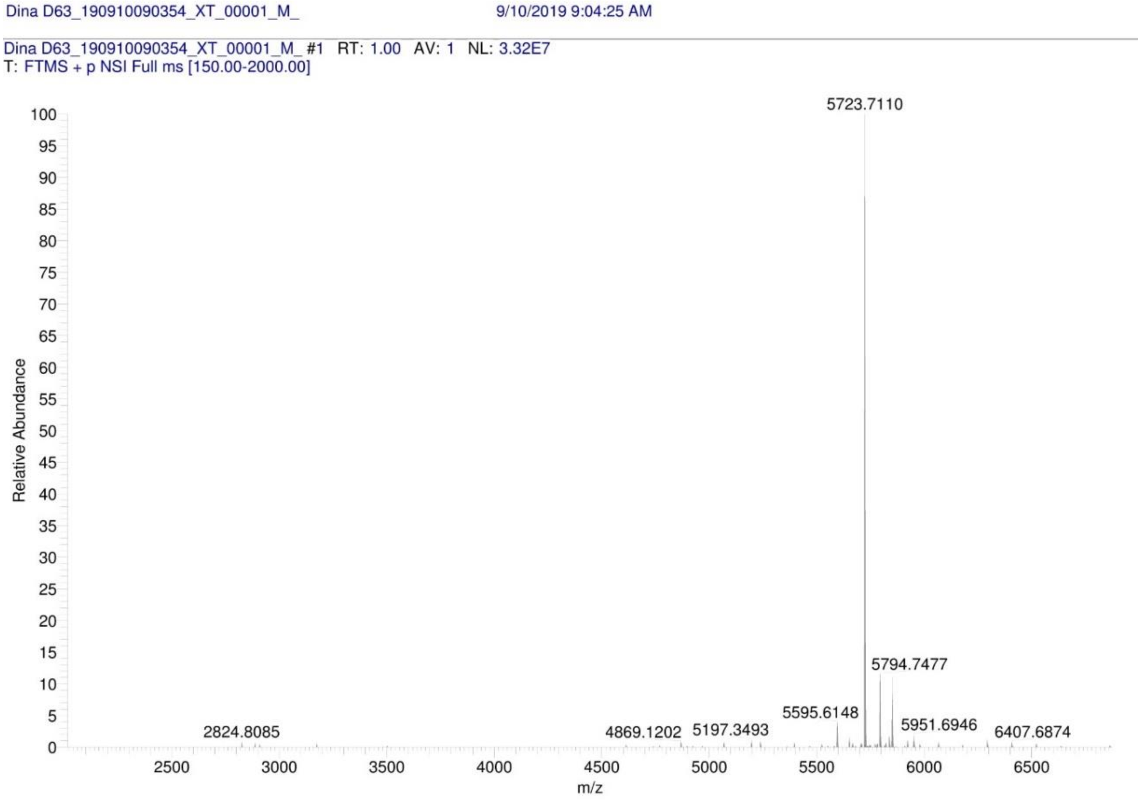
Dina D63_190910090354_XT_00001_M_
9/10/2019 9:04:25 AM
Dina D63_190910090354_XT_00001_M_ #1 RT: 1.00 AV: 1 NL: 3.32E7
T: FTMS + p NSI Full ms [150.00-2000.00]
5723.7110
5794.7477
5595.6148
5951.6946
2824.8085
4869.1202
5197.3493
6407.6874
Relative Abundance
m/z

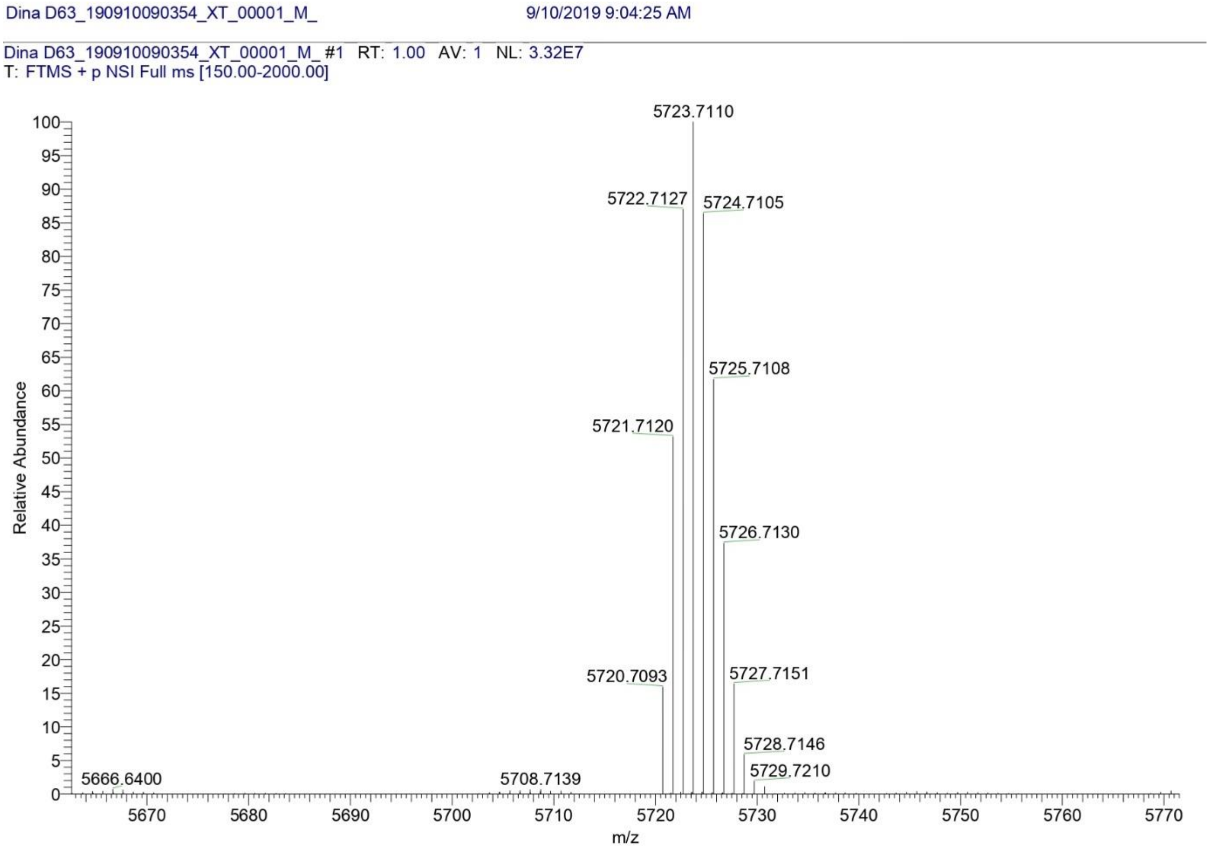
Dina D63_190910090354_XT_00001_M_
9/10/2019 9:04:25 AM
Dina D63_190910090354_XT_00001_M_ #1 RT: 1.00 AV: 1 NL: 3.32E7
T: FTMS + p NSI Full ms [150.00-2000.00]
5723.7110
5722.7127
5724.7105
5725.7108
5721.7120
5726.7130
5720.7093
5727.7151
5728.7146
5729.7210
5666.6400
5708.7139
Relative Abundance
m/z

Ac**36 ((AcKA)$_8$(*K*KKAK)$_4$(*K*EKA)$_2$*K*AKEAYCA-NH$_2$)** was obtained from the CEM Liberty Blue synthetiser as foamy colourless solid after preparative RP-HPLC (28.6 mg, 1.1 µmol, 9.4%). Analytical RP-HPLC: t_R=1.23 min (100% A to 100% D in 5 min, λ= 214 nm). HRMS (ESI+): $C_{274}H_{499}N_{83}O_{67}S$ calc./obs. 6056.7912/6056.7988 Da [M].

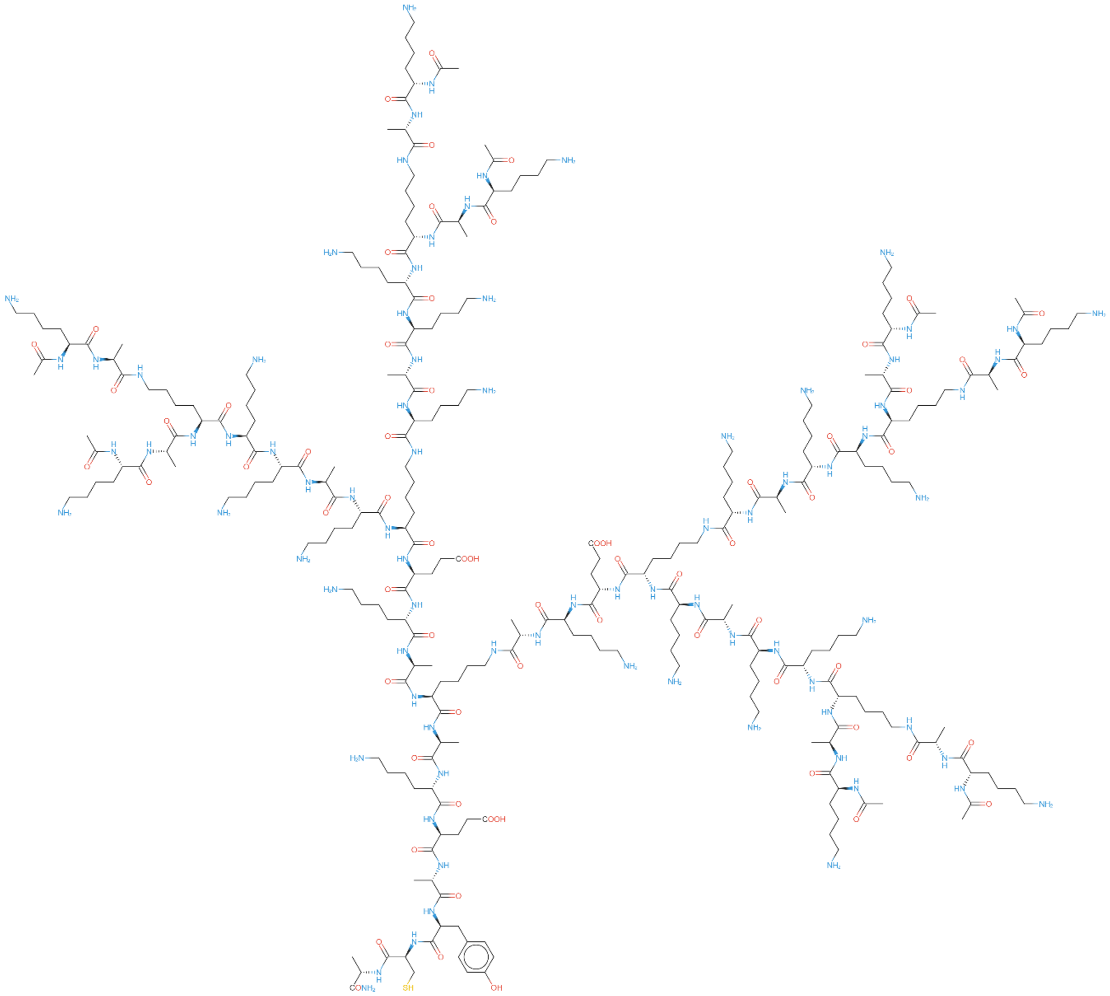

Analytical RP-HPLC:

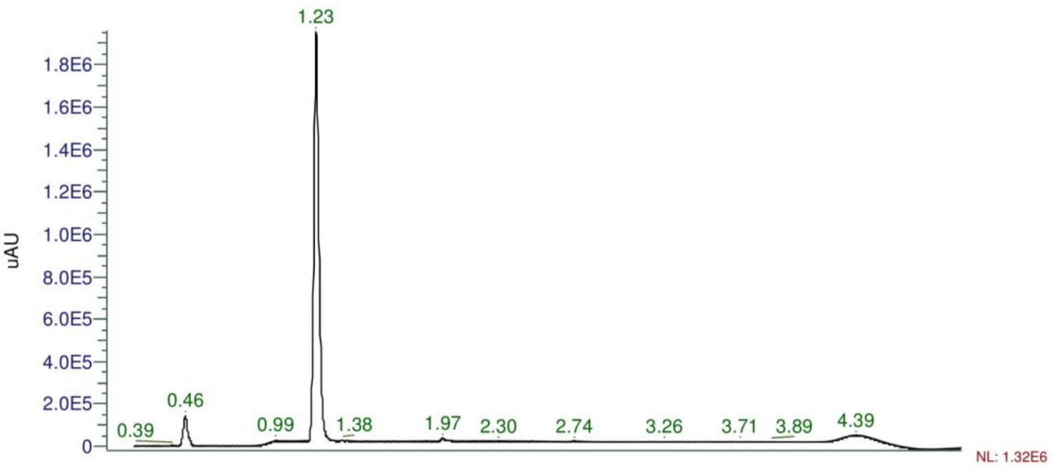

HRMS (NSI+):

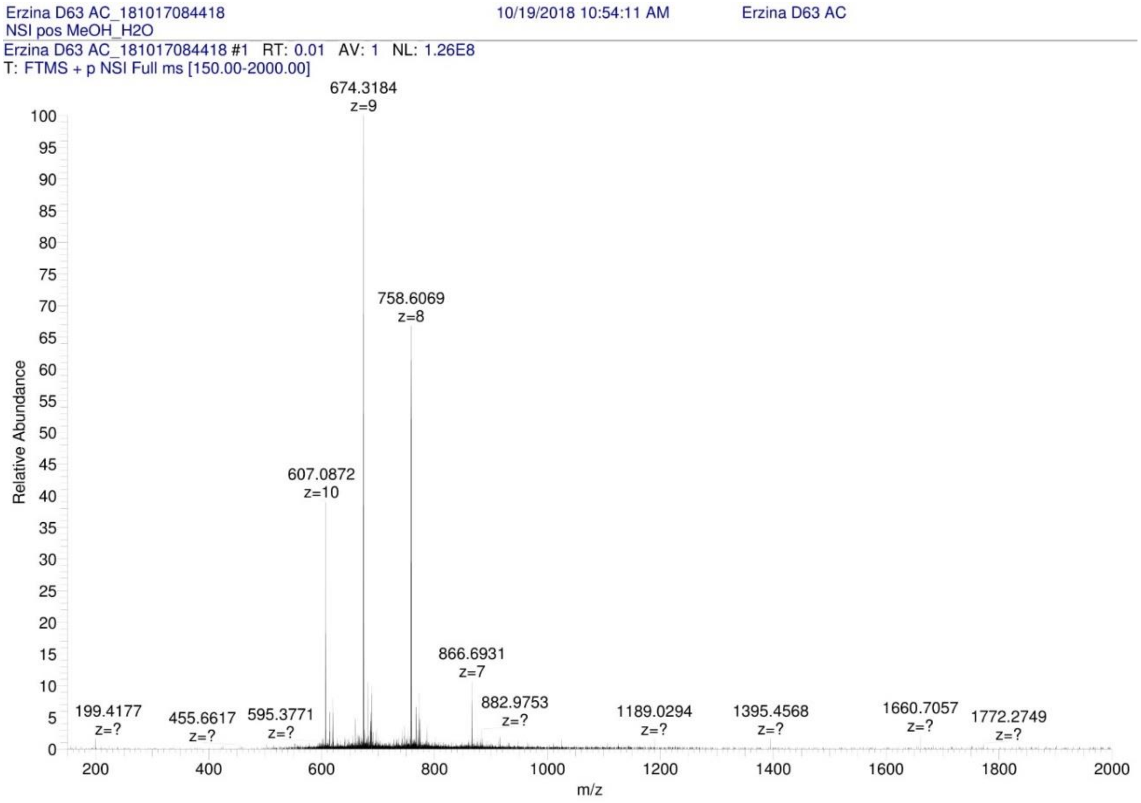
Erzina D63 AC_181017084418
10/19/2018 10:54:11 AM
Erzina D63 AC
NSI pos MeOH_H2O
Erzina D63 AC_181017084418 #1 RT: 0.01 AV: 1 NL: 1.26E8
T: FTMS + p NSI Full ms [150.00-2000.00]
674.3184 z=9
758.6069 z=8
607.0872 z=10
866.6931 z=7
882.9753 z=?
199.4177 z=?
455.6617 z=?
595.3771 z=?
1189.0294 z=?
1395.4568 z=?
1660.7057 z=?
1772.2749 z=?
Relative Abundance
m/z

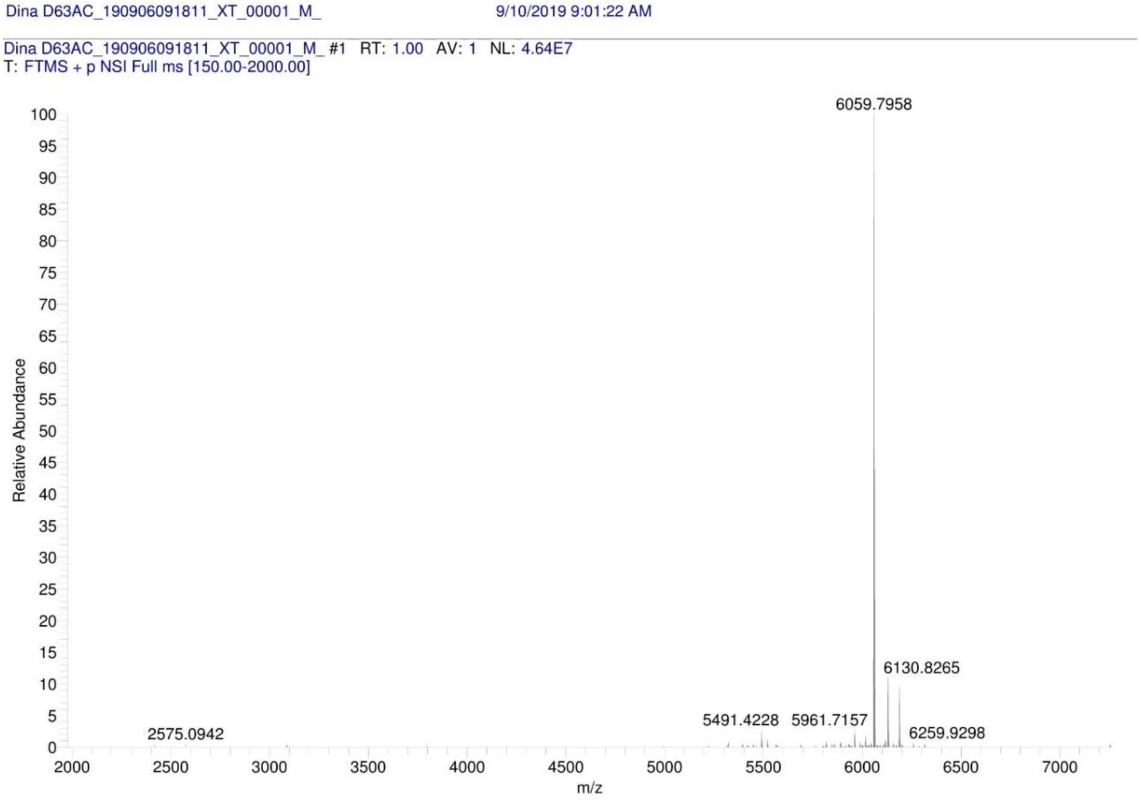
Dina D63AC_190906091811_XT_00001_M_
9/10/2019 9:01:22 AM
Dina D63AC_190906091811_XT_00001_M_ #1 RT: 1.00 AV: 1 NL: 4.64E7
T: FTMS + p NSI Full ms [150.00-2000.00]
6059.7958
6130.8265
5491.4228
5961.7157
6259.9298
2575.0942
Relative Abundance
m/z

Erzina D63 AC_181017084418_XT_00001_M_ 10/19/2018 10:54:33 AM

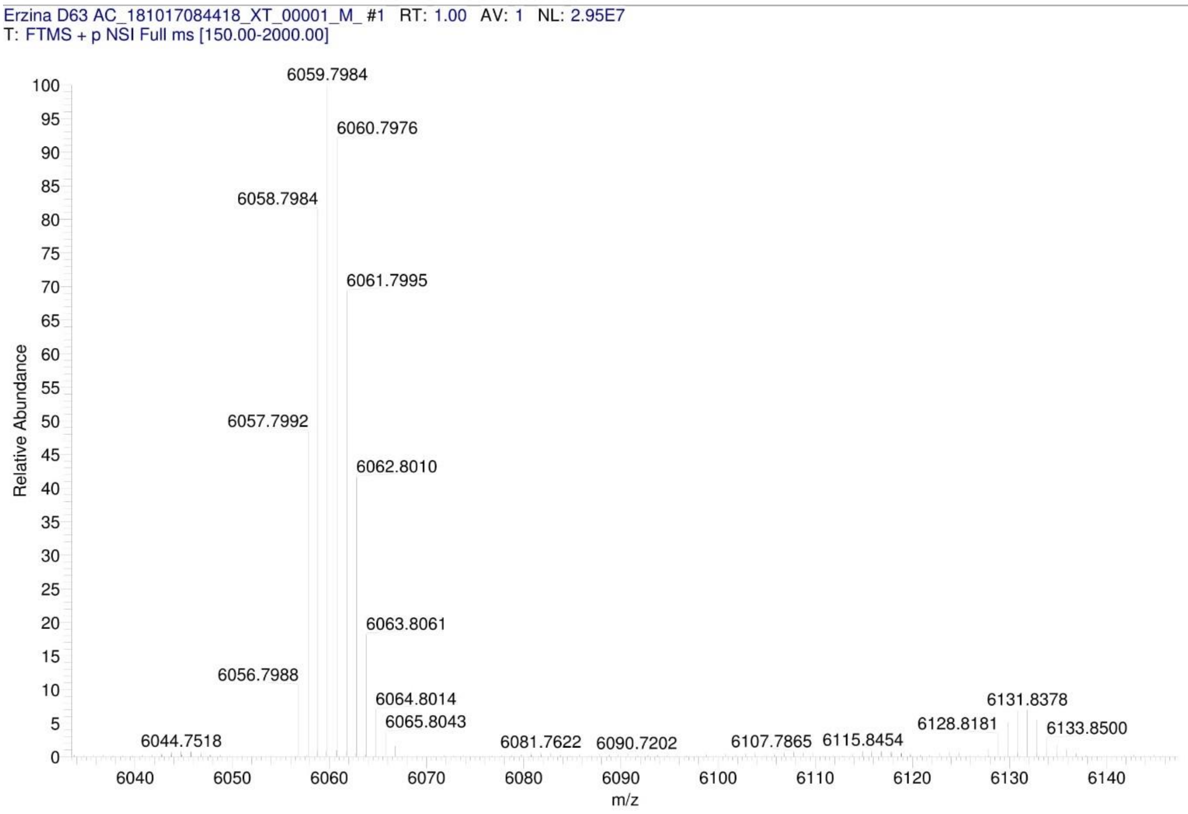

Fum**36 ((FumKA)$_8$(*K*KKAK)$_4$(*K*EKA)$_2$*K*AKEAYCA-NH$_2$)** was obtained from the CEM Liberty Blue synthetiser as foamy colourless solid after preparative RP-HPLC (27.5 mg, 0.9 µmol, 8.3%). Analytical RP-HPLC: t_R=1.42 min (100% A to 100% D in 5 min, λ= 214 nm). $C_{306}H_{531}N_{83}O_{83}S$ calc./obs. 6728.9602/6728.9614.00 Da [M].

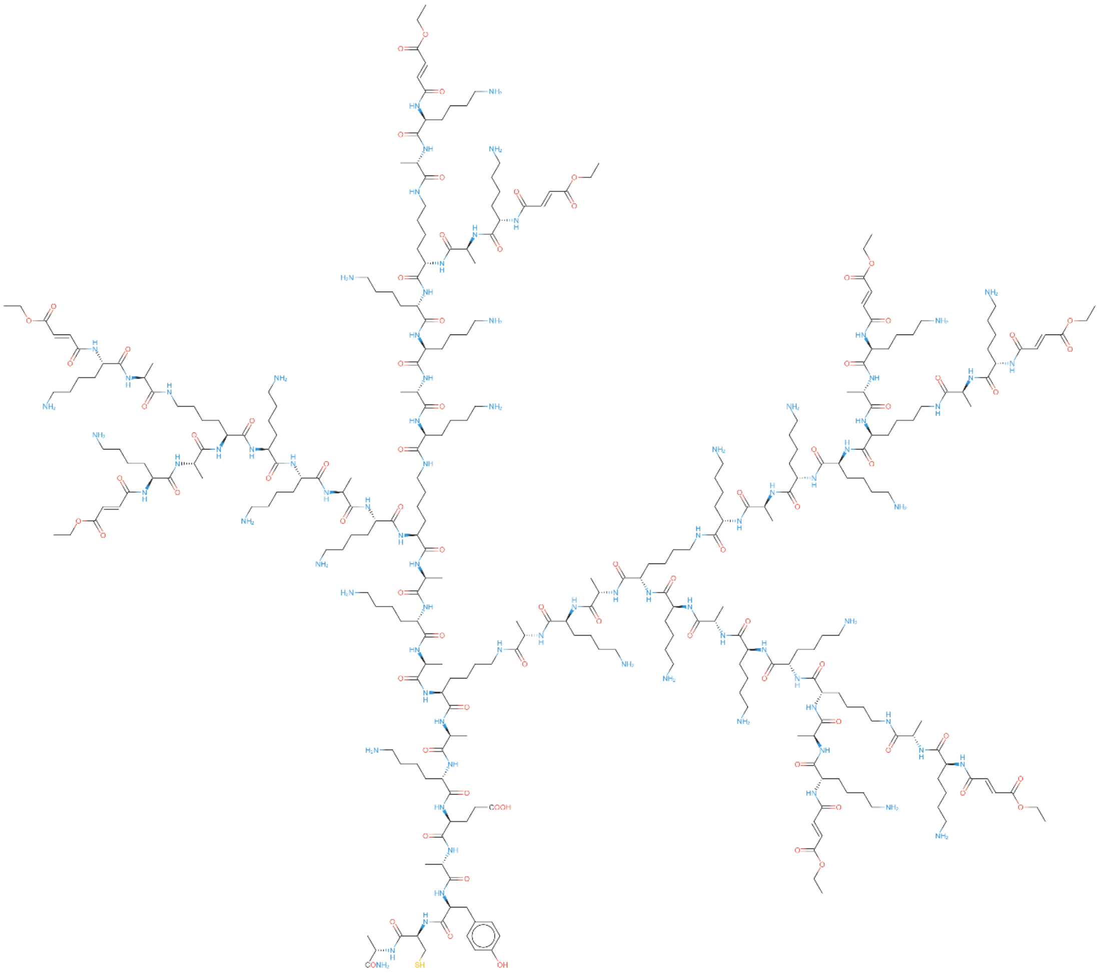

Analytical RP-HPLC:

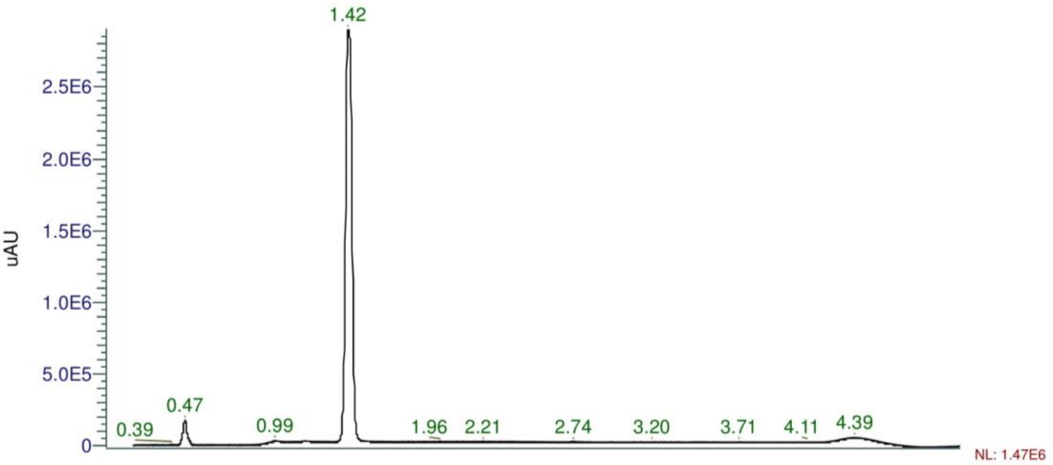

HRMS (NSI+):

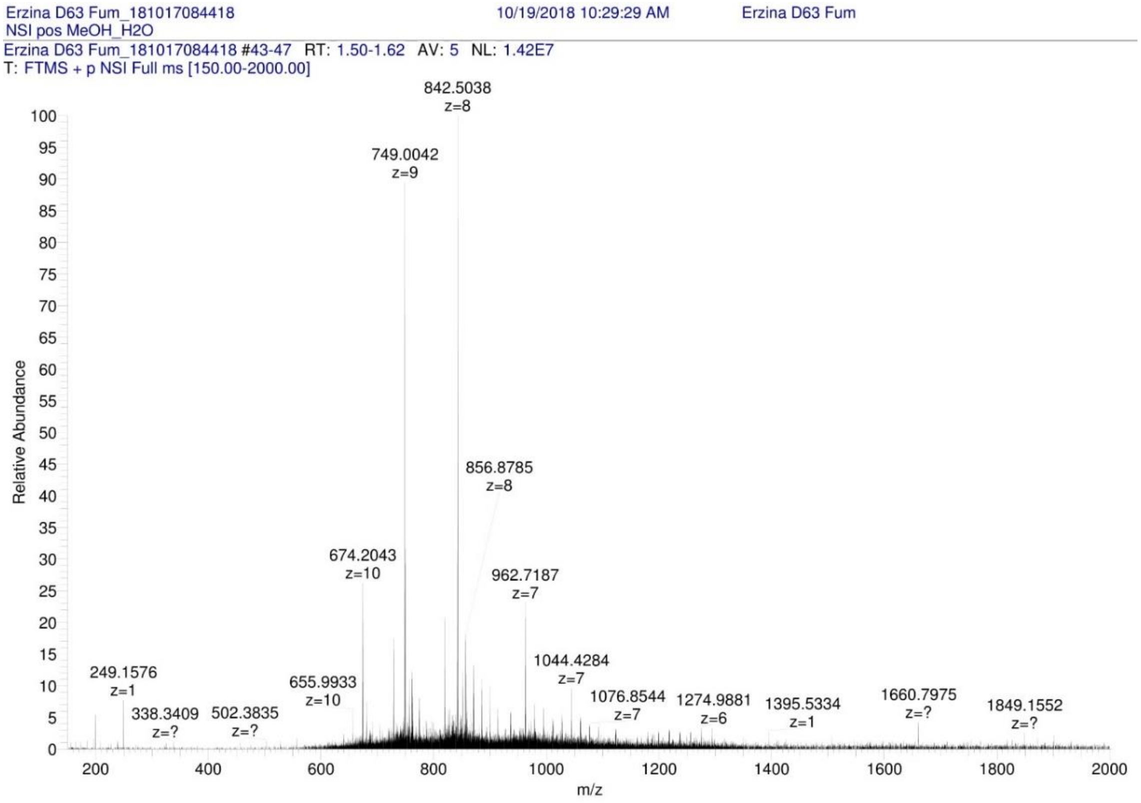

Erzina D63 Fum_181017084418
10/19/2018 10:29:29 AM
Erzina D63 Fum
NSI pos MeOH_H2O
Erzina D63 Fum_181017084418 #43-47 RT: 1.50-1.62 AV: 5 NL: 1.42E7
T: FTMS + p NSI Full ms [150.00-2000.00]
842.5038 z=8
749.0042 z=9
856.8785 z=8
674.2043 z=10
962.7187 z=7
1044.4284 z=7
249.1576 z=1
655.9933 z=10
1076.8544 z=7
1274.9881 z=6
1395.5334 z=1
1660.7975 z=?
1849.1552 z=?
338.3409 z=?
502.3835 z=?
Relative Abundance
m/z

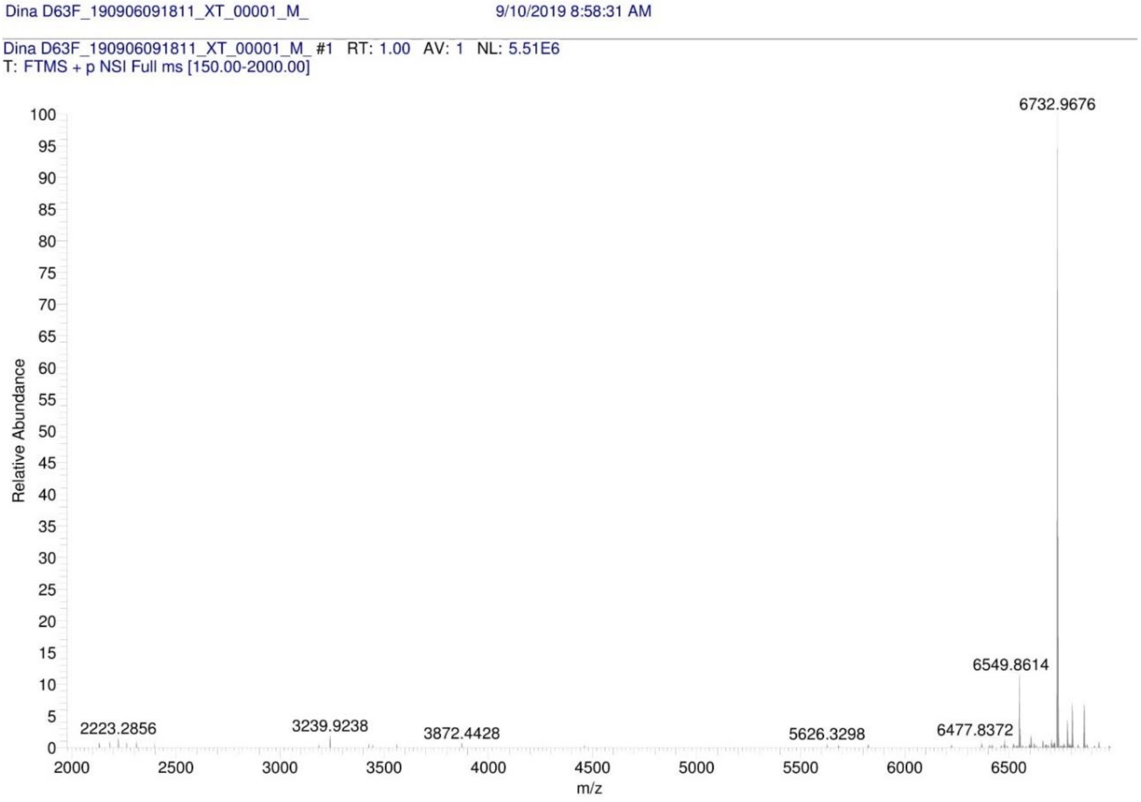

Dina D63F_190906091811_XT_00001_M_
9/10/2019 8:58:31 AM
Dina D63F_190906091811_XT_00001_M_ #1 RT: 1.00 AV: 1 NL: 5.51E6
T: FTMS + p NSI Full ms [150.00-2000.00]
6732.9676
6549.8614
2223.2856
3239.9238
3872.4428
5626.3298
6477.8372
Relative Abundance
m/z

Dina D63F_190906091811_XT_00001_M_ 9/10/2019 8:58:31 AM

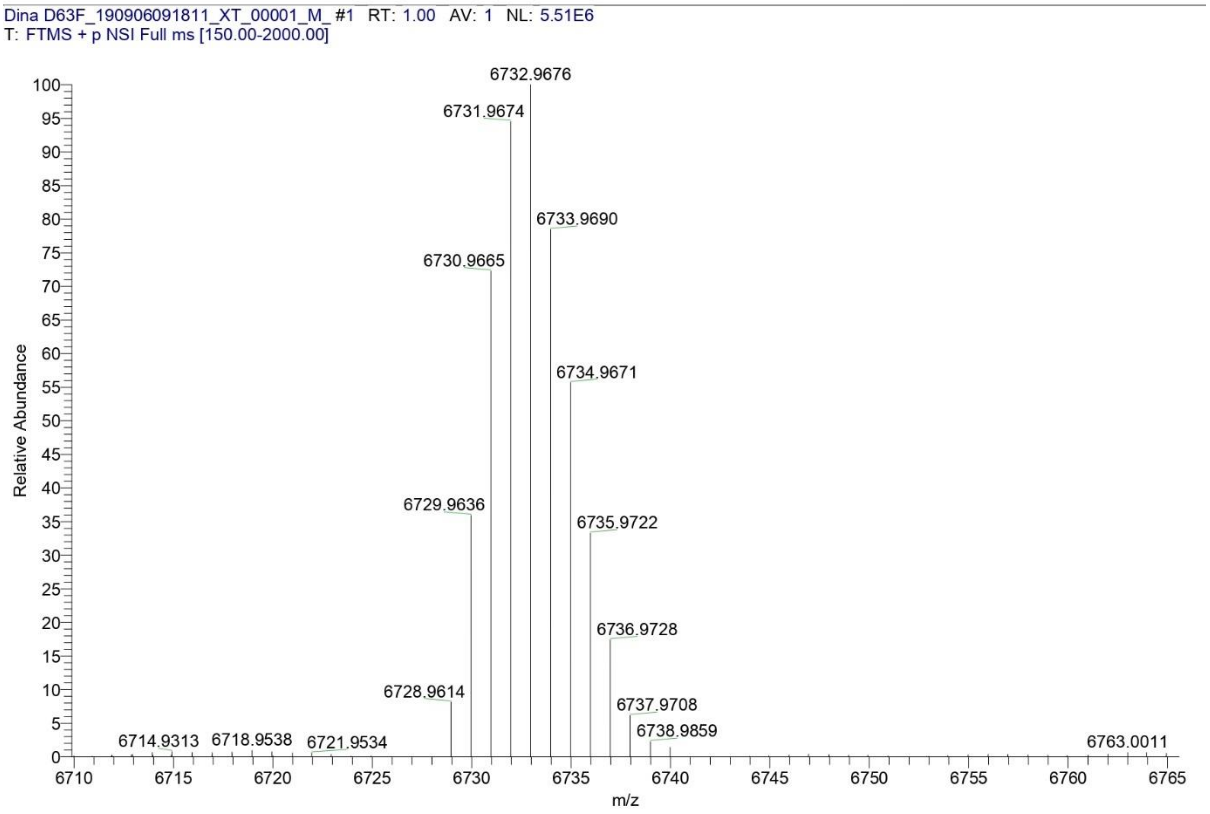

1-1 ((KA)$_8$(*K*KA)$_4$(*K*KAE)$_2$*K*YEKACA-NH$_2$)$_2$) for dimerization the initial dendrimer was refluxed in 50 mM NH_4HCO_3 buffer for 48 h, foamy colourless solid was obtained after preparative RP-HPLC (7.9 mg, 1.0 µmol, 43.6%). Analytical RP-HPLC: t_R=1.20 min (100% A to 100% D in 5 min, λ= 214 nm). HRMS (ESI+): $C_{414}H_{762}N_{132}O_{100}S_2$ calc./obs. 9247.8040/9247.8422 Da [M].

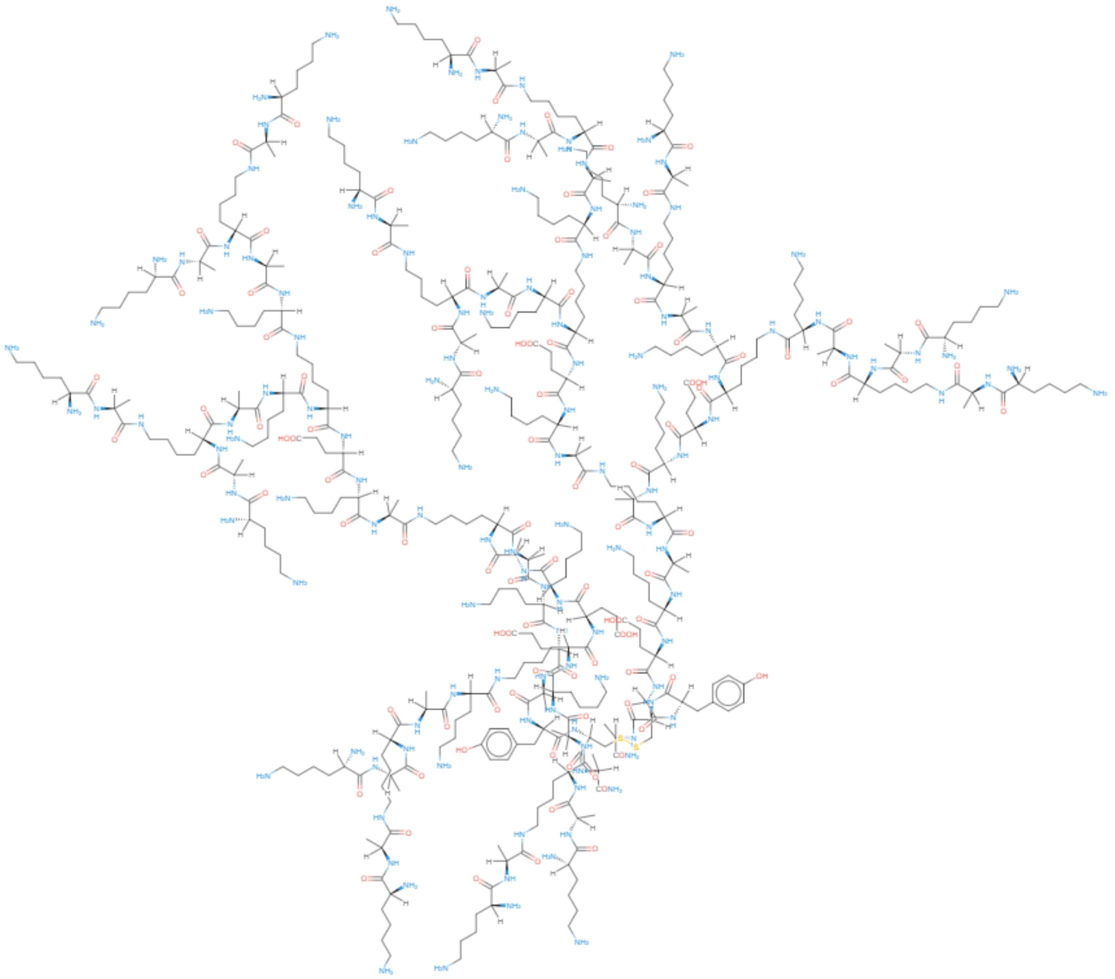

Analytical RP-HPLC:

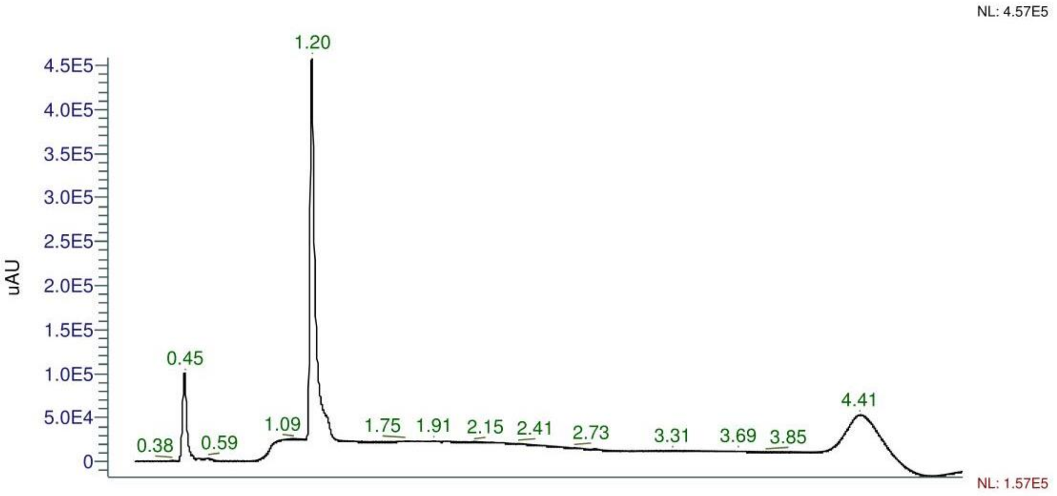

HRMS (NSI+):

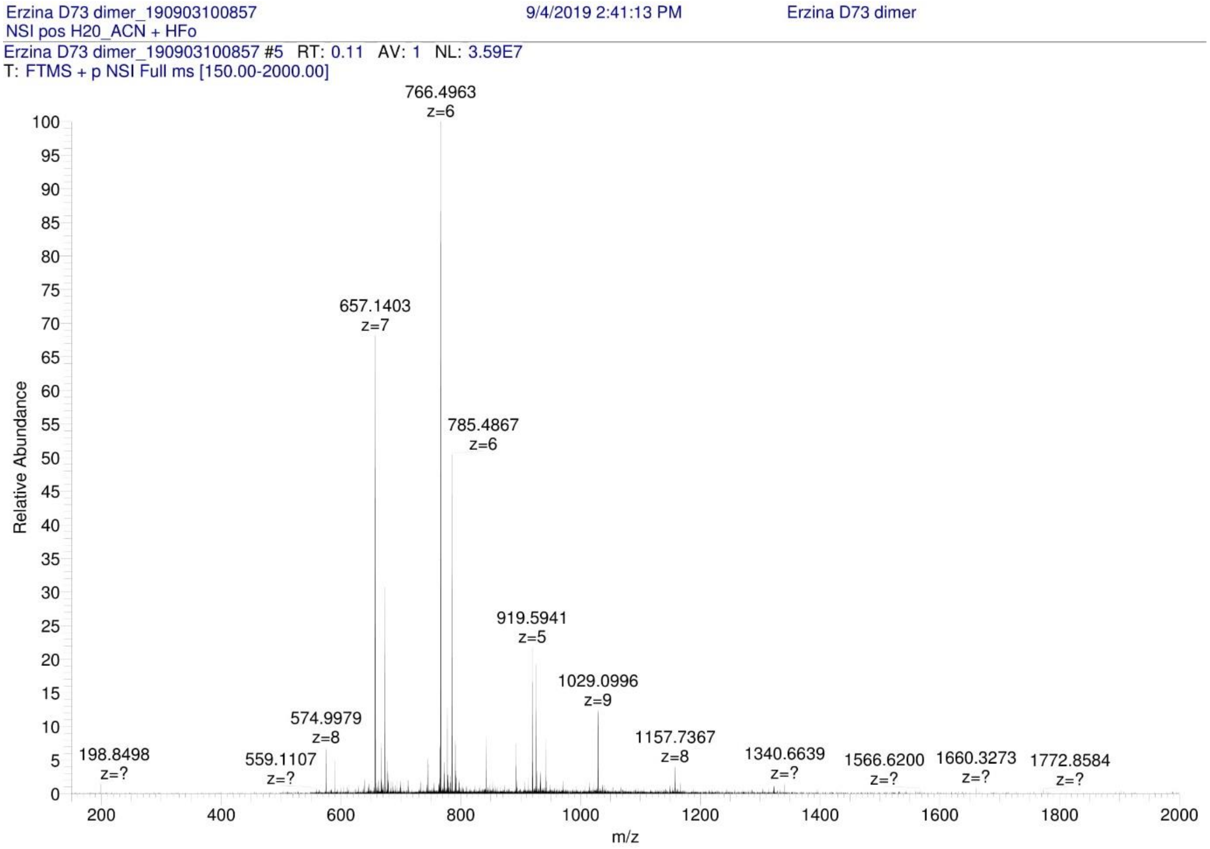
Erzina D73 dimer_190903100857
9/4/2019 2:41:13 PM
Erzina D73 dimer
NSI pos H20_ACN + HFo
Erzina D73 dimer_190903100857 #5 RT: 0.11 AV: 1 NL: 3.59E7
T: FTMS + p NSI Full ms [150.00-2000.00]
766.4963
z=6
657.1403
z=7
785.4867
z=6
919.5941
z=5
1029.0996
z=9
574.9979
z=8
1157.7367
z=8
198.8498
z=?
559.1107
z=?
1340.6639
z=?
1566.6200
z=?
1660.3273
z=?
1772.8584
z=?
Relative Abundance
m/z

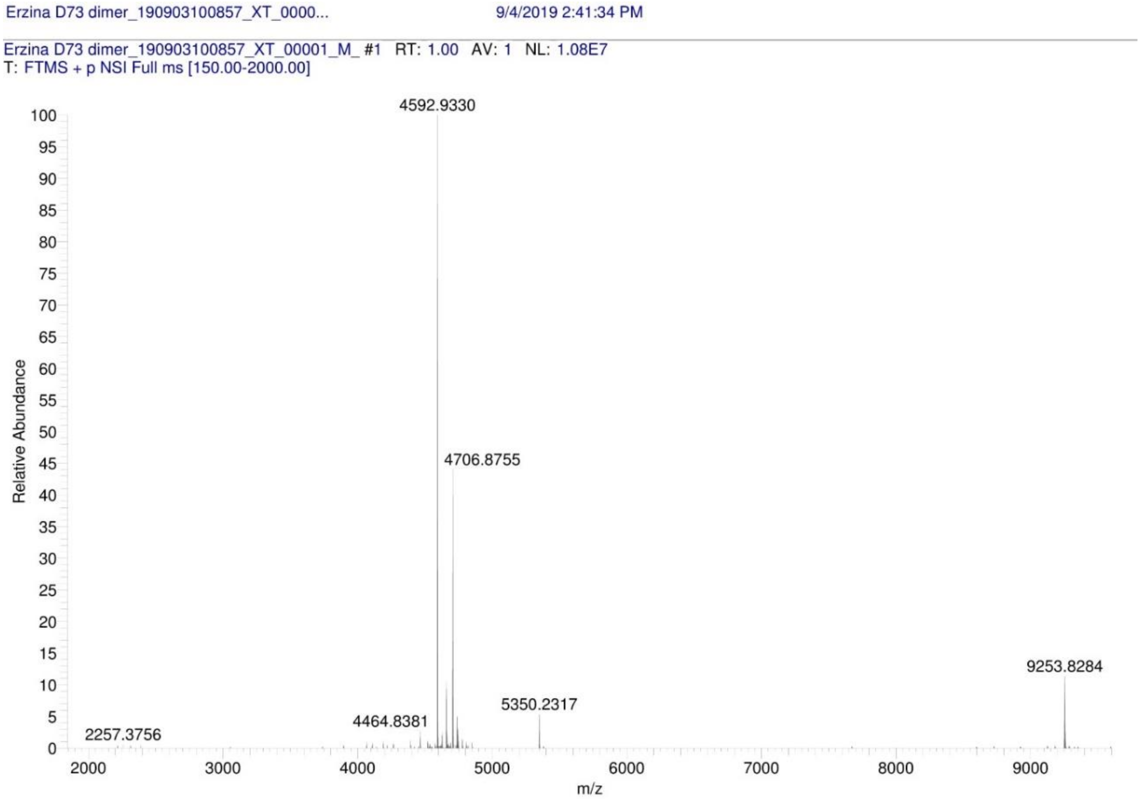
Erzina D73 dimer_190903100857_XT_0000...
9/4/2019 2:41:34 PM
Erzina D73 dimer_190903100857_XT_00001_M_ #1 RT: 1.00 AV: 1 NL: 1.08E7
T: FTMS + p NSI Full ms [150.00-2000.00]
4592.9330
4706.8755
9253.8284
5350.2317
4464.8381
2257.3756
Relative Abundance
m/z

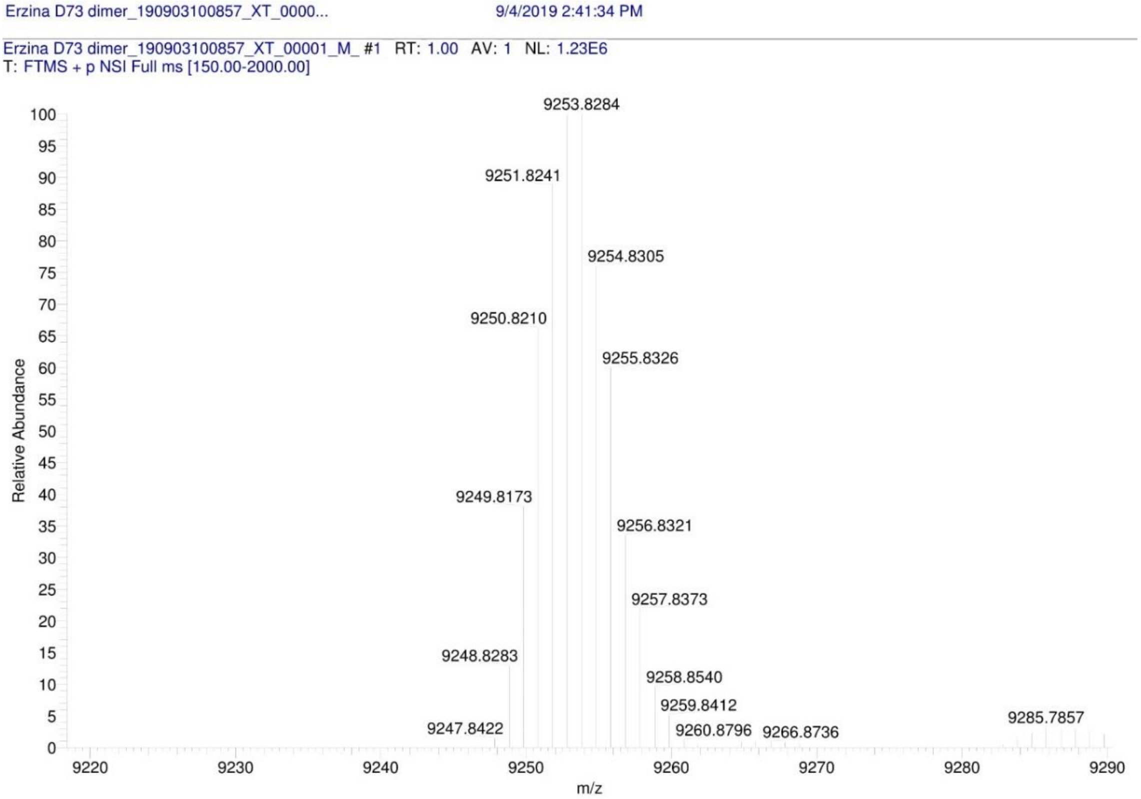
Erzina D73 dimer_190903100857_XT_0000...
9/4/2019 2:41:34 PM
Erzina D73 dimer_190903100857_XT_00001_M_ #1 RT: 1.00 AV: 1 NL: 1.23E6
T: FTMS + p NSI Full ms [150.00-2000.00]
9253.8284
9251.8241
9254.8305
9250.8210
9255.8326
9249.8173
9256.8321
9257.8373
9248.8283
9258.8540
9259.8412
9247.8422
9260.8796
9266.8736
9285.7857
Relative Abundance
m/z

D-**1** **((ka)$_8$(*k*ak)$_4$(*k*eka)$_2$*k*akeayca-NH$_2$)** was obtained from the CEM Liberty Blue synthetiser as foamy colourless solid after preparative RP-HPLC (56.8 mg, 2.5 µmol, 18.3%). Analytical RP-HPLC: t_R=1.33 min (100% A to 100% D in 5 min, λ= 214 nm). HRMS (ESI+): $C_{210}H_{387}N_{67}O_{51}S$ calc./obs. 4695.9470/4695.9606 Da [M].

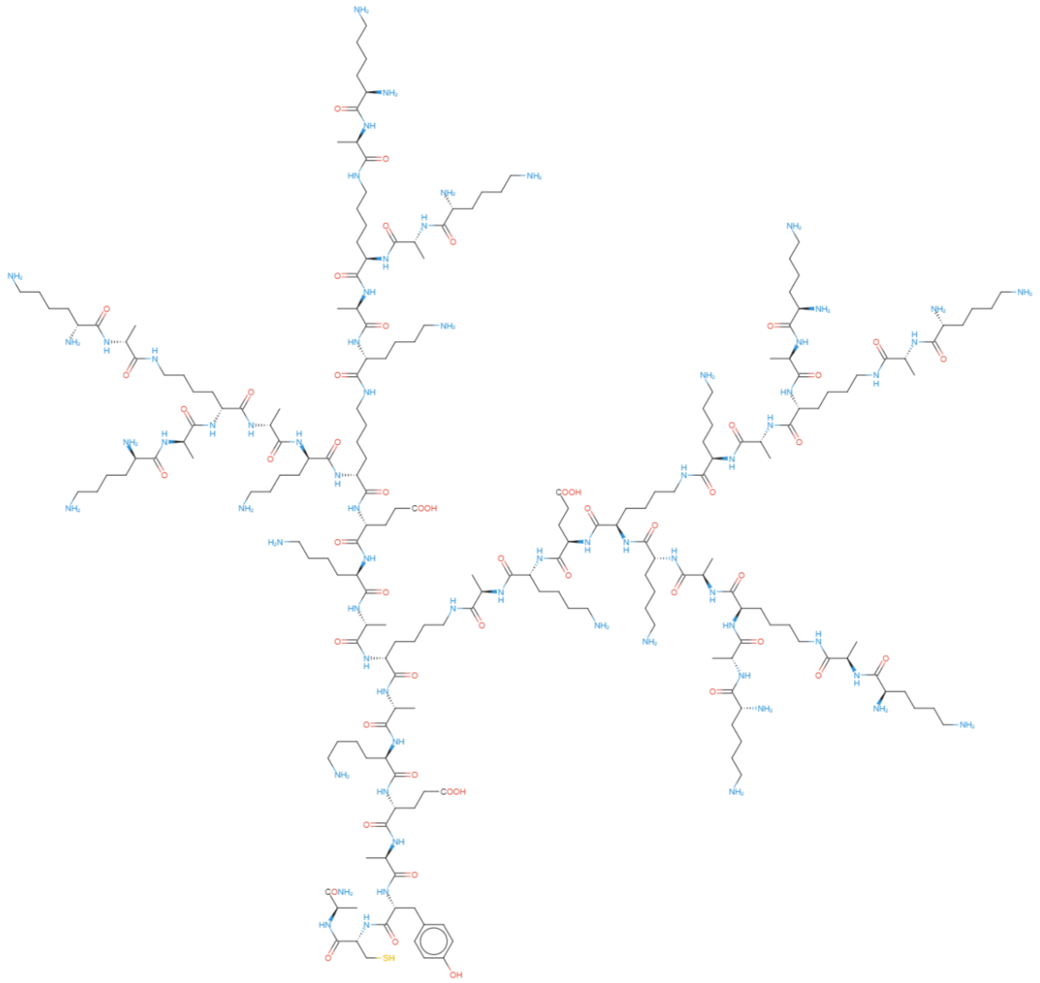

Analytical RP-HPLC:

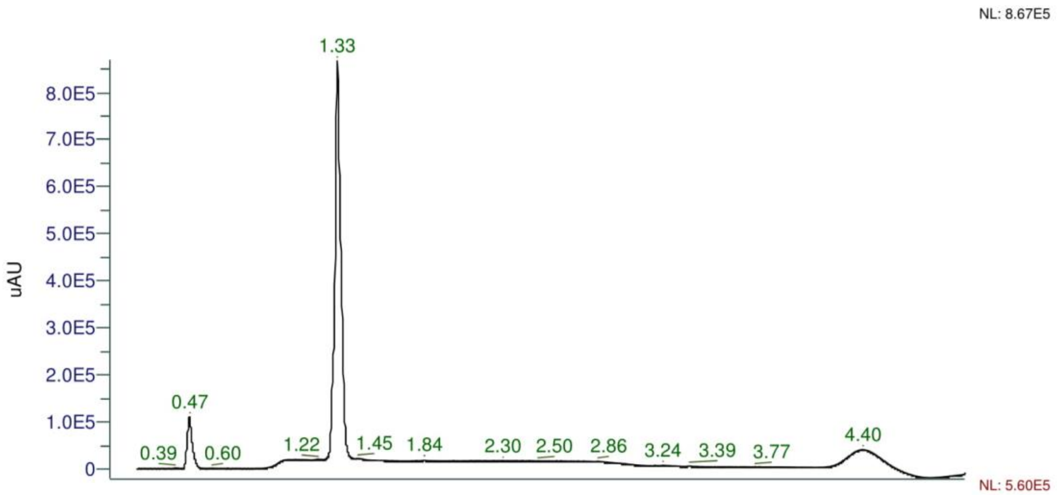

HRMS (NSI+):

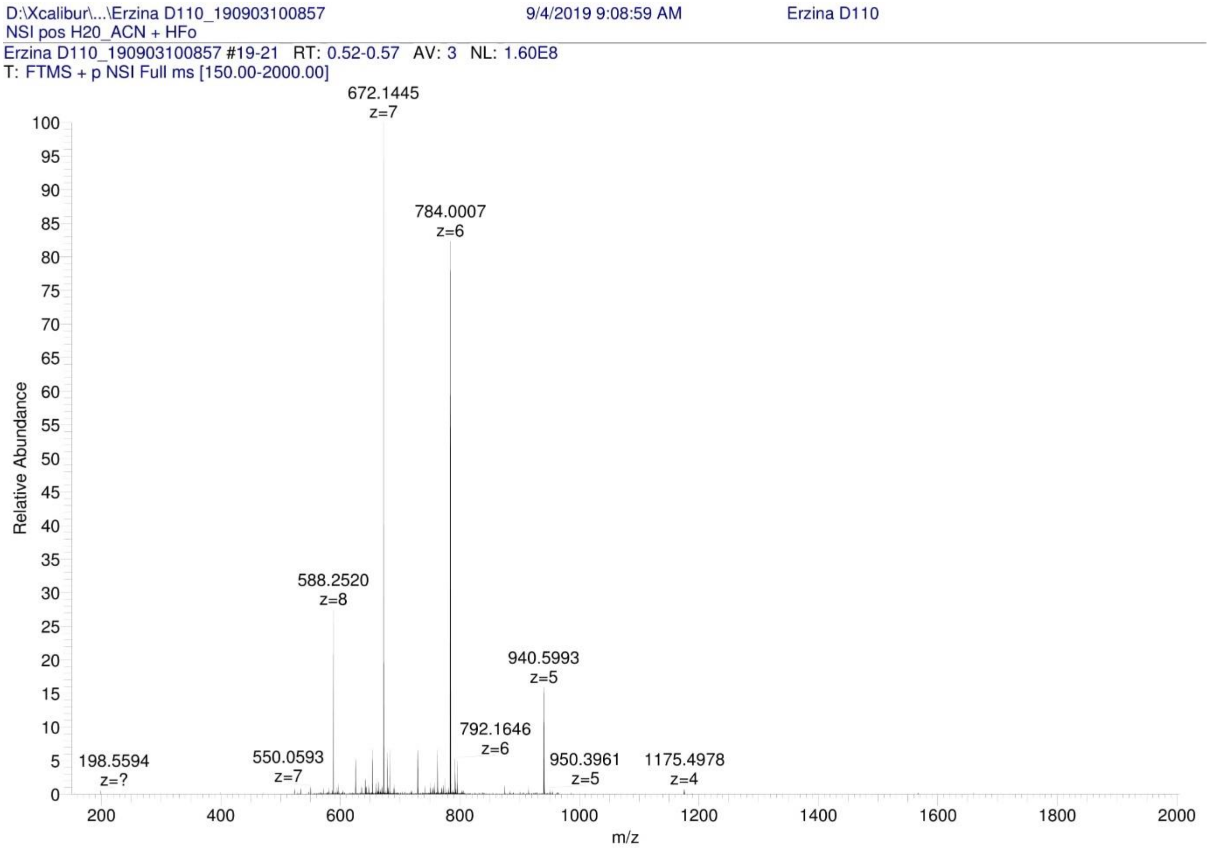
D:\Xcalibur\...\Erzina D110_190903100857
9/4/2019 9:08:59 AM
Erzina D110
NSI pos H20_ACN + HFo
Erzina D110_190903100857 #19-21 RT: 0.52-0.57 AV: 3 NL: 1.60E8
T: FTMS + p NSI Full ms [150.00-2000.00]
672.1445 z=7
784.0007 z=6
588.2520 z=8
940.5993 z=5
792.1646 z=6
198.5594 z=?
550.0593 z=7
950.3961 z=5
1175.4978 z=4
Relative Abundance
m/z

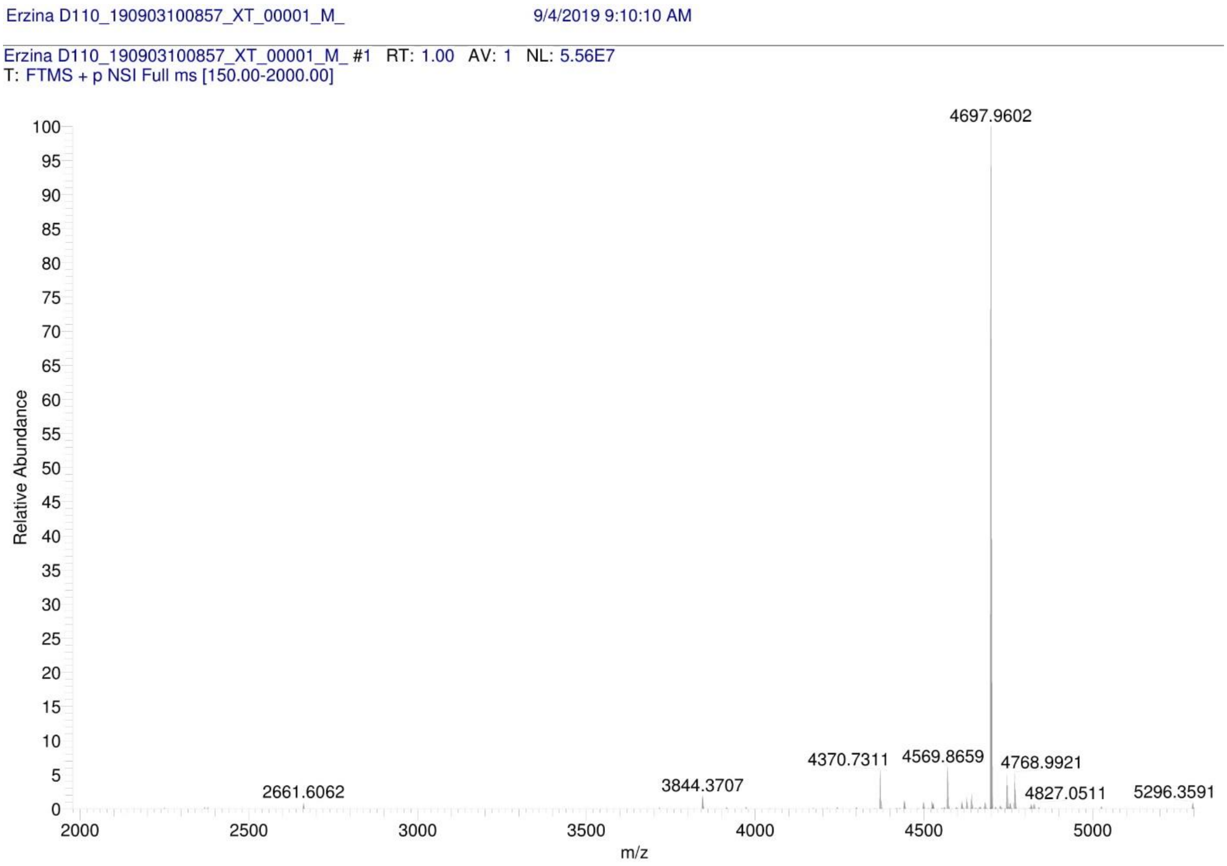
Erzina D110_190903100857_XT_00001_M_
9/4/2019 9:10:10 AM
Erzina D110_190903100857_XT_00001_M_ #1 RT: 1.00 AV: 1 NL: 5.56E7
T: FTMS + p NSI Full ms [150.00-2000.00]
4697.9602
4370.7311
4569.8659
4768.9921
3844.3707
2661.6062
4827.0511
5296.3591
Relative Abundance
m/z

Erzina D110_190903100857_XT_00001_M_ 9/4/2019 9:10:10 AM

Erzina D110_190903100857_XT_00001_M_ #1 RT: 1.00 AV: 1 NL: 5.56E7
T: FTMS + p NSI Full ms [150.00-2000.00]

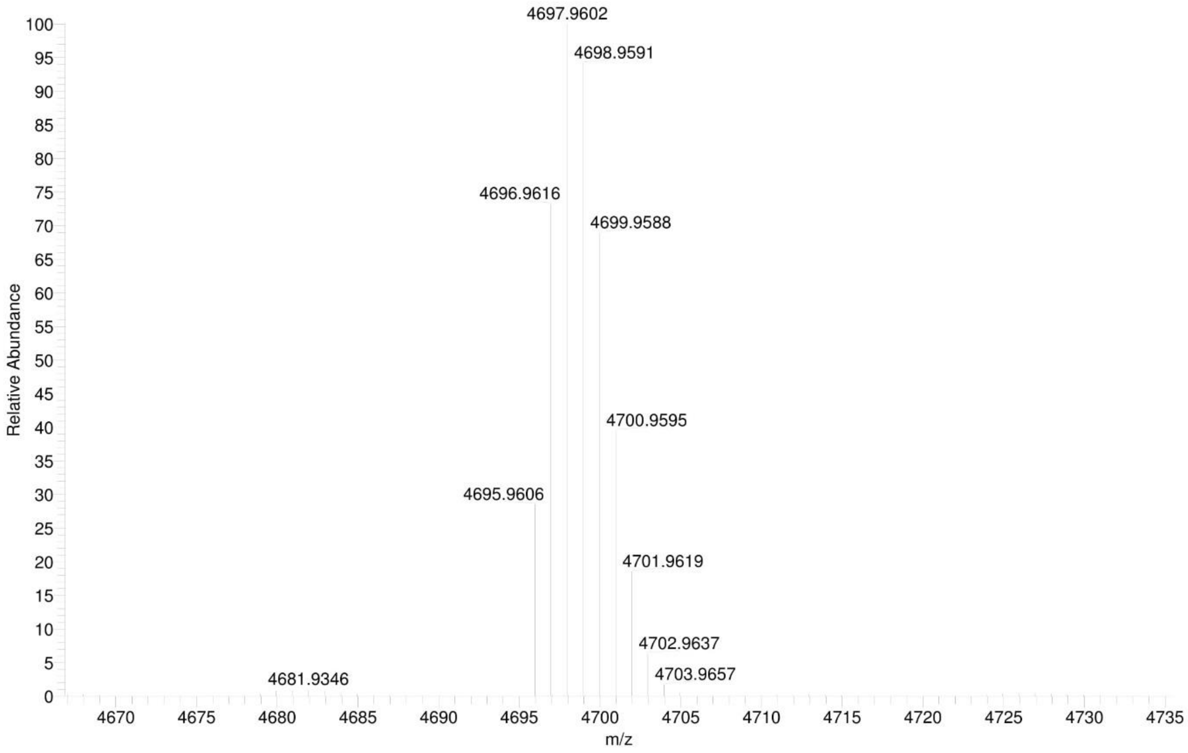

sr-**1 ((KA)$_8$(*K*KA)$_4$(*K*KAE)$_2$*K*YEKACA-NH$_2$)** was obtained after manual synthesis as foamy colourless solid after preparative RP-HPLC (32.9 mg, 1.0 µmol, 7.6%). Analytical RP-HPLC: t_R=1.18 min (100% A to 100% D in 5 min, λ= 214 nm). HRMS (ESI+): $C_{207}H_{382}N_{66}O_{50}S$ calc./obs. 4624.9098/4624.9212 Da [M].

Analytical RP-HPLC:

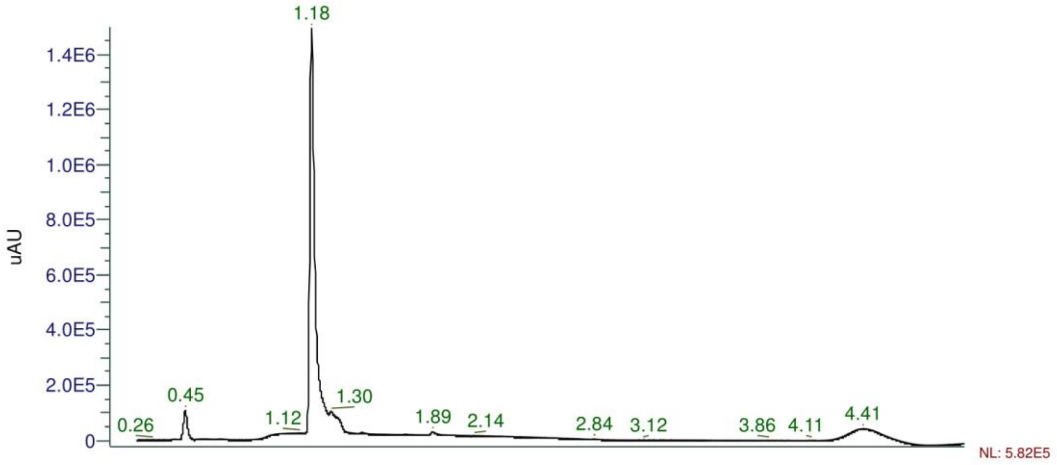

HRMS (NSI+):

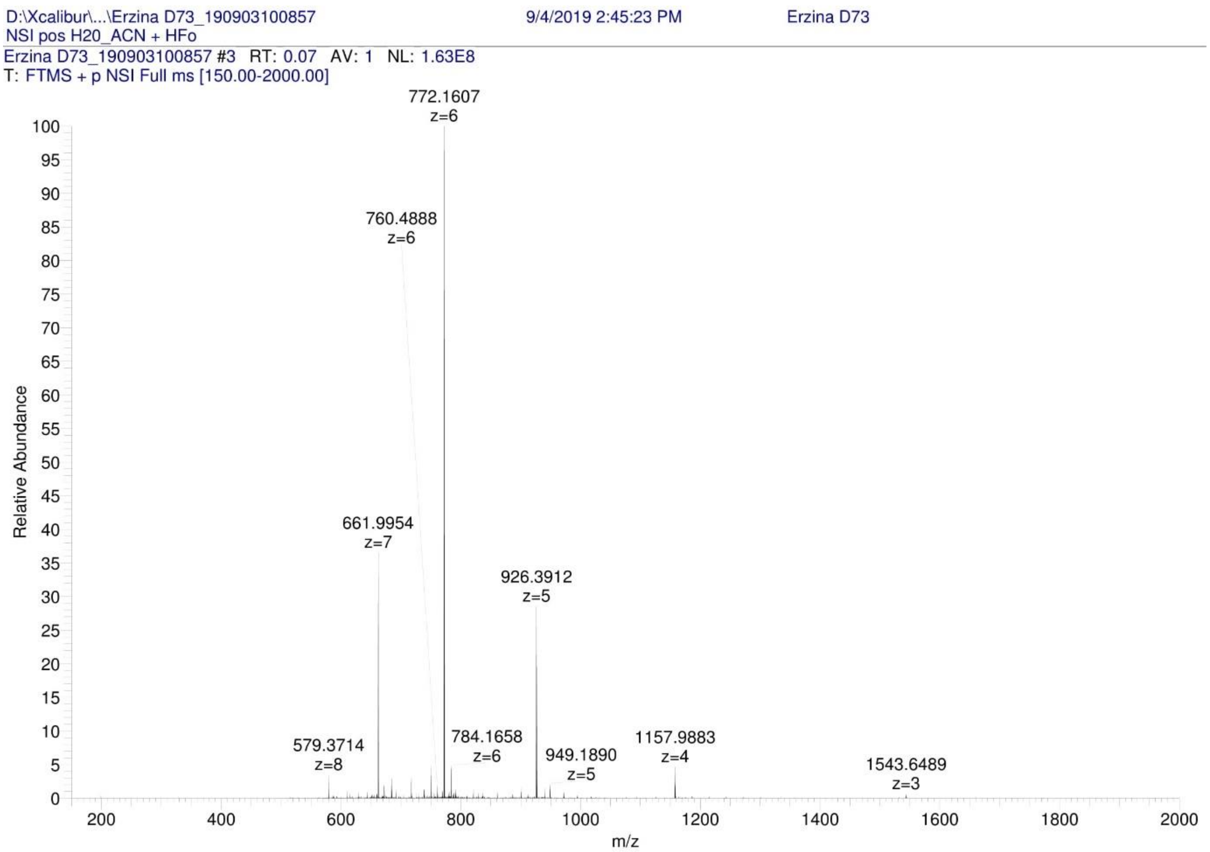

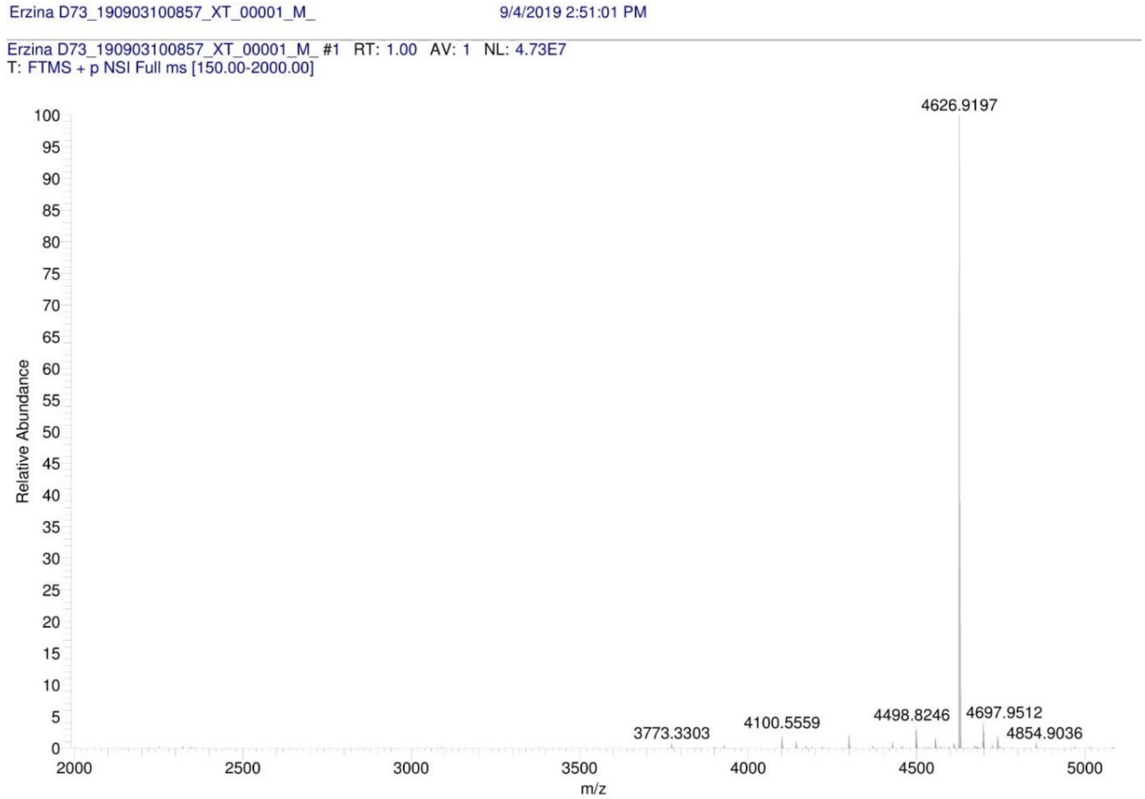
Erzina D73_190903100857_XT_00001_M_
9/4/2019 2:51:01 PM
Erzina D73_190903100857_XT_00001_M_ #1 RT: 1.00 AV: 1 NL: 4.73E7
T: FTMS + p NSI Full ms [150.00-2000.00]
4626.9197
3773.3303
4100.5559
4498.8246
4697.9512
4854.9036
Relative Abundance
m/z

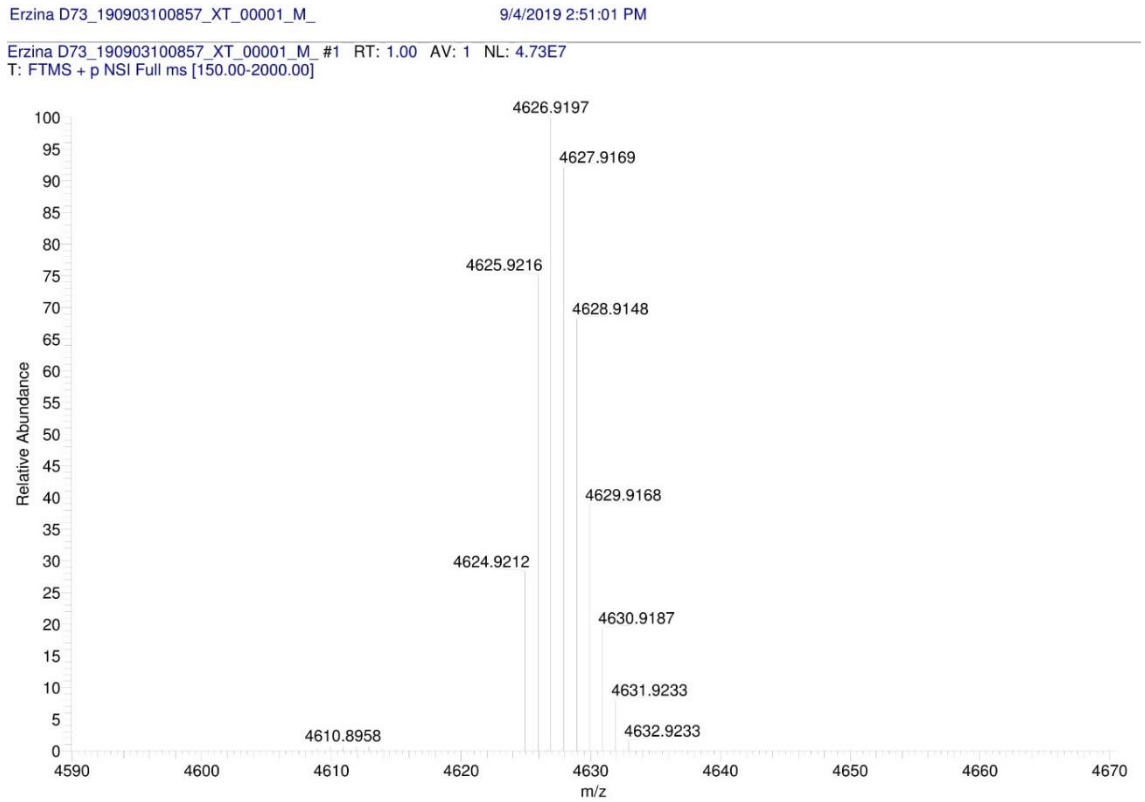
Erzina D73_190903100857_XT_00001_M_
9/4/2019 2:51:01 PM
Erzina D73_190903100857_XT_00001_M_ #1 RT: 1.00 AV: 1 NL: 4.73E7
T: FTMS + p NSI Full ms [150.00-2000.00]
4626.9197
4627.9169
4625.9216
4628.9148
4629.9168
4624.9212
4630.9187
4631.9233
4632.9233
4610.8958
Relative Abundance
m/z

D-**4** **((ka)$_8$(*k*kake)$_4$(*k*ykaka)$_2$*k*aykka-NH$_2$)** was obtained from the CEM Liberty Blue synthetiser as foamy colourless solid after preparative RP-HPLC (67.1 mg, 1.8 µmol, 16.7%). Analytical RP-HPLC: t_R=1.37 min (100% A to 100% D in 5 min, λ= 214 nm). HRMS (ESI+): $C_{275}H_{496}N_{84}O_{65}$ calc./obs. 6015.8089/6015.8149 Da [M].

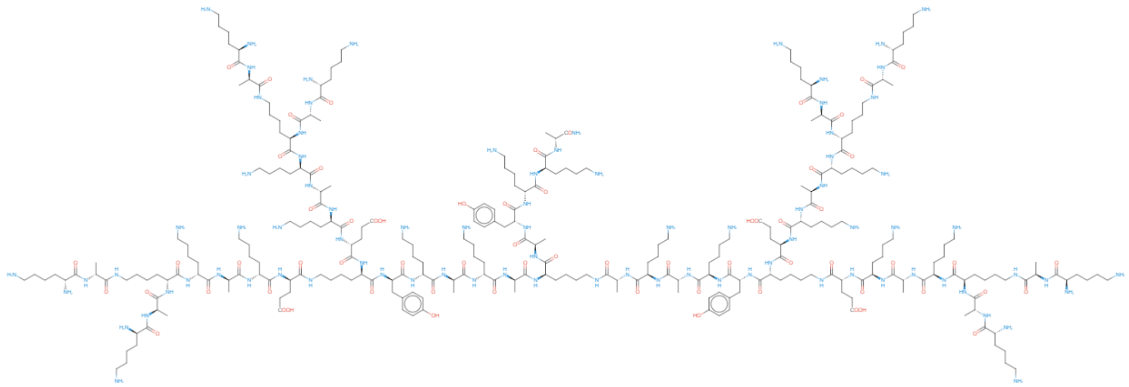

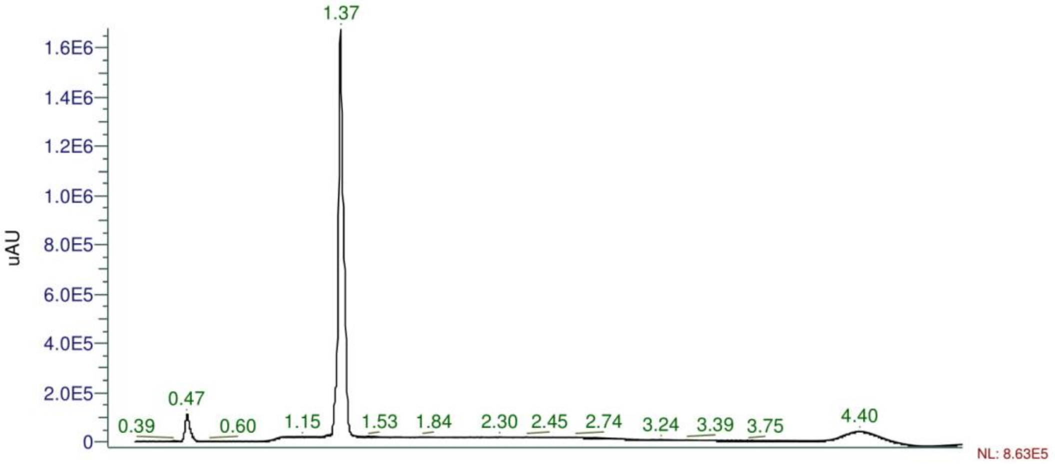

HRMS (NSI+):

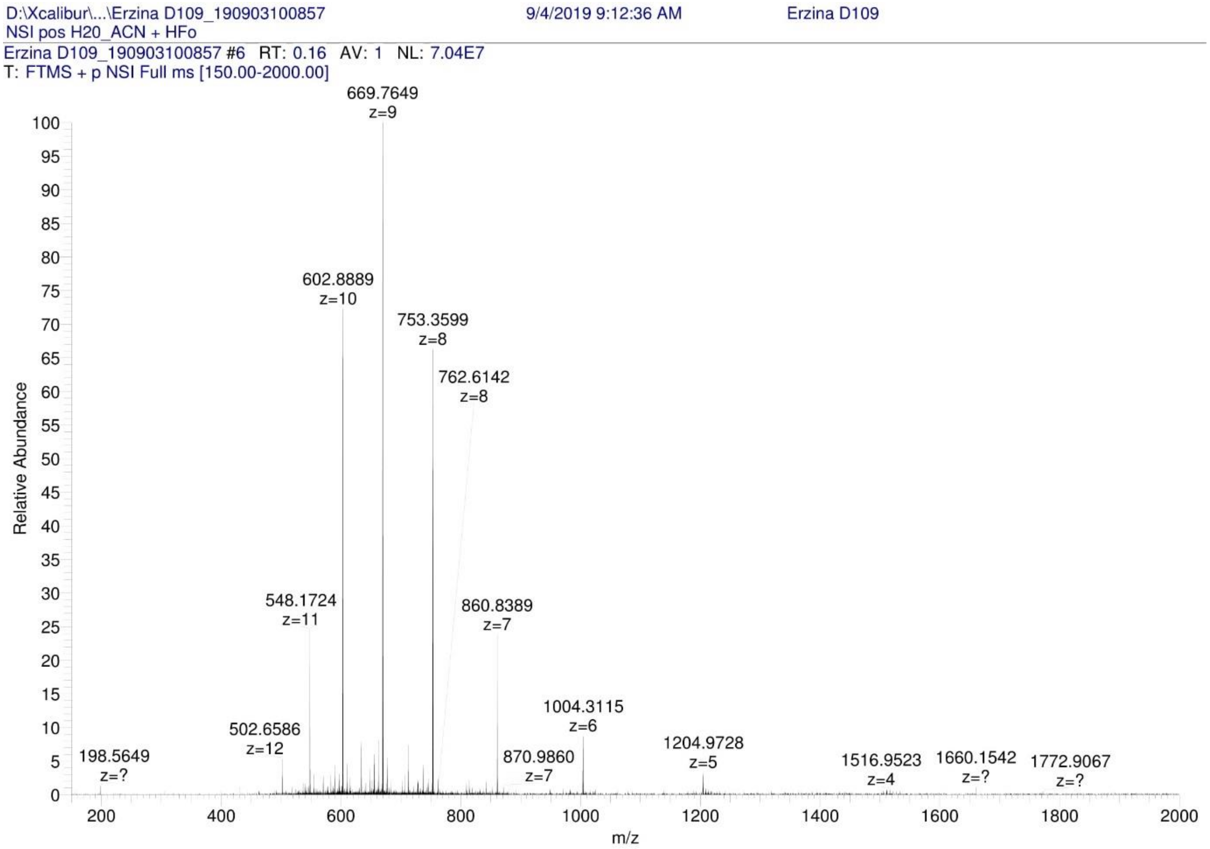
D:\Xcalibur\...\Erzina D109_190903100857
9/4/2019 9:12:36 AM
Erzina D109
NSI pos H20_ACN + HFo
Erzina D109_190903100857 #6 RT: 0.16 AV: 1 NL: 7.04E7
T: FTMS + p NSI Full ms [150.00-2000.00]
669.7649 z=9
602.8889 z=10
753.3599 z=8
762.6142 z=8
548.1724 z=11
860.8389 z=7
1004.3115 z=6
502.6586 z=12
1204.9728 z=5
870.9860 z=7
198.5649 z=?
1516.9523 z=4
1660.1542 z=?
1772.9067 z=?
Relative Abundance
m/z

Erzina D109_190903100857_XT_00001_M_ 9/4/2019 9:12:57 AM

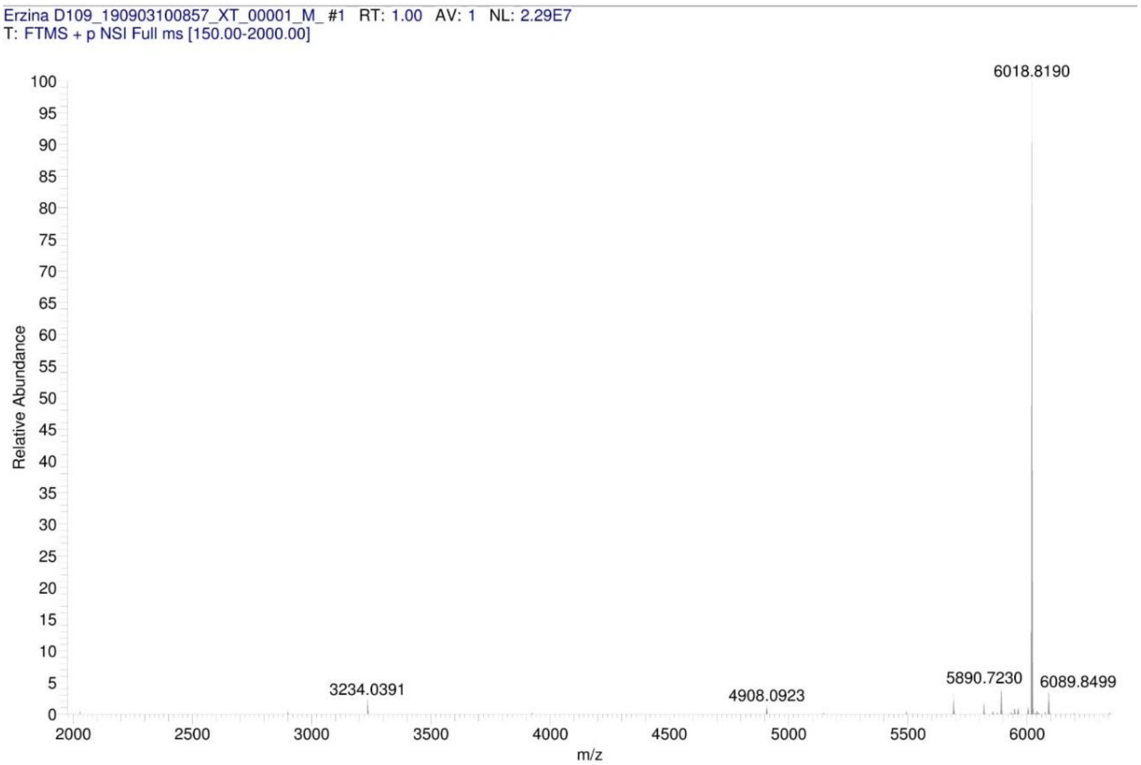

Erzina D109_190903100857_XT_00001_M_ 9/4/2019 9:12:57 AM

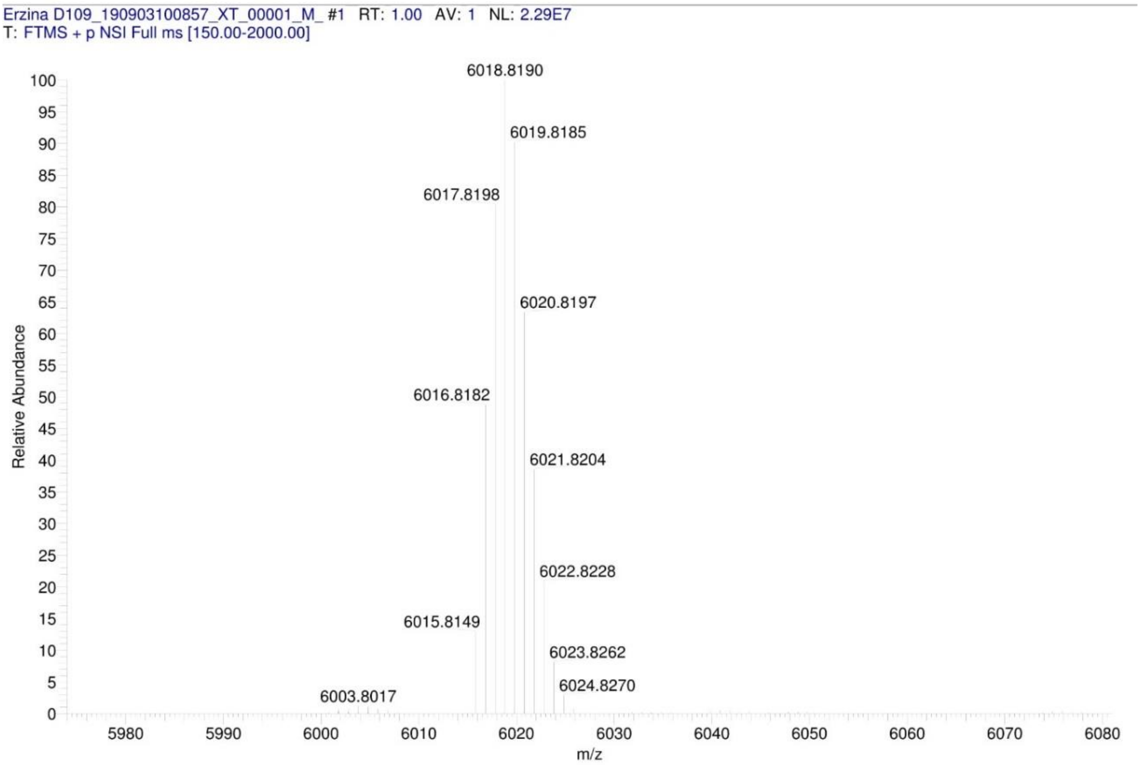

sr-**4 ((KA)$_8$(*K*EKAK)$_4$(*K*AYAKK)$_2$*K*KAYAK-OH)** was obtained after manual synthesis as foamy colourless solid after preparative RP-HPLC (89.6 mg, 1.7 µmol, 15.6%). Analytical RP-HPLC: t_R=1.19 min (100% A to 100% D in 5 min, λ= 214 nm). HRMS (ESI+): $C_{275}H_{495}N_{83}O_{66}S$ calc./obs. 6016.7929/6016.8081 Da [M].

Analytical RP-HPLC:

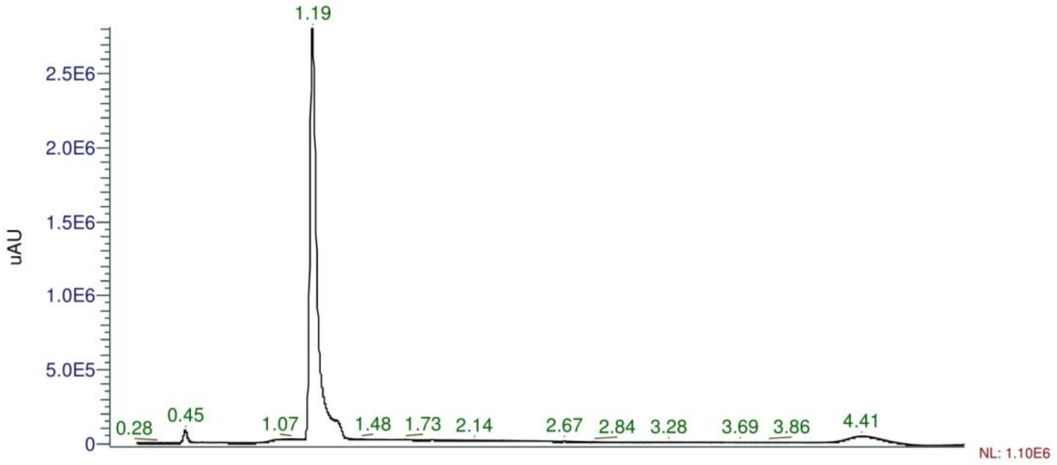

HRMS (NSI+):

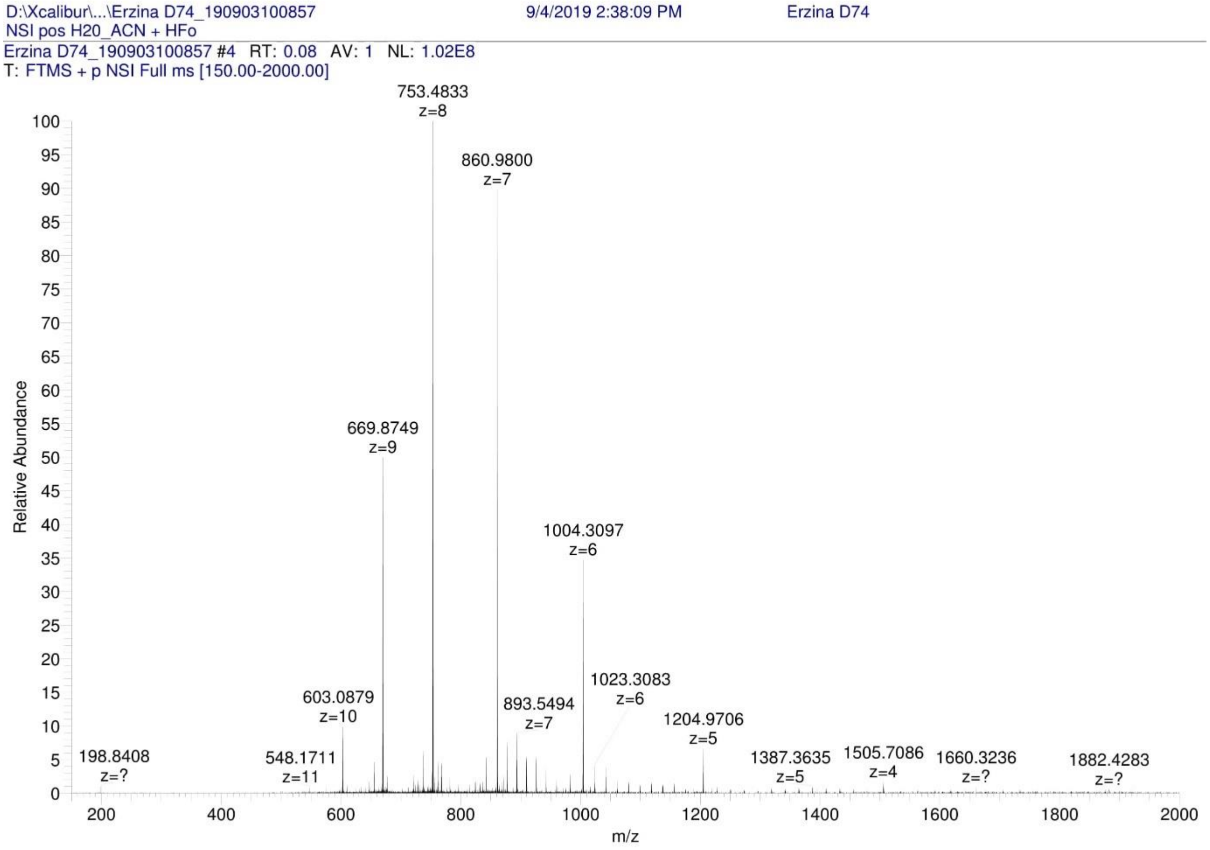

Erzina D74_190903100857_XT_00001_M_ 9/4/2019 2:38:26 PM

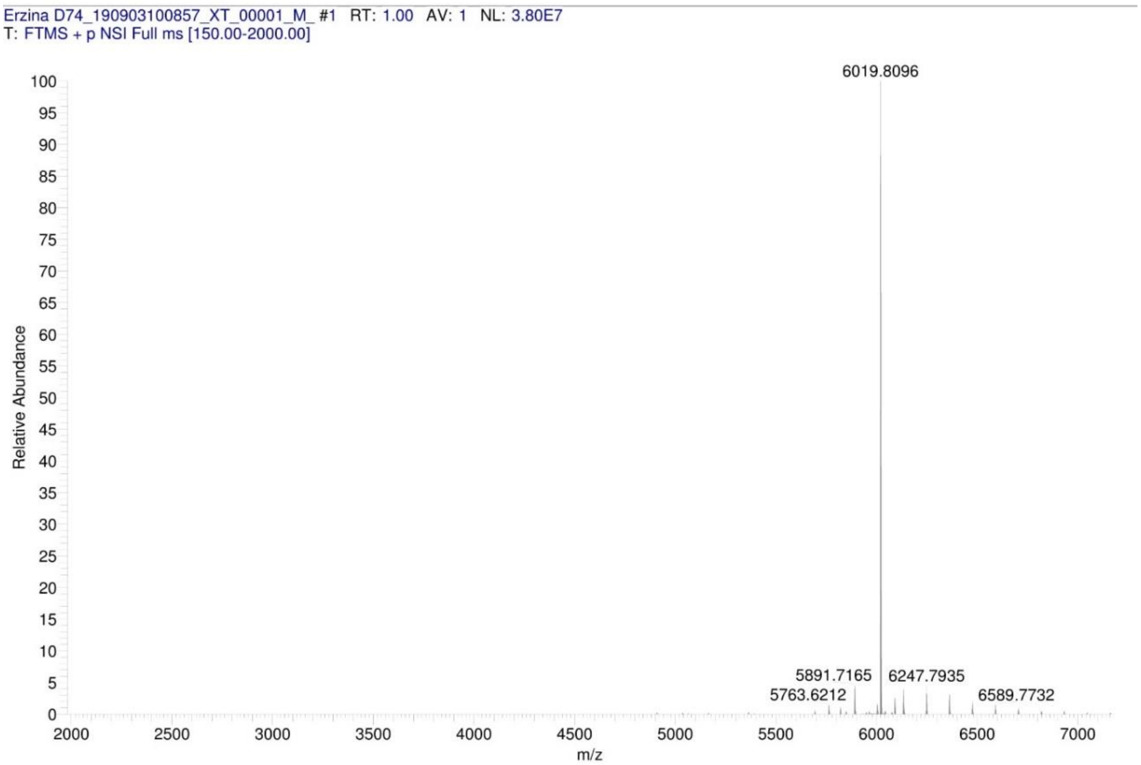

Erzina D74_190903100857_XT_00001_M_ 9/4/2019 2:38:26 PM

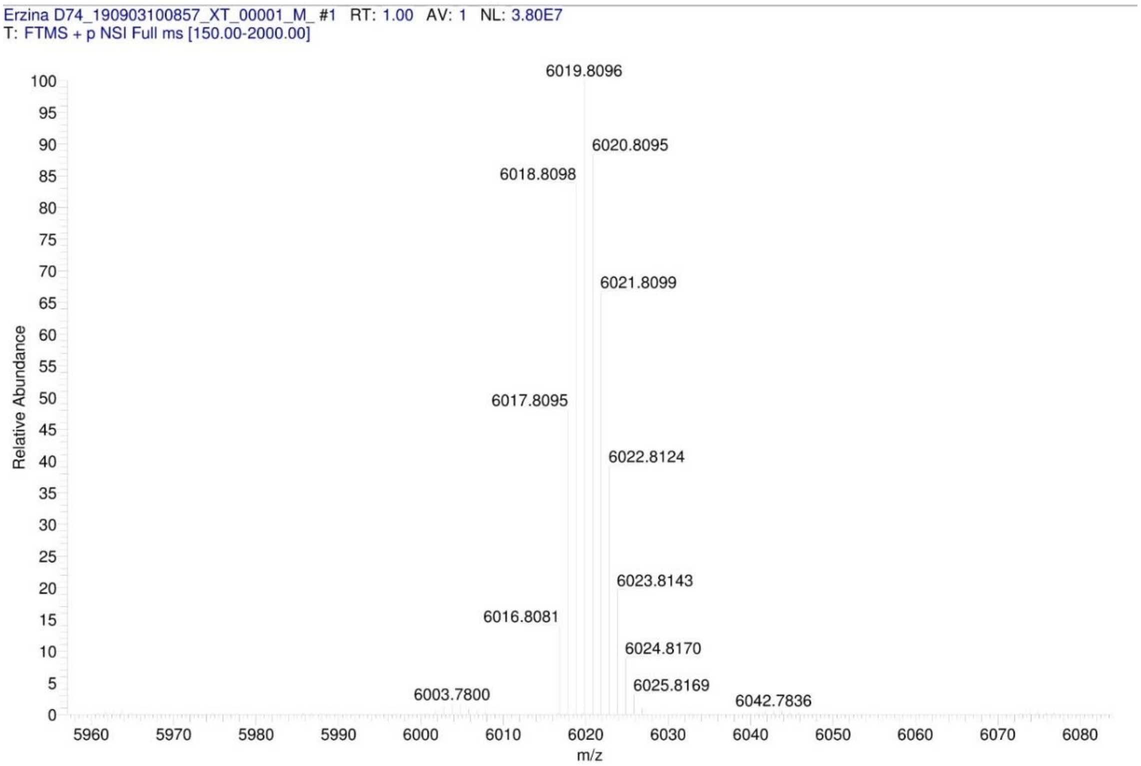

1Fl **((KA)$_8$(*K*AK)$_4$(*K*EKA)$_2$*K*AKEAYC(Fl)A-NH$_2$)** initial dendrimer was obtained as foamy white solid after preparative RP-HPLC, then 1eq. Of dendrimer was coupled with 1.1 eq. of fluorescein-diacetat-5-maleinimid in H_2O/ACN solution with NH_4HCO_3 50 mM buffer, pH 8. (6.5 mg, 2.1 µmol, 40.6%). Analytical RP-HPLC: t_R=1.16 min (100% A to 100% D in 5 min, λ= 214 nm). HRMS (ESI+): $C_{238}H404N_{68}O_{60}S$ calc./obs. 5207.0373/5207.0391 Da [M].

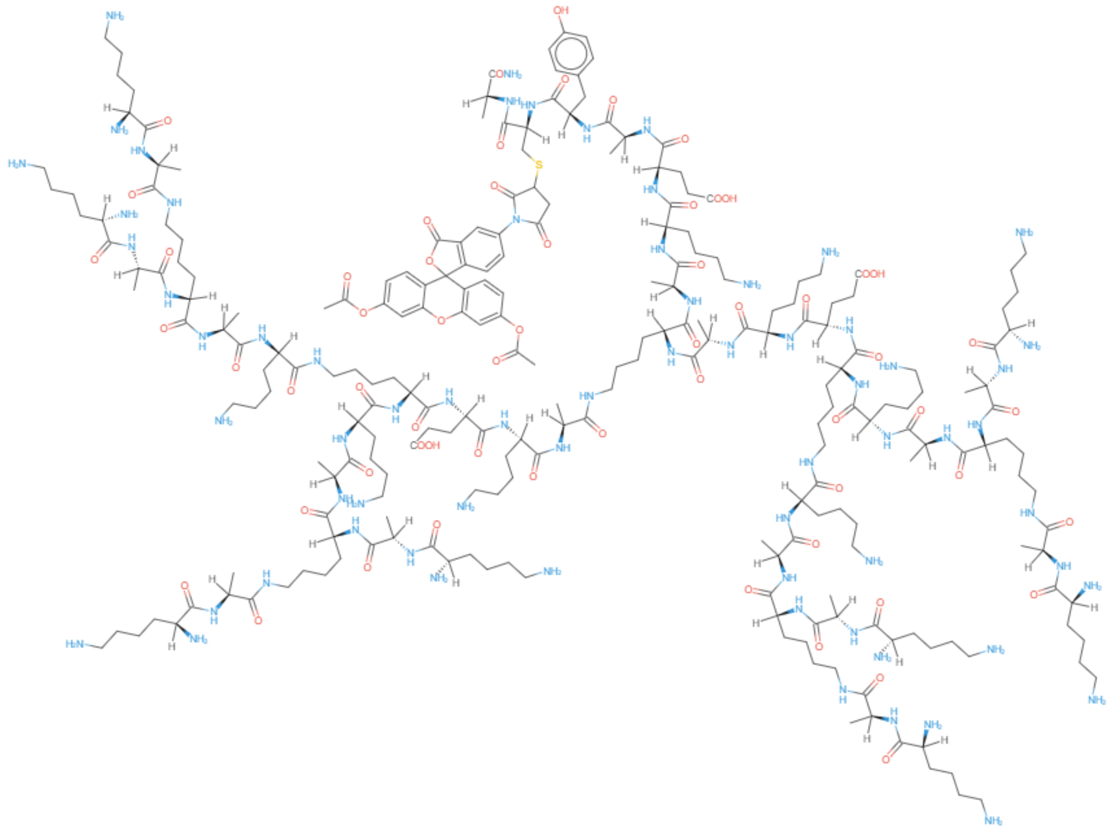

Analytical RP-HPLC:

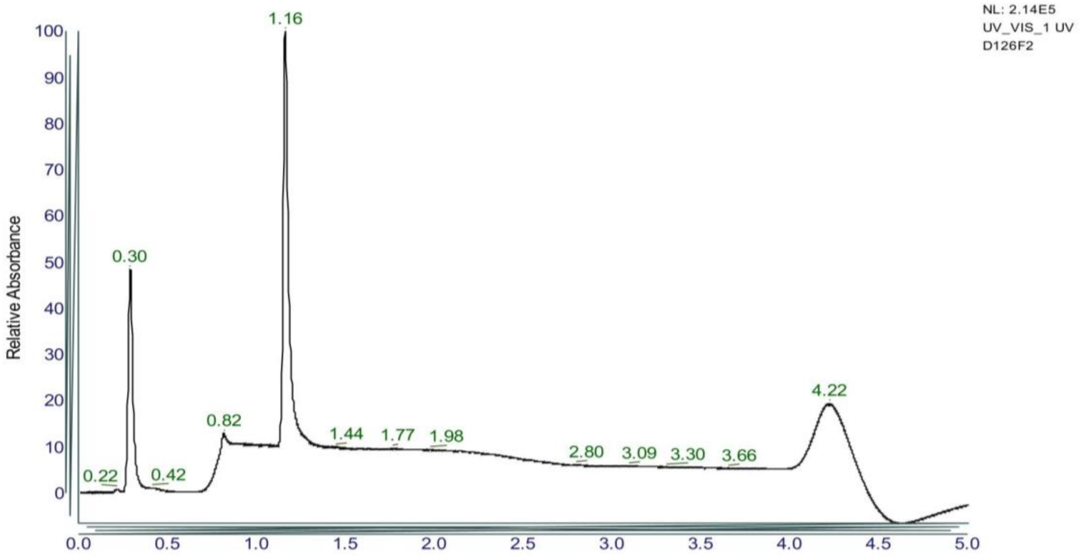

HRMS (NSI+):

Erzina D126_200715102840_XT_00001_M_ 7/16/2020 9:04:12 AM

Erzina D126_200715102840_XT_00001_M_ #1 RT: 1.00 AV: 1 NL: 1.04E6
T: FTMS + p NSI Full ms [150.00-2000.00]

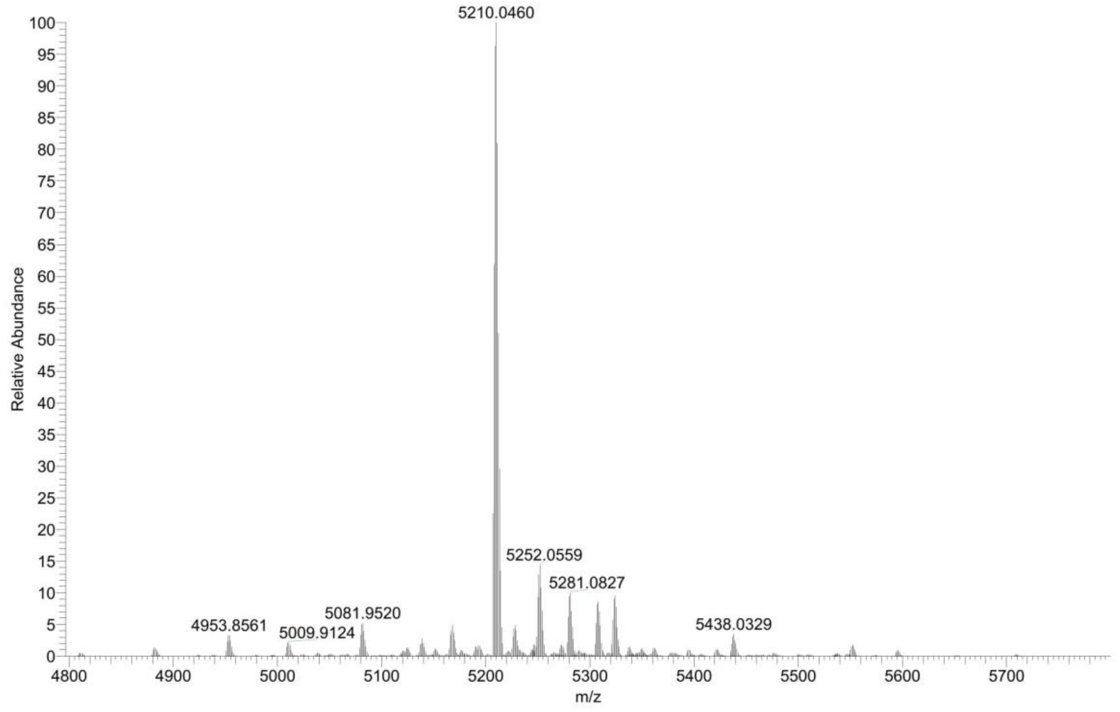

D:\Xcalibur\...\Erzina D126_200715102840 7/16/2020 9:00:59 AM D126
NSI pos MeCN 1%HFo
Erzina D126_200715102840 #18-30 RT: 0.47-0.80 AV: 13 NL: 3.60E6
T: FTMS + p NSI Full ms [150.00-2000.00]

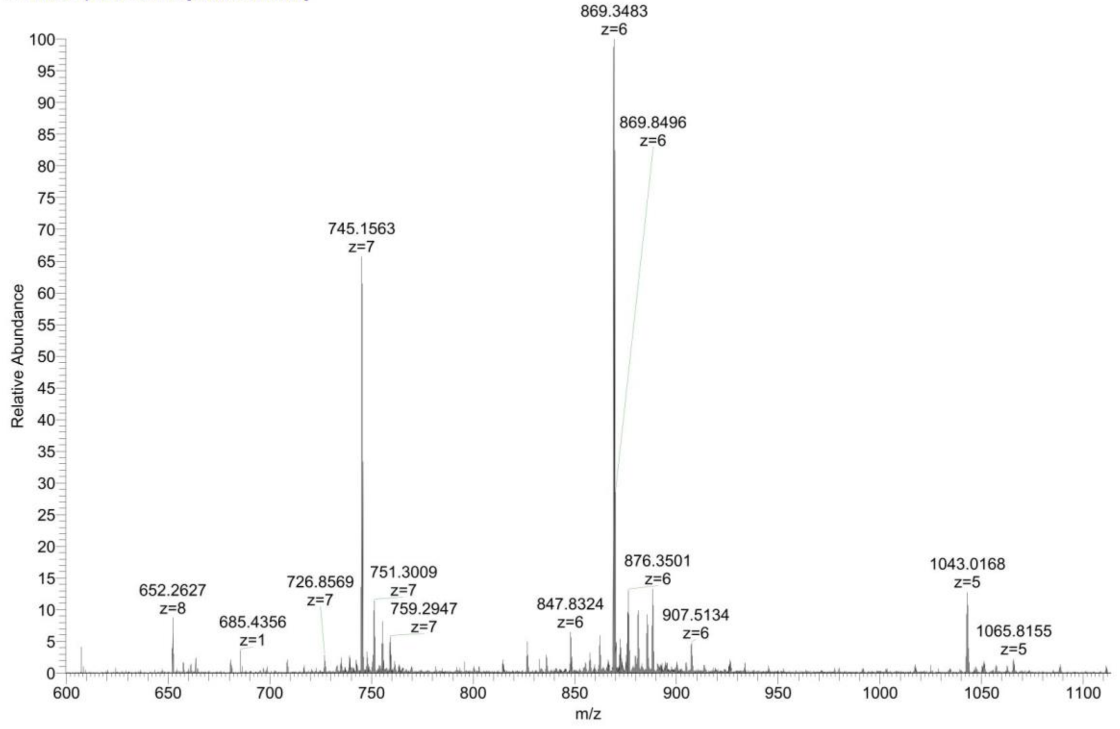

Supplement: Supplementary file 1 — Supporting Information [file ANIE-60-26403-s002.pdf]
